# Proceedings of Réanimation 2019, the French Intensive Care Society International Congress

**DOI:** 10.1186/s13613-018-0474-7

**Published:** 2019-03-29

## Oral communications Oral communications: Physiotherapists

### COK-1 Bench assessment of the effect of a collapsible tube on the efficacy of a mechanical insufflation-exsufflation device

#### Romain Lachal (*speaker*)

##### Réanimation médicale, Hôpital de la Croix-Rousse, Hospices Civils de Lyon, Lyon, FRANCE

###### **Correspondence:** Romain Lachal - romain.lachal@gmail.com

*Annals of Intensive Care* 2019, **9(Suppl 1)**:COK-1

**Introduction**: Mechanical Insufflation-Exsufflation (MI-E) by using a specific device is commonly used to increase weak cough, as in patients with chronic neuromuscular weakness or in intensive care unit (ICU) patients with ICU-acquired neuro-myopathy. The assessment of the efficacy of MI-E device is commonly done by measuring peak cough flow (PCF). Upper airways collapse is frequently associated with neuromuscular disease and may compromise MI-E efficacy. Tracheomalacia is another disease that may impede PCF to increase with MI-E device. The goal of present study was to carry out a bench study to assess the effect of MI-E on PCF with and without the presence of a collapsible tube. Our hypothesis was that PCF was lower with than without collapsible tube.

**Patients and methods**: We used a lung simulator (TTL Michigan Instruments) with adjustable compliance (C) and resistance (R) to which a MI-E (CoughAssist E70, Philips-Respironics) was attached, with or without a latex collapsible tube. Flow and pressure were proximal to the lung simulator. Six C-R combinations were tested, each with and without the collapsible tube. For each C-R combination, we set ± 30, ± 40 and ± 50 cmH2O inspiratory expiratory pressure at the MI-E device. MI-E device was set in automatic mode with inspiratory time of 3 s, expiratory time of 3.2 s and pause of 2 s. Each set was recorded by using a data logger (Biopac 150, Biopac inc.) and the last 5 cycles were used for the analysis done by using Acqknowledge software (Biopac inc.). The peak expiratory flow during the first 100 ms after onset of expiration was taken as the surrogate of PCF. The corresponding pressure was also recorded.

**Results**: Contrary to our hypothesis, the peak expiratory flow during the first 100 ms of exsufflation phase is higher with than without the collapsible tube in every C-R condition, as shown in figure 1. For the C20R5 condition the effect of the collapsible tube on the intercept (− 0.35 cm H_2_O) was not significant but this was offset by a significant increase in slope (+ 0.12 L s cm H_2_O). For the other conditions, the collapsible tube significantly increased PCF at 30 cm H_2_O expiratory pressure and the gap further increased above this pressure because the slope increased with the collapsible tube.

**Conclusion**: We found that peak expiratory was higher with than without collapsible tube. In vivo measurements in patients should be done to confirm this finding.


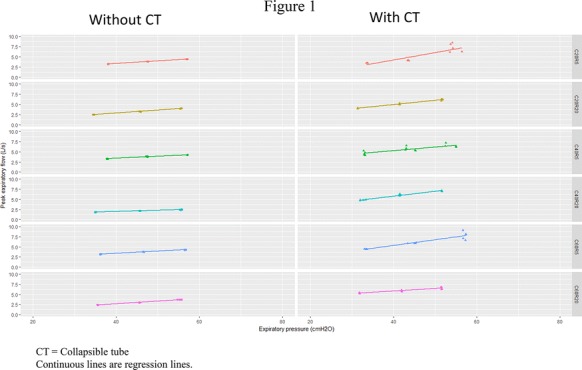


### COK-2 Early verticalization in neurologic intensive care units with a weight suspension system

#### Margrit Ascher (*speaker*), Francisco Miron Duran, Fanny Pradalier, Claire Jourdan, Kevin Chalard, Flora Djanikian, Isabelle Laffont , Pierre-François Perrigault

##### CHU Montpellier, Montpellier, FRANCE

###### **Correspondence:** Margrit Ascher - m-ascher@chu-montpellier.fr

*Annals of Intensive Care* 2019, **9(Suppl 1)**:COK-2

**Introduction**: Background. Current literature and French guidelines recommend early mobilization in Intensive Care Units (ICU), including verticalization and walking. Verticalization for neurologic patients in ICU is challenging because of neurological impairments, risks of falls and of clinical worsening. In the neuro-ICU of Montpellier university hospital, a weight suspension system (LiteGait^®^) is used. Objectives. To study the feasibility and safety of walking with the weight suspension system in a neuroICU. Feasibility involved proportion of patients who benefited from suspension walking, reasons for not using it, physiotherapists’ time required. Safety involved rate of adverse events, changes in vital parameters, pain.

**Patients and methods**: Design. Monocentric, prospective, descriptive study, including all neurogical patient hospitalized for > 48 h in ICU with initial mechanical ventilation from mid-February to mid-September 2018 (excluding deceased patients). Criteria for using suspension walking where respiratory stability without mechanical ventilation (tracheostomy and or oxygen therapy possible), hemodynamic and neurologic stability, sufficient respond to command (head control, testing of one quadriceps > 3 or two quadriceps > 2). Data included general description of patients + clinical status before suspension walking (pain, MRC testing, sitting balance, RASS, hemodynamic and respiratory parameters, medical equipment) + pain, hemodynamic and respiratory parameters during sessions + description of adverse events and consequences + duration of walking.

**Results**: Among 83 patients included (see table for characteristics), 25% benefited from suspension walking during their stay + for 25% of patients, suspension walking was needed but hindered by organization difficulties (such as timetable challenges, early discharge from ICU) + 22% patients could walk without suspension on first verticalization + 20% were too impaired to use suspension walking. A total of 41 suspension walking sessions were performed. Five sessions needed to be interrupted for the five following reasons- pain, hypotension, dizziness, diarrhea and dysfunction of the device. No adverse event had clinical consequences beyond the session. Pain score raised significantly for one patient only (5 points in BPS score). Mean delay between extubation (or tracheotomy) and first suspension walking was 7 days. Mean delay between first suspension walking and first walking without suspension was 8 days.

**Conclusion**: Verticalization with a suspension device in a neurologic ICU is feasible and safe, with a trained and supported team. Obstacles such as medical equipment, language impairments, absence of balance, heavy weight, poor tonicity and participation can be overcome using such a device. Suspension could enable several neurologic ICU patients to walk one week earlier.

### COK-3 Effects of active exercise on red blood cell deformability in critically ill patients

#### Vinciane Scaillet (*speaker*)^1^, Mohera Potvin^1^, Damien Wathelet^1^, Adrien Fievet^2^, Ingrid Leclercq^2^, Karim Zouaoui Boudjeltia^3^, Patrick Biston ^7^, Michael Piagnerelli^1^

##### ^1^Service de Kinésithérapie, Charleroi, BELGIUM; ^2^Ecole de Kinésithérapie-HEPH-Condorcet, Charleroi, BELGIUM; ^3^Laboratoire de Médecine Expérimentale. ULB 222 Unit., Charleroi, BELGIUM; ^4^Réanimation, Charleroi, Charleroi

###### **Correspondence:** Vinciane Scaillet - vinciane.scaillet@chu-charleroi.be

*Annals of Intensive Care* 2019, **9(Suppl 1)**:COK-3

**Introduction**: Early mobilization is recommended in critically ill patients to limit the rapid decrease in muscle mass and function. Several methods, in addition of mobilization are used including passive active in-bed cycling and muscle electrostimulation (ESM). This latter could improve the muscle microcirculation assessed by tissue oxygen saturation and red blood cell (RBC) deformability in untrained athletes by a RBC-nitric oxide production mechanism. In a monocentric, randomized trial, we investigated in critically ill patients, the effects of a passive active exercise alone or associated with a daily ESM or with a daily in-bed cycling sessions on RBC deformability.

**Patients and methods**: Patients were randomized during the first 48 h of ICU admission in three groups- passive and or active mobilization (M) twice a day or M once a day associated with a daily session of 30-min legs passive active in-bed cycling (CYCLE) or M once a day associated with a daily session of 30-min legs ESM (ESM). Demographic data, PaO2 FiO2 and lactate levels were recorded before and after the session. RBC deformability was assessed ex vivo, before and after exercise, by the Laser-assisted Optical Rotational Cell Analyzer (LORCA, Mechatronics Instruments BV, AN Zwaag, Netherlands) with the elongation index (EI) in relation to the shear stress (0.3–50 Pa) applied on the RBC membrane. Data were expressed by the median value 25–75% of delta EI (EI after minus EI before exercise) and compared by Kruskal–Wallis one way test. A p value < 0.05 was considered as statistically significant.

**Results**: 10 patients in each group were included. PaO2 FiO2 (before- 318 (280–381) versus 310 (246–376) after + p = 0.46) and lactate levels (1.3(0.8–2.0) versus 1.4(1.0–2.0) mmol/L L + p = 0.65) were not modified whatever the type of exercise. Only, in the ESM group, delta EI was significantly decreased for several low shear stress (0.76, 1.21 and 1.93 Pa) compared to the two other groups.

**Conclusion**: Association of active passive mobilization and ESM alter at least for low shear stress, the RBC deformability in critically ill patients. Effects on long term treatment by this technique on RBC rheology and on muscle metabolism need to be investigated.

### COK-4 Neurogenic para-osteoarthropathy in severe brain injury in the emergency department of CHU Oran Emergencies (Algeria)

#### Youcef Oubadi (*speaker*)^1^, Soumia Benbernou^2^, Khalida Bouyacoub^1^, Nabil Ghomari^1^, Abdelkader Azza^1^, Houria Pr Djebli France^2^

##### ^1^Centre Hospitalo Universitaire d’Oran, Oran, ALGERIA; ^2^Faculté de médecine, Oran, ALGERIA

###### **Correspondence:** Youcef Oubadi - youcefoubadi2@gmail.com

*Annals of Intensive Care* 2019, **9(Suppl 1)**:COK-4

**Introduction**: Neurogenic para-osteoarthropathy (NOPO) is a common complication after severe head trauma, and can be complicated by joint damage ranging from amplitude limitation to ankylosis. This complication is frequently encountered in the unit. Objectives-Determine the frequency of NOPOs in the Emergency Resuscitation Department Establish a prevention protocol established by the physiotherapists of the unit.

**Patients and methods**: The exposed work was carried out at the URC resuscitation unit of CHUOran. This is a prospective study spread over two years (2015 and 2017) involving 76 patients. The study population consists of severe head injuries (GCT) with a Glasgow score of 8 or less. We studied the time of onset, the affected joint, the degree of limitation + risk factors.

**Results**: Of the 76 TCG patients, 29 patients presented with NOPO. The onset time was an average of 14 days with extremes ranging from 10 to 20 days. The elbow was the most affected joint with knee involvement in some cases. In this series the risk factors favoring the onset of NOPO were- severity of neurological lesions, periarticular lymphoedema, arterial compression of blood pressure cuffs, duration of sedation, type of drug used.

**Conclusion**: The NOPO is a complication occurring at the TCG, it causes functional sequelae that will jeopardize its socio-professional reintegration that only prevention will prevent.

### COK-5 Standing the ARDS patient

#### Guillaume Fossat (*speaker*), Emmanuelle Desmalles

##### CHR, Orléans, FRANCE

###### **Correspondence:** Guillaume Fossat - guillaume.fossat@chr-orleans.fr

*Annals of Intensive Care* 2019, **9(Suppl 1)**:COK-5

**Introduction**: Standing the ICU patient has become a routine care in early mobility programs. In the awake patient, stand can improve minute ventilation, paO2 and awareness. Undesirable effect can be a decreased in Arterial Pressure. To perform stand position in the ICU patient the physiotherapist needs to move him out of the ICU bed. Recently, standing position can be performed directly in the bed. In ARDS patient, positioning is one of the major component of the care. Upright and prone positioning showed many benefits. Prone decreased the mortality rate up to 50% in ARDS patients. Upright position improves the oxygenation effects in ARDS patients. Instead, prone had several adverse effects such as pressure ulcer, endotracheal tube obstruction, and ventilator acquired pneumonia.

**Patients and methods**: In order to decrease the adverse effect of prone positioning we proposed to evaluate whether standing position is safe and can produced the same oxygenation effects as the prone positioning. The standing is performed directly in a dedicated ICU bed, in severe and moderate ADRS patients according to the BERLIN definition. To prevent hypotension, we use an anti shock pants that can be inflate up to 40 mmHg. Standing is performed on a multiple step angulation program, based on the clinical patient responses during 15 min each step-25° => 40° => 55° => 40° => 25° Arterial blood sample is performed before and at the completion of the technique. Static compliance is calculated at each angulation step.

**Results**: For now we only performed one Stand in ARDS patient. No adverse event occurred during the technique. The SpO2, the Blood Pressure remain stable and the anti-shock pants was no inflate. The heart rate rise up from 112 BPM to 132 BPM. The static compliance was stable between the begin and the end (25.00 and 26.92). The P F ratio increase from 171 to 211 at the end of the manoeuver.

**Discussion**: Upright position was already evaluated in some studies, it shows benefits only in responders patient during ARDS. Whereas upright is not standing, because the real incline angle during upright is 26°. With this technique, the patient can be stand for real on his feet, in a posture that is similar as the human stand position.

**Conclusion**: This technique appears to be safe and can produce positive effects on the P F ratio without change in static compliance during ARDS Volume Controlled Ventilation. Further patients need to test this technique to prove real effects.

### COK-6 Aerodigestive tract ultrasound imaging- principles and interests for physsiotherapy

#### Carlos Diaz Lopez (*speaker*)^1^, Aymeric Le Neindre^2^

##### ^1^Hôpital Forcilles, Férolles-Attilly, France; ^2^Hôpital Foch, Suresnes, FRANCE

###### **Correspondence:** Carlos Diaz Lopez - carlosdl90@hotmail.com

*Annals of Intensive Care* 2019, **9(Suppl 1)**:COK-6

**Introduction**: The clinical assessment of swallowing disorders in ICU has many limitations in its reliability and accuracy. The reference standard imaging techniques, such as fluoroscopy, still rely their analysis on the operator’s subjective conclusion (see table 1) (1) (2). In this context, ultrasound imaging, that can be performed at the patient’s bedside, is a non-invasive tool allowing the evaluation of the main structures involved in all the swallowing phases and may be a promising tool to dysphagia assessment (3).

**Patients and methods**: This is a narrative review on the interests of ultrasound imaging for swallowing assessment as well as on its perspective of use in physiotherapy. The figures and practical descriptions are the result of the authors’ everyday use of ultrasound imaging.

**Results**: Ultrasound imaging allows a quantitative and qualitative examination of the majority of the swallowing structures, such as tongue, oropharyngeal muscles, larynx, and upper esophageal sphincter (UES). It assesses the tongue kinetics, looking for abnormal movements, and the contractility and thickness of the oropharyngeal muscles, which enables to assess a potential dysfunction (4) (5). Physiotherapists may also follow the larynx movements (in coronal and sagittal planes) evaluating the displacement of the hyoid bone (6). A decrease in the hyoid bone displacement and contraction of the tongue may explain a dysphagia (7). Finally, ultrasound imaging can measure the displacement and the opening-closing diameters of the upper esophagus sphincter (UES), allowing to detection of dysphagia related to UES disorders.

**Conclusion**: Ultrasound imaging of the aerodigestive tract may improve the quantitative and qualitative examination of the swallowing structures. The use of this method requires a deep understanding of the ultrasound imaging principles and of the swallowing physiopathology in order to be able to benefit from its potential. Ultrasound imaging seems to be a promising tool for physiotherapy, used as an outcome in clinical research or during clinical practice in order to improve the diagnosis of the dysfunctions related to dysphagia.


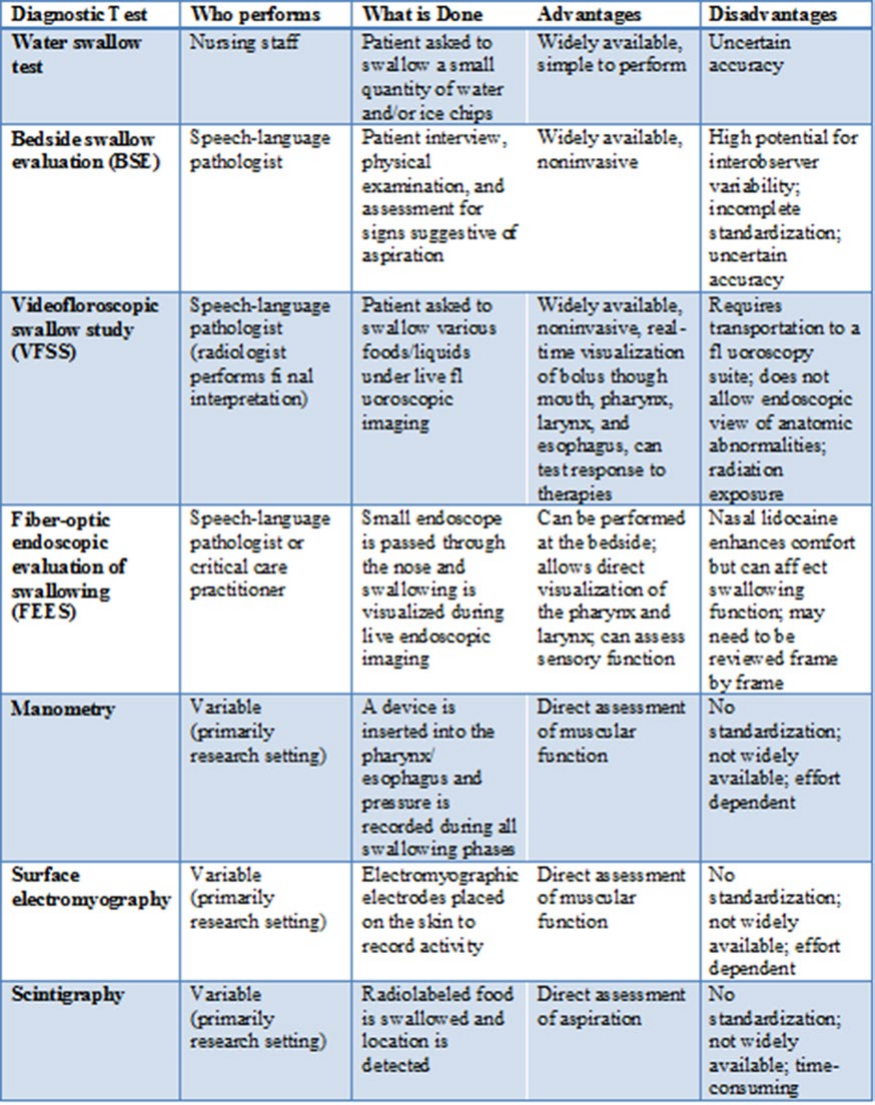


## Oral communications: Doctors

### CO-01 Impact of mechanical ventilation setting on the occurrence of acute kidney injury in ICU patients - insights from the MIMIC-III database

#### Guillaume Geri (*speaker*)^1^, Loic Ferrer^2^, Nam Tran^3^, Matthieu Jamme^4^, Leo Anthony Celi^5^, Joon Lee^6^, Antoine Vieillard-Baron ^1^

##### ^1^Ambroise Paré Hospital, APHP, Boulogne-Billancourt, FRANCE; ^2^Curie Intitute, Paris, FRANCE; ^3^Waterloo University, Waterloo, CANADA; ^4^Versailles Saint Quentin University, Versailles, FRANCE; ^5^Massachusetts Institute of Technology, Cambridge, UNITED STATES; ^6^University of Waterloo, Waterloo, CANADA

###### **Correspondence:** Guillaume Geri - guillaume.geri@aphp.fr

*Annals of Intensive Care* 2019, **9(Suppl 1)**:CO-01

**Introduction**: Mechanical ventilation in ICU patients may induce acute kidney injury (AKI). We aimed to describe the effect of mechanical ventilation (MV) settings, on AKI worsening, as well as the potential role of mean perfusion pressure (MPP).

**Patients and methods**: We included from the MIMIC-III database adult patients admitted for the first time in the ICU. We excluded patients with known chronic kidney disease, no KDIGO information and who suffered KDIGO3 AKI at day-1. The main outcome was one-KDIGO category AKI worsening (compared to the day before) and included as a categorical variable - discharged alive without AKI worsening and death before AKI worsening. We used a multinomial logistic regression at day 1 and day 2 according to a landmark-approach, with a two-days sliding perspective.

**Results**: 26,884 patients met the inclusion criteria (15,046 male, 56.0% + median age 65 [iqr 52, 78]). ICU and hospital mortality were 7.4 and 10.7%, respectively. Between day 1 and day 3, 529 patients died without AKI worsening, 11,230 were discharged alive and 1,656 suffered an AKI worsening. Between day 2 and day 4, 128 patients suffered an AKI worsening, 5,686 were discharged alive and 313 died. In multivariable analysis, using the “no MV” modality as a reference (n = 19,666), MV at day 1 was associated with AKI worsening at day 3 (relative risk ratio [RRR] 15.7 [7.7, 32], 23.2 [17.6, 30.6] and 55.6 [29, 106.4] for MV with PEEP < 5 (n = 283), PEEP between 5 and 8 (n = 4,153) and PEEP > 8cmH2O (n = 1,134)). **RRR of AKI worsening at day-4 were 2.3 [0.3, 19.4], 13.3 [5.8, 30.6] and 44.9 [17.2, 117.2] for MV with PEEP < 5 (n = 254), PEEP between 5 and 8 (n = 2,751) and PEEP > 8cmH2O (n = 1,088) at day 2 compared to no MV (n = 16,397). Hemodynamic parameters are shown on Figure. MPP significantly differed across MV groups (67, 70, 68 and 64 mmHg in patients receiving no MV, MV with PEEP < 5, PEEP 5–8 and PEEP > 8cmH2O, p < 0.001).

**Conclusion**: Mechanical ventilation was associated with AKI worsening in an increasing PEEP dependent manner at the early phase of ICU management in a large cohort of patients. Interaction between MPP and mechanical ventilation should be explored further.


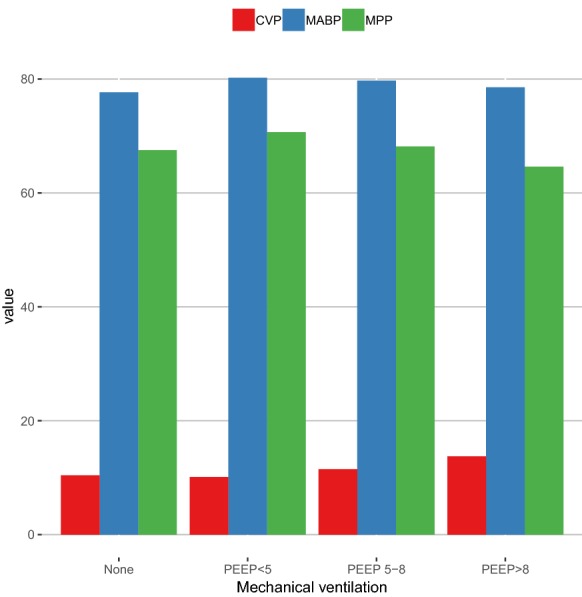


### CO-02 Transvenous Renal Biopsy of Critically Ill Patients- Safety and Diagnostic Yield

#### Marc Pineton de Chambrun (*speaker*)^1^, Philippe Cluzel^2^, Isabelle Brocheriou^3^, Nicolas Brechot^1^, Guillaume Hekimian^1^, Guillaume Franchineau^1^, Côme Bureau ^1^, Simon Bourcier^1^, Ania Nieszkowska^1^, Loic Le Guennec^4^, Zahir Amoura^4^, Alexis Mathian^4^, Matthieu Schmidt^1^, Alain Combes^1^, Charles-Edouard Luyt^1^

##### ^1^Service de médecine intensive réanimation, ICAN, Hôpital La Pitié-Salpêtrière, Sorbonne Université, APHP, Paris, France., Paris, FRANCE; ^2^Hôpital La Pitié-Salpêtrière, Sorbonne Université, APHP, Paris, FRANCE; ^3^Service d’anatomopathologie, Hôpital La Pitié-Salpêtrière, Sorbonne Université, APHP, Paris, France, Paris, FRANCE; ^4^Service de médecine interne 2, Hôpital La Pitié-Salpêtrière, Sorbonne Université, APHP, Paris, France, Paris, FRANCE

###### **Correspondence:** Marc Pineton de Chambrun - marc.dechambrun@gmail.com

*Annals of Intensive Care* 2019, **9(Suppl 1)**:CO-02

**Introduction**: Introduction- Transvenous renal biopsy is an alternative way to obtain kidney samples from patients with bleeding-risk factors (e.g., antiplatelet therapy, anticoagulation or coagulation disorders…). This study was undertaken to determine the safety and diagnostic yield of transvenous renal biopsy of critically ill patients.

**Patients and methods**: Patients and Methods - Monocenter, retrospective, observational cohort study in a 26-bed French tertiary ICU. All patients undergoing in-ICU transvenous renal biopsy between January 2002 and February 2018 were included.

**Results**: Results- Eighty patients (male female sex ratio, 0.95 + mean ± SD age, 47.3 ± 18.3 years) were included. A histologic diagnosis was obtained for 77 (96.3%) patients, with acute tubular necrosis being the most frequent- 23 (29.9%). A potentially treatable cause was found for 47 (58.7%) patients. The numbers of patients with 0, 1, 2 or 3 factors (i.e., antiplatelet therapy, thrombopenia (< 150 G L) and preventive or curative anticoagulation) at the time of the biopsy were, respectively- seven (8.8%), 37 (46.2%), 31 (38.7%) and five (6.3%). Four (5%) and two (2.5%) patients, respectively, had renal hematoma and macroscopic hematuria + none required any specific treatment. Six (7.5%) patients died in-ICU and 90-day mortality was 8 80 (10%). No death was related to transvenous renal biopsy and median biopsy-to-death interval was 38 [IQR, 19.7 + 86] days.

**Conclusion**: Conclusions- Based on this cohort of ICU patients with acute renal failure, transvenous renal biopsy was safe and obtained a high diagnostic yield for these critically ill patients, even in the presence of multiple bleeding risk factors.

### CO-03 The incidence of chronic kidney disease three years after non-severe acute kidney injury in critically ill patients - a cohort study

#### Arthur Orieux (*speaker*), Sébastien Rubin, Benjamin Clouzeau, Claire Rigothier, Christian Combe, Didier Gruson, Alexandre Boyer

##### CHU, Bordeaux, FRANCE

###### **Correspondence:** Arthur Orieux - arthur.orieux@chu-bordeaux.fr

*Annals of Intensive Care* 2019, **9(Suppl 1)**:CO-03

**Introduction**: The risk of chronic kidney disease (CKD) following severe acute kidney injury (AKI) in critically ill patients is well documented. However, the long-term risk of CKD for less severe AKI in critically ill patients has never been assessed.

**Patients and methods**: This prospective single-center intensive care unit (ICU) observational 3-years follow-up study was carried out from 2013 to 2015 in Bordeaux (France). All patients with both severe (KDIGO 3) and non-severe AKI (KDIGO stage 1, 2) were enrolled. Patients with prior CKD were excluded. The primary outcome was the 3-years prevalence of CKD (defined by estimated glomerular filtration rate lower than 60 mL/min 1.73m2) in the non-severe AKI group. Secondary outcomes were risk factors for 3-years CKD, renal survival during follow-up and to identify the proportion among CKD patients followed by a nephrologist. Renal recovery was defined as return of creatinine at ICU discharge to < 26.5 µmol L above baseline.

**Results**: We screened 304 patients and after exclusion of 72 because of prior CKD 232 patients were enrolled. Severe AKI was observed in 120 (52%) and non-severe AKI in 112. At the end of the follow-up and after exclusion of patients who died, 41 and 30 patients remained in the non-severe and severe AKI group respectively. The global prevalence of CKD was 23 71 (32%). It was higher in the severe vs. non-severe AKI group (14 30 (47%) vs. 9 41 (22%) + p < 0.05) (table 1). Independent risk factors for 3-years CKD were diabetes (OR = 5.7 [1.5–21.5]) and absence of renal recovery at ICU discharge (OR = 14.2 [2.9–69.1]). Eleven out of 23 (48%) CKD patients were followed by a nephrologist.

**Discussion**: This study is the first to give the prevalence of CKD 3 years after non-severe AKI in critically-ill patients. No study has focused on stage 1–2 AKI in critically ill patients and only few studies have explored stage 1- 2 AKI in non-critically ill patients. The high prevalence of CKD at 3 years and the limited amount of CKD patients followed by a nephrologist confirm the need for accurate follow-up planning after ICU discharge in all patients diagnosed with AKI.

**Conclusion**: The risk of developing CKD at 3 years after non-severe AKI, despite lower than after severe AKI, remains high and is underestimated.


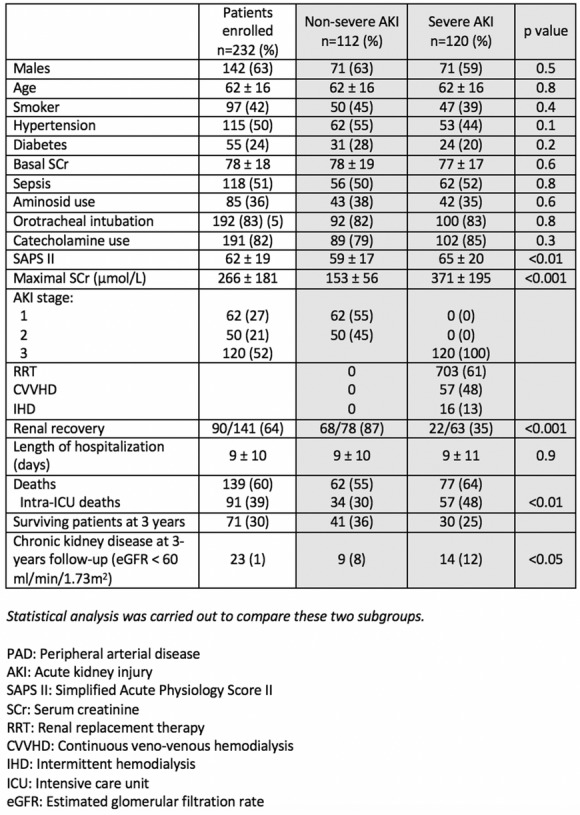


### CO-04 Initiation strategies for renal replacement therapy for acute kidney injury and long-term survival- a follow-up from the AKIKI randomized controlled trial

#### Khalil Chaïbi (*speaker*)^1^, Frank Ehooman^2^, David Hajage^3^, Frederique Schortgen^4^, Laurent Martin-Lefevre^5^, Charles Verney^6^, Bertrand Pons ^7^, Eric Boulet^8^, Alexandre Boyer^9^, Guillaume Chevrel^10^, Nicolas Lerolle^11^, Dorothée Carpentier^12^, Nicolas De Prost^13^, Alexandre Lautrette^14^, Anne Bretagnol^15^, Julien Mayaux^16^, Saad Nseir^17^, Bruno Megarbane^18^, Marina Thirion^19^, Jean-Marie Forel^20^, Julien Maizel^21^, Yonis Hodane^22^, Philippe Markowicz^23^

##### ^1^Avicenne Hospital, Bobigny, FRANCE; ^2^INSERM, Paris, FRANCE; ^3^Hôpital La Pitié Salpêtrière, Département Biostatistique, Santé Publique et Information Mé, Paris, FRANCE; ^4^CHU Henri Mondor, Creteil, FRANCE; ^5^Réanimation Médico-Chirurgicale, Centre Hospitalier Général, La Roche-Sur-Yon, FRANCE; ^6^Hopital Louis Mourier, Colombes, FRANCE; ^7^CHU Les Abymes Guadeloupe, Les Abymes, FRANCE; ^8^CH René Dubos, Pontoise, FRANCE; ^9^Hôpital Pellegrin-Tripode, Bordeaux, Bordeaux, FRANCE; ^10^CH Sud Francilien, Corbeil-Evry, Corbeil-Evry, FRANCE; ^11^CHU, Angers, FRANCE; ^12^CHU, Rouen, FRANCE; ^13^CHU Henri Mondor, Créteil, FRANCE; ^14^CHU Gabriel Montpied, Clermond-Ferrand, FRANCE; ^15^CHR, Orléans, FRANCE; ^16^Hôpital La Pitié Salpetrière, Paris, FRANCE; ^17^Hôpital R. Salengro, CHU, Lille, FRANCE; ^18^Hopital Lariboisière, Paris, FRANCE; ^19^CH Victor Dupouy, Argenteuil, FRANCE; ^20^Hopital Nord, Marseille, FRANCE; ^21^CHU, Amiens, FRANCE; ^22^CHU, Lyon, FRANCE; ^23^CH, Chollet, FRANCE

###### **Correspondence:** Khalil Chaïbi - khalilchaibi@gmail.com

*Annals of Intensive Care* 2019, **9(Suppl 1)**:CO-04

**Introduction**: Epidemiological and experimental studies showed a link between acute kidney injury (AKI) and long-term outcome such as mortality and chronic kidney disease (CKD). Whether the timing of renal replacement therapy (RRT) during AKI eventually affects these outcomes is unknown. This study analyzes the long-term outcome of patients included in the Artificial Kidney Initiation in Kidney Injury (AKIKI) according to RRT initiation strategy.

**Patients and methods**: The AKIKI trial was a prospective, multicenter, open-label, two-arm randomized controlled trial that compared two RRT initiation strategies in patients receiving catecholamines and or mechanical ventilation and presenting with severe KDIGO3 AKI and no potentially life-threatening condition (NEJM, 2016 + 375-122-133). In the early strategy, RRT was started within 6 h after randomization criteria. In the delayed one, RRT was initiated only if one or more following criteria occurred- severe hyperkalemia, severe acidosis, severe pulmonary edema due to fluid overload resulting in severe hypoxemia, oligo-anuria > 72 h or serum urea concentration > 40 mmol/L. We assessed long-term (> 2 years) outcomes of the patients who survived 60 days after randomization. We collected vital status, last renal function value and health-related quality of life. We used a Kaplan–Meier estimator and a log rank test to analyze long-term survival according to randomization group.

**Results**: The median follow-up was 2.5 years (confidence interval [CI], 2.3 to 2.7 years). The early strategy group initially included 311 patients of whom 151 were alive 60 days after randomization and 145 were still alive at 2 years. The delayed strategy group initially included 308 patients of whom 153 were alive 60 days after randomization and 145 were still alive at 2 years (p = 0.94 for comparison between groups). Kaplan–Meier curves (see Figure 1) were superposed (*p* value- 0.79). The analyses of long-term renal function and health related quality of life are still in progress.

**Conclusion**: Although initial mortality of AKI in critically ill patients was high (49%), patients who survived the acute episode had a good vital prognosis at 2 years with only 6% additional deaths. Long-term death rate was not influenced by the initial RRT initiation strategy. Data on renal function and health quality will be available at the time of presentation.


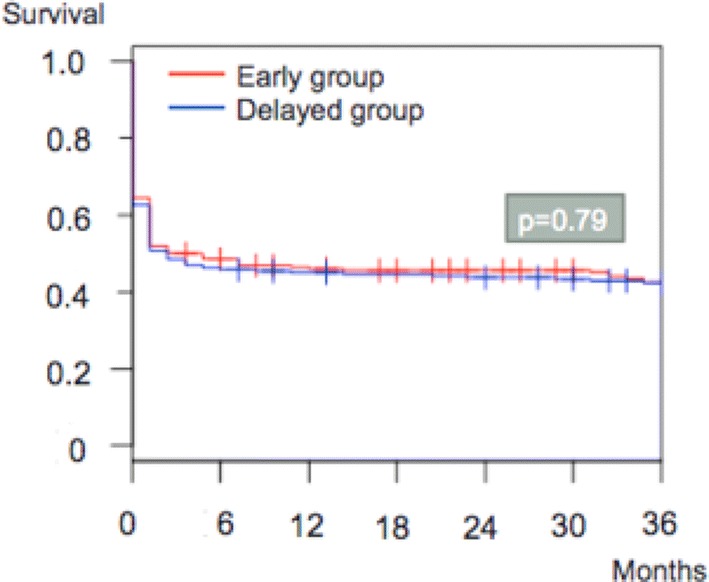


### CO-05 Epidemiology of Post-influenza Bacterial Pneumonia Due to a Panton-Valentine Leukocidin Positive Staphylococcus aureus in Intensive Care Unit- a retrospective nationwide study

#### Audrey Jacquot (*speaker*)^1^, Bruno Levy^1^, Charles Edouard Luyt^2^, Charles Vidal^3^, Sami Hraiech^4^, Jean-Pierre Quenot^5^, Lara Zafrani ^6^, Francis Schneider^7^, Pierre Kalfon^8^, Vincent Piriou^19^, Saadalla Nseir^10^, Paul Jaubert^11^, Jonathan Messika^12^, Muriel Fartoukh^13^, Xavier Valette^14^, Alexandre Lautrette^15^, Antoine Marchalot^16^, Nicolas Terzi^17^, Guillaume Schnell^18^, Charlène Le Moal^19^, Emmanuel Novy^20^, Jérémie Lemarié^1^, Arnaud Galbois^21^

##### ^1^CHU, Nancy, FRANCE; ^2^APHP Pitié-Salpétrière, Paris, FRANCE; ^3^CHU Felix Guyon, La Réunion, FRANCE; ^4^APHM Hôpital Nord, Marseille, FRANCE; ^5^CHU, Dijon, FRANCE; ^6^APHP Saint Louis, Paris, Paris; ^7^CHU Hautepierre, Strasbourg, FRANCE; ^8^CH, Chartres, FRANCE; ^9^CHU Lyon Sud, Pierre Bénite, FRANCE; ^10^CHU, Lille, FRANCE; ^11^APHP Cochin, Paris, FRANCE; ^12^APHP Colombes, Colombes, FRANCE; ^13^APHP Tenon, Paris, FRANCE; ^14^CHU, Caen, FRANCE; ^15^CHU, Clermont-Ferrand, FRANCE; ^16^CH, Dieppe, FRANCE; ^17^CH Grenoble, La Tronche, FRANCE; ^18^CHU, Le Havre, FRANCE; ^19^CHU, Le Mans, FRANCE; ^20^CHU Nancy, Vandoeuvre-Les-Nancy, FRANCE; ^21^Hôpital privé Claude Galien, Quincy-sous-Sénart, FRANCE

###### **Correspondence:** Audrey Jacquot - audreyjacquot@hotmail.com

*Annals of Intensive Care* 2019, **9(Suppl 1)**:CO-05

**Introduction**: Superinfection of Influenza pneumonia with Staphylococcus aureus is a well-known adverse event. Conversely, published data on the epidemiology of superinfection with a Staphylococcus aureus producing the Panton-Valentine Leukocidin (SA PVL) remains scarse. This study evaluates the mortality and the epidemiology of this specific complication of influenza pneumonia in Intensive Care Unit (ICU).

**Patients and methods**: We conducted a multicenter retrospective epidemiological study over a period from January 2009 to December 2017. Were included patients admitted to ICU for SA PVL co infecting influenza pneumonia. The primary endpoint was mortality in ICU. Secondary endpoints included demographic, clinical characteristics of this population, description of complications during ICU stay, microbiological and therapeutic data.

**Results**: Of the 25 participating centres, 1970 patients admitted to ICU for influenza pneumonia were screened during the inclusion period. Prevalence of influenza pneumonia co infected with SA PVL was 1.1% (22 patients). Patients are young and had neither comorbidity, nor risk factors for severe influenza. The overall mortality in ICU of the cohort was 54.5% (12 patients). On admission to intensive care, patients were leucopenic (1.9 G L [0.7–4.8]) and 17 (81%) presented a severe ARDS (PaO2 FiO2 104 [66–320]) requiring vvECMO in 10 patients (45.5%). The ICU length of stay was 39.0 days [13.0–53.0] for survivors and 3.5 days [1.0–6.5] for non-survivors. No difference of anti-viral or antibiotic treatment between the two groups was recorded.

**Conclusion**: SA PVL superinfection of influenza pneumonia is rare but extremely severe complication, with a high and early mortality. Patients most often presented with severe ARDS frequently requiring vvECMO.

 Declarations: Any conflict of interest for this work.

### CO-06 Invasive pulmonary aspergillosis is a rare complication in critically ill patients with influenza

#### Anne Coste (*speaker*)^1^, Aurélien Frérou^2^, Jean Morin^3^, François-Xavier Blanc^4^, Jean Reignier^5^, Gilles Névez^6^, Patrice Le Pape ^7^, Jean-Marie Tonnelier^8^, Cédric Bretonnière^9^, Cécile Aubron^10^

##### ^1^Service de maladies infectieuses, Brest, FRANCE; ^2^CHU Pontchaillou, Rennes, FRANCE; ^3^CHU - Service de pneumologie, Nantes, FRANCE; ^4^Service de pneumologie-CHU, Nantes, FRANCE; ^5^Réanimation médicale, Nantes, FRANCE; ^6^Département de mycologie parasitologie, Brest, FRANCE; ^7^Nantes University Hospital, Nantes, Nantes; ^8^Réanimation médicale, Brest, FRANCE; ^9^Réanimation médicale, Nantes, FRANCE; ^10^Réanimation médicale, Brest, FRANCE

###### **Correspondence:** Anne Coste - annecoste89@gmail.com

*Annals of Intensive Care* 2019, **9(Suppl 1)**:CO-06

**Introduction**: Some studies show a high incidence of invasive pulmonary aspergillosis (IPA) in patients with severe influenza, and suggest to assess benefits of an antifungal prophylaxis. However, those studies come from the same area and lack of external validity. We aimed to measure the incidence of invasive pulmonary aspergillosis (IPA) and aspergillosis colonization in patients with severe influenza admitted to the intensive care unit (ICU).

**Patients and methods**: This retrospective multicenter cohort study recruited all patients with influenza admitted to four ICU in three French tertiary hospitals between September 1, 2009 and April 30, 2018. Patients were adults and had a confirmed influenza infection based on a positive airway PCR test. Patients with an Aspergillus-positive lower respiratory tract specimen culture (patients with Aspergillus) were diagnosed with Aspergillus colonization and invasive pulmonary aspergillosis (putative or proven) according to AspICU criteria. A multivariate logistic regression was performed to determine the factors associated with invasive pulmonary aspergillosis (IPA) and aspergillosis colonization.

**Results**: Four hundred and ninety-one patients were included. Three hundred and fifty-three patients (78.3%) were infected with Influenza A and 98 patients (21.7%) with Influenza B. Mean Simplified Acute Physiology Score II was 43.9 (SD = 20.1) and 97 patients (19.6%) died in ICU. Three hundred and fifty-six patients (72.2%) were under invasive mechanical ventilation and 32 patients (6.5%) underwent extra-corporeal membrane oxygenation. Twenty-six patients (5.2%) had an Aspergillus-positive lower respiratory tract specimen culture, including 12 patients (2.4%) diagnosed with IPA according to AspICU criteria. In multivariate analysis, factors associated with Aspergillosis-positive lower respiratory tract specimen culture were liver cirrhosis, haematological malignancy, use of vasopressors and H1N1 influenza (Table). Mortality rate did not significantly differ between patients with and without Aspergillus (26.9% and 19.2%, p = 0.32).

**Conclusion**: In this large French cohort study of patients with severe influenza, IPA was diagnosed in 2.4% of critically ill patients with Influenza infection. Prospective research is warranted to confirm our findings.

**Table:** Table 1. Factors associated with Aspergillosis-positive lower respiratory tract specimen culture in multivariate analysis

Factor	Adjusted odds ratios	95% Confidence interval	p-value
Liver cirrhosis	12.36	[3.95–37.77]	9.4 × 10^−6^
Haematological malignancy	5.05	[1.72–14.21]	2.3 × 10^−3^
Vasopressors	3.57	[1.31–11.52]	0.019
H1N1 *Influenza* infection	3.32	[1.19–8.77]	0.017

### CO-07 Influenza virus infection is associated to global and persistent immunosuppression in ICU patients

#### Ana Catalina Hernandez Padilla (*speaker*)^1^, Robin Jeannet^2^, Thomas Lafon^3^, Olivier Barraud^4^, Sébastien Hantz^4^, Philippe Vignon^5^, Bruno François ^5^, Thomas Daix^6^

##### ^1^Limoges, FRANCE; ^2^CNRS UMR 7276 Inserm U1262, CHU Dupuytren, Limoges, FRANCE; ^3^Inserm CIC 1435 Service d’Accueil des Urgences, CHU Dupuytren, Limoges, FRANCE; ^4^Inserm UMR 1092 Laboratoire de Bactériologie-Virologie-Hygiène, Université de Limoges CHU Dupuytren, Limoges, FRANCE; ^5^Inserm CIC 1435 Réanimation polyvalente Inserm UMR 1092, CHU Dupuytren, Limoges, FRANCE; ^6^Inserm CIC 1435 Réanimation polyvalente, CHU Dupuytren, Limoges, FRANCE

###### **Correspondence:** Ana Catalina Hernandez Padilla - AnaCatalina.HERNANDEZPADILLA@chu-limoges.fr

*Annals of Intensive Care* 2019, **9(Suppl 1)**:CO-07

**Introduction**: Seasonal Influenza virus infection (IVI) is associated to high morbidity and frequent complications. Immune dysregulation secondary to IVI may contribute in both ARDS and overmortality and increase the risk of secondary infections. This study aimed at characterizing the immune profile of ICU patients admitted for IVI.

**Patients and methods**: Prospective, single-center, observational study in immunocompetent adults admitted to the ICU for IVI confirmed by PCR from nasopharyngeal swabs between January and March 2018. Immune-profile was assessed by flow cytometry on peripheral blood at admission and during the first two weeks of ICU stay. Subsets analyzed included- immature (CD16-) and mature (CD16 +) granulocytes, activated monocytes (CD14 + CD16 +), HLA-DR- monocytes (CD14 + HLA-DR-) and T-CD3 + lymphocytes. Bacterial coinfection was defined as presence of positive cultures from samples acquired within standard of care associated to clinical evidence of infection.

**Results**: Thirteen patients (51 ± 14 years old + 9 men) were admitted to ICU (SOFA score 4 ± 3 + APACHE 16 ± 5) with confirmed IVI (Influenza A 69%). Twelve patients required invasive mechanical ventilation, 4 patients had coinfections at admission and 4 had secondary infections between days 8 and 16. All coinfections were of pulmonary origin. Most of the community acquired pneumonia (CAP) cases were related to pneumococcus coinfection (75%), while isolations related to ventilator-associated pneumonia (VAP) were Staphylococcus aureus (n = 2), Staphylococcus epidermidis (n = 1) and Aspergillus fumigatus (n = 1). Four patients (31%) died, all with documented coinfection (CAP n = 2, VAP n = 2). All patients showed T-lymphopenia at admission that persisted until day 7 (0.539 ± 0.501 G L to 0.561 ± 0.095 G L) (Figure 1A). Activated monocytes were also low at admission and trended towards decrease (0.051 ± 0.047 G L to 0.007 ± 0.006 G L) (Figure 1B). Concomitantly, the number of myeloid derived suppressive cells increased over time- HLA-DR- monocytes (0.099 ± 0.097 G L to 0.128 ± 0.118 G L) and immature granulocytes (0.592 ± 0.912 G L to 2.808 ± 2.014 G L) (Figure 1B and C).

**Conclusion**: Patients admitted to ICU for IVI had an immunosuppressive profile characterized by decreased mature active cell subsets with concurrent increase in immature suppressive ones. This immunosuppression lasted over the first week of ICU stay, and could explain the increased risk for secondary infections and its related worst prognosis.


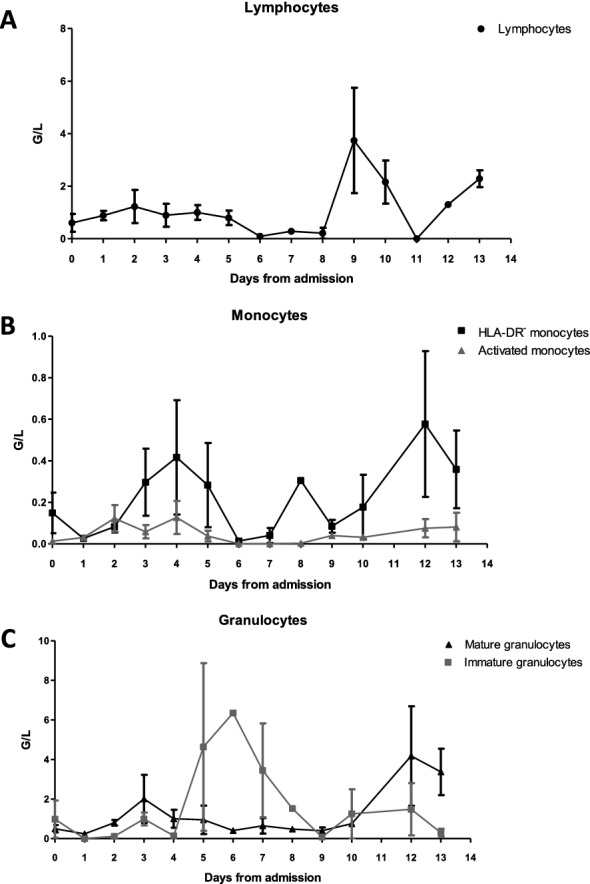


### CO-08 Impact of respiratory viruses in ICU patients with community acquired-pneumonia (CAP)- a one-year retrospective single center study

#### Marion Giry (*speaker*), Marie Gueudin, Déborah Boyer, Adeline Baron, Gaetan Beduneau, Soumaya Skallil, Marie-Anne Melone, Steven Grange, Dorothée Carpentier, Fabienne Tamion, Christophe Girault, Benoît Misset

##### CHU Rouen, Rouen, FRANCE

###### **Correspondence:** Marion Giry - marion.giry7@gmail.com

*Annals of Intensive Care* 2019, **9(Suppl 1)**:CO-08

**Introduction**: Pneumonia is the most frequent community-acquired infection responsible for ICU admission. Multiplex PCR enables early diagnosis of viral infection in daily practice. Few series have been described since this technique has been made available in routine. Our objective was to assess the prevalence and distribution of viruses among ICU patients with CAP and their relationship with severity and outcome.

**Patients and methods**: Retrospective analysis of the consecutive viral multiplex PCR (Eplex™, Genmark Dx) between November 2016 and October 2017 in a French 21 bed medical ICU admitting around 1,000 patients per year. Patients’ nasopharynx was sampled within 72 h following their ICU admission. We selected those patients with a diagnosis of CAP and split them into 4 groups according to causal agent- none, viruses, bacteria, combination of both. Comparisons were made with non-parametric Kruskall-Wallis and Fischer’s exact tests.

**Results**: 223 patients were sampled, of whom 109 had CAP, 38 aspiration or opportunistic pneumonia, 22 non-pulmonary infections, 11 pulmonary edema, 19 exacerbations of chronic lung disease, and 24 other diagnoses. Patients with CAP had the following characteristics- age 59 ± 16 y, male sex 60%, SAPS 2 score 40 ± 18, ICU length of stay (LOS) 8.7 ± 9.0 d, mortality 10%. No infectious agent was found in 32 (29%), a virus in 28 (26%), bacteria in 33 (30%) and both a virus and bacteria in 16 (15%). The main bacteria were S. pneumonia (23%) and H. influenza (6.5%). The main viruses were Rhinovirus Enterovirus (13.8%), Influenzae A (11%) and Parainfluenzae (5.5%). The most frequent virus-bacteria association was S. pneumoniae and Influenzae A (3%) and S. pneumoniae and Rhinovirus Enterovirus (3%). The SAPS 2 score was higher in the mixed group (p = 0.02). The ICU-LOS was 5.2 (no agent), 7.7 (virus), 10.1 (bacteria) and 14.8 (mixed) days respectively (p = 0.04). Mortality was similar among groups.

**Conclusion**: In our ICU population, respiratory viruses were present in 40% of CAP. Patients with mixed infection had higher severity at admission and longer ICU-LOS.

### CO-09 Invasive Pulmonary Aspergillosis in Critically Ill Patients with Hematological Malignancies

#### Emmanuel Pardo (*speaker*)^1^, Virginie Lemiale^2^, Djamel Mokart^3^, Annabelle Stoclin^4^, Anne-SophieMoreau^5^, Lionel Kerhuel^2^, Etienne Ghrenassia ^2^, Laure Calvet^2^, Audrey De Jong^2^, Sandrine Valade^2^, Eric Mariotte^2^, Lara Zafrani^2^, Michael Darmon^2^, Elie Azoulay^2^

##### ^1^CHU Saint-Antoine, Paris, FRANCE; ^2^Hôpital Saint-Louis, Paris, FRANCE; ^3^Institut Paoli-Calmettes, Marseille, FRANCE; ^4^Institut Gustave Roussy, Villejuif, FRANCE; ^5^Hôpital Albert Calmette, Lille, FRANCE

###### **Correspondence:** Emmanuel Pardo - pardo.emmanuel@gmail.com

*Annals of Intensive Care* 2019, **9(Suppl 1)**:CO-09

**Introduction**: Invasive fungal infections remain associated with high mortality rates among patients with hematological malignancy. Recent medical advances in antifungal prophylaxis, diagnostic criteria and treatments of invasive pulmonary aspergillosis (IPA) have been reported. We sought to assess whether these advances translate into change in survival in patients with acute respiratory failure and IPA.

**Patients and methods**: Retrospective, multicenter study performed in four centers. Adult patients with hematological malignancy, proven or probable IPA, and acute respiratory failure requiring ICU between January 1998 and December 2017 were included. Results are reported as n (%) or median (IQR). A cox regression model was used to identify variables independently associated with 6 months survival.

**Results**: Overall, 219 patients were included. 138 (63%) were of male gender and median age was of 55 (IQR 44–64). Acute myeloid leukemia (30.1%) and non-Hodgkin lymphoma (22.8%) were the most frequent malignancies. 134 patients were neutropenic at study inclusion (62%), and 53 were allogeneic stem cell recipients (24.2% + including 64.2% who suffered from Graft-versus-Host Disease) and 22 receiving antifungal prophylaxis (10%). Median SOFA score at admission was 9 [7–12] and median time before introduction of invasive mechanical ventilation was 1 [0–3] day. 154 patients (70.3%) had positive serum galactomannan and 136 (62.1%) a positive culture. ICU and 6-months mortality remained unchanged during the 20-years study period, being respectively 58.4% and 80.1% (Figure 1A). Need for invasive mechanical ventilation, at admission or following failure of non-invasive techniques, was associated with a high mortality rate (Figure 1B). Use of Voriconazole (HR 0.67, IC95 0.48–0.94) and focal radiologic pulmonary infiltrate (HR 0.58, IC95 0.41–0.80) were independently associated with better survival by multivariable analysis adjusted on day-1 SOFA score (HR 1.1, IC95%1.06–1.15).

**Conclusion**: IPA still mostly affects patients with neutropenia and BMT recipients. Case fatality remains high and sustained over time. Voriconazole treatment and focal infiltrate (when compared to diffuse infiltrate) are associated with better survival. Strategies to improve survival in these high-risk patients are warranted.


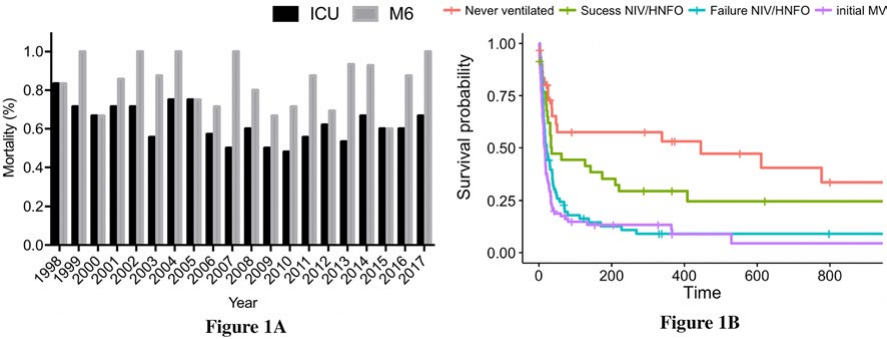


### CO-10 Changes in outcome of critically ill cancer patients over the last decades- Results of a systematic review on individual data

#### Michael Darmon (*speaker*)^1^, Aurélie Bourmaud^2^, Quentin Georges^3^, Marcio Soares^4^, Kyeongman Jeon^5^, Sandra Oeyen^6^, Chin Kook Rhee ^7^, Pascale Gruber^8^, Marlies Ostermann^9^, Quentin Hill^10^, Peter Depuydt^11^, Christelle Ferra^12^, Anne-Claire Toffart^13^, Peter Schellongowski^14^, Alice Muller^15^, Virginie Lemiale^1^, Fabien Tinquaut^16^, Djamel Mokart^17^, Elie Azoulay^1^

##### ^1^Saint-Louis University Hospital, Paris, FRANCE; ^2^Robert Debre Hospital, Paris, FRANCE; ^3^Saint-Etienne University Hospital, Saint-Etienne, FRANCE; ^4^D’or Institute, Rio de Janeiro, BRAZIL; ^5^Samsung Medical Center, Seoul, REPUBLIC OF SOUTH KOREA; ^6^Ghent University Hospital, Ghent, BELGIUM; ^7^Catholic University of Korea, Seoul, Seoul; ^8^Royal Mardsen Hospital, Londres, UNITED-KINGDOM; ^9^Guy’s & St Thomas’ NHS Foundation Hospital, Londres, UNITED-KINGDOM; ^10^Leeds Teaching Hospitals, Leeds, UNITED-KINGDOM; ^11^Ghent University Hospital, Ghent, UNITED-KINGDOM; ^12^Universitat Autònoma de Barcelona, Barcelone, SPAIN; ^13^Grenoble University Hospital, Grenoble, FRANCE; ^14^Comprehensive Cancer Center, Vienne, AUSTRIA; ^15^Universidade Federal do Rio Grande do Sul, Porto Alegre, BRAZIL; ^16^Hygée Centre and Public Health Department, Saint-Etienne, FRANCE; ^17^Paoli Calmette Institute, Marseille, FRANCE

###### **Correspondence:** Michael Darmon - michael.darmon@aphp.fr

*Annals of Intensive Care* 2019, **9(Suppl 1)**:CO-10

**Introduction**: Overall prognosis of critically ill patients is thought to have improved over the last decades (1). However, this assumption is based upon unadjusted findings and before after studies (1, 2). The aim of this study was to assess the influence ICU admission year on outcome of the critically-ill cancer patient population. Secondary objectives were to assess changes of prognosis in pre-specified subgroup of patients, and independent risk factors of mortality.

**Patients and methods**: This study resulted from a systematic review and meta-analysis on individual data performed according to the PRISMA statements. Public-domain databases (PubMed and Cochrane) were searched by using predefined keywords. The research was restricted to articles published in English and studies focusing on critically ill adult patients from May 2005 to May 2015. The study protocol was registered in the PROSPERO database (CRD42015026347). Selected manuscripts’ authors were then contacted and individual data included in this analysis. Results are reported in n (%) or median (IQR). Adjusted mortality was assessed using a mixed logistic regression model taking year of ICU admission as both fixed and random effect, and study as random effect.

**Results**: Overall, 7,356 patients were included in this study, including 1,666 patients with neutropenia at ICU admission. Median age was 60 years (IQR 49–69). Median SAPSII score at ICU admission was 42 (IQR 28–57). Mechanical ventilation, vasopressors, and renal replacement therapy were required in 50.7% (n = 3.729), 41.1% (n = 3.024) and 16.1% (n = 1.174) of the included patients. Median ICU admission year was 2007 (IQR 2004–2010 + range 1994–2012). Hospital mortality was 47.4% in the overall population, ICU admission year being associated with a progressive decrease in hospital mortality (OR per year 0.94 + 95%CI 0.93–0.95). After adjustment for confounders, year of ICU admission was independently associated with hospital mortality (OR for hospital mortality per year- 0.96 + 95%CI 0.95-.97). This finding was confirmed in all of the subgroups except in patients with allogeneic stem cell transplantation (figure—mortality per year, trend + 95%CI).

**Conclusion**: Our results confirm that hospital mortality of critically ill cancer patients steadily decreased over time after adjustment for patients’ characteristics, patients’ severity and clustering effect. This finding was confirmed in pre-defined subgroups with the exception of allogeneic stem cell transplant recipients.

**References**

1. Shimabukuro-Vornhagen CA-Cancer 2016
2. Peigne ICM 2009

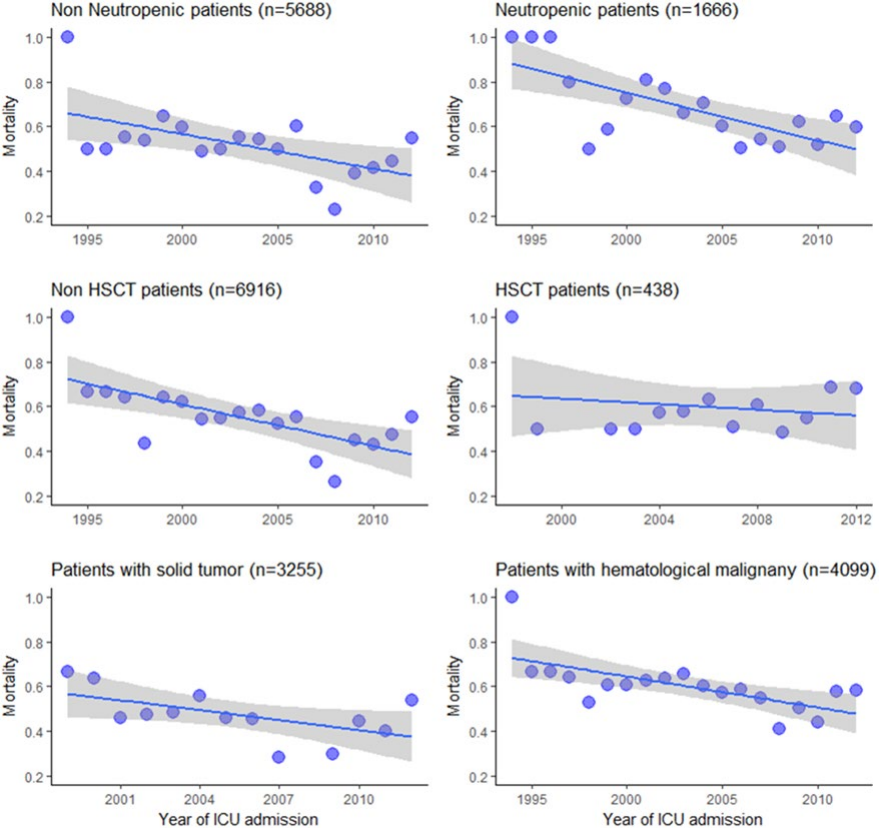


### CO-11 Role of targeted therapy for cancer patients admitted to ICU

#### Virginie Lemiale (*speaker*)^1^, Anne-Pascale Meert^2^, Anne-Claire Toffart^3^, François Vincent^1^, Aude Gibelin^4^, Djamel Mokart^5^, Andry Van de Louw ^6^, Stefan Hatzl^7^, Karin Amrein^8^, Gaelle Rousseau-Bussac^9^, Philip Bauer^10^, Dorothée Carpentier^11^, Fabrice Bruneel^12^, Gabo Moreno^13^, Lucas-Maria Montini^14^, Anne-Sophie Moreau^15^, Pleun Hemelaar^16^, Elie Azoulay^1^

##### ^1^APHP Saint Louis, Paris, FRANCE; ^2^Institut Jules Bordet, Bruxelles, BELGIUM; ^3^CHU, Grenoble, FRANCE; ^4^CHU Tenon, Paris, FRANCE; ^5^Institut Paoli Calmettes, Marseilles, FRANCE; ^6^Penn State College of Medicine, Hershey, UNITED-STATES; ^7^Medical University of Graz, Graz, AUSTRIA; ^8^CHI Creteil, Creteil, FRANCE; ^9^Mayo Clinic, Rochester, UNITED STATES; ^10^CHU, Rouen, FRANCE; ^11^Hôpital Mignot, Versailles, FRANCE; ^12^Hospital Universitari de Bellvitge, Barcelone, SPAIN; ^13^A. Gemelli University Hospital, Roma, ITALY; ^14^CHU Lille, Lille, FRANCE; ^15^VU medical center, Amsterdam, THE NETHERLANDS

###### **Correspondence:** Virginie Lemiale - vlemiale@yahoo.fr

*Annals of Intensive Care* 2019, **9(Suppl 1)**:CO-11

**Introduction**: Mortality of cancer patients remains high. In the last years, new treatments such as targeted therapies, improved outcome of patients with solid tumors. However, those new therapies have been associated with complications leading to ICU admission. The incidence of those severe adverse events is currently unknown. The aim of this observational study was to describe severe adverse effects of targeted therapy.

**Patients and methods**: We conducted an observational multicentric study in 14 international centers belonging to our research group. Critically ill adult patients admitted for any reason between January 1st 2015 and December 31 2016 and treated for solid tumor with targeted therapy prior to ICU admission were included.

**Results**: 91 patients, aged of 61 (51–69) years were included, 56 (62%) were non- smokers patients. Underlying cancer were gasto-intestinal tract (n = 24.22%), breast (n = 18, 20%), kidney (n = 17, 19%), lung (n = 17, 19%) and melanoma (n = 4.4%). 81 (83% patients had metastasis, 44 (48%) patients were PS < 2.21 patients (23%) were treated with anti-EGFR, 25 (28%) patients with anti-VEGF and 5 (5.5%) anti-BRAF. Targeted therapy was started 72 days [17–151] before ICU admission. At ICU admission, the median SOFA score was 3 (1–6). Symptoms at ICU admission were respiratory failure (n = 31 + 35%), infectious diseases (n = 15 + 17%), cardiac failure (n = 14 + 19%) and metabolic disorder (n = 10 + 11%). For 16 (20%) patients, ICU admission was directly related to targeted therapy. Side-effects related to targeted therapy were anaphylaxia (n = 1), cardiac failure (n = 3), bleeding (n = 2), peritonitis (n = 1), metabolic disorder (n = 3), lung injury (n = 4), encephalopathy (n = 1) and SIRS without other etiology (n = 1). 12% (2 16) of them died during ICU stay (2 patients with digestive cancer treated with anti-VEGF who had peritonitis (1 patient) and cardiogenic failure (1 patient)). Other diagnosis were mostly acute respiratory failure related to tumor progression or pneumonia (n = 28), infectious diseases (n = 15) or cardiac failure (n = 13). 65 (71%) patients were discharged alive from ICU. Median ICU stay duration was 2 (1–6) days and1 month survival rate was 55% (n = 50).

**Conclusion**: In this preliminary study, 20% (IC 11.8–28.2%) of ICU admission were related to severe adverse event for targeted therapy. Intensivists should be aware of those complications particularly anti –VEGF when more patients would be treated with those therapies in the further years.

### CO-12 Cytomegalovirus reactivation in intensive care unit patients with hematological malignancies- characteristics and clinical outcomes

#### Alistair Baber (*speaker*)^1^, Camille Vissac^1^, Maud Salmona^2^, Jérôme Le Goff^3^, Audrey De Jong^1^, Eric Mariotte^1^, Lara Zafrani^1^, Elie Azoulay^1^, Michael Darmon^1^, Laure Calvet^1^

##### ^1^AP-HP, ICU, Saint Louis Teaching Hospital, Paris, FRANCE; ^2^AP-HP, Virology Department, Saint Louis Teaching Hospital, Paris, FRANCE

###### **Correspondence:** Alistair Baber - alistair.baber@gmail.com

*Annals of Intensive Care* 2019, **9(Suppl 1)**:CO-12

**Introduction**: Cytomegalovirus (CMV) reactivation occurs frequently in the critically ill and is associated with poor outcomes (1, 2). However, data on immunocompromised patients are scarce. The primary objective of this study was to describe characteristics and outcomes in critically-ill patients with hematological malignancies and CMV reactivation. Secondary objectives included description of CMV disease incidence and characteristics, associations between whole blood viral load and CMV disease and outcomes.

**Patients and methods**: Retrospective single center study (Jan 2010-Dec 2017). Adult patients, admitted to the ICU, having underlying hematological malignancy and CMV reactivation were included. CMV disease was defined according to recent guidelines (2). Results are reported as median (interquartile—IQR) or n (%). Factors associated with hospital mortality or CMV disease were analyzed using logistic regression.

**Results**: 178 patients were included (median age 55y [42–64], 123 patients [69.1%] male). Underlying malignancies were mainly non-Hodgkin’s lymphoma (n = 89 + 50%), acute leukemia (n = 34 + 19.1%) and myeloma (n = 16 + 9.0%) + 37 patients (20.8%) were allogeneic stem cell transplant (SCT) recipients. At admission, median SOFA score was 6 [4–9], and 98 patients required mechanical ventilation (55%), 85 vasopressors (47.8%) and 58 renal replacement therapy (32.6%). Overall, hospital mortality was 53% (n = 95). Median CMV load (whole blood) was 2.7 Log [2.3–3.5]. 44 (24.7%) patients developed CMV disease, including 7 probable or proven CMV diseases (3 pneumonia, 2 encephalitis, 1 retinitis, 1 colitis) and 37 possible CMV diseases (including 19 cytopenia, 11 hepatitis and 8 pneumonia). Mortality was associated with CMV viral load quartiles (Figure, P = 0.02). After adjustment for confounders, need for vasopressors (OR 2.53 + 95%CI 1.11–5.97) and CMV load (OR 1.88 per Log + 95%CI 1.29–2.85) were associated with hospital mortality. When forced one by one in the model, neither CMV disease nor CMV treatment were associated with outcomes. SCT (OR 2.55 + 95%CI 1.05–6.16), mechanical ventilation (OR 4.11 + OR 1.77–10.54) and viral load (OR 1.77 per Log + 95%CI 1.23–2.61) were independently associated with CMV disease.

**Conclusion**: In critically ill hematology patients, CMV viral load is independently associated with hospital mortality. Conversely, neither CMV disease nor treatment was associated with outcome suggesting CMV reactivations reflect an immunodeficiency rather than a cause of poor outcome.


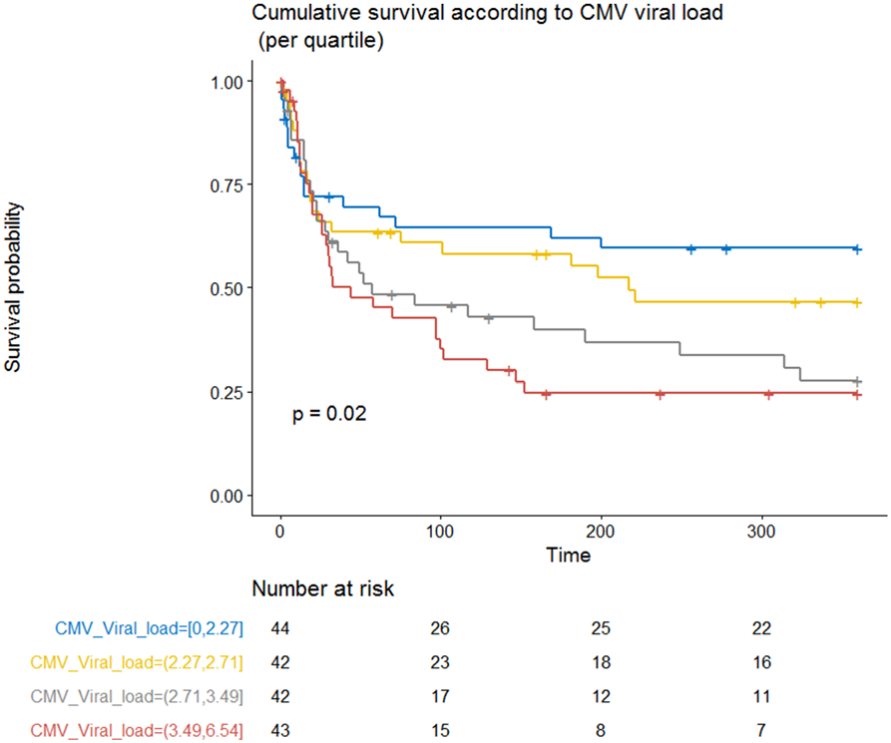


### CO-13 Diaphragm thickening fraction correlates with diaphragm electrical activity in patients under mechanical ventilation

#### Suela Demiri (*speaker*)^1^, Bruno-Pierre Dube^2^, Julien Mayaux^1^, Elise Morawiec^1^, Thomas Similowski^1^, Martin Dres^1^, Alexandre Demoule^1^

##### ^1^Hôpital Universitaire La Pitié-Salpêtrière, Paris, FRANCE; ^2^Centre Hospitalier de l’Université de Montréal, Montreal, CANADA

###### **Correspondence:** Suela Demiri - demiri.suela@gmail.com

*Annals of Intensive Care* 2019, **9(Suppl 1)**:CO-13

**Introduction**: The evaluation of patient’s inspiratory effort is helpful to optimally tailor ventilator settings. While diaphragm electrical activity has been proposed as a valuable tool to assess inspiratory effort, it requires a dedicated tool, namely the NAVA (Neurally Adjusted Ventilatory Assist) catheter that is not widely available. By contrast, the use of ultrasound to evaluated diaphragm contractile activity has been growing in the ICU. However, the relationship between diaphragm contractility activity, i.e. diaphragm thickening fraction (TFdi) and diaphragm electrical activity (EAdi) has not been explored so far. Our hypothesis was that TFdi correlates with EAdi. In the present study, we sought to investigate the correlation between TFdi and EAdi through several conditions of ventilatory assistance.

**Patients and methods**: Patients intubated and ventilated for at least 24 h were eligible for inclusion in the study if they had been previously mechanically ventilated with NAVA mode and the NAVA catheter had been left in place. Ultrasound measurements of diaphragm contractile activity were made synchronously with the measurement of maximal EAdi (EAdimax) during 6 consecutive, randomly assigned ventilatory conditions (NAVA 1 μV/ml, pressure support levels of 5, 10, 15 and 20 cmH2O (with Positive end exipartory pressure PEEP of 5 cm H2O), and zero PEEP with 7 cm H2O pressure support. Overall correlation between EAdimax and TFdi was assessed using Spearman’s correlation coefficient. We also sought to determine the correlation between EAdimax and TFdi for each condition.

**Results**: Twenty patients 60 (53–67) years old were enrolled in the study after a median (25–75 IQR) period of mechanical ventilation of 6 days (3–67). Overall, the correlation between TFdi and EAdimax was r = 0.34, p < 0.001, as shown in Figure 1. The Spearman’s correlation coefficient decreased when the level of ventilatory assistance increased. It was 0.54 (p = 0.03) for zero PEEP with 7 cm H2O pressure support and 0.53 (p = 0.03), 0.53 (p = 0.3), 0.34 (p = 0.2) and 0.15 (p = 0.57) for a level of pressure support of 5, 10, 15 and 20 cmH2O with PEEP of 5 cmH2O and 0.38 (p = 0.14) in NAVA.

**Conclusion**: There was a moderate correlation between diaphragm thickening fraction and diaphragm electrical activity. Our findings suggest that diaphragm thickening fraction behaves as a relevant surrogate of patient’s inspiratory effort but the determinants of this correlation deserve further studies.


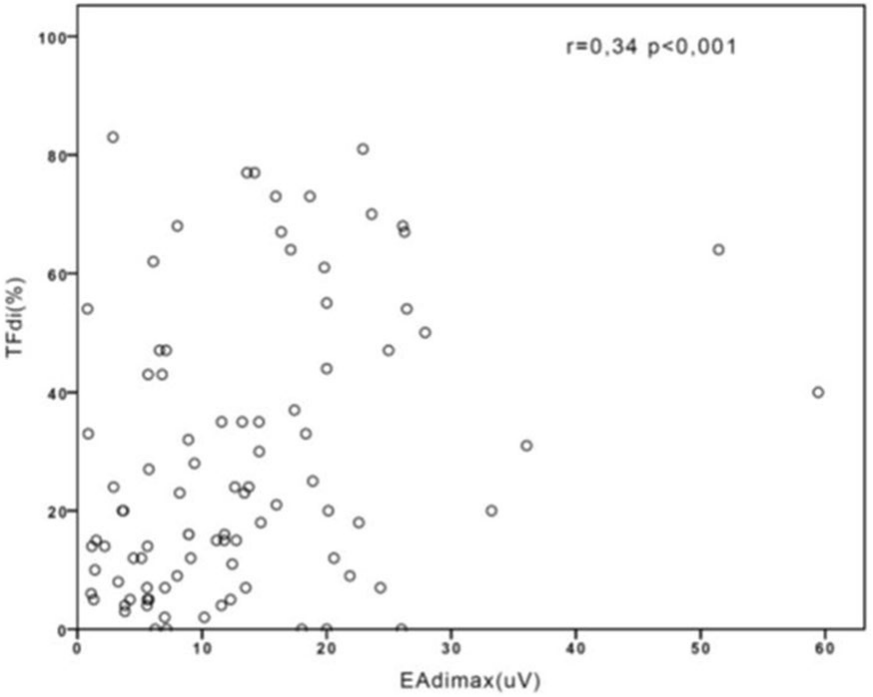


### CO-14 Transpulmonary pressures and computed tomography in experimental ARDS

#### Jean-Christophe Richard (*speaker*)^1^, Maciej Orkisz^2^, Marcela Hernandez Hoyos^3^, Alfredo Morales Pinzon^3^, ClaudeGuérin^4^

##### ^1^HCL, Tassin | Rhone, FRANCE; ^2^CREATIS UMR 5220, Villeurbanne, FRANCE; ^3^Systems and Computing Engineering Department, School of Engineering, Universidad de los Andes, Bogota, COLOMBIE; ^4^Hospices Civils de Lyon- Hôpital de la Croix-Rousse, Lyon, FRANCE

###### **Correspondence:** Jean-Christophe Richard - j-christophe.richard@chu-lyon.fr

*Annals of Intensive Care* 2019, **9(Suppl 1)**:CO-14

**Introduction**: Esophageal pressure to compute transpulmonary pressure (TPP) is an appealing technique to individualize PEEP in ARDS. TPP can be computed 1. as the difference between airway and esophageal pressures (absolute measurement PLABS) + 2. as the product of airway pressure by the ratio of lung to respiratory system elastance (elastance-derived measurement PLEL). The aim of the study was to evaluate the relationships between both TPP measurements and computed tomography (CT) in an experimental model of ARDS.

**Patients and methods**: ARDS was performed by saline lavage on 16 piglets. A recruitment maneuver was performed followed by mechanical ventilation with VT 6 ml kg body weight. PEEP was set to 20 cmH2O then decreased by 2 cmH2O-steps down to 2 cmH2O. Piglets were then randomized into 3 PEEP groups based on best compliance during PEEP trial, best end-expiratory lung volume during PEEP trial, or a PEEP-FiO2 table. Finally, 7 levels of VT ranging from 4 to 20 ml kg were applied at optimal PEEP. TPP measurements and CT were performed at end-expiration and end-inspiration after ARDS onset, during the PEEP trial, 1 h after setting optimal PEEP, and during the variable VT trial.

**Results**: PLABS ranged from -6 to 58 cmH2O, and PLEL ranged from 1 to 65 cmH2O. PLABS and PLEL were significantly correlated (R2 = 0.87, p < 0.001). Bias between PLEL and PLABS amounted to 4 cmH2O (limits of agreement ranging from -2 to 11 cmH2O). Non-inflated compartment significantly increased in deciles of end-expiratory PLABS and PLEL below 4.6 cmH2O and 11.8 cmH2O, respectively (cf. figure). The table provides the diagnostic performance of end-expiratory PLABS and PLEL to detect a non-inflated compartment below 5% and 10% of total lung volume. Overinflated compartment significantly increased in deciles of end-inspiratory PLABS and PLEL above 25.8 cmH2O and 30.7 cmH2O, respectively. ROC curve identified an end-inspiratory PLABS above 19.6 cmH2O and an end-inspiratory PLEL above 24.6 as best thresholds to identify an overinflated compartment greater than 2% of total lung volume.

**Conclusion**: Both TPP measurement techniques have similar diagnostic performance to identify near complete alveolar recruitment at end-expiration and hyperinflation at end-inspiration. Near complete alveolar recruitment may be achieved with PLABS or PLEL greater than 5 and 10 cmH2O, respectively.


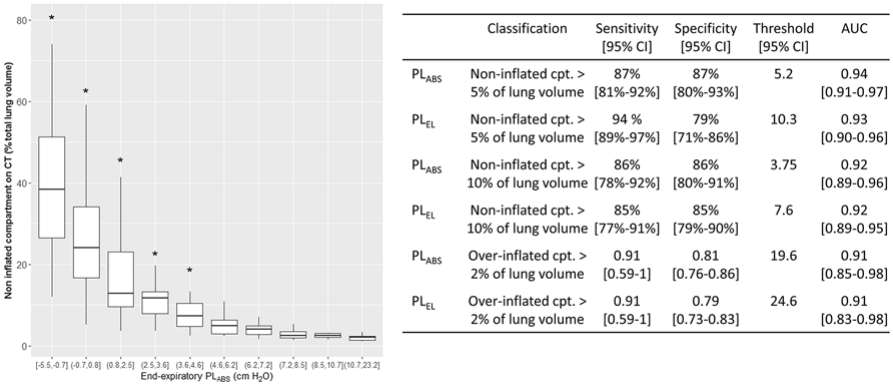


### CO-15 Prone positioning monitored with electrical impedance tomography on patients with severe ECMO-supported ARDS

#### Guillaume Franchineau (*speaker*)^1^, Nicolas Brechot^1^, Guillaume Hekimian^1^, Guillaume Lebreton^2^, SimonBourcier^1^, Come Bureau^1^, Loïc Le Guennec ^1^, Nieszkowska Ania^1^, Pascal Leprince^2^, Luyt Charles-Edouard^1^, Alain Combes^1^, Matthieu Schmidt^1^

##### ^1^APHP, Pitié-Salpêtrière Hospital, Medical Intensive Care Unit, Paris, FRANCE; ^2^APHP, Pitié-Salpêtrière Hospital, Cardiac Surgery Department, Paris, FRANCE

###### **Correspondence:** Guillaume Franchineau - guillaume.franchineau@aphp.fr

*Annals of Intensive Care* 2019, **9(Suppl 1)**:CO-15

**Introduction**: Prone positioning (PP) during veno-venous extracorporeal membrane oxygenation (ECMO) is feasible but its relative effects have never been thoroughly described. Objectives are to describe through electrical impedance tomography (EIT) the impact of PP on regional ventilation and optimal PEEP level, and to derive from EIT baseline predictive factors to identify “PP responders” on ECMO.

**Patients and methods**: ECMO-supported severe ARDS patients, ventilated with a pressure-controlled mode, a 14cmH2O driving pressure, and an EIT-based “optimal PEEP” were included. Before, during, and after a 16 h-PP session, EIT based distribution and variation of tidal impedance, centre of gravity index, end-expiratory lung impedance (EELI), and static compliance were collected. “PP responders” were identified as patients who increased their static compliance by more than 3 ml cmH2O after 16 h of proning.

**Results**: Thirteen (62%) out of 21 studied-patients were considered as “PP responders” on ECMO. “PP responders” had a greater body mass index, more frequently a viral pneumonia and a shorter ECMO duration compared to “PP non-responders” (p < 0.01). For both groups, tidal volume and EELI were redistributed from ventral to dorsal regions during PP. However, “optimal PEEP” was significantly lower in PP than in supine position with 14 (12 -16) and 11 (8–14.5) vs 12 (10–14) and 8 (7.5 -10.5) cmH2O in “PP responders” and “PP non-responders”, respectively (figure 1). Lastly, baseline center of gravity index was lower in “PP responders” (p = 0.03).

**Conclusion**: EIT allows monitoring PP impact on ventilation and seems to be a relevant tool to identify patients who will more likely respond to PP on ECMO.


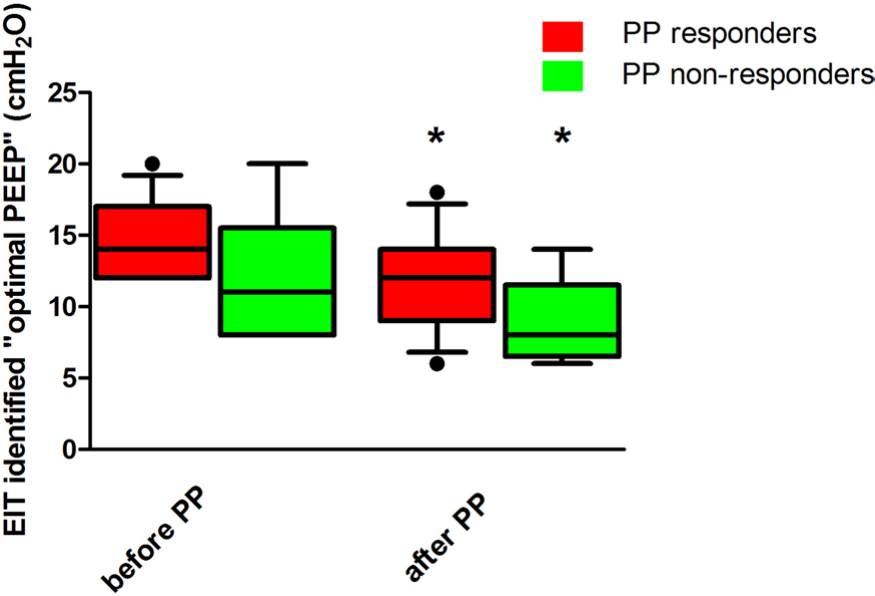


### CO-16 Respiratory muscles contraction after successful ventilator liberation trial predicts extubation failure

#### Martin Dres (*speaker*)^1^, Domenico Luca Grieco^2^, Wissalle Ouechani^1^, Irene Telias^2^, Detajin Junhasavasdikul^2^, Luana Melo^2^, Thomas Piraino ^2^, Felipe Damiani^2^, Lilya Sergenyuk^1^, Lauriane Degravi^1^, Tai Pham^2^, Alexandre Demoule^1^, Thomas Similowski^1^, Laurent Brochard^2^

##### ^1^APHP, Paris, FRANCE; ^2^St Michael Hospital, Toronto, CANADA

###### **Correspondence:** Martin Dres - martin.dres@aphp.fr

*Annals of Intensive Care* 2019, **9(Suppl 1)**:CO-16

**Introduction**: Even for patients who successfully pass a ventilator liberation trial, the period after extubation is at risk of complications. In the present study we investigated whether assessment of respiratory muscles contraction within 2 h after extubation could predict extubation failure (acute respiratory failure, re-intubation or death during a 7-days period following extubation).

**Patients and methods**: Patients from two intensive care units who were intubated since at least 48 h and who successfully passed a ventilator liberation trial were enrolled in the study. Within the two hours following extubation, right hemi-diaphragm (TFdi) and right upper intercostal muscle thickening fraction (TFci) were obtained with ultrasound. TFdi and TFic were compared between patients with and without extubation failure (unpaired test). Receiver operating characteristic (ROC) curves were constructed to predict the risk of extubation failure for each indice.

**Results**: Of the 122 patients who participated in the study, 22 (18%) had extubation failure. Respectively in patients with and without extubation failure, TFdi was 11.9% (9.8–19.4) and 21.5% (15.8–26.3) (p < 0.01) and TFic was 12.8% (8.2–22.2) and 6.5% (4.2–9.1) (p < 0.01). AUC of the ROC curves were 0.73 (0.59–0.86) and 0.76 (0.60–0.91) for TFdi and TFic respectively (p = 0.54). A value of TFdi lower than 16% predicted extubation failure with a sensitivity of 70% (46–88) and a specificity of 73% (63–82). A value of TFic greater than 9% predicted extubation failure with a sensitivity of 75% (51–91) and a specificity of 74% (63–82). Eventually, TFdi TFic ratio was 3.4 (2.0–5.4) and 0.8 (0.3–2.6) in patients with and without extubation failure respectively (p = 0.003). The AUC of the ROC curve for TFdi TFic was 0.82 (0.70–0.94) providing a sensitivity of 71% (60–81) and a specificity of 74% (49–91) to predict extubation failure with a cut-off of 2.2.

**Conclusion**: Assessment of respiratory muscles contraction within two hours after extubation could be used to identify patients at risk of extubation failure. The potential interest of our cut-offs could be used at the time of considering preventive post extubation ventilation strategy.

### CO-17 Effect of empirical aminoglycoside combination therapy on mortality in patients with septic shock- a propensity-based analysis

#### Jean-François Llitjos (*speaker*)^1^, Simon Meslin^1^, Julienn Charpentier^1^, Alain Cariou^1^, Jean-Daniel Chiche^1^, Jean-Paul Mira^1^, Matthieu Jamme ^2^, Frédéric Pène^1^

##### ^1^Médecine-Intensive Réanimation, Paris, FRANCE; ^2^Urgences néphrologues et transplantation rénale, Paris, Paris

###### **Correspondence:** Jean-François Llitjos - jllitjos@gmail.com

*Annals of Intensive Care* 2019, **9(Suppl 1)**:CO-17

**Introduction**: Current guidelines recommend combination antibiotic therapy for patients with septic shock. However the supporting evidence is scarce. The aim of this study is to address the impact of aminoglycoside combination therapy on mortality in septic shock.

**Patients and methods**: This was a 9-year (2008–2016) monocenter retrospective study. All adult patients diagnosed with septic shock within the first 48 h were included. Septic shock was defined as a microbiologically proven or clinically suspected infection, associated with acute circulatory failure requiring vasopressors. Empirical combination therapy was defined by the addition of aminoglycosides within 36 h from the diagnosis of septic shock. A propensity score for aminoglycoside administration was built using day 1 demographic and clinical characteristics. The determinants of mortality were assessed using a propensity-adjusted Cox proportional hazards competing risk analysis.

**Results**: During the study period, 1040 patients were admitted for septic shock. Among them, 64% were males, the median age was 69 [57 + 78] years old. The crude in-ICU mortality rate was 35%. The antibiotic regimen was based on beta lactam therapy in almost all patients (99%), combined with aminoglycosides in 616 (59%) patients. First-line aminoglycosides were distributed into amikacin (61%), gentamicin (37%) and tobramycin (2%). Patients receiving aminoglycoside combination therapy were more frequently immunocompromised (40.4% vs. 31.84%, p = 0.005), had more positive blood cultures (39.1% vs. 25%, p < 0.0001) and had a higher creatininemia at admission (144 [95 + 228] vs. 131 [80 + 201] μmol L, p = 0.007). After propensity score-based matching, 348 patients receiving aminoglycoside combination therapy were compared to 348 patients treated without aminoglycosides. Demographics and clinical characteristics on admission were similar in the two matched groups. The mortality rates in patients treated or not with aminoglycoside combination therapy were 36.2% and 30% (p = 0.07 in univariate analysis), respectively. In propensity-adjusted Cox proportional hazards multivariate competing risk analysis, aminoglycoside combination therapy was not associated with mortality (CSH = 1.19, 95%CI = 0.90–1.55, p = 0.25). After matching, day 3 creatininemia was similar in both groups (91 [54 + 158] vs. 89.5 [54.75 + 158] μmol L, p = 0.86) whereas creatininemia at discharge was higher in patients treated with aminoglycoside combination therapy (92.5 [58–177] vs. 79.5 [54–137.5] μmol L, p = 0.02).

**Conclusion**: In septic shock patients, antibiotic combination with aminoglycosides was associated with neither benefit nor harm.

### CO-18 Potential impact of real-time processing and rapid susceptibility testing of blood samples in severe gram-negative bloodstream infections

#### Sophie Alviset (*speaker*), Julien Charpentier, Hélène Poupet, Claire Poyart, Jean-Paul Mira, Solen Kerneis

##### APHP Hôpital Cochin, Paris, FRANCE

###### **Correspondence:** Sophie Alviset - sophie.alviset@gmail.com

*Annals of Intensive Care* 2019, **9(Suppl 1)**:CO-18

**Introduction**: Timely appropriate therapy is critical in patients with gram-negative bloodstream infections. In standard care, blood cultures are processed during operating hours of the laboratory (8-30-18-30 in our setting). We evaluated the potential impact of real-time processing (24 h a day) and rapid antimicrobial susceptibility testing (AST) on antibiotic regimen in intensive care unit (ICU) patients.

**Patients and methods**: We retrospectively reviewed all episodes of gram-negative bloodstream infections occurring in the ICU of our 1500-bed hospital between 1 st January and 31s December 2017. We collected data on demographics, outcomes, timeframes of sample processing testing and antibiotic changes. Secondly, three scenarios were simulated on the dataset (1) Real-time processing + conventional techniques (gram stain and standard AST) + (2) Standard processing (8-30-18-30) + rapid AST + (3) Real-time processing + rapid AST**In scenarios 1 and 3, transportation time of blood samples to the laboratory was set at 2 h. In scenarios 2 and 3, time to AST result was set at 7 h.

**Results**: We included 86 episodes in 76 patients- 52 men (68%), median age 67 [interquartile range- 53–78]). Median Charlson’s score was 5 [0–15], and 28 patients (37%) died in the ICU. Infection episodes were mainly digestive (26 + 30%) or catheter related (16 + 19%). Most frequent pathogens were- E. coli (36, 33%), Enterobacter sp (20, 18%), P. aeruginosa (11, 10%). Reception of the AST led to a change of the antibiotic regimen in 53 86 (62%) episodes (de-escalation in 46). In standard care, median timeframe from blood collection to gram stain was 26 h [19–34] and from gram stain to AST 29 h [27–50]. Median timeframe for complete processing of blood samples (from collection to result of definitive AST) was 61 h [53–77] in standard care, 48 h [42–66] in scenario 1, 33 h [26–41] in scenario 2 and 22 h [20–27] in scenario 3. Over the whole sample, scenario 1, 2 and 3 had the potential to prompt antibiotic change to pathogen-directed therapy by an average of 5.6, 18.6 and 24.1 h respectively.

**Conclusion**: Real-time processing and rapid AST of blood cultures have a high potential to decrease time to antibiotic change in severe gram-negative bloodstream infections. This effect must be balanced with cost of diagnostic kits and around-the-clock staffing expenses.

### CO-19 Prevalence of insufficient plasma concentration of beta-lactam antibiotics ICU patients- a one-year retrospective single centre study

#### Sacha Sarfati (*speaker*), Fabien Lamoureux, Elsa Desmarest-Durand, Soumaya Skallil, Thomas Clavier, Dorothée Carpentier, Steven Grange, Gaetan Beduneau, Fabienne Tamion, Christophe Girault, Benoît Misset

##### CHU, Rouen, FRANCE

###### **Correspondence:** Sacha Sarfati - sacha.sarfati@gmail.com

*Annals of Intensive Care* 2019, **9(Suppl 1)**:CO-19

**Introduction**: Beta-lactam antibiotics effectiveness is time-dependent and requires that their plasmatic concentration is constantly over the Minimal Inhibiting Concentration (MIC) of the targeted bacterial strain. Dosing recommended by the pharmaceutical companies is based on data derived from patients with non severe diseases and may not be appropriate for ICU patients with sepsis, due to variable alteration of elimination or volume distribution. Our objective was to assess the prevalence of insufficient plasma concentrations of beta-lactam antibiotics in ICU septic patients treated with usual dosing and frequency.

**Patients and methods**: Retrospective analysis of therapeutic drug monitoring (mass spectrometry) between December 2016 and November 2017 for 6 molecules. We selected those assessments performed between 4 and 8 h after IV administration for amoxicillin (AMX) + 3 and 6 h for cloxacillin (CLO), 6 and 12 h for piperacillin (PIP), 6 and 12 h for meropinem (MEM), 8 and 24 h for cefepime (FEP), and 12 and 24 h for ceftriaxone (CRO). EUCAST inferior breakpoint was used for each molecule to approach usual MICs.

**Results**: 178 samples were assessed, of which 127 were residual concentrations (AMX = 24, CLO = 7, PIP = 62, MEM = 14, FEP = , 7, CRO = 13) in 88 patients. Age = 61 + − 4, SAPS 2 = 50 + − 19, weight = 75 + − 22 kg, body mass index = 28 + − 6, shock = 56%, renal replacement therapy = 17%, LOS in ICU = 15.2 + − 12.8 days, mortality = 25%. 30 127 plasma concentrations (23.6%) were below the EUCAST inferior breakpoint- AMX = 4 (17%), CLO = 1 (14%), PIP = 15 (24.1%), MEM 8 (57.1%), FEP = 1 (14.3%), and CRO = 1 (5.8%). Except for MEM and CRO, plasma concentrations were poorly correlated with the time from infusion to sample. Patients with insufficient concentrations were younger (52 + − 15 versus 64 + − 13, p 0.006) and had longer ICU stays before dosage (14 + − 9 vs 7 + − 6 days, p = 0.0001). They had similar ICU length of stay after dosage (11 + − 13 vs 7 + − 8 days, p = 0.16), sex ratio, SAPS 2 scores, weight, BMI, shock, RRT use, and mortality.

**Conclusion**: In our ICU population, usual dosing and frequency of beta-lactam antibiotics led to insufficient plasma level concentrations in 24% of cases, particularly for antibiotics with short half-lives and in younger patients. Insufficient concentrations were more frequently observed during late ICU antibiotic treatments.

### CO-20 Impact of the Unyvero HPN test in ICU patients with ventilator-associated pneumonia or severe hospital-acquired pneumonia

#### Nathan Peiffer-Smadja (*speaker*), Lila Bouadma, Kahina Allouche, Martin Reboul, Philippe Montravers, Jean-François Timsit, Laurence Armand-Lefèvre

##### Hôpital Bichat - Claude Bernard, Paris, FRANCE

###### **Correspondence:** Nathan Peiffer-Smadja - nathan.psmadja@gmail.com

*Annals of Intensive Care* 2019, **9(Suppl 1)**:CO-20

**Introduction**: Early appropriate antibiotic therapy reduces morbidity and mortality of severe hospital-acquired pneumonia (HAP) and ventilator-associated pneumonia (VAP). However, the emergence of bacterial resistance requires the early use of antibiotics with the narrowest possible spectrum.The Unyvero Hospitalized Pneumonia (HPN, Curetis) test is a multiplex PCR detecting 21 bacteria and 19 resistance genes on respiratory samples within 5 h. We assessed the performance and impact of the Unyvero HPN test in ICU patients.

**Patients and methods**: In this prospective single-center study, conducted in 3 ICUs, we performed a Unyvero HPN test on bronchoalveolar lavage (BAL) or plugged telescoping catheters (PTC) samples of patients suspected of VAP or HAP with Gram-negative bacilli or clustered Gram-positive cocci on Gram staining. We evaluated the performance (sensitivity and specificity) of the test compared to conventional culture methods and its potential impact on the choice of antibiotic therapy.

**Results**: We analyzed 71 samples (54 BAL and 10 PTC) from 63 patients (52 males, median age 64 years). A total of 69 84 bacteria were detected by the Unyvero test with a sensitivity of 82% (95% CI, 72–90%) and a specificity of 99% (95% CI 98–100). The performance of the test is shown in the table below. For Pseudomonas aeruginosa, the most frequently isolated pathogen (n = 22), sensitivity and specificity were 100% and 98%, respectively. Two cases of relapses of pneumonia were observed with bacteria detected by the Unyvero test but not initially found with the conventional culture. The 4 extended-spectrum beta-lactamases (ESBL) and 2 carbapenemases (NDM) were all detected by the Unyvero test. The clinical evaluation of 33 episodes of pneumonia showed that the Unyvero test would have led to 22 (67%) therapeutic changes. Of these, 3 corresponded to antibiotic therapy initiations and 19 to modifications in antibiotic treatment including 11 (33%) de-escalations, 5 (15%) adequations or improvement of antibiotic therapy and 3 (10%) less adapted therapies.

**Conclusion**: The Unyvero HPN test has good performance. Its use could lead to changes in antibiotic therapy that are mainly favorable for patients and limit the selection pressure by reducing the antibiotic spectrum early.

**Table:** 

	Organism	Correct identification (Unyvero + / Culture +)	False positive (Unyvero + / Culture −)	Sensitivity (%)	Specificity (%)	Positive predictive value (%)	Negative predictive value (%)
Gram-positive bacteria	Staphylococcus aureus	4/7	0	57	100	100	96
	Streptococcus pneumoniae	0/2	0	0	100	-	97
Enterobacteriaceae	Escherichia coli	13/13	1	100	98	93	100
	Enterobacter cloacae complex	4/7	2	57	97	67	95
	Proteus spp.	5/5	0	100	100	100	100
	Klebsiella pneumonia	8/12	0	67	100	100	94
	Klebsiella oxytoca	2/2	2	100	97	50	100
	Serratia marcescens	3/3	0	100	100	100	100
	Morganella morganii	1/3	0	33	100	100	97
Non-fermenting bacteria	Pseudomonas aeruginosa	22/22	1	100	98	96	100
	Actinetobacter baumanii complex	2/2	0	100	100	100	100
	Strenotrophomonas maltophilia	0/0	2	-	97	0	100
Others	Legionella pneumophila	1/1	0	100	100	100	100
	Haemophilus influenzae	1/2	0	50	100	100	99
Total*		69/84	9	82	99	88	99

*Total including all organisms in the panel

### CO-21 Medical rhabdomyolysis in critically ill patients- initial presentation and short- and long-term outcomes

#### Ines Gragueb-Chatti (*speaker*), Romain Hernu, Marie Simon, Thomas Baudry, Thomas Madelaine, Adeline Grateau, Martin Cour, Laurent Argaud

##### Hospices Civils de Lyon, Lyon, FRANCE

###### **Correspondence:** Ines Gragueb-Chatti - ines.gragueb-chatti@chu-lyon.fr

*Annals of Intensive Care* 2019, **9(Suppl 1)**:CO-21

**Introduction**: Rhabdomyolysis is a common pathology in intensive care unit (ICU), often associated with acute kidney injury (AKI) and high mortality rate. To date, clinical course and prognostic factors of medical rhabdomyolysis in ICU are poorly documented. The aim of the study was to describe characteristics and evolution of critically-ill patients with medical rhabdomyolysis and to identify prognostic factors associated with short and long-term outcomes.

**Patients and methods**: A retrospective study was conducted between January 2006 and December 2016, in the three medical ICUs of Lyon (France). All adult patients hospitalized with severe medical rhabdomyolysis (creatine kinase [CK] > 5,000 IU L) were included. Demographic features, comorbidities (Charlson score), clinical and laboratory data including AKI (according to KDIGO classification), organ failure support, day-28 outcome and one-year mortality were collected.

**Results**: A total of 427 patients were included. The mean age was 55 ± 17 years, and sex ratio was 1.8. The most common causes of rhabdomyolysis were immobilization (n = 159, 37%) and sepsis (n = 116, 27%). At admission, SOFA score was 8.8 ± 5.1, and mean CK level reached 16.814 ± 28.616 IU L. During ICU stay, 324 patients (76%) developed AKI and 132 (31%) required renal replacement therapy. ICU, day-28, and one-year mortality were 27, 26 and 32%, respectively. Initial CK level did not significantly differ between patients alive (18.186 ± 30.238 IU L) and dead (12,644 ± 22,617 IU L) at day 28 (p = 0.05). After multivariate analysis, Charlson score and organ failures (cardiovascular, respiratory or neurological) were found to be independently associated with day-28 mortality (p < 0.05). Age, Charlson score and highest SOFA score during ICU stay were independent factors associated with one-year mortality (p < 0.05).

**Conclusion**: These results emphasize the poor outcome of critically-ill patients with severe medical rhabdomyolysis. In this population, comorbidities and organ failures, but not initial CK level, appear to be major determinants of short and long-term prognosis.

### CO-22 Are ambulance resources associated with outcome in out-of-hospital cardiac arrests? Insights from the Paris- Sudden Death Expertise Centre registry

#### Florence Dumas (*speaker*)^1^, Richard Chocron^1^, Thomas Loeb^1^, Lionel Lamhaut^1^, Daniel Jost^1^, Frédéric Adnet^1^, Eric Lecarpentier^1^, Wulfran Bougouin^2^, Franckie Beganton^3^, Philippe Juvin^4^, Eloi Marijon^4^, Xavier Jouven^4^, Alain Cariou^5^

##### ^1^Emergency medicine, Paris, FRANCE; ^2^ICU, Paris, FRANCE; ^3^CEMS, Paris, FRANCE; ^4^HEGP, Paris, FRANCE; ^5^Cochin, Paris, FRANCE

###### **Correspondence:** Florence Dumas - dumasflorence@gmail.com

*Annals of Intensive Care* 2019, **9(Suppl 1)**:CO-22

**Introduction**: In out-of-hospital cardiac arrest (OHCA), geographic disparities in outcomes may reflect baseline variations in patients’ characteristics but may also result from differences in the number of ambulances providing basic life support (BLS) and advanced life support (ALS). We aimed at assessing the influence of allocated ambulance resources on outcome in OHCA patients in a large urban community.

**Patients and methods**: From May 2011 to January 2016, we analyzed a prospectively collected Utstein database for all OHCA adults. Cases were geocoded according to 19 neighborhoods and the number of BLS (firefighters performing cardiopulmonary resuscitation and applying automated external defibrillator) and ALS ambulances (medicalized team providing advanced care such as drugs and endotracheal intubation) was collected. We assessed the respective influence of Utstein parameters, socio-economic characteristics and ambulance resources of these neighborhoods using a mixed-effect model with successful return of spontaneous circulation (ROSC) as the primary endpoint and survival at hospital discharge as a secondary endpoint.

**Results**: During the study period, 8754 non-traumatic OHCA occurred in the Greater Paris area. Overall ROSC rate was 3675 8754 (41.9%) and survival rate at hospital discharge was 788 8754 (9%), ranging from 33% to 51.1% and from 4.4% to 14.5% respectively, according to neighborhoods (p < 0.001). Patients’ and socio-demographics’ characteristics significantly differed between neighborhoods (p for trend < 0.001). After adjustment, a higher density of ambulances was associated with successful ROSC (respectively aOR = 1.31 (1.14–1.51) + p < 0.001 for ALS ambulances > 1.5 per neighborhood and aOR = 1.21 (1.04–1.41) + p = 0.01 for BLS ambulances > 4 per neighborhood). Regarding survival at discharge, only the number of ALS ambulances > 1.5 per neighborhood was significant (aOR = 1.30 (1.06–1.59) p = 0.01).

**Conclusion**: In this large urban population-based study of out-of-hospital cardiac arrests patients, we observed that allocated resources of Emergency Medical Service (EMS) are associated with outcome, suggesting that improving healthcare organization may attenuate disparities in prognosis.

### CO-23 Assessment of severe eosinopenia for the diagnosis of infection in the Emergency Department and comparison with other biomarkers

#### Jérémy Rosman (*speaker*)^1^, Simon Hainguerlot^2^, Aurélien Cordonnier^3^, Karelle Staffe^3^, Olivier Gallon^4^, Thomas Beuvelet^5^, Xavier Fontaine ^2^, Philippe Mateu^1^

##### ^1^Service de Médecine Intensive Réanimation, Centre Hospitalier de Charleville-Mézières, Charleville-Mézières, FRANCE; ^2^Service d’Accueil des Urgences, Centre Hospitalier de Charleville-Mézières, Charleville-Mézières, FRANCE; ^3^Département de l’Information Médicale, Centre Hospitalier de Charleville-Mézières, Charleville-Mézières, FRANCE; ^4^Service des Maladies Infectieuses, Centre Hospitalier de Charleville-Mézières, Charleville-Mézières, FRANCE; ^5^Laboratoire de Biologie, Centre Hospitalier de Charleville-Mézières, Charleville-Mézières, FRANCE

###### **Correspondence:** Jérémy Rosman - jrosman@ch-charleville-mezieres.fr

*Annals of Intensive Care* 2019, **9(Suppl 1)**:CO-23

**Introduction**: Infectious diseases represent a frequent cause of consultation in the Emergency Department (ED). Biomarkers may help clinicians to diagnose infection from other causes. Severe eosinopenia may reflect level of systemic inflammation. The aim of our study was to evaluate severe eosinopenia, as defined a value < 0.01 G L (< 10 mm3), as a marker of infection.

**Patients and methods**: Patients admitted in the ED with at least one complete blood count were included. Biomarkers of infection were evaluated by multivariable logistic regression.

**Results**: Among 15.638 patients screened, 11.520 patients were finally included, 14% were infected. Severe eosinopenia was present in 49% of infected patients and 23% in non-infected patients (p < 0.001). Severe eosinopenia had a modest accuracy for diagnosis of infection with 49% sensitivity, 77% specificity, 26% positive predictive value, 90% negative predictive value, 2.1 positive likelihood ratio and 0.47 négative likelihood ratio. Accuracy was lower than other biomarkers as C-reactive protein, fibrinogen, procalcitonin and (neutrophilic) leukocytosis. In mutivariable analysis, severe eosinopenia was an independant factor of infection [adjusted odds ratio 1.3, (95% confidence interval 1.1–1.5), but lower than other biomarkers).

**Conclusion**: Severe eosinopenia, a reflect of systemic inflammation, was an independent factor associated with infection, but is not better than other usual biomarkers.

### CO-24 QSOFA as predictor of mortality and prolonged ICU admission in Emergency Department patients with suspected infection

#### Emmanuel Canet (*speaker*)^1^, David Mcd Taylor^2^, Richard Khor^2^, Vivek Krishnan^2^, RinaldoBellomo^2^

##### ^1^Intensive Care Unit, Nantes, FRANCE; ^2^Austin Health Hospital, Melbourne, AUSTRALIA

###### **Correspondence:** Emmanuel Canet - emmanuel.canet@chu-nantes.fr

*Annals of Intensive Care* 2019, **9(Suppl 1)**:CO-24

**Introduction**: We assessed the quick Sequential Organ Failure Assessment (qSOFA) score as a predictor of in-hospital mortality or prolonged ICU stay in Emergency Department (ED) patients with suspected infection.

**Patients and methods**: We measured qSOFA in a cohort of 11,205 ED patients with suspected infection. The primary outcome was in-hospital mortality and or ICU stay ≥ 3 days.

**Results**: The qSOFA score was positive in 2,429 (21.7%) patients. In-hospital mortality, and in-hospital mortality or ICU stay ≥ 3 days were 12.8% and 17.2% respectively for qSOFA positive patients vs 2.2% and 4.2% for qSOFA negative patients (p < 0.0001). For the prediction of in-hospital mortality, a positive qSOFA had a positive predictive value (PPV) of 13% (95% CI, 11–14) and a negative predictive value (NPV) of 98% (95% CI, 97–98). For the prediction of in-hospital mortality or ICU stay ≥ 3 days, the PPV and NPV of a positive qSOFA were 17% (95% CI, 16–19) and 96 (95% CI, 95–96), respectively.

**Conclusion**: Among ED patients with suspected infection, a positive qSOFA identified those at much greater risk of mortality and longer ICU stay.

### CO-25 Prevalence, management and prognosis of hypoxemia among obese patients in the ICU- insights from the SPECTRUM study

#### Florence Boissier (*speaker*)^1^, David Grimaldi^2^, Sami Hraiech^3^, Philippe Michel^4^, Jean-BaptisteLascarrou^5^, Tai Pham^6^, Jean-Christophe Richard^7^, Arnaud W Thille^8^, Stephan Ehrmann^9^, Nadia Aissaoui^10^, Jean-Claude Lacherade^11^, Gregoire Muller^12^

##### ^1^Service de réanimation médicale CHU, Poitiers, FRANCE; ^2^Intensive care unit, Erasme Hospital, Bruxelles, BELGIUM; ^3^réanimation des détresses respiratoires et infections sévères, Hopital Nord APHM, Marseille, FRANCE; ^4^Service de réanimation, CH René-Dubos, Pontoise, FRANCE; ^5^Médecine Intensive Réanimation CHU, Nantes, FRANCE; ^6^Critical Care Medicine, St Michael’s Hospital, Toronto, CANADA; ^7^Réanimation médicale, hôpital de la Croix Rousse, Lyon, FRANCE; ^8^Réanimation médicale, CHU, Poitiers, FRANCE; ^9^Médecine Intensive Réanimation, CHRU, Tours, FRANCE; ^10^Réanimation médicale, HEGP, APHP, Paris, FRANCE; ^11^Médecine Intensive - Réanimation, CHD Vendée, site de la Roche sur Yon, La Roche-Sur-Yon, FRANCE; ^12^Service de Médecine Intensive Réanimation, Orléans, FRANCE

###### **Correspondence:** Florence Boissier - floboissier@yahoo.fr

*Annals of Intensive Care* 2019, **9(Suppl 1)**:CO-25

**Introduction**: Using the SPECTRUM study, we aimed to evaluate whether hypoxemia in ICU among obese patients had different causes and management compared to non-obese patients.

**Patients and methods**: Subgroup analysis of a prevalence-point-day conducted in 117 French-speaking ICU aiming to report the patterns and outcomes of hypoxemic patients (defined by P F < 300 mmHg). Obesity was defined as a body mass index (BMI) > 30 kg m2.

**Results**: Among 1571 patients hospitalized in ICUs the day of the study with BMI data, 428 were obese (27%). 247 of them were hypoxemic (57.7%) as compared with 597 1143 (52.2%) non-obese patients (p 0.05). They exhibited more frequent obesity-hypoventilation syndrome (23% versus 1.5% p < 0.001) and sleep apnea (19.4% versus 3.5% p < 0.001), less frequent non-obstructive chronic respiratory disease (2% versus 8.6% p < 0.001) and interstitial pulmonary disease (1.2% versus 4.4% p 0.02) and had more frequently home non-invasive ventilation (NIV) (5.7 versus 2.9% p 0.05). Hypoxemia was mild in 52%, moderate in 39% and severe in 9%, similar to non-obese patients. ARDS criteria were fulfilled in 21.5% (versus 20.6% in non-obese patients). They required high flow oxygen, NIV and invasive ventilation respectively in 5.7, 6.9 and 58.7% of cases, which was not different from non-obese patients. PEEP was higher (7 (IQR 5–10) versus 6 (IQR 5–8) cmH2O, p 0.03). Tidal volume in intubated patients was 7 ml kg (IQR 6–8.4) in obese patients versus 6.8 (6.1–7.7) (p 0.06). Plateau pressure was not different between the 2 groups (24 versus 22 cm H2O), as well as PaO2, PaCO2 and pH. Diagnosis of atelectasis was frequent in the 2 groups (23% versus 25.7%). There was no difference in the use of prone position (5.2 versus 4.3%, p 0.66). ICU mortality of obese patients was 20.6% versus 27.4% (p 0.04). Multivariate Cox model confirmed a negative independent association between obesity and ICU mortality.

**Conclusion**: Obese patients represent more than 25% of the patients hospitalized in ICU the day of the study. Hypoxemia seems more frequent in obese patients. Respiratory support and hypoxemia severity were similar between obese and non-obese patients. PEEP was higher in obese patients. Obesity was associated with a lower mortality.

### CO-26 Do all immunocompromised patients with ARF respond equally to oxygenation strategy?

#### Virginie Lemiale (*speaker*)^1^, Audrey De Jong^2^, Guillaume Dumas^3^, Jordi Rello^4^, Michael Darmon^5^, Philip Bauer^6^, Andry Van de Louw ^7^, Julien Mayaux^8^, Ignacio Martin-Loesche^9^, Djamel Mokart^10^, Peter Schellongowski^11^, Sangeeta Mehta^12^, Achille Kouachet^13^, Frédéric Pene^14^, Peter Pikkers^15^, Gaston Burghi^16^, Massimo Antonelli^17^, Fabrice Bruneel^18^, Andreas Barratt-Due^19^, Miia Valkonen^20^, Victoria Metaxa^21^, Anders Perner^22^, Julien Dessajan^5^

##### ^1^APHP Saint Louis, Paris, FRANCE; ^2^Anesthesiology and Intensive Care + Anesthesia and Critical Care Department B, Montpeller, FRANCE; ^3^Medical ICU APHP Saint Antoine, Paris, FRANCE; ^4^Vall d’Hebron Hospital, Barcelone, SPAIN; ^5^Medical ICU APHP Saint Louis, Paris, FRANCE; ^6^Mayo Clinic, Rochester, UNITED STATES; ^7^Penn State College of Medicine, Hershey, UNITED STATES; ^8^Medical ICU APHP Pitié Salpétriere, Paris, FRANCE; ^9^St James’s University Hospital, Dublin, IRELAND; ^10^Institut Paoli Calmettes, Marseilles, FRANCE; ^11^Medical University of Vienna, Vienne, AUSTRIA; ^12^Mount Sinai Hospital, Toronto, CANADA; ^13^Medical ICU CHU, Angers, FRANCE; ^14^Medical ICU APHP Cochin, Paris, FRANCE; ^15^Radboud University Medical Center, Nijmegen, THE NETHERLANDS; ^16^Montevideo University hospital, Montevideo, URUGUAY; ^17^Catholic University of Rome- A. Gemelli University Hospital, Roma, ITALY; ^18^Medical ICU A Mignot, Versailles, FRANCE; ^19^Oslo University Hospital, Oslo, NORVÈGE; ^20^Helsinki University Hospital, Helsinki, FINLANDE; ^21^King’s College Hospital, Londres, UNITED-KINGDOM; ^22^Rigshospitalet, University of Copenhagen, Copenaghe, DENMARK

###### **Correspondence:** Virginie Lemiale - vlemiale@yahoo.fr

*Annals of Intensive Care* 2019, **9(Suppl 1)**:CO-26

**Introduction**: In immunocompromised patient with acute respiratory failure (ARF), mortality remains high. First oxygenation strategy with non-invasive ventilation or high flow nasal oxygen has not been clearly demonstrated according to the patient status. We assessed assess outcomes in patients with hematological malignancies and acute respiratory failure (ARF) according to the initial ventilation strategy, radiological lesion and ARF etiology.

**Patients and methods**: All patients with ARF included in MINIMAX, TRIALOH and IVNICTUS studies and who were not intubated at admission, were included in this post hoc analysis of three multicenter studies. An external validation was then performed in the EFRAIM cohort.

**Results**: 847 patients admitted with ARF were included. At ICU admission, radiological pattern was subnormal (n = 75, 9%), focal lesion (n = 159 + 20%), diffuse alveolar lesion (n = 444 + 55%) or interstitial lesion (n = 127 + 16%). Diagnosis of ARF was mainly related to bacterial or viral pneumonia (335, 40%). Diagnosis of ARF was not found for 147 (17%) patients. First oxygenation strategy was standard oxygen (n = 310), NIV (n = 400), HFNO (n = 65) and NIV + HFNC (n = 72). Bilateral alveolar pattern (OR = 2.05 (1.00–4.22), p = 0.05) was independently associated with day-28 mortality after adjusting on NIV use within the 2 first days (OR = 1.76 (1.14–2.72), p = 0.01), SOFA score without respiratory item at ICU admission (OR = 1.16 (1.09–1.24), p < 0.001) and PaO_2_ FiO_2_ < 100 at ICU admission (OR = 1.69 (1.16–2.50), p = 0.007). Opportunistic infection (OR = 2.16 (1.14–4.09), p = 0.01) and no identified cause (OR = 1.97 (1.08–3.58)) were independently associated with day-28 mortality after taking into account NIV use within the 2 first days (OR = 1.86 (1.22–2.83), p = 0.0004), SOFA score without respiratory item at ICU admission (OR = 1.17 (1.10–1.25), p < 0.001) and PaO2 FiO2 < 100 at ICU admission (OR = 1.69 (1.16–2.50), p = 0.01). The analyses performed in the validation cohort confirmed the results found in the initial cohort.

**Conclusion**: NIV use, opportunistic or no identified diagnosis and bilateral alveolar radiological pattern were associated with mortality, after taking into account the severity of ARF disease using SOFA score and PaO2 FiO2 ratio.

### CO-27 High-flow oxygen therapy vs non invasive ventilation- a prospective cross-over physiological study of alveolar recruitment in acute respiratory failure

#### Elise Artaud-Macari (*speaker*), Michael Bubenheim, Gurvan Le Bouar, Dorothée Carpentier, Steven Grangé, Déborah Boyer, Gaëtan Béduneau , Benoît Misset, Antoine Cuvelier, Fabienne Tamion, Christophe Girault

##### Pulmonary, Respiratory Intensive Care and Thoracic oncology Department, Rouen University Hospital, UPRES EA-3830 I, Rouen, FRANCE

###### **Correspondence:** Elise Artaud-Macari - eliseartaudmacari@yahoo.fr

*Annals of Intensive Care* 2019, **9(Suppl 1)**:CO-27

**Introduction**: High-flow oxygen therapy (HFNC) has shown a benefit for the prognosis of patients with hypoxemic acute respiratory failure (ARF), while noninvasive ventilation (NIV) remains debated in this indication. We evaluated the effect of HFNC on alveolar recruitment and lung volumes in hypoxemic ARF compared to NIV and facial mask (FM).

**Patients and methods**: A prospective cross-over physiological study was conducted. Eligible patients had to present a hypoxemic ARF due to pneumonia requiring HFNC and or NIV according to ICU physician. Cardiogenic pulmonary oedema and underlying respiratory disease were excluded. Each patient was investigated with the Pulmovista^®^ (Dräger, Lübeck, Germany) device and underwent 15 min periods of HFNC or NIV in a randomized order, interspersed with 15 min periods of FM used as reference. The primary endpoint was the comparison of global and regional end-expiratory electrical lung impedance (EELI) between NIV and HFNC. Secondary endpoints were the comparison, between the 3 techniques, of lung volumes (global and regional tidal variations (TV), respiratory parameters, hemodynamic tolerance, dyspnea and comfort.

**Results**: NIV and HFNC significantly increased the global EELI compared with FM (2056 [1070 + 2825] vs. 4083 [2928 + 5134], p = 0.001 and 1448 [1028 + 3542] vs 2921 [1706 + 4850], p = 0.0001, respectively). No global EELI difference was found between NIV and HFNC (4083 [2928 + 5134] vs 2921 [1706 + 4850], p = 0.4) (fig 1.1). Global and regional TV increased under NIV compared to HFNC (p < 0.05) or FM (p < 0.05), while HFNC did not modify TV over FM. NIV significantly improved the SpO2 FiO2 ratio compared to HFNC (p = 0.001) (fig 1.2). HFNC significantly reduced respiratory rate vs FM (p = 0.04) but not NIV. No difference was found for dyspnea score between the 3 techniques. Patient comfort was similar between HFNC and FM but decreased with NIV.

**Conclusion**: This study demonstrates a similar benefit of HFNC and NIV on alveolar recruitment with the settings used, as compared to FM. By contrast to HFNC and despite a better oxygenation, NIV also increases lung volumes which may contribute to its potentially deleterious effect during hypoxemic ARF leading to the recent concept of Patient Self Inflicted Lung Injury or P-SILI (Brochard L et al. AJRCCM 2017 + 195-438-42).


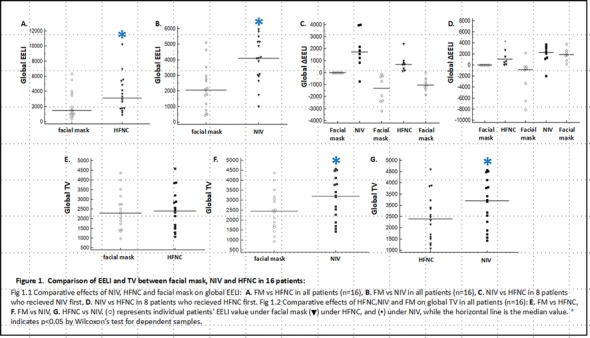


### CO-28 Lung and chest wall mechanics of patients admitted to Intensive Care Unit

#### Elise Yvin (*speaker*), Pierre-Yves Olivier, Lise Piquilloud, Satar Mortaza, Alain Mercat, François Beloncle

##### Département de Médecine Intensive-Réanimation et Médecine Hyperbare, CHU d’Angers, Angers, FRANCE

###### **Correspondence:** Elise Yvin - yvin.elise@gmail.com

*Annals of Intensive Care* 2019, **9(Suppl 1)**:CO-28

**Introduction**: Data concerning respiratory mechanics of intubated patients separately analyzing the lung and the chest wall are scare. This study aims at describing respiratory mechanics (respiratory system compliance (CRS), lung compliance (CL), chest wall compliance (CCW) and end-expiratory lung volume (EELV)) of all intubated patients admitted to Intensive Care Unit (ICU).

**Patients and methods**: This is an interim analysis of a prospective single-center study. All patients admitted to ICU, intubated and paralyzed as part of the routine care, without contraindication to esophageal pressure measurement were included. Respiratory mechanics measurements were performed at their admission to ICU with standardized ventilator settings. CL and CCW were measured using esophageal pressure measurement (Nutrivent catheter, Sidam ^®^, San Giacomo Roncole, Italy). EELV was measured, at a positive end-expiratory pressure of 5 cmH2O, using the nitrogen washout-washin technique (CRF inview, GE-Healthcare ^®^, Madison, WI, USA). Results are presented as median [IQR]. Correlations were analyzed using Spearman test.

**Results**: 30 patients were included in the study. ICU admission diagnoses were sepsis other than pulmonary (9 patients, 30%), pneumonia (5 patients, 17%), cardiac arrest (6 patients, 20%), acute cardiac failure (5 patients, 16%) or neurologic disorder (5 patients, 17%). Ten patients (33%) met Acute Respiratory Distress Syndrome criteria at the day of respiratory mechanics measurement. Distributions of CRS, CL and CCW are presented in figure 1. There was a wide distribution of CCW values. CCW impairment (defined as CCW < 100 mL cmH2O) was observed in 7 patients (23%). No clinical history of abdominal hypertension was found in these patients. CCW was not correlated with the body mass index (p = 0.21 + r = 0.24). CL was well correlated with EELV (p < 0.001 + r = 0.80).

**Conclusion**: This study shows that, in a non-selected population of intubated patients admitted to ICU for various reasons, distributions of CL and CCW are wide. CCW impairment is not rare and cannot be predicted by the patient’s medical history or morphology. The good correlation between CL and EELV is consistent with data establishing that CL represents the aerated lung volume.

### CO-29 Incidence of ventilatory acquired pneumonia in PICU- a one-year prospective multicenter data-base (the INCIPAVE study)

#### Stephane Dauger (*speaker*)^1^, Yves Gallien^2^, Josephus Van Gestel^3^, Michael Levy^1^, Astrid Botte^4^, Fleur Cour-Andlauer^5^, Camille Guillot ^6^, Fabrice Lesage^7^, Jérôme Rambaud^8^, Catherine Vanbaelen^9^, Maryline Chomton^1^, Matthieu Resche-Rigon^10^

##### ^1^Réanimation pédiatrique, hôpital Robert Debré, Paris, FRANCE; ^2^Hôpital Saint-Louis-Sce de Biostatistique et Information Médicale, Paris, FRANCE; ^3^PICU, Utrecht, THE NETHERLANDS; ^4^Réanimation pédiatrique, Hôpital Pellegrin, Bordeaux, FRANCE; ^5^Réanimation pédiatrique, HFME, Lyon, FRANCE; ^6^Réanimation pédiatrique, Hôpital Jeanne de Flandres, Lille, FRANCE; ^7^Réanimation pédiatrique, Hôpital Necker Enfants malades, Paris, FRANCE; ^8^Réanimation pédiatrique, Hôpital Armand Trousseau, Paris, FRANCE; ^9^Réanimation pédiatrique, Hôpital des enfants, Toulouse, FRANCE; ^10^Service de Biostatistique et Information Médicale, Hôpital Saint-Louis, Paris, FRANCE

###### **Correspondence:** Stephane Dauger - stephane.dauger@aphp.fr

*Annals of Intensive Care* 2019, **9(Suppl 1)**:CO-29

**Introduction**: Ventilatory Acquired Pneumonia (VAP) is one of the main nosocomial infection in adult ICU. Only one prospective multicenter study performed during six months in 16 PICUs of the US has prospectively described pediatric VAP. We design the INCIPAVE study to report the occurrence of VAP in european PICUs, focusing on patients, risk factors (RF), micro-organisms involved, diagnostic tools and antibiotics used. The first aim of this study was to calculate the incidence of VAP.

**Patients and methods**: Multicenter prospective cohort study from 03/04/2017 to 03/04/2018 including all patients mechanically ventilated (MV) at least once in eight PICUs, one in the Netherlands and seven in France. VAP was defined using the 2015 CDC criteria, applied during PICU stays, excluding the 48 h preceeding and following PICU. Patients were described on admission and main RF ever tested in the medical litterature were daily included by a pediatric intensivist of each PICU in an electronic database on a securized dedicated website. The Ethics Committee of the French Society of Intensive Care approved the study, which has been declared to the CNIL and recorded on Clinical-Trials.org. All parents were individually informed by a dedicated sheet. Descriptive data are reported as number (%) or medians [first-third quartiles]. Incidence was calculated as the number of VAP for 1000 days of MV.

**Results**: These results are based on declarative information reported in the INCIPAVE database during its first opening on September 2018, before cleaning. During one year, 2047 episodes of MV were included in 1856 patients (26.5 months [6 + 92], 12kgs [6.4 + 23.22], 56% of males). Main reasons for admission to PICU were post-operative care (35.1%) and respiratory (23.7%), neurological (17.1%) or circulatory (12.2%) failures. PIM-2 score was 2.8% [1.3 + 7.5] with an observed mortality of 10.9%. PELOD-2 score on day-1 was 5 [4.7]. Patients were ventilated via uncuffed tubes in 19.6% of cases (7.8% of tracheostomy). A total of 158 VAP was declared during 11685 days of MV. The incidence of VAP was 13.5 1000 days of MV (IC95% - [11.4 + 15.6]).

**Conclusion**: This incidence of VAP in INCIPAVE study is higher than the incidence reported recently by Gupta (7.0 1000 days of MV) (1). New analyses are planned after cleaning of the database, including a special reading of each case of VAP.

### CO-30 Severe sepsis and septic shock in hematological pediatric patients admitted to picu - a bicentric retrospective study from 2011 to 2017

#### Aurélia Alimi (*speaker*)^1^, Jérôme Rambaud^2^, Julie Sommet^1^, Sandrine Jean^2^, Michael Levy^1^, Maryline Chomton^1^, Fleur Le Bourgeois^1^, Stephane Dauger^1^

##### ^1^Hôpital Robert Debré APHP, Paris, FRANCE; ^2^Hôpital Armand Trousseau APHP, Paris, FRANCE

###### **Correspondence:** Aurélia Alimi - aurelia.alimi@aphp.fr

*Annals of Intensive Care* 2019, **9(Suppl 1)**:CO-30

**Introduction**: Pediatric sepsis remains a burdensome public health problem, especially in patients with immuno-deficiency. Adherence to Survival Sepsis Campaign (SSC) would be associated with lower mortality. However, this correlation had never been evaluated in children with hematologic malignancies.

**Patients and methods**: Retrospective bicentric cohort study of children with identified hematologic malignancy or hematopoietic stem cell transplantation and requiring intensive care for severe sepsis between January 2011 and August 2017. Detailed description of sepsis resuscitation just before and within six hours after the diagnosis of sepsis. Evaluation of adherence to SSC recommandations, before and after admission to Pediatric Intensive Care Unit (PICU). Accurate analysis of microbiologic etiologies. Assessment of mortality 6 months after the sepsis.

**Results**: 65 patients were included from the 78 who have been screened. Before admission in ICU - 38% of them did not receive oxygen, 47% of patients showed low blood pressure for age without continuous infusion of inotropes, and lactic acid was measured in few patients (14%). Within 6 h of PICU admission, 28% did not receive oxygen and only 24% of patients with continuous infusion of inotropes were ventilated. Lactic acid and central venous oxygen saturation measurements were obtained in only one third of patients. An infectious organism was isolated in 51% of patients. The most common primary site of infection was central venous catheter (70%) and 5 patients (13%) showed a fungic infection. The mortality within 6 months was evaluated at 18.5%.

**Discussion**: Adherence to SCC recommandations for hematologic children with sepsis appeared inadequate. However, mortality was lower in our study than in other previous data.

**Conclusion**: Higher adherence to SSC recommandations for children with hematologic malignancies and better sensitization of medical and nurse staff would be potentially associated with much better prognosis.

### CO-31 High-flow nasal canula (HFNC) in infant hospitalized with moderate bronchiolitis- results of a multicenter open-label RCT in pediatric health care (Bronchopti study)

#### Philippe Durand (*speaker*)

##### Assistance Publique Hôpitaux de Paris, Le Kremlin-Bicêtre, FRANCE

###### **Correspondence:** Philippe Durand - philippe.durand2@aphp.fr

*Annals of Intensive Care* 2019, **9(Suppl 1)**:CO-31

**Introduction**: HFNC has emerged as a promising method to provide respiratory support in bronchiolitis. Only two RCT had evaluated HFNC in less severe bronchiolitis admitted in general wards but failed to demonstrate a reduction in the length of oxygen therapy or in the proportion of patient transferred in PICU (Kepreotes 2017, Franklin 2018). Therefore, we performed a superiority trial to test the hypothesis that HFNC could reduce the proportion of treatment failure requiring non-invasive ventilation among infants in these setting.

**Patients and methods**: Inclusion- first episode of bronchiolitis in infant (less 6 months-old), SpO2 in room air < 95%, m-WCAS score > or = 2 and < or = 5. Exclusion - urgent need for NIV, mWCAS > 5 or 6, lack of consent. Randomization - standard oxygen therapy (up to 2 L min)(control) or HFNC (3 l kg min)(experimental). Cross-over wasn’t allowed. Failure criteria- FiO2 > or = 40% or nasal flow oxygen > 2L min to maintain SpO2 target, increasing m-WCAS score or > 5–6, refractory apnea and or increasing PaCO2 at H6. Primary endpoint- proportion of patient in treatment failure requiring non-invasive within 7-days following randomization. Secondary endpoint- percentage admitted in ICU, assessment of short term respiratory status, general ward unit LOS, oxygen and nutritional - support free days.

**Results**: During the 6 months study period (2016–2017), 2630 patients admitted for bronchiolitis into the 17 PED’s network were screened of whom 271 underwent randomization (268 in the intention-to-treat analysis) (table 1). HFNC didn’t improved the primary outcome. Failure occurred in 21 of 133 patients (15%) in the HFNC group and 27 of 135 (20%) in the control group (OR IC 95% + 0.75 [0.40 + 1.40] + p = .36). HFNC didn’t reduced the risk of admission in ICU (21 (15%) in HFNC group versus 26 (19%) in control (OR IC 95% + 0.78 [0.41 + 1.41] + p = .45). Any patient was intubated. The main reason for treatment failure was the worsening of mWCAS score. Mean LOS on general ward unit was lower in the control group (3.8 + - 2.7 days versus 4.4 + -2.4 days + p = 0.04). Short-term assessment of respiratory status didn’t shown difference except for mWCAS score and RR in favor HFNC. Three pneumothorax was reported in HFNC group.

**Conclusion**: There was neither evidence of lower rate of non-invasive ventilation support among patients receiving HFNC therapy nor difference in the rate of ICU admission.

**Table:** 

Characteristics	HFNC group (N = 133)	Standard group (N = 135)	P
Age, Mean (SD)-d	68 ± 48	65 ± 46	0,53
Weight, Mean (SD)-kg	5,1 ± 1,5	4,9 ± 1	0,38
Female sex-no.(%)			0,135
Gestational age, Mean (SD)-weeks	38 ± 2	38 ± 2	0,92
-proportion of premature, no.(%)	16 (12%)	16 (11%)	0,9
Duration of symptoms before randomization, Mean (SD)-days	3,3 ± 2,1	3,1 ± 2,2	0,19
Temperature, Mean (SD) -°C	37,2 ± 0,6	37,2 ± 0,5	0,85
RR, Mean (SD) -bpm	53 ± 13	55 ± 14	0,34
Heart rate, Mean (SD)-bpm	156 ± 18	154 ± 18	0,51
SpO2, Mean (SD)-% in room air	90 ± 3	90 ± 3	0,70
m-WSCA score, Mean (SD)	3,3 ± 0,8	3,1 ± 0,7	**0,028**
PtCO2, Mean (SD)-mmHg	50 ± 11	50 ± 10	0,59
pH, Mean (SD)	7,34 ± 0,07	7,33 ± 0,05	0,17
Viral cause-no./total no.(%)			
Number tested, no	103	105	0,94
RVS status, no. (%)	85 (82%)	87 (82%)	0,27

### CO-32 Prospective bicentric observationnal study of non-invasive ventilation in pediatric acute chest syndrome - the nivipacs cohort

#### Charlotte Idier (*speaker*)^1^, Pierre-Louis Leger^2^, Julie Sommet^3^, Julia Guilbert^1^, Fleur Le Bourgeois^3^, Jérôme Naudin^3^, Michael Levy ^3^, Stephane Dauger^3^

##### ^1^CHU Gatien de Clocheville, Tours, FRANCE; ^2^Hopital Armand Trousseau, AP-HP, Paris, FRANCE; ^3^Hôpital Robert Debré APHP, Paris, FRANCE

###### **Correspondence:** Charlotte Idier - charlotte.idier@gmail.com

*Annals of Intensive Care* 2019, **9(Suppl 1)**:CO-32

**Introduction**: Acute chest syndrome (ACS) is one of the most frequent and severe manifestations of sickle cell disease in childhood. The physiopathological mechanisms of ACS are complex and resulted in severe abnormalities of ventilation perfusion ratio. Our hypothesis is that non-invasive ventilation (NIV) could be an important component of ACS treatment. The main objective of this study is to describe the tolerance of NIV in the pediatric ACS.

**Patients and methods**: Bicentric prospective study from 01 09 2016 to 01 09 2017, including all sickle cell patients admitted to pediatric intensive care unit (PICU) for ACS. The main composite objective was to describe the tolerance of NIV by identifying the following four components- i) frequency of use, ii) effects on work of breathing evaluated by the clinical respiratory score (CRS), iii) circumstances of NIV weaning, iv) child’s comfort scored by the COMFORT scale, and the occurrence of complications.

**Results**: From the thirty-seven children included, 30 (81%) were ventilated with NIV for 34 [22 + 53] hours for a duration of stay in PICU of 3.0 [2.0 + 4.1] days. All children were discontinuously ventilated in Pressure Support (PS) mode. The CRS dropped rapidly between H0 and H4- from 6 [4 + 7] at H0 to 4 [3 + 6] at H4 (p = 0.0006), without change in the SpO2 FIO2 ratio during the same time (385 [320 + 463] versus 398 [331 + 438], p = 0.25). The COMFORT score was the same with or without NIV. We did not report any NIV complication.

**Conclusion**: The majority of children admitted to PICU with an ACS are broken down into NIV in PS mode. NIV was very well tolerated and was able to decrease respiratory distress in the first hours without modifying oxygen requirement.

### CO-33 Association between early endotracheal intubation and ICU mortality in septic shock - a prospective multicentric observational study

#### Sophie Jacquier (*speaker*)^1^, Agathe Delbove^2^, Cédric Darreau^3^, Marjorie Saint-Martin^3^, Frédéric Martino^5^, Jean-François Hamel^6^, Mai-Anh Nay^7^, Nicolas Terzi^8^, Geoffrey Ledoux^9^, Ferran Roche-Campo^10^, Laurent Camous^11^, Frédéric Pene^12^, Thibault Balzer^13^, François Bagate^14^, Julien Lorber^15^, Pierre Bouju^16^, Clémence Marois^17^, René Robert^18^, Stéphane Gaudry^19^, Morgane Commereuc^20^, Matthieu Debarre^21^, Nicolas Chudeau^2^, Pierre Labroca^22^

##### ^1^CHRU, Tours, FRANCE; ^2^CHU, Nantes, FRANCE; ^3^CH, Le Mans, FRANCE; ^5^CHU Guadeloupe, Abymes, FRANCE; ^6^CHU, Angers, FRANCE; ^7^CH, Orléans, FRANCE; ^8^CHU, Grenoble, FRANCE; ^9^CHRU, Lille, FRANCE; ^10^Hospital of Santa Creu i Sant Pau, Barcelone, SPAIN; ^11^CHU Bicetre, Paris, FRANCE; ^12^CHU Cochin, Paris, FRANCE; ^13^CHU, Brest, FRANCE; ^14^CHU Mondor, Paris, FRANCE; ^15^CH, La Roche-Sur-Yon, FRANCE; ^16^CH, Lorient, FRANCE; ^17^CHU La Pitié Salpétrière, Paris, FRANCE; ^18^CHU, Poitiers, FRANCE; ^19^CHU Colombes, Paris, FRANCE; ^20^CHU HEGP, Paris, FRANCE; ^21^CH, Saint-Brieuc, FRANCE; ^22^CHU, Nancy, FRANCE

###### **Correspondence:** Sophie Jacquier - sophiejacquier45@gmail.com

*Annals of Intensive Care* 2019, **9(Suppl 1)**:CO-33

**Introduction**: By contrast to neurological and respiratory failure, the place for endotracheal intubation and mechanical ventilation is not detailed in guidelines on septic shock, evidencing a lack of dedicated studies. The objective of this study was to assess the association between early endotracheal intubation (before H8 following vasopressor initiation - H0) and outcome (ICU survival) in septic shock patients, taking into account presence or absence of classical intubation criteria (i.e. neurologic and or respiratory failure).

**Patients and methods**: This prospective multicenter observational study was conducted from May 2016 to October 2017 in 32 ICUs (France and Spain). All successive adult patients suffering from septic shock and admitted in participating ICUs were considered. Criteria defining three groups of patients were defined a priori according to the potential neurologic respiratory motivation of intubation between H0 and H8—1) “classical indication group” (CIG) in presence of straightforward indication for endotracheal intubation, 2) “intermediate group”(IG) in presence of moderate impairment, 3) and non-classical motivation group (NCG) in absence of significant impairment. Then, a propensity score was constructed to establish the probability of being intubated.

**Results**: Eight hundred and fifty-nine patients were recruited. Two hundred and twenty-six patients were sorted into the CIG, 329 into the IG and 190 into the NCG. Early intubation was performed in 51% of the CIG, 22% of IG and 9% in NCG. Multivariate analysis showed that groups (CIG > IG > NCG), higher SAPS II and early intubation (OR = 1.65 [1.07–1.56]) vs. no early intubation, were independently associated with higher ICU mortality. The propensity score was constructed entering 586 patients. An increased risk of death was observed throughout the range of the propensity score in early intubated patients vs. not early intubated patients, all the more that the propensity score was increased (p < 0.001), see figure *In a multivariate analysis, delayed intubation showed no impact on mortality vs. early intubation with OR = 0.99 [0.52–1.87] + p = 0.97 for patients intubated between H8-H24 vs. patients intubated before H8 and OR = 1.66 [0.76–3.65] + p = 0.21 for patients intubated between H24 and H72.

**Conclusion**: An association between early intubation and higher mortality was observed both in a multivariate model and using a propensity score in septic shock patients. This study opens the way for a prospective interventional trial.


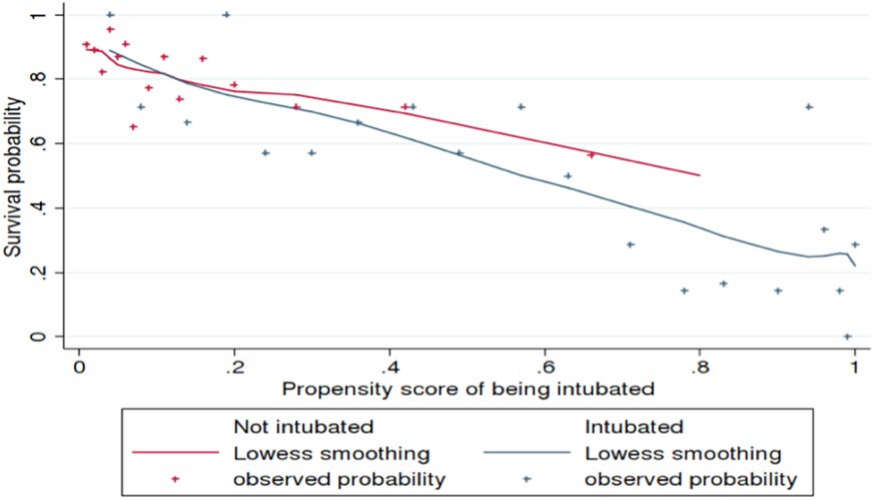


### CO-34 VA-ECMO to rescue refractory septic shock with severe myocardial dysfunction

#### Nicolas Bréchot (*speaker*)^1^, David Hajage^1^, Julien Demiselle^2^, Antoine Kimmoun^3^, Santi Montero^1^, Matthieu Schmidt^1^, Guillaume Hékimian^1^, Guillaume Lebreton^1^, Charles-Edouard Luyt^1^, Elie Zogheib^4^, Erwan Flecher^5^, Daniel Brodie^6^, Bruno Levy^3^, Pierre Asfar^2^, Alain Combes^1^

##### ^1^La Pitié-Salpêtrière University Hospital, Paris, FRANCE; ^2^Angers University Hospital, Angers, FRANCE; ^3^Nancy-Barbois University Hospital, Vandoeuvre-Lès-Nancy, FRANCE; ^4^Amiens University Hospital, Amiens, FRANCE; ^5^Rennes University Hospital, Rennes, FRANCE; ^6^Columbia University Medical Center, New-York, UNITED STATES

###### **Correspondence:** Nicolas Bréchot - nicolas.brechot@aphp.fr

*Annals of Intensive Care* 2019, **9(Suppl 1)**:CO-34

**Introduction**: Some patients with septic shock may develop very severe myocardial dysfunction refractory to medical treatments. Several cohorts and case-report studies reported salvage therapy with veno-arterial extracorporeal membrane oxygenation (VA-ECMO) in those patients. The aim of this study was to assess the usefulness of VA-ECMO as rescue therapy during refractory myocardial dysfunction associated with septic shock.

**Patients and methods**: In this multicenter international retrospective study, patients from 4 ECMO centers implanted with VA-ECMO as a rescue therapy from 2008 to 2018 for refractory septic shock with severe myocardial dysfunction (cardiac index below 3 L min m2 and or left ventricular ejection fraction below 35%) were included. As controls, 228 patients exhibiting severe myocardial dysfunction during the same period of time but who did not receive ECMO were isolated from 3 large septic shock databases, and were included the first day they met criteria for severe myocardial dysfunction. ECMO and non-ECMO patients outcomes were compared after matching for disease severity. The primary end point was mortality at 90 days.

**Results**: (mean ± SD and n (%) are shown) Eighty-two patients rescued with VA-ECMO were included. Patients exhibited extremely severe myocardial dysfunction (mean cardiac index at 1.54 ± 0.54 L min m2 and left ventricular ejection fraction 28.1 ± 5.7%), despite very high doses of catecholamines (mean inotropic score 279 ± 247 µg kg min). They also had profound lactic acidemia (pH 7.13 ± 0.15 and lactatemia 8.9 ± 4.4 mmol/L) and severe multiple organ failure (SOFA score 16.6 ± 2.9 and SAPS-II score 78.3 ± 16.1). Seventy-eight of them could be matched with non-ECMO patients based on a propensity score for disease severity (lactatemia, cardiac index and SOFA score). Mortality at 90 days was dramatically reduced in matched ECMO patients compared to controls, (41% vs. 87%, p < 0.0001). In a sensitivity analysis, all patients with cardiac index monitoring were weighted using matching weight ponderation on inotropic score, lactatemia, SOFA score, age and cardiac index at inclusion. Mortality at 90 days remained significantly reduced in weighted ECMO patients. Finally, survivors in the ECMO group reported acceptable SF-36 evaluated health-related quality of life in the months following ICU discharge.

**Conclusion**: In this retrospective propensity-matched study, rescue therapy with VA-ECMO was associated with a dramatic reduction in 90 days mortality for patients with refractory septic shock and severe myocardial dysfunction.

### CO-35 Persistent fluid responsiveness is infrequent after fluid expansion- a multicenter, prospective physiological study

#### Hélène Beringuer (*speaker*)^1^, Antoine Kimmoun^2^, Pierre-Eric Danin^3^, Julien Pottecher^4^, Florence Fagot-Gandet^4^, Eliane Albuisson^2^, Xavier Monnet^5^, Jean-Louis Teboul^5^, Martin Dres^1^

##### ^1^APHP, Paris, FRANCE; ^2^CHRU de Nancy, Vandoeuvre-Les-Nancy, FRANCE; ^3^CHU, Nice, FRANCE; ^4^Hôpitaux Universitaires de Strasbourg, Strasbourg, FRANCE; ^5^Hôpital de Bicêtre, Le Kremlin-Bicêtre, Le Kremlin-Bicêtre

###### **Correspondence:** Hélène Beringuer - helene.beringuer@wanadoo.fr

*Annals of Intensive Care* 2019, **9(Suppl 1)**:CO-35

**Introduction**: Fluid expansion is the first therapeutic option in patients presenting acute circulatory failure but its hemodynamic effects (persistency and time of maximal increase in cardiac output) are unknown. We sought to describe the time course of cardiac output over a 2-hours period after a fluid expansion. Our objectives were 1) to identify patterns of fluid responsiveness and 2) to determine the time of maximal increase in cardiac output during and after fluid expansion.

**Patients and methods**: It was a prospective multicentre observational study conducted in four intensive care units. To be included, mechanically ventilated patients with acute circulatory failure (infusion of norepinephrine) had to be equipped with a transpulmonary thermodilution device (PiCCO 2) and a decision of fluid expansion (500 ml of saline over a standardized 10-minutes period) had to be made. Calibration was achieved before and at two hours after fluid expansion. Transpulmonary thermodilution derived indices were collected over the two-hours period of observation. Fluid responsiveness was defined as an increase in cardiac index 15% from the start of the fluid expansion. Four patterns of fluid responsiveness were predefined- never responders, ultrafast responders (before the end of the fluid expansion), short-term responders (at the end of the fluid expansion) and persistent responders (over the 2-hours observation period). No change in drugs dosage nor ventilatory settings were allowed during the study.

**Results**: Fifty-eight patients (79 cases) were included. Septic shock was the main reason of acute circulatory failure (46 58, 79%). Patterns of fluid responsiveness were the followings- non-responders (36 79, 46%), ultrafast responders (3 79, 4%), short-term responders (13 79, 16%) and persistent responders (6 79, 8%). In addition, 21 (27%) patients had hemodynamic instability and required a second fluid expansion during the protocol. No significant difference was found between patterns in terms of baseline hemodynamic characteristics nor drugs administrated. The increase in cardiac output was maximal after 300 [189–411] seconds in never responders, after 516 [396–672] seconds in ultrafast responders, after 456 [360–660] seconds in short-term responders and after 348 [315–372] seconds in persistent responders.

**Conclusion**: Persistent fluid responsiveness occurred in a minority of patients presenting acute circulatory failure. Maximal increase in cardiac output could be observed during fluid expansion between 5 and 10 min.

### CO-36 Molar Sodium Lactate Improves Mesenteric MicroCirculation, Cardiac Function, Capillary Leakage and Inflammation in a Rat Sepsis Model

#### Emmanuel Besnier (*speaker*)^1^, David Coquerel^2^, Geoffrey Kouadri^1^, Mathieu Soulié^3^, Nicolas Perzo^3^, Raphael Favory^4^, Thibault Duburcq^4^, Olivier Lesur^2^, Soumeya Bekri^5^, Vincent Richard^3^, Paul Mulder^3^, Fabienne Tamion^6^

##### ^1^Department of Anesthesia and Critical Care, Rouen University Hospital, Rouen, FRANCE; ^2^Université de Sherbrooke, Division of Intensive Care Units, Centre de Recherche Clinique du CHUS, Sherbrooke, CANADA; ^3^Normandie Univ, Unirouen, Inserm U1096 EnVi, Rouen, FRANCE; ^4^Intensive care unit, Lille University Hospital, Lille, FRANCE; ^5^Institute of Clinical Biology, Rouen University Hospital, Rouen, FRANCE; ^6^Department of Medical Intensive Care, Rouen University Hospital, Rouen, FRANCE

###### **Correspondence:** Emmanuel Besnier - besnier.emmanuel@gmail.com

*Annals of Intensive Care* 2019, **9(Suppl 1)**:CO-36

**Introduction**: Fluids composed of molar sodium lactate have recently been identified as beneficial in endotoxinic animal models1. The objective of our work was to evaluate the effects of molar sodium lactate on micro- and macro-circulation, capillary leakage and different biological parameters in a sepsis rat model of caecal ligation and puncture (CLP).

**Patients and methods**: We realized CLP in 30 rats randomized in 3 groups (n = 10 per group)- Sham + CLP-NaCl 0.9% + CLP-Lactate 11.2%. Immediately after CLP, fluids were infused intravenous (2.5 mL kg h) during 18 h. Then, we evaluated mesenteric microcirculation (laser speckle imager), cardiac function (echocardiography) and inflammation (uremia, albuminemia, VEGF-A, IL-1β, IL-10, TNFα). Assay of capillary leakage using Blue Evans extravasation in the lung and gut were realized on additional rats (n = 5 group). Results are expressed as medians with interquartiles and comparisons versus CLP-NaCl were realized using Kruskall-Wallis or ANOVA test.

**Results**: Mesenteric microcirculation was lower in CLP-NaCl vs. Sham (240.6 [209.3–390.8] vs. 935.9 [855.0–1067.0] pixels unit, p < 0.0001) and CLP-Lactate (735.5 [407.4–878.8], p = 0.0006). CLP-NaCl rats presented a lower cardiac output vs. Sham (0.14 [0.10–0.18] vs. 0.30 [0.26–0.34] mL/min g, p = 0.004) and CLP-lactate (0.34 [0.28–0.43], p < 0.0001) and lower left ventricular shortening fraction vs. CLP-Lactate (39.1 [32.9–51.8] vs. 55.2 [46.2–73.2]  %, p = 0.009). There was no difference concerning albuminemia, left ventricular diastolic diameter, central venous pressure or E A mitral flow ratio between CLP-NaCl and CLP-lactate, suggesting comparable volemia. Mean arterial pressure between CLP-NaCl and CLP-Lactate was similar at the end of infusion. Evans Blue diffusion was reduced in the gut and the lung for CLP-lactate (37.2 [31.0–43.3] vs. 112.7 [63.3–141.6] and 107.5 [82–174.3] vs. 272.7 [221.8–444.5] ng EB mg of tissue). Plasma levels of lactate and 3OH-butyrate were higher in CLP-lactate vs. CLP-NaCl (6.03 [3.08–10.3] vs. 3.19 [2.42–5.11] mmol/L, p = 0.04 + 400 [174–626] vs. 189 [130–301] µmol L, p = 0.03), but no difference for plasma pyruvate or acetoacetate. Inflammatory response was reduced in CLP-lactate (IL-1β- 172.2 [119.0–446.3] vs. 927.7 [244.8–1470] pg mL, p = 0.004 + TNFα- 17.9 [12.5–50.3] vs. 53.9 [30.8–85.6] pg mL, p = 0.005 + IL-10- 351.6 [267.0–918.6] vs. 904.5 [723.1–1243] pg mL) as well as VEGA-A plasma levels (198.2 [185.3–250.0] vs. 260.7 [249.8–268.9] pg mL, p = 0.009). No difference was observed for syndecan plasma level.

**Conclusion**: We demonstrate for the first time that molar lactate fluid perfusion protects against CLP-induced cardiovascular dysfunction, mesenteric microvascular alteration and capillary leakage, in association with a significant reduction in inflammatory process. These results suggest that molar lactate fluid perfusion may be an attractive target for the treatment of sepsis.

### CO-37 Severe pneumocystis jiroveci pneumonia in human immunodeficiency virus negative patients

#### Julien Grouille (*speaker*)^1^, Jean Baptiste Lascarrou^2^, Delphine Chatellier^3^, Mickael Landais^4^, Pierre Fillatre^5^, Thomas Daix^6^, Virginie Verrier ^7^, Jean Claude Lacherade^8^, Grégoire Muller^9^, Marc Feller^10^, Michel Hira^11^, Maud Jonas^12^, Denis Garot^1^

##### ^1^CHRU Bretonneau, Tours, FRANCE; ^2^CHU, Nantes, FRANCE; ^3^CHU, Poitiers, FRANCE; ^4^CH Le Mans, FRANCE; ^5^CH, Saint Brieuc, FRANCE; ^6^CHRU, Limoges, FRANCE; ^7^CH, La Rochelle, FRANCE; ^8^CH, La Roche Sur Yon, FRANCE; ^9^CHR, Orleans, FRANCE; ^10^CH, Blois, FRANCE; ^11^CH, Chateauroux, FRANCE; ^12^CH, Saint Nazaire, FRANCE

###### **Correspondence:** Julien Grouille - julien.grouille@outlook.fr

*Annals of Intensive Care* 2019, **9(Suppl 1)**:CO-37

**Introduction**: Pneumocystis jiroveci pneumonia (PCP) is one of the main opportunistic complications in patients infected with the human immunodeficiency virus (HIV), but it also affects non-HIV-infected patients, with mortality rates of 30 to 60%. We have described the population of HIV-negative patients hospitalized in intensive care units (ICU) for PCP.

**Patients and methods**: A French multicenter retrospective study performed in 12 ICUs from ARCO (Association des Réanimateurs du Centre Ouest) between January 1st 2012 and December 31st 2017.

**Discussion**: 117 patients were included, mean age 63.9 years. The main underlying diseases were hematological diseases (46 cases, 39.3%). 99 patients (83.9%) received chemotherapeutic treatments, 77 (65.8%) were on steroids. Of 44 patients undergoing steroids potentially**eligible for PCP prophylaxis, 43 did not received it (97.7%).

105 patients (89.7%) had dyspnea as their main symptom. A biological inflammatory syndrome was present in 116 cases**(98.8%). Chest CT revealed an interstitial lung disease for 53 patients (65.4%). The diagnosis was made during bronchial fibroscopy with bronchoalveolar lavage (BAL) in 106 cases (90.6%) and by non-invasive method (specific sputum PCR, B D glucan assay) in 11 cases (9.4%) with a mean diagnostic delay after admission of 4.5 days.

65 subjects (55.5%) were mechanically ventilated (average duration of 15.5 days). 35 patients (30.3%) developed septic shock with vasopressor support (average duration of 6.4 days). 39 patients (33.3%) had acute renal failure according to the KDIGOcriteria. The average length of stay in ICU was 14.8 days. 36 patients (30.7%) were treated for a possible PCP upon admission and the average treatment time for the other patients was 3.9 days. 112 (97.5%) received curative treatment with cotrimoxazole and 26 (23.3%) developed side effects. Steroids were associated with anti-infective treatment for 96 patients (82%) with no impact on evolution. The mortality rate in ICU was 46 (39.3%).

**Conclusion**: PCP is an increasingly infection over immunosuppressed non HIV infected patients. Our study confirms the severity of this pathology and the main populations at risk. PCP should be mentioned in cases of pulmonary involvement in at-risk patients and a probabilistic treatment should be implemented quickly. The interest of an adjuvant steroid treatment remains to be demonstrated. Recommendations on prophylactic treatment should be more widely distributed and probably further refined.

No authors have any conflicts of interest.

### CO-38 Candidemia in critically ill immuno-compromised patients

#### Etienne Ghrenassia (*speaker*)^1^, Djamel Mokart^2^, Julien Mayaux^3^, Alexandre Demoule^3^, Imène Rezine^1^, Lionel Kerhuel^1^, Laure Calvet^1^, Audrey De Jong^1^, Elie Azoulay^1^, Michael Darmon^1^

##### ^1^Hôpital Saint Louis, Paris, FRANCE; ^2^Insititut Paoli-Calmettes, Marseille, FRANCE; ^3^Hôpital Pitié Salpétrière, Paris, FRANCE

###### **Correspondence:** Etienne Ghrenassia - etienne.ghrenassia@gmail.com

*Annals of Intensive Care* 2019, **9(Suppl 1)**:CO-38

**Introduction**: Candidemia is a major threat for patients with cancer. This study sought to identify outcomes of critically ill immunocompromised patients with candidemia. Our secondary objectives were to describe the clinical phenotype of patients, Intensive Care Units (ICU) features, Candida ecology, and factors associated with hospital mortality.

**Patients and methods**: Adult critically ill immunocompromised patients with candidemia were retrospectively included from 3 ICUs. Immune defect was defined as an underlying solid tumor, hematological malignancy or autoimmune disease.

**Results**: Overall, among 31792 patients admitted in 3 ICUs over a 15-y period (2002 to 2017), 219 developed a Candidemia (0.7%). Of these, 121 had an underlying immune defect and were ultimately included. Two third of the patients had hematological malignancy, chiefly non-Hodgkin’s lymphoma (37%), and acute leukemia (21%). Respectively, 10% and 7% of included patients underwent autologous or allogeneic stem cell transplantations. Most patients had one or more risk factors of candidemia. During ICU stay, 91 (75%) patients needed invasive mechanical ventilation, 71 (61%) patients needed renal replacement therapy, and 91 (75%) patients needed vasopressors. One out of five patients (21%) was considered as emergency surgery patients. Candida albicans (54%) was the predominant specie, followed by glabrata (19%), tropicalis (11%), parapsilosis (7%) and krusei (7%). One third of identified Candida was resistant to Fluconazole (30%) and 8% to Echinocandins. ICU mortality was 52% and hospital mortality was 60%. Candida specie or susceptibility was not associated with outcome (figure). Median time from feature of candidemia onset to first antifungal therapy was 3 (1–3) days. After adjustment for confounders, need for vasopressors (HR- 1.8 + CI95%- 1.1–3.1), need for mechanical ventilation (HR- 2.0 CI95%- 1.1–3.8) and allogeneic stem cell transplantation (HR- 2.5 + CI95%- 1.1–6.0) were independently associated with hospital mortality. Half the patients (58%) developed candidemia more than 24 h after ICU admission and 42% were admitted in ICU for severe candidemia. There was no difference in clinical or Candida features or outcomes between these two kinds of patients.

**Conclusion**: Candidemia in critically ill immunocompromised patients is associated with high mortality rates. Despite high prevalence of non-albicans Candida, only severity and preexisting allogeneic stem cell transplantation were independently associated with poor outcome. Last, antifungal therapy proved to be started late as regard to symptoms. Then, development of strategies to reduce this delay are warranted.

### CO-39 Dynamic risk factors of invasive Candida infection in intensive care unit- a landmark method

#### Clément Le Bihan (*speaker*)^1^, Sébastien Bailly^1^, Maité Garrouste-Orgeas^1^, Elie Azoulay^2^, Lila Bouadma^3^, Carole Schwebel^4^, Claire Dupuis^1^, Guillaume Marcotte^5^, Yves Cohen^6^, Bertrand Souweine^7^, Laurent Papazian^8^, Jean Reignier^10^, Stéphane Ruckly^11^, Jean-Francois Timsit^1^

##### ^1^UMR 1137 - IAME Team 5 - DeSCID- Decision SCiences in Infectious Diseases, control and care Inserm University Paris Diderot, Sorbonne Paris Cité, Paris, FRANCE; ^2^Medical ICU, Saint-Louis Hospital, APHP, Paris, FRANCE; ^3^AP-HP, Bichat Hospital, Medical and infectious diseases Intensive Care Unit, Paris Diderot university, Paris, FRANCE; ^4^Medical ICU, Albert Michallon University Hospital, Grenoble, FRANCE; ^5^Surgical ICU, Edouard Herriot University Hospital, Lyon, FRANCE; ^6^AP-HP, Avicenne Hospital, Intensive Care Unit, Paris and Medicine University, Paris 13 University, Bobigny, FRANCE; ^7^Medical ICU, Gabriel Montpied Hospital, Clermont-Ferrand, FRANCE; ^8^Respiratory and infectious diseases ICU, APHM Hôpital Nord, Aix Marseille University, Marseille, FRANCE; ^10^Medical ICU, Hôtel-Dieu University Hospital, Nantes, FRANCE; ^11^ICU REsearch, Department of Biostatistics, Paris, FRANCE

###### **Correspondence:** Clément Le Bihan - clement.lebihan@gmail.com

*Annals of Intensive Care* 2019, **9(Suppl 1)**:CO-39

**Introduction**: Invasive candidiasis (IC) is associated with a high incidence of mortality in intensive care unit (ICU). Delayed antifungal therapy for IC contributes to poor outcomes in ICU. None of the predictive published risk scores are able to accurately predict IC. Therefore, new scores introducing previous anti-infective therapy, elapsed time between admission, risk evaluation and differentiating candidemia and other invasive candidiasis should be developed.

**Patients and methods**: Retrospective analysis of data from the OUTCOMEREA prospective multicenter cohort. Critically ill patients with an ICU length of stay of at least 3 days were included. Multivariable sub-distribution survival models with competing risk were used at each day, from day 1 to day 10 using a landmark method with a sliding window and an horizon of 10 days. The impact of every identified risk was analysed for candidemia and invasive visceral candidiasis (IVC- Candida peritonitis or pleural candidiasis) separately. Competing risks were ICU-death and ICU-discharge. Predicted probability of infection during the next 10 days was calculated at day 1, 4 and 7 based on cumulative incidence function of every combination of risk.

**Results**: 16901 patients were included. Candidemia occurs in 96 patients and IVC in 87 patients. Antifungal therapy was used in 2019 (12%) patients. In multivariate analysis, 10 risk factors for candidemia and 12 for IVC were identified (figure). We confirmed the impact of some previously described risk factors (i.e. septic shock, multifocal colonization, SOFA score, hospital stay before ICU, parenteral nutrition, corticosteroids, blood transfusion, mechanical ventilation and surgery). Those risks were differently associated with candidemia and IVC. Some variables such as corticosteroids therapy, blood transfusion and mechanical ventilation were only associated with candidemia. Risks were time dependent with some having more impact on early state (landmark time < 5) or on late onset (landmark time > day 5). Antibiotherapy (broad spectrum, piperacillin-tazobactam), number of PPI days of use and a medical history of cancer were only associated with IVC after landmark time 5. Prediction model found maximum predicted probability of 38, 17 and 16% at landmark 1, 4 and 7 respectively for candidemia and 58, 52 and 41% for IVC. A shiny app was developed to compute the predicted risk of candidemia or IVC depending on risk factors combination.

**Conclusion**: Risk factors of Candida infection are different for candidemia and IVC and must be analysed as distinct diseases. Moreover, those risks are highly time-related with an early and late pattern during ICU stay.


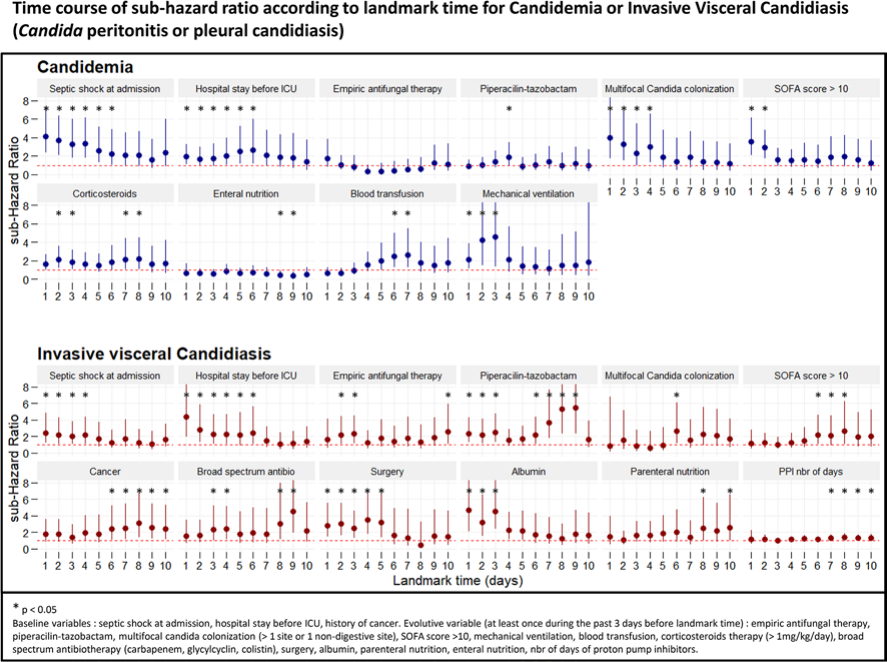


### CO-40 Zygomycosis in Intensive care unit- surgery as a major prognostic factor

#### Johanna Claustre (*speaker*)^1^, Thomas Jouve^1^, Romaric Larcher^3^, Saad Nseir^4^, Julien Cadiet^5^, Yoann Zerbib^6^, Alexandre Lautrette ^7^, Jean-Michel Constantin^7^, Pierre-Emmanuel Charles^8^, Cédric Daubin^9^, Remy Coudroy^10^, Jean Dellamonica^11^, Laurent Argaud^12^, Pierre Phelouzat^13^, Damien Contou^14^, Juliette Pocquet^15^, Guillaume Voiriot^16^, Jean-Christophe Navellou^17^, Pierre Lavagne^18^, Michel Durand^19^, Carole Schwebel^20^, Nicolas Terzi^20^

##### ^1^CHU Grenoble Alpes, Grenoble, FRANCE; ^3^CHU, Médecine intensive et Réanimation, Montpellier, FRANCE; ^4^CHU, Urgence respiratoire et réanimation médicale, Lille, FRANCE; ^5^Service de médecine intensive Réanimation, Nantes, FRANCE; ^6^Réanimation Médicale, Amiens, FRANCE; ^7^Réanimation Médicale, Clermont-Ferrand, FRANCE; ^8^Médecine intensive Réanimation, Dijon, FRANCE; ^9^Réanimation Médicale, Caen, FRANCE; ^10^Réanimation Médicale, Poitiers, FRANCE; ^11^Réanimation Médicale, Nice, FRANCE; ^12^Réanimation Médicale, Lyon, FRANCE; ^13^Réanimation Médicale, Rennes, FRANCE; ^14^Réanimation polyvalente, Argenteuil, FRANCE; ^15^Médecine intensive Réanimation, Tours, FRANCE; ^16^Réanimation médico-chirurgicale, Paris, FRANCE; ^17^Réanimation Médicale, Besançon, FRANCE; ^18^Réanimation polyvalente chirurgicale, Grenoble, FRANCE; ^19^Réanimation cardiovasculaire et thoracique, Grenoble, FRANCE; ^20^Médecine intensive Réanimation, Grenoble, FRANCE

###### **Correspondence:** Johanna Claustre - jclaustre@chu-grenoble.fr

*Annals of Intensive Care* 2019, **9(Suppl 1)**:CO-40

**Introduction**: Zygomycosis is an invasive fungal infection, with an increasing incidence especially in patients with hematological malignancy. Its prognosis is poor because of its high invasive power, leading to fungal dissemination and tissular necrosis. No study aimed to describe patients with zygomycosis admitted in intensive care units (ICU). We aimed to describe epidemiology of zygomycosis in French ICU and evaluate outcomes.

**Patients and methods**: We performed a retrospective multi-center study in 16 French ICUs (universitary hospitals or general hospitals) between 2008 and 2017. During this period, 74 patients were diagnosed as having probable or proven zygomycosis. We reported demographic baseline and evolutive clinical data and compared the patients who survived in ICU (21 patients) and the patients who did not (53 patients) with univariate and multivariate analyses to identify factors associated with ICU survival. Then, we also focus on the subgroup of patients with hematological malignancy.

**Results**: Sixty of the 74 patients admitted in ICU and having zygomycosis were deeply or mildly immunocompromised- 41 had an hematological malignancy, 4 had a solid malignancy, 9 were solid organ transplant recipients, 2 had an inflammatory disease, 31 had long-term steroids, 11 had a diabetes, 24 had a malnutrition. Among the 74 patients, only 21 patients survived to ICU stay (28.4%) with a median survival of 22 days (Q1-Q3 = 9–106). In the univariate and multivariate analyses, survivors were significantly younger (p < 0.001), hematological malignancy was less frequent (p = 0.003), as well as malnutrition (p = 0.02). Trauma was associated with better survival (p = 0.02) in univariate analysis, while all patients with a disseminated infection (2 or more non-contiguous infected organ) died in ICU (p = 0.007). **Regarding patients with hematological malignancy, median survival (n = 41) was 15 days (Q1-Q3 = 5–23.5 days), worse than 22 days (Q1-Q3 = 9–106) in the whole cohort. In multivariate analysis, surgery was the only factor associated with survival (p = 0.02)- no long-term survival was observed in the subgroup of patients without surgical management (Log-rank p < 0.001, Figure). The association of two antifungal treatment did not demonstrate any benefit in survival (p = 0.21).

**Conclusion**: Overall prognosis of zygomycosis in ICU remains poor, especially in patients with hematological malignancy. Despite an increased frailty, an aggressive management including early surgery was the only factor improving survival in this ICU population.


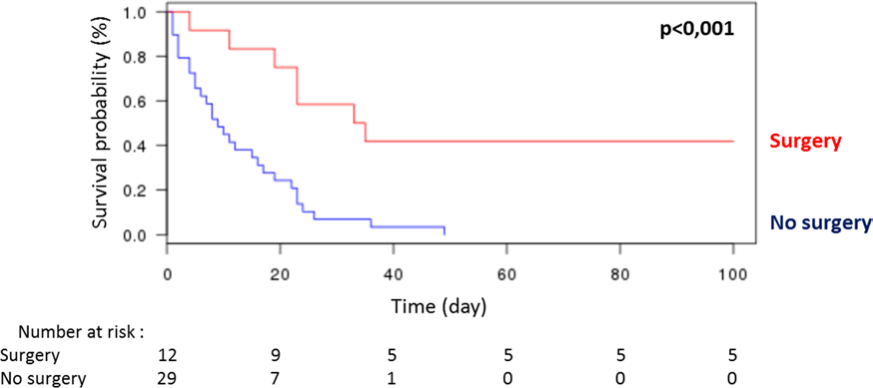


### CO-41 Clinical features and outcome of life-threatening Adult Onset Still Disease- a French multi center retrospective study

#### Lucie Mortier (*speaker*)^1^, Marc Pineton de Chambrun^2^, Eliane Albuisson^3^, Nathalie Lerolle^4^, Matthieu Groh^5^, Philippe Guiot^6^, Mathilde Neuville ^7^, Jérémie Joffre^8^, Damien Du Cheyron^9^, Roland Jaussaud^10^, Pierre-Edouard Bollaert^1^, Jérémie Lemarié^1^

##### ^1^Service de Médecine Intensive Réanimation, Nancy, FRANCE; ^2^Hôpital de la Pitié Salpêtrière - AP-HP, Paris, FRANCE; ^3^Unité de Méthodologie, Datamanagement & Statistiques, Vandoeuvre-Les-Nancy, FRANCE; ^4^Service de Médecine Interne Hôpital Bicêtre, APHP, Le Kremlin-Bicêtre, FRANCE; ^5^Service de Médecine Interne Hôpital St Louis, Paris, FRANCE; ^6^Grpe Hosp Region Mulhouse & Sud Alsace, Hopital Emile Muller, Mulhouse, FRANCE; ^7^Réanimation Médicale et Infectieuse Hôpital Bichat-Claude Bernard, Paris, FRANCE; ^8^Service de réanimation médicale, Paris, FRANCE; ^9^Service de réanimation médicale, Caen, FRANCE; ^10^Service de Médecine Interne Hôpital St Louis, Vandoeuvre-Les-Nancy, FRANCE

###### **Correspondence:** Lucie Mortier - mortierlucie@gmail.com

*Annals of Intensive Care* 2019, **9(Suppl 1)**:CO-41

**Introduction**: Adult onset Still disease (AOSD) is a rare and complex auto-inflammatory disorder with several systemic life-threatening manifestations and empirical treatment. Our objectives were to describe of clinical features, management and outcome of AOSD patients who required intensive care support.

**Patients and methods**: We undertook a 17-year retrospective multicenter cohort study in 44 French academic ICUs from January 2001 to June 2017 including all cases of ICU admission related to life-threatening AOSD. All patients older than 18 years were considered for inclusion if they met the following criteria- (1) admission to ICU, (2) related to systemic AOSD flare (according to Yamaguchi and or Fautrel criteria). The study methodology and the data privacy policy (French methodology of reference MR-003) were approved by our institutional ethics committee (application no. 156-2017) and registered on ClinicalTrials.gov (NCT03276650).

**Results**: Forty-five patients were included (median age 34.5 (1st-3rd quartiles 24–42.5) years). Men were more affected (54%) than women. It was the first AOSD flare for 60% of population. Median SAPS II was 32.5 (21.5–44). Cardio-circulatory (shock, 42% and pericarditis, 40%), respiratory (Acute Respiratory Distress Syndrome, 22%) and hematologic (Reactive Hemophagocytic Syndrome, 16%) disorders were the most frequent life-threatening manifestations. Multiple organ failure was frequent (24%) and associated with bad prognosis if presented at admission (p = 0.021). Nine patients died (20%, median age of 24 (21–41) years) among whom 8 with previously diagnosed AOSD, versus 10 without previous diagnosis (p = 0.001). Comparison between survivors and non-survivors during ICU stay are shown in Table 1. Corticosteroids (CS) were used as first line therapy in 43 (96%) patients with efficacy of CS monotherapy in 27 (63%). Three patients were successfully treated by anakinra, with 43% global efficacy. Four patients with more severe organ failures at admission died despite treatment with anakinra, introduced after several lines of other immunosuppressive agents (cyclosporine, etoposide, intravenous immunoglobulins).

**Conclusion**: Herein, we report the largest cohort of patients admitted to ICU for life-threatening AOSD. It can lead to life-threatening presentation with miscellaneous presenting clinical spectrum and high mortality rate among young people with previous diagnosis of AOSD. Because of the lack of specific clinical and biological signs and its frequent cardiovascular collapse, the key point is to quickly eliminate septic diagnosis and add immunosuppressive treatment to empiric antibiotherapy.

### CO-42 Septic shock among patients with systemic lupus erythematosus- short and long-term outcome and associated costs. Analysis of a French nationwide database

#### Arthur Mageau (*speaker*)^1^, Karim Sacre^2^, Anne Perozziello^1^, Stéphane Ruckly^1^, ClaireDupuis^1^, Lila Bouadma^1^, Thomas Papo^3^, Jean-François Timsit^1^

##### ^1^IAME UMR 1137 Team 5 Descid, Paris, FRANCE; ^2^Hôpital Bichat, Service de Médecine Interne, Paris, FRANCE; ^3^Service de Médecine Interne, Hôpital Bichat, Paris, FRANCE

###### **Correspondence:** Arthur Mageau - arthur_mageau@hotmail.fr

*Annals of Intensive Care* 2019, **9(Suppl 1)**:CO-42

**Introduction**: We aimed to assess the characteristics, outcome and associated costs of septic shock complicating Systemic Lupus Erythematosus (SLE).

**Patients and methods**: Characteristics of SLE patients experiencing a septic shock in France from 2010 to 2015 were analyzed through the French nationwide medico-administrative database. The factors associated with the 1-year post-admission mortality were analyzed, the crude 1-year survival of SLE patients experiencing septic shock was compared to those admitted for another reason, and we compared the 1-year outcome and costs associated with SLE septic shock survival to a matched SLE ICU control population.

**Results**: Among 28.522 SLE patients, 1,068 experienced septic shock. Septic shock was severe (mean SAPSII score- 47.0 [± 21.6]) + 369 (34.6%) patients had invasive mechanical ventilation and 342 (32.0%) required renal replacement therapy. The 1-year mortality rate was 43.4% (n = 463). The hospital stay mean cost was € 25,327 [± 23,396]. Factors assocaited with 1-year mortality are on Table 1. Independently of the severity of comorbidities and acute illness, an associated Sjögren syndrome was the only specific SLE phenotype significantly associated with 1-year mortality (HR [CI95]- 1.392 [1.021–1.899]). Within one year, post-septic shock survivors (n = 738) were re-admitted 6.42 [17.3] times for 64.1 [48.9] days with a total cost of € 14,431 [20,444]. Unmatched analysis showed that the outcome of patients admitted in ICU for septic shock was poorer than that of patients admitted in ICU or hospital for another disease. Post-ICU 1-year hospital outcome and costs of septic shock survivors were not different from the other ICU survivors when matched on the severity of acute illness.

**Conclusion**: Associated Sjögren syndrome was the main determinant of the 1-year mortality among SLE patients experiencing septic shock. Although septic shock was a turning point in SLE history, survivors did not use more healthcare than survivors of another life-threatening disease.


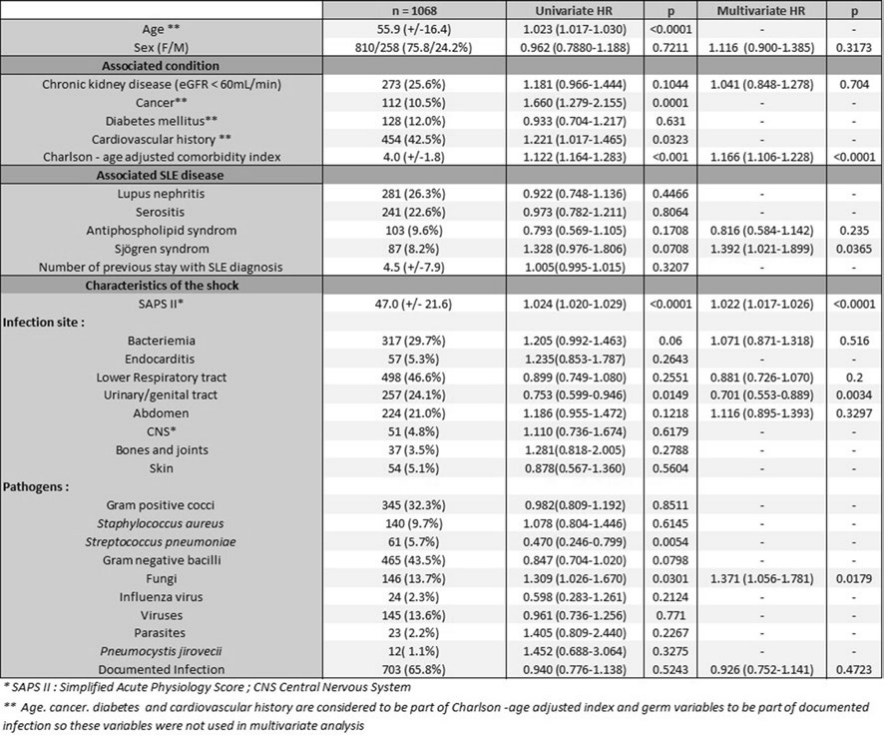


### CO-43 A 15 year retrospective audit of plasma exchange in the critically ill- Predictive factors of associated infections

#### Delphine Daubin (*speaker*)^1^, Florian Rissel^1^, Fanny Garnier^1^, Vincent Brunot^1^, Romaric Larcher^1^, Laura Platon^1^, Noemie Besnard^1^, Tarik Kanouni^2^, Pascal Latry^2^, Boris Jung^1^, Kada Klouche^1^

##### ^1^Intensive Care Unit Department, Montpellier, FRANCE; ^2^Hematology Department, Montpellier, FRANCE

###### **Correspondence:** Delphine Daubin - d-daubin@chu-montpellier.fr

*Annals of Intensive Care* 2019, **9(Suppl 1)**:CO-43

**Introduction**: Plasma exchange (PE) often leads to adverse effects. Sepsis resulting from impaired immunity caused by the removal of antibodies and related to catheter-associated infections represents the most severe complications. The study evaluate the infectious risk associated with the current use of PE and identify its predictive factors.

**Patients and methods**: All major patients admitted to our tertiary ICU and received PE therapy between 2002 and 2017 were included. Patients who developed secondary infection were compared to those who did not by the use of univariate and multivariate analysis.

**Results**: Eighty-eight patients underwent 616 PE procedures. 56% patients were in mechanical ventilation, 44% received constrictive drugs and 40% were in renal replacement therapy. Indications were Thrombotic Thrombocytopenic Purpura 48 (55%), Neurologic diseases 18 (20%), Vasculitis 14 (16%), autoimmune diseases 4 and other indications 4.

Adverse effects per-procedure were observed in 22% of patients in witch half of them were life-threathening. PE-associated infections occurred in 39 patients (44%). Median time elapsed from ICU admission and EP initiation to infection diagnosis were respectively 8 [5–14] and 4 [2–10] days. Infectious complications included 19 ventilation-associated pneumonia, 6 nosocomial pneumonia, 16 viral reactivations, 5 bacteremia, 4 catheter-associated infections, 4 urinary infections. Most of them were severe with a septic shock in 22 39 patients (58%) and 8 patients deceased (20%).

The comparison by univariate analysis showed that patients with associated infection had a significantly higher age, need more frequently a mechanical ventilation and vasoactive drugs. The number of PE and ICU and in-hospital length of stay were significantly higher in the infectious group. No significant differences were observed between infectious and no infectious groups regarding to severity score at admission, corticoids, immunosuppressive therapy, type of substitute liquid, PE volume. In multivariate analysis, age ([OR] = 1.05, CI95% 1.01–1.09, p = 0.02), number of session ([OR] = 1.22, CI 95% 1.08–1.39, p = 0.001) and requirement of mechanical ventilation ([OR] = 4.19, CI95% 1.27–13.81, p = 0.02) were predictive of the infection occurrence. ICU mortality was higher in the infectious group (28% versus 16% p = 0.28).

**Conclusion**: PE was associated with an infection, in particular pneumonia, in 44% of patients and lead to a life-threatening condition in 25% of them. Age, number of PE session and requirement of mechanical ventilation were predictive of the occurrence of infection.

### CO-44 A reappraisal of short and long-term outcome for patients with systemic rheumatic diseases admitted to intensive care unit and reassessment of prognostic factors- a multicenter retrospective study of 415 patients

#### Romaric Larcher (*speaker*)^1^, Marc Pineton de Chambrun^2^, Emma Rubenstein^1^, Laura Platon^1^, Kevin Chalard^1^, Delphine Daubin^1^, Noemie Besnard^1^, Philippe Corne^1^, Jonathan Charbit^1^, Charles-Edouard Luyt^2^, Zahir Amoura^2^, Samir Jaber^1^, Boris Jung^1^, Kada Klouche^1^

##### ^1^Montpellier University Hospital, Montpellier, FRANCE; ^2^La Pitie-Salpetriere Hospital, APHP, FRANCE

###### **Correspondence:** Romaric Larcher - r-larcher@chu-montpellier.fr

*Annals of Intensive Care* 2019, **9(Suppl 1)**:CO-44

**Introduction**: Recent advances in critical and rheumatic care may have impacted the outcome for patients with systemic rheumatic diseases (SRD) admitted to the intensive care unit (ICU). We aimed to reassess the short and long-term outcome and prognostic factors for SRD patients admitted to the ICU.

**Patients and methods**: We conducted a multicenter retrospective observational study in seven French ICU. All SRD patients admitted between 2006 and 2016 were included. In-hospital mortality and one-year survival after discharge were estimated through the Kaplan–Meier method and prognostic factors through Cox models. We compared survived and non-survived patients by univariate and multivariate analysis in order to identify prognostic factors of in-hospital mortality and one-year survival.

**Results**: In the 415 patients (female- 58%, mean age- 57 years) included, predominant SRD were vasculitides, rheumatoid arthritis and systemic lupus erythematosus. Main causes of admission were shock (39%) and acute respiratory failure (33%) and predominant diagnoses in ICU were infections (39%) and SRD flare-up (36%). At admission, mean SOFA score was 7 ± 4 and mean SAPS II was 46 ± 20. 276 patients (67%) required mechanical ventilation, 211 (51%) vasoactive drugs, 122 (29%) renal replacement therapy and 37 (9%) extracorporeal membrane oxygenation. In-hospital mortality rate was 29.4%, 95%CI [25–33.8] and one-year survival probability was 91.7%, 95%CI [88.3–95.2] (figure 1). Prognostic factors were age (HR 1.02, 95%CI [1.01–1.04], p = 0.002), previous corticosteroid treatment (HR 1.71, 95%CI [1.13–2.58], p = 0.01), SOFA score (HR 2.03, 95%CI [1.31–3.15], p = 0.002) and SAPS II (HR 1.63, 95%CI [1.09–2.45], p = 0.02) for in-hospital mortality. Besides age (HR 1.05, 95%CI [1.01–1.09], p = 0.007) no other variable was associated with one-year mortality after discharge.

**Conclusion**: Our study showed that most of two third of SRD patients admitted to the ICU survived at hospital discharge and a probability of one-year survival at 91.7% for these patients. This satisfactory survival rate may be related to the recent improvement in critical care and rheumatic care. We also found that SOFA score, SAPS II and previous corticosteroid treatment were predictive factors of in-hospital outcome.


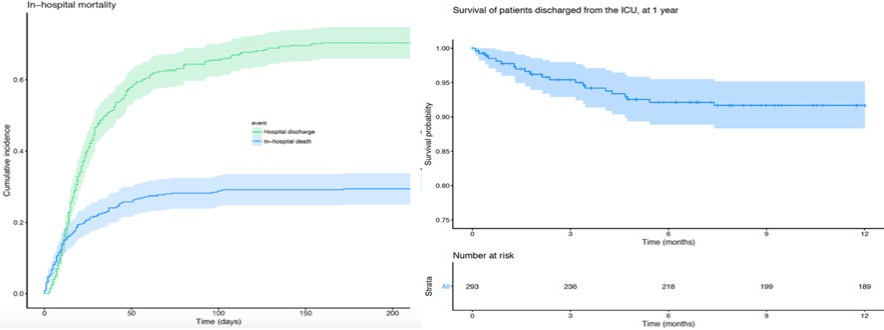


### CO-45 Chest CT scan and alveolar procollagen III to predict lung fibroproliferation in acute respiratory distress syndrome

#### Annabelle Hamon (*speaker*)^1^, Ugo Scemama^2^, Jeremy Bourenne^3^, Florence Daviet^2^, Benjamin Coiffard^2^, Nicolas Persico^2^, Mélanie Adda^2^, Christophe Guervilly^2^, Sami Hraiech^2^, Kathia Chaumoitre^2^, Antoine Roch^2^, Laurent Papazian^2^, Jean-Marie Forel^2^

##### ^1^Chiap, Marseille, FRANCE; ^2^Hôpital Nord AP-HM - CHU, Marseille, FRANCE; ^3^Hôpital Timone AP-HM - CHU, Marseille, FRANCE

###### **Correspondence:** Annabelle Hamon - annabelle.hamon@hotmail.fr

*Annals of Intensive Care* 2019, **9(Suppl 1)**:CO-45

**Introduction**: Lung fibroproliferation in ARDS patients is associated with mortality. Alveolar procollagen III (NT-PCP-III) is a biomarker of lung fibroproliferation. Chest CT scan could be useful for the diagnosis of fibroproliferative radiologic lesions. The main objective of the present study was therefore to quantify chest CT scan lesions in ARDS patients with a high alveolar level of NT-PCP-III indicating an active fibroproliferation.

**Patients and methods**: This retrospective cohort study over 6 years (January 2011-January 2017), included ARDS who had at least one dosage of alveolar NT-PCP-III obtained by performing a bronchoalveolar lavage (BAL) and a chest CT scan within 3 days before or after BAL. According to a previous study alveolar level of NT-PCP-III > 9µG L indicated a histological lung fibroproliferation (1). CT scan was scored on interstitial (honeycombing) and alveolar (ground-glass) abnormalities (2). Crude score and corrected score (related to the number of scored lobes in case of important lobar condensation) were used.

**Results**: One hundred ninety-two patients were included, either 228 “couples” alveolar NT-PCP-III and CT scan. Using a threshold of NT-PCP-III > 9µG L, crude and corrected fibrosis score was highest when lung fibroproliferation are present. Values of lung fibroproliferation CT scan score according to alveolar NT-PCP-III level appear in table 1. The median delay between the onset of ARDS and CT scan was of 5 [0–14] days, between the onset of ARDS and BAL 5 [0–14] days, between BAL and CT scan 0 [− 1–1] days.

**Conclusion**: When alveolar NT-PCP-III is used as a surrogate of lung fibroproliferation, CT scan fibrosis score is significantly higher in patient with active pulmonary fibroproliferation.

**References**

1. Forel J-M, Guervilly C, Hraiech S, Voillet F, Thomas G, Somma C, et al. Type III procollagen is a reliable marker of ARDS-associated lung fibroproliferation. Intensive Care Med. 1 janv 2015 + 41(1): 1-11.
2. Kazerooni EA, Martinez FJ, Flint A, Jamadar DA, Gross BH, Sp

### CO-46 Ultra-protective ventilation without extracorporeal circulation in moderately severe and severe ARDS patients

#### Jean-Christophe Richard (*speaker*)^1^, Sophie Marqué^2^, Antoine Gros^3^, Michel Muller^4^, Gwenaël Prat^5^, Gaëtan Beduneau^6^, Jean-Pierre Quenot ^7^, Jean Dellamonica^8^, Romain Tapponnier^9^, Edouard Soum^10^, Bitker Laurent^11^, Jack Richecoeur^12^

##### ^1^Hospices Civils de Lyon, FRANCE; ^2^Centre Hospitalier Sud-Francilien, Corbeil-Essonnes, FRANCE; ^3^Hôpital André Mignaud, Le Chesnay, FRANCE; ^4^Centre Hospitalier Annecy Genevois, Pringy, FRANCE; ^5^CHU de la Cavale Blanche, Brest, FRANCE; ^6^CHU Charles Nicolle, Rouen, FRANCE; ^7^Hôpital François Mitterrand, Dijon, FRANCE; ^8^CHU Nice- Hôpital Archet 1, Nice, FRANCE; ^9^Hospices Civils de Lyon- Centre Hospitalier Lyon Sud, Pierre-Benite, FRANCE; ^10^CHU Gabriel-Montpied, Clermont-Ferrand, FRANCE; ^11^Hospices Civils de Lyon- Hôpital de la Croix-Rousse, Lyon, FRANCE; ^12^Centre Hospitalier, Beauvais, FRANCE

###### **Correspondence:** Jean-Christophe Richard - j-christophe.richard@chu-lyon.fr

*Annals of Intensive Care* 2019, **9(Suppl 1)**:CO-46

**Introduction**: Ultraprotective ventilation with tidal volume (VT) reduction below 6 ml kg predicted body weight (PBW) in severe ARDS may reduce alveolar strain, driving pressure and hence ventilator-induced lung injury, with main drawback of worsening respiratory acidosis. We hypothesized that VT could be reduced up to 4 ml kg, with clinically significant decrease in driving pressure (DeltaP), without the need for extracorporeal CO2 removal, while maintaining pH in the range targeted in recent ARDS trials.

**Patients and methods**: We conducted a non-experimental before-and-after multi-center study on 35 ARDS patients with PaO2 FiO2 <= 150 mm Hg, within 24 h of ARDS diagnosis. After inclusion (H0), VT was reduced to 4 ml kg PBW and further adjusted to maintain pH > = 7.20, respiratory rate was increased up to 40 min and PEEP was set using a PEEP-FiO2 table favoring high PEEP. This strategy was applied until positivity of a PEEP weaning trial. The primary judgment criterion was DeltaP on day 2, as compared to study inclusion.

**Results**: Patients’ age was 62 ± 14 year, SAPS II amounted to 47 ± 15, 29 patients (85%) had pneumonia as ARDS risk factor, SOFA at inclusion was 13 ± 3, and PaO2 FiO2 at inclusion was 107 ± 35 mm Hg under a PEEP of 10 ± 4 cmH2O. From inclusion to day 2, DeltaP decreased significantly from 12.1 ± 4.3 to 8.6 ± 3.1 cmH2O, while VT decreased from 6.1 ± 0.6 to 4.4 ± 0.7 ml kg. On day 2 of the study (table 1), VT was below 4.2 ml kg in 22 patients (65% [IC95% 48%-79%]), and below 5.25 ml kg in 30 patients (88% CI95% [74%-95%]). Time with VT below 4.2 ml kg averaged 1.7 ± 1.4 days. Sedation drugs were not significantly modified. **Two patients (6%) developed acute cor pulmonale after inclusion. Right ventricle left ventricle ratio increased non-significantly from 0.59 ± 0.16 at H0 to 0.69 ± 0.22 on day 2. Eleven patients (32%) presented with severe respiratory acidosis (pH < 7.15). Fourteen patients (41%) died before day 90.

**Conclusion**: Ultraprotective ventilation may be applied in approximately 2 3 of moderately severe to severe ARDS patients, with a mean reduction of DeltaP approximating 3.5cmH2O, at the price of severe respiratory acidosis in 1 3 of the patients.

**Table:** Table 1

	H0	H2–H6	H8–H12	H14–H18	Day2	Day3
Driving pressure (cmH_2_O)	12.1 ± 4.3	9.6 ± 3.2†	9.2 ± 3.9†	9.0 ± 3.8†	8.6 ± 3.1†	9.0 ± 3.5†
VT (mL.kg^−1^ PPT)	6.1 ± 0.6	4.2 ± 0.5†	4.3 ± 0.5†	4.3 ± 0.7†	4.4 ± 0.7†	4.7 ± 1.1†
Respiratory rate (min^−1^)	27 ± 5	35 ± 5†	36 ± 5†	36 ± 5†	37 ± 5†	35 ± 6†
PEEP (cmH_2_O)	10 ± 4	15 ± 3†	15 ± 4†	15 ± 4†	14 ± 4†	13 ± 4†
pH	7.32 ± 0.10	7.23 ± 0.09†	7.25 ± 0.09†	7.28 ± 0.07	7.28 ± 0.08	7.33 ± 0.09
PaCO_2_ (mm Hg)	47 ± 11	62 ± 15†	59 ± 12†	56 ± 12†	55 ± 12†	55 ± 15†
PaO_2_/FiO_2_ (mm Hg)	107 ± 35	152 ± 61†	188 ± 69†	189 ± 54†	197 ± 50†	197 ± 53†
Midazolam (mg.h^−1^)	8 ± 7	–	–	–	6 ± 3	7 ± 3
Morphine (mg.h^−1^)	15 ± 12	–	–	–	15 ± 12	17 ± 15
Vasopressor (µg.kg.min^−1^)	0.57 ± 0.64	–	–	–	0.53 ± 0.75	0.29 ± 0.25†

†p < 0.05 vs H0.

### CO-47 Improved mortality associated with prone positioning during ECMO for ARDS: a retrospective study in 169 patients

#### Eloi Prud’Homme (*speaker*)^1^, Christophe Guervilly^1^, Vanessa Pauly^1^, Florence Daviet^1^, Jeremie Bourenne^2^, Benjamin Coiffard^1^, Jean-Marie Forel^1^, Sami Hraiech^1^, Melanie Adda^1^, Antoine Roch^3^, Nicolas Persico^3^, Laurent Papazian^1^

##### ^1^Medical Intensive Care Unit, North Hospital, APHM, Marseille, FRANCE; ^2^Medical and Emergency Resuscitation, la Timone 2 University Hospital, Aix-Marseille University, Marseille, FRANCE; ^3^Emergency Department, Hôpital Nord, APHM, Marseille, FRANCE

###### **Correspondence:** Eloi Prud’Homme - eloiprudhomme@gmail.com

*Annals of Intensive Care* 2019, **9(Suppl 1)**:CO-47

**Introduction**: Early, prolonged and repeated sessions of prone positioning (PP) have proven substantial outcome benefit in ARDS patients. In the most severe forms of ARDS, veno venous extracorporeal membrane oxygenation (vvECMO) is considered as a therapeutic option performed on a case by case. About 50% of patients treated with vvECMO have previously been turned prone. However, only few studies have evaluated the interest of maintaining PP during ECMO. Therefore, the objective of this study was to compare the 90-day survival rate of patients supported by vvECMO with concomitant PP (prone ECMO group) to those not turned prone during the ECMO period (ECMO alone group).

**Patients and methods**: We included all adults treated with vvECMO for ARDS between January 2012 and April 2017 in a university teaching hospital in Marseille, France. Patients were either cannulated in our ICU or in a referring hospital then transported by the ECMO mobile team. Before considering patients for ECMO, a PP session was systematically performed in our center if feasible, and strongly encouraged for patients from referral centers. Criteria for vvECMO have been previously published. During ECMO, patients were considered for prone position in case of 1 persistent hypoxemia defined by SpO2 < 88% or PaO2 < 55 mmHg despite a 100% FdO2 and FiO2 with a maximal ECMO blood flow, 2 failure of attempt to wean ECMO after at least 10 days of ECMO and the presence of lung consolidations on chest X-ray or ultrasounds echography, 3 according to the physician in charge. Technical issues have been previously described. We compared the prone ECMO group to the ECMO alone group. We performed Kaplan–Meier 90-day survival curves and Coxmodel for 90-day mortality.

**Results**: 169 patients were included in the analysis, 92 in the prone group and 77 in the ECMO alone group. The prone group has a 57.6% 90-day survival rate as compared with a 37.7% 90-day survival rate of the ECMO alone group, p = 0.01. SAPS2 [HR = 1.040 (95% CI 1.024–1.056) and use of iNO before ECMO [HR = 1.86 (95% CI 1.156–2.992)] were independently associated with increase in 90-day mortality whereas the use of PP during ECMO was independently associated with decrease in 90-day mortality [HR = 0.439 (95% CI 0.270–0.714)].

**Conclusion**: The continuation of PP during vvECMO could be of clinical interest. Further prospective controlled study is warranted.

### CO-48 Mechanical ventilation management on ECMO for ARDS An International Multicenter Prospective Cohort - The ventiLatIon management oF patients with Extracorporeal membrane oxyGenation for Acute Respiratory Distress Syndrome - LIFEGARDS study

#### Matthieu Schmidt (*speaker*)^1^, Tai Pham^2^, Christophe Guervilly^3^, Mathilde Neuville^4^, Elie Zogheib^5^, Jean-Paul Mira^6^, Hadrien Rozé ^7^, Marc Pierrot^8^, Daniel Brodie^9^, Alain Combes^10^

##### ^1^Hopital Pitié Salpetrière Medical ICU, Paris, FRANCE; ^2^Interdepartmental Division of Critical Care Medicine, University of Toronto, CANADA; ^3^CHU Hopital Nord-Service de Medecine Intensive et Reanimation, Marseille, FRANCE; ^4^AP-HP, Bichat Hospital, Medical and Infectious Diseases Intensive Care Unit, Paris, FRANCE; ^5^Cardiothoracic and Vascular Intensive Care Unit, Amiens, FRANCE; ^6^Cardiothoracic and Vascular Intensive Care Unit, APHP, Groupe Hospitalier Universitaire de Paris Centre, Paris, FRANCE; ^7^South Department of Anesthesiology and Critical Care, Pessac, FRANCE; ^8^Service de Réanimation Médicale, Angers, FRANCE; ^9^Department of Medicine, New York, UNITED STATES; ^10^Service de Réanimation Médicale, Paris, FRANCE

###### **Correspondence:** Matthieu Schmidt - matthieuschmidt@yahoo.fr

*Annals of Intensive Care* 2019, **9(Suppl 1)**:CO-48

**Introduction**: The objectives were to report current practices regarding mechanical ventilation and use of adjunct therapies in patients treated with extracorporeal membrane oxygenation (ECMO) for severe acute respiratory distress syndrome (ARDS) and to describe major ECMO-related complications and 6-month outcomes.

**Patients and methods**: International, multi-center, prospective cohort study of patients undergoing ECMO for ARDS during a one-year period in a convenience sample of 23 international intensive care units (ICUs).

**Results**: We collected demographics, daily pre- and per-ECMO mechanical ventilation settings and use of adjunctive therapies, ICU- and 6-month–outcome data for 350 patients (median ± standard deviation age 47 ± 17 years, pre-ECMO PaO2 FiO2 71 ± 34 mmHg). Pre-cannulation use of prone positioning and neuromuscular blockers were 26% and 62%, respectively. Tidal volume, plateau pressure, and minute ventilation were significantly reduced during ECMO. The driving pressure was significantly reduced from 18.6 ± 8.3 to 11.6 ± 5.3 cm H2O. Median ECMO duration and ICU stay were respectively 10 and 24 days and six-month survival was 61%. Multivariable analyses retained immunodeficiency, older age, extra-pulmonary sepsis, lower pre-ECMO pH, higher pre-ECMO respiratory rate, greater delay from intubation to ECMO start, and higher mean fluid balance in the first ECMO days as being independently associated with 6-month mortality.

**Conclusion**: Pre-ECMO management of patients with severe forms of ARDS in high case-volume centers was notable for low use of prone positioning, suggesting the potential for improvement in pre-ECMO ARDS management that might impact the number of cases considered for ECMO. Once receiving ECMO, the use of least damaging lung ventilation strategies was largely adopted.

### CO-49 Extracorporeal vs. Conventional Cardiopulmonary Resuscitation Comparison- A matched pairs retrospective study

#### Daniel Patricio (*speaker*)^1^, Lorenzo Peluso^1^, Alexandre Brasseur^1^, Olivier Lheureux^1^, Mirko Belliato^2^, Jean-Louis Vincent^1^, Creteur Jacques^1^, Fabio Silvio Taccone^1^

##### ^1^Hôpital Erasme, Université Libre de Bruxelles, Bruxelles, BELGIUM; ^2^Policlinico San Matteo, Pavia, Italy, Pavia, ITALY

###### **Correspondence:** Daniel Patricio - patriciodan@ymail.com

*Annals of Intensive Care* 2019, **9(Suppl 1)**:CO-49

**Introduction**: The potential benefit of extracorporeal cardiopulmonary resuscitation (ECPR) for patients with refractory cardiac arrest (CA) remains unsettled.

**Patients and methods**: Retrospective analysis of a database from our prospective observational cohort of CA patients including all consecutive adult patients referred to the Department of Intensive Care with CA between January 2012 and December 2017. The decision to initiate ECPR was dependent on the attending physician and initiated by the ECPR team, which is composed of ICU physicians. A propensity score was derived using a logistic regression model, including characteristics that varied between groups with a p < 0.10 and others that potentially related to outcome. The primary outcomes were survival to ICU discharge and 3-month favorable neurologic outcome, assessed by the Cerebral Performance Categories (CPC) of 1–2.

**Results**: On a total of 635 CA patients admitted over the study period (ECPR, n = 112), 80 patients with ECPR were matched to 80 patients who underwent CCPR (median age- 57 years—out-of-hospital CA, 62%). Time from arrest to termination of CPR (i.e. return of spontaneous circulation—ROSC, ECMO initiation or death) was 54 ± 22 and 54 ± 19 min in the ECPR and CCPR groups, respectively. ROSC rates were 77 80 (96%) and 30 (38%) in the two groups (p < 0.001). Survival at ICU discharge was 18 80 (23%) vs. 14 80 (18% + p = 0.46), respectively, and 3-month favorable outcome (17 80, (21%) and 9 80, 11%), receptively (p = 0.13). Kaplan–Meier survival analysis showed a better neurological outcome in the matched ECPR group than the CCPR group (log-rank p = 0.01).

**Conclusion**: ECPR after cardiac arrest can be associated with an improved long-term neurological outcome.

### CO-50 Unexpected cardiac arrest occurring in the ICU- preliminary results of a French multicenter study (ACIR)

#### Maxime Leloup (*speaker*), Isabelle Briatte, Alice Langlois, Alexandre Herbland, Olivier Lesieur, Study Group Acir

##### Hôpital Saint Louis, La Rochelle, FRANCE

###### **Correspondence:** Maxime Leloup - leloup.mx@gmail.com

*Annals of Intensive Care* 2019, **9(Suppl 1)**:CO-50

**Introduction**: To our knowledge, no study mapped the epidemiology of in-ICU cardiac arrest (CA) in France. Our survey aims to describe demographics, management, vital and neurological prognosis of patients concerned.

**Patients and methods**: “ACIR” (in French- Arrêt Cardiaque Inattendu en Réanimation) is a prospective observational study implemented in 45 French ICUs throughout 2017. All victims of unexpected CA with resuscitation attempt (chest compression, adrenaline and or electric shock) once admitted to the ICU were included in the “in-ICU CA” chart. The data collected comprises medical history, circumstances and ongoing treatments at the time of the event, resuscitation maneuvers that were performed, survival rate and Cerebral Performance Category (CPC) score.

**Results**: Of the approximately 30000 patients admitted over the study period, 677 (69% men, 68 ± 13 years, SAPS2 71 ± 25) endured an unexpected CA. Among them, 36% had been hospitalized for shock, 25% for respiratory distress and 15% for out-of-ICU CA. One quarter had 3 or more organ failures. Half of the CA occurred within the first 24 h from admission and 58% during procedures deemed at risk (mainly endotracheal intubation 15%, dialysis 9%, nursing care 6%, and intra-hospital transport 4%). A recognizable etiology was identified in 79% of CA- 18% were attributed to current life-sustaining interventions (mechanical ventilation 7%, circulatory assistance 7%, drug-related complications 4%), and 82% to life-threatening conditions (mainly hypoxemia 33%, metabolic disorder 23% and hypovolemia 18%).

The cardio pulmonary resuscitation was started without delay. A shockable rhythm (ventricular tachycardia or fibrillation) was diagnosed in 18% of cases, with the first shock delivered within 2 [0–3] minutes. In cases of non-shockable rhythm (asystole, pulseless bradycardia), the first adrenaline injection was given at 1 [0–2] minute. Return of spontaneous circulation (ROSC) was achieved in 477 677 (70%) patients, of whom 146 677 (21.6%) were discharged alive from hospital. The number of CA survivors with a CPC score of 1 or 2 (considered satisfactory) was 135 146 (92%) at hospital discharge and 117 128 (91%) 6 months after CA.

**Conclusion**: In-ICU CA occurred in severely ill patients, during the first few days of hospitalization and more than half of cases during at-risk procedures. One-fifth was attributed to the life-sustaining intervention(s) in place. Despite a high rate of ROSC (70%), only 21.6% of victims were discharged alive from the hospital. However, survivors maintained a good neurological status over time.

### CO-51 Temporal trends in TTM use after cardiac arrest and association with outcome in a large registry

#### Jean-Baptiste Lascarrou (*speaker*)^1^, Florence Dumas^2^, Richard Chocron^2^, Wulfran Bougouin^3^, Stephane Legriel^3^, Nadia Aissaoui^3^, Nicolas Deye^3^, Eloi Marijon^3^, Xavier Jouven^3^, Alain Cariou^3^

##### ^1^Service de Médecine Intensive Réanimation, Nantes, FRANCE; ^2^Emergency department, Cochin Hotel Dieu Hospital, APHP, Paris, FRANCE; ^3^Paris Sudden Death Expertise Center, Paris, FRANCE

###### **Correspondence:** Jean-Baptiste Lascarrou - jeanbaptiste.lascarrou@chu-nantes.fr

*Annals of Intensive Care* 2019, **9(Suppl 1)**:CO-51

**Introduction**: Emphasis regarding the importance of early cardiopulmonary resuscitation (CPR) and defibrillation led to an improvement in outcome after cardiac arrest (CA). Meanwhile, recent data raised some concerns regarding the benefit of targeted temperature management (TTM) at 33 °C for comatose cardiac arrest survivors. This may have resulted in a loss of interest in TTM in these patients, with uncertain consequences on outcome. We use data from the Sudden Death Expertise Center (SDEC) of the Great Paris area to assess the relationship between changes over time in the use of TTM and patients’ outcome after adjustment for potential confounders.

**Patients and methods**: We used data prospectively collected in SDEC registry between May 2011 and December 2016. Variables were collected according to Utstein style. All non-traumatic OHCA patients with stable return of spontaneous circulation (ROSC) were included in the analysis. We compared patients’ characteristics and outcome according to TTM use. We performed a multivariable logistic regression using survival at ICU discharge as the main endpoint.

**Results**: During study period, 3667 patients were retained in the analysis, of whom 1794 received TTM. As compared with controls, patients with TTM were significantly more frequently male and younger + CA occurred more frequently in a public place + a shockable rhythm was more frequent and the proportion of patients with a short no-flow (< 3 min) was greater (all P < 0.001). Regarding time trends, gender, age and location of CA did not change according to the year of inclusion in the registry. Bystander CPR increased from 56% in 2011 to 82% of patients in 2016 (P < 0.001), the rate of shockable rhythms increased from 40% to 51% (P = 0.005). The rate of patients with a no-flow > 3 min decreased from 58% to 43% (P < 0.001) (Figure 1). TTM use decreased over time, from 55% in 2011 to 48% in 2016 (P < 0.001). Meanwhile, survival rate at ICU discharge increased significantly from 20% to 28% of patients (P = 0.018) (Figure). In multivariate analysis, year of CA occurrence was associated with outcome at ICU discharge. The TTM use decrease over years was not driven by any particular Utstein criteria.

**Conclusion**: We report an improvement over time in ICU survival of post-CA patients, which appears mostly associated with an increase in bystander CPR and higher rate of shockable rhythms. TTM use declined over years in all subgroups of CA patients with no evident consequences regarding outcome.


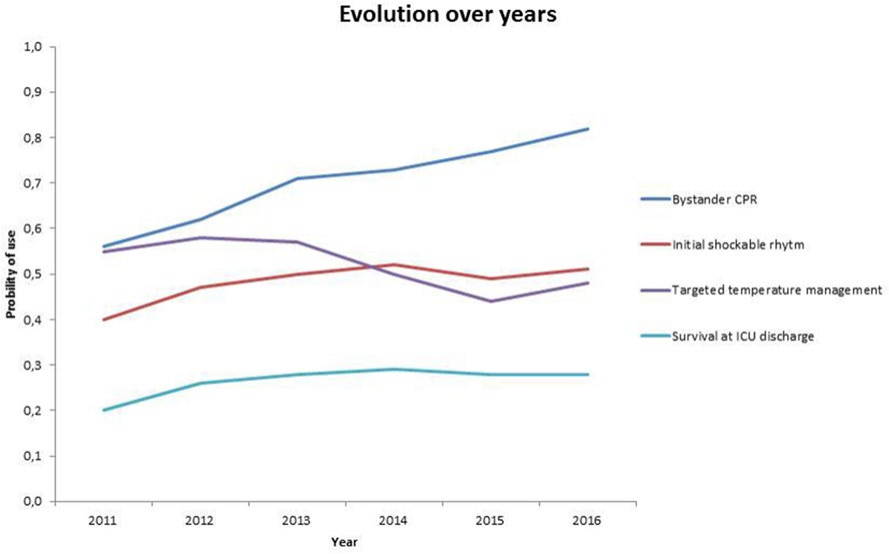


### CO-52 Incidence and outcome of mesenteric ischemia after cardiac arrest

#### Marine Paul (*speaker*)^1^, Paul Jaubert^1^, Guillaume Savary^1^, Stephane Andre^1^, Pierre Jaquet^1^, Arnaud Roccabianca^1^, Jean François Llitjos^1^, Sarah Benghanem^1^, Wulfran Bougouin^2^, Frederic Pene^1^, Jean Paul Mira^1^, Florence Dumas^3^, Alain Cariou^1^

##### ^1^Medical icu cochin hospital, Paris, FRANCE; ^2^INSERM U970 (team 4), Paris, FRANCE; ^3^Emergency department cochin hospital, Paris, FRANCE

###### **Correspondence:** Marine Paul - marine.1604@hotmail.fr

*Annals of Intensive Care* 2019, **9(Suppl 1)**:CO-52

**Introduction**: Post-resuscitation shock has a high incidence after cardiac arrest (CA) due to the whole-body ischemia–reperfusion and may be complicated by multiple organ failure (MOF) and death. Mesenteric ischemia (MI) may occur in this setting but incidence and outcome are poorly documented.

**Patients and methods**: We performed a retrospective monocentric study aiming to describe characteristics and outcome of MI after CA. All consecutive patients admitted in a tertiary CA center between 2007 and 2017 with a confirmed MI during their ICU stay were studied. MI was confirmed by endoscopy, abdominal CT-scan or surgical laparotomy.

**Results**: Among 1503 CA post-CA patients, MI was clinically suspected in 59 patients (4%) and confirmed in 28 59 patients. Median age was 62 years, median time from CA to cardio-pulmonary resuscitation (CPR) was 3 min (0–5), and time from CPR to return of spontaneous circulation was 22 min (18.3–29.5). Patients had an initial shockable rhythm in 57%, they received epinephrine during CPR in 82% and 93% of patients developed a post-resuscitation shock. Median initial blood lactate was 8.5 mmol/L. Delay between CA and the diagnosis of MI was 2 days (2–4), mostly because of clinical abdominal symptoms (79%) including hemorrhage and feeding intolerance, confirmed with CT scan abnormalities (54%) and lower digestive endoscopy (39%). Despite surgical intestinal resection performed in 7 28 patients, ICU mortality was 96%, as only 1 patient survived with good neurologic outcome. Cause of death was the MOF itself (41%) or post-anoxic brain injury for 37% despite a recovery after the initial shock.

**Conclusion**: In this cohort, we observed that MI after CA is an uncommon complication associated with a very poor outcome. Efforts should be made in order to obtain an earlier recognition of MI in patients with preserved brain function. These preliminary results need to be confirmed in a multicentric study.

### CO-53 BOTUREA STUDY - A descriptive retrospective multicenter study of severe adult cases of botulism between 2000 and 2017 in French intensive care units

#### Nathalie Courtois (*speaker*)^1^, Laurent Argaud^2^, Pierre Asfar^3^, Paul Mercury^4^, Juliette Pocquet^5^, Hervé Dupont^6^, Julien Charpentier ^7^, Djillali Annane^8^, Cédric Bretonniere^9^, Jean-Christophe Callahan^1^, Mickael Landais^1^, Christophe Guitton^1^

##### ^1^Réanimation Médico-Chirurgicale et USC, Le Mans, FRANCE; ^2^Service de Réanimation Médicale, Lyon, FRANCE; ^3^CHU Angers-Service de Médecine Intensive- Réanimation et Médecin Hyperbare, Angers, FRANCE; ^4^Service de Réanimation Médico-Chirurgicale et USC, Bastia, FRANCE; ^5^Service de Médecine Intensive - Réanimation, Tours, FRANCE; ^6^Service de Réanimation Polyvalente, Amiens, FRANCE; ^7^Service de Médecine Intensive - Réanimation Cochin, Paris, FRANCE; ^8^Service de Médecine Intensive - Réanimation Garches, Paris, FRANCE; ^9^Service de Médecine Intensive - Réanimation, Nantes, FRANCE

###### **Correspondence:** Nathalie Courtois - courtoisnathalie@gmail.com

*Annals of Intensive Care* 2019, **9(Suppl 1)**:CO-53

**Introduction**: Botulism is a rare potentially severe neuroparalytic disease caused by neurotoxins produced in most cases by Clostridium botulinum. Treatment mainly consists in life support techniques, and some specific medications such as antitoxins. In France, there are no guidelines regarding the use of these antitoxins, and they are only available with a Temporary Authorization for Use. The primary goal of this study was to describe and evaluate morbidity and mortality of severe adult botulism in ICUs in France. Our secondary goal was the evaluation of current patient care in French ICUs, especially concerning administration of antitoxins.

**Patients and methods**: We conducted a retrospective observational multicenter study in French ICUs. Among 197 adult ICUs, 93 units agreed to participate. Patients’ data were collected through a standardised CRF. We included patients aged above 15 years of age who were admitted in an ICU between January 2000 and June 2017 for an acute botulism. Study protocol was approved by the SRLF ethics committee. Data collection was conducted according French law and CNIL recommendations.

**Results**: Among the participating units, 59 didn’t identify any botulism cases and 6 were unable to collect patient’s data. Twenty-eight ICUs identified 52 patients. At admission, median (IQR) age was 49.2 years (37–65) and 51% of patients were male. 94.1% of the patients had foodborne botulism. Median SAPS II was 21 (17–26). For clinical presentation, ocular symptoms (especially diplopia 84% and ptosis 67%) and oro-pharyngeal symptoms (especially swallowing impairment 78%) were the most frequent, and were usually the first to be described. 65% of patients were intubated. Median duration of mechanical ventilation was 13 days (0–53) with a maximum of 333 days. Tracheotomy was performed for 36% of patients. Median ICU hospitalisation duration was 27 days (8–47). The current botulism episode was associated with sequelae evaluated by the modified Rankin Scale. At ICU discharge, hospital discharge and last follow-up, 43, 22 and 10% of patients respectively had a score above 3. Two patients died during their hospitalisation (1 during ICU stay). Twenty-five patients (48.1%) were treated with botulism antitoxin. We were unable to show any significant difference in outcomes between patients treated or not treated with antitoxin.

**Conclusion**: Although botulism rarely causes death nowadays, it is a potentially severe disease, with a high morbidity and possible extended hospitalisation and mechanical ventilation. Recent studies are in favour of the use of antitoxins, and clear recommendations are needed.

### CO-54 First-in patients TREM-1 Pathway Inhibitory Peptide in Septic Shock- The MOT-C-201 Clinical Trial Results

#### Bruno Francois (*speaker*)^1^, Xavier Wittebole^2^, Ricard Ferrer^3^, Jean-Paul Mira^4^, Thierry Dugernier^5^, Sébastien Gibot^6^, Marc Derive ^7^, Peter Pickkers^8^, Jean-Jacques Garaud^7^, Miguel Sanchez^9^, Margarita Salcedo-Magguilli^7^, Pierre-François Laterre^2^

##### ^1^Medical-Surgical ICU department and Inserm CIC1435, CHU, Limoges, FRANCE; ^2^Department of Critical Care Medicine, St Luc University Hospital, Université Catholique de Louvain, Brussels, BELGIUM; ^3^ICU department, Vall d’Hebron University Hospital, Barcelona, SPAIN; ^4^Medical ICU, Cochin Hotel-Dieu, AP-HP, Paris, FRANCE; ^5^ICU department, Clinique St. Pierre, Ottignies-Louvain-La-Neuve, BELGIUM; ^6^Medical ICU department, Hospital Central, CHU, Inserm U1116, Nancy Medical Faculty, Nancy, FRANCE; ^7^Inotrem SA, Paris, FRANCE; ^8^ICU department, Radboudumc Hospital, Nijmegen, THE NETHERLANDS; ^9^ICU department, Hospital Clínico San Carlos, Madrid, Spain, Madrid, SPAIN

###### **Correspondence:** Bruno Francois - bruno.francois@chu-limoges.fr

*Annals of Intensive Care* 2019, **9(Suppl 1)**:CO-54

**Introduction**: Nangibotide peptide is a specific TREM-1 inhibitor. In preclinical septic shock models, nangibotide was able to restore appropriate inflammatory response, vascular function, and improved survival. In phase I, nangibotide was found to be safe and well tolerated up to the highest dose.

**Patients and methods**: International, multi-center phase IIa randomized, double-blind, two-stage, placebo-controlled study (NCT03158948). Main inclusion criteria were septic shock according to Sepsis 3 definition and nangibotide to be initiated within 24 h of shock onset. Patients were randomized to receive either placebo, 0.3, 1 or 3 mg kg h of nangibotide. Study drug was infused until end of vasopressors + 12 h or up to 5 days. Safety data were reviewed by an independent Data Safety Monitoring Board (DSMB). Primary endpoint was safety and tolerability. Patient follow-up period was 90 days.

**Results**: 50 patients were randomized and 49 treated (1 patient died before dosing). All groups were well balanced in terms of baseline characteristics, except for APACHE II score which tend to be non-significantly lower in placebo group. Primary infection source was 40% abdominal, 50% pulmonary and 10% urinary.

Nangibotide was safe and well tolerated in all groups. The DSMB did not raise any safety concern. Number of SAEs AEs and number of patients with SAEs AEs was comparable between all groups. Most frequent AEs were atrial fibrillation, anemia, pleural effusion and thrombocytopenia.

A trend toward a decrease in circulating levels of pharmacodynamic markers was observed in nangibotide-treated patients. All-cause mortality at day-28 was 14% (5/37) in pooled nangibotide groups and 25% (3/12) in placebo group. In the subgroup with sTREM-1 levels above median, the day-5 mortality was calculated as 40% (2/5) and 20% (4/20) in placebo and nangibotide groups respectively. In this group with high sTREM-1 levels, a trend towards an increase in organ support free days alive was seen for nangibotide-treated patients versus placebo.

**Conclusion**: Nangibotide was shown to be safe and well tolerated in septic shock patients. Although this small exploratory study was not powered to conclude on efficacy, a non significant lower mortality was observed in the nangibotide group. These results support the need of a larger study to demonstrate the role of nangibotide in the treatment of septic shock and the exploration of sTREM-1 as potential efficacy predictive biomarker for nangibotide.

### CO-55 Anxiety and depression in patients admitted in ICU for more than 48 h- results of the multicentre, observational, prospective MOOD-ICU study

#### Camille Vissac (*speaker*)^1^, Saad Nseir^2^, Françis Schneider^3^, Jean-Francois Timsit^4^, Alexandre Boyer^5^, Jean-Paul Mira^6^, Vincent Peigne ^7^, Carole Schwebel^8^, Kada Klouche^9^, Ferhat Meziani^10^, Walter Picard^11^, Emmanuelle Mercier^12^, Emmanuel Pontis^13^, Charles-Edouard Luyt^14^, Daniel Silva^15^, Aurélie Le Thuaut^16^, Jean Reignier^17^, Group Mood-Icu^18^

##### ^1^Médecine Intensive-Réanimation, Nantes, FRANCE; ^2^Médecine Intensive Réanimation, Lille, FRANCE; ^3^Chu-Médecine Intensive Réanimation, Strasbourg, FRANCE; ^4^Médecine Intensive Réanimation, Paris, FRANCE; ^5^Médecine Intensive Réanimation, Bordeaux, FRANCE; ^6^Médecine Intensive Réanimation, Paris, FRANCE; ^7^Médecine Intensive Réanimation, Chambéry, Chambéry; ^8^Médecine Intensive Réanimation, La Tronche, FRANCE; ^9^Médecine Intensive Réanimation, Montpellier, FRANCE; ^10^Médecine Intensive-Réanimation, Strasbourg, FRANCE; ^11^Médecine Intensive-Réanimation, Pau, FRANCE; ^12^Médecine Intensive-Réanimation, Tours, FRANCE; ^13^Médecine Intensive-Réanimation, Rennes, FRANCE; ^14^Médecine Intensive-Réanimation, Paris, FRANCE; ^15^Médecine Intensive-Réanimation, Saint-Denis, FRANCE; ^16^Maison de la recherche en santé, Nantes, FRANCE; ^17^CHU, Nantes, FRANCE; ^18^Médecine Intensive-réanimation, Nantes, FRANCE

###### **Correspondence:** Camille Vissac - vissac.camille@live.fr

*Annals of Intensive Care* 2019, **9(Suppl 1)**:CO-55

**Introduction**: Several studies have been published on psychiatric disorders after intensive are unit (ICU) and risk factors associated with these disorders. No studie have evaluated the frequency of anxiety and depression disorders during the ICU stay.

**Patients and methods**: The main objective of this prospective, multicentre, observational study was to assess rates of patients with anxiety and or depression in ICU. The secondary objectives were to assess rates of patients with anxiolytic and antidepressant drugs and to identify factors associated with anxiety and depression in ICU. All patients hospitalized since more than 48 h in ICU at study day were eligible. The evaluation of patients anxiety and depression symptoms was performed by the bedside physician and the nurse caring for the patient at the day of the study.

**Results**: 81 French ICU participated to the study, and 799 patients were included. 389 patients were reported as having anxiety and or depression by caregivers (51.0%, 95% CI [47.3–54.6]). 21.2% and 5.1% of the patients included in the study received drugs to treat anxiety and depression, respectively. Risk factors independently associated with anxiety and or depression were- a history of anxiety or depressive syndrome (OR 1.75, 95% CI [1.19–2.57], p = 0.004), announcement of severe illness and or loss of autonomy during ICU stay (OR = 2.12, 95% CI [1.32–3.43], p = 0.002), having tracheostomy performed during ICU stay (OR = 2.04, 95% CI [1.08–3.85], p = 0.03), administration of antipsychotic medication in ICU (OR = 1.94, 95% CI [1.20–3.14], p = 0.003), and reading of a book by cargivers or relatives during the ICU stay (OR = 2.01, 95% CI [1.08–3.74], p = 0.03). Organ failure was independently associated with less anxiety and or depression (OR = 0.57, 95% CI [0.33–0.99], p = 0.03).

**Conclusion**: Anxiety and depression symptoms were common in critically ill patients during their ICU stay. History of anxiety or depression, announcement of severe illness and or loss of autonomy, tracheostomy, antipsychotic medication and reading of book by caregivers were associated with increased risks of anxiety and depression disorders in ICU. However, severity of critical illness was associated with decreased risk of anxiety and depression. Future studies are required to assess the relationship between anxiety and depression disorders during and after ICU stay.

### CO-56 Assessment of care-givers’ experience of end of life in the ICU

#### Florence Boissier (*speaker*)^1^, Valérie Seegers^2^, Stéphane Legriel^3^, Alain Cariou^4^, Samir Jaber^5^, Jean-Yves Lefrant^6^, Bernard Floccard ^7^, Anne Renault^8^, Isabelle Vinatier^9^, Armelle Mathonnet^10^, Danielle Reuter^11^, Olivier Guisset^12^, Christophe Cracco^13^, Amélie Seguin^14^, Jacques Durand-Gasselin^15^, Beatrice Eon^16^, Marina Thirion^17^, Jean-Philippe Rigaud^18^, Bénédicte Philippon-Jouve^19^, Laurent Argaud^7^, Renaud Chouquer^20^, Mélanie Adda^21^, Laurent Papazian^21^

##### ^1^Réanimation médicale, CHU, Poitiers, FRANCE; ^2^Departement of Clinical Research, Integrated Center of Oncology, Angers, FRANCE; ^3^Service de réanimation, CH de Versailles André Mignot, Le Chesnay, FRANCE; ^4^Service de réanimation médicale, hôpital Cochin APHP, Paris, FRANCE; ^5^Service de réanimation, CHU Saint Eloi, Montpellier, FRANCE; ^6^Service de réanimation chirurgicale, CHU Carémeau, Nîmes, FRANCE; ^7^Service d’anesthésie réanimation, Hospices civils de Lyon Edouard Herriot, Lyon, FRANCE; ^8^Service de réanimation, CHU Cavale Blanche, Brest, FRANCE; ^9^Service de réanimation, CH Vendée, La Roche-Sur-Yon, FRANCE; ^10^Service de réanimation, hôpital de la Source, Orléans, FRANCE; ^11^Groupe de recherche FAMIREA, Hôpital Saint Louis APHP, Paris, FRANCE; ^12^Service de réanimation, CHU de Bordeaux Saint André, Bordeaux, FRANCE; ^13^Service de réanimation, CH, Angoulême, FRANCE; ^14^Service de réanimation, CHU, Caen, FRANCE; ^15^Service de réanimation, Hopital Sainte Musse, Toulon, FRANCE; ^16^Service de réanimation, Hopital La Timone APHM, Marseille, FRANCE; ^17^Service de réanimation, CH, Argenteuil, FRANCE; ^18^Service de réanimation, CH, Dieppe, FRANCE; ^19^Service de réanimation, CH, Roanne, FRANCE; ^20^Service de réanimation, CH Annecy, Annecy, FRANCE; ^21^Service de réanimation, Hopital Nord APHM, Marseille, FRANCE

###### **Correspondence:** Florence Boissier - floboissier@yahoo.fr

*Annals of Intensive Care* 2019, **9(Suppl 1)**:CO-56

**Introduction**: An increasing number of deaths occur in the ICU. In this setting, research has focused on describing, understanding, and improving end-of-life care as well as improving the patients and the families’ experience. The aim of this study was to describe and evaluate the experience of physicians and nurses involved in end-of-life situations in the ICU and describe the factors associated with a poor or good score.

**Patients and methods**: A 15-item questionnaire was validated in a multicenter prospective study conducted from July 2011 to July 2013 in 41 French ICUs. In each ICU, consecutive adults who died after at least 48 h after admission in the ICU were included. The physician and the nurse in charge of the patient at the time of death were asked to complete the questionnaire within 24 h after the death. The psychometric validation was conducted using two datasets- a learning cohort and a validation cohort.

**Results**: Among 475 patients, 398 nurse scores and 417 physician scores were analyzed. The median global CAESAR score for nurses was 62 75 and 64 75 for physicians. Lower nurse scores were significantly associated with the following factors- conflict with physicians, pain managed by nurses alone rather than with physicians, death disclosed to the family by phone or upon arrival rather than at the bedside and use of invasive treatment at the end of life. Higher physician scores were significantly associated with the following factors- availability of a dedicated information room, joint family information with the nurse, total rather than partial family information, joint implementation with the nurse of the decision to withdrawing withholding therapies, open visiting policy. Higher scores were also associated with patients’ characteristics- McCabe ≤ 1, not immunocompromised, no dementia, no liver failure, no haematological disease and no use of psychotropic medication. Last, higher score were associated with End Of Life conditions- decision to withdrawing withholding therapies, no cardiopulmonary resuscitation, death disclosed to the family at the bedside rather than by phone or upon arrival at the ICU.

**Conclusion**: We described and validated a new instrument for assessing the experience of physicians and nurses involved in end-of-life situations in the ICU. This study shows important areas for improving practices, including adapted level of intensive care, quality teamwork, quality communication and implication of family members.

## Flash Communications

### F-01 Efficiency of a combined geriatric and intensivist evaluation to identify clusters of elderly patients admitted to the intensive care unit - the SENIOREA study

#### Julien Demiselle (*speaker*)^1^, Guillaume Duval^1^, Jean François Hamel^1^, Anne Renault^4^, Dominique Perrotin^5^, Laurent Martin-Lefèvre^6^, Dominique Vivier ^7^, Daniel Villers^8^, Montaine Lefèvre^9^, René Robert^10^, Philippe Markowicz^11^, Sylvain Lavoué^12^, Anne Courte^13^, Eddy Lebas^14^, Stéphanie Chevalier^15^, Cédric Annweiler^1^, Nicolas Lerolle^1^

##### ^1^CHU, Angers, FRANCE; ^4^CHU, Brest, FRANCE; ^5^CHU, Tours, FRANCE; ^6^CHD Vendée, La Roche Sur Yon, FRANCE; ^7^CH, Le Mans, FRANCE; ^8^CHU, Nantes, FRANCE; ^9^CH, Morlaix, FRANCE; ^10^CHU, Poitiers, FRANCE; ^11^CH, Cholet, FRANCE; ^12^CHU, Rennes, FRANCE; ^13^CH, Saint-Brieuc, FRANCE; ^14^CH, Vannes, FRANCE; ^15^CH, Saint-Malo, FRANCE

###### **Correspondence:** Julien Demiselle - jdemiselle@gmail.com

*Annals of Intensive Care* 2019, **9(Suppl 1)**:F-01

**Introduction**: Improving the care for older patients in the ICU is a challenge both in terms of survival and of quality of life (QoL). We set up a prospective observational to assess the effectiveness of a combined geriatric and intensivist initial evaluation in determining clusters of older patients and the one-year outcome of these clusters.

**Patients and methods**: Patients aged 75 years and older admitted to 13 ICUs in Western France and requiring mechanical ventilation from September 2012 to December 2013 were included. Proxy members were consulted at ICU admission using a predefined comprehensive geriatric assessment. Survival was assessed at one year and comprehensive geriatric assessment was performed on place of living in survivors. Clustering was performed based on baseline characteristics and ICU admission parameters using Jaccard similarity measure.

**Results**: Five hundred and one patients were included in the analysis. Age was 80 ± 4 years, 53.5% were male. Ninety-two percent of patients were living at home before ICU admission, mean comorbidity burden assessed with CIRS-G score was 8.3 ± 4.3, 78.9% of patients had ADL score ≥ 5 and SOFA at ICU admission was 7 ± 3.7. Clustering resulted in three groups. Group 1 (n = 166) was characterized by older age, lower comorbidity burden and intermediate acute severity. Group 2 (n = 111) was characterized by lower age, intermediate comorbidity, and higher acute severity. Group 3 (n = 98) had higher age, higher comorbidity, and lower acute severity. 126 patients could not be clustered due to incomplete data. Clusters experienced different trajectories throughout ICU stay with higher ICU mortality for group 2, and the highest amount of organ support. Moreover, clusters evolution was different after hospital discharge. Overall, one-year survival was 53.8%, mortality was higher in group 2 and 3 when compared to group 1 (p = 0.03, see figure). 163 patients (62% of survivors) underwent one-year at-home evaluation. If a moderate decrease in independency was observed, perceived QoL remains satisfactory with 88% of patients indicated that they were happy or very happy. No difference in one-year geriatric evaluation was observed between clusters.

**Conclusion**: Comprehensive geriatric assessment and acute severity parameters are useful to identify three different patterns of patients with different outcomes. Such patterns may be useful for design future interventional studies. Long term survival of older patients after an ICU stay has improved compared to previous studies.


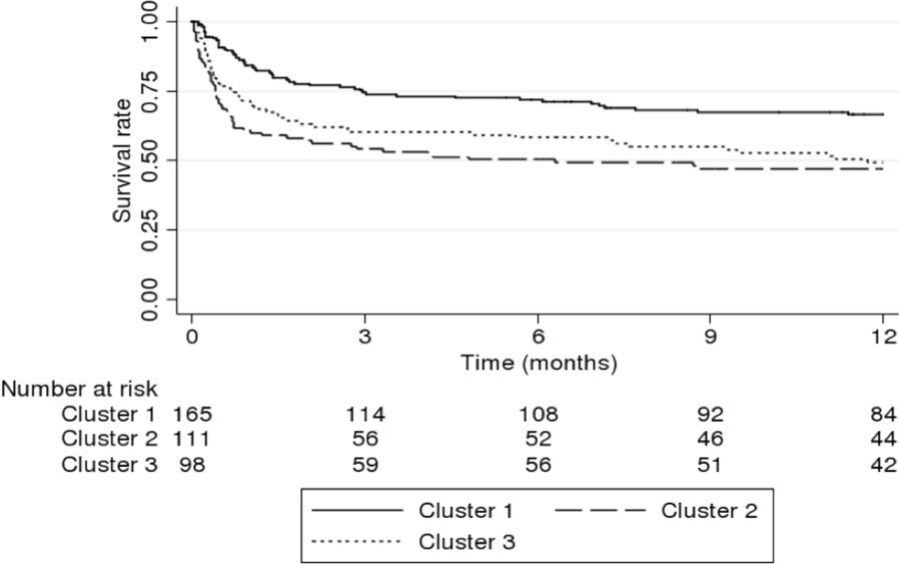


### F-02 Unique blood culture in ICU is associated with a dramatic reduction of blood culture contamination rates: an interrupted time-series study

#### Rafael Mahieu (*speaker*)^1^, Matthieu Eveillard^1^, Valérie Seegers^1^, Carole Lemarié^1^, Vincent Dubée^2^, Alain Mercat^1^, Achille Kouatchet^1^

##### ^1^Médecine Intensive et Réanimation, Médecine Hyperbare, CHU, Angers, FRANCE; ^2^Infectious and Tropical Diseases Department, Angers University Hospital, Angers, FRANCE

###### **Correspondence:** Rafael Mahieu - rafael.mahieu@chu-angers.fr

*Annals of Intensive Care* 2019, **9(Suppl 1)**:F-02

**Introduction**: Blood cultures (BC) remain the first-line tool for identification of the causative agent in patients with severe infection. However, several parameters affect diagnostic performances of BC. Sensibility depends on volume of collected blood + false-positive BC may arise from blood contamination during venipuncture. Our objective was to assess the effect of a unique high-volume blood culture (UBC) strategy on the rate of BC contamination and pathogen detection.

**Patients and methods**: An interrupted time-series study was conducted in a medical 24-bed ICU from January 2013 to December 2016. After the UBC protocol was implemented on January 2015, all patients with suspected bacterial infection had 40 mL of blood collected by a single phlebotomy. Ten milliliters of blood were inoculated into two BacT Alert FA aerobic bottles and two BacT Alert FN anaerobic bottles. Performing additional blood culture within 48 h was strongly discouraged unless a new bloodstream infection was suspected. Nurses’ and physicians’ knowledge on BC collection and practice skills were reinforced through an educational program on December 2014. Protocol adherence was evaluated at 2 and 12 months through monitoring of BC volume.

**Results**: During the study period, there were 3497 ICU stays with at least one BC. No difference was observed between the two periods regarding severity (IGS2 and SOFA at admission) or in-ICU death. After the educational program, the median volume of blood culture per bottle increased from 3.2 mL [95% confidence interval (CI) 3–3.5] to 9.1 mL [95% CI- 8.7–9.5] at 2 months and decreased to 7.1 mL [95% CI- 6.3–8] at 12 months (p < 0.001). During the UBC period, the rate of bloodstream infection per patient (bacteremia with a true pathogen) was 14.3%, as compared with 14.7% before (p = 0.1). The rate of BC contamination amongst positive BC decreased by 55.8% (from 43% before protocol implementation to 19% after; p < 0.001). The median number of BC bottles collected per week decreased by 58.5% (from 188 [interquartile range 149–235] before UBC to 78 [64–90] after protocol implementation), corresponding to a saving of 37,000 € per year.

**Conclusion**: Implementation of a UBC protocol was associated with an important decrease in rate of BC contamination and BC-associated costs, without affecting pathogen detection rate.

### F-03 Risk factors of post-traumatic stress disorder (PTSD) among ICU survivors

#### Marwa Zghidi (*speaker*), Imen Ben Saida, Said Kortli, Hend Zorgati, Abdelbaki Azouzi, Khaoula Meddeb, Ahmed Khedher , Mohamed Boussarsar

##### Farhat Hached University Hospital, Medical Intensive Care Unit, Sousse, TUNISIA

###### **Correspondence:** Marwa Zghidi - marwa_zghidi@outlook.fr

*Annals of Intensive Care* 2019, **9(Suppl 1)**:F-03

**Introduction**: Extended follow-up of ICU survivors has shown that many patients suffer from long-term physical and psychological sequalae. PTSD is an increasingly psychiatric disorder reported in ICU survivors. The aim was to determine the frequency of PTSD and its predictors.

**Patients and methods**: It is a mixed method study conducted in a medical ICU from January 2017 to January 2018. Data were obtained from medical records. At 3 months post-ICU discharge, patients were contacted by phone to complete the Impact Event Scale- Revised questionnaire (IES-R). Univariate and logistic regression analyses were used to identify variables independently associated with IES-R (≥ 33).

**Results**: 393 patients were admitted during the study period. 191 (48.6%) were discharged alive, 56(29.3%) were never successfully contacted and 21(11%) died within the 3 months’ period. 114 patients fulfilled the inclusion criteria. Patients’ characteristics were - mean age 56.29 ± 17.88 years; male, 66(57.9%); median Charlson comorbidity index, 1 [1–2]; mean SAPSII, 25.04 ± 12.1; invasive mechanical ventilation (IMV), 47(41.2%) and vasopressors use, 30(26.3%). The median duration of IMV and length of stay were respectively 0 days [0–4] and 6 days [4–10]. 25 (21.9%) patients met diagnostic criteria for PTSD. In univariate analysis, risk factors of PTSD were respectively for PTSD group and controls - age (42.7 ± 19 vs 60.1 ± 15.6, p = 0.000), female sex (64% vs 36%, p = 0.012); Alcoholism (24% vs 7.9%, p = 0.035); IMV (76% vs 31.5%, p = 0.000); Sedative use (76% vs 31.5%, p = 0.000); polyneuromyopathy (28% vs 2.2%, p = 0.000); delirium (16.7% vs 1.1%, p = 0.008); catheterization (56% vs 21.6%, p = 0.001); duration of IMV (6.58 ± 7 vs 2.32 ± 6.3, p = 0.005) and physical restraint (72% vs 23.6%, p = 0.000). On multivariable logistic regression, age (OR, 0.95 + 95%CI, [0.92 -0.98]; p = 0.001), female sex (OR, 3.88; 95%CI, [1.08 -13.9]; p = 0.038), physical restraint (OR, 6.27; 95%CI, [1.66–23.67]; p = 0.007) and polyneuromythy (OR, 11.15; 95%CI, [1.5–80.1]; p = 0.01) were identified as risk factors independently associated to PTSD.

**Conclusion**: PTSD is common in ICU survivors. Physical restraint, female sex, polyneuromyopathy and younger age were the only factors independently associated to PTSD.

### F-04 Help, my hospital is burning - Experience return on fire in the Guadeloupe University Hospital (GUH)

#### Frédéric Martino (*speaker*), Pascale Piednoir, Bertrand Pons, Benjamin Madeux, Khalid Elkoun, Elain Elie, Roland Lawson, Rémi Lazdunsk, Rémi Malhomme, Cyril Marimoutou, Juliette Masse, Alessia Napoleone, Eric Paris, Jean-Baptiste Putegnat, Asma Benguerrah, Morgane De Oliveira, David Fedida, Philippe Le Noach, Zakaria Mahi, Mathilde Pibarot, Mathieu Tournay, Killian Yao, Mohamed Zidani

##### CHU - Abymes, Pointe-À-Pitre, FRANCE

###### **Correspondence:** Frédéric Martino - frederic.martino@chu-guadeloupe.fr

*Annals of Intensive Care* 2019, **9(Suppl 1)**:F-04

**Introduction**: The GUH fire causing its total evacuation is an unprecedented event in France. We report factual elements of the first days after the disaster involving intensive care unit (ICU) activities with specific proposed imposed responses, as it is the sole ICU of Guadeloupe island (near half million people).

**Patients and methods**: Data are extracted from ICU medical files, experience reports, and Emergency department softwares.

**Results**: The fire started on 28.11.2017 around 13:45 on the 2nd floor of the building, between the ICU (1st floor) and operating room (3rd floor). At 14:00, the ICU (25 occupied beds over 26, including 12 ventilated patients) was invaded by a thick smoke, not triggering the fire alarm. The evacuation of the ICU was decided by doctors before the activation of the official evacuation plan (> 1 h) in absence of any administrative instructions.

The 12 ventilated patients were relocated at the radiology level (access to medical gases), 8 non-ventilated patients evacuated to the outdoor car park, 5 patients to other services. At 17:00 all patients were extracted from the ICU. Patients were then evacuated to other centers- 3 ventilated patients to the Basse Terre public hospital (Post Anesthesia Care Unit), 9 ventilated patients and 8 non-ventilated patients to a private hospital located at 11 km, into a unit of 12 ambulatory surgery beds, thus the same day re-affected as ICU with 2 to 3 patients per room.

At 22:53, all ICU patients were evacuated from the GUH. On 29.11.2017, 11 patients were prioritized for medical evacuation to the Martinique University Hospital (MUH) thanks to military vectors - 8 patients simultaneously (CASA military aircraft), and 3 by medical helicopter (SAMU). No worse initial progression was noted, except for 1 patient (septic shock needing intubation and norepinephrine) at the radiology level. ICU activities were heavily impacted during this period by difficulties related to emergency services, medico-surgical services and support structures (laboratories, radiology, pharmacy) rearrangement.

**Conclusion**: This disastrous never experienced situation had a limited immediate impact (no deaths) due to the fast management of patient’s transfer, related to ICU doctors initiatives. Regional support (MUH and military vector) helped to propose an immediate response for the most severe ICU patients. Mid- and long-term consequences are significant for evacuated patients as well as for the Guadeloupe care provision, in term of ICU and other hospital components, related to the distance away from France.

### F-05 Evaluation and description of alarms in an Intensive Care Unit, Improvement of professional practices- optimizing monitoring and reducing noise pollution

#### Pierre-Yves Delannoy (*speaker*)^1^, Lydie Martel^2^, Nicolas Boussekey^2^, Damien Thellier^2^, Hugues Georges^2^, Olivier Robineau^2^, Olivier Leroy^2^

##### ^1^CH, Marcq En Baroeul, FRANCE; ^2^CH, Tourcoing, FRANCE

###### **Correspondence:** Pierre-Yves Delannoy - pydelannoy@ch-tourcoing.fr

*Annals of Intensive Care* 2019, **9(Suppl 1)**:F-05

**Introduction**: The excess of alarms in intensive care has multiple consequences- the noise generated alterate the quality of patient’s stay and participate in the genesis of the phenomenon called alarm fatigue. This, by altering the quality of surveillance, directly impacts patient safety in a pejorative term. The objective of this project is to improve the quality of care and working conditions in intensive care unit in Tourcoing by optimizing the monitoring of our patients through a collaboration between the medical team and the company providing the monitoring products.

**Patients and methods**: An analysis of alarms from Philips Intelivue monitors for one month is performed, then corrective measures to reduce their number are implemented through a collaborative DMAIC approach (Define, Measure, Analyze, Improve, Control). These corrective measures include a personalization of the monitoring with a medical reflection on the alarm’s thresholds and creation of specific profile patient, training of caregivers on the good use of the monitoring, creation of an alarm culture via these trainings and the creation of communication tools. To these actions are added technical interventions on intensive care ventilators and monitors. The impact of alarms on caregivers and patients present during the analysis period are evaluated using questionnaires. A second alarm analysis assess an evaluation of the effectiveness of the implemented measures.

**Results**: 127.927 alarms (equivalent to 243 alarms day patient) including 40.118 vital alarms, 58.647 critical and 29.162 techniques are found in the first period. The analysis of the patient questionnaire is not very contributive with 20 usable questionnaires. Among the responders, the majority did not feel any significant discomfort during their stay. The health questionnaire has a response rate of 91% (n = 51). Responders believe that there is a decrease in attention and an increase in reaction time due to false alarms. They also believe that irrelevant alarms disrupt the quality of the patient’s stay and that better alarm management could prevent incidents. Nearly 86% of caregivers say that alarms could not be heard and were missed. In the second period, a 30% reduction in the total number of alarms (90,096 alarms, equivalent to 202 alarms day patient, p = 0.002) is obtained.

**Conclusion**: By creating and implementing targeted corrective measures with DMAIC approach, a reduction in the number of alarms is achieved. This reduction probably enhance patient safety and improve healthcare environement for patients and caregivers.

### F-06 Can we routinely use electronic medical data management systems to drive the prescription of red blood cell transfusion in Canadian and British pediatric intensive care units?

#### Camille Jutras (*speaker*)^1^, Geneviève Du Pont-Thibodeau^1^, Marisa Tucci^1^, Simon Stanworth^2^, Samiran Ray^3^, Barney Scholefield^4^, Samuel Kadoury ^5^, Philippe Jouvet^1^, Patricia Fontela^6^, Jacques Lacroix^1^

##### ^1^CHU Sainte Justine, Montreal, CANADA; ^2^NHS Blood & Transplant Oxford John Radcliffe Hospitals, Oxford, UNITED-KINGDOM; ^3^Great Ormond Street Children’s Hospital, London, UNITED-KINGDOM; ^4^Birmingham Children’s Hospital, Birmingham, UNITED-KINGDOM; ^5^Centre de recherche, CHU Sainte-Justine, Montreal, CANADA; ^6^Montreal Children’s Hospital, Montreal, CANADA

###### **Correspondence:** Camille Jutras - camille.jutras@gmail.com

*Annals of Intensive Care* 2019, **9(Suppl 1)**:F-06

**Introduction**: There is evidence that electronic medical data management systems (eMDMS) may help practitioners to improve the appropriateness of red blood cell (RBC) transfusion in hospitalised adults, but there is a lack of data supporting this in children. It is unclear if eMDMS are used in paediatric intensive care units (PICUs). This survey aims to document the availability of eMDMS in British and Canadian PICUs and characteristics of the data recorded.

**Patients and methods**: An electronic self-administered questionnaire was sent out thrice, using Survey Monkey, from November 2017 to February 2018, to the director of 16 Canadian and 27 British PICUs. Respondents were asked to describe their PICU and to indicate what eMDMS was used in their institution.

**Results**: Among the 43 units studied, 12 Canadian and 25 British PICUs (37/43 = 86%) answered to the survey. Three of them were excluded because they exclusively took care of premature infants and newborns. Some eMDMS were used in 24 out of the 34 remaining PICUs (71%). The table below details the data collected by these eMDMS in PICUs. Seventeen PICUs (71%) used Windows as their eMDMS operating system. Two types of Electronic Medical Records (EMR) were used to document patient care- IntelliSpace Critical Care and Anesthesia (ICCA, Philips, 9 PICUs) and Allscripts Professional (1 PICU) (total- 42%). EMR name was unknown in 14 instances. Electronic medical data were known to be stored in a Structured Query Language (SQL) server in seven PICUs (29.17% %).

**Conclusion**: Data management by eMDMS is common in British and Canadian PICUs. Computerized physician order for RBC transfusion is reported to be available in 79.17% of these PICUs. Using eMDMS to enable an electronic medical decision support tool for RBC transfusion should be feasible in most PICUs, and may facilitate efficient data collection for clinical trials.

**Table:** Table 1. Data management by eMDMS in 24 PICUs

	Yes	No	Unknown
Data collected by eMDMS			
Main diagnosis	22 (92%)	2 (8%)	0
Co-morbidities	22 (92%)	2 (8%)	0
Symptoms and signs	21 (88%)	3 (13%)	0
Laboratory data	24 (100%)	0	0
Data on red blood cell (RBC) transfusions	24 (100%)	0	0
Plasma and/or platelet transfusions	24 (100%)	0	0
Outcomes	20 (83%)	4 (17%)	0
Dates when events happen†	23 (96%)	1 (4%)	0
Computerized physician order (CPO) entry			
Prescription of RBC transfusion	19 (79%)	5 (21%)	0
Prescriptions other than RBC transfusion	19 (79%)	5 (21%)	0

*†* *Example of events: transfusion, adverse outcomes, death…*

**Table:** Table 1. Availability of physiologic measures for RBC transfusions in 34 PICUs

Physiologic measures	Not available	Some beds	All beds
Systemic physiologic measures			
Point of care PaO_2_	4 (12%)	2 (6%)	28 (82%)
Pulse oximetry (SpO_2_)	0	0	34 (100%)
Mean airway pressure (MAP) *	0	0	34 (100%)
Swan-Ganz catheter	29 (85%)	3 (9%)	2 (6%)
Systemic O_2_ consumption †	27 (79%)	5 (15%)	2 (6%)
Point of care lactate measurement	4 (12%)	1 (3%)	29 (85%)
Heart rate variability	14 (41%)	0	20 (59%)
Plethysmographic variability	23 (68%)	1 (3%)	10 (29%)
Near infrared spectroscopy (NIRS)	13 (38%)	15 (44%)	6 (17%)
Microvascular blood flow	33 (98%)	1 (3%)	0
Organ specific or local physiologic measurements			
Troponin	2 (6%	2 (6%)	30 (88%)
Pro-brain natriuretic protein (BNP)	17 (50%	2 (6%)	15 (44%)
Brain PO_2_ measured in situ ‡	32 (94%)	2 (6%)	0
Jugular bulb O_2_ saturation	27 (79%)	5 (15%)	2 (6%)
Gastric tonometry (pHi)	31 (91%)	1 (3%)	2 (6%)
Peripheral O_2_ extraction ¶	31 (91%)	1 (3%)	2 (6%)
Microvascular blood flow	34 (100%)	0	0

* *MAP can be used to calculate oxygenation index (OI) and oxygenation saturation index (OSI), two physiologic markers advocated by some experts, They can be calculated using the following equations given that FiO*_*2*_ *is always available in mechanically ventilated patients:*

*OI* = *(FiO*_*2*_ *x MAP* *×* *100)/PaO*_*2*_*); OSI* = *(FiO*_*2*_ *x MAP x 100)/SpO*_*2*_*).*

*†* *Measured by equipment like Deltatrac metabolic monitor.*

*‡* *Brain PO*_*2*_ *measured by intracerebral device like Licox*^*®*^ *Brain Tissue Monitoring System.*

*¶* *Peripheral O*_*2*_ *extraction measured by pulse oximetry.*

### F-07 Short and long term outcome of patients with systemic rheumatic disease related interstitial lung disease admitted to intensive care unit- a multicentre retrospective study

#### Romaric Larcher (*speaker*), Lorrain Banuls, Fanny Garnier, Matthieu Amalric, Laura Platon, Jonathan Charbit, Kevin Chalard, Samir Jaber, Boris Jung, Kada Klouche

##### Montpellier University Hospital, Montpellier, FRANCE

###### **Correspondence:** Romaric Larcher - r-larcher@chu-montpellier.fr

*Annals of Intensive Care* 2019, **9(Suppl 1)**:F-07

**Introduction**: Owing their high mortality rate, admission to the intensive care unit (ICU) of patients with interstitial lung disease (ILD) is questioning. Systemic rheumatic diseases related ILD (SRD-ILD) seem to be associated with a better outcome but data are still lacking. We aimed therefore to evaluate short and long-term outcome and prognostic factors for SRD patients admitted to the ICU.

**Patients and methods**: This multicenter retrospective study was conducted, between 2006 and 2016, in five French ICUs and included patients with SRD-ILD admitted for acute respiratory failure. In-hospital and one-year crude mortalities were assessed and potential prognostic factors were identified using logistic regression.

**Results**: Seventy-one patients (female- 55%, mean age- 65 years [58–74], median SAPS2 43 [32–59], median SOFA Score 7 [4–9]) entered the study. ILD was related to connective tissue disease (43%), vasculitis (28%), myositis (13%), sarcoidosis (13%), and ankylosing spondylitis (3%). Causes of acute respiratory failure were sepsis (48%), pulmonary flare-up (28%) and miscellaneous (24%). Fifty-five patients (77%) required mechanical ventilation, 38 patients (53%) vasoactive drugs and 24 (34%) renal replacement therapy. One patient had an extracorporeal membrane oxygenation. Occurrence of severe, moderate and mild ARDS was 39%, 21%, 28% respectively. Thirty-seven survived at hospital discharge and 24 one year later. By univariate analysis, the following factors were significantly associated with mortality- ICU admission SAPS II (p = 0.009), PaO2/FiO2 ratio (p = 0.007), vasoactive drugs (p = 0.01) and mechanical ventilation requirement (p = 0.02). Among these factors, multivariate analysis showed that only low PaO2 FiO2 ratio (OR 3.63, 95% CI [3.59–298.74]) was associated with one year- mortality.

**Conclusion**: In our study, SRD-ILD patients admitted to the ICU have an in-hospital and one-year crude mortality rate at 48% and 66% respectively. Though this mortality rate remains high, it should not discourage intensivists to admit these patients. We found also that a low PaO2/FiO2 ratio during ICU stay was associated with one-year mortality. Further studies are however needed to best help physicians in the ICU management of ILD patients.

### F-08 Impact of Advance Directives on the decision-making in Intensive Care Unit

#### Margot Smirdec (*speaker*), Bruno Pereira, Alexandre Lautrette

##### CHU Clermont-Ferrand, Clermont-Ferrand, FRANCE

###### **Correspondence:** Margot Smirdec - msmirdec@chu-clermontferrand.fr

*Annals of Intensive Care* 2019, **9(Suppl 1)**:F-08

**Introduction**: Physicians do not know how to follow the patients’ wishes when they are unable to express themselves. We assessed the impact of Advances Directives (AD) and how they have been written on the physician’s decisions.

**Patients and methods**: A multicentre, prospective, interventional, simulation study was carried out. Eight patients were recruited and wrote AD after receiving clear and complete information by video and interview with one ICU physician. Two simulation scenarios including ten questions about ICU admission and situations of withholding withdrawing therapies using the patients’ characteristics were submitted to ICU physicians from 28 French ICU, in three rounds (R)- simulation without knowledge of the patient’s AD (R1), with these AD (R2) and with these AD and the knowledge of how they were carried out (R3).

**Results**: The qualitative analysis of these 8 AD highlights a form of living will or end-of-life will and not formal guidelines on medical care. The results were performed on complete data of 102 physicians [Figure 1]. The variability between physicians themselves was high- among the 80 questions of R1, there were 37, 26 and 17 questions with an agreement > 80%, 80–60% and < 60% respectively. The AD significantly decreased the number of questions with an agreement > 80%, and increased the number of questions with an agreement < 60% (p = 0.02). There was no difference between the rounds 2 and 3 (p = 0.84). Few physicians’ characteristics were associated with the inter-individual variability. The intra-individual variability between R1 and R2 was very high (52 questions on 80 with kappa coefficient k < 0.4) and the AD were significantly associated with this variability after adjustment on characteristics of the physicians and the patients (p < 0.001). It is relevant to notice that the knowledge of how AD were carried out has a very low impact on the physicians’ decisions.

**Discussion**: We were expecting find some help in the AD of patients to diminish this variability among physicians and respect the patients’ autonomy. But the variability between the physicians themselves was increased in our study. We assume that this variability reflects a difference in the interpretation of these AD among physicians themselves.

**Conclusion**: The AD have a major impact on the physicians’ decisions for admission, withholding and withdrawing therapies decisions and increase the inter-individual variability but not the knowledge of how these AD have been written. It might be a limit of the autonomous model.


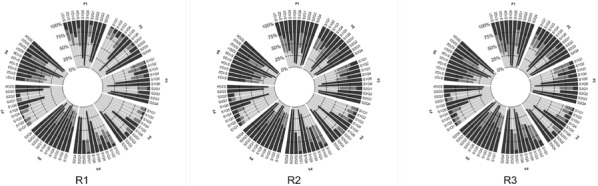


### F-09 End of life decisions in acute respiratory failure immunocompromised patients

#### Gaston Burghi (*speaker*)^1^, Victoria Metaxa^2^, Peter Pickkers^3^, Marcio Soares^4^, Anders Perner^5^, Jordi Rello^6^, Philippe Bauer ^7^, Andry Van de Louw^8^, Pleun Hemelaar^9^, Virginie Lemiale^10^, Fabio Silvo Taccone^11^, Ignacio Martin Loeches^12^, Tine Sylvest Meyhoff^5^, Jorge Salluh^4^, Peter Schellongowski^15^, Katerina Rusinova^16^, Nicolas Terzi^17^, Geeta Mehta^18^, Massimo Antonelli^19^, Achille Kouatchet^20^, Andreas Barrat Due^21^, Miia Valkonen^22^, Pearl Landburg^23^

##### ^1^Terapia Intensiva, Hospital Maciel, Montevideo, Montevideo, URUGUAY; ^2^Frank Stansil Critical Care Unit King’s College Hospital, Londres, UNITED-KINGDOM; ^3^The Department of Intensive Care Medicine (710), Radboud University Medical Center, Nijmegen, THE NETHERLANDS; ^4^Department of Critical Care and Graduate Program in Translational Medicine, D’Or Institute for Research and Education, Programa de Pós-Graduação em Cl, Rio de Janeiro, BRAZIL; ^5^Department of Intensive Care, Rigshospitalet, University of Copenhagen, DENMARK; ^6^Universitat Autonòma de Barcelona, European Study Group of Infections in Critically Ill Patients (ESGCIP), Barcelona, SPAIN; ^7^Pulmonary and Critical Care Medicine, Mayo Clinic, Rochester, UNITED STATES; ^8^Pulmonary and Critical Care Medicine, Mayo Clinic, Rochester Division of Pulmonary and Critical Care, Penn State University College of Medicine, Hershe, Hershey, UNITED STATES; ^9^Pulmonary and Critical Care Medicine, Mayo Clinic, Rochester Division of Pulmonary and Critical Care, Penn State University College of Medicine, Hershe, Nijmegen, THE NETHERLANDS; ^11^Department of Intensive Care, Hôpital Erasme, Université Libre de Bruxelles (ULB), Brussels, BELGIUM; ^12^Department of Intensive Care Medicine, Multidisciplinary Intensive Care Research Organization (MICRO), St. James’s Hospital, Dublin, IRELAND; ^15^Department of Medicine I, Medical University of Vienna, Vienna, Vienne, AUSTRIA; ^16^Department of Anesthesiology and Intensive Care Medicine and Institute for Medical Humanities, 1st Faculty of Medicine, Charles University in Prague a, Prague, THE CZECH REPUBLIC; ^17^CHU Grenoble Alpes, Service de réanimation médicale, Faculté de Médecine, INSERM, U1042, Université Grenoble-Alpes, Grenoble, FRANCE; ^18^Department of Medicine and Interdepartmental Division of Critical Care Medicine, Sinai Health System, University of Toronto, CANADA; ^19^Agostino Gemelli University Hospital, Università Cattolica del Sacro Cuore, Rome, ITALY; ^20^Department of Medical Intensive Care Medicine, University Hospital of Angers, FRANCE; ^21^Department of Emergencies and Critical Care, Oslo University Hospital, Oslo, NORWAY; ^22^Division of Intensive Care Medicine, Department of Anesthesiology, Intensive Care and Pain Medicine, University of Helsinki and Helsinki University Ho, FINLAND; ^23^Department of Critical Care, University Medical Center, Groningen, FINLAND

###### **Correspondence:** Gaston Burghi - burghig@gmail.com

*Annals of Intensive Care* 2019, **9(Suppl 1)**:F-09

**Introduction**: In recent years, there has been an increase in immunocompromised patients admitted to the intensive care unit (ICU). End-of life decisions are challenging in these severe patients with often life-threatening underlying disease. The main aim of this study was to identify patient and organizational factors associated to decisions to forgo life-sustaining therapies (DFLSTs) among immunocompromised ICU patients.

**Patients and methods**: A secondary analysis of the EFRAIM study including 1611 immunocompromised patients with acute respiratory failure enrolled in 68 ICUs from 16 countries between October 2015 and June 2016 was performed. A multivariate logistic analysis was performed to identify independent predictors of DFLSTs.

**Results**: In the 1382 patients with data about DFLSTs available, 485 (35%) had a DFLST. Figure 1 presents the code status on ICU admission and the final decision about DFLSTs for each code status. The following variables were independently associated with increased incidence of DFLST: age (OR 1.024 per one year increase, IC 95% 1.012–1.035 + p < 0.0001), SOFA at ICU admission (OR 1.065, IC 95% 1.025–1.107, p = 0.0013), need for orotracheal intubation (OR 1.478, IC 95% 1.068–2.004, p = 0.0183), and performance status measured by the Eastern Cooperative Oncology Group scale (ECOG) (OR 1.7 per point, IC 95% 1.504–2.028, p < 0.0001), units without protocoled admission criteria (OR 1.701, IC 95% 1.176–2.462, p = 0.0022), palliative care involvement (OR 1.688, IC 95% 1.216–2.343, p = 0.0018) and units with frequent admission of transplant patients (OR 1.662, IC 95% 1.200–2.302, p = 0.0022). Two variables were independently associated with a decreased incidence of DFLST, namely acute respiratory failure with an easy aetiology identification (OR 0.913, IC 95% 0.860–0.968, p = 0.0025) and the use of checklists for global ICU care (OR 0.531, IC 95% 0.387–0.728, p < 0.0001). Centre effect was not significant after multivariate analysis.

**Conclusion**: The current study suggests that, in addition to patient-related factors, ICU characteristics and critical-care organization also influence decisions to forgo life sustaining therapies. Palliative care involvement, frequent admission of transplant patients, protocoled admission criteria at admission and the use of checklists are organizational variables associated with DFLSTs.


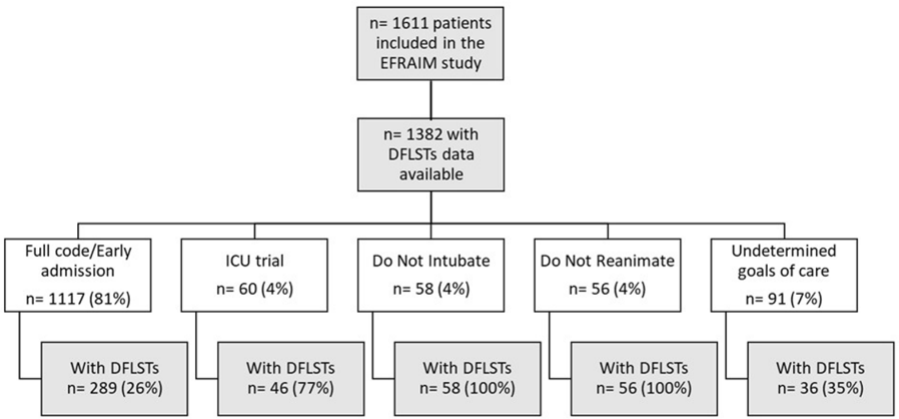


### F-10 Practices assessment ten years after setting up a procedure of collegial decision for Withholding or Withdrawing Treatment (WWT) in Intensive Care

#### Marc Amouretti (*speaker*), Martha Gomis, Fabien Merrina, Sarah Benhamida, Nicolas Lau, Matthieu Le Meur, Remy Paulet, Nicolas Roucaud, Jordane Lebut, Martial Thyrault

##### Groupe Hospitalier Nord Essonne, Longjumeau, FRANCE

###### **Correspondence:** Marc Amouretti - marc.amouretti@gmail.com

*Annals of Intensive Care* 2019, **9(Suppl 1)**:F-10

**Introduction**: Since the 2002 “Kouchner law” and the 2016 “Leonetti Claeys law”, WWT decisions have become essential in discussions within intensive care units. Our Intensive Care Unit (ICU) has conducted a work on this topic in 2008, leading to the implementation of WWT procedural forms and follow-ups. We assessed our practices 10 years after having implemented this traceability.

**Patients and methods**: Patient Limitation Records (PLR) are initiated and reassessed during weekly ICU meetings bringing together medical and paramedical staff. They can also be initiated at any other time during exceptional meetings. PLR are initiated at the request of any member of the paramedical team, medical team or patient’s entourage as soon as the meaningfulness of continuing care is contested. They include advanced medical instructions, an estimation of the patient’s quality of life before and after the ICU stay, patient’s wellbeing during the stay, interviews with the patient, his relatives and his practitioners. They also mention the conditions and time of application for the limitation. Analyzed records extend over a period of 18 months ending in June 2018. In 2008, the analysis period lasted 10 months from September to June.

**Results**: 106 PLR were established over the 2017–2018 period compared to 32 in 2008. 70% of patients are limited during the first ethical meeting during the 2017–2018 period. 18% have no limitation of life sustaining treatments. Patient’s will is unknown in 72% of cases. Among patients whose will is known, 21% express their opposition to therapeutic relentlessness. Patient’s general practitioner interview before the meeting is made for 42% of the patients. The most frequent therapeutic limitations are cardiac arrest resuscitation (70%) and renal replacement therapy (57%). Invasive mechanical ventilation is limited in 35% of cases. Nutrition and hydration are never limited.

**Conclusion**: Ten years after setting up a WWT procedure in our ICU, limitations are more frequent, appear earlier, concern younger patients, and are more often at the paramedic initiative, which reflects a stronger involvement of caregivers in end of life issues.


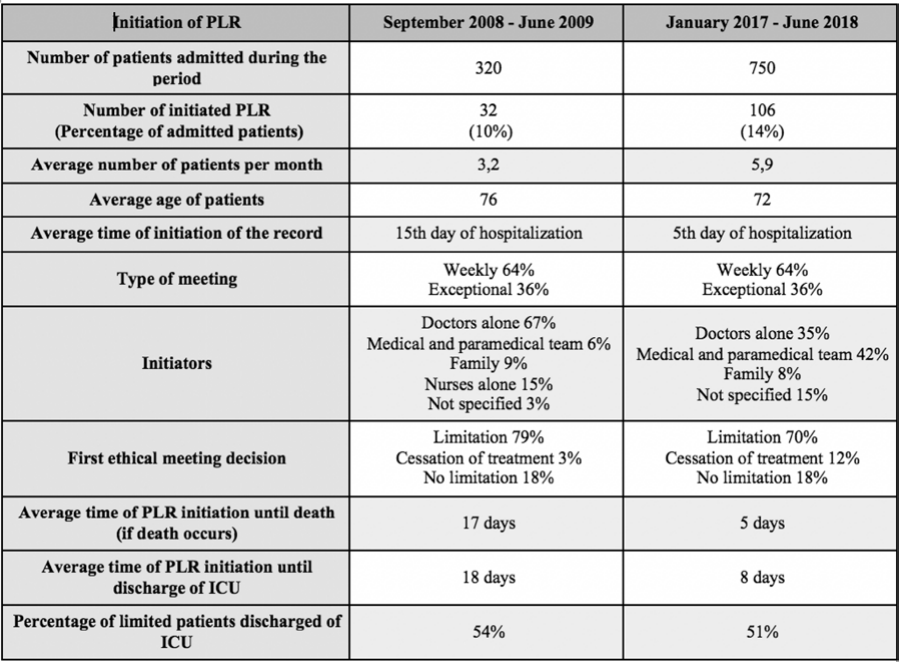


### F-11 Assessment of the psychological consequences of hospitalization in intensive care unit- Patients and their relatives are both concerne

#### Ghada Sbouii (*speaker*)^1^, Nesrine Baili^2^, Rabia Atig^2^, Olfa Beji^2^, HoussemHmouda^2^

##### ^1^Yasminet hospital, Kairouan, ABKHAZIA; ^2^Hopital sahloul sousse TUNISIA, Sousse, TUNISIA

###### **Correspondence:** Ghada Sbouii - ghadasrlf@hotmail.com

*Annals of Intensive Care* 2019, **9(Suppl 1)**:F-11

**Introduction**: The aim of our study was to evaluate the psychological impact of critical illness on patients and their relatives in a Tunisian medical ICU (MICU).

**Patients and methods**: In this study, included the patients who were admitted in our MICU from January 2015 and December 2016. We used the Impact of Events Scale (IES) 22-items for patients. Scores above 30 on the IES indicate severe psychological trauma symptoms, and individuals scoring in the range between 24 and 35 are likely to meet diagnostic criteria for PTSD. The Burden (Mini-Zarit) 7-items was used for relatives. All patients and relatives were interviewed by telephone.

**Results**: We contacted 46 families, 18 patients (39%) died, 28 patients (60.9%) participated with their relatives to this study, sex ratio was F M = 1.09, mean age was 75 years [65 to 90 years], average length of stay (LOS) was 11.89 days [1 to 60 days], 45.7% of patients had mechanical ventilation with a mean duration of 4.84 days, 8.7% of the patients had a tracheostomy, dementia was diagnosed in 6.5% of patients, 78.3% of the patients were autonomous, and the main reason of ICU admission was respiratory distress in 30.4%. The average of IES on patients was 22.7 [6 to 35], the IES was significantly correlated with male gender (p = 0.039), LOS in the ICU (p = 0.043), oro-tracheal intubation (OTI) (p = 0.03), tracheostomy (p = 0.013), and use of vasopressor agents (p = 0.045). The average of Mini-Zarit on relatives was 3.35 [1.5 to 5.5], the Mini-Zarit was significantly correlated with LOS (p‹0.001), OTI (p = 0.001), duration of OTI (p = 0.045), tracheostomy (p = 0.012), duration of tracheostomy (p = 0.024), use of vasopressor agents (p = 0.034) and the APACHE II score (p = 0.047).

**Conclusion**: Patients recovering from critical illness can be left with significant physical and cognitive problems that can deeply affect quality of life of both patients and relatives. Understanding the nature of the relationship between critical illness and PTSD is a challenge that demands attention, particularly in an era when mental health professionals are beginning to recognize the profound costs associated with this psychiatric syndrome.

### F-12 Impact of frailty on elderly patients (≥ 80 years) admitted in French Intensive Care Units: a post hoc analysis from the international VIP study

#### Jérémy Rosman (*speaker*)^1^, Aurélien Cordonnier^2^, Xavier Forceville^3^, Guillaume Besch^4^, Hervé Mentec^5^, Philippe Michel^6^, Philippe Michel ^7^, Lucie Vettoretti^8^, Jérémy Bourenne^9^, Nathalie Marin^10^, Max Guillot^11^, Nadia Aissaoui^12^, Cyril Goulenok^13^, Nathalie Thieulot-Rolin^14^, Jonathan Messika^15^, Lionel Lamhaut^16^, Cyril Charron^17^, Bertrand Guidet^18^, Philippe Mateu^1^

##### ^1^Service de Médecine Intensive Réanimation, CH, Charleville-Mézières, FRANCE; ^2^Département de l’Information Médicale, CH, Charleville-Mézières, FRANCE; ^3^Service de réanimation médico-chirurgicale, CH, Meaux, FRANCE; ^4^Département d’Anesthésie Réanimation Chirurgicale, CHR, Besançon, FRANCE; ^5^Service de Réanimation Polyvalente, CH Victor Dupouy, Argenteuil, FRANCE; ^6^Service de Réanimation médico-chirurgicale, CH de Carnelle - Portes de l’Oise, Saint-Martin-Du-Tertre, FRANCE; ^7^Service de Réanimation médico-chirurgicale, CH René Dubos, Pontoise, FRANCE; ^8^Service de réanimation médicale, CHU, Besançon, FRANCE; ^9^Service de Réanimation des Urgences et Médicale, CHU - Timone, Marseille, FRANCE; ^10^Service de réanimation médicale, Centre Hospitalier Cochin, Paris, FRANCE; ^11^Service de Réanimation médicale, Hôpital de Hautepierre, Strasbourg, FRANCE; ^12^Service de Réanimation médicale, Hôpital Européen Georges Pompidou, Paris, FRANCE; ^13^Service de Réanimation Médicale, Hôpital Privé Jacques Cartier, Massy, FRANCE; ^14^Service de réanimation, Centre Hospitalier de Melun, FRANCE; ^15^Service de Réanimation Médico-Chirurgicale, Centre Hospitalier Louis Mourier, Colombes, FRANCE; ^16^Service de réanimation polyvalente, Hôpital Necker, Paris, FRANCE; ^17^Service de réanimation médico-chirurgicale, Hôpital Ambroise Paré, Boulogne-Billancourt, FRANCE; ^18^Service de réanimation médicale, Hôpital Saint-Antoine, Paris, FRANCE

###### **Correspondence:** Jérémy Rosman - jrosman@ch-charleville-mezieres.fr

*Annals of Intensive Care* 2019, **9(Suppl 1)**:F-12

**Introduction**: Very elderly Intensive care Patients (VIP1) is an international multicentric prospective study endorsed by the European Society of Intensive Care Medicine assessing prognosis of elderly patients (≥ 80 years) admitted in the intensive care unit (ICU). Our study is a subgroup analysis of French patients.

**Patients and methods**: All elderly patients (≥ 80 years) admitted in 18 French ICUs and included in VIP1 study from October 2016 to May 2017 were analyzed in a post hoc subgroup study. Frailty was defined as a Clinical Frailty Scale ≥ 5/9. Multivariable analysis [with adjusted odd ratio (aOR) and 95% confidence interval (IC 95%)] was performed to determine factors associated with in-ICU and 30-days mortality.

**Results**: Among 368 patients admitted in French ICUs, 38% were frail (44% in the entire cohort). Compared to non-frail patients, frailty was associated with the similar in-ICU (34% vs 35%, p = 0.93) and 30-days mortality (51% vs 44%, p = 0.22), despite a lower severity (SAPS2 46 vs 54, p = 0.04), lower use of mechanical ventilation (46% vs 62%, p = 0.003), and lower use of vasoactive drugs (39% vs 55%, p = 0.004). However, withholding was more frequent (57% vs 35%, p < 0.001), but not withdrawing (20% vs 21%, p = 0.76). In multivariable analysis, frailty was not an independent risk factor for mortality, contrary to withdrawing [aOR 18.8 (IC95% 8.1–43.8)], withholding [aOR 3.9 (IC95% 2.0–7.4)], use of mechanical ventilation [aOR 2.3 (IC95% 1.1–5.0)] and SOFA at admission (per one point increase) [aOR 1.2 (IC95% 1.1–1.3)].

**Conclusion**: In elderly patients admitted in French ICUs, frail patients have the same mortality than non-frail patients, despite a lower initial severity and higher frequency of withholding decisions.

**Table:** 

	Univariate			Multivariate	
	Alive (n = 241)	Deceased (n = 126)	p-value	Adjusted OR (CI_95 %_)	p-value
Age, years	85 [81–89]	83 [81–86]	0.18		
Age 80–89 years	212 (88%)	114 (91%)	0.47		
Sex, male Frailty	118 (49%) 91 (38%)	66 (52%) 47 (37%)	0.53 0.93		
Cause of admission					
Respiratory failure	108 (45%)	36 (29%)	0.002	/	NS
Shock	27 (11%)	36 (29%)	< 0.001	/	NS
Elective surgery	9 (4%)	4 (3%)	1.0		
Urgent surgery	11 (5%)	3 (2%)	0.40		
Neuro (non trauma)	17 (7%)	11 (9%)	0.57		
Medical admission	218 (91%)	113 (90%)	0.81		
Trauma	3 (1%)	6 (5%)	0.07		
Prior hospital stay > 7 days	33 (14%)	23 (18%)	0.25		
Admission SOFA	5 [3–7]	12 [8–14]	< 0.001	1.2 (1.1–1.3)	< 0.001
SAPS 2	43 [37–50]	65 [57–82]	< 0.001	not analysed	
ICU interventions					
Non invasive ventilation	91 (38%)	36 (29%)	0.08		
Invasive ventilation	106 (44%)	99 (79%)	< 0.001	2.3 (1.1–5.0)	0.04
Vasoactive drugs	89 (37%)	91 (72%)	< 0.001	**/**	NS
Renal replacement therapy	22 (9%)	22 (18%)	0.02	**/**	NS
Withholding	72 (30%)	87 (69%)	< 0.001	3.9 (2.0–7.4)	<0.001
Withdrawing	10 (4%)	65 (52%)	< 0.001	18.8 (8.1–43.8)	<0.001

### F-13 Evaluation of patients refused admission to intensive care unit

#### Alix Leurent (*speaker*)^1^, Matthieu Jamme^1^, Claire Pichereau^1^, Siu-Ming Au^1^, Christophe Barbier^1^, Yann Loubieres^1^, Jan Hayon^1^, Renaud Getti^2^, Hervé Outin^1^, Omar Ben Hadj Salem^3^

##### ^1^Reanimation, CHI Poissy, Poissy, FRANCE; ^2^SAU, CHI Poissy, Poissy, FRANCE; ^3^ Cochin, Paris, FRANCE

###### **Correspondence:** Alix Leurent - alix_leurent@hotmail.fr

*Annals of Intensive Care* 2019, **9(Suppl 1)**:F-13

**Introduction**: Some patients referred to intensive care unit (ICU) are refused by intensivist because they are considered to be too well or too ill to benefit from intensive care treatment. This study aims to evaluate factors associated with decision to refuse ICU admission and to assess the outcome of excluded patients.

**Patients and methods**: All inpatients referred to our ICU between February and August 2018 were included in the analysis. All patients were evaluated in emergency unit or in hospitalization areas by a senior intensivist. Refused patients were considered to be too well (inappropriate referral (IR)) or too ill (futility) for ICU admission. The main outcome was survival 28 days after first referral to ICU. Patient characteristics, number of beds available at the time of referral, quality of life 28 days after ICU referral were also assessed.

**Results**: Out of 379 patients, 267 were admitted and 112 were refused (29.6%). Reasons for refusal were futility (“too ill”) (n = 68 (60.7%)) and IR (“too well”) (n = 44 (39.3%)). The median mortality probability model (MPMII) score for IR group and for futility group at day 0 was -1.65 and -0.4. Admission request came from emergency unit (65.2% (n = 73)), medical department (24% (n = 27)) or surgery department (10.8% (n = 12)). The main outcome for futility and IR groups were 52.9% and 4.5% respectively. In the futility group, modified Rankin score increased from 3.52 to 4.15 between inclusion and final evaluation. Lack of autonomy, elderly people and dementia were the main causes for refusal in the futility group (n = 57, 42 and 31 respectively).

**Conclusion**: Refusal of ICU admission occurs in 29.6% of cases. Refusal for futility is strongly but not invariably associated with deaths whereas patients considered to be too well for intensive care treatment rarely died. Strategy should be developed to create admission criteria for patients.

### F-14 Carbapenem- sparing regimens for infections caused by ESBL-producing Enterobacteriaceae in ICU patients

#### Matthieu Bosset (*speaker*)^1^, Anne Gaelle Si Larbi^1^, Solen Kerneis^2^, Julien Charpentier^2^, Mathilde Phillips Houlbracq^1^, François Parquin^1^, Jean Paul Mira^2^, Charles Cerf^1^, Rémy Gauzit^2^, Philippe Lesprit^2^

##### ^1^Hôpital Foch, Suresnes, FRANCE; ^2^Hôpital Cochin, Paris, FRANCE

###### **Correspondence:** Matthieu Bosset - matthieu.bosset@gmail.com

*Annals of Intensive Care* 2019, **9(Suppl 1)**:F-14

**Introduction**: The objectives of this study were, in ICU patients with an ESBL-producing Enterobacteriaceae infection- (i) to identify predictors of de-escalation of the empirical treatment with a carbapenem (CP) to a CP-sparing regimen (CPS) and (ii) to compare outcomes of patients de-escalated to those maintained on CP (CPM).

**Patients and methods**: Retrospective study performed in ICUs from 2 hospitals in which all CP prescriptions were reviewed within 72 h by the antimicrobial stewardship team and the ICU physicians. Demographics, clinical and therapeutic data were compared between CPS and CPM patients. Unfavorable clinical outcome (defined as mortality or relapse) was evaluated at day 60 of follow-up. Results are presented as median (IQR) or n (%).

**Results**: Ninety patients were included (54 CPM and 36 CPS), mainly treated for pneumonia (n = 35) or urinary tract infection (UTI, n = 30). Septic shock was found in 20 CPM (37%) and 19 CPS (53%), p = 0.14. The main CP-sparing antibiotics were piperacillin tazobactam (n = 11), cefoxitin (n = 11) and temocillin (n = 7). Median CP duration was 3 days (2–5]) in CPS patients. Cohort patients received a total of 1263 days of antibiotic (CP 827 days, CPS 436 days). Therefore, 35% of CP daily doses were saved. Factors associated with the use of a CPS were- older age (CPS 71 years [66–77], CPM 64 years [51–76] + p = 0.02), UTI (CPS- n = 24 (69%), including 19 male UTI + CPM- n = 6 (11%) + p < 0.001), absence of immunosuppressive therapy (CPS- n = 6 (21%), CPM- n = 17 (39%), p = 0.002) and total duration of antibiotic therapy (CPS 14 d [9–21], CPM- 9 d [7–15], p = 0.014). An unfavorable outcome was observed in 9 CPS (25%) and 22 CPM (41%), respectively (p = 0.5). Factors associated with unfavorable outcome were UTI (Hazard ratio [HR] 0.4, p = 0.045), respiratory tract infection (HR 2.76, p = 0.01) and immunosuppressive therapy (HR 3.5, p < 0.001). Use of a CPS had no effect (HR 0.6, p = 0.20).

**Conclusion**: CP de-escalation was performed in 40% of the patients, mainly those who were immunocompetent and treated for an UTI. This strategy seemed not to be associated with a worse outcome and reduced CP exposure. However, total duration of antibiotic therapy was longer with the CPS, because of the high rate of male IU treated.

### F-15 Influence of the intervention of an infectiologist on the strategy of antibiotic therapy in a intensive care unit

#### Marie Bénistand (*speaker*)^1^, Arnaud Salmon Rousseau^2^, Stéphanie Honoré^3^, Thomas Rogier^2^, Mylène Herrera^2^, René-Gilles Patrigeon^3^

##### ^1^CHU Dijon, Nîmes, FRANCE; ^2^CHU, Dijon, FRANCE; ^3^CH, Auxerre, FRANCE

###### **Correspondence:** Marie Bénistand - marie.benistand@hotmail.fr

*Annals of Intensive Care* 2019, **9(Suppl 1)**:F-15

**Introduction**: Impact of the discussion with bacteriologists and an infectiologist on antibiotic therapy in the 14-bed intensive care unit of the Auxerre hospital.

**Patients and methods**: Observational and retrospective study was conducted from 01/01/2014 to 31/12/2017. It was a before after study. First period - a weekly infectiology meeting of one hour composed of intensive care pratician, bacteriologist and pharmacist. Second period - intervention of an infectiologist. The primary outcome was the change in antibiotic consumption in daily doses per 1,000 days of hospitalization (DDD/1000 D). The secondary outcome was the change in the number of multi-resistant bacteria (BMR) isolated by weekly screenings after 72 h of hospitalization. The various proportions were compared using a Chi2 test.

**Results**: 1712 patients were hospitalized in intensive care unit (833 in 2014-15 vs 879 in 2016-17). Populations were comparable in age, sex, average length of stay, simplified seriousness index, and mortality. Antibiotic consumption differs between two periods.

**Discussion**: There is a statistically significant impact in antibiotic consumption. Fluoroquinolones and Amoxicillin-clanulanic acid which are known to induce resistance have decreased during the second period. Prescriptions of some antibiotics have improved such as Cefepime for Enterobacter, Ceftazidime and Ciprofloxacin for Pseudomonas thus saving Colimycin and Imipenem Cilastatin. There is a non-significant trend to lower multi-resistant broad-spectrum betalactamase-producing bacteria, cephalosorinases and carbapenemases in screening tests.

**Conclusion**: The intervention of an infectiologist in our service allowed an effective adaptation of the strategy of the antibiotic therapy in the hope that, in the coming years, the selection of BMR will decrease.

**Table:** 

	2014–2015	2016–2017
**TOTAL ANTIBIOTICS**	3476.64	3095.01*
**Total Beta lactam**	1881.8	1805.7*
Amoxicillin clavulanic acid	255.91	206.94
Cephalosporins	710.4	606.9*
Cefepime	38	90*
**Total Quinolones**	359.77	231.42*
Ciprofloxacine	90.91	136.02*
**Imipenem**	248.6	168.8*
**Ceftazidime**	166.5	188.1*
**Colistin**	117.4	92.74*

### F-16 Appropriateness of empiric antimicrobial therapy with imipenem colistin in severe septic patients- An observational cohort study

#### Ahlem Trifi (*speaker*)^1^, Cyrine Abdennebi^2^, Sami Abdellatif^2^, Foued Daly^2^, Yosr Touil^2^, Rochdi Nasri^2^, Salah Ben Lakhal^2^

##### ^1^Faculty of Medicine of Tunis, TUNISIA; ^2^Medical intensive care unit. La Rabta Hospital, Tunis, TUNISIA

###### **Correspondence:** Ahlem Trifi - trifiahlem2@gmail.com

*Annals of Intensive Care* 2019, **9(Suppl 1)**:F-16

**Introduction**: empiric antimicrobial therapy (EAMT) using imipenem colistin is commonly prescribed as a first line therapy in critically ill patients with severe sepsis. We aimed to assess the appropriateness of prescribing imipenem colistin as EAMT in ICU patients.

**Patients and methods**: a 3-years observational prospective study including ICU patients that required imipenem colistin as EAMT. EAMT was assessed according to microbiological and clinical outcome. Outcomes were: delay to apyrexia, delay to decrease biological inflammatory parameters (BIP), requirement for vasoactive agents, bacteriological eradication, length of stay (LOS), ventilator days and 30-day mortality.

**Results**: 79 administrations of EAMT in 70 patients were studied. EAMT was appropriate in 52% of studied cases. An ICU stay > 6 days was related to inappropriateness and chronic respiratory failure was associated to appropriateness. In the appropriate EAMT group, we showed- earlier apyrexia, shorter delay to decrease biological inflammatory parameters (BIP) and less significant vasopressors requirement (attached fig). Furthermore, EAMT improved survival with a median gain of 4 days. Inappropriate EAMT increased the mortality risk by six. Acquisition of NI in ICU was also an independent factor of mortality.

**Conclusion**: EAMT using imipenem-colistin was appropriate in half of cases and inappropriateness was associated with increased ICU mortality risk.


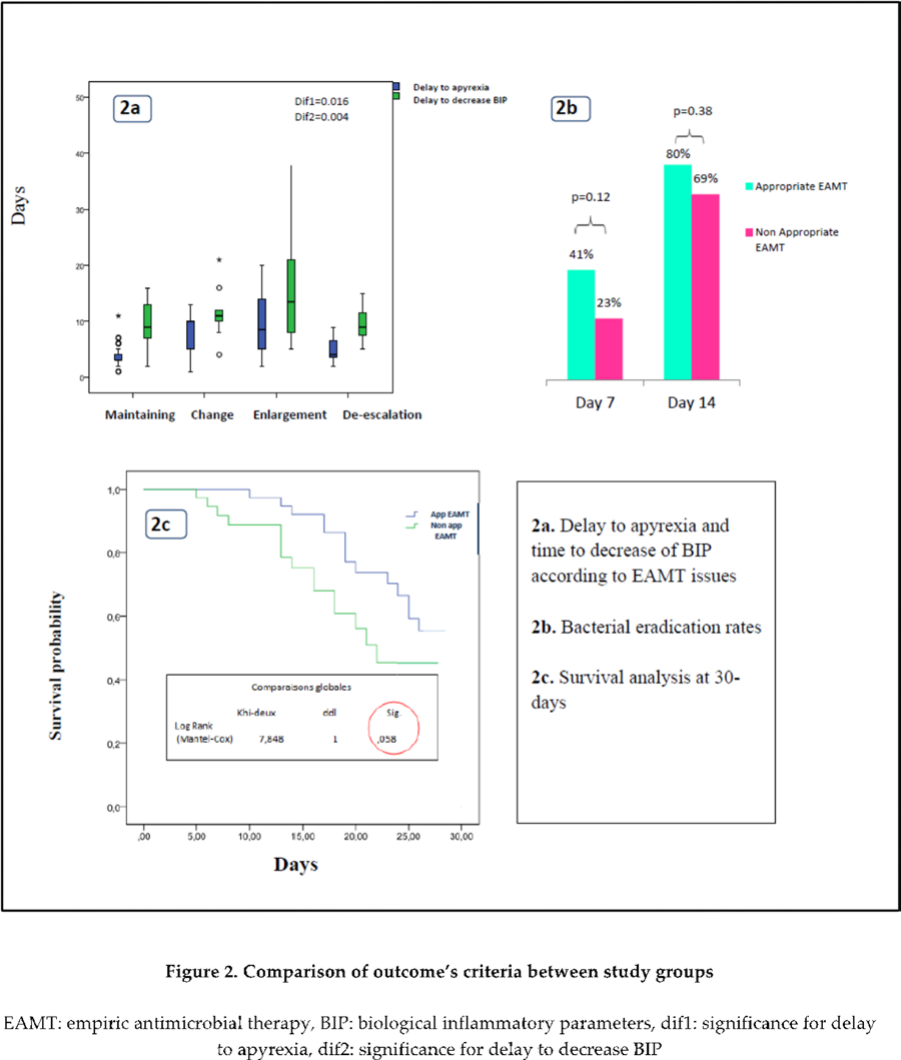


### F-17 Treatment of infections with extended spectrum beta-lactamase producing Enterobacteriaceae in ICU patients

#### Lev Volkov (*speaker*)^1^, Elisabeth Baux^2^, Sandrine Henard^2^, Bruno Levy^2^, Sebastien Gibot^1^, Pierre-Edouard Bollaert^1^

##### ^1^Réanimation médicale de l’Hôpital Central, Nancy, FRANCE; ^2^Maladies infectieuses et tropicales Hôpital Brabois, Vandoeuvre-Les-Nancy, FRANCE

###### **Correspondence:** Lev Volkov - leva.volk.tours@gmail.com

*Annals of Intensive Care* 2019, **9(Suppl 1)**:F-17

**Introduction**: The incidence of extended-spectrum beta-lactamase producing-enterobacteriaceae (ESBL-PE) is growing, with an emerging worldwide distribution. The aim of this study was to describe the outcome of patients hospitalized in an ICU with an extended spectrum beta-lactamase producing Enterobacteria (ESBL-PE) infection treated with carbapenems and alternative carbapenem-sparing therapy.

**Patients and methods**: We performed a retrospective, descriptive, monocentric study in a Public University Hospital. Patients over 18 years, hospitalized in one of the ICUs of the hospital and presenting at the admission or during the stay an infection with an ESBL-PE between January 1st 2016 and March 31st 2018 were included. Patients colonized but not infected by ESBL-PE were not included. Primary study outcome was patient’s vital status at discharge of the ICU. Secondary outcomes were vital status at discharge of the ICU in patients treated with carbapenems and in patients treated with an alternative therapy, relapse and antibiotic change after reception of the antibiotic susceptibility test results.

**Results**: In our study, 109 patients were included. Among them, 93.6% had at least one risk factor of ESBL-PE acquisition, mostly a recent hospitalization within 3 months and a use of antibiotics within 3 months. Overall, 32 patients (29.4%) died during the ICU stay. The mean SAPS II score at admission was 49.8 ± 22.1. Invasive mechanical ventilation was required for 66% of the patients. Catecholamine use was required for 64% of patients. Escherichia coli was the most frequent bacteria identified (41%), followed by Enterobacter cloacae complex (33%) and Klebsiella pneumonia (19%). Distribution of the infection sites is summarized in Figure 1. Relapse occurred in 13 (12.1%) patients. In our study 84 of 109 patients (77.1%) received an empirical antimicrobial treatment, of which 22 (26.2%) received a carbapenem and 62 (73.8%) a non-carbapenem treatment. Among the 95 patients with documented treatment, carbapenems were used in 55 (57.9%) patients and non-carbapenems in 40 (42.1%) patients. Piperacillin tazobactam was the most used non-carbapenem treatment. There was no statistically significant difference in mortality between patients treated with a carbapenem and patients treated with a non-carbapenem therapy.

**Conclusion**: Infections with ESBL-PE in critically ill patients are associated with a great mortality. Because of the sample size of this retrospective study, no difference between the groups is observed. Therefore, a larger study should be drawn.


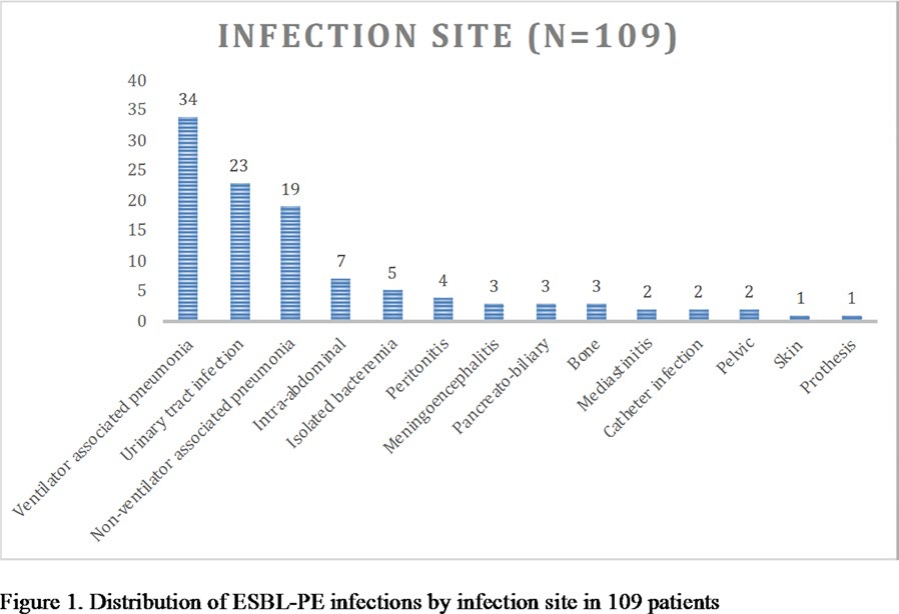


### F-18 Risk factors in intravascular catheter-related infections

#### Hayfa Fazzeni (*speaker*), Sahar Habacha, Ameni Sghaier, Ines Fathallah, Eya Seghir, Asma Mehdi, Ghada Sboui, Khaoula Ben Ismail, Emna Ennouri, Nadia Kouraichi

##### Ben Arous Regional Hospital, Intensive care Unit, Ben Arous, TUNISIA

###### **Correspondence:** Hayfa Fazzeni - fazzenihayfa@yahoo.com

*Annals of Intensive Care* 2019, **9(Suppl 1)**:F-18

**Introduction**: Catheter-related infection (CRI) is common in critically ill patients. Different factors are incriminated in developing CRI. Our study aimed to identify CRI risk factors.

**Patients and methods**: We conducted retrospective study including all patients admitted in our intensive care unit (ICU) during the period from October 2016 to August 2018. Catheter related infections were identified using the criteria of Infectious Diseases Society of America of 2009. Only patients with valid bacteriological data were included.

**Results**: During the study period, 164 patients were collected with a mean age of 55 ± 20 years and a sex ratio of 1.92. Median SOFA score was 4 [2-7] The most frequent causes of admission in ICU were acute respiratory failure (48.2%), coma (15.1%) and septic shock (7.9%). Diabetes mellitus was the main medical antecedent (34.1%). The median length of stay was 14 days [1-117]. The overall mortality rate was 29.9% (49 cases).

Among the 96 central venous catheters inserted, only twenty-two catheter related infections were diagnosed. SOFA score superior to 5 on admission was related to a higher risk of developing a CRI (OR, 1.75, 95%CI, [0.44–4.66], p = 0.033). In addition, time delay to develop a ventilator-associated pneumonia (VAP) was an independent risk factor for developing a CRI, for patients who developed a CRI median time to develop a VAP was 6 days versus 14 days for patients who did not develop a CRI (p = 0.04).

**Conclusion**: SOFA score at admission superior to five was a predictive factor of developing a catheter-related infection.

### F-19 Cannula-related infection (Ca-RI) and insertion site colonization in patients supported by peripheral veno-arterial extracorporeal membrane oxygenation (VA-ECMO) managed with a standardized dressing procedure

#### Faiza Sayagh (*speaker*)

##### Réanimation médicale, hôpital bichat, Clichy, FRANCE

###### **Correspondence:** Faiza Sayagh - f.sayagh@yahoo.fr

*Annals of Intensive Care* 2019, **9(Suppl 1)**:F-19

**Introduction**: VA-ECMO is a life support technique used in patients with cardiac failure. High rates of Ca-RI have been reported in patients supported by ECMO. Based on evidences from Ca-RI prevention, we developed a standardized procedure- maximal sterile barrier precautions, antiseptic skin preparation using with 2% chlorhexidine-70% isopropyl alcohol solution, semi-permeable transparent and highly adhesive chlorhexidine dressing. Dressings were changed 24 h after catheter insertion and then every 7 days, but leaking or soiled dressings were changed immediately.

**Patients and methods**: Study design - We retrospectively review charts of all consecutive patients who underwent VA-ECMO support for > 48 h from January 2015 to December 2017. Peripheral blood samples were collected for culturing each VA-ECMO day and the insertion site was sample at each dressing by pressing a nutritive trypticase-soy agar plate (Count-tact + Biomerieux, France) on the skin for 5 s, centering the plate on the insertion site. Evaluation criteria were cannula-related bloodstream infection (Ca-RBSI) (defined as a combination of 1 or more positive peripheral blood cultures without any other infection explaining the positive blood culture result), Ca-RI (defined as local infections signs with or without Ca-RBSI), and skin colonization defined as neither Ca-RBSI nor Ca-RI but a positive insertion-site skin culture at catheter dressing.

**Results**: Over the study period, 131 patients were supported by 133 VA-ECMO (femoro-femoral VA-ECMO- n = 47, femoro-axillary- n = 86)- age- 56.4 ± 13.8; male- 97(73.6%); SAPS II- 56.7 ± 19.3; SOFA at cannulation- 9 ± 3.7; ECMO duration- 9 ± 8.8, corresponding to 1524 ECMO-days. Primary ECMO indications were post-cardiac surgery- 32 (24.1%), myocardial infarction- 30 (22.5%), primary graft dysfunction- 26 (19.5%), cardiac arrest- 20 (15%). Ca-RBSI and Ca-RI occurred in 13 (9.8%) and 31 (23.3%) patients respectively corresponding to an incidence rate of 8.5 1000 ECMO-days and 20.3 1000ECMO-days. Colonization of insertion site was frequent and mainly related to Coagulase-negative staphylococci, however, we found no Ca-RBSI and no Ca-RI related to this microorganism. Skin colonization was more frequent for cannulas in femoral position than for cannulas in axillary position.

**Conclusion**: Cannula-related infection and insertion site colonization were frequent despite a standardized procedure including semi-permeable transparent and highly adhesive Chlorhexidine dressing in patients supported by VA-ECMO.


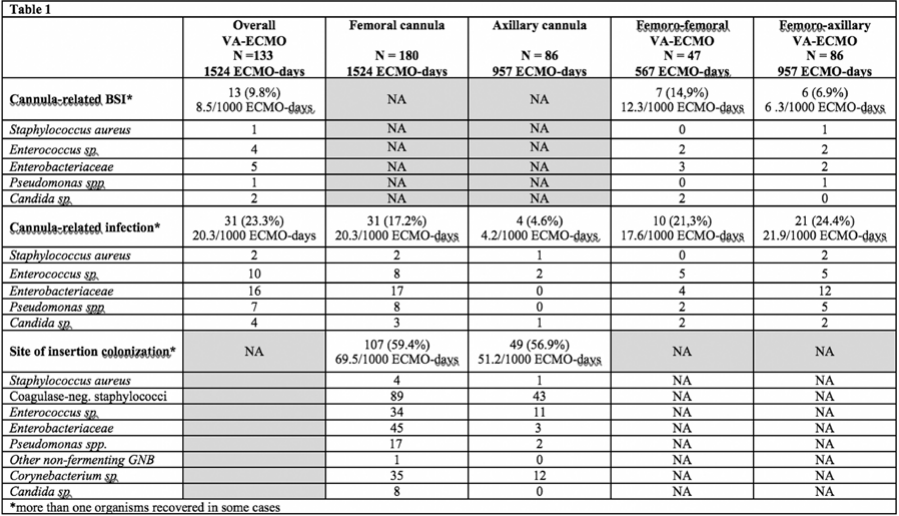


### F-20 Impact of the implementation of a multimodal and multidisciplinary care bundle for necrotizing skin and soft tissue infections

#### Tomas Urbina (*speaker*)^1^, Camille Hua^2^, Emilie Sbidian^2^, Romain Bosc^3^, Françoise Tomberli^4^, Raphael Lepeule^5^, Jean-Winoc Decousser^6^, Armand Mekontso Dessap^7^, Olivier Chosidow^2^, Nicolas De Prost^7^

##### ^1^APHP, Paris, FRANCE; ^2^Service de Dermatologie, Hôpitaux Universitaires Henri Mondor – Albert Chenevier, AP-HP, Créteil, FRANCE; ^3^Service de Chirurgie Plastique et Reconstructrice, Hôpitaux Universitaires Henri Mondor – Albert Chenevier, AP-HP, Créteil, FRANCE; ^4^Service d’anesthésie et de réanimation chirurgicale, Hôpitaux Universitaires Henri Mondor – Albert Chenevier, AP-HP, Créteil, FRANCE; ^5^Service d’immunologie clinique et maladies infectieuses, Hôpitaux Universitaires Henri Mondor – Albert Chenevier, AP-HP, Créteil, FRANCE; ^6^Service de bactériologie-virologie, Hôpitaux Universitaires Henri Mondor – Albert Chenevier, Assistance Publique – AP-HP, Créteil, Créteil; ^7^Service de Réanimation Médicale, Hôpitaux Universitaires Henri Mondor – Albert Chenevier, AP-HP, Créteil, FRANCE

###### **Correspondence:** Tomas Urbina - tomasurbina75@hotmail.com

*Annals of Intensive Care* 2019, **9(Suppl 1)**:F-20

**Introduction**: Prompt antibiotic administration and surgical debridement of infected tissues are the main modifiable prognostic factors for necrotizing skin and soft tissue infections (NSTI). A multidisciplinary and multimodal care bundle was implemented in our center in order to standardize patient management. The aim of this work was to evaluate its impact on patient recruitment, management, and outcomes.

**Patients and methods**: This retrospective cohort study included all NSTI cases admitted in our center between 2006 to 2017. A multimodal care bundle was progressively implemented through 2012 to 2013. It consisted mainly in (1) the creation of an NSTI multidisciplinary team involving intensive care and infectious diseases physicians, dermatologists available 24/7 for patient referral, plastic surgeons and microbiologists + (2) the use of a triage algorithm including a multidisciplinary bedside assessment to accelerate access to the operating room + (3) the prospective identification of all NSTI cases + (4) and an active communication policy towards the medical community about the existing bundle. We compared patients from the pre-implementation period (2006–2011) to patients from the post-implementation period (2014–2017) regarding clinical features, management and outcomes. The primary endpoint was 60-day survival.

**Results**: Overall, 224 patients were admitted during the study period (pre-implementation, n = 60; per-implementation, n = 35, and post-implementation, n = 129). The number of yearly NSTI cases increased significantly between the pre- and the post-implementation periods (median [IQR] 9 [8–13] vs 30 [24–42]); p = 0.014). There was no significant difference between these periods regarding age, comorbidities, NSTI location or potentially modifiable prognostic factors, including time to surgery (0 [0–1] vs 0 [0–1] days; p = 0.281), the proportion of patients undergoing surgery within 24 h of admission (79% vs 78%; p > 0.99), antibiotic administration within 48 h (98% vs 100% + p = 0.683) and the adequation of antibiotherapy to guidelines (93% vs 94%; p = 0.960). Inclusion during the post-implementation period was associated with an increase in 60-day survival in univariable analysis (70% vs 85%; p = 0.020 by log-rank analysis) (Figure 1). Yet, this association was not statistically significant after adjustment for patient comorbidities and severity in a multivariable Cox model (adjusted hazard ratio = 0.88; 95% CI [0.41–1.87]; p = 0.741).

**Conclusion**: The implementation of a multidisciplinary and multimodal care bundle for NSTI was associated with higher patient recruitment and better 60-day censored survival in univariable but not multivariable analysis.


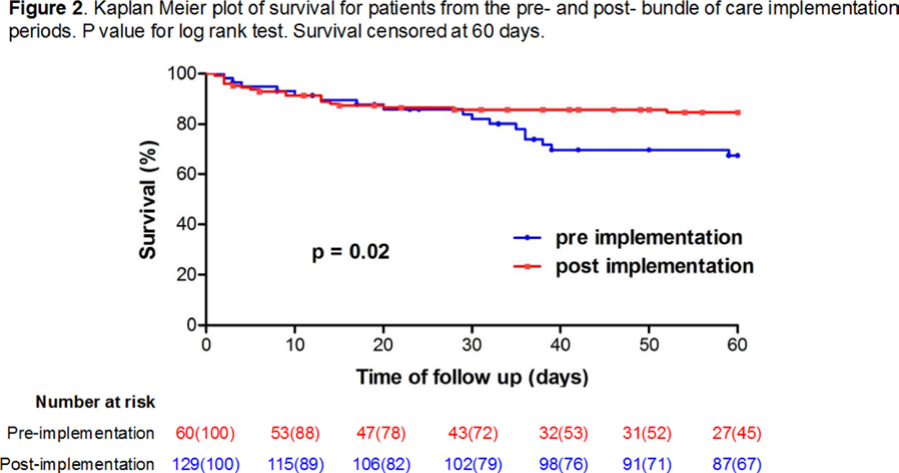


### F-21 Does prior home ventilation positively impact the outcome of ICU-conducted Non Invasive ventilation?

#### Syrine Maatouk (*speaker*), Manel Lahmar, Zeineb Hamouda, Islem Ouanes, Fahmi Dachraoui, Wiem Nouira, Fekri Abroug, Lamia Besbes

##### CHU fattouma Bourguiba Monastir, Monastir, TUNISIA

###### **Correspondence:** Syrine Maatouk - syrinemaatouk@gmail.com

*Annals of Intensive Care* 2019, **9(Suppl 1)**:F-21

**Introduction**: NIV failure is associated with increased morbi-mortality in the ICU. NIV failure prediction could help improving overall outcome by prompting earlier intubation, and paying more attention to the management of patients at risk. In addition to the type of disease leading to acute respiratory failure, technical considerations might account for NIV failure. The aim of the current study is test the hypothesis that patients who are under home ventilation do experience a lower rate of failure when ventilated non-invasively in the ICU.

**Patients and methods**: This retrospective study with prospective data collection included all patients admitted between January 2011 and December 2017 to the ICU of CHU F.Bourguiba Monastir, for acute hypercapnic respiratory failure. In included patients we collected demographic and clinical data, details of diagnosis workup, ventilatory support, and its outcome (success vs failure). Variables usually considered with impact on ventilatory outcome were compared between patients with successful NIV course and those with NIV failure. Continuous variables are presented as mean ± SD, and p < 0.05 was considered statistically significant.

**Results**: During the study period, 355 patients were admitted in the ICU for hypercapnic respiratory failure. Of these 341 (96%) had NIV as the primary mode of ventilatory support. 94/341 had home ventilation, and the main cause of decompensation overall, was cardiac dysfunction (54%). ICU-conducted NIV failed in 50 patients (14.6%) with similar rates in patients with prior home ventilation (n = 16/94, 17%) compared to patients without prior home ventilation (n = 34/247, 14%). Table 1 depicts the risk factors of NIV failure disclosed by statistical analysis.

**Conclusion**: Prior home ventilation is not associated with a lower rate of NIV failure suggesting that failure from technical reasons was not highly prevalent in our series. NIV failure risk-factors disclosed pertain to clinical severity of the decompensation and its cause.

**Table:** Table 1 Depicts the risk factors of NIV failure disclosed by statistical analysis

	**NIV Failure**	**NIV Success**
pH	7.26 ± 0.9	7.28 ± 0.4
PaCO2 (kPa)	9.9 ± 2.8	8.9 ± 2.2
E/Ea	6.6 ± 2	8 ± 2.6
ProBNP (pg/ml)	1680 ± 1462	2146 ± 1911
CRP (mg/l)	101 ± 92	83 ± 97

### F-22 Physiological study of minimally invasive ECCO2R in exacerbation of COPD requiring invasive mechanical ventilation (EPHEBE study)

#### Jean-Luc Diehl (*speaker*)^1^, Lise Piquilloud^2^, Damien Vimpere^3^, Nadia Aissaoui^3^, Emmanuel Guérot^3^, Jean-Loup Augy^3^, Marc Pierrot^4^, Delphine Hourton^3^, Armelle Arnoux^3^, Christian Richard^5^, Jordi Mancebo^6^, Alain Mercat^7^

##### ^1^Paris Descartes Faculty, Paris, FRANCE; ^2^CHU Vaudois, Lausanne, SWITZERLAND; ^3^Hopital Européen georges Pompidou, Paris, FRANCE; ^4^CH, Angers, FRANCE; ^5^Hopital de Bicètre, Le Kremlin-Bicètre, FRANCE; ^6^Hospital de la Santa Creu i Sant Pau, Barcelona, SPAIN; ^7^Angers University, Angers, FRANCE

###### **Correspondence:** Jean-Luc Diehl - jean-luc.diehl@aphp.fr

*Annals of Intensive Care* 2019, **9(Suppl 1)**:F-22

**Introduction**: Mechanical ventilation for exacerbations of COPD (AE-COPD) aims to provide adequate gas exchanges and to reduce the work of breathing (WOB). Extracorporeal CO2 removal (ECCO2R) can be a valuable additional modality for AE-COPD patients requiring invasive mechanical ventilation (IMV), by improving gas exchanges without deleterious consequences in terms of dynamic hyperinflation. However, little is known about the quantification of such ECCO2R-induced benefits.

**Patients and methods**: Open phase II-III prospective crossover (fixed order) study performed in 12 deeply sedated IMV AE-COPD patients. Dynamic hyperinflation and gas exchanges were compared without and with ECCO2R (Hemolung, Alung, Pittsburgh, USA) and adjustment of the respiratory rate (Carescape R860, GE Healthcare). The adjustment algorithm (either positive or negative) aimed to normalize arterial pH value and was based mainly on native lungs VCO2 values. When possible, WOB (Campbell’s method) with and without ECCO2R was measured at the end of the weaning process. Results are expressed in median [IQR]. Non-parametric tests were used.

**Results**: Patients (SAPS2- 33[28.5–38.5] were included in 2 centers during an 18-months period. Table indicates the main results obtained without and with ECCO2R (using the higher permitted value of sweep gas flow) and adjustments of respiratory rate. WOB measurements (Joules/min and Joules/L) were possible in 5 patients, indicating near-significant higher values when stopping the sweep gas flow for a 1 h. period- 11.7[7.5 ± 15.0] versus 22.6[13.9 ± 34.7] J/min, p = 0.0625 and 1.1[0.8 ± 1.4] versus 1.5[0.9 ± 2.8] J/L, p = 0.0625.

Three patients died in-ICU. The other patients were successfully hospital-discharged. The total duration (days) of tracheal intubation was 8 [6–18]. The duration of tracheal intubation after ECCO2R initiation was 6 [4; 16.5]. The duration of ICU stay was 14.5 [8; 22.5]. There were 4 VAP episodes, 3 hemorrhagic and 3 thrombotic complications.

**Conclusion**: Significant PaCO2, pH and SatHbO2 improvements were observed. The algorithm for pH normalization performed rather well, but without reduction in respiratory rate in the whole group and accordingly improvements in dynamic hyperinflation. This can be due to (i) mixed respiratory-metabolic acidosis in some patients and to (ii) intrinsic Hemolung properties (low to middle extracorporeal blood flow and membrane surface area). Results in terms of WOB are in line with previous publications.


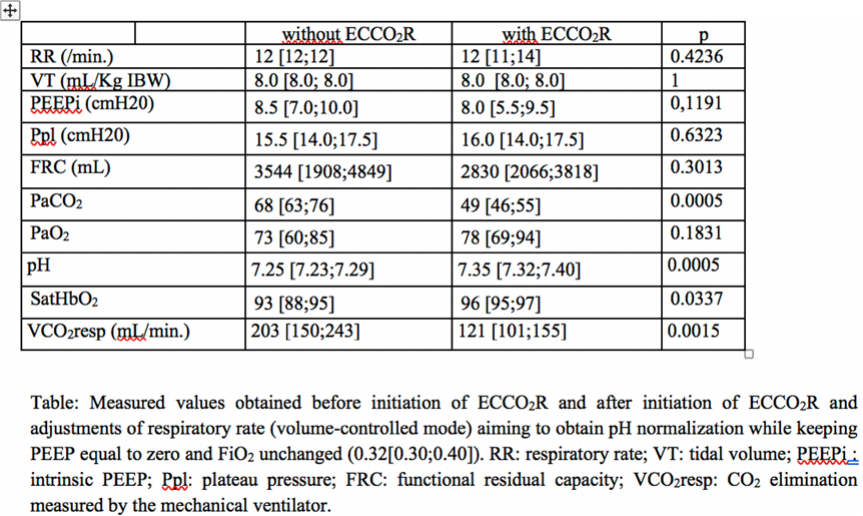


### F-23 Hyperoxia is toxic in patients with septic shock according to the Sepsis-3 criteria

#### Julien Demiselle (*speaker*)^1^, Martin Wepler^2^, Clair Hartmann^3^, Peter Radermacher^2^, Frédérique Schortgen^4^, Ferhat Meziani^5^, Mervyn Singer^6^, Valérie Seegers^1^, Pierre Asfar^1^

##### ^1^CHU, Angers, FRANCE; ^2^Institut für Anästhesiologische Pathophysiologie und Verfahrensentwicklung, Universitätsklinikum, Ulm, GERMANY; ^3^Klinik für Anästhesiologie, Abteilung Klinische Anästhesiologie, Universitätsklinikum, Ulm, GERMANY; ^4^Service de Réanimation adulte, Centre Hospitalier Intercommunal de Créteil, FRANCE; ^5^Université de Strasbourg (UNISTRA), Faculté de Médecine, Hôpitaux universitaires de Strasbourg, Service de Réanimation, Strasbourg, FRANCE; ^7^Bloomsbury Institute of Intensive Care Medicine, London, UNITED-KINGDOM

###### **Correspondence:** Julien Demiselle - jdemiselle@gmail.com

*Annals of Intensive Care* 2019, **9(Suppl 1)**:F-23

**Introduction**: The Sepsis-3 definition of septic shock includes vasopressor treatment to maintain a mean arterial pressure over 65 mmHg and a lactate concentration over 2 mmol/L. The impact of hyperoxia in patients fulfilling these criteria is unknown.

**Patients and methods**: We conducted a post hoc analysis of the HYPER2S trial, including patients requiring vasopressor therapy with an available plasma lactate value at study inclusion. We compared the effect of hyperoxia and normoxia treatment on mortality of patients with hyperlactatemia (> 2 mmol/L) and of patients requiring vasopressor for hypotension without hyperlactatemia.

**Results**: 397 patients were enrolled in this analysis, in whom 230 had lactate over 2 mmol/L and 167 had lactate lower or equal to 2 mmol/L. Among patients with lactate > 2 mmol/L, 108 and 122 were “hyperoxia”- and “normoxia”-treated, respectively. Patients with lactate > 2 mmol/L had significantly less coronary artery disease, more cirrhosis and required surgery more frequently. They also had higher illness severity (SOFA 10.6 ± 2.8 vs 9.5 ± 2.5, p = 0.0001), required more renal replacement therapy (RRT), and received vasopressor and mechanical ventilation for longer time. Mortality rate at day 28 was higher in the “hyperoxia”-treated patients with lactate > 2 mmol/L as compared to “normoxia”-treated patients (57.4% vs 44.3%, p 0.054), despite similar RRT requirements as well as vasopressor and mechanical ventilation-free days. A multivariate analysis showed an independent association between hyperoxia and mortality at day 28 and 90. In patients with lactate ≤ 2 mmol/L, hyperoxia had no effect on mortality, nor on other outcomes.

**Conclusion**: This study suggests that hyperoxia may be associated with a higher mortality rate in patients with septic shock using the Sepsis-3 criteria, but not in patients with hypotension requiring vasopressor without hyperlactatemia.

### F-24 Acute respiratory failure in obesity-hypoventilation syndrome managed in the intensive care unit

#### Nader Chebib (*speaker*)^1^, Pascale Nesme^2^, Nathalie Freymond^3^, Laurent Argaud^4^, Thomas Rimmele^4^, Julien Bohe^4^, Gilles Devouassoux^2^, Pierre Jean Souquet^3^, Claude Guerin^1^

##### ^1^Réanimation Médicale Hôpital de la Croix Rousse, Hospices Civils, Lyon, FRANCE; ^2^Pneumologie Hôpital de la Croix Rousse, Hospices Civils de Lyon, FRANCE; ^3^Pneumologie Centre Hospitalier Lyon Sud, Hospices Civils de Lyon Pierre-Bénite, Lyon, FRANCE; ^4^Réanimation Médicale Hôpital Edouard Herriot, Hospices Civils de Lyon Pierre-Bénite, Lyon, FRANCE

###### **Correspondence:** Nader Chebib - nader.chebib@chu-lyon.fr

*Annals of Intensive Care* 2019, **9(Suppl 1)**:F-24

**Introduction**: Obesity-hypoventilation syndrome (OHS) is an increasing cause of acute hypercapnic respiratory failure (AHRF) in intensive care unit (ICU). Our objective was to describe the epidemiology, the ventilatory management and the outcome of patients with OHS admitted to the ICU for AHRF.

**Patients and methods**: We retrospectively built a cohort of OHS patients staying in 2 pneumology wards and admitted for AHRF in the 4 ICUs taking part of the tertiary University teaching hospital in Lyon, France, between January 1st 2013 and September 30th 2017. Clinical, functional and biological characteristics of patients at the time of OHS diagnosis were used as covariates. The main end-point was the rate of success of noninvasive ventilation (NIV) in the ICU. The secondary end-points were patient survival from OHS diagnosis to last follow-up, risk factors for ICU admission and for long-term survival including the role of ICU stay. Data were expressed as median (1st-3rd quartiles) and counts (percentage) and compared with nonparametric tests. Survival was measured by the Kaplan–Meier method and the difference in restricted mean survival time (RMST). Multivariate logistic regression analysis was used to assess risk factors of ICU admission. Cox proportional hazard model and multistate model were used to determine the risk factors of long-term survival.

**Results**: One-hundred and fifteen prevalent patients with OHS were included. Over a median follow-up period of 3.5 (1.4–6) years, 37 patients (32.1%) were admitted to the ICU for AHRF. The most frequent cause of AHRF was congestive heart failure (54%). Fourteen patients (37.8%) were treated for OHS with continuous or bilevel positive airway pressure prior to ICU admission. NIV was used as a first-line ventilatory support in 36 patients (97.2%) and was successful in 33 patients (89.2%). ICU mortality was low (2.7%). Patients admitted to ICU had significantly higher age, lower forced expiratory volume in 1 s and lower vital capacity (VC) at the time of OHS diagnosis. Difference in RMST was significant with a gain in survival of 663 days for patients not admitted to ICU. Multivariate analysis showed that lower VC at OHS diagnosis was significantly associated with higher risk of ICU admission. No factor was independently associated with long-term overall mortality in multivariate analysis.

**Conclusion**: AHRF is frequent in OHS diagnosis and is generally responsive to NIV. Lower VC is associated with a higher risk of ICU admission.

**Table:** Table 1. Clinical, biological and treatment characteristics of patients with OHS admitted to the ICU

	OHS in ICU
Age at ICU admission (years)	72 (66–79)
ABG at ICU admission	
pH	7.06 (7.22–7.31)
PaCO2 (mmHg)	70 (61–76)
PaO2 (mmHg)	79 (66–83)
Bicarbonate (mmol/L)	30 (26–35)
Lactate (mmol/L)	1.2 (0.8–1.7)
Vital signs	
MAP (mm Hg)	81 (66–95)
HR (beats per minute)	91 (80–95)
RR (cycles per minute)	25 (20–30)
Glasgow coma score	13 (13–15)
IGSII score	36 (23–49)
AHRF cause n (%)	
Congestive heart failure	20 (54)
OHS	11 (29.7)
Pulmonary embolism	4 (10.8)
Pneumonia	1 (2.7)
Neoplastic pleural effusion	1 (2.7)
Prior OHS diagnosis n (%)	14 (37.8)
Delay OHS diagnosis-ICU admission (months)	11.5 (− 2.4 to 25)
Treatment	
NIV first-line n (%)	36 (97.2)
Invasive ventilation first-line n (%)	1 (2.7)
Invasive ventilation after NIV failure n (%)	3 (8.1)
Length invasive ventilation (days)	4.4 (1–7)
Length NIV (days)	6 (3–8)
Time to correct acidosis with NIV (days)	2.9 (1–3)
NIV at ICU discharge n (%)	23 (62.1)
Renal dialysis n (%)	3 (8.1)
Vasopressor agents n (%)	8 (21.6)
Length of stay (days)	
ICU	7.2 (4–8)
Post-ICU	14.2 (10–16.5)
Death in ICU n (%)	1 (2.7)

Quantitative values are expressed as median with first and third quartile; MAP: mean arterial pressure; HR: heart rate; RR: respiratory rate; IGSII: simplified severity index II; NIV: non invasive ventilation

### F-25 Critical Illness-Related Corticosteroid Insufficiency during difficult weaning from mechanical ventilation

#### François Bagate (*speaker*), Alexandre Bedet, Françoise Tomberli, Keyvan Razazi, Nicolas De Prost, Guillaume Carteaux, Armand Mekontso Dessap

##### CHU Henri Mondor, Créteil, FRANCE

###### **Correspondence:** François Bagate - francois.bagate@aphp.fr

*Annals of Intensive Care* 2019, **9(Suppl 1)**:F-25

**Introduction**: Critical illness-related corticosteroid insufficiency (CIRCI) is common during critical illness and is usually associated with poor outcomes, as prolonged duration of mechanical ventilation (MV) and higher mortality. CIRCI may alter cardiac and vascular functions. Weaning-induced pulmonary oedema (WiPO) is a major mechanism of weaning failure. The aim of this study was to evaluate the role of CIRCI in patients with difficult ventilator weaning and its possible relation with WiPO.

**Patients and methods**: Prospective study conducted in the intensive care of a university hospital in France. Patients under MV for more than 24 h, meeting weaning criteria and having failed the first spontaneous breathing trial (SBT) underwent a corticotropin stimulation test, with assessment of total blood cortisol levels immediately before (T0) 0.25 mg iv of tetracosactrin and 30 and 60 min afterward. Δmax was defined as the difference between the maximal value after the test and T0. CIRCI was defined as T0 < 10 μg/dL (276 nmol/L) and or Δmax < 9 μg/dL (248 nmol/L) and inadequate adrenal reserve as Δmax < 9 μg/dL. Biomarkers (natriuretic peptide and protidemia) sampling and echocardiograms were performed during the second SBT and were used to diagnose WiPO, which was defined according to two definitions (one liberal and one conservative) derived from recent publications on the topic. Successful extubation was defined as patient alive without reintubation 7 days after extubation.

**Results**: Seventy-six consecutive patients (63 ± 14 years; 49 men) with difficult weaning were enrolled. CIRCI and inadequate adrenal reserve occurred in 25 (33%) and 17 (22%) patients, respectively. The probability of successful extubation (alive was significantly decreased in patients with CIRCI or inadequate adrenal reserve (Figure 1A and 1B), as compared to their counterparts, and this association persisted after adjustment on severity (SOFA score at first SBT). WiPO occurred in 44 (58%) and 8 (11%) patients, according to the liberal and conservative definition, respectively. WiPO was not associated with CIRCI or with inadequate adrenal reserve, and did not influence weaning outcome, whatever the definition used.

**Conclusion**: CIRCI was common during difficult weaning and was associated with its prolongation. We did not find a significant association between CIRCI and WiPO.


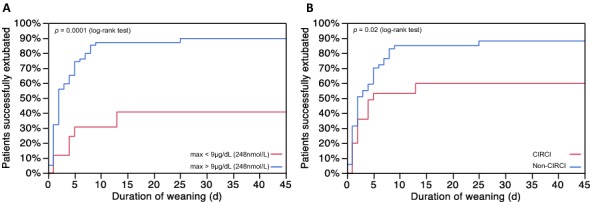


### F-26 Bedside predictors of successful weaning from high-flow nasal cannula in ICU

#### Rémi Coudroy (*speaker*), Maeva Rodriguez, René Robert, Jean-Pierre Frat, Arnaud W. Thille

##### Service de Médecine Intensive et Réanimation, CHU, Poitiers, FRANCE

###### **Correspondence:** Rémi Coudroy - r.coudroy@yahoo.fr

*Annals of Intensive Care* 2019, **9(Suppl 1)**:F-26

**Introduction**: High-flow nasal cannula oxygen therapy (HFNC) is a recent technique that can provide high FiO2, decrease the work of breathing, and recruit lungs. Despite promising clinical results, its weaning has never been investigated. Our objective is to describe factors associated with successful weaning of HFNC.

**Patients and methods**: In a retrospective monocenter study over a 2-year period, all patients admitted to ICU and treated with HFNC for acute respiratory failure were included. Patients who were never weaned from HFNC during ICU stay, those who were treated with HFNC associated to NIV, and those who received preventive post-extubation HFNC were excluded. Our primary outcome was to compare patients according to the outcome of the first HFNC weaning attempt. Our secondary outcome was to compare the evolution of patients who failed HFNC weaning at first attempt, and then who were successfully weaned from HFNC.

**Results**: From the 190 patients included, 168 (88%) were successfully weaned from HFNC at the first attempt. At time of the first attempt, those who were successfully weaned had lower FiO2 (39 vs. 48%, p = 0.02), were more likely to have a SpO2/FiO2 > 235 (corresponding to a PaO2/FiO2 > 200 mm Hg) (74% vs. 41%, p = 0.005), and had a higher ROx index (SpO2/FiO2/RR) than those who failed (p = 0.002). The 19 patients who failed the first attempt significantly increased the ROx index at time of successful weaning (p = 0.04).

**Conclusion**: If confirmed in further studies, SpO2/FiO2 and ROx index could be 2 useful noninvasive predictors of success of HFNC weaning available at bedside.

### F-27 Is immunosuppression status a risk factor for noninvasive ventilation failure in acute hypoxemic respiratory failure?

#### Rémi Coudroy (*speaker*)^1^, Tài O. Pham^2^, René Robert^1^, Jean-Pierre Frat^1^, Arnaud W. Thille^1^

##### ^1^Service de Médecine Intensive et Réanimation, CHU de Poitiers, Poitiers, FRANCE; ^2^Interdepartmental Division of Critical Care Medicine, Toronto, CANADA

###### **Correspondence:** Rémi Coudroy - r.coudroy@yahoo.fr

*Annals of Intensive Care* 2019, **9(Suppl 1)**:F-27

**Introduction**: Recent European American clinical practice guidelines recommend first-line noninvasive ventilation (NIV) to manage acute hypoxemic respiratory failure in immunocompromised patients. In contrast, in non-immunocompromised patients, the experts were unable to offer a recommendation given the uncertainty of evidence. Immunocompromised patients have particularly high mortality rates when they require invasive ventilation. However, it is not clear whether immunosuppression status is a risk factor for NIV failure. We aimed to assess the influence of immunosuppression status on outcomes of patients treated with NIV for acute respiratory failure.

**Patients and methods**: We performed a post hoc analysis pooling 2 prospective studies on acute hypoxemic respiratory failure. Patients treated by NIV were included. Those with cardiogenic pulmonary edema, acute-on-chronic respiratory failure or hypercapnia were excluded.

**Results**: Among the 208 patients analyzed, 71 (34%) were immunocompromised whose main reason was cancer (n = 37, 52%). Immunocompromised patients had higher severity scores upon ICU admission, were more likely to have bilateral lung infiltrates and received higher pressure-support levels under NIV than non-immunocompromised patients. Intubation and in-ICU mortality rates were higher in immunocompromised patients than in the others- 61% (43 out of 71 patients) vs. 43% (p = 0.02), and 38% (27 patients) vs. 15% (p < 0.001), respectively. Using multivariate analysis, immunosuppression was independently associated to intubation with an adjusted OR of 2.18 (95% CI 1.18–4.14, p = 0.01) and to ICU mortality (OR 3.44, 95%CI 1.73–7.03, p < 0.001).

**Conclusion**: Immunosuppression status influences outcomes in patients with acute hypoxemic respiratory failure treated with NIV. Studies in this specific population are mandatory.

### F-28 Diagnostic tools used and pathogens involved in Ventilatory Acquired Pneumonia in PICU: a one-year prospective multicenter database (the INCIPAVE study)

#### Stephane Dauger (*speaker*)^1^, Yves Gallien^2^, Marcel Tinnevelt^3^, Maryline Chomton^1^, Alexandra Binoche^5^, Olivier Brissaud^6^, Capucine Didier ^7^, Pierre-Louis Leger^8^, Sonia Pelluau^9^, Laure De Saint-Blanquat^10^, Jérôme Naudin^1^, Matthieu Resche-Rigon^12^

##### ^1^Réanimation pédiatrique, hôpital Robert Debré, Paris, FRANCE; ^2^Service de Biostatistique et Information Médicale, Paris, FRANCE; ^3^PICU, Utrecht, FRANCE; ^5^Réanimation pédiatrique, Hôpital Jeanne de Flandres, Lille, FRANCE; ^6^Réanimation pédiatrique, Hôpital Pellegrin, Bordeaux, FRANCE; ^7^Réanimation pédiatrique, Lyon, FRANCE; ^8^Réanimation pédiatrique, Hôpital Armand Trousseau, Paris, FRANCE; ^9^Réanimation pédiatrique, Hôpital des enfants, Toulouse, FRANCE; ^10^Réanimation pédiatrique, Hôpital Necker enfants malades, Paris, FRANCE; ^12^Service de Biostatistique et Information Médicale, Hôpital Saint-Louis, Paris, FRANCE

###### **Correspondence:** Stephane Dauger - stephane.dauger@aphp.fr

*Annals of Intensive Care* 2019, **9(Suppl 1)**:F-28

**Introduction**: Ventilatory Acquired Pneumonia (VAP) is one of the main nosocomial infection in adult ICU. To date, only one prospective multicenter study performed during six months in 16 PICUs of the US has prospectively described pediatric VAP. We design the INCIPAVE study to report the occurrence of VAP in european PICUs. One of the aim of the INCIPAVE study was to precisely describe the diagnostic tools used and the pathogens involved in pediatric VAP.

**Patients and methods**: Multicenter prospective cohort study from 03/04/2017 to 03/04/2018 including all patients mechanically ventilated (MV) at least once in eight PICUs, one in the Netherlands and seven in France. VAP was defined using the 2015 CDC criteria, applied during PICU stays, excluding the 48 h preceeding and following PICU. Patients were described on admission and main risk factors ever tested in the medical litterature were daily included by a pediatric intensivist of each PICU in an electronic database on a securized dedicated website. The Ethics Committee of the French Society of Intensive Care approved the study, which has been declared to the CNIL and recorded on Clinical-Trials. All parents or legal representatives were individually informed by a dedicated sheet. Descriptive data are reported as number (%) or medians [first-third quartiles].

**Results**: These results are based on declarative information reported in the INCIPAVE database during its first opening on September 2018, before cleaning. A total of 158 VAP (76.8% according to CDC criteria) was declared during 11685 days of MV. At least one pathogen was identified in 58.7% case of VAP, with more than one in 11.6%. Diagnosis was made on Tracheal Aspirates, with or without quantitative cultures (51% and 12.9% respectively), and on Blind Protecting Specimen Brush with or without quantitative cultures (7.7% and 2.6% respectively). Identified pathogens were Pseudomonas aeruginosas (PA, 12.1%), Haemophilus influenzae (HI, 12.1%), Staphylococcus aureus (SA, 7.4%), Streptococcus pneumoniae (SP, 5.4%), Stenotrophomonas maltophilia (SM, 3.4%). HI and SP were involved in “early VAPs” (< 6 days) and PA, SM and SA were reported in “late onset” VAPs.

**Conclusion**: Tracheal aspirate with quantitative culture is still the method of choice to diagnose VAP in PICU. Nosocomial pathogens are mainly involved after 6 days of MV. A more precise analysis is planned after cleaning of the database, including a special reading of each case of VAP.

### F-29 Management of pleural infection in children at a University Hospital: A retrospective study over 6 **years**

#### Ombeline Roignot (*speaker*), Anne-Sophie Guilbert, Charlie De Melo, Amélie Stern

##### CHU de Strasbourg - Hôpital de Hautepierre, Strasbourg, FRANCE

###### **Correspondence:** Ombeline Roignot - ombeline.roignot@chru-strasbourg.fr

*Annals of Intensive Care* 2019, **9(Suppl 1)**:F-29

**Introduction**: Parapneumonic effusions and pleural empyema are common complications of community-acquired bacterial pneumonia in children. There is not yet a consensus concerning the management of this pathology, and expert’s recommendations are scarce. The objective of this study was to compare whether the evolution of our patients benefited or not from drainage procedures.

**Patients and methods**: This retrospective single-center study collected data from 79 children treated for pleural infection at the Strasbourg University Hospital from May 2010 to May 2016. 2 groups of children were compared concenring medical and or invasive treatment received (thoracentesis, chest drain insertion with or without instillation of fibrinolytic agents and surgical techniques).

**Results**: 43 children benefited from an invasive treatment strategy (IT), and 36 from antibiotics alone (AA). The epidemiological data of the 2 groups was comparable (comorbidities, vaccination status). Significant differences were the duration of oxygen dependence- 3.1 days in the AA group versus 7.6 days in the IT group (p < 0.001), the time elapsed to obtaining apyrexia- 4 days in the AA group versus 7.9 days in the IT group (p = 0.009), overall hospital stay and duration of intensive care. Initial C-reactive Protein levels were measured at 160.5 mg/L in the AA group versus 258 mg/L in the IT group (p < 0.001). We proposed a predictive score for an invasive procedure based on initial CRP and pleural ultrasound data.

**Conclusion**: Pneumonia in children with low to moderate volume effusion may show a favorable clinical course treated by antibiotics only. Prospective randomized controlled trials are needed to reassess the indication of invasive procedures in the management of children’s pleural infection.

### F-30 Mechanical ventilation under pediatric V–V ECMO

#### Jerome Rambaud (*speaker*), Julien Jegard, Isabelle Guellec, Pierre Louis Leger, Sandrine Jean, Yohan Soreze, Jean Eude Piloquet, Julia Guilbert, Cecile Valentin, Alexandra Bower

##### Armand-Trousseau, pediatric intensive care, Paris, FRANCE

###### **Correspondence:** Jerome Rambaud - jerome.rambaud@aphp.fr

*Annals of Intensive Care* 2019, **9(Suppl 1)**:F-30

**Introduction**: Protective mechanical ventilation and adjuvant therapies for severe ARDS are well-defined in adult and pediatric population. However, no clearly identified recommendations are available to perform a protective ventilation during pediatric V–V ECMO. The aim of this study was first to describe potential associations between ventilatory settings during ECMO and outcome in ARDS patients. The secondary goal was to compare three periods of interest to identified significant modification and their potential consequences on the survival rate.

**Patients and methods**: We performed an observational monocentric retrospective study, from January 2007 to December 2017. All patients treated by ECMO V–V for a refractory ARDS were included. We collected data’s at day 1, day 3 day 7 and day 14 of ECMO. Three periods of interest were defined (before 2010, from 2010 to 2014 and after 2014). We retrospectively collected the data’s from our local database, approved by the French Data Protection Authority.

**Results**: 83 patients treated by extracorporeal membrane oxygenation were included. We identified an increase of the number of ECMO V–V for pediatric refractory ARDS associated with a higher survival rate throughout the three periods. The OSI (oxygenation saturation index) was the only pre-ECMO parameter significantly associated with a higher mortality.

We identified a significant modification the adjuvant therapy illustrated by a sharpe increase for the use of neuromuscular blockers (from 14% to 52%) and the prone positioning before ECMO (from 5% to 85%). We also show evidence of a strong modification of the ventilatory parameters during ECMO. As example, the tidal volumes are significantly lower throughout the periods (5 cc/kg vs 3.5 cc/kg) such as the driving pressure (28 vs 14 cm of H2O). In contrary, the PEEP is higher in the most recent period. Finally, we identified an improvement of the survival rate all over the three period.

**Conclusion**: Recent modifications of ventilatory parameters during V–V ECMO for pediatric ARDS aimed at implementing a better lung protection. These modifications are associated with a better survival. However, the correlation between survival and ventilators settings remained unclear and a multicentric study should help physician to identify prognosis factors.

### F-31 Septic shock and toxic shock syndrome- two infectious shocks with different immune response

#### Solenn Remy (*speaker*)^1^, Karine Kolev-Descamps^2^, Morgane Gossez^3^, Fleur Cour-Andlauer^2^, Fabienne Vene^3^, Tiphanie Ginhoux^6^, Guillaume Monneret^3^, Etienne Javouhey^2^

##### ^1^Hospices Civils de Lyon, Lyon, FRANCE; ^2^Réanimation Pédiatrique - Hôpital Femme Mère Enfant, Lyon, FRANCE; ^3^Laboratoire d’Immunologie cellulaire - Hôpital Edouard Herriot, Lyon, FRANCE; ^6^Centre D’Epidémiologie Clinique - EPICIME, Lyon, FRANCE

###### **Correspondence:** Solenn Remy - solene.remy@chu-lyon.fr

*Annals of Intensive Care* 2019, **9(Suppl 1)**:F-31

**Introduction**: Septic shock (SS) has recently been redefined in adults as life-threatening organ dysfunction caused by dysregulated host response to infection. Due to pediatric specificities, adult definition cannot be just transpose to children. Toxic shock syndrome (TSS) is a particular entity of infectious shock, with large pediatric prevalence. Some toxins specific to Streptococcus A and Staphylococcus aureus lead to superantigenic activation of T-lymphocytes, responsible for major cytokines storm with multi-organ failure. While immunosuppression induced by SS is now demonstrated in adults and children, we investigated whether similar immune disorders arise during TSS.

**Patients and methods**: Single-center prospective study included all children under 18 years-old, consecutively admitted into Pediatric Intensive Care Unit for SS (“Surviving Sepsis Campaign” Goldstein criteria), or TSS (Center for Disease Control), between September 2014 and July 2018. Controls were recruited from outpatients admitted for an elective benign surgery, without any criteria of infection. Immune monitoring realized by flow cytometry included HLA-DR expression on monocytes (mHLA-DR), total lymphocyte count, and lymphocyte sub-populations’ proportions (CD4* and CD8* T cells, regulatory T cells, NK cells, B cells). Samples were analyzed at Day 1, 3 and 7, after shock onset. Clinical data were collected prospectively, as well as severity scores and secondary nosocomial infection occurrence.

**Results**: Forty-six SS, 12 TSS and 30 controls were recruited. At each time points, mHLA-DR in SS and TSS groups were decreased, compared with controls (fig 1). Moreover, mHLA-DR was significantly higher in TSS at day 1 and 3, than SS. Lymphocytes’ time course also differs between SS and TSS- more profound lymphopenia occurred at day 1 in TSS than SS, but correction was faster in TSS between day 1 and 3, while between day 3 and 7 in SS. No difference was observed concerning regulatory T cells. Thirteen patients with SS presented secondary infections (28%), compared to only one in TSS group (8%).

**Conclusion**: Our study showed that despite similar initial shock, immune response is significantly different between SS and TSS. TSS didn’t induce persistent immune-suppression, as seen in SS- highlighted by different time course of mHLA-DR, lymphocytes and secondary infection occurrence. There is no only one type of septic shock but different infectious shocks with different immune responses and clinical outcomes. These results reinforce objective to better characterize immune state of patients in intensive care, in order to propose personalized medicine with adapted immune-modulatory therapies.


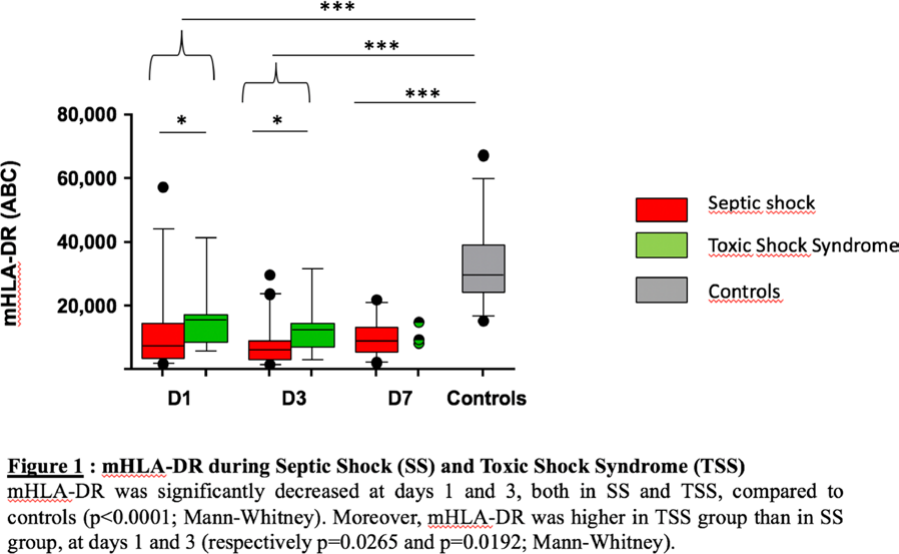


### F-32 The Septic Shock Score in children in septic shock treated with extracorporeal assistance

#### Clémence Marais (*speaker*)

##### APHP, Paris, FRANCE

###### **Correspondence:** Clémence Marais - clemence.marais@aphp.fr

*Annals of Intensive Care* 2019, **9(Suppl 1)**:F-32

**Introduction**: Septic shock is a common pathology in intensive care units responsible for a high mortality rate. Extracorporeal life support (ECLS) is used when patients no longer respond to standard treatments, including inotropes. Recently, in a multicenter study involving more than 500 children with septic shock, the Septic Shock Score (SSS) proved highly reliable in identifying patients at risk of death and was able to define refractory septic shock. The objective of our study is to evaluate two versions of the SSS, the bedside SSS (bSSS) and the computed (cSSS) in a group of patients hospitalized with septic shock who received ECLS support.

**Patients and methods**: This retrospective study includes patients aged 1 month to 18 years hospitalized in the intensive care units of the Necker Enfant Malade, Trousseau and Bicêtre hospitals for septic shock requiring ECLS assistance between January 2010 and March 2018. Five data collection times were chosen- sepsis time, ECLS decision time, ECLS starting time and end of hospitalization time. At the first 4 collections, clinical and biological criteria were collected to calculate the different predictive scores of septic shock. The group of deceased patients and the group of living patients were compared at these different times.

**Results**: 38 patients were included in our study, 24 of which died during the hospitalization. At all times studied, both the bSSS and the cSSS had poor reliability in identifying deceased children. The vasoactive-inotropic score is significantly higher at ECLS starting time in deceased patients and it decreases between ECLS decision time and ECLS starting time in living patients.

**Conclusion**: This is a pilot study that tested a database of children in septic shock who had received ECLS in APHP’s intensive care units. The continuation of the study will be carried out as part of a collaborative project of the European Society of Pediatric and Neonatal Intensive Care (ESPNIC), and will extend to 27 other pediatric intensive care units to clarify ECLS success criteria, characterize the evolution of organ failure and thus better define ECLS uses in children with septic shock.

### F-33 Prediction of Complicated Outcomes in children with sickle cell anemia: a CARABDREPA Cohort

#### Amélie Rolle (*speaker*)^1^, Raphael Blanc^2^, Jérôme Pignol^2^, Frédéric Martino^1^, Pascale Piednoir^1^, Bertrand Pons^1^, Hossein Mehdaoui^1^, Michel Carles^1^

##### ^1^CHUG, Pointe-à-Pitre, FRANCE; ^2^CHUM, Fort-De-France, FRANCE

###### **Correspondence:** Amélie Rolle - melie9712@hotmail.com

*Annals of Intensive Care* 2019, **9(Suppl 1)**:F-33

**Introduction**: Sickle cell disease (SCD) is an increasing global health problem + approximately 300,000 infants born every year. SCD is associated with a decreased life expectancy, half of the deaths occurring in the ICU. Preexisting prediction model built by The Cooperative Study of SCD do not show relationship existed between early clinical predictors and complicated outcome (CO). The identification of risk factors could potentially have an immediate effect in preventing clinical complications and improving the quality of life for hundreds of thousands of children worldwide. We aimed to identify early predictors of a CO, defined as an ICU stay > 2 days, the need for vital support or death in children with SCD.

**Patients and methods**: Retrospective observational cohort study of SCD patients over a 5-year period were conduced in French territories in the Americas teaching hospital and SCD referral center.

**Results**: Of the 2559 infant’s admissions in the Carabdrepa cohort, 174 (6.8%) had a CO, of whom 6 (0.2%) death. Using multivariate analysis, we found significant predictors of CO- an episode of dactylitis (defined as pain and tenderness in the hands or feet) before the age of one year (OR 3, IC 95% 1.9–45.5), a hemoglobin level of less than 7 g per deciliter (OR 1.75, IC 95% IC 0.98–2.84), and leukocytosis (OR 1.21, IC 95% 0.8–1.83), a respiratory rate more than or equal to 32 cycles min (OR 1.01, IC 95% 0.84–1.18).), a Delay between first symptom and medical contact (OR 1.3, IC 95% 1.04–1.65), and an admission for sepsis (OR 1.32, IC 95% 0.44–3.85). Our model demonstrated good predictive performances in terms of discrimination (c-statistic- 0.813) and calibration.

**Conclusion**: Sickle-cell disease children are at high risk of life threatening complications. Episodes of dactylitis, with a sustained drop of hemoglobin + associated with a sepsis context and a delay in medical care are strong predictors of a complicated outcome.

### F-34 Hemorrhagic shock in multiple trauma children- epidemiological aspects and application of the TRISS methodology in this population

#### Luis Ferreira (*speaker*)^1^, Gilles Orliaguet^2^, Caroline Duracher-Gout^2^, Stephane Blanot^2^, Estelle Vergnaud^2^, Thomas Baugnon^2^, Philippe Meyer^2^

##### ^1^Necker enfant malade, Ivry Sur Seine, FRANCE; ^2^APHP- Hopital Necker, Paris, FRANCE

###### **Correspondence:** Luis Ferreira - ferreiralfb@gmail.com

*Annals of Intensive Care* 2019, **9(Suppl 1)**:F-34

**Introduction**: Traumatology is the leading cause of death in young adults and children. Hemorrhagic shock is a major aggravating factor in trauma, however there are very few data available in the pediatric population. TRISS method [1] offers a standard approach for evaluating outcome of trauma care, enabling the determination of an individual probability of survival (Ps) for each patient according to Trauma and Injury Severity Score. The aims of our study were- 1) to identify multiple trauma children admitted with hemorrhagic shock in our pediatric Trauma Center, 2) to analyze factors that could influence the outcome using the TRISS method.

**Patients and methods**: We performed a monocentric, observational, descriptive, retrospective study on medical records. The included patients were children under 18 years of age admitted for multiple trauma and presenting with hemorrhagic shock upon arrival. The patients included were identified by means of a computerized database internal to the service. The primary outcome was death.

**Results**: From January 2014 to April 2018, 947 multiple trauma children were admitted in the service. Among them, 41 (4.3%) were in hemorrhagic shock upon admission and included in the study. The median interquartile age and weight were 3.0 [2.0–10.7] years and 16.0 [12.9–32.5] kg, respectively. Their trauma profile was very similar to other polytraumatized children, with 73% of head trauma. The global principles of Damage Control Resuscitation have been met, including the application of the massive transfusion protocol. However, only 10% of children actually correspond to the usual definition of “massive blood transfusion” (70 ml kg over 24 h) and only 37% required a surgical hemostasis procedure. The overall mortality was 41% with an average TRISS of 45%. We had 2 “unexpected” survivors and 1 “unexpected” death according to the TRISS method in our population.

**Conclusion**: In our study, 4.3% of the multiple trauma children were admitted in hemorrhagic shock in our center. These children were heavily traumatized with an overall mortality rate of 41%. However, the TRISS method revealed an observed mortality 3.9% lower than the predicted mortality, with +2.4% of “excess survivors”. When we checked the medical records of the 2 “unexpected” survivors, they seemed to have beneficiated from a more aggressive prehospital resuscitation care.

**Reference**

1. Boyd CR, Tolson MA, Copes WS (1987) Evaluating trauma care- the TRISS method. Trauma Score and the Injury Severity Score. (J Trauma 27- 370-378).

**Table:** 

	Entire population (n = 41)	«Unexpected» survivors (n = 2)
Predicted death probability by TRISS	37.4 [10.3-81.5]	73.2 ; 86.7
Mean TRISS death probability (%)	44.9	80.0
Prehospital major events and treatment		
Cardiac arrest before emergency teams arrived	8 (19%)	0
Cardiac arrest during transport	4 (10%)	0
Received Intravenous fluids (ml kg^−1^)	35 (85%) / 20.0 [10.5-33.0]	2 (100%) / 33.0 ; 50.0
Received Blood transfusion (ml kg^−1^)	3 (7%) / 13.0 [8.0-15.7]	0
Blood transfusion and plasma expanders	1 (2%)	0
Received Tranexamic Acid (ml kg^−1^)	4 (10%) / 12.0 [10.0-17.5]	1 (50%) / 10.0
Patient intubated upon arrival	32 (78%)	2 (100%)
Administration of catecholamine	21 (51%)	2 (100%)
Norepinephrine only (μg kg^−1^ min^−1^)	9 (22%) / 0.4 [0.3-0.6]	2 (100%) / 0.3 ; 0.5

Title: Prehospital major events and treatment in the entire population and the « unexpected » survivors group found through the TRISS methodology. Results shown as number of patients (%) and mediane [interquartile].

### F-35 Diagnosis performance of repeated ECG for prediction of a coronary cause after cardiac arrest

#### Pierre Dupland (*speaker*)^1^, Florence Dumas^2^, Wulfran Bougouin^3^, Julien Charpentier^4^, Olivier Varenne^5^, Lionel Lamhaut^6^, Marine Paul ^4^, Jean-Daniel Chiche^4^, Frédéric Pène^4^, Jean-Paul Mira^4^, Alain Cariou^4^

##### ^1^APHP, Paris, FRANCE; ^2^Emergency Department, Cochin University Hospital (APHP) and Paris Descartes University, Paris, FRANCE; ^3^Paris Cardiovascular Research Center, INSERM U970 (team 4), Paris, FRANCE; ^4^Medical Intensive Care Unit, Cochin University Hospital (APHP) and Paris Descartes University, Paris, FRANCE; ^5^Cardiology, Cochin University Hospital (APHP) and Paris Descartes University, Paris, FRANCE; ^6^SAMU 75, Necker University Hospital (APHP) and Paris Descartes University, Paris, FRANCE

###### **Correspondence:** Pierre Dupland - pierre.dupland@gmail.com

*Annals of Intensive Care* 2019, **9(Suppl 1)**:F-35

**Introduction**: Electrocardiogram (ECG) is an essential tool for the diagnosis of acute coronary syndromes (ACS). However, diagnostic performances of post-resuscitation ECG are usually poor in the setting of out-of-hospital cardiac arrest (OHCA). Our aim was to evaluate the performances of repeated ECG during the pre-hospital period in order to identify patients who require an emergency percutaneous coronary intervention (PCI) after OHCA.

**Patients and methods**: We included a consecutive series of OHCA patients with no obvious extra-cardiac cause, in whom an immediate coronary angiogram (CAG) was performed at admission. The first ECG (early ECG) after the return of spontaneous circulation (ROSC) and the last ECG performed prior to CAG (late ECG) were classified into 4 groups, blinded to the angiographic result- (1) ST segment elevation (ST +), (2) left bundle branch block, (3) anomaly other than ST + but suspected ischemia, (4) no sign of ischemia. Respective performances of early and late ECGs were assessed using the need for early PCI as the main endpoint.

**Results**: Between 2011 and 2016, 287 patients were included, of whom 34% had a PCI at admission. A change in classification between early and late ECGs was observed in 111 patients (39%). A pattern of ST-elevation (group 1) was present on 26% of late ECGs, which predicted the need for PCI with a good specificity (87.3%) but a poor sensitivity (53.1%) (Table). In multivariate analysis, ST-elevation pattern (group 1) on late ECG was a stronger predictor for the need of PCI (OR = 6.81 (3.58–12.93), p < 0.001) as compared with the same pattern observed on early ECG (OR = 4.5 (2.59- 7.82 p < 0.001). Presence of any pattern of ischemia on the late ECG was also an independent predictor for the need of PCI (OR = 3.63 (1.74–7.6) p < 0.001), with a good sensitivity (86.4%), but a low specificity (36.2%). (Table 1) Absence of any ischemic aspect on late ECG performed after 43 min from ROSC was an independent predictor of no PCI (OR = 15.94 (3.26–77.99) p < 0.001).

**Conclusion**: Although performing better than the immediate post-resuscitation ECG, we observed that the reliability of the late ECG is insufficient in order to distinguish patients who will require an early PCI and those in whom an early CAG can be avoided.


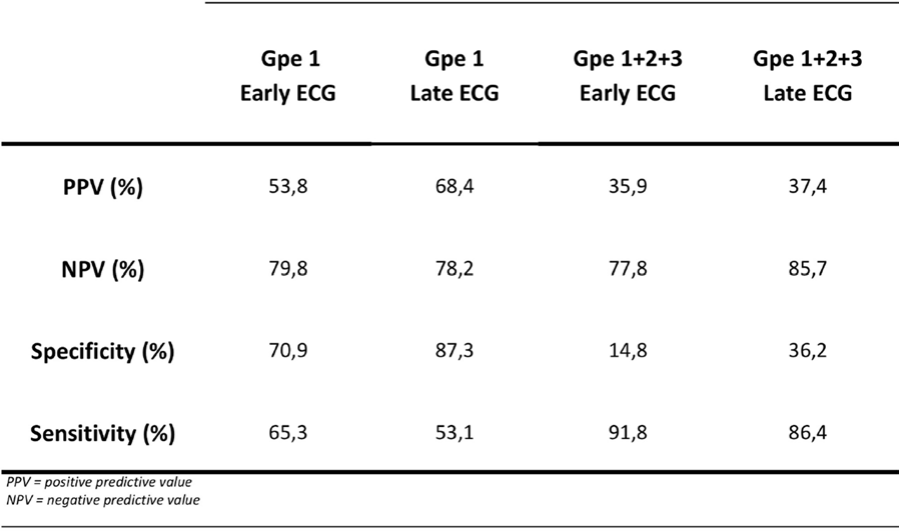


### F-36 Major traumatic complications after out-of-hospital cardiac arrest- Insights from the Parisian registry

#### Pierre-Alexandre Haruel (*speaker*)

##### Ambroise Paré, Paris, FRANCE

###### **Correspondence:** Pierre-Alexandre Haruel - pierrealexandre.haruel@gmail.com

*Annals of Intensive Care* 2019, **9(Suppl 1)**:F-36

**Introduction**: Due to collapse and cardiopulmonary resuscitation (CPR) maneuvers, major traumatic injuries may complicate the course of resuscitation for out-of-hospital cardiac arrest patients (OHCA). Our goals were to assess the prevalence of these injuries, to describe their characteristics and to identify predictive factors.

**Patients and methods**: We conducted an observational study over a 9-year period (2007–2015) in a French cardiac arrest (CA) center. All non-traumatic OHCA patients admitted alive in the ICU were studied. Major injuries identified were ranked using a functional two-level scale of severity (life-threatening or consequential) and were classified as CPR-related injuries or collapse-related injuries, depending of the predominant mechanism. Factors associated with occurrence of a CPR-related injury and ICU survival were identified using multivariable logistic regression.

**Results**: A major traumatic injury following OHCA was observed in 91 1310 patients (6.9%, 95%CI- 5.6, 8.3%), and was classified as a life-threatening injury in 36% of cases. The traumatic injury was considered as contributing to the death in 19 (21%) cases. Injuries were related to CPR maneuvers in 65 patients (5.0%, (95%CI- 3.8, 6.1%)). In multivariable analysis, age [OR 1.02 + 95%CI (1.00, 1.04); p = 0.01], male gender [OR 0.53 + 95%CI (0.31, 0.91); p = 0.02] and CA occurring at home [OR 0.54 + 95%CI (0.31, 0.92); p = 0.02] were significantly associated with the occurrence of a CPR-related injury. CPR-related injuries were not associated with the ICU survival [OR 0.69 + 95%CI (0.36, 1.33); p = 0.27].

**Conclusion**: Major traumatic injuries are common after cardiopulmonary resuscitation. Further studies are necessary to evaluate the interest of a systematic traumatic check-up in resuscitated OHCA patients in order to detect these injuries.

### F-37 PROSEDA, effectiveness of Procedural Sedation and Analgesia in emergency department, a prospective multicentric observational study

#### Romain Bouygues (*speaker*), Pierre Deneau, Geoffroy Rousseau, Saïd Laribi

##### Emergency Department, CHRU Tours, Chambray Les Tours, FRANCE

###### **Correspondence:** Romain Bouygues - r.bouygues@chu-tours.fr

*Annals of Intensive Care* 2019, **9(Suppl 1)**:F-37

**Introduction**: The objective of our study was to observe practices of procedural sedation and analgesia (PSA) in emergency departments (EDs) and to evaluate its effectiveness.

**Patients and methods**: From January to July 2018, we conducted a prospective and multicentric study, in both EDs and prehospital setting. We enrolled adult patients needing painful procedures that would require PSA. Procedures were divided into 3 groups (A- dislocations + B- displaced fractures + C- other procedures such as abscess drainage or foreign body removal…). PSA drugs were divided into 3 groups (1- sedative drugs such as Propofol, Ketamine, or Midazolam + 2- Morphine + 3- absence of drugs or 50% Oxygen Nitrous-Oxyd Premix). We used a composite primary outcome to define the success of the PSA- successful procedure, EP feeling of sufficient sedation, and the patient’s absence of painful memories from the procedure.

**Results**: 108 patients were enrolled. 61 PSA (56.5%) were successful according to our primary outcome criteria (3 points out of 3) and 35 (32.4%) reached only 2 points. Pain decrease was measured by numeric rating scale (NRS) and its median was 4.5 (IQR 3–7). Number of patient in each procedure groups were as follow- Group A- n = 48 (44.44%), Group B n = 32 (29.63%), group C n = 28 (25.93%). Drug’s group 1 was predominant (n = 59, 54.6%) and its success rate was higher (76.3%) compared to group 2 and group 3 (22.7% and 40.7% respectively, p < 0.0001). In multivariable analysis, PSA success was independently associated with only 2 factors: Ramsay sedation scale > 2 (OR = 4.584 {1.927–11.501}, p = 0.0008), as a protective factor, while morphine group was against PSA success (OR = 0.181 {0.051–0.555} p = 0.0044). Adverse event rate in our study (15.7%) was comparable with the rate in other international studies. All adverse events were easily treated and had no serious consequences.

**Conclusion**: This prospective and multicentric study of 108 patients showed that efficiency was perfect in only 56.5% of the cases, and had a satisfactory result in 88.9%. Global efficiency was positively linked to the use of sedative drugs and negatively to cutaneous procedures such as abscess drainage.


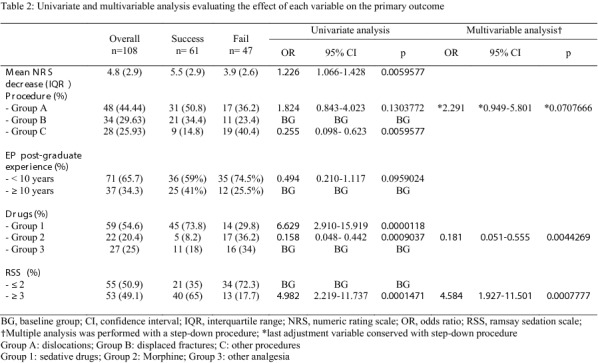


### F-38 CT scan quantification of pelvic and retroperitoneal hematoma predicts transfusion requirements, pelvic hemostatic management and outcome of severe trauma patients with pelvic fracture

#### Séverin Ramin (*speaker*), Pierre Cavaille, Margaux Hermida, Ingrid Milliet, Pauline Deras, Xavier Capdevila, Jonathan Charbit

##### Hôpital Lapeyronie, CHRU Montpellier, Montpellier, FRANCE

###### **Correspondence:** Séverin Ramin - severin.ramin@gmail.com

*Annals of Intensive Care* 2019, **9(Suppl 1)**:F-38

**Introduction**: Retroperitoneal hematoma (RPH) is frequently observed in case of pelvic fracture. RPH is the sign of traumatic pelvic hemorrhage. No study has validated a quantification score of RPH in a traumatic context. The aim of this study was to test the performance of a HRP quantification score to predict the outcome of polytraumatized patients with pelvic fracture.

**Patients and methods**: A retrospective study was performed in our trauma center between 2010 and 2015. All severe trauma patients with pelvic fracture who underwent a CT scan on admission were included. The amount of retroperitoneal effusion was quantified using a semi-quantitative method by counting the number of compartments affected by the spread of blood. Ten compartments in the retroperitoneal cavity were considered- prevesical space, laterovesical space, laterorectal space, presacral space, right and left iliopsoas space, periaortic and psoas space, right and left parietocolic space, and perirenal fascia extension. RPH was categorized in each compartment as absent (0), moderate (1), and large or bilateral (2) for a total score on 20. Patients were classified into 3 groups according to their RPH score (mild or none [0–5], moderate [6–9] and abundant [10–20]) and compared in terms of transfusion requirements, pelvic hemostatic management and prognostic variables.

**Results**: A total of 311 severe trauma patients were included (mean age- 42 ± 20 years, mean ISS- 27 ± 19, average RPH score- 6.5 ± 4.6). Among these patients, 68 (22%) had abundant RPH, 115 (37%) had RPH moderate and 128 (41%) had mild or no RPH. Massive transfusions requirement was more important in abundant RPH group (53% vs. 9% vs. 10%) as well as pelvic embolization requirement (10% vs. 3% vs 0%), pelvic fixator (15% vs. 4% vs. 1%), and mortality (26% vs. 10% vs. 8%) + P < 0.001. Similarly, days of mechanical ventilation and length of stay were significantly associated with RPH abundance (P < 0.001). The predictive value of moderate and abundant HRP for determining transfusion and interventional needs, as well as the risk of death were presented in figure 1. Using ROC curves analysis, the global ability of RPH score was robust to predict transfusion > 5 RBCs [0.75 (0.69–0.82)], massive transfusion [0.74 (0.65–0.83)] and death [0.70 (0.62–0.79)].

**Conclusion**: CT scan semi-quantitative analysis of HRP at admission allows for reliable prediction of transfusion requirements and outcome for Trauma patients with pelvic fracture.


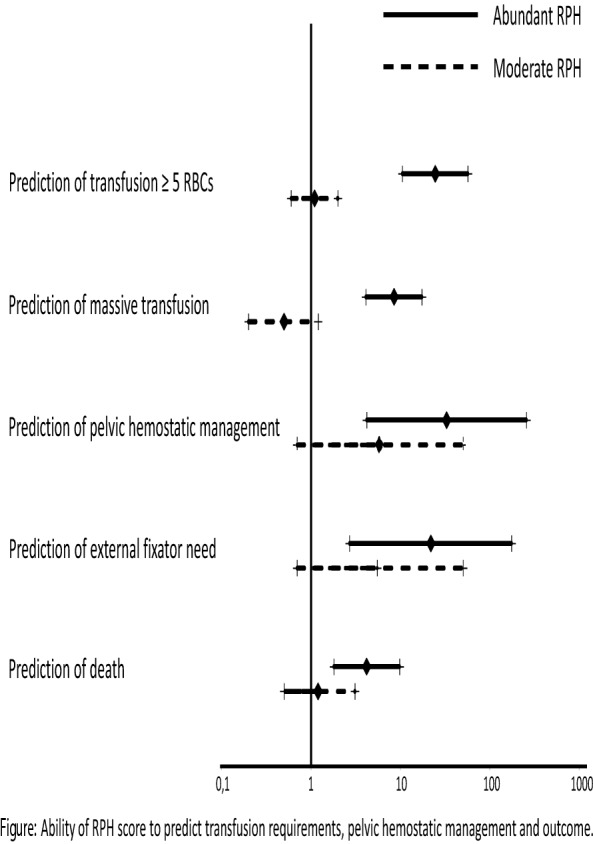


### F-39 Early post traumatic pulmonary embolism in intensive care unit

#### Mariem Dlela (*speaker*), Abir Bouattour, Olfa Turki, Hela Kallel, Mabrouk Bahloul, Hédi Chelly, Mounir Bouaziz

##### Hbib bourguiba university hospital, Sfax, TUNISIA

###### **Correspondence:** Mariem Dlela - mariem241090@gmail.com

*Annals of Intensive Care* 2019, **9(Suppl 1)**:F-39

**Introduction**: Venous thromboembolism (VTE) is a well-established complication of trauma. Recent studies suggest that pulmonary embolism (PE) may occur very early and even immediately, after injury. The aim of this study was to analyze the incidence, risk factors and prognosis of early PE among intensive care unit (ICU) trauma patients.

**Patients and methods**: We conducted a twenty month long prospective cohort, including all trauma patients with a confirmed PE diagnosis, who were admitted to our ICU between January 1st, 2017 and August 31st, 2018. Early post traumatic PE was defined as pulmonary embolism diagnosed within the first 72 h of injury. All patients, included, were screened for early PE at day 3. Factors associated with early PE were identified using both univariate and multivariate analysis.

**Results**: During the study period’s, 66 patients with positive diagnosis of PE, were included. According to our analysis, 45% (30 cases) of the patients presented with PE within 72 h of trauma events.The patients in early PE group were older than those in the late PE group (p = 0.038), had a body-mass-index (BMI) above thirty (p = 0.021) and high sequantial organ failure score (SOFA) on admission (p = .001). On the day of PE diagnosis, early group also presented with higher SOFA scoring (p < 0.001) and higher infection rate (p = 0.005). Biological assessment revealed lower platelet levels (p = 0.001) and lower P F ratio in the early group (p = 0.008). Our study showed that early PE was associated with more transfusions (p = 0.002) and surgical treatment measures (p = 0.023). The incidence of long bone fractures in lower extremities was higher in those with early PE compared with the other patients (p = 0.039). Using the multivariable logistic regression model, higher age (p = 0.028), SOFA score (p = 0.013), BMI over thirty (p = 0.002), and the use of surgical treatment measures (p = 0.046) were predictive of early timing of PE in trauma patients. Whereas, pulmonary infection was independently associated with late PE. Long bone fracture was not independently related to early occurrence of PE.

**Conclusion**: Our cohort demonstrated that many of the post-traumatic PEs occur early in the post-traumatic period. To the best of our knowledge, this is the first prospective study conducted in ICU. Further studies with larger patient populations are required to create more accurate predictive models.

### F-40 Gastric inflation induced by bag mask ventilation during different strategies of chest compressions in a cadaver model for cardiac arrest

#### Dominique Savary (*speaker*)^1^, Emmanuel Charbonney^2^, Ian Drennan^3^, Bilal Badat^4^, Paul Ouellet^5^, Stephane Delisle^6^, Caroline Fritz ^7^, Alain Mercat^8^, Laurent Brochard^9^, Jean Christophe Richard^1^

##### ^1^CH Annecy Genevois, Annecy, FRANCE; ^2^Centre de Recherche de l ‘Hôpital du Sacré-cœur, Montreal, CANADA; ^3^St Michael’s Hospital, Toronto, CANADA; ^4^Air Liquide Medical Systems, Antony, FRANCE; ^5^Vitalité Health Network, Edmunston, CANADA; ^6^Collège Ellis, Drummondville, CANADA; ^7^Inserm U1116, Nancy, Nancy; ^8^CHU, Angers, FRANCE; ^9^ID Crit Care Med University, Toronto, CANADA

###### **Correspondence:** Dominique Savary - dsavary@ch-annecygenevois.fr

*Annals of Intensive Care* 2019, **9(Suppl 1)**:F-40

**Introduction**: Bag mask ventilation is the most spread technic for ventilation for cardio pulmonary resuscitation (CPR) despite several adverse effects. Gastric gas insufflation may favor lung regurgitation and as result occurrence of aspiration pneumonia (1). We hypothesized that continuous chest compression (CC) may limit the risk of high tidal volume and gastric inflation compared to a 30–2 interrupted CC strategy. The aim of this experimental study was assess the impact of different CC ventilation strategies on gastric inflation and ventilation during a 6 min prolonged simulated CPR.

**Patients and methods**: 5 Thiel Embalmed Cadavers (TEC) from a donation program of the Université du Quebec Trois-Rivieres (CER-14-201-08-03.17) were ventilated 30 min to recruit lungs. (2). Flow and Airway Pressure were measured at the airway opening (AcqKnowledge software Biopac©). A surgical gastrostomy was performed through a 5 cm midline laparotomy to introduce a cuffed tracheal tube (size 6) into the stomach cavity + this tube was connected to a Wright spirometer to measure cumulated gastric inflated volume. Experimental protocol- 3 strategies were randomly applied during 6 min on each cadaver. 1. 30-2 with Interrupted Chest Compressions (ICC 30-2). Two successive bag insufflations after interrupting CC every 30 CC + . 2. 30-2 with Continuous Chest Compressions (CCC 30-2). Same CC ventilation ratio than in the first strategy but without interrupting CC. 3. Continuous CC with 1 bag insufflation every 6 s (CCC 10 min). Before each strategy the stomach was completely emptied through the gastrostomy tube, and the order of each strategy experimented were randomized for each cadaver.

**Results**: 5 cadavers were analyzed (mean age 75 ± 8 years, 60% female, PBW 56 ± 10 kg). Expired bag tidal volume averaged during the 6 min long period was 319 ± 165 ml during (ICC 30-2), 341 ± 142 ml during (CCC 30-2) and 277 ± 103 ml (CCC 10 min). Cumulated gastric inflated volume was significantly higher during (ICC 30-2) compared to CCC strategies (fig)- 5.9L 6 min (ICC 30-2) + 2.1L 6 min (CCC 30-2) and 2.24L 6 min (CCC 10 min) (p < 0.005).

**Conclusion**: Compared to the recommended (ICC 30-2) strategy, continuous chest compression significantly reduced cumulated gastric inflation. Interestingly, CCC did not affect ventilation actually delivered during CPR. The optimal ratio between chest compression en ventilation during continuous chest compression remains open to discussion.


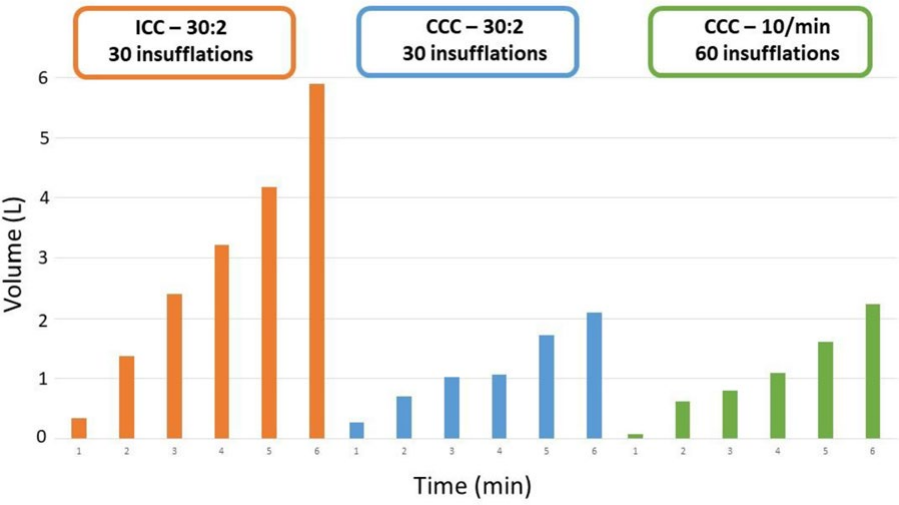


### F-41 Heart-brain interaction in acute cerebral injury

#### Mohamed Anass Fehdi (*speaker*), Amine Raja, Mohammed Mouhaoui

##### CHU Ibn Rochd, Casablanca, MOROCCO

###### **Correspondence:** Mohamed Anass Fehdi - mohamedanassf@gmail.com

*Annals of Intensive Care* 2019, **9(Suppl 1)**:F-41

**Introduction**: Cardiac events are often seen in acute cerebral palsy, and they would be an indicator of poor prognosis. The purpose of this study was to clarify the heart-brain interaction in terms of incidents and repercussions on the morbidity and mortality of cerebral palsy patients.

**Patients and methods**: It was a prospective study, over 6 months, including consecutively all the acute cerebral palsy patients, initially admitted to the vital emergency room, and having benefited from a systematic cardiac assessment, namely an ECG, an echocardiogram and a troponin assay during the 24 h. Patients transferred from another hospital and or having a cardiac check-up after 24 h were excluded. The epidemiological, clinical, paraclinical, therapeutic and evolutionary parameters were studied. A univariate statistical study was carried out to deduce the prognostic factors of early mortality (< 48 h) among cardiac assessment components (p < 0.05).

**Results**: 76 patients were included, with an average age of 56.16 ± 13.56 years, and sex ratio 1.23 (42H 34F). The diagnoses related to the cerebro-lesion were- ischemic stroke (28 cases 36%), severe head trauma (24 cases 32%), hemorrhagic stroke (20 cases 26%), severe meningitis (4 cases 6%). The cardiac events observed were- an electrical anomaly in 19 patients (25%), an increase in troponin in 13 patients (17%) and an echocardiographic anomaly in 7 patients (9%). The distribution of cardiac events by type of brain injury is shown in Table 1. The early mortality rate (< 48 h) was 16% (12 deaths)- 6 hemorrhagic stroke, 3 ischemic stroke, 3 severe head trauma, and no severe meningitis. The prognostic value of various cardiac events in cerebral palsy patients by type of aggression is shown in Table 1.

**Discussion**: Our study did not show a statistically significant difference in terms of early mortality according to the presence or absence of one or more cardiac incidents. On the other hand, very important NPVs have been noted, all brain lesions combined.

**Conclusion**: The absence of cardiac events in cerebral palsy patients would favor a better early evolution. This finding should be confirmed by a broader study. We recommend performing a cardiac checkup in any cerebral palsy patient during the first 24 h.


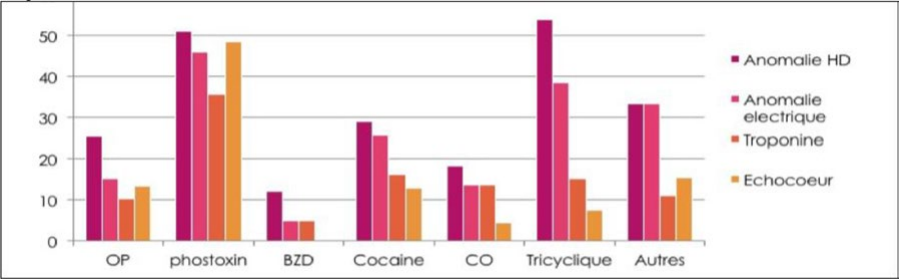


### F-42 Renal Doppler in predicting acute kidney injury (AKI) within 3 days in critically ill patients without AKI- Results of a multicenter cohort study

#### David Schnell (*speaker*)^1^, Aurélie Bourmaud^2^, Marie Reynaud^3^, Stéphane Rouleau^1^, Ferhat Meziani^4^, Alexandra Boivin^4^, Mourad Benyamina^5^, François Vincent^6^, Alexandre Lautrette^7^, Christophe Leroy^7^, Yves Cohen^8^, Matthieu Legrand^5^, Jérôme Morel^3^, Jeremy Terreaux^3^, Michael Darmon^9^

##### ^1^Réanimation, CH, Angoulême, FRANCE; ^2^Lucien Neuwirth Cancerology Institute, Saint-Priest-En-Jarez, FRANCE; ^3^Réanimation chirurgicale, Hôpital nord, Saint-Priest-En-Jarez, FRANCE; ^4^Médicale, Nouvel Hôpital Civil, Strasbourg, FRANCE; ^5^Réanimation chirurgicale et unité des brûlés, Hôpital Saint Louis, Paris, FRANCE; ^6^Réanimation polyvalente, CH, Montfermeil, FRANCE; ^7^Réanimation médicale, hôpital Gabriel Montpied, Clermont-Ferrand, FRANCE; ^8^Réanimation médico-chirugicale, CHU Avicenne, Bobigny, FRANCE; ^9^Réanimation médicale, Hôpital Saint Louis, Paris, FRANCE

###### **Correspondence:** David Schnell - david.schnell1@gmail.com

*Annals of Intensive Care* 2019, **9(Suppl 1)**:F-42

**Introduction**: Doppler-based resistive index (RI) and semi-quantitative evaluation of renal perfusion using colour-Doppler (SQP) have been suggested as potential predictors of AKI occurrence in ICU patients without renal dysfunction (1). These results are however limited to preliminary data (1). This study aimed at evaluating the performance of RI and SQP to predict AKI within 3 days in critically-ill patients without overt AKI.

**Patients and methods**: Post-hoc analysis of a multicentre prospectively collected dataset. Adult patients without cardiac arrhythmia and requiring mechanical ventilation were included. Patients with severe chronic renal dysfunction or known renal artery stenosis were excluded. AKI was defined according both urinary output and serum creatinine criteria of the KDIGO definition. Renal Doppler was performed at study inclusion. Results are reported in n (%) or median (IQR). Adjusted factors associated with AKI development were assessed using mixed logistic regression model taking centre as random effect.

**Results**: Overall, of the 351 patients included in this study, 118 had no AKI at study inclusion and were ultimately included in the post hoc analysis. Half of the patients were of male gender (55.6%; n = 66) and median age was 58 [IQR 44–67]. In addition to mechanical ventilation, 47 patients (39.8%) required vasopressors, and 46 (39.0%) had a sepsis at inclusion. Median LOD score was 7 [IQR 5–9]. Overall, 34 patients developed AKI during the first 3 days of ICU stay (28.8%). Semi-quantitative perfusion score (ranging from 3—full perfusion to 0 no perfusion) was 2 [2–3] and 2 [1–3] respectively in patients with and without AKI at day 3 (P = 0.06). Doppler-based resistive index was 0.64 [0.57–0.70] and 0.67 [0.62–0.70] respectively in patients with and without AKI at day 3 (P = 0.18; figure). Area under ROC curve in predicting AKI within 3 days was 0.60 (0.49–0.71) for SQP and 0.58 (0.47–0.60) for RI. After adjustment for confounders, neither SQP (OR 0.58; 95%CI 0.31–1.10) nor Doppler-based RI (OR 29.15; 95%CI 0.12–703) were associated with AKI at day 3.

**Conclusion**: Our results suggest that neither Doppler-based resistive index nor semi-quantitative renal perfusion is accurate in predicting occurrence of AKI in ICU patients requiring mechanical ventilation.


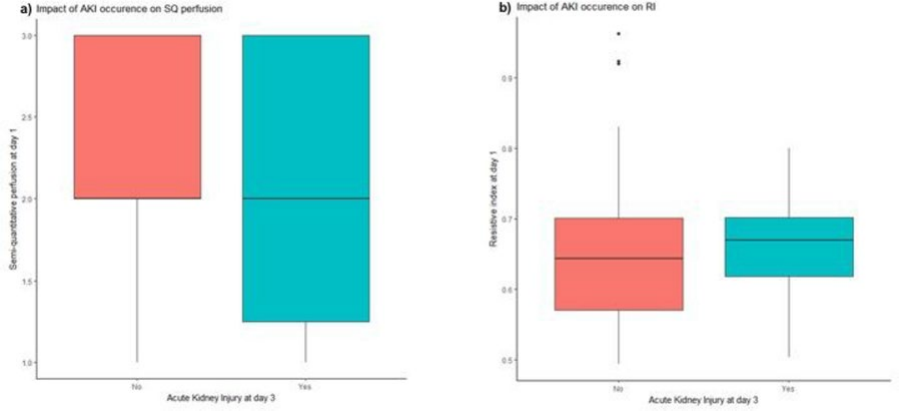


### F-43 Performance of urinary TIMP-2 and IGFBP7 and Doppler-based Resistive Index to predict reversibility of acute kidney injury in critically ill patients

#### Fanny Garnier (*speaker*)^1^, Delphine Daubin^1^, Romaric Larcher^1^, Anne-Sophie Bargnoux^2^, Laura Platon^1^, Vincent Brunot^1^, Yassir Aarab^1^, Noémie Besnard^1^, Anne Marie Dupuy^2^, Boris Jung^1^, Jean Paul Cristol^2^, Kada Klouche^1^

##### ^1^Department of Intensive Care Medicine, Lapeyronie University Hospital, Montpellier, FRANCE; ^2^Department of Biochemistry, Lapeyronie University Hospital, Montpellier, FRANCE

###### **Correspondence:** Fanny Garnier - garnier.fanny26@gmail.com

*Annals of Intensive Care* 2019, **9(Suppl 1)**:F-43

**Introduction**: The performance of urinary tissue inhibitor of metalloproteinase-2 and insulin-like growth factor binding protein7 (TIMP-2*IGFBP7) to predict renal recovery has been poorly studied. In preliminary studies, Doppler-based renal resistive index (RI) might help in differentiating transient from persistent acute kidney injury (AKI). The aim of this study was to compare the performance of TIMP-2*IGFBP7 and RI in predicting short-term reversibility of AKI in critically ill patients.

**Patients and methods**: This prospective and monocentric study included consecutive critically ill patients with AKI. RI was measured within 12 h after admission and urinary TIMP-2*IGFBP7 was measured at H0, H6, H12 and H24. Renal recovery was evaluated at day 3. Receiver-operating characteristic curves (ROCs) were plotted to evaluate diagnostic performance of RI and TIMP-2*IGFBP7 to predict a persistent AKI.

**Results**: Of the 100 patients included, 50 had transient AKI and 50 had persistent AKI. The RI was 0.61 ± 0.05 in the transient AKI group and 0.72 ± 0.05 in the persistent AKI group (p < 0.001). TIMP-2*IGFBP7 was not significantly different at each time between both groups. The performance of TIMP-2*IGFBP7 was poor with respectively an area under ROC curves of 0.57(95%CI 0.45–0.68), 0.58(95%CI 0.47–0.69), 0.61(95%CI 0.50–0.72), 0.57(95%CI 0.46–0.68) at H0, H6, H12 and H24. The area under the ROC curve for RI was 0.93 (95%CI 0.89–0.98). A RI > 0.685 predicting persistent AKI with 78% (95% CI 64–88) sensitivity and 90% (95%CI 78–97) specificity. The RI was neither correlated with age (rho = 0.12, p = 0.23), nor with mean arterial pressure (rho = -0.14, p = 0.16) nor with quantity of fluid (rho = 0.07, p = 0.49). Logistic regression found that RI (Odds ratio [OR] = 83.29 0.1-unit step, CI95% 14.91–465.14, p < 0.0001) and Sepsis-related Organ Failure Assessment score (OR = 1.51, CI95% 1.12–2.03, p = 0.001) predicted persistent AKI.

**Conclusion**: Doppler-based renal resistive index had the better performance for predicting the reversibility of AKI in critically ill patients. Urinary TIMP-2*IGFBP7 was unable to differentiate transient from persistent AKI. Further studies are needed to precise adequately the factors influencing RI.


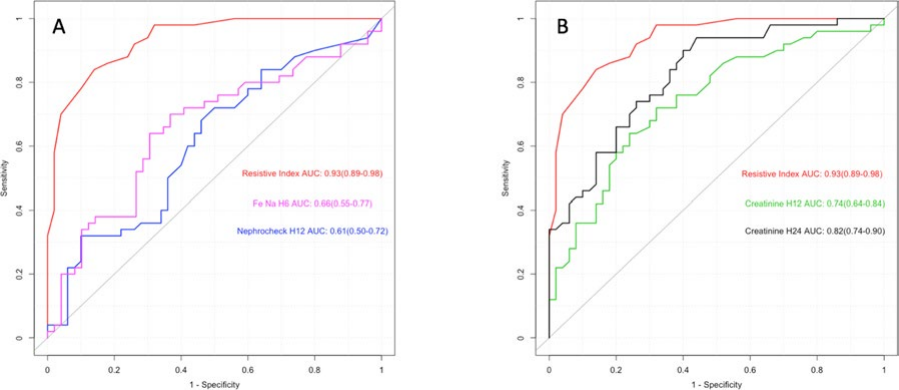


### F-44 Renin–angiotensin–aldosterone system blockers after severe acute kidney injury- use and impact on 2-year mortality

#### Mathilde Scarton (*speaker*)^1^, Anne Oppenheimer^2^, Khalil Chaibi^1^, Didier Dreyfuss^1^, Stéphane Gaudry^3^

##### ^1^Hôpital Louis Mourier, Colombes, FRANCE; ^2^Hôpital Antoine-Béclère, Clamart, FRANCE; ^3^Hôpital Avicenne, Bobigny, FRANCE

###### **Correspondence:** Mathilde Scarton - m.scarton@laposte.net

*Annals of Intensive Care* 2019, **9(Suppl 1)**:F-44

**Introduction**: Acute kidney injury (AKI) in intensive care units (ICU) carries high mortality and morbidity. Potential activation of the renin–angiotensin–aldosterone system during AKI may play a role through pro-fibrotic pathways. Renin–angiotensin–aldosterone blockers (ACEi ARB) have well known benefits for chronic kidney diseases but may be potentially nephrotoxic during AKI. Nevertheless, an ancillary study from the FROG-ICU cohort (ICM May 2018) has recently shown a lower mortality after 1 year of follow-up for patients receiving an ACEi ARB after an episode of AKI (KDIGO 1 to 3) at ICU discharge (20 109 (18%) vs 153 502 (31%), p = 0.001). The present study analyzes the use of ACEi ARB after KDIGO 3 AKI and their potential effect on long-term mortality.

**Patients and methods**: Ancillary of the AKIKI study (NEJM, 2016 + 375-122-133). All patients discharged alive from ICU were included and their long-term prognosis (2-year mortality) was assessed according to treatment with ACEi ARB at ICU discharge using both univariate and multivariate analyses after adjustment for potential confounding factors.

**Results**: Among 348 patients discharged alive, 45 (12.9%) received an ACEi ARB at ICU discharge. Table 1 details patient characteristics. Patients without ACEi ARB were more severe as attested by a higher SAPS 3 (p = 0.02) and a higher rate of catecholamine infusion (p = 0.008) during AKI. However, 2-year mortality did not significantly differ between the two groups (12 45 (27%) with ACEi ARB vs 55 303 (18%), p = 0.18). Mortality risk was not associated to non-prescription of ACEi ARB after adjustment for prognostic variables (p = 0.16).

**Discussion**: A substantial proportion of patients received an ACEi ARB at ICU discharge after an episode of severe AKI. We did not find a difference in mortality in favor of the renin–angiotensin–aldosterone system blockers, such as observed in the ancillary study of FROG-ICU (including 109 patients with ACEi ARB whereas the present study included 45). This could be explained by a different population (less severe AKI in FROG-ICU) and or a lack of power of our study.

**Conclusion**: This study does not confirm a positive effect on long-term mortality. A randomized controlled trial of ACEi ARB at ICU discharge after an episode of severe AKI is warranted.


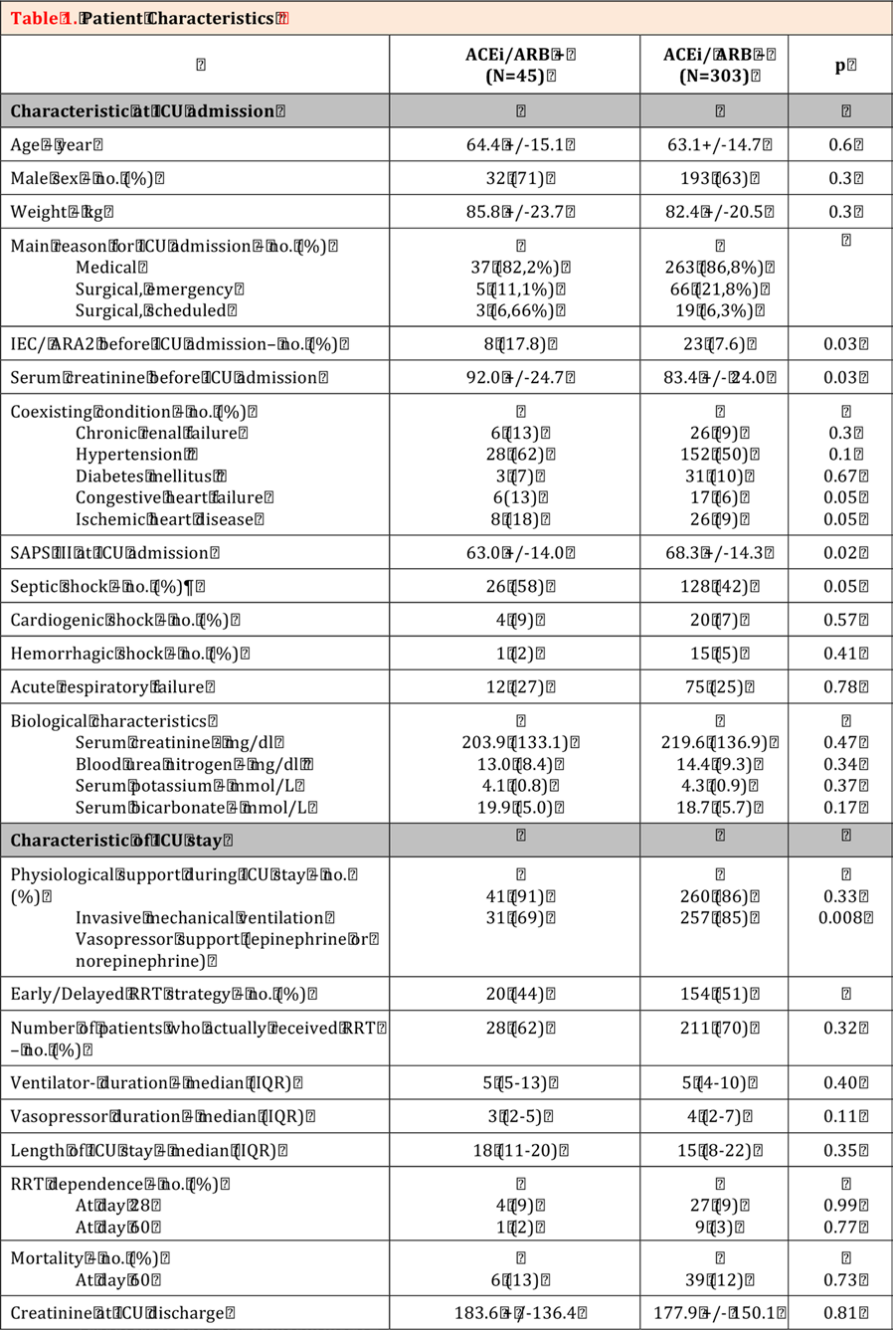


### F-45 Kinetic of uremic toxins’ concentrations during acute kidney injury and their role during endotoxemia

#### Pauline Caillard (*speaker*)^1^, Eleonore Ourouda-Mbaya^1^, Youssef Bennis^1^, Said Kamel^1^, Gabriel Choukroun^2^, Ziad Massy^3^, Julien Maizel^1^

##### ^1^University of Picardie, Amiens, FRANCE; ^2^EA7517, University of Picardie, Amiens, FRANCE; ^3^Hopital Ambroise Paré, Paris-Ile-de-France-Ouest University, Amiens, FRANCE

###### **Correspondence:** Pauline Caillard - caillard.pauline@chu-amiens.fr

*Annals of Intensive Care* 2019, **9(Suppl 1)**:F-45

**Introduction**: Chronic kidney disease (CKD) is associated with high mortality related to sepsis. Some studies have already shown uremic toxins’ action on vascular and immune disorders during CKD but their potential role during acute kidney injury (AKI) is undetermined. The kinetic of uremic toxin’s concentrations and their consequences during AKI disserve to be addressed. The aim of our study was to evaluate the kinetic of three uremic toxin’s concentrations (Indoxyl sulfate (IS), Para-crésyl sulfate (PCS) and FGF-23) during the first weeks of kidney failure in a uremic mice model and their consequences during endotoxemia.

**Patients and methods**: In this study we explored in vivo the kinetic of the three uremic toxins’ concentrations between the 7th and 45th days in controls (sham) and after kidney injury induction (KI). KI was obtained by electrocauterization followed by contralateral nephrectomy two weeks later. Uremic toxin’s concentrations were determined in sera after sacrifice at 7th, 15th and 45th days after KI induction. LPS challenge was performed (5 mg kg IP) in sham and KI groups at 15th and 45th days for survival follow up. Two other groups of KI mice fed with arabinoxylan-oligosaccharides (a chelator of PCS -group AXOS-) and sevelamer (a chelator of phosphate known to decrease FGF 23 -group sevelamer-) were exposed to LPS at 45th days of KI and followed for survival.

**Results**: PCS, IS and FGF 23 concentrations increased rapidly after kidney injury at day 7 and last until 45th in the KI group compared to the group sham (figure 1). This was associated with the elevation of cytokines concentration in serum (TNFα, IL-1β, and IL-6). At 15th and 45th days, all KI mice exposed to LPS died whereas all sham LPS animals survived (Fig 2). The treatment with Axos and sevelamer during the 45th days preceding the LPS challenge decreased respectively the PCS concentration (AXOS group), the FGF 23 concentration (Sevelamer group) and improved the survival of KI animals.

**Conclusion**: In this experimental study, the accumulation of uremic toxins appeared early in the course of kidney failure and was associated with local expression of pro-inflammatory molecules. This accumulation of toxins was associated with a higher mortality to endotoxemia. Decrease of PCS and FGF 23 concentrations was associated with the improvement of survival to LPS challenge. The early accumulation of uremic toxins and their potential role in sepsis mortality need to be confirmed during AKI in humans.


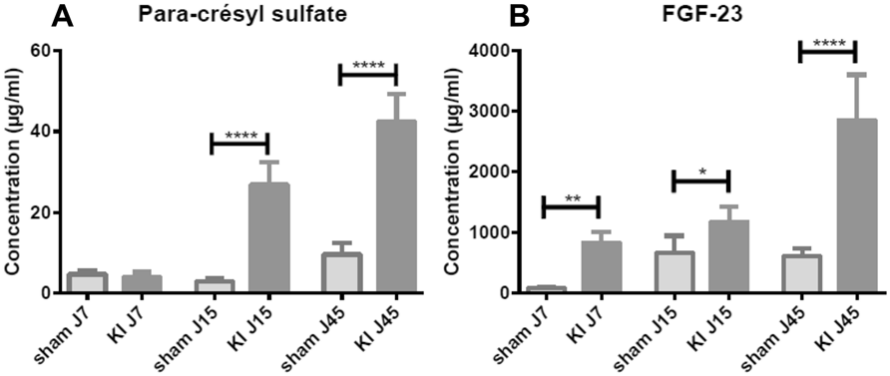


### F-46 Incidence and patterns of renal recovery in critically-ill patients with hematological malignancies

#### Arthur Orieux (*speaker*)^1^, Jean-Baptiste Lascarrou^2^, Cyril Touzeau^3^, Amélie Seguin^2^, Arnaud-Felix Miailhe^2^, Maëlle Martin^2^, Jean Reignier^2^, Emmanuel Canet^2^

##### ^1^CHU de Bordeaux, L’Herbergement, FRANCE; ^2^Médecine Intensive Réanimation - CHU, Nantes, FRANCE; ^3^Service d’hématologie - CHU, Nantes, FRANCE

###### **Correspondence:** Arthur Orieux - arthurorieux@gmail.com

*Annals of Intensive Care* 2019, **9(Suppl 1)**:F-46

**Introduction**: AKI is a dreaded complication in patients with hematological malignancies, associated with increased morbidity and mortality. Moreover, AKI may preclude the ability to receive further chemotherapy. However, current knowledge on the kinetics of renal recovery or persistent renal dysfunction is limited.

**Patients and methods**: We conducted a retrospective single-center study which included all patients with hematological malignancies admitted to the intensive care unit (ICU) of Nantes University Hospital from January to December 2017. Our purpose was to study the epidemiology of AKI and the occurrence of Major Adverse Kidney Events (MAKE).

**Results**: One hundred and three patients were included, among which 92 (89%) had AKI. Median age was 62.5 (49.75–68) years old and 59 (64%) were male. Most common malignancies were lymphoma (26, 28%), acute myeloblastic leukemia (20, 22%) and myeloma (19, 21%). 17 (18%) patients were newly diagnosed for the malignancy and 25 (27%) had relapsing diseases. Chemotherapy was administered in 64 (70%), 14 (2–31) days before ICU admission. Simplified Acute Physiology Score II (SAPS) at day 1 was 48 (39–62). During ICU stay, 37 (40%) patients received vasopressors and 28 (30%) required invasive mechanical ventilation. ICU, hospital, day-90, and day-180 mortalities were 22, 33, 38, and 43%, respectively. According to the KDIGO criteria, 47 (51%) had AKI stage 1, 16 (17%) AKI stage 2, and 29 (32%) AKI stage 3, of whom 14 (15%) required renal replacement therapy (RRT). ICU mortality of RRT patients was 78%. Overall, median duration of AKI was 5 (3–10) days, and increased from 4 (3–7) days in KDIGO stage 1 patients, to 3.5 (2.8–7.5) days in KDIGO stage 2 patients, and 12 (5–25) days in KDIGO stage 3 patients. Among survivors, renal recovery occurred in 48 (69%) patients at ICU discharge, 35 (61%) patients at day-90, and 32 (60%) patients at day-180. MAKE were reported in 32 (35%) patients at ICU discharge, 35 (38%) patients at hospital discharge, 41 (45%) 90 days after discharge, and 44 (48%) at day-180.

**Conclusion**: Critically-ill patients with hematological malignancies had a high incidence of AKI. Although almost 80% of the patients were discharged alive from the ICU, MAKE at day-90 accounted for roughly 1 in every 2 patients. Further research is needed to identify predictors of MAKE, so as to develop new therapeutic strategies which might translate into better long-term outcomes.

### F-47 Medico-economic impact of renal replacement therapy initiation strategies in the ICU

#### Abirami Thiagarajah (*speaker*)^1^, Pierre-Antoine Billiet^1^, Anne Oppenheimer^2^, Julien Maizel^3^, Fouad Fadel^4^, Eric Boulet^5^, Guillaume Chevrel^6^, Laurent Martin-Lefevre^7^, Saad Nseir^8^, Didier Dreyfuss^9^, Stéphane Gaudry^10^

##### ^1^APHP Louis Mourier, Colombes, FRANCE; ^2^APHP Antoine Béclère, Clamart, FRANCE; ^3^CHU Amiens-Picardie, Amiens, FRANCE; ^4^Centre Hospitalier René Dubos, Pontoise, FRANCE; ^5^Groupe Hospitalier Carnelles Portes de l’Oise, Beaumont Sur Oise, FRANCE; ^6^Centre Hospitalier Sud Francilien, Corbeil Essonnes, FRANCE; ^7^Centre Hospitalier Départemental Vendée, La Roche Sur Yon, FRANCE; ^8^Centre Hospitalier régional, Lille, FRANCE; ^9^APHP Louis Mourier, Colombes, FRANCE; ^10^ APHP Avicenne, Bobigny, FRANCE

###### **Correspondence:** Abirami Thiagarajah - th.abirami@gmail.com

*Annals of Intensive Care* 2019, **9(Suppl 1)**:F-47

**Introduction**: Indications and modalities of renal replacement therapy (RRT) in intensive care unit (ICU) patients with acute kidney injury (AKI) are still debated. The AKIKI trial (1) showed that a delayed RRT strategy (in the absence of life-threatening condition) did not affect mortality but allowed nearly 50% patients to escape RRT compared to an early RRT strategy in patients with KDIGO3 AKI. This has obvious economic counterparts which are evaluated in this study.

**Patients and methods**: Financial costs of RRT (both continuous and intermittent) were assessed in 45 patients in seven French ICUs from September 2017 to August 2018 taking into account catheters, circuits, dialyzer membranes and dialysate replacement fluid prices. Medical and nursing working times including time for venous catheterization and RRT duration (including circuit preparation and restitution time only) were also recorded and their cost was computed. We then extrapolated these figures to the AKIKI population in order to estimate the cost difference between the two strategies (early and delayed). Analysis was restricted to the first 72 h after inclusion.

**Results**: The mean working time was 121 (+ -60) minutes for intermittent RRT and 119 (+ -91) minutes for continuous RRT. The mean financial costs for the first 72 h after RRT initiation were 111.53 (+ -44.91) euros for the intermittent RRT and 636.21 (+ - 254.05) euros for continuous RRT (p < 0.001). Extrapolating these figures to the 619 patients included in AKIKI, revealed that the early strategy was associated with a cost of 104,619 euros and the delayed one with a cost of 54,702 euros during the first 72 h after randomization.

**Conclusion**: This study highlights the considerable savings that can safely be obtained with a delayed RRT strategy in ICU patients with severe AKI.

**Reference**

(1) Gaudry S, Hajage D, Schortgen F, Martin-Lefevre L, Pons B, Boulet E, et al. Initiation Strategies for Renal-Replacement Therapy in the Intensive Care Unit. N Engl J Med. 2016 Jul 14 + 375(2)-122–33.

### F-48 At-risk drinking is independently associated with acute kidney injury in non-trauma critically ill patients

#### Arnaud Gacouin (*speaker*), Mathieu Lesouhaitier, Aurélien Frerou, Benoit Painvin, Florian Reizine, Sonia Rafi, Adel Maamar , Yves Le Tulzo, Jean Marc Tadié

##### Service des maladies infectieuses et réanimation médicale, Rennes, FRANCE

###### **Correspondence:** Arnaud Gacouin - arnaud.gacouin@chu-rennes.fr

*Annals of Intensive Care* 2019, **9(Suppl 1)**:F-48

**Introduction**: Unhealthy use of alcohol and acute kidney (AKI) injury both are major public health problems. Chronic alcohol exposure may be directly or indirectly associated with kidney damage but little is known about the impact of current excessive alcohol consumption on kidney function in non-trauma critically ill patients. We aimed to determine whether unhealthy use of alcohol is independently associated with AKI in the intensive care unit (ICU) and worst kidney function at hospital discharge.

**Patients and methods**: Prospective cohort study on non-cirrhotic, non-liver transplant recipients, and non-chronically dialyzed patients admitted in a 21- bed polyvalent ICU in a university hospital. The study was designed to have a 90% power to detect a 15% difference in the incidence of AKI between not at-risk drinkers and at-risk drinkers at a two-sided alpha error of 5%. At-risk dinking was defined according to the National Institute on Alcohol Abuse and Alcoholism criteria and AKI according to Kidney Disease Improving Global Outcomes (KDIGO) criteria. Patients were followed until hospital discharge or day 60.

**Results**: Over a 30-months period we calculated the cumulative incidence of stage 2–3 AKI in the 320 at-risk drinkers (29%) and 787 in not at-risk drinkers (71%). Stage 2–3 AKI was significantly more frequent at admission to the ICU in at-risk drinkers than in not at-risk drinkers (42.5% versus 18%, p < 0.0001). The cumulative incidence of stage 2–3 AKI was significantly higher in at-risk than in not at-risk drinkers (Figure 1) (p < 0.0001, log-rank test). After adjustment on susceptible and predisposing factors for AKI, at-risk drinking was significantly associated with AKI (Hazard ratio (HR) = 2.53 (2.08–3.08), p < 0.0001). The proportion of patients with stage 2–3 AKI at hospital discharge among survivors was significantly higher in at-risk than in not at-risk drinkers (10% versus 5% respectively, p = 0.01). At-risk drinking remained independently associated with stage 2–3 AKI in the subgroup of 832 patients without stage 2–3 AKI at admission to the ICU (HR = 2.34 (1.68–3.72), p < 0.0001).

**Conclusion**: Our results suggest that kidney dysfunction is significantly more frequent in at-risk than in not at-risk drinkers. We believe that systematic and accurate identification of patients with prior alcohol misuse may allow for prevention of AKI.


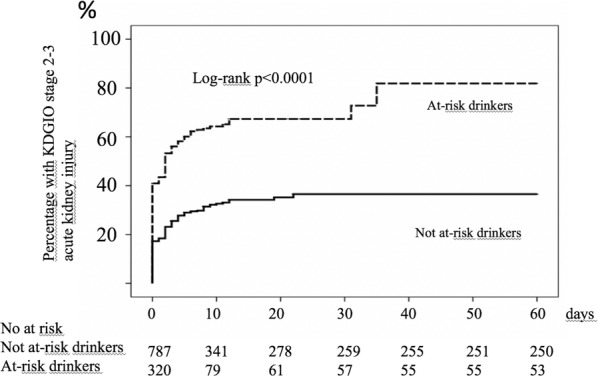


### F-49 Incidence, Epidemiology and Prognosis of Viral Respiratory Infections in Severe Acute Respiratory Failure in Human immunodeficiency virus (HIV) infected Adults

#### Alexandre Elabbadi (*speaker*)^1^, Jérémy Pichon^2^, Benoit Visseaux^3^, Quentin Philippot^2^, Aurélie Schnuriger^4^, Muriel Fartoukh^2^, Stéphane Ruckly^5^, Jean-François Timsit^8^, Guillaume Voiriot^2^

##### ^1^CHU Tenon, Paris, FRANCE; ^2^Service de Réanimation médico-chirurgicale, Hôpital Tenon, Paris, FRANCE; ^3^Service de Virologie, Hôpital Bichat, Paris, FRANCE; ^4^Service de Virologie, Hôpital Trousseau, Paris, FRANCE; ^5^UMR 1137-IAME Team 5-DeSCID, Inserm Université Paris Diderot, Sorbonne Paris Cité, Paris, Paris; ^8^Service de Réanimation médicale et infectieuse, Hôpital Bichat, Paris, FRANCE

###### **Correspondence:** Alexandre Elabbadi - alexandre.elabbadi@gmail.com

*Annals of Intensive Care* 2019, **9(Suppl 1)**:F-49

**Introduction**: Acute respiratory failure remains the main reason for admission to intensive care in HIV-infected Adults. There is little data on viral epidemiology in lower respiratory tract infections in this population.

**Patients and methods**: Cases of acute respiratory failure in HIV-infected adults admitted to two intensive care units between 2011 and 2017, who underwent screening for respiratory virus by multiplex polymerase chain reaction, were retrospectively selected.

**Results**: A total of 123 cases were included. An HIV infection was newly diagnosed in 9% of cases and 72% of the population was taking antiretroviral therapy with treatment compliance in almost 76% of cases. A documented viral respiratory infection was found in 33 patients (27%). Rhinovirus was the main virus, found in 33% of cases (n = 15) followed by Parainfluenza (n = 5) and Influenza A (n = 5). A co-infection was found in 22 patients (67% of cases) with only one virus–virus co-infection. Overall, neither the level of HIV-related immunodeficiency nor the use of ARVs at admission seems to be associated with an increased risk of respiratory viral infection. Nevertheless the subpopulation of Rhinovirus infection was associated with a low CD4 count. Outcome in the ICU was similar regardless of whether or not a respiratory viral infection was present.

**Conclusion**: Respiratory viruses are frequently found during the acute respiratory failure of the HIV subject. This proportion appears to be similar to other studies that have looked at the proportion of respiratory viruses in the general population.

### F-50 Is Human Metapneumovirus identified in the Respiratory Tract of Immunocompromised Patients with Acute Respiratory Failure Clinically Relevant?

#### Natacha Kapandji (*speaker*), Jerome Le Goff, Maud Salmona, Michael Darmon, Elie Azoulay, Virginie Lemiale

##### Hôpital Saint Louis, Paris, FRANCE

###### **Correspondence:** Natacha Kapandji - natacha.kapandji@gmail.com

*Annals of Intensive Care* 2019, **9(Suppl 1)**:F-50

**Introduction**: Human metapneumovirus (hMPV) may be responsible for pulmonary infections in immunocompromised patients. We sought to assess clinical and radiological characteristics associated with the presence of hMPV in the respiratory tract of critically ill immunocompromised patients.

**Patients and methods**: This single center retrospective cohort included adult immunocompromised patients admitted to intensive care unit (ICU) in whom hMPV was detected in respiratory tract from September 2010 to June 2018. Results are reported as n (%) and median (IQR). Comparison were performed by Fisher exact test or Wilcoxon test as appropriate.

**Results**: Of the 1185 immunocompromised patients admitted to ICU during this period, 26 (2.1%) patients presented hMPV- 15 (58%) males, aged 66 (IQR 56–74), 21 (81%) with hematological malignancies including 11 (42%) allogeneic stem cell transplant recipients. In addition to respiratory failure, 5 (19%) presented with shock. Clinical picture included fever in 23 patients (89%), cough in 19 (73%), whereas extrapulmonary symptoms were less common (11 to 31%). CT-scan patterns included alveolar consolidations in 14 (54%) patients, ground glass opacities in 10 (38%), and septa thickening in 3 (12%). All but one patient presented another pathogen- bacterial infection in 15 patients (70%), viral infection in 8 (31%) and fungal infection in one (invasive aspergillosis). At ICU admission, SAPS2 score was 41 (IQR 37–58), mechanical ventilation being required in 11 patients (42%), vasopressors in 11 (42%) and renal replacement therapy in 4 (15%). Hospital mortality rates was 31% and was found to be associated with hemodynamic failure, renal replacement therapy and neutropenia.

**Conclusion**: hMPV is found in the respiratory tract of 2.1% of immunocompromised patients with acute respiratory failure, and in all but 8 cases, another pathogen is also identified. CT patterns have nothing unique and could be ascribable to the second pathogen. Whether positive hMPV is a relevant result is unclear and remains unaddressed by this set of data. Comparing clinical and radiographic pictures and outcomes of patients with positive hMPV to matched patients with documented influenza infections is ongoing and will be displayed at the conference.
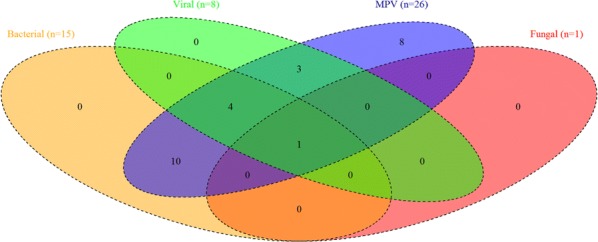


### F-51 Relative lymphopenia in patients with severe influenza A in ICU- Incidence and relation with severity illness

#### Helmi Amri (*speaker*)^1^, Amira Jamoussi^1^, Samia Ayed^1^, Takoua Merhebene^1^, Dhouha Lakhdher^1^, Amine Slim^2^, Jalila Ben Khelil^1^, Mohamed Besbes^1^

##### ^1^Medical Intensive Care Unit, Abderrahmen Mami pneumology hospital, Ariana, TUNISIA; ^2^Virology Unit, Microbiology Laboratory, National Influenza Centre, Charles Nicolle’s Hospital, Tunis, TUNISIA

###### **Correspondence:** Helmi Amri - dr.amri.helmi@gmail.com

*Annals of Intensive Care* 2019, **9(Suppl 1)**:F-51

**Introduction**: Relative lymphopenia (RL) is considered when lymphocytes count is ≤ 21% of all white blood cells. It has been reported as a possible marker of influenza A and especially pandemic influenza A H1N1 2009 infection. The aim of this study was to assess RL incidence in patients hospitalized in intensive care unit (ICU) for influenza A (H1N1 or H3N2) infection and to find whether it was related to severity illness.

**Patients and methods**: This was a retrospective monocentric study conducted in the medical ICU of Abderrahmen Mami hospital in Tunisia, between September 2009 and September 2018. Inclusion criteria were ICU patients admitted for influenza A infection. We collected clinical, biological and outcome data. Then we compared initial severity and outcome between RL patients group and normal lymphocyte count patients group.

**Results**: During the ten-year study period, we collected 72 patients with influenza A infection. The strain detected with PCR technique was H1N1 (n = 65) and H3N2 (n = 7). The mean age was of 48.2 ± 15.2 years and the sex-ratio M F was of 1.4 (42 30). Seven women were pregnant. White blood cell (WBC) value was found to be normal in 47.2% and high in 38.9% of patients with influenza A infection. Leucopenia was seen in only 10 patients (13.9%). Thrombocytopenia (≤ 150 × 103 platelets mm3) was present in 24 patients (30.6%). RL was present in 56 patients (77.8%). Ratio Lymphocyte Monocyte < 2 was noticed in 7 patients (9.7%). At admission, severity was assessed by SAPS II score (31.2 ± 16.5) and APACHE II score (12.5 ± 8.12). The main reason for ICU admission was acute respiratory failure (97.2%) from whom 35 patients (48.6%) had ARDS. Non-invasive ventilation was needed in 68% patients and 45.8% were intubated. The mean duration of mechanical ventilation was 7.96 days [0–47].The mean length of stay in ICU was 10.56 days [1–65] and the ICU mortality rate was 33.3%. Comparison of severity illness components according to RL presence are represented in table 1.

**Conclusion**: RL is frequent in ICU patients with influenza A infection. It is significantly more frequent in older patients. RL is associated to higher severity scores, but not to respiratory impairment degree or death.

**Table:** Table 1. Univariate analysis: Severity illness components according to RL

	RL (n = 56)	NL (n = 16)	p
Age mean ± SD; years	50.09 ± 15.02	41.69 ± 14.36	**0.050**
APACHE II mean ± SD	13.95 ± 8.34	7.38 ± 4.54	**0.040**
SAPS II mean ± SD	33.98 ± 17.02	21.44 ± 10.01	**0.070**
PaO_2_/FiO_2_ mean ± SD; mm Hg	165.39 ± 104.28	157 ± 78.41	0.773
ARDS n (%)	26 (46.4%)	9 (56.2%)	0.488
IMV n (%)	29 (51.7%)	4 (25%)	0.058
ICU mortality n (%)	21 (37.5%)	3 (18.7%)	0.161

RL: relative lymphopenia; NL: normal lymphocyte; ARDS: acute respiratory distress syndrome; IMV: invasive mechanical ventilation

### F-52 Critically ill influenza patients- co-infections and cardiovascular events

#### Erwan Begot (*speaker*)^1^, Renaud Prevel^2^, Fabrice Camou^3^, Walter Picard^4^, Chloé Gisbert-Mora^5^, Alexandre Boyer^3^, Didier Gruson^3^

##### ^1^Lescar, FRANCE; ^2^ICU RESIDENT, Bordeaux, FRANCE; ^3^ICU DOCTOR, Bordeaux, FRANCE; ^4^ICU DOCTOR, Pau, FRANCE; ^5^ICU DOCTOR, Bayonne, FRANCE

###### **Correspondence:** Erwan Begot - begoterwan@gmail.com

*Annals of Intensive Care* 2019, **9(Suppl 1)**:F-52

**Introduction**: Critically ill influenza patients have a mortality rate about 20%. Co-infections, especially invasive aspergillosis, have recently been documented amongst these patients and are thought to play a major role in the prognosis of these patients. Nevertheless, the need of systematic microbiological documentation is still not widely accepted by ICU health workers. Moreover, a high incidence of cardiovascular events (acute coronary syndrome or stroke) within the first 15 days after the influenza illness has been described for a few years. The aims of our study were to assess the daily practice regarding microbiological investigation of these coinfections and to assess the cardio-vascular events rates.

**Patients and methods**: A retrospective, observational study from November 2017 to April 2018 in four French ICUs including all critically ill influenza patients was conducted. Influenza diagnosis was confirmed by polymerase chain reaction or rapid test. Co-infections were confirmed using clinical and standard microbiological criteria. Stroke was diagnosed by sudden neurological symptom with MRI or contrast tomodensitometry confirmation. Acute coronary syndrome was diagnosed according to ESC guidelines.

**Results**: 111 patients were included. 98/106 patients (92%) were classified as suffering from Acute Respiratory Syndrome. 69/111 patients (62%) received non invasive ventilation during 5.7 (± 2.8) days. 57/111 patients (51%) received invasive mechanical ventilation for 10.5 (± 8.9) days. 76/111 patients (69%) have been treated by oseltamivir for 5.1 (± 1.9) days. 81/111 (73%) were still alive at Day 28. 90/111 (81%) had a bacterial sputum culture with 38/90 (42%) suffering from a bacterial co-infection, mainly S. pneumoniae and Methicillin-Susceptible Staphylococcus aureus. Only 22/111 (20%) patients had a fungal sputum culture and 27/111 (24%) a blood galactomannan dosage. Amongst those 22 patients, 5 exhibited fungi in their sputum culture, 2 of which corresponding to Aspergillus spp. and 3 to Candida spp. Viral co-infection occurred among 14/73 patients (19%), especially by Coronavirus. Regarding cardiovascular events, acute coronary syndrome occurred in 11/111 patients (10%) and stroke in 2/111 patients (1.8%) during the month after the diagnosis of influenza.

**Conclusion**: Critically ill influenza patients are frequently co-infected, by bacteria but also by virus and fungi. Fungal and viral coinfections seem to be still under investigated by ICU workers. Further studies investigating the prophylaxis and management of these coinfections are needed. Cardiovascular events prevention should also be studied among critically ill influenza patients.

### F-53 Human metapneumovirus and risk of lower respiratory tract infection, ICU admission and hospital mortality- results of a systematic review and meta-analysis

#### Natacha Kapandji (*speaker*), Virginie Lemiale, Lara Zafrani, Elie Azoulay, Michael Darmon

##### Hôpital Saint Louis, Paris, FRANCE

###### **Correspondence:** Natacha Kapandji - natacha.kapandji@gmail.com

*Annals of Intensive Care* 2019, **9(Suppl 1)**:F-53

**Introduction**: Impact of human metapneumovirus (hMPV) on lower respiratory tract infections is unclear in both children and adult patients. The aim of this review was to evaluate the prevalence of lower respiratory tract infections (LRTI), need for intensive-care-unit (ICU) admission and hospital mortality in immunocompromised adults with previous detection of hMPV.

**Patients and methods**: This systematic review was performed according to PRISMA statements and registered in the PROSPERO database (CRD42018106617). Studies reporting rate of LRTI, ICU admission and mortality were searched on PubMed (2008–2018) for immunocompromised patients. Prevalence and its confidence interval (95%CI) were plotted. Publication bias was assessed by visually inspecting the funnel plot and summary estimates of relative risk and their 95% confidence interval were calculated using random-effects model.

**Results**: Overall, 42 citations were identified and 29 studies, reporting 1407 patients, were ultimately included (21 cohort studies and 8 case series). Median sample size was 13 patients (IQR 7–48) and median admission year was 2009 (IQR 2008–2012). Diagnosis of hMPV infection was performed using RT-PCR on nasopharyngeal swab in 22 studies (76%). Proportion of patients with hematological malignancies was 97% (IQR 0–100), and proportion of allogeneic stem cell transplantation was 11% (IQR 0–47). LRTI prevalence was 60% (95%CI 48–73 + I2 = 94%). ICU admission was required in 20% of patients (95%CI 14–27 + I2 = 90%). Last, hospital mortality was 6% (4–9 + I2 = 79%). Factors associated with heterogeneity were assessed using meta-regression. Heterogeneity of the results were partly explained by study design, proportion of patients with hematological malignancy and proportion of patients with co-infection.

**Conclusion**: In this systematic review, two third of immunocompromised patients in whom hMPV was detected had LRTI, and as many as 20% required ICU admission. A high heterogeneity was noted that may be explained by study design, underlying immune status and underlying disease and rate of co-infections. Most of the included studies were however at high risk of bias justifying need for additional studies in this field.


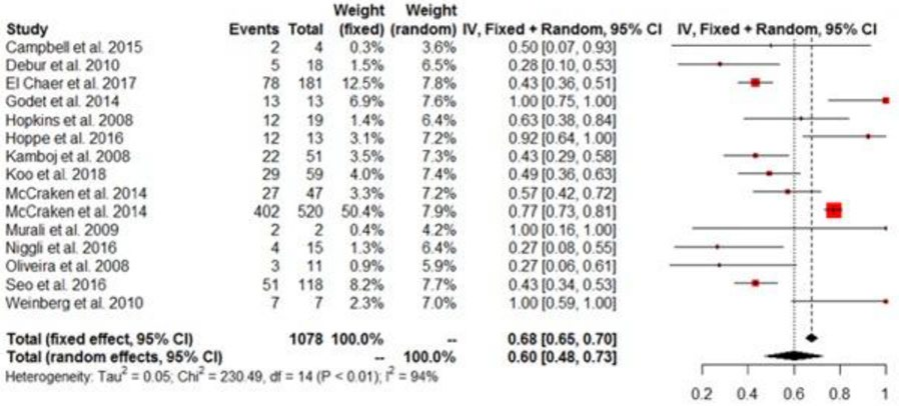


### F-54 Impact and characteristics of respiratory viruses infection during severe acute respiratory failure in adults

#### Jérémie Pichon (*speaker*)^1^, Lila Bouadma^2^, Benoit Visseaux^3^, Aurélie Schnuriger^4^, Jean-François Timsit^2^, Muriel Fartoukh^5^, Guillaume Voiriot^6^

##### ^1^CHU Tenon, Paris, FRANCE; ^2^Service de Réanimation médicale et infectieuse, Hôpital Bichat, Hôpitaux Universitaires Paris Nord Val de Seine, APHP, FRANCE; ^3^Université Paris Diderot, Sorbonne Paris Cité, Service de Virologie, Hôpital Bichat, Hôpitaux Universitaires Paris Nord Val de Seine, APHP, FRANCE; ^4^Service de Virologie, Hôpital Trousseau, Hôpitaux Universitaires de l’Est Parisien, APHP, Sorbonne Université, Paris, FRANCE; ^5^Service de Réanimation médico-chirurgicale, Hôpital Tenon, Hôpitaux Universitaires de l’Est Parisien, APHP, Sorbonne, Paris, FRANCE

###### **Correspondence:** Jérémie Pichon - jeremie.pichon1@gmail.com

*Annals of Intensive Care* 2019, **9(Suppl 1)**:F-54

**Introduction**: The mPCR (multiplex Polymerase Chain Reaction) tests are widely used in clinical routine. However, clinical and paraclinical features of virus-associated respiratory tract infections in adults are poorly described, especially in acute respiratory failure requiring critical care.

**Patients and methods**: This was a prospective non-interventional 2-center clinical study. All adult patients admitted to intensive care unit for an acute respiratory failure and subjected to a multiplex PCR (16 virus, nasopharyngeal swab and or distal sample) during the first 72 h of ICU stay were included. Prior to get the mPCR results, clinicians were invited to subject patients to a questionnaire asking for 37 symptoms (respiratory and extra-respiratory) coded from 0 (none) to 4 (maximum). Other sources may be examined, such as patient relatives, general practitioner or medical reports.

**Results**: From December 2015 to April 2017, 339 patients were included (209 men, age 63 years [50–73]), of whom 82 were intubated. The final diagnosis was pneumonia in 121 patients, exacerbation of chronic bronchial disease in 117 subjects and another diagnosis in 101 subjects. At least one virus was identified in 121 patients, including 40 Rhinovirus, 34 Influenza, 13 Respiratory syncytial virus, 14 Metapneumovirus and 15 Coronavirus. In univariate analysis, an active smoking, a close contact with somebody ill, a rhinorrhea, a bronchorrhea, an earache, a diarrhea, a lymphopenia, a thrombocytopenia and elevated CPK were associated with the documentation of respiratory viruses within airways.

**Conclusion**: A phenotype suggesting a virus-associated respiratory tract infection was described. It may help to rationalize the use of mPCR tests.


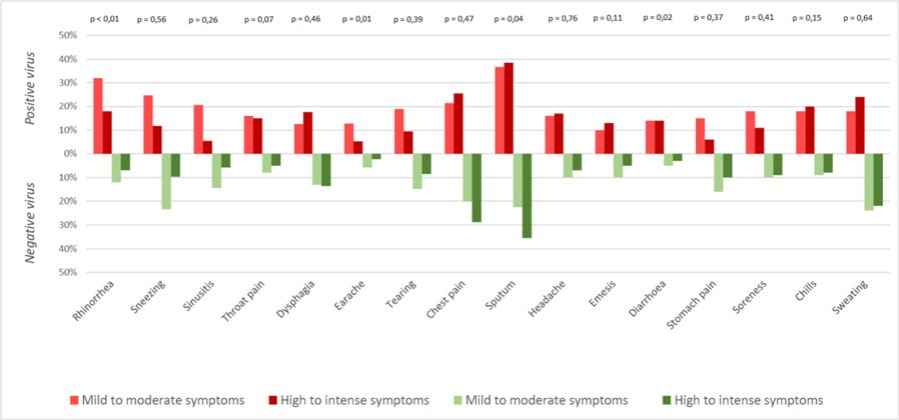


### F-55 Transfusion of packed red blood cells is associated with an increased risk of ICU-acquired infections and mortality in septic shock patients

#### Edwige Peju (*speaker*)^1^, Jean-François Llitjos^1^, Julien Charpentier^1^, Alain Cariou^1^, Jean-Daniel Chiche^1^, Jean-Paul Mira^1^, Matthieu Jamme^2^, Frédéric Pène^1^

##### ^1^Médecine-Intensive Réanimation, Paris, FRANCE; ^2^Urgences néphrologiques et transplantation rénale, Paris, FRANCE

###### **Correspondence:** Edwige Peju - edwigepeju@hotmail.fr

*Annals of Intensive Care* 2019, **9(Suppl 1)**:F-55

**Introduction**: Transfusion of packed red blood cells (RBC) is commonly indicated in septic patients to improve tissue oxygen delivery. Besides uncertain benefits, RBC transfusions carry immunomodulatory properties likely to increase the susceptibility to further ICU-acquired infections or the mortality rate. The aim of this study is to address the impact of RBC transfusion on ICU-acquired infections and mortality in septic shock patients.

**Patients and methods**: This was a 10-year (2008–2017) monocenter retrospective study. All consecutive adult patients diagnosed for septic shock within the first 48 h were included. Septic shock was defined as a microbiologically proven or clinically suspected infection, associated with acute circulatory failure requiring vasopressors. The number of packed RBC and the transfusion day were recorded. The diagnosis of nosocomial infections was based on current international guidelines. Patients alive at day 3 were evaluated for the risk of ICU-acquired infections. The determinants of ICU-acquired infections and 30-day mortality were addressed in a multivariate time-dependent Cox regression analysis.

**Results**: Among 1152 patients admitted for septic shock, 63% were males, the median age was 69 (57–79) years old and the crude 30-day mortality rate was 31.5%. Overall, 512 patients (44%) received RBC transfusions, with a median of 3 (2–6) units. In multivariate analysis, transfusion was independently associated with prior immunosuppression (OR = 1.57, 95%IC [1.21–2.04], p = 0.001), with chronic kidney disease (OR = 1.89, 95%IC [1.30–2.74], p = 0.001) and a higher admission SOFA score (OR = 1.03, 95%IC [1.01–1.06], p = 0.007). 1038 patients were alive at day 3, of whom 253 (24%) developed ICU-acquired infections. Among them, 197 (78%) patients had received RBC prior to the episode of ICU-acquired infection. In multivariate analysis, RBC transfusion was independently associated with the development of ICU-acquired infections (OR = 1.88, 95%IC [1.21–2.93], p = 0.005). 30-day mortality was significantly higher in patients receiving RBC (37.6% vs. 27.8%, p < 0.001). In multivariate analysis, RBC transfusion was independently associated with 30-day mortality (OR = 1.50, IC95% [1.07–2.09], p = 0.02).

**Conclusion**: Transfusion of RBC in septic shock patients is associated with an increased risk of ICU-acquired infections and 30-day mortality. These results support a restrictive RBC transfusion policy in septic shock patients.

### F-56 Effect of age on mortality in patients with haematological malignancy in intensive care units

#### Jean-Edouard Martin (*speaker*)^1^, Michael Darmon^1^, Virginie Lemiale^1^, Djamel Mokart^2^, Frédéric Pène^3^, Achille Kouatchet^4^, Julien Mayaux ^5^, Francois Vincent^6^, Martine Nyunga^7^, Fabrice Bruneel^8^, Christine Lebert^9^, Pierre Perez^10^, Anne-Pascale Meert^11^, Dominique Benoit^12^, Rebecca Hamidfar^13^, Mercé Jourdain^14^, Lionel Kerhuel^1^, Laure Calvet^1^, Etienne Ghrenassia^1^, Samir Jaber^15^, Elie Azoulay^1^, Audrey De Jong^15^

##### ^1^Saint-Louis Hospital, Paris, FRANCE; ^2^Institut Paoli Calmette, Marseille, FRANCE; ^3^Cochin Hospital, Paris, FRANCE; ^4^Centre Hospitalier Universitaire, Angers, FRANCE; ^5^Pitié-Salpétrière Hospital, Paris, Paris; ^6^Avicenne Hospital, Bobigny, FRANCE; ^7^Victor Provo Hospital, Roubaix, FRANCE; ^8^Mignot Hospital, Versailles, FRANCE; ^9^Montaigu Hospital, La Roche-Sur-Yon, FRANCE; ^10^Brabois Hospital, Nancy, FRANCE; ^11^Institut Jules Bordet, Bruxelles, BELGIUM; ^12^Ghent University Hospital, Ghent, BELGIUM; ^13^Albert Michallon Hospital, Grenoble, FRANCE; ^14^Salengro Hospital, Lille, FRANCE; ^15^CHU, Montpellier, FRANCE

###### **Correspondence:** Jean-Edouard Martin - j.edouard.martin@gmail.com

*Annals of Intensive Care* 2019, **9(Suppl 1)**:F-56

**Introduction**: Respective influence of age and performance status (PS) of critically ill patients with malignancy has been poorly studied. The main objective of this study was to analyse the impact of age on day-90 mortality in this population.

**Patients and methods**: We performed a post hoc analysis of prospective multicentre data from France and Belgium to identify the relation between age and day-90 mortality. Five classes of age were computed according to quintile of ages. The best threshold of age was determined using a Youden index analysis. Univariate and multivariate Cox analysis of day-90 mortality were performed. Kaplan–Meier curves of day-90 mortality according to age and main risk factors were computed.

**Results**: 1011 patients were included. Age categories according to quintile repartition were the following- 18 to 45 years old (y) (n = 206), 46 to 56 y (n = 194), 57 to 63 y (n = 213), 64 to 71 y (n = 189), and 72 to 87 y (n = 209). Next, two age groups was separated- younger group, age < 64 y and older group age ≥ 64 y with a median age of 52 y (25–75% IQR, 41–59) and 72 y (67–77) respectively. Older age (≥ 64) was significantly associated with higher mortality rate in univariate analysis (HR = 1.54 (1.26–1.88), p < 0.0001). After multivariate cox analysis, main risk factor for mortality were age ≥ 64 (HR = 1.55 (1.25–1.92), p < 0.0001), PS ≥ 3 (HR = 1.47 (1.40–1.87), p = 0.0024) and severity at inclusion assessed by sequential organ failure assessment (SOFA) score at admission in ICU per one unit increase (HR = 1.18 (1.15–1.21), p < 0.0001). However, haematological malignancy, Charlson comorbidity score index without age and reason for ICU admission were not significantly associated with day-90 mortality. Figure 1 shows the Kaplan–Meier survival analysis of the relation between age, PS and day-90 mortality (p < 0.0001).

**Conclusion**: Older age is significantly associated with higher day-90 mortality. A threshold of 64 y was found to be the most accurate to discriminate dead from alive patients. Despite its prognostic impact, survival was meaningful in subgroups of oldest patients with moderately limited autonomy. This study may allow a better selection of oldest patients likely to benefit of ICU admission according to three simple variables- age, PS and SOFA score.


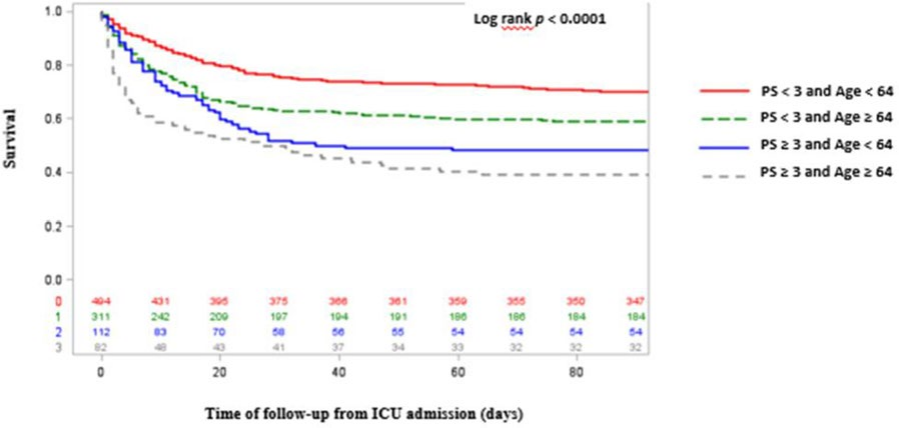


### F-57 Hemodynamic failure in critically ill patients with hemophagocytic syndrome

#### Thomas Frapard (*speaker*)^1^, Sandrine Valade^1^, Eric Mariotte^1^, Jehane Fadlallah^2^, Lionel Galicier^2^, Michael Darmon^1^, Elie Azoulay^1^

##### ^1^Médecine intensive et réanimation Saint Louis, APHP, Paris, FRANCE; ^2^Immunopathologie clinique Saint Louis, APHP, Paris, FRANCE

###### **Correspondence:** Thomas Frapard - t.frapard@gmail.com

*Annals of Intensive Care* 2019, **9(Suppl 1)**:F-57

**Introduction**: Hemophagocytic syndrome (HS) is a rare life-threatening condition that can lead to multi organ failure, including shock. In severe HS, symptomatic treatment relies on Etoposide (VP16) infusion. Hemodynamic instability during HS has been poorly studied. Objectives of this study were to describe the characteristics of HS patients with shock, prognostic factors and the impact of etoposide injection on the hemodynamic parameters.

**Patients and methods**: Adult critically ill patients with HS and managed in a multidisciplinary national reference center between 2007 and 2017 were retrospectively included. Patients without vasopressors or not requiring Etoposide infusion were excluded.

**Results**: Forty patients were included. Two-third (n = 28) were of male gender and median age was 48y [IQR 37–62]. Shock (n = 15, 37%), acute respiratory failure (n = 10, 25%) and monitoring (n = 8, 20%) were the main reasons for ICU admission.

The most common HS-triggers were underlying hematologic disease (malignancies HHV8-related disease) in 31 patients (77%), infectious diseases in 4 (10%), and systemic rheumatic diseases in 3 (8%). Median SOFA score was 11 [9–13], 85% of the patients required mechanical ventilation (n = 34) and median lactate level was 4 mmol/L [2.7–6.9]. Hospital mortality was 47% (n = 19) and was associated with severity as assessed by need for mechanical ventilation (100% vs. 73% + P = 0.04) and male gender (90% vs. 47% in survivors + P = 0.01). Etoposide infusion (H0) was followed by increased norepinephrine doses (P = 0.03) and a trend toward higher lactate levels (P = 0.07 + figure 1). No statistically significant change was observed as regard to mean arterial pressure, heart rate and renal function, assessed by serum creatinine and oliguria.

**Conclusion**: Our results suggest a high severity of HS patients with acute circulatory failure, a high hospital mortality and a hemodynamic worsening in the 24 h following etoposide infusion. Change in hemodynamic through ICU stay and comparison to patients with cytopenias and shock in the absence of HS are currently ongoing and will be presented at the congress.


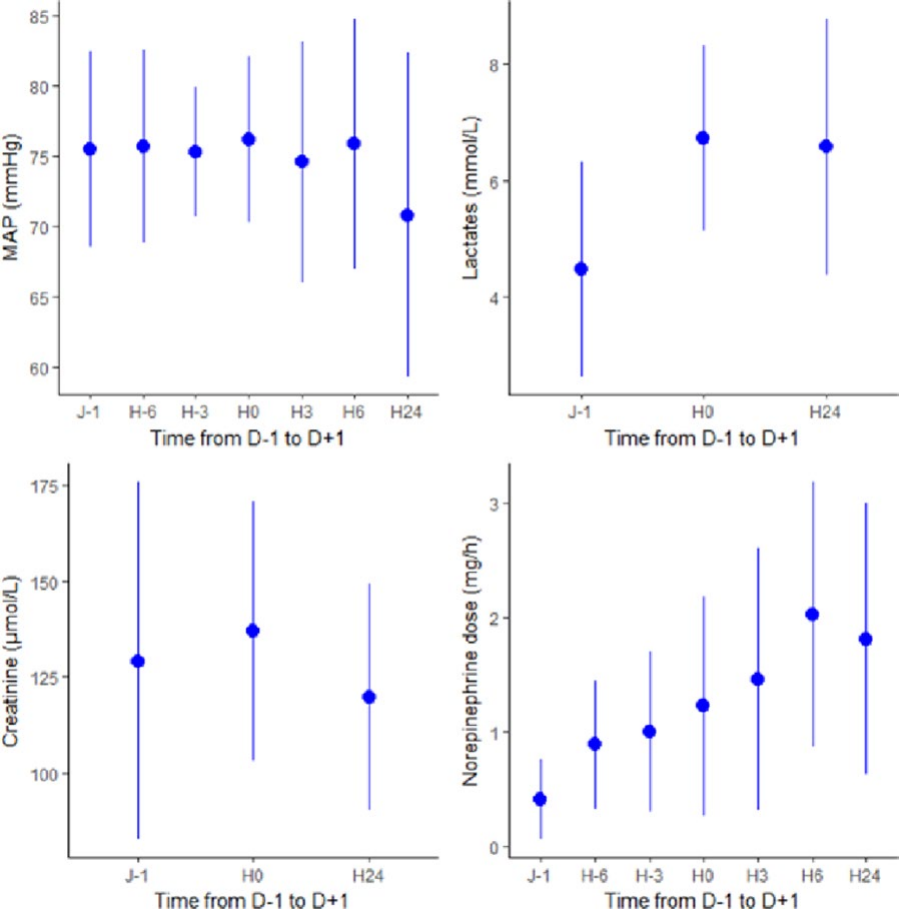


### F-58 Long-term prognosis of high-grade glioma admitted in the intensive care unit

#### Maxens Decavèle (*speaker*)^1^, Nicolas Gatulle^2^, Nicolas Weiss^3^, Léa Lemasle^2^, Ahmed Idbaih^5^, Julien Mayaux^2^, Thomas Similowski^2^, Alexandre Demoule^2^

##### ^1^Hôpital La Pitié-Salpêtrière, Paris, FRANCE; ^2^AP-HP, Groupe Hospitalier Pitié-Salpêtrière Charles Foix, Service de Pneumologie et Réanimation Médicale (Département R3S), Paris, FRANCE; ^3^Unité de Réanimation Neurologique, Département de Neurologie, Pôle des Maladies du Système Nerveux et Institut de Neurosciences Translationnelles, IHU, Paris, FRANCE; ^4^Inserm U 1127, CNRS UMR 7225, Sorbonne Universités, UPMC Univ Paris 06, Institut du Cerveau et de la Moelle épinière, ICM. AP-HP, Paris, FRANCE

###### **Correspondence:** Maxens Decavèle - maxencesar@hotmail.fr

*Annals of Intensive Care* 2019, **9(Suppl 1)**:F-58

**Introduction**: Only limited data are available concerning prognosis of primary malignant brain tumors in the intensive care unit (ICU). Among them, high-grade gliomas (HGG) are the most frequent and those associated with the poorest survival. Whereas long-term prognosis after ICU admission of patients with other malignancies is now better known, no such data exist regarding patients with HGG. The aims of our study were 1) to analyze factors associated with 1-year mortality in patients with HGG admitted to the ICU and 2) to assess the functional status and anti-cancer therapy course in ICU survivors.

**Patients and methods**: Eight-year, bicentric, retrospective cohort study. All consecutive patients with HGG, admitted to the ICUs were included. Functional status was assessed with the Karnofsky Performance Status (KPS). Mutation in isocitrate dehydrogenase (IDH) 1 and 2 was also collected. The anti-cancer therapy course after ICU discharge was classified in 1) continued without change, 2) changed (modified or stopped after ICU discharge), and 3) initiated after ICU discharge for a HGG diagnosed during the ICU stay).

**Results**: Seventy-eight patients (age 58 [45–67] year-old, SAPSII 32 [21–52]) were included, of which 62 (79%) were glioblastoma. Main reasons for admission were coma and acute respiratory failure (47 (60%) and 17 (22%), respectively). Mechanical ventilation and vasopressors were required in 40 (51%) and 17 (22%) of cases. ICU and 1-year mortality was 12 (15%) and 62 (79%). Among ICU survivors, anti-cancer therapy course was continued, changed and initiated in 26 (33%), 41 (53%) and 11 (14%) patients, respectively. One-year survival was significantly higher in patients in whom anti-cancer therapy was continued, as compared to others (16 (62%) vs. 46 (92%), p = 0.002). Among ICU survivors, the KPS did not vary between 1 month and 1 year after ICU discharge (55 [48–78] vs. 60 [50–58], p = 0.212). In multivariate analysis, factor associated with 1-year survival were the KPS at admission (OR 0.894 95%CI [0.807–0.953], p = 0.005), and anti-cancer therapy course continued (OR 0.009 [0.001–0.102], p = 0.002). The IDH status did not impact on 1-year mortality.

**Conclusion**: Despite high long-term mortality rate, 85% of patients survived to the ICU, near a half continued their planned anti-cancer therapy course and more than 20% were alive 1 year after ICU discharge, with good functional status.

### F-59 Early Identification of Sickle-Cell Disease Patients at Risk for Complicated Outcome in Intensive Care Unit. Aggregation of multiple sickle cell prediction model, updating and validation on CARADBDREPA cohort

#### Amélie Rolle (*speaker*)^1^, N’Guyen Tri Long^2^, Thomas P. A. Debray^3^, Zakaria Mahi^1^, Bertrand Pons^1^, Ruddy Valentino^4^, Hossein Mehdaoui ^4^, Michel Carles^1^

##### ^1^CHUG, Pointe-à-Pitre, FRANCE; ^2^CHU Montpellier_Nimes, Montpellier, FRANCE; ^3^Julius Center for Health Sciences and Primary Care, Utrecht, THE NETHERLANDS; ^4^CHUM, Fort-De-France, FRANCE

###### **Correspondence:** Amélie Rolle - melie9712@hotmail.com

*Annals of Intensive Care* 2019, **9(Suppl 1)**:F-59

**Introduction**: Sickle cell disease (SCD) is an increasing global health problem. Better prediction of the severity of sickle cell disease could lead to more precise a treatment and management. However, though several prediction models for SCD have been published, their external validity remains unclear. Objectives- The objective of our study was to validate existing prediction models SCD patients at risk for complicated outcome, and to combine and simultaneously update these models into a new so-called ‘meta-model’. Primary endpoint- Composite, binary outcome (‘complicated outcome’) defined by intensive care unit stays > 2 days, need for vital support or death.

**Patients and methods**: From January 2012 to December 2017, a retrospective cohort study was conducted at the University Hospital of Guadeloupe (French territories in the Americas). It included all patients (children and adults) admitted to this institution. To combine existing prediction models into a ‘meta-model’, we performed a systematic review searching in PubMed, EMBASE and bibliographies of articles retrieved. We screened relevant studies that enrolled sickle-cell disease children and adult patients for whom a prediction model was presented.

**Results**: Of the 3829 patient admissions in our cohort, 210 (23%) experienced a complicated outcome, defined as death (8 patients, 3.8%), acute respiratory failure (119 patients, 56%) hemodynamic failure (24 patients, (11%), or renal failure (12 patients, 6%). Four prediction models were combine to predict patient risk of complicated outcomes. The resulting meta-model demonstrated good predictive performances in terms of discrimination (c-statistic- 0.8) and calibration.

**Conclusion**: Combining existing prediction models might help clinicians obtain valid predictions of complicated outcomes and rapidly improve the quality of care of SCD patients admitted to emergency department.


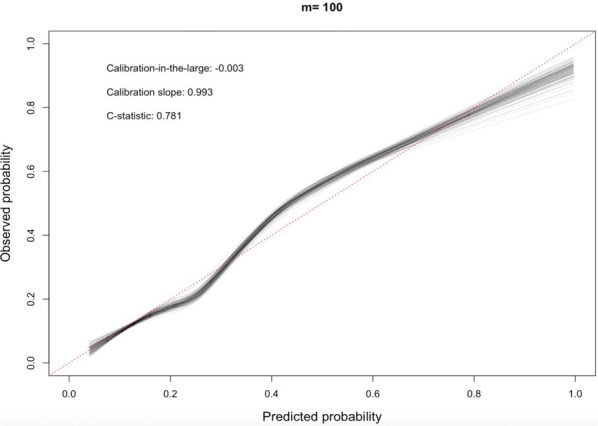



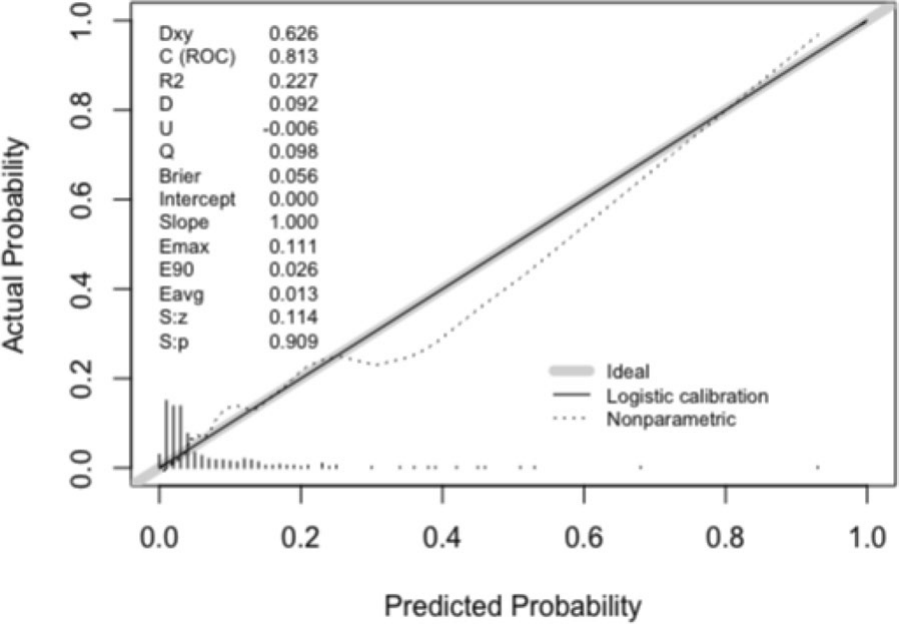


### F-60 Performances of HLH criteria and H-Score in ICU patients with severe hemophagocytic syndrome

#### Sandrine Valade (*speaker*), Grégoire Monseau, Laure Calvet, Eric Mariotte, Virginie Lemiale, Zafrani Lara, Elie Azoulay, Michaël Darmon

##### Réanimation Médicale, Hôpital Saint-Louis, AP-HP, Paris, FRANCE

###### **Correspondence:** Sandrine Valade - sandrine.valade@aphp.fr

*Annals of Intensive Care* 2019, **9(Suppl 1)**:F-60

**Introduction**: Hemophagocytic syndrome (HS) is a serious condition that can lead patients to intensive care unit (ICU) admission. Diagnosis may be difficult in these patients who may have multiple organ failures. HLH criteria are the most commonly used, but a new diagnostic score has recently been established (the H-Score). The main objective of this study is to analyze diagnostic performance of these diagnostic scores in ICU patients.

**Patients and methods**: Two convenient samples were analyzed including a sample of 150 patients with confirmed HS (HS +). A second sample of 1011 patients without HS (HS-) was obtained from a multicenter cohort of onco-hematological patients. Results are presented as median (interquartile range) and numbers (%).Area under ROC curves were established to assess discriminancy of both scores in diagnosing HS. A sensitivity analysis was performed after propensity score (PS) matching according to temperature and cytopenia.

**Results**: Overall, 1161 patients were included in this study. HS + patients were younger (median age 48.5 years [38–59] vs 60 [49–70], p < 0.001), had more severe cytopenia (hemoglobin 8.3 g/dL [7.23–9.17] and platelets 44000 mm3 [21000–79000] vs 62000 [29000–140000]), had more often organomegaly (hepatomegaly in 68.7% vs 8%, splenomegaly in 61.3% vs 9%). Mortality rate was 45.8% in hemophagocytic patients and 38.8% in control patients. Median H-Score was 235 [205–262] in SH + patients and 42 [18–62] in SH- patients. Number of HLH criteria was 4 [4–5] and 1 [0–1] respectively. Diagnostic performance of both score was excellent with area under ROC curve of 0.99 (95%CI according to DeLong Method of 0.99–0.99) and 0.99 (95%CI 0.99–0.99) for HLH and H-score respectively (figure). After propensity score matching (n = 144*2), the median H-Score was of 234 [205–262] in SH + patients versus 49 [18–71] in SH- patients. Median number of HLH criteria were 4 [4–5] in SH + and 1 [0–1] in SH- patients. Area under ROC curve was of 0.98 (CI95% 0.96–0.99) for HLH criteria and 0.99 (CI95% 0.99–1) for H-Score.

**Conclusion**: H-Score and HLH criteria are highly sensitive and specific in ICU patients. Further studies in unselected cohort of consecutive ICU patients with suspected HS are warranted in order to confirm our results and optimal cut-off for these scores.


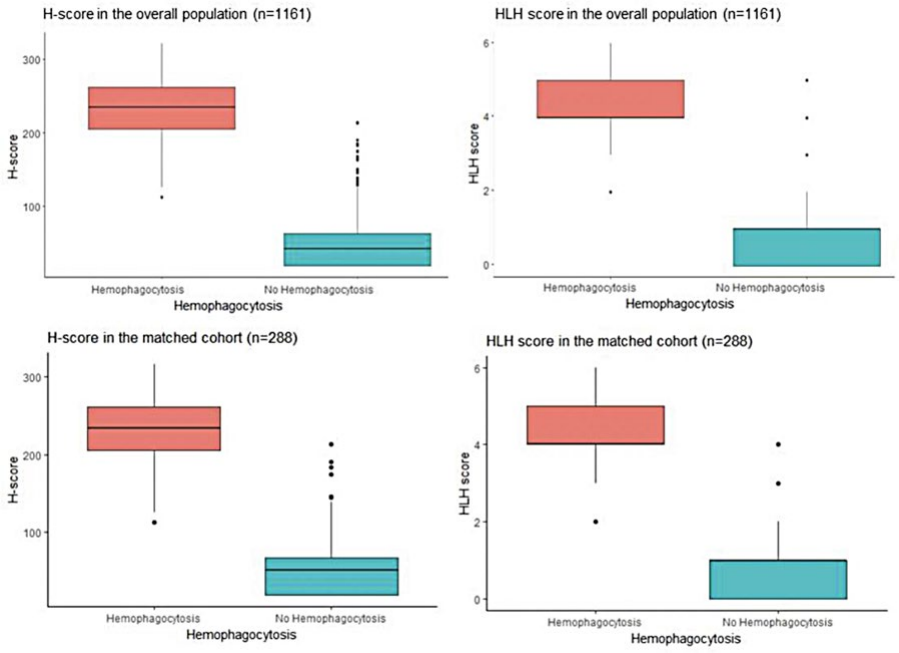


### F-61 Post-transfusion platelet increments in critically ill cancer patients with hypoproliferative thrombocytopenia

#### Elodie Baron (*speaker*)^1^, Anne François^2^, Julien Charpentier^1^, Habib Ben Hadj Amor^3^, Bassem Habr^1^, Alain Cariou^1^, Jean-Daniel Chiche^1^, Jean-Paul Mira^1^, Matthieu Jamme^4^, Frédéric Pène^1^

##### ^1^Réanimation, CHU Cochin, Paris, FRANCE; ^2^EFS, CHU HEGP, Paris, FRANCE; ^3^CHU Cochin, EFS, Paris, FRANCE; ^4^UNTR, CHU Tenon, Paris, FRANCE

###### **Correspondence:** Elodie Baron - elodie.psa@gmail.com

*Annals of Intensive Care* 2019, **9(Suppl 1)**:F-61

**Introduction**: Thrombocytopenia is a common disorder in intensive care unit (ICU) and is associated with an increased risk of bleeding. Most data about platelet transfusions in the ICU have been obtained from general cohorts with peripheral thrombocytopenia and ongoing active bleeding or subjected to invasive procedures. In patients with hypoproliferative thrombocytopenia, the management of platelet transfusions remains somewhat empirical, derived from studies performed in hematology patients under stable clinical conditions. We herein described and analysed the determinants of post-transfusion platelet increments in cancer patients with hypoproliferative thrombocytopenia in the ICU.

**Patients and methods**: This was a single-center retrospective observational study over a 9-year period (2009–2017). Patients with malignancies and hypoproliferative thrombocytopenia who had received at least one platelet transfusion in the ICU were included. For each transfusion episode, a poor platelet yield was defined as a body surface area-adjusted corrected count increment (CCI) < 7, or alternatively as a weight-adjusted platelet transfusion yield (RTP) < 0.2. Patients were considered refractory to platelet transfusions when they experienced poor platelet increments (CCI < 7 or RTP < 0.2) following two consecutive ABO-compatible transfusions containing at least 0.5x1011 platelets per 10 kg bodyweight.

**Results**: 326 patients who received a total of 1470 platelet transfusions were analyzed. Indications for platelet transfusions were distributed into prophylactic (44.5%), securing an invasive procedure (18.1%) and therapeutic for active bleeding (37.4%). Transfusion thresholds were lower for prophylactic indications than for securing an invasive procedure or for therapeutic indications (13 [8–22] G L vs. 20 [13–31] G L vs. 21 [11–36] G L, respectively). Regardless of indications, 54.6% and 55.4% of transfusion episodes were associated with a CCI < 7 or a RTP < 0.2. Compared to prophylactic indications, the transfusion yields were better when securing an invasive procedure. In multivariate analysis, the factors associated with poor post-transfusion increments were lower body mass index (BMI), severity on the day of transfusion, depth of pre-transfusion thrombocytopenia, time between platelet transfusion and post-transfusion platelet count, fever ≥ 39 °C, antibiotic therapy, and storage duration of platelet concentrates. 48 patients developed refractoriness to platelet transfusion, associated with lower BMI, stem cell transplantation and spleen enlargement.

**Conclusion**: Platelet transfusions are often associated with poor increments in critically ill cancer patients with hypoproliferative thrombocytopenia. Our data suggest ways to improve the efficiency platelet transfusion in this setting.

### F-62 Differential Clinical Characteristics, Management, and Outcome Of Delirium in Ward and ICU Patients

#### Emmanuel Canet (*speaker*)^1^, Sobia Amjad^2^, Raymond Robbins^2^, Jane Lewis^2^, Michelle Matalanis^2^, Daryl Jones^2^, Rinaldo Bellomo^2^

##### ^1^Nantes, FRANCE; ^2^Austin Health Hospital, Melbourne, AUSTRALIA

###### **Correspondence:** Emmanuel Canet - emmanuel.canet@chu-nantes.fr

*Annals of Intensive Care* 2019, **9(Suppl 1)**:F-62

**Introduction**: To study patient demographics, clinical phenotype, management, and outcomes of patient with delirium in hospital wards compared to the ICU.

**Patients and methods**: Cohort of patients admitted to an Australian university-affiliated hospital between March 2013 and April 2017 and coded for delirium using the ICD-10 criteria.

**Results**: Among 61,032 hospitalized patients, 2,864 (4.7%) were coded for delirium. From these, we selected a random sample of 100 ward patients and 100 ICU patients for detailed analysis. Ward patients were older (median age- 84 vs. 65 years + P < 0.0001), more likely to have pre-existing neurological disease (53% vs. 13% for ICU patients + P < 0.0001) and less likely to have had surgery (24 vs. 62% + P < 0.0001). Of ward patients, 74% had hypoactive delirium, while 64% of ICU patients had agitated delirium (P < 0.0001). Persistent delirium at hospital discharge was more common among ward patients (66% vs 17%, p < 0.0001). On multivariate analysis, age and pre-existing neurological disease predicted persistent delirium, while surgery predicted recovery.

**Conclusion**: Delirium in ward patients is profoundly different from delirium in ICU patients. It has a dominant hypoactive clinical phenotype, is preceded by chronic neurological conditions, is managed with fewer drugs and is less likely to recover at hospital discharge.

### F-63 Traumatic quadriplegia- diagnostic and therapeutic strategy. (About 75 patients)

#### Amine Benhamed (*speaker*), Amel Zerhouni, Medjahed Medjahed, Lahcen Senhadji, Radouane Rachi

##### Faculté de medecine Oran, Oran, ALGERIA

###### **Correspondence:** Amine Benhamed - benhamedamine@hotmail.fr

*Annals of Intensive Care* 2019, **9(Suppl 1)**:F-63

**Introduction**: trauma to the spine is a common pathology that is constantly increasing in Algeria, mainly to road accidents, they are potentially serious, and associated with a spinal cord injury, they are life-threatening. We treated 75 traumatized cervical and thoracolumbar spine patients in our spine unit at CHU Oran.

**Patients and methods**: - Department of orthopedic and traumatological surgery- spine unit of CHU Oran. - Period- 24 months especially during the summer season. - Secondary support after the UAS. - Middle age- 38 years (14–81 years).

**Results**: 75 quadriplegic patients (58 men and 17 women) aged between 14 and 81 years, 60% of patients were between 14 and 60 years old, 27% between 40 and 60 years old and 13% over 60 years old, the main The causes of these traumatic quadriplegia are mainly due to AVP (67%), falls (27%) and sports accidents (judo, gymnastics) represent 6%.

Histopathological lesions are at C1-C2 level in 6 patients (8%) and C3-C7 in 69 patients (92%). clinically 36 patients (48%) already had complete quadriplegia at admission and 39 patients (52%) had incomplete tetraplegia. The neurological involvement was classified according to the FRANKEL classification. Our practical conduct was a decompression, arthrodesis, graft with a screwed plate. 30% of the patients benefited from a conservative treatment in a reduction by cranial traction. the course was marked by recovery in 8 patients (10.6%) who had incomplete tetraplegia and 13 patients (17.3%) died as a result of their complications.

**Discussion**: Traumatic quadriplegia is a major public health problem, few injuries are as devastating as those affecting the spinal cord; adult, youthful and adolescent men have the highest prevalence and suffer most of the time from a permanent deficit; quickly, the quadriplegic or paraplegic becomes aware of its deficit and its consequences. Hospitalization and rehabilitation, through their costs, represent a huge investment. The emotional damage the patient and his family are not measurable. After several weeks of treatment (surgery, resuscitation, rehabilitation ...) we found adverse results and treatment failure, which further complicated their insertion and care for their families.

**Conclusion**: Traumatic quadriplegia is especially aggravated during transport, the goal of surgery is to decompress the marrow and stabilize the spine.

### F-64 Prognostic significance of standard electroencephalography findings in adult patients with delayed awakening in the intensive care unit

#### Camille Legouy (*speaker*), Laura Girard-Stein, Lila Bouadma, Claire Dupuis, Sonia Abid, Camille Vinclair, Stéphane Ruckly, Ruben Wanono, Anny Rouvel-Tallec, Marie-Pia D’Ortho, Jean-François Timsit, Romain Sonneville

##### Hôpital Bichat-Claude Bernard, Paris, FRANCE

###### **Correspondence:** Camille Legouy - camille.legouy@gmail.com

*Annals of Intensive Care* 2019, **9(Suppl 1)**:F-64

**Introduction**: Despite daily interruption of sedative infusions, delayed awakening is frequently observed in critically ill patients requiring invasive mechanical ventilation. We aimed to identify the prognostic significance of standard electroencephalography findings in adult patients with delayed awakening in the intensive care unit.

**Patients and methods**: Our retrospective study included consecutive patients under invasive mechanical ventilation in the intensive care unit who underwent standard EEG because of delayed awakening. Delayed awakening was classified in 3 groups- coma, hypo-active delirium or hyperactive delirium according to RASS at inclusion. The primary endpoint was a good neurological outcome, defined as the proportion of patients alive and awake (i.e. responding to simple commands on 2 consecutive days) 7 days after EEG. Secondary endpoints included the prevalence of the different etiologies of delayed awakening, defined in 6 categories (hypoxic + metabolic + septic + antibiotic + sedation + acute brain injury) and the proportion of patients alive and awake at ICU discharge and at 90 days. Data are presented as median (interquartile range) or numbers (percentages). Cause-specific prevalence models were used to identify independent parameters associated with awakening and death, respectively.

**Results**: 121 patients (age 64 years [54 + 71], SAPS2 score of 61 [45 + 76]) with a RASS of -4 [-4 + -3] at inclusion were studied. At 7 days, 58 (48%) patients were awake, 40 (33%) were alive but not awake, and 23 (19%) were dead. In univariate analysis, the only parameter associated with awakening was RASS ≥ - 3, whereas parameters associated with mortality were a slow EEG background, a discontinuous EEG background and unreactive EEG background. Multivariate analysis revealed that discontinuous EEG background was associated with mortality (Table). By contrast, background frequency > 4 Hz associated with a preserved reactivity were protective. The etiologies of delayed awakening were- 65 (54%) sepsis, 49 (41%) hypoxia, 32 (27%) sedations, 25 (21%) neurotoxic antibiotics, 14 (12%) metabolic causes, with for some patients a multifactorial origin. Hypoxic encephalopathy was associated with short-term mortality. At the end of ICU stay, 60 (50%) patients were awake, 7 (6%) were alive but not awake and 53 (44%) were dead. At 90 days, 55 (45%) were awake, 6 (5%) were not awake and 60 (50%) were dead.

**Conclusion**: Delayed awakening in ICU is likely of multifactorial origin and characterized by a favorable outcome in about 50% of cases. Background EEG abnormalities (frequency, continuity) and reactivity provide major prognostic information on short-term mortality in this population.


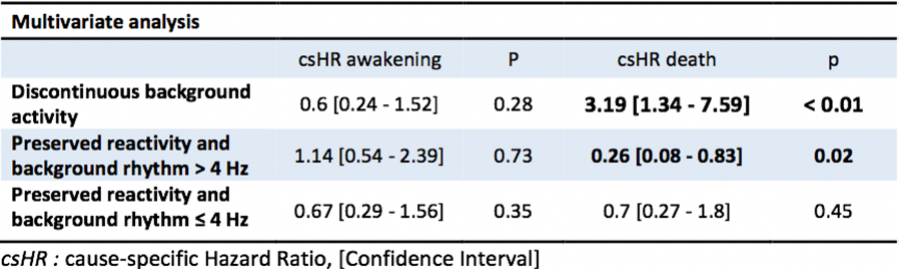


### F-65 Cardiac Arrest in Patients Managed for Convulsive Status Epilepticus- Characteristics, Predictors and Outcome

#### Stephane Legriel (*speaker*)^1^, Edouard Bresson^1^, Nicolas Deye^2^, David Grimaldi^3^, Bertrand Sauneuf^4^, Olivier Lesieur^5^, Jean-Baptiste Lascarrou^6^, Laurent Argaud^7^, Jonathan Chelly^8^, Pascal Beuret^9^, David Schnell^10^, Anne-Laure Chateauneuf^1^, Mathilde Holleville^1^, François Perier^1^, Virginie Lemiale^11^, Cédric Bruel^12^, Pierrick Cronier^13^, Nicolas Pichon^14^, Nicolas Mongardon^15^, Nicolas De Prost^15^, Florence Dumas^16^, Alain Cariou^16^

##### ^1^Centre Hospitalier de Versailles, Le Chesnay, FRANCE; ^2^Centre Hospitalier Universitaire Lariboisiere, Paris, FRANCE; ^3^Université Libre de Bruxelles (ULB), Erasme Hospital, Bruxelles, BELGIUM; ^4^Cotentin Public Hospital Center, Cherbourg-En-Cotentin, FRANCE; ^5^Centre Hospitalier Saint-Louis de la Rochelle, La Rochelle, FRANCE; ^6^Centre Hospitalier Départemental Vendée, La Roche-Sur-Yon, La Roche-Sur-Yon; ^7^Hospices civils de Lyon, Edouard Herriot Teaching Hospital, Lyon, FRANCE; ^8^Centre Hospitalier Marc Jacquet, Melun, FRANCE; ^9^Centre Hospitalier de Roanne, Roanne, FRANCE; ^10^Centre Hospitalier d’Angouleme, Angoulême, FRANCE; ^11^Centre Hospitalier Universitaire Saint Louis, Paris, FRANCE; ^12^Groupe Hospitalier Paris Saint-Joseph, Paris, FRANCE; ^13^Centre Hospitalier Paris Sud Francilien, Corbeil-Essonnes, FRANCE; ^14^Centre Hospitalier Universitaire de Limoges, Limoges, FRANCE; ^15^Centre Hospitalier Universitaire Henri Mondor, Créteil, FRANCE; ^16^Centre Hospitalier Universitaire Cochin, Paris, FRANCE

###### **Correspondence:** Stephane Legriel - slegriel@ch-versailles.fr

*Annals of Intensive Care* 2019, **9(Suppl 1)**:F-65

**Introduction**: Cardiac arrest (CA) is among the most catastrophic early complication seen during convulsive status epilepticus (CSE) management. Factors that may contribute to CSE-related CA (CSE-CA) include comorbidities, severe systemic complications (particularly in the event of uncontrolled seizure activity, injuries caused by the loss of consciousness and seizure, treatment complications, and cause of CSE. Although CSE-CA is an event of considerable concern, few studies have assessed its characteristics and long-term survival and functional outcomes. The objective of this retrospective study was to identify early factors associated with CA in adults managed for CSE and admitted to the intensive care unit (ICU). Knowledge of such factors might help to identify areas for improvement in the management of CSE.

**Patients and methods**: Retrospective multicenter study including consecutive patients admitted to 17 university or university-affiliated ICUs in France and Belgium for management of successfully resuscitated out-of-hospital cardiac arrest complicating the initial management of CSE between 2000 and 2015. Patients were compared with controls without CA identified in a single-center registry of CSE patients, regarding characteristics, management, and outcome.

**Results**: We included 49 cases with CSE-CA and 235 controls. In the cases, median time from medical team arrival to CA was 25 min [IQR, 5–85]. First recorded rhythm was asystole in 25 (51%) and pulseless electrical activity in 13 (27%) patients. A significantly larger proportion of patients had a favorable 1-year outcome (Glasgow Outcome Scale score of 5) among controls (90 235, 38%) than among cases (10 49, 21%, P = 0.02). By multivariate analysis, independent predictors of CA were pulse oximetry < 97% on scene (OR, 2.66 + 95%CI, 1.03–7.26, P = 0.04), drug poisoning as the cause of CSE (OR, 4.13 + 95%CI, 1.27–13.53, P = 0.02), and complications during early management (OR, 11.98 + 95%CI, 4.67–34.69, P < 0.0001). Having at least one comorbidity among cardiac, respiratory, and neurological (other than epilepsy) conditions predicted absence of CA (OR, 0.28 + 95%CI, 0.10–0.80, P = 0.02).

**Conclusion**: In patients managed for CSE, relative hypoxemia, on-scene management complications, and drug poisoning as the cause of CSE were strong early predictors of CA, suggesting areas for improvement.

### F-66 Feasibility and reliability of somatosensory evoked potentials performed by intensivists in the prognosis of post-cardiac arrest coma

#### Damien Bouvier (*speaker*), Quentin Levrat, Virginie Verrier, Olivier Lesieur

##### Hôpital Saint-Louis La Rochelle, Saint-Brice Sous Forêt, FRANCE

###### **Correspondence:** Damien Bouvier - damienbouvier.ar@gmail.com

*Annals of Intensive Care* 2019, **9(Suppl 1)**:F-66

**Introduction**: Post-cardiac arrest coma is a common cause of brain injury in the ICU. Predicting neurological outcome is of crucial importance to provide the most objective information to loved ones and opt for the best therapeutic options (including withholding or withdrawal of treatments deemed hopeless). Among the prognostic tools available, somatosensory evoked potentials have proven efficiency- under certain conditions, the absence of N20 cortical wave is associated with an unfavorable neurological prognosis with a specificity closed to 100% (1). Traditionally, this test requires the expertise and availability of a neurophysiologist. We assume that this technique can also be performed by trained intensivists.

**Patients and methods**: Two physicians from our ICU received two days of specific training in a university neurophysiology laboratory by a specialist in the interpretation of evoked potentials. Patients concerned had prolonged post-cardiac arrest coma after cessation of sedation. The records were interpreted and sent to the neurophysiology referral center for review by a specialist within 24 h. The feasibility and reliability of tests were evaluated retrospectively.

**Results**: From September 2011 to June 2018, somatosensory evoked potentials were recorded in 59 patients (64 [55–74] year old + M F ratio 3.9). Circulatory arrests were of cardiac origin in 42% of cases and respiratory in 46%. All patients underwent electroencephalography and had no reactivity to stimuli. Most of the recordings were made and interpreted easily by intensivists, with a similar conclusion by the neurophysiologist. Only one was doubtful and could only be assessed by the neurophysiologist. N20 wave was present in 26 patients and absent in 31 patients. Only two tests were uninterpretable.

**Conclusion**: Recording somatosensory evoked potentials in the ICU is simple, reliable, reproducible and can be performed by trained critical care physicians. However, its interpretation must be validated by a neurophysiologist given the implications in terms of therapeutic decisions.

### F-67 Sodium disturbances in the neuro-intensive care unit

#### Mariem Dlela (*speaker*), Manel Zekri, Rania Ammar, Aziza Talbi, Chokri Ben Hamida, Mounir Bouaziz

##### Hbib bourguiba university hospital, Sfax, TUNISIA

###### **Correspondence:** Mariem Dlela - mariem241090@gmail.com

*Annals of Intensive Care* 2019, **9(Suppl 1)**:F-67

**Introduction**: Sodium disturbances are the most common and probably the most poorly understood electrolyte disorders in neurological diseases. Complications can be minimized by better recognition, diagnosis, and treatment of sodium disorders. In this study, we aim to analyze the incidence, etiologies and impact of dysnatremia on brain damaged population, and we hypothesize that changes in sodium levels could be indicative of recent neurological deterioration.

**Patients and methods**: We conducted a six month long prospective cohort, including all brain damaged patients, who were admitted to our ICU between March 1st, 2018 and August 31st, 2018 and with a minimum length of stay (LOS) of 14 days. All patients, included, were screened for sodium disorders in the first 2 weeks of ICU stay. Outcome was measured by incidence of death, Glasgow outcome scale (GOS) on discharge and LOS. Patients were also monitored for neurological deterioration, including cognitive decline, convulsive seizures, increase in cerebral edema and brain herniation that were contemporary to sodium disorders. Both univariate and multivariate analysis were used to determine level of significance.

**Results**: During the study period, one hundred patients were admitted to our ICU for neuro-intensive care, among which 77 were included in this study. Patients were admitted for traumatic brain injury (TBI) in 75.3% of cases. According to our analysis, 35 (45.45%) patients presented with hyponatremia, 26 (74.3%) among them, were diagnosed with the syndrome of inappropriate antidiuretic hormone secretion (SIADH), 8 (22.9%) with corticosteroid deficiency and in one case with cerebral salt wasting syndrome. SIADH was attributed to convulsive seizures in 7(26.9%) cases, meningitis in 3(11.5%) cases and TBI in 11 (42.3%) cases. Hyponatremia was found to be a predictive factor of mortality in ICU (p = 0.022), of LOS (p = 0.032) and a sign of neurological deterioration (p = 0.03) on the day of diagnosis. Our study results’ showed an incidence of hypernatremia of 26% (20 cases), among which 55% (11 cases) were attributed to central diabetes insipidus. Hypernatremia was found to be a predictive factor of mortality in ICU (p < 0.00), of GOS (p < 0.00) and a sign of neurological deterioration (p < 0.00) on the day of diagnosis.

**Conclusion**: In summary, this study demonstrates that sodium disturbances are common in neuro-intensive care units and associated with increased ICU mortality. Besides it indicates that changes in sodium levels could be revealing of serious neurological complications.

### F-68 Characterisation of cardiovascular phenotypes in septic shock. Focus on LV systolic dysfunction and its impact on prognosis

#### Guillaume Geri (*speaker*)^1^, Philippe Vignon^2^, Alix Aubry^1^, Anne-Laure Fedou^2^, Cyril Charron^1^, Stein Silva^3^, Xavier Repesse^1^, Antoine Vieillard-Baron^1^

##### ^1^University Hospital Ambroise Paré, Boulogne-Billancourt, FRANCE; ^2^University Hospital, Limoges, FRANCE; ^3^University hospital, Toulouse, FRANCE

###### **Correspondence:** Guillaume Geri - guillaume.geri@aphp.fr

*Annals of Intensive Care* 2019, **9(Suppl 1)**:F-68

**Introduction**: Left ventricular (LV) systolic dysfunction is frequent in septic shock patients, but its prognostic impact remains unknown.

**Patients and methods**: Two published databases from 12 different ICUs including echocardiographic monitoring performed at the initial phase of septic shock were merged. Patients with a history of chronic heart failure or atrial fibrillation or dobutamine infusion at the time of echocardiography were excluded from the analysis. Hierarchical clustering in a principal components approach was used to define five cardiovascular phenotypes using haemodynamic, clinical and echocardiographic parameters. Missing data were imputed. The relationship between cluster and mortality (day-7 and ICU) was evaluated using a multivariable logistic regression.

**Results**: 324 patients (median age 64 [55, 74]) were included in the analysis. Five different clusters were individualised- patients well resuscitated (cluster 1, n = 76) without LV systolic function, right ventricular (RV) failure or fluid responsiveness, patients with LV systolic dysfunction (cluster 2, n = 41), patients with hyperkinetic profile (cluster 3, n = 70), patients with RV failure (cluster 4, n = 76), and patients with persistent hypovolemia (cluster 5, n = 61). Day-7 mortality was higher in cluster 2 than in the others (37 vs. 12, 13, 24 and 20%, in clusters 1, 3, 4, and 5, respectively, p = 0.04), while ICU mortality did not differ across clusters. In multivariable logistic regression, LV systolic dysfunction was independently associated with increased day-7 mortality (odds ratio 2.80 [95% confidence interval 1.05, 7.76]).

**Conclusion**: Among the five cardiovascular phenotypes individualised in this large cohort of septic shock patients, LV systolic dysfunction was independently associated with day-7 mortality.

### F-69 Early predictive factors of 30-days mortality in cardiogenic shock- An analysis of the FRENSHOCK multicenter prospective registry

#### Clément Delmas (*speaker*)^1^, Bruno Levy^2^, Nicolas Lamblin^3^, Nadia Aissaoui^4^, Etienne Puymirat^4^, Guillaume Leurent^5^, Vincent Labbe^6^, Sébastien Champion^7^, Stéphane Manzo-Silberman^8^, Meyer Elbaz^1^, Laurent Bonello^9^, Edouard Gerbaud^10^, Francois Roubille^11^, Eric Bonnefoy^12^, Patrick Henry^8^

##### ^1^Rangueil University Hospital, Toulouse, FRANCE; ^2^Intensive Care Unit, Vandoeuvre Les Nancy, FRANCE; ^3^Lille University Hospital, Lille, FRANCE; ^4^Hôpital Européen Georges Pompidou, Paris, FRANCE; ^5^Rennes University Hospital, Rennes, FRANCE; ^6^Tenon Hospital AP-HP, Paris, Paris; ^7^Parly II Clinic, Le Chesnay, FRANCE; ^8^Lariboisière University Hospital AP-HP, Paris, FRANCE; ^9^Aix-Marseille University Hospital, Marseille, FRANCE; ^10^Bordeaux University Hospital, Pessac, FRANCE; ^11^Montpellier University Hospital, Montpellier, FRANCE; ^12^Lyon University Hospital, Lyon, FRANCE

###### **Correspondence:** Clément Delmas - delmas.clement@chu-toulouse.fr

*Annals of Intensive Care* 2019, **9(Suppl 1)**:F-69

**Introduction**: Cardiogenic shock (CS) remains a severe but poorly understood pathology. Many predictive death scores have been previously described but have focused in ischemic CS and took into account data related to the management of these patients. So, there is an urgent need for simple and objective criteria to assess the short-term CS mortality regardless of the initial etiology.

**Patients and methods**: FRENSHOCK registry (NCT02703038) was a large prospective multicenter registry of CS patients admitted in intensive cardiac and general critical care units between April and October 2016 in France. Patients were prospectively included regardless of the CS etiology if they met at least one criterion of (1) low cardiac output (systolic blood pressure (SBP) < 90 mmHg and or the need of amines, or a low cardiac index < 2.2L min m2 on echocardiography or right heart catheterization + and (2) clinical, radiological, biological (NtproBNP or BNP), echocardiographical, or invasive hemodynamics overload signs + and (3) a clinical (oliguria, marbling, confusion) and or biological hypoperfusion (lactates > 2 mmol/L, hepatic and or renal failure). We studied factors related to 30d mortality using Kaplan–Meier analyses and Cox proportional hazards modeling.

**Results**: 772 patients were included (male 72%, median age 66yo). Non-ischemic CS were predominant (n = 491, 64%) although type 1 infarction was infrequent (n = 134, 17%). Mortality at 30-days was 26% (n = 201). Non survivors were older, had more previous renal failure, marbles, and atrial fibrillation at admission. They had lower SBP and DBP. Diagnostic tests revealed higher arterial lactate – CRP – natriuretic peptids – kaliemia + and lower pH - prothrombin time – hemoglobin – eGFR but also LVEF. Multivariate analysis retained age (especially > 75y), low systolic blood pressure (especially < 90 mmHg), high arterial lactate (especially > 4 mmol/L), low eGFR (especially < 30 ml/min/m_2_), low LVEF (especially < 30%) as significant predictors of 30-days mortality. Ischemic etiology or type 1 infarction were not predictive.

**Conclusion**: Our multicentric and prospective design confirmed the heterogeneity of CS in terms of presentation and prognosis. Five simple, practical and easy to find signs were found significant predictors of short term mortality and could be useful in providing a more accurate and stratified definition of CS’s patients in order to tailor additional therapies.


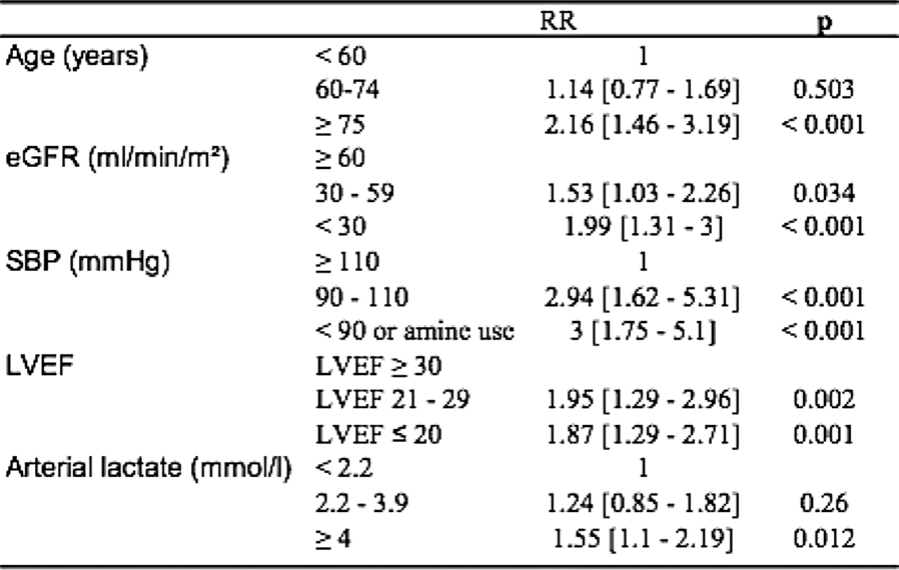


### F-70 Incidence, predisposing factors and prognosis of acute postoperative Right ventricular failure in cardiac surgery- a prospective cohort study

#### Ahlem Trifi (*speaker*)^1^, Imen Ben Naoui^2^, Sami Abdellatif^3^, Adel Ammous^2^, Raouf Denguir^4^, Mohamed Sami Mourali^5^, Salah Ben Lakhal^6^

##### ^1^Faculty of Medicine of Tunis, Tunis, TUNISIA; ^2^Anaesthesia and Surgical Intensive Care department, La Rabta Hospital, Tunis, TUNISIA; ^3^Medical intensive care unit. La Rabta Hospital, Tunis, TUNISIA; ^4^Cardiovascular surgery service. La Rabta Hospital, Tunis, TUNISIA; ^5^Department of Functional Investigations and Cardiac intensive care unit. la Rabta hopital, Tunis, TUNISIA; ^6^Medical intensive care unit. La Rabta Hospital, Tunis, TUNISIA

###### **Correspondence:** Ahlem Trifi - trifiahlem2@gmail.com

*Annals of Intensive Care* 2019, **9(Suppl 1)**:F-70

**Introduction**: Acute postoperative cardiac surgery (POCS) right ventricular failure (RVF) is uncommon and worsened the patient’s prognosis. We aimed to study the incidence, risk factors and outcome of acute RVF in cardiac surgery under extracorporeal circulation (ECC) patients.

**Patients and methods**: a prospective cohort study over one year (December 2016-December 2017). Were included, patients candidates for cardiac surgery (CS) with extra corporeal circulation and having a normal RF systolic function. Transthoracic-echocardiography (TTE) Doppler was performed on day 1, day 3, day 7 and 1 postoperative month. TAPSE < 13 mm and an S-wave velocity < 10 cm s during the first postoperative week defined the POCS-RVF. Thus, patients were divided into two groups (POCS-RVF group versus non POCS-RVF group) and compared. Outcomes were- catecholamine support, septic events, length of stay (LOS), ventilator days and 30-day mortality.

**Results**: 128 among 131 patients were included (POCS-RVF group, n = 49 versus non POCS-RVF, n = 79). The incidence of acute POCS-RVF was 38.2%. Acute RVF occurred at the 1st post operative day and remained during 30 days (attached fig). Mitral valve replacement, aortic clamping time above than 90 min, preoperative arrhythmia and bleeding were significantly related to acute POCS-RVF with respectively (OR = 11.75 + IC [2.18–13.16]), (OR = 4.36 + IC [1.01–18.68]), (OR = 6.55 + IC [2.38–17.96]), (OR = 3.4 + IC [2.38–17.96]). Acute POCS-RVF increased mortality [21(43%) vs 16 (20%), p = 0.006] and reduced survival time by 5 days but no significant link was showed between POCS-RVF and death. It depended to the left ventricular (LV) systolic function. LV dysfunction in POCS-RVF patients increased the death risk by 3 and its absence improved survival. Other factors were significantly associated to mortality Bentall and coronary tube procedures and ECC time > 120 min.

**Conclusion**: the incidence of acute POCS-RVF is not negligible and several preoperative factors predispose to this phenomenon. LV failure worsened the outcome. These findings should sustain preventive measures to limit myocardial damage during cardiac surgery.


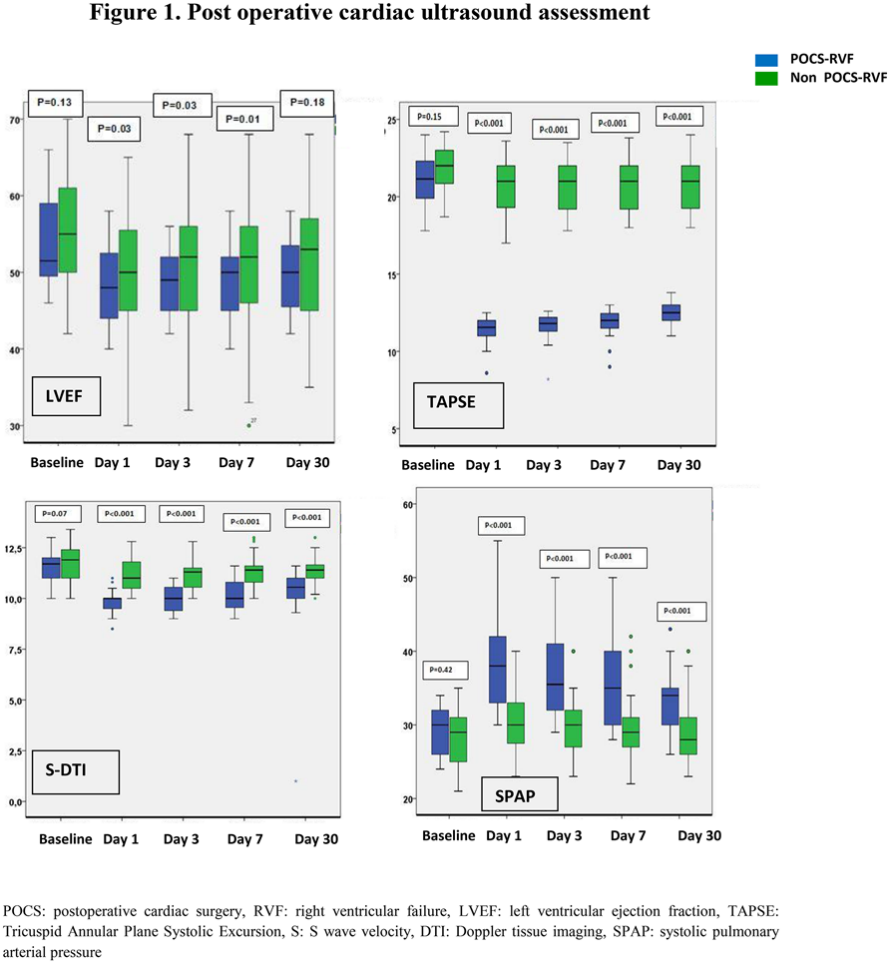


### F-71 High risk of Chronic Kidney Disease after VA-ECMO - Results of one year follow-up of a monocentric cohort of 132 patients

#### Camille Vinclair (*speaker*)^1^, Romain Sonneville^1^, Jean Reuter^1^, Radj Cally^1^, Mathilde Neuville^1^, Jordane Lebut^1^, Claire Dupuis^1^, Stéphane Ruckly^2^, Jean-François Timsit^1^, Lila Bouadma^1^

##### ^1^HUPNVS - Hôpital Bichat - Claude-Bernard, Paris, FRANCE; ^2^UMR 1137 - IAME Inserm. Paris-Diderot University, Paris, FRANCE

###### **Correspondence:** Camille Vinclair - cvinclair@live.fr

*Annals of Intensive Care* 2019, **9(Suppl 1)**:F-71

**Introduction**: Veno-Arterial Extra-Corporeal Membrane Oxygenation (VA-ECMO) is a life support technique associated with a major incidence of acute kidney injury (AKI). Risk of chronic kidney disease (CKD) following AKI is high. The objectives of the study were to describe renal natural history within one year following VA-ECMO and to identify early predictors of long-term renal impairment.

**Patients and methods**: We retrospectively analyzed consecutive adult patients without preexisting end-stage renal disease (ESRD) who received VA-ECMO for more than 48 h in the 20-bed medical ICU of a university hospital, in Paris, France, between January 2014 and December 2016. AKI severity during ICU stay was defined according to KDIGO classification. Renal function at 1 year was assessed with estimated glomerular filtration rate (eGFR) using the MDRD equation. The primary endpoint was a composite of poor renal outcome (eGFR ≤ 60 mL/min 1.73m2) or death at one year, defining a bad outcome. Factors associated with bad outcome were identified by multivariate logistic regression analysis. Quantitative variables are reported as median (interquartile range, IQR) and qualitative variables as numbers (percentage). Results of multivariate analysis are reported as odds-ratio (OR) and 95% confidence interval.

**Results**: 132 patients with available 1-year follow-up (male sex 75.5%, age 58 [46 + 66] years, SAPS II 55[38 + 67], SOFA 9 [6 + 12], time on ECMO 7.5 [4 + 12] days) were included in the study. 72 (54%) patients died in ICU and 80 (60.6%) within a year. 121 (92%) patients developed AKI during ICU stay, 73 (55%) required renal replacement therapy. 38 (74%) of survivors had an abnormal decline of eGFR at one year- median decline rate was 30 [18 + 55] mL/min 1.73m2. Four (3%) patients had ESRD at one year, three of them did not required RRT during ICU but experienced recurrent AKI afterwards. In multivariate analysis, a best baseline renal function was protective of a bad renal outcome at one year (OR = 0.981 for a higher eGFR, [0.966 + 0.996], p = 0.01). Severity of AKI in ICU was significantly associated with one year renal outcome. (OR = 67.190 [6.48 + 697] p < 0.01 for KDIGO stage 3).

**Conclusion**: Among survivors of VA-ECMO therapy, long-term renal impairment is major particularly in those with previous CKD and severe AKI during ICU stay. ESRD is rare and occurs in patient with recurrent AKI.

**Table:** 

Parameter	**OR**	**95% CI**		***p***
Baseline eGFR	0.981	0.966	0.996	0.0128
AKI KDIGO stage at canulation time				
No AKI (ref)	1			0.8252
Stage 1	0.904	0.166	4.913	0.9070
Stage 2	0.728	0.091	5.821	0.7651
Stage 3	0.385	0.047	3.118	0.3708
Worst AKI KDIGO stage in ICU				
No AKI (ref)	1			0.0052
Stage 1	4.396	0.662	29.189	0.1253
Stage 2	4.428	0.762	25.732	0.0975
Stage 3	67.190	6.477	696.951	0.0004
SOFA without hemodynamic and renal score	0.820	0.650	1.034	0.0930
Indication of ECMO				
Medical cause	4.177	0.842	20.728	0.0802
Post-operative with CBP < 140 min	0.889	0.159	4.977	0.8931
Post-operative with CBP ≥ 140	1			0.0834
Number of RBC transfusion	1.041	0.983	1.104	0.1708
Activated Partial Thromboplastin Time	1.727	0.732	4.075	0.2120

### F-72 Impact of levosimendan on peripheral veno-arterial extracorporeal membrane oxygenation weaning in intensive care unit

#### Shamir Vally, Cyril Ferdynus, Romain Persichini, Eric Braunberger, Hugo Lo Pinto, Bruno Bouchet, Olivier Martinet , Thomas Aujoulat, Jérôme Allyn, Nicolas Allou

##### CHU Félix Guyon, Saint-Denis, FRANCE

###### **Correspondence:** Shamir Vally- sham.vally@hotmail.fr

*Annals of Intensive Care* 2019, **9(Suppl 1)**:F-72

**Introduction**: Few data are available concerning the impact of levosimendan in patients with refractory cardiogenic shock supported by peripheral veno-arterial extracorporeal membrane oxygenation (VA-ECMO). The aim of this study was to evaluate impact of levosimendan on VA-ECMO weaning in patients hospitalized in intensive care unit (ICU).

**Patients and methods**: This retrospective cohort study was conducted in a French university hospital, in one ICU from 2010 to 2017. All patients hospitalized in ICU who underwent VA-ECMO were consecutively evaluated.

**Results**: A total of 150 patients with VA-ECMO were eligible for the study. A propensity score matched 38 patients in the levosimendan group and 65 patients in the non-levosimendan group. In patients treated with levosimendan, 24 h after infusion of medication left ventricular ejection fraction increased from 21.5 ± 9.1% to 30.7 ± 13.5% (P < 0.0001) while aortic velocity–time integral increased from 8.9 ± 4 cm to 12.5 ± 3.8 cm, (P = 0.002). After matching on propensity score, levosimendan was the only remaining factor associated with a significant reduction of VA-ECMO weaning failure (Hazard Ratio = 0.16, 95%confidence interval- 0.04–0.7, P = 0.01). Kaplan–Meier survival curves showed that the survival rates at 30 days were 78.4% in the levosimendan group and 49.5% in the non-levosimendan group (P = 0.0.37). However, no significant difference was found between the levosimendan and non-levosimendan groups regarding 30 days mortality after propensity score analysis (Hazard Ratio = 0.55, 95% confidence interval- 0.27–1.10, P = 0.09).

**Conclusion**: This study suggests that levosimendan could be associated with a beneficial effect on VA-ECMO weaning in ICU patients. However, the use of levosimendan tended to decrease 30 days mortality after propensity matched analysis (P = 0.09).

### F-73 Serial neuron specific enolase (NSE) serum levels and neurologic outcome of cardiogenic shock patients treated by venoarterial extracorporeal membrane oxygenation (ECMO)

#### Jean Reuter (*speaker*)^1^, Katell Peoc’H^2^, Lila Bouadma^1^, Dorothée Faille^1^, Marie-Charlotte Bourrienne^1^, Claire Dupuis^1^, Eric Magalhaes ^3^, Sébastien Tanaka^1^, Camille Vinclair^1^, Etienne De Montmollin^1^,Nadine Ajzenberg^1^, Jean-François Timsit^1^, Romain Sonneville^1^

##### ^1^HUPNVS - Hôpital Bichat - Claude-Bernard, Paris, FRANCE; ^2^HUPNVS - Hôpital Beaujon, Clichy, FRANCE; ^3^Centre Hospitalier Sud Francilien, Corbeil-Essonnes, FRANCE

###### **Correspondence:** Jean Reuter - reuterjang@gmail.com

*Annals of Intensive Care* 2019, **9(Suppl 1)**:F-73

**Introduction**: Cardiogenic shock patients treated with venoarterial ECMO (VA-ECMO) may develop brain injury during ECMO support. We aimed to assess the predictive value of serial measurements of neuron specific enolase (NSE) to identify poor neurologic outcome and CT-defined acute brain injury in this setting.

**Patients and methods**: We conducted a prospective cohort study in consecutive adult patients cannulated with VA-ECMO for refractory cardiogenic shock in the medical ICU of a university hospital in Paris, France. Plasma was sampled at predefined time points, 1 day, 3 days and 7 days after VA-ECMO cannulation, until ECMO removal or death. Plasma samples were collected and stored at − 80 °C. The primary endpoint was poor outcome, a composite endpoint of CT-defined brain injury or death 28 days after VA-ECMO cannulation. The secondary endpoint was CT-defined brain injury. Plasma NSE levels were measured at the end of study. Data are presented as median (interquartile range) or number (percentages). The association between NSE levels and outcome was explored by multivariate logistic regression analysis, with NSE levels being dichotomized according to median values at day 1 and day 3.

**Results**: A total of 104 patients (males (n = 67, 64%)) with a SOFA score at admission of 11 (8–14) were included, of whom 26 (25%) underwent cardiopulmonary resuscitation before VA-ECMO cannulation. At VA-ECMO cannulation, all patients were mechanically ventilated, 83 (80%) were sedated, and 81 (78%) were receiving vasopressors. Plasma NSE levels were 36 (26–50) µg L at day 1, 25 (19–38) µg L at day 3 and 22 (16–31) µg L at day 7. A poor outcome occurred in 56 (53%) patients and CT-defined brain injury was observed in 16 45 (36%) patients.

Plasma NSE levels at day 1 and day 3 were associated with poor outcome in crude analyses. In multivariate analysis, only NSE levels at day 3 remained independently associated with a poor outcome (table). In patients who underwent brain CT during VA-ECMO support, both NSE levels at day 1 and day 3 were associated with CT-defined brain injury.

**Conclusion**: In cardiogenic shock patients treated by VA-ECMO, plasma NSE levels measured 3 days after VA-ECMO initiation are independently associated with short term acute brain injury or death, irrespective of pre-ECMO characteristics. Patients with persistent elevated NSE levels 3 days after VA-ECMO initiation may benefit from advanced neuromonitoring while on ECMO support.

**Table:** 

Variable	Odds ratio	95% CI	Odds ratio	95% CI
	(univariate analysis)		(multivariate analysis)	
Age	1.03	(1.00–1.06)	1.01	(0.97–1.05)
CPR before ECMO cannulation	3.01	(1.14–7.97)	1.25	(0.34–4.57)
SOFA at ICU admission	1.13	(1.02–1.25)	1.10	(0.94–1.29)
SOFA at ECMO initiation	1.21	(1.07–1.36)	1.16	(0.97–1.39)
Day-3 NSE > 25 mg/L	4.29	(1.60–11.49)	**4.72**	**(1.58**–**14.11)**

### F-74 Impact of hyperoxia on patients hospitalized in intensive care unit for pulmonary congestion due to acute heart failure

#### Julien NaËl (*speaker*)^1^, Mathilde Ruggiu^1^, Clotilde Bailleul^1^, Sofia Ortuno^1^, Jean-Luc Diehl^1^, Damien Vimpere^1^, Aymeric Lancelot ^1^, Amélie Couteau^1^, Emmanuel Guerot^1^, Nicolas Danchin^2^,Etienne Puymirat^2^, Nadia Aissaoui^3^

##### ^1^HEGP Critical care, Paris, FRANCE; ^2^HEGP Department of cardiology, Paris, FRANCE; ^3^HEGP- AP-HP-Université Paris Descartes, Paris, FRANCE

###### **Correspondence:** Julien NaËl - julien.nael@gmail.com

*Annals of Intensive Care* 2019, **9(Suppl 1)**:F-74

**Introduction**: Oxygen therapy (OT) remains a cornerstone of acute heart failure (AHF) therapy in patients with pulmonary congestion (PC). While avoiding hypoxemia has long been a goal of critical care practitioners, less attention has been paid to the potential hazard related to excessive oxygenation and or hyperoxia. Recent studies highlighted the uselessness or the potential hazard of hyperoxia in patients admitted for acute medical emergencies. Our main objective was to evaluate the impact of an early hyperoxia exposure among critically ill patients hospitalized for AHF.

**Patients and methods**: In this observational, retrospective study led in a Parisian medical intensive care unit (ICU), we assessed AHF patients admitted for PC from 01 01 2015 to 12 31 2016. Patients with cardiac arrest, severe chronic obstructive pulmonary disease, and long-term OT were not included. Hyperoxia was defined as a PaO2 > 100 mmHg on blood gaz analysis. The hyperoxia group was defined by having at least one PaO2 > 100 mmHg the first day following the ICU admission. The principal endpoint was a 30-day composite one combining all-cause mortality and unplanned hospital admission. The secondary endpoints were occurrence of a pneumonia bacteriemia, ICU length of stay (LOS) and hospital LOS. Multivariate analysis was performed to determine if hyperoxia was independent risk factor of 30-day mortality.

**Results**: Among the 1541 patients admitted in ICU during the period study, 75 patients with PC due to AHF were included. Forty-one patients (54.7%) required mechanical ventilation. During the first 24 h, 43 patients (57.3%) presented at least one hyperoxia on ABG [the hyperoxia group (H)] whereas 32 patients (42.7%) did not [the control group (C)]. The baseline characteristics according to the two groups did not differ [Table 1]. The composite primary endpoint did not differ between the two groups (27.9% vs 21.8%, P = 0.85). 30-day mortality was 14% in H versus 12.5% in C, P = 0.85. 30-day unplanned hospital admission was increased in H (16.3%) compared to C but it did not reach the significance (P = 0.21). The secondary endpoints were not significantly different between the two groups [Table 1]. In multivariate analysis, hyperoxia was not associated with 30-day mortality [OR = 0.44(95%CI- 0.14–1.40), P = 0.44].

**Conclusion**: Hyperoxia is not useful in critically ill patients with AHF but its benefit on the short-term outcome remains to demonstrate.

**Table:** Table 1. Baselines characteristics and Outcomes

	Total patients (n = 75)	Hyperoxia group n = 43	Control group n = 32	P
Age, (years)	76 [68–83]	74 [64–85]	76.5 [65–88]	0.30
Male sex	38 (51)	25(58)	13 (41)	0.9
Supraventricular arythmia	42 (56)	20 (46.5)	22 (69)	0.42
Chronic lung disease	14 (19)	3 (7.0)	11 (34)	< 0.001
Chronic kidney failure	23 (31)	11 (26)	12 (37.5)	0.78
Ischemic cardiopathy	33 (44)	23 (53.5)	10 (31)	0.69
Ejection Fraction, (%)	50 [47–54]	49 [44–53]	49 [44–53]	0.11
SOFA Score	5 [4–6]	4 [3–7]	5 [3–7]	0.20
Mechanic ventilation				
- Mechanical Ventilation	40 (53.3)	23 (53)	17 (53)	0.96
- Non-invasive ventilation	28 (37.3)	13(30)	15 (47)	
- Invasive ventilation	12 (16)	10 (23)	2 (6)	
Duration of mechanical ventilation, (days)	2 [1–3]	2 [1–3]	2 [0.6–3.4]	0.37
Medications				
- Inotropic agents	11 (15)	9 (21)	2 (6)	< 0.01
- Diuretics	59 (79)	35 (81)	24 (75)	0.90
- Vasodilators	26 (35)	16 (37)	10 (31)	0.92
Dialysis	7 (9)	3 (7)	4 (12.5)	0.76
Duration of inotrope infusion, (days)		0.6 [0.2–1]	0.6 [0.2–1.4]	0.47
Duration of dialysis, (days)	3	NA	NA	
Infection occurence	12 (16)	6 (14)	6 (19)	0.12
LOS in ICU, (days)	4.0 [2.4–7.3]	3.3 [2.0–6.9]	4.5 [3.4–7.8]	0.16
LOS in hospital, (days)	12.2 [7.5–17.5]	12.2 [7.5–16.3]	12.1 [8.3–17.6]	0.20
30-day mortality	10 (13.3)	6 (14)	4 (12.5)	0.85
30-day unplanned hospital admission	10 (13.3)	7 (16.3)	3 (9.3)	0.21
30-day death and/or unplanned hospital admission	20 (26.7)	13 (27.9)	7 (21.8)	0.65

Abbreviations: AF = Atrial fibrillation, SOFA = Sequential Organ Failure Assessment , LOS = length of stay

Values are median [Q1–Q3] or n (%)

### F-75 Does having multiple causes of hypoxemia increase hypoxemia severity ? An ancillary study of the SPECTRUM study

#### David Grimaldi (*speaker*)^1^, Sami Hraiech^2^, Florence Boissier^3^, Philippe Michel^4^, Jean-Claude Lacherade^5^, Tai Pham^6^, Jean-Christophe Richard ^7^, Arnaud Thille^3^, Stephan Ehrmann^8^, Nadia Aissaoui^9^,Gregoire Muller^10^

##### ^1^CHUB Hôpital Erasme, Bruxelles, BELGIUM; ^2^Hôpital Nord AP-HM, Marseille, FRANCE; ^3^CHU, Poitiers, FRANCE; ^4^CH, Pontoise, FRANCE; ^5^CH, La Roche Sur Yon, FRANCE; ^6^St Michael’s hospital, Toronto, CANADA; ^7^Hôpital de la Croix Rousse, Lyon, FRANCE; ^8^CHU, Tours, FRANCE; ^9^HEGP-APHP, Paris, FRANCE; ^10^CH, Orléans, FRANCE

###### **Correspondence:** David Grimaldi - david_grimaldi2001@yahoo.fr

*Annals of Intensive Care* 2019, **9(Suppl 1)**:F-75

**Introduction**: In ICU patients several causes mechanisms of hypoxemia can be present. However the cumulative effect of multiple causes of hypoxemia is unknown. Using the data from a point-prevalence-day study on hypoxemia, we analyzed the relationship between the number of hypoxemia causes and hypoxemia severity.

**Patients and methods**: The SPECTRUM study was conducted in 117 ICUs in 7 countries during spring 2016. We collected the presence of 23 causes mechanisms of hypoxemia based on investigators judgment. We compared hypoxemia causes according to the class of hypoxemia. We classified the patients in 4 groups according to the number of causes (0, 1, 2 and > 2) and analyzed if the number of conditions was associated with hypoxemia severity and ICU-mortality using univariate followed by multivariate analyses.

**Results**: Among the 859 hypoxemic patients included the day of the study, 853 could be analyzed. The median number of hypoxemia causes was 2 (1–3). 107 (12%) patients had no cause of hypoxemia recorded, 230 (27%) had 1 cause, 221 (26%) had 2 causes and 295 (35%) had 3 or more causes. Main causes were pneumonia (53%), fluid overload (33%), pleural effusion (23%), atelectasis (21%), acute on chronic respiratory failure (19%). Repartition of causes differ across the 3 class of hypoxemia severity.

Patients with more hypoxemia causes had more often chronic respiratory, heart and renal failure, had a higher SAPS-2 and were more often under vasopressors, (p < 0.05 for all). Patients with more causes of hypoxemia were more invasively ventilated (p < 0.001) and the number of causes was associated with hypoxemia severity (p < 0.001). After multivariate linear regression, number of causes of hypoxemia was negatively associated with P/F (coeff -3 (95%CI -6 + 0), p = 0.05). ICU-mortality across the 4 groups was 16, 21, 27 and 35% (p < 0.001). Multivariate logistic regression showed that in addition to age, chronic heart failure, SAPS-2, admission diagnosis, P/F < 100, and ARDS, having > 2 causes of hypoxemia was associated with ICU-mortality (OR 2 (95%CI- 1.03–3.8)), whereas obesity was protective.

**Conclusion**: Over 60% of hypoxemic patients had 2 or more causes of hypoxemia. The number of causes of hypoxemia was independently associated with a lower P/F ratio. Having 3 or more causes of hypoxemia was independently associated with ICU-mortality. Preventing patients to acquire supplementar conditions that induce hypoxemia may improve their prognosis.

### F-76 Recruitment maneuver use in ARDS patients and mortality, a systematical review and meta-analysis

#### Joris Pensier (*speaker*), Audrey De Jong, Gérald Chanques, Nicolas Molinari, Samir Jaber

##### Département d’Anesthésie-Réanimation B - CHRU St Éloi, Montpellier, France

###### **Correspondence:** Joris Pensier - pensier.joris@gmail.com

*Annals of Intensive Care* 2019, **9(Suppl 1)**:F-76

**Introduction**: Acute respiratory distress syndrome (ARDS) is a common injury in intensive care units (ICU) patients, which is worsened by atelectasis. Lung recruitment maneuver (LRM) aims to open collapsed alveoli, increasing airway pressure for a short duration. Single studies of LRM’s use for ARDS patients in ICU, along with recent meta-analyses, have shown discrepancies regarding prognosis of ARDS patients. Two recent RCTs have been published, entailing around 1300 new patients. The aim of this study was to critically review the literature to investigate whether LRM reduces mortality in ARDS patients compared to a no-LRM strategy.

**Patients and methods**: We performed a systematic review and meta-analysis of randomized controlled trials (RCTs), by searching PubMed, CENTRAL, Web of Science and bibliographies of articles retrieved, among data from 1998 to 2018. We screened for relevant studies that enrolled ARDS adult patients. We included studies reporting mortality to perform a meta-analysis. We generated pooled relative risks (RR) across studies. The primary outcome was 28-day mortality. The secondary outcomes were ICU mortality and 60-day mortality.

**Results**: Eight RCTs with a total of 2,735 participants (1297 in LRM group and 1438 in No-LRM group) were included in the analysis (table 1). Compared to a No-LRM strategy, LRM did not reduce the 28-day mortality (RR = 0.90 (95% Confidence Interval (CI) 0.75–1.08, p = 0.26)). A significant heterogeneity was observed (p < 0.03, I2 = 52%). A sensitivity analysis excluding a study which did not use protective ventilation in control group showed similar results (RR = 0.96 (CI95% 0.83–1.12, p = 0.62)). No significant difference was found on mortality in the ICU (6 studies, 2412 patients, RR = 0.87 (CI95% 0.71–1.06, p = 0.16)). A significant heterogeneity was observed (p = 0.02, I2 = 63%). A sensitivity analysis excluding a study which did not use protective ventilation in control group showed similar results (5 studies, 2359 patients, RR = 0.93 CI95% 0.78–1.10, p = 0.40). No significant difference was found on mortality at day 60 (3 studies, 559 patients, RR = 0.92 (CI95% 0.71–1.19, p = 0.51)). No heterogeneity was found for this outcome (p = 0.84, I2 = 0%).

**Conclusion**: These results suggest that systematical LRM does not provide a significant benefit regarding mortality in ARDS. However, heterogeneity between studies was high and remains to be further explored.


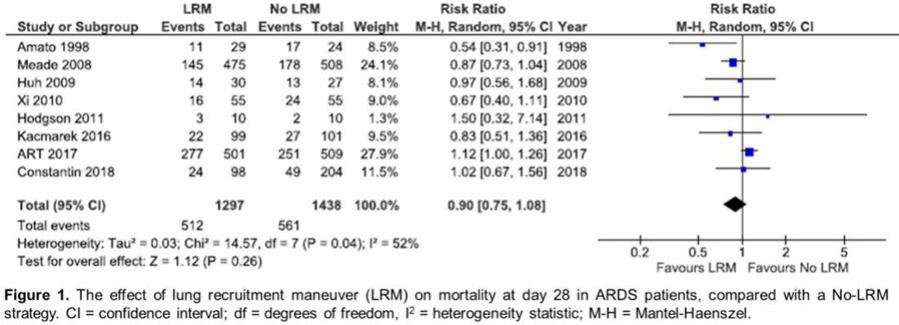


### F-77 Assessment of electrical impedance tomography to set optimal positive end-expiratory pressure for veno-venous ECMO-treated severe ARDS patients

#### Floriane Puel (*speaker*), Christelle Soulé, Fanny Bounes Vardon, Thierry Seguin, Vincent Minville, Bernard Georges, Jean-Marie Conil, Laure Crognier

##### CHU Rangueil, Toulouse, FRANCE

###### **Correspondence:** Floriane Puel - puel.f@chu-toulouse.fr

*Annals of Intensive Care* 2019, **9(Suppl 1)**:F-77

**Introduction**: In patients with severe acute respiratory distress syndrome (ARDS) on extracorporeal membrane oxygenation (ECMO) and ventilated with very low tidal volume, the optimal positive end-expiratory pressure (PEEP) should combine the best alveolar recruitment with minimal overdistension. Electrical impedance tomography (EIT) provides a non-invasive, real-time, bedside imaging of the lungs. The aim of our study was to assess EIT’s ability to choose the best PEEP in patients on ECMO receiving ultra-protective mechanical ventilation.

**Patients and methods**: ARDS patients on veno-venous ecmo were included. A written information was given to all patients’ relatives before inclusion. A recruitment maneuver and a decremental PEEP trial from 20 to 5 cmH20 (in 5 cmH20 steps) were monitored by EIT, with lung images divided into four ventral-to-dorsal horizontal regions of interest. Driving pressure was maintained constant at 10cmH2O. For each patient, four EIT-based PEEP were defined- PEEP ODCLmin (pressure with the lowest EIT-assessed collapse lung [CL] and overdistension [OD]), PEEP ODCL15 (the lowest pressure able to limit EIT-assessed collapse below 15% with the least overdistension), PEEP Comp (PEEP with the EIT-based compliance maximum), and PEEP GI (PEEP with EIT-based global inhomogeneity (GI) index minimum). The concordance between these four EIT-based PEEP and the reference pulmonary PEEP P (defined according the respiratory clinical and ultrasound usual parameters) was evaluated by the Cohen’s kappa coefficient.

**Results**: In fourteen patients, the decremental PEEP trial induced a decrease in tidal impedance variation (TIV) of dependent regions, and an increase in TIV of non-dependent lung regions (Figure 1). High PEEP levels were significantly associated with more overdistension (Rho = 0.908 [0.848–0.945]) and fewer collapsed zones, while decreasing PEEP led to more collapsed zones (Rho = − 0.909 [− 0.946 to − 0.849]). The PEEP ODCL15 and the PEEP Comp were in successful agreement with the reference PEEP P (respectively 0.714 [0.363–1] and 0.714 [0.348–1]). The PEEP ODCLmin was in medium agreement with PEEP P (0.571 [0.146–0.997]), and PEEP GI was in disagreement (− 0.0833 [− 0.218–0.0517]).

**Discussion**: EIT allows to individually study the effects of PEEP levels during ultra-protective ventilation on ECMO with EIT-based overdistension, collapse, and compliance.

**Conclusion**: EIT may be an interesting non-invasive bedside tool to provide real-time monitoring of PEEP impact in severe ARDS patients under ECMO.

No conflict of interest.


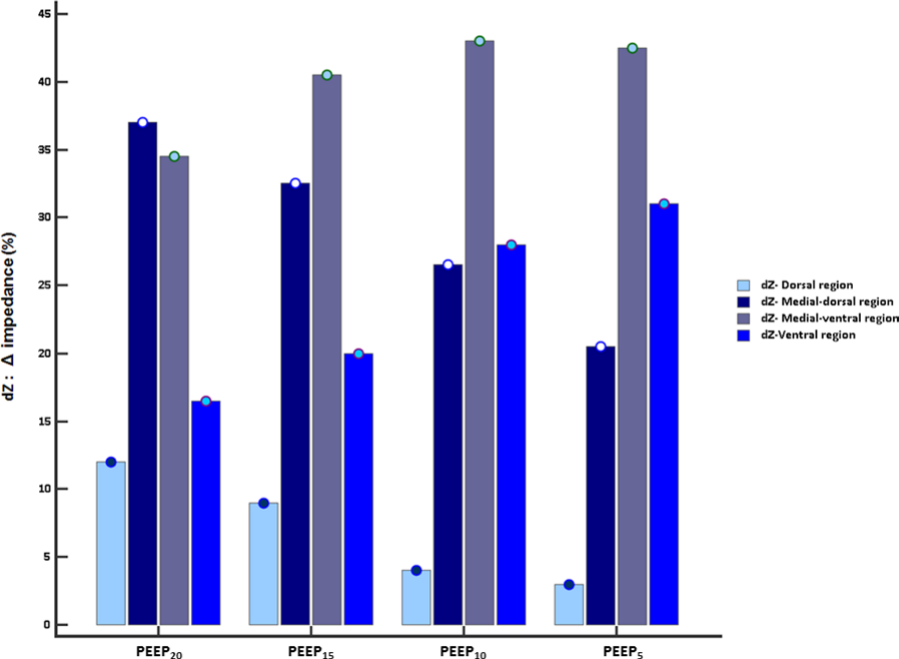


### F-78 Prone position is feasible with an extracorporeal decarboxylation during adult’s acute respiratory distress syndrome. Analysis of 58 sessions

#### Nicolas Boquillon (*speaker*), Jean François Georger

##### CHI Villeneuve Saint Georges, Villeneuve-Saint-Georges, FRANCE

###### **Correspondence:** Nicolas Boquillon - nicolas.boquillon@mailoo.org

*Annals of Intensive Care* 2019, **9(Suppl 1)**:F-78

**Introduction**: Toxicity of mechanical ventilation during acute respiratory distress syndrome (ARDS) can possibly be prevented by using “ultra-protective” ventilation which favors hypercapnia. An extracorporeal removal of CO2 (ECCO2R) can correct it. The issue of hypoxemia remains for which prone positioning (PP) is known to be effective. We therefore studied PP feasibility with ECCO2R.

**Patients and methods**: We conducted a retrospective analysis from August 2014 to December 2017 in our center. Indication for ECCO2R was a pH < 7.20 with an arterial partial pressure of CO2 > 50 mmHg with “protective ventilation”. Indication for PP was a PaO2 FiO2 ratio (arterial partial pressure of O2 fraction of inspired O2) < 150. Primary endpoint was discontinuation before 15 h of PP. Patients with ARDS (Berlin’s criteria) and ECCO2R were compared to patients without ECCO2R with Fischer’s exact test.

**Results**: 23 patients with ECCO2R underwent 58 PP procedures whose 56 lasted more than 15 h. 38 patients without ECCO2R underwent 53 PP procedures whose 47 lasted more than 15 h. There was no difference for early discontinuation of PP (p = 0.15).

**Discussion**: Our results were in accordance with scientific evidence and did not advocate for a change of practice.

**Conclusion**: It was possible to safely conduct 58 PP sessions with ECCO2R and “ultra-protective” ventilation during ARDS.

### F-79 Ultra-protective ventilation reduces biotrauma in patients on veno-venous extracorporeal membrane oxygenation for severe acute respiratory distress syndrome

#### Sacha Rozencwajg (*speaker*)^1^, Amélie Guihot^2^, Guillaume Franchineau^1^, Mickael Lescroat^1^, Nicolas Bréchot^1^, Guillaume Hekimian^1^, Guillaume Lebreton^1^, Pascal Leprince^1^, Brigitte Autran^2^, Charles-Edouard Luyt^1^,Alain Combes^1^, Matthieu Schmidt^1^

##### ^1^Sorbonne Universités, UPMC Univ Paris 06, INSERM UMRS_1166-iCAN, Institute of Cardiometabolism and Nutrition, Paris, FRANCE; ^2^Assistance Publique–Hôpitaux de Paris, Pitié–Salpêtrière Hospital, Immunology department, 75651 Paris Cedex 13, France, Paris, FRANCE

###### **Correspondence:** Sacha Rozencwajg - sacha.rozencwajg@gmail.com

*Annals of Intensive Care* 2019, **9(Suppl 1)**:F-79

**Introduction**: Ventilator settings in patients with severe Acute Respiratory Distress Syndrome (ARDS) supported by Veno-Venous Extra-Corporeal Membrane Oxygenation (VV-ECMO) are currently set arbitrarily. Our study aimed to evaluate the impact on serum and pulmonary markers of biotrauma of a) the transition to ultra-protective ventilation settings following the initiation of VV-ECMO, and b) different mechanical ventilation strategies while on VV-ECMO.

**Patients and methods**: Monocentric randomized clinical trial conducted during a 6-month period in patients receiving VV-ECMO for refractory severe ARDS. Once VV-ECMO started, patients were switched to the APRV mode with one second of 24 cmH2O high pressure (Phigh) and 2 s of 12 cmH2O low pressure (Plow) for 24 h. Patients were then randomized and assigned to each of the following three experimental steps according to a computer-generated allocation sequence- Phigh 24 cmH2O and Plow 20 cmH2O (“very high PEEP—very low driving pressure [VHPEEP-VLDP] step”) + Phigh 24 cmH2O and Plow 5 cmH2O (“low PEEP—high driving pressure [LPEEP-HDP] step”) + and Phigh 17 cmH2O and Plow 5 cmH2O (“low PEEP –low driving pressure [LPEEP-LDP] step”). Plasma and bronchoalveolar soluble receptor for advanced glycation end-products (sRAGE), and serum cytokines were dosed before VV-ECMO and after 12 h at each mechanical ventilation strategies.

**Results**: Sixteen patients on VV-ECMO after 7 (1–11) days on mechanical ventilation were included. “Ultra-protective” mechanical ventilation settings following ECMO initation was associated with a significant reduction in both plasma sRAGE and plasma cytokines. However, plasma sRAGE and cytokines were similar within each experimental steps during ECMO. Nevertheless, broncho-alveolar levels of sRAGE were at their lowest when driving pressure was minimal.

**Conclusion**: VV-ECMO allows an “ultra-protective ventilation”, which combines a significant reduction of tidal volume and driving pressure. This ventilation strategy significantly reduced pulmonary biotrauma, which could therefore decrease ventilator induced lung injury. However, the optimal “ultra-protective ventilation” strategy once VV-ECMO is initiated remains undetermined and warrants further investigations.


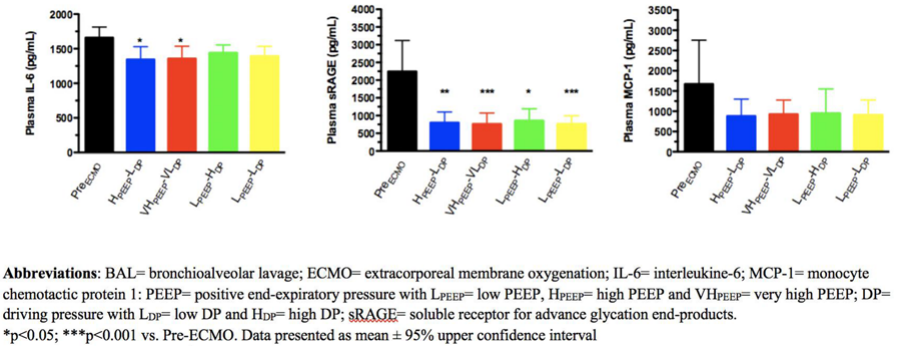


### F-80 Effect of Prone Position during Acute Respiratory Failure Apart From ARDS- A Prospective Study

#### Sabrine Nakaa (*speaker*), Nejla Tilouche, Oussama Jaoued, Habiba Ben Sik Ali, Rim Gharbi, Mohamed Fekih Hassen, Souheil Elatrous

##### Hopital taher sfar mahdia, Mahdia, TUNISIA

###### **Correspondence:** Sabrine Nakaa - nakaasabrine89@hotmail.com

*Annals of Intensive Care* 2019, **9(Suppl 1)**:F-80

**Introduction**: Prone position (PP) has been shown to be a useful adjunct in the treatment of patients with ARDS. But there is a lack of conclusive studies concerning the efficacy of PP in acute respiratory failure (ARF) without ARDS criteria. Objective- to evaluate the effect of prone positioning on oxygenation and survival among patients receiving invasive mechanical ventilation (MV) and having severe persistent hypoxemia due to another reason than ARDS.

**Patients and methods**: This prospective study was conducted in a 10- bed ICU between February 2015 and October 2017. Inclusion criteria were- patients requiring MV and who have a refractory hypoxemia. Exclusion criteria were- ARDS according to Berlin criteria and or ventilator associated pneumonia, contraindication to PP and a decision to withdraw or withhold therapy. Patients were placed in PP for at least 8 h. An improvement in oxygenation was defined as an increment of 20 mmHg or more in the PaO2 FiO2 after prone positioning. Outcome measures were improvement in oxygenation, ICU mortality, ventilator free days and ICU length of stay.

**Results**: During the study MV was used in 500 patients. We excluded 470 patients (70 patients didn’t met ARDS criteria and 400 patients were not hypoxemic). Thirty patients with a mean age of 50 ± 17 years were included. ARF was the most frequent reason of admission (36.7%). Twenty five patients (83.4%) were responders to PP. Indeed, PaO2 FiO2 increased significantly after PP (from 107 ± 41 to 185 ± 66 mmHg, p < 10-3) and the PaCO2 has decreased significantly (from 56 ± 17 to 48 ± 12, p < 10^−3^) compared to the supine position. Twelve patients (40%) presented one or more complications related to the PP. The most common complication was decubitus ulcers in 27% of cases. The comparaison between responders and non responders to PP shows no differences in demographic characteristics, co morbidities and PaO2 FIO2. The ICU mortality rate in PP responders was 32% compared to 20% in non responders (p = 1.00). The post hoc analysis in the sub groups of patients according to the degree of hypoxemia before PP (PaO2 FIO2 before PP <=100 and PaO2 FiO2 before PP > 100) has shown that the ICU mortality, ICU LOS and Ventilator-free days were similar in both groups.

**Conclusion**: In the present study prone positioning applied to severely hypoxemic patients without ARDS criteria was associated with an improvement in oxygenation and in carbon dioxide washing.

### F-81 Incidence of and risk factors for thrombotic complications following venovenous extracorporeal membrane oxygenation- a CT-scan study

#### Gabriel Parzy (*speaker*)

##### APHM, Marseille, FRANCE

###### **Correspondence:** Gabriel Parzy - gabriel.parzy@gmail.com

*Annals of Intensive Care* 2019, **9(Suppl 1)**:F-81

**Introduction**: The aims of this study were to 1) analyze Cannula-associated deep vein thrombosis (CaDVT) incidence after veno-venous extracoporeal membrane oxygenation (VV-ECMO) using a CT-scan and 2) identify the associated risk factors of CaDVT.

**Patients and methods**: This retrospective observational analysis was conducted in a tertiary referral university teaching hospital. Patients under VV-ECMO with a femoro-femoral or femoro-jugular cannulation admitted for ARDS or primary dysfunction graft after pulmonary transplantation were included. Diagnosis of CaDVT was performed with a Iodinized CT-scan within 4 days after decannulation.

**Results**: 228 patients were screened, 105 were included. Bacterial pneumonia was the main indication of VV-ECMO (46.7%). A CaDVT was found in 75 patients (71.4%) despite a mean APTT at 1.60 ± 0.31. Specifically for femoral CaDVT, femoro-femoral cannulation induced more CaDVT than femoro-jugular cannulation (69.2% vs 63.1% respectively, p = 0.04). The mean number of patients requiring ECMO circuit replacement was significantly higher in CaDVT group (38.7% vs 16.7%, p = 0.04). Multivariate logistic regression analysis showed that thrombocytopenia < 100 G L was significant for a decreased risk to develop a CaDVT (HR 0.98 + CI95% [0.98–1.00], p = 0.02).

**Conclusion**: Cannula associated deep vein thrombosis after VV-ECMO is a frequent event (71.4%). This suggest that a systematic vascular axes imaging is necessary after VV-ECMO. Thrombocytopenia is associated with reduced thrombotic events.

### F-82 Impact of the length of stay in emergency department on the mortality in intensive care unit of septic shock patients- a cohort study

#### Noémie Teixera (*speaker*)^1^, Geoffroy Rousseau^2^, Emeline Laurent^3^, Saïd Laribi^4^

##### ^1^CHRU Tours, Cinq Mars La Pile, FRANCE; ^2^CHRU Tours - Hopital Trousseau, Tours, FRANCE; ^3^CHRU Tours-Hopital Bretonneau, Tours, FRANCE; ^4^CHRU Tours, Tours, FRANCE

###### **Correspondence:** Noémie Teixera - teixera.noemie@gmail.com

*Annals of Intensive Care* 2019, **9(Suppl 1)**:F-82

**Introduction**: The overcrowding of emergency departments (EDs) increases. That affects the management of our patients, especially critically ill patients. Septic shock is a life-threatening disease with a mortality of 40%. The management of these patients and their transfer to intensive care units (ICUs) must be a priority. Did the length-of-stay (LoS) of septic shock patients in ED increase their mortality in ICU?

**Patients and methods**: We performed a monocentric retrospective cohort study. Patients hospitalized in ICU for septic shock between 2012 and 2017 and admitted via our ED were included. We excluded “Do not resuscitate” patients and patients admitted in ICU from another ED. We compared the LoS in ED between “alive group” and “dead group” in ICU, as IGS II score, age, delay of antibiotics, need of mechanical ventilation, use of vasopressive drugs, fluid management and qSOFA. Then, we realized a multivariate analysis.

**Results**: 115 patients were included in ICU for septic shock. The median LoS in ED was 317 [245–496] minutes. The mortality rate in ICU was 25.2%. In “alive group”, the LoS in ED was 343 [244–565] minutes vs 295 [240–367] minutes in “dead group”, there was no significant difference. In the univariate analysis, qSOFA, lactates, IGS II score and the use of mechanical ventilation in ED were significant. In multivariate analysis, IGS II score and the use of mechanical ventilation in ED were significantly associated with the mortality in ICU.

**Discussion**: The LoS in ED was not associated with the mortality in ICU in our study. “Dead group” was more often associated with high comorbidities and altered qSOFA at admission in ED. The use of mechanical ventilation in ED was associated with poor prognosis, whatever the origin of septic shock. It is important to confirm these results with a large prospective study.

**Conclusion**: Despite the overcrowding of EDs, emergency physicians and paramedics seemed to remain vigilant to detect, manage and transfer quickly septic shock patients.

### F-83 Fluid balance impact in sepsis and septic shock

#### Khaoula Ben Ismail (*speaker*), Ines Fathallah, Ghada Sboui, Sahar Hbecha, Ameni Sghier, Asma Mehdi, Haifa Fazeni, Nadia Kouraichi

##### Hôpital Ben Arous Yassminet, Hammam Plage | Ben Arous, TUNISIA

###### **Correspondence:** Khaoula Ben Ismail - khaoula87@hotmail.fr

*Annals of Intensive Care* 2019, **9(Suppl 1)**:F-83

**Introduction**: Aggressive fluid resuscitation is the initial approach for cardiovascular instability. Consequently, large volumes of fluid are given to septic patients during their management. Our study aimed to investigate the impact of cumulative fluid balance on critically ill patients with sepsis or septic shock admitted in intensive care unit (ICU).

**Patients and methods**: Retrospective monocentric study conducted in a medical ICU from September 2017 to September 2018. Patients with septic shock who required dialysis prior to hospitalization were not included.We included patients who presented sepsis or septic shock during their hospitalization and we studied the relation between fluid balance and prognosis.

**Results**: We enrolled 57 patients with an average age of 58 ± 16 years. The main reasons of hospitalization were respiratory failure and neurological disturbances (70% of cases). Median APACHE II and SOFA score at admission were respectively 15 [10 + 21] and 5 [3 + 8]. Sepsis and septic shock were related to pulmonary infections in 65% of cases, followed by urinary tract infections (14%) and catheter related infections (7%). The most frequent infectious agents were Gram negative bacilli (30%). Mechanical ventilation was required in 39 patients. Vasoactive drugs were used in 34 patients. Median duration of mechanical ventilation and hospital stay were respectively 12 [6 + 37] and 14 [5 + 24] days. The overall mortality was 40%. Multivariate analysis reveled that mortality was associated with positive cumulated fluid balance before and on the sepsis onset with relative risks respectively at 1.55(IC 95% [1.11 + 2.18], p = 0.013) and 3.32 (IC 95% [1.74 + 6.32], p < 0.001).

**Conclusion**: Positive fluid balance is associated to poor prognosis in septic patients.

### F-84 Incidence and risk factors of venous thromboembolism in patients with septic shock

#### Sabrine Bradai (*speaker*), Manel Zekri, Abir Bouattour, Amal Triki, OlfaTurki, Mabrouk Bahloul, Mounir Bouaziz

##### Department of Intensive Care, Habib Bourguiba University Hospital, Sfax, TUNISIA

###### **Correspondence:** Sabrine Bradai - Sabrine.bradai2@gmail.com

*Annals of Intensive Care* 2019, **9(Suppl 1)**:F-84

**Introduction**: Venous thromboembolism (VTE) is a common and preventable complication among hospitalized patients in intensive care unit (ICU). However, little is known about it incidence and its particularities in patients with septic shock.

**Patients and methods**: It is a prospective analytical study, conducted at the ICU of Habib Bourguiba university hospital, Sfax, Tunisia, between January 01, 2017, and December 31, 2017. All Patients who developed septic shock due to bacterial infection during the study period were enrolled. Thrombo-prophylaxis was recorded for all patients. The diagnosis of TVE is confirmed by spiral computed tomography scan and compression venous ultrasound.

**Results**: Sixty patients with septic shock were included in the study. Among them, 24 developed VTE during their hospitalisation. The incidence of VTE was 40%. VTE was associated with increased length of stay, longer mechanical ventilation and more use of tracheotomy. The mortality rate was not higher in patients with acute VTE. (Table I).

**Conclusion**: Patients who develop septic shock are considered at higher risk for developing VTE. It is the result of multiple factors including immobility, activation of thrombo-inflammatory pathways, disseminated intravascular coagulation, venous stasis and central venous catheter insertion. Thus, more effective VTE prevention strategies are necessary in patients with sepsis.

**Table:** Table I: Comparison between the two groups (with and without VTE)

CHARACTERISTIC/BIOLOGICAL PARAMETERS ON SHOCK DAY	VTE GROUP (N = 24)	VTE-FREE GROUP (N = 36)	P VALUE
Age (years)	51.8 ± 16.4	45.1 ± 20.3	0.183
Sex ratio (M/F)	21/3	25/11	0.105
SAPS II	35.3 ± 14.5	44.3 ± 16.2	**0.032**
SOFA	5.5 ± 1.3	8 ± 4.1	**0.017**
GCS	10.6 ± 4	9.5 ± 4.3	0.329
Type of admission:			0.335
- Traumatism	10	22	
- Medical	11	11	
- Surgical	3	3	
Duration of mechanical ventilation (days)	32.7 ± 11.4	12.9 ± 9.3	**<0.001**
Tracheotomy	23	23	**0.004**
DIC	6	17	0.083
Acute kidney failure	18	23	0.365
Dialysis	2	13	**0.015**
Length of stay (days)	37.3 ± 11.6	16.3 ± 11.7	**<.001**
Mortality rate	58.3%	63.9%	0.665
pH	7.39 ± 0.09	7.37 ± 0.10	0.740
PACO_2_ (mmHg)	38.13 ± 8.38	37.45 ± 9.66	0.779
PAO_2_/FiO_2_ ratio	270 ± 112.8	267.4 ± 109.8	0.931
HCO_3_^−^ (mmol/l)	22.47 ± 4.41	21.88 ± 6.10	0.751
Troponin (ngl/l)	0.079 ± 0.086	0.57 ± 1.03	0.869
SGOT (UI/l)	71.4 ± 59.3	91.5 ± 142.6	0.952
SGPT (UI/l)	57.6 ± 65.8	54.3 ± 55.5	0.922
Bilirubin (μmol/l)	45.9 ± 51.1	56.4 ± 118.6	0.717
Blood urea (mmol/l)	12.4 ± 7.5	17 ± 16	0.763
Blood creatinine (μmol/l)	109 ± 72	157 ± 126	0.381
CRP (mg/L)	231.9 ± 103	230 ± 138	0.594
Procalcitonin (ng/mL)	14.6 ± 20.9	11.3 ± 15.5	0.682
SChEA (UI/L)	3391 ± 1430	3423 ± 1151	0.922

VTE: Venous thromboembolism; GCS Glasgow coma scale score; SAPSII: Simplified acute physiology score; DIC: Disseminated intra-vascular coagulation; SOFA score: Sepsis-related Organ Failure Assessment score; SGOT: Sérum Glutamooxaloacétate Transférase; SGPT: Sérum Glutamopyruvate Transférase; SChEA: Serum Cholinesterase Activity

### F-85 Muscle Lactate and lactate to pyruvate ratio clearance as useful biomarkers for the prediction of mortality in septic shock patients

#### Zied Hajjej (*speaker*), Mayssa Daiki, Walid Sellami, Hedi Gharsallah, Iheb Labbene, Mustapha Fejani

##### Military Hospital, Tunis, TUNISIA

###### **Correspondence:** Zied Hajjej - hajjej_zied@hotmail.com

*Annals of Intensive Care* 2019, **9(Suppl 1)**:F-85

**Introduction**: Microcirculatory alterations are frequently observed in patients with sepsis. In vivo microdialysis (MD) is a bedside technique that can monitor tissue metabolic changes. We conducted this study aiming to assess the performance of muscle Lactate and lactate to pyruvate (L P) ratio clearance in predicting mortality in septic shock patients by using microdialysis.

**Patients and methods**: The study was designed as a prospective, controlled, clinical trial and performed in a multidisciplinary intensive care unit. 56 septic shock patients were enrolled. Interstitial tissue concentrations of lactate, pyruvate, glucose and glycerol were obtained at baseline and every 6 h for 3 days by using muscle microdialysis. Clearances of muscle lactate, and L P ratio were defined as the percentage change in muscle lactate level or L P ratio compared to baseline (H0) values. A positive value of clearance means a decrease in the rate of the parameter under study.

**Results**: We found an association between muscle lactate clearance and hospital mortality with a statistically significant difference at H54 (p = 0.037), H60 (p = 0.033) and H72 in the study (p = 0.012). We also found an association between clearance of muscle lactate to pyruvate ratio and hospital mortality with a statistically significant difference at H54 (p = 0.015), H60 (p = 0.001), and H72 of the study (p = 0, 04).

**Conclusion**: Among patients with septic shock, improvement of muscle Lactate and lactate to pyruvate ratio clearance from the 54th hour may indicate a resolution of global tissue hypoxia and is associated with decreased mortality rate.


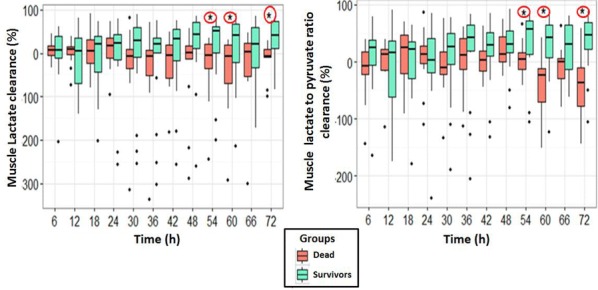


### F-86 Performance of the Sepsis- definitions in a Tunisian intensive care unit

#### Zied Hajjej (*speaker*), Ben Mahmoud Khalthoum, Walid Samoud, Olfa Yengui, Mayssa Daiki, Mustapha Ferjani

##### Military Hospital, Tunis, TUNISIA

###### **Correspondence:** Zied Hajjej - hajjej_zied@hotmail.com

*Annals of Intensive Care* 2019, **9(Suppl 1)**:F-86

**Introduction**: Since the first publication, in 2016, Sepsis-3 definitions are not universally accepted and are becoming a matter of controversy. Because clinical and laboratory parameters used for the development of these definitions were derived mainly from patients hospitalized in United States Intensive Care Units (ICU). The aim of this study was to evaluate the performance of the Sepsis 3 definitions for prediction of ICU-mortality in a Tunisian ICU population as compared to 1992 Consensus Definitions (Sepsis-2 definitions).

**Patients and methods**: It was a retrospective descriptive study performed in an 18-bed medical surgical intensive care unit at Tunis military hospital (Tunisia). From January 2012 to January 2016, all patients admitted to the ICU were eligible for this study. Inclusion criteria were as follows- age > 18 years, and an admission diagnosis of sepsis, severe sepsis or septic shock as defined according to the Surviving Sepsis Campaign guidelines (Sepsis-2 consensus). The new Sepsis-3 definition was secondary used. The primary outcome of interest was ICU mortality defined as death before ICU discharge.

**Results**: Of 3246 participants enrolled between 2012 and 2016, we included 1080 individuals with follow-up information available. When the Sepsis-2 definitions were used there was a difference in mortality only between septic shock and sepsis patients. While Sepsis-3 definitions show that mortality increased from 16% in no-dysfunction-infected patients to 30% in patients with qSOFA ≥ 2 and 44% or 46% for sepsis or septic shock patients, respectively.

**Conclusion**: sepsis-3 was better than sepsis-2 definitions at stratifying mortality among septic patients admitted to an ICU of an emerging country (Tunisia).


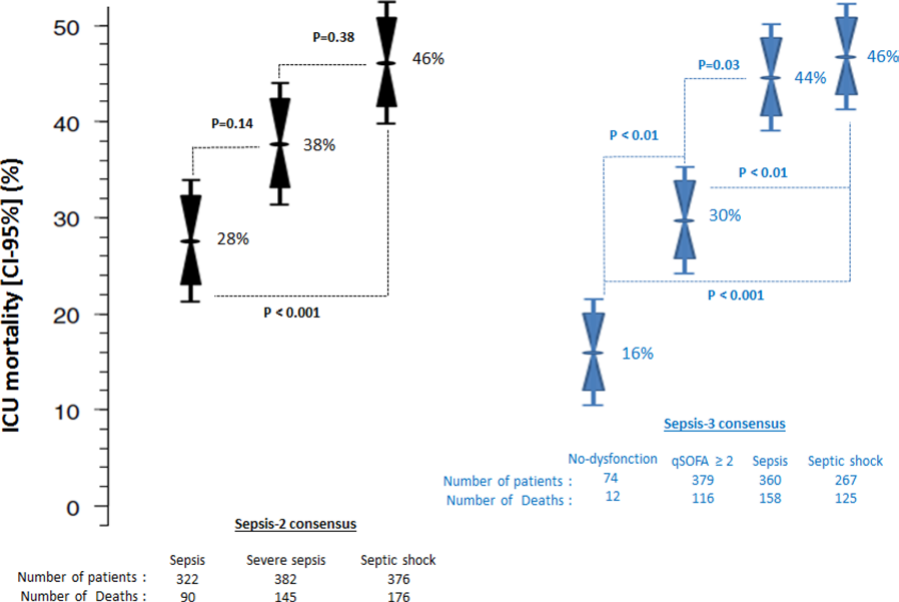


### F-87 Prognostic factors and impact of hyperbaric oxygen therapy for the management of necrotizing soft tissue infections in critically ill patients

#### Cécile Bouges (*speaker*), Olivier Marion, Edith Hourcastagnou, Jean Ruiz, Hélène Vinour, Véronique Ramonda, Guillaume Ducos, Thierry Seguin, Béatrice Riu-Poulenc, Stein Silva

##### Critical care unit - Purpan University Teaching Hospital, Toulouse, FRANCE

###### **Correspondence:** Cécile Bouges - bouges.c@chu-toulouse.fr

*Annals of Intensive Care* 2019, **9(Suppl 1)**:F-87

**Introduction**: Necrotizing soft tissue infections (NSTI) is a medical-surgical emergency related to severe bacterial soft tissue infections. Hyperbaric oxygen therapy (HBO) has been proposed as adjuvant therapy in this indication. The objective of this study was to assess the prognostic factors and the impact of HBO in critically ill patients with NSTI.

**Patients and methods**: Between January 2005 and December 2017, 172 patients with NSTI admitted in intensive care units in a tertiary care teaching hospital were included in this retrospective study. Among them, one hundred and twenty-eight patients (74.4%) received HBO therapy. Prognostic factors associated with 90-day mortality in univariate analysis were included in a logistic regression model. Complications associated with the use of HBO were also reported.

**Results**: Eighty-four patients (48.8%) had NSTI of the limbs, 74 patients (43.3%) had perineal NSTI and 14 patients (8.1%) had infections of the cervical-thoracic region. The median SAPSII score was 51.5 [36–70] and 152 patients (87.2%) had septic shock. The overall mortality at day 90 was 30.8% (n = 53). In multivariate analysis, factors significantly associated with 90-day all-cause mortality were admission-based severity score (SAPSII, OR = 1.06 [1.03–1.09], p < 0.001), the delay between diagnosis and surgery (OR = 1.48 [1.05–2.09], p = 0.021), hyperlactatemia at admission (OR = 1.40 [1.13–1.74], p = 0.002) and chronic renal disease (OR = 7.8 [1.16–52.40], p = 0.031). Protective factors were a streptococcal infection (OR = 0.11 [0.02–0.51], p = 0.004) and an effective probabilistic antibiotic therapy (OR = 0.24 [0.07–0.91], p = 0.032). HBO was not associated with decreased mortality in multivariate analysis. Twenty-three patients suffered from severe complications of HBO. HBO complications were favored by the use of norepinephrine (OR = 9.63 [1.65–56.36], p = 0.010), mechanical ventilation (OR = 4.89 [1.59–15.08], p = 0.005) or the combination of the two (OR = 10.38 [3.76–28.65], p < 0.001) during the HBO sessions.

**Conclusion**: The initial severity, a chronic renal disease, hyperlactatemia and the delay between diagnosis and surgery were independent risk factors for 90-day mortality of critically ill patients with NSTI. HBO therapy was not associated with patient survival but may be responsible for serious complications in this indication.

### F-88 A new prognostic indicator in septic shock: muscle mass measured by the thickness of the quadriceps in ultrasound

#### Marc Selim (*speaker*), Caroline Lemaitre, Fabienne Tamion

##### CHU, Rouen, FRANCE

###### **Correspondence:** Marc Selim - marcselim@gmail.com

*Annals of Intensive Care* 2019, **9(Suppl 1)**:F-88

**Introduction**: Mortality from septic shock remains high. Undernutrition during septic shock is responsible for significant morbidity and mortality.The objective of our study is to evaluate the interest of muscle mass loss by ultrasound measurement of quadriceps thickness in patients admitted to intensive care for septic shock, as a prognostic factor for mortality.

**Patients and methods**: This was a prospective observational study conducted in the medical intensive care unit of the Centre Hospitalier Universitaire of ROUEN.The inclusion period extended from April 2017 to October 2017.The main objective was to evaluate the relationship between the variability of muscle thickness measured by ultrasound at d1-d4 and mortality at d28.The secondary objective was to assess the difference in variability in mean quadriceps thickness between living and deceased patients at D28.

**Results**: 34 patients admitted to septic shock were included.The median of the quadriceps thickness variability between J1 and J4 was -6.77% and was defined as a threshold value.We observed higher mortality in the group whose quadriceps thickness decreased by more than 6.77% (p = 0.02 + CI [0.0015 + 0.7157]). Sensitivity was 88%, IC95%[50 + 99] and specificity was 64%, IC95%[42 + 82]. At D28 the average thickness loss was greater for deceased patients (13%[5.5 + 20.7]) than for survivors (1.8%[- 9 + 5.26], p = 0.02).

**Conclusion**: An early loss of more than 6.77% quadriceps thickness could be associated with increased mortality on D28 during septic shock.

### F-89 Outcome of patients after a decision of pursue, withdrawing or withholding life sustaining treatment in a french Pediatric Intensive Care Unit

#### Cécile Thivent (*speaker*), Stéphane Dauger, Julie Sommet, Fleur Le Bourgeois, Armelle Nicolas-Robin, Géraldine Poncelet

##### CHU Robert Debré, Paris, FRANCE

###### **Correspondence:** Cécile Thivent - cecile.thivent@gmail.com

*Annals of Intensive Care* 2019, **9(Suppl 1)**:F-89

**Introduction**: The ethical and reasonable aspect of the treatments in Pediatric Intensive Care Unit (PICU) is regularly evaluated taking into account the prognosis of the patients. These particular situations lead to multidisciplinary discussions about continuation, withholding or withdrawing life sustaining treatment (LST) for the patient. The outcome of these patients following a LST decision is variable and poorly described in the pediatric literature. The main purpose of this study is to describe this population in terms of mortality and morbidity 3 months after a LST discussion.

**Patients and methods**: It is an observational descriptive retrospective and monocentric study in a french PICU, including patients under 18 years of age for whom at least one meeting about LST has taken place in the intensive care unit, between 2015 and 2017, and whose discussion has been recorded on a standardized form provided for this purpose.

**Results**: 47 patients were included, with a median age of 7.8 months. The majority had a chronic condition (76.6%) and the main reason for hospitalization in PICU was a neurological failure (29.8%). The mortality rate at 3 months was 66% - 100% in case of withdrawing LST vs 60.9% in case of withholding LST vs 36.4% in case of continuation of LST. 83.9% of the deaths took place in PICU, the median delay of which was 3 days [1–8.5] after the discussion of LST. Patients for whom a decision to withdraw LST has been taken were younger, had a higher predictive mortality score, had more frequent invasive ventilation and curare treatment. Among the group of withdrawing LST, 92.3% of patients were extubated under deep and continued sedation. About one third of patients survived 3 months after a discussion of LST, of which 25% despite a decision of withdrawing or withholding LST, and the major part lived at home (68.8%) and benefited from rehabilitative care (75%). Among survivors, median Pediatric Overall Performance Category (POPC) and Pediatric Cerebral Performance Category (PCPC) scores were 4 out of a maximum of 6.

**Conclusion**: Pediatric patients for whom an ethical discussion about LST in PICUs have a high mortality rate, regardless the decision taken. Survivors have severe functional and neurological disabilities and need regular follow up. The collaboration between PICU team and palliative medicine team could improve the quality of the care and the accompanying process of those patients.


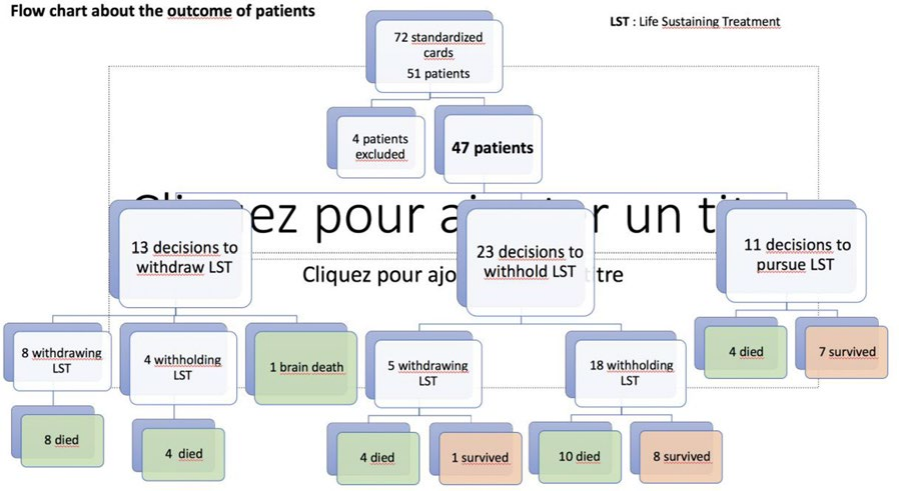


### F-90 Perceptible end-of-life signs in Paediatric Intensive Care Unit, parent’s experiment and difficulties to lead studies

#### Fleur Le Bourgeois (*speaker*)^1^, Violaine Mattioni^1^, Céline Ricignuolo^2^, Vincent Amelot^3^, GeraldinePoncelet^1^, Julie Sommet^1^, Michaël Levy^1^, Jérôme Naudin^1^, Stephane Dauger^1^, Charlotte Pierron^4^

##### ^1^Hôpital Robert Debré, Service de Réanimation et Surveillance Continue Pédiatriques, Paris, FRANCE; ^2^Hôpital Necker Enfants Malade, service de réanimation et surveillance continue pédiatriques, Paris, FRANCE; ^3^Laboratoire Psychopathologie et Processus de Santé (LPPS) Université Paris Descartes, Paris, FRANCE; ^4^Service de Néonatologie et Soins Intensifs Pédiatriques, Centre Hospitalier du Luxembourg, LUXEMBOURG

###### **Correspondence:** Fleur Le Bourgeois - fleur.lebourgeois@aphp.fr

*Annals of Intensive Care* 2019, **9(Suppl 1)**:F-90

**Introduction**: The agony in paediatric intensive care unit (PICU) has been poorly described in the literature. Although never proven, better information of parents could reduce the trauma of death. The main objective of this study is to define which perceptible end-of-life symptoms in PICU are the most traumatic for parents and to determine their wishes as for the information they would like to receive about it.

**Patients and methods**: Prospective ongoing study, conducted in a 20-bed PICU of a French University Hospital with the authorization of the Robert Debré Hospital Ethic Committee. All patients under 18 years, and likely to die in the PICU, are included. Before death, both parents are informed about the study by a physician. Parents are systematically recalled four months after death to participate to a semi-structured interview conducted by a psychologist. The questionnaire concerned the shocking perceptible symptoms during the agony and the information they would have liked to receive about it.

**Results**: After 17 months’ enrolment, 18 patients have been included among 62 deaths in PICU. No parent approached refused the study. Finally, 20 parents could be interviewed, of whom 15 were recalled, and four parents accepted the interview. One mother described changes in skin colour and bleeding, and was shocked. Both parents of an infant described weird movements but said it helped them to recognize death, and one mother did not notice any sign, but was grateful for having been informed about possible symptoms. We did not call three fathers because their wives refused the interview, and a couple because it was bad timing.

**Discussion**: The difficulties encountered are not those expected, such as parents’ refusal at inclusion. The lack of parental involvement in the interviews is the main problem. It is partially due to a strong apprehension to recall them, with a feeling of guilt that overtakes the benefit expected from the study. For the parents who participated, there was always a motivation- a counter-gift for caregivers, a suffering and the desire to avoid that to other bereaved parents.

**Conclusion**: This ongoing study is the first prospective study about the experience of parents about their child end-of-life perceptible signs in PICU. Most of our difficulties to include parents are coming from the caregivers. Caregivers need to remove their taboo about end-of-life research and find out which motivation might help parents to testify.

### F-91 Perceptible end-of-life signs in PICU, a prospective study

#### Charlotte Pierron (*speaker*)^1^, Géraldine Poncelet^2^, Julie Sommet^2^, Jérome Naudin^2^, Fleur Le Bourgeois^2^

##### ^1^Centre Hospitalier du Luxembourg, Strassen, LUXEMBOURG; ^2^Hôpital Robert Debré, Unité de réanimation et soins continus pédiatriques, Paris, FRANCE

###### **Correspondence:** Charlotte Pierron - pierron.charlotte@chl.lu

*Annals of Intensive Care* 2019, **9(Suppl 1)**:F-91

**Introduction**: IFiReaPed study is a prospective ongoing study about perceptible end-of-life signs in Paediatric Intensive Care Unit (PICU). IFiReaPed main hypothesis is that a better information of parents about those perceptible end-of-life signs could help them to live better this situation. In the literature, there is no study on this topic in PICU, therefore our first step is to observe dying children to determine which signs are worsening or appearing during end-of-life period.

**Patients and methods**: The study is ongoing in a French PICU. All patients, likely to die at short term in PICU are included, after informing the parents of the study. After obtaining their non-opposition, caregivers observe the dying child until his death. The child is excluded if the death doesn’t occur in the five next days. A non-exhaustive list of perceptible signs is proposed to the caregivers, and they have to fulfill the presence, absence, or worsening of the signs during the dying process.

**Results**: Between April 2017 and August 2018, 18 patients have been included (44 have been excluded because of unpredictable death, non-french speakers parents, oversight or brain death). The median age is 0.9 year, 78% had chronic disease, the main causes of death are respiratory or neurological failure and the death followed limitation or withdrawal of the treatments in 17 patients. The most frequent perceptible signs appearing or worsening during the dying process concern the child’s appearance (cyanosis, pallor, marbling, petechiae, edema…), sometimes respiratory signs are perceptible, but 7 patients were still under invasive mechanical ventilation at the time of death (details in the table). Unsurprisingly, some physiological parameters are presents during the dying process (bradycardia, hypotension, desaturation), and they could be noticed by the parents only because of the monitor. Gasps have been noted in 8 patients. Few patients presented digestive or neurological signs, but all the patient except one received an sedoanalgesia.

**Conclusion**: IFiReaPed is the first prospective study trying to determine what are the perceptible signs during the end-of -life in PICU. The most frequent signs are gasp or concern changes in skin color or in appearance. This signs could hurt the parents and could be easier to support if they received information before their onset. The next steps of the study are to determine the signs noticed by the parents, their need of explanation and the impact of these informations on their experience.

**Table:** 

Type of signs	Sign	Appearing	Worsening
Cutaneous signs/Appearance signs	Pallor	2	7
	Cyanosis	3	7
	Marbling	4	5
	Bedsore	1	1
	Jaundice	1	1
	Petechiae		
	Edemas	2	6
Other signs	Cold body	5	3
	Sweat	1	0
	Change in smell	1	0
	Polyuria	1	0
Respiratory signs	Gasps	8	0
	Breathing Congestion	3	0
	Apnea	5	0
	Bradypnea	4	0
	Increased respiratory work	0	1
	Stridor	0	0
Neurological signs	Anxiety or delirium or abnormal movements	0	0
	Consciousness disorder not linked to sedoanalgesia	1	2
Digestive signs	Diarrhea	3	1
	Hiccups or thirstiness	0	0
	Vomiting	2	0
	Hypersalivation	3	0
Others	Bleeding	1	2
Physiological parameters	Bradycardia	10	0
	Desaturation	4	8
	Hypotension	3	5

### F-92 Predictive factors of life support withholding at admission in PICU for children suffering from multiple disabilities

#### David Brossier (*speaker*)^1^, Christophe Duval^2^

##### ^1^Service de réanimation pédiatrique, Caen, FRANCE; ^2^GHH Hopital Monod, Montivilliers, FRANCE

###### **Correspondence:** David Brossier - brossier-d@chu-caen.fr

*Annals of Intensive Care* 2019, **9(Suppl 1)**:F-92

**Introduction**: Children suffering from multiple disabilities are frequently hospitalised in paediatric intensive care unit (PICU). As the illness evolves the burden of the treatments increases, sometimes at the expense of children’s comfort. Thus, palliative cares should be considered for, they could improve their quality of life and minimize pain. This study aimed to identify the predictive factors of life support withholding at admission in PICU.

**Patients and methods**: A retrospective study was conducted in the PICU of the CHU of Caen. All children under 18 years old and suffering from multiple disabilities, who were hospitalized between January 2006 and December 2016, were included in the study and allocated into two groups (withholding group vs control group), depending on the notification on their medical file of a life support withholding.

**Results**: 119 patients were included. The life support withhold rate at admission was 9.6% (12). Withholding group was younger than control group (median age 59[29–182] vs. 102[29–160], p < 0.01) and most hospitalised for respiratory issues (n = 6(50) vs 20(25), p < 0.05). There was no difference for gender (girl = 50(47%) vs. 7(58%), p = 0.5), hospitalization for surgical issues and nature of disabilities. The predictive factors of a life support withholding were a previous hospitalization in PICU (11(92%), p < 0.01, OR 14.9[2–662.5]), the requirement of respiratory (5(42%), p = 0.01, OR 5.4[1.2–23]) or feeding support (10(83%), p = 0.01, OR 9.7[1.9–94.3]), and a higher functional status score (POPC- 4[4–4], p = 0.01).

**Conclusion**: The withholding of life support for children suffering from multiple disabilities appeared limited in our paediatric department. The main predictive factor identified in our study was at least one admission in PICU which raised the question of the role of paediatrician in the withholding of life support.

### F-93 **Effect of a nursing-implemented sedation-analgesia protocol on duration of mechanical ventilation in pediatric intensive care unit**

#### Marie Emilie Lampin (*speaker*), Claire Cavrois, Jeremie Rousseaux, Claire Lereun, Stéphane Leteurtre

##### Univ. Lille, CHU, Lille, FRANCE

###### **Correspondence:** Marie Emilie Lampin - marie-emilie.lampin@chru-lille.fr

*Annals of Intensive Care* 2019, **9(Suppl 1)**:F-93

**Introduction**: Sedation-analgesia is part of the management of ventilated pediatric patients and must be adjusted to each patient. The objective was to evaluate the implementation of nursing-driven protocol sedation-analgesia on duration of mechanical ventilation.

**Patients and methods**: This was a before-and-after prospective, observational study in a pediatric intensive care unit that included children aged 28 days to 17 years, receiving mechanical ventilation longer than 24 h. During the observational period, sedation-analgesia was administred according to medical prescription. During the intervention period, sedation-analgesia was adjusted by nurses according to protocol-based sedation-analgesia guideline, including an evaluation by Comfort-B scale.

**Results**: A total of 98 children were included (50 before protocol and 48 after). The decrease in the duration of mechanical ventilation after protocol was not statistically significant (median duration was 3 days [2.2–4.9] vs. 3.8 days [2.2–7.2] before protocol, p = 0.25). After protocol, the mean of Comfort-B was 13.5 (for an optimal objective fixed by the protocol between 13 and 17). The monitoring of Comfort-B was significantly more frequent. There was no decrease in total doses of hypnotics or opioids. The compliance to protocol by nurses was insufficient (median rate- 46.3% [30.5–57.8]). There was not appearing to be an increase in complications related to insufficient sedation-analgesia (auto-extubation, low number of medical alert procedures).

**Conclusion**: The implementation of nursing-driven protocol sedation-analgesia in children did not reduce the duration of mechanical ventilation and the total doses of sedation-analgesia, but the protocol was insufficiently applied. However, it ensured the comfort of ventilated children and was safe.

### F-94 Withdrawal syndrome in a pediatric intensive care unit: comparison of practices and risk factors in 2016 and 2018

#### Mélanie Rapp (*speaker*)^1^, Marine Bernard^2^, Marion Grimaud^2^, Laure De Saint Blanquat^2^, Sylvain Renolleau^2^

##### ^1^Hopital Trousseau, Paris, FRANCE; ^2^Hopital Necker, Paris, FRANCE

###### **Correspondence:** Mélanie Rapp - melanie.rapp.23@gmail.com

*Annals of Intensive Care* 2019, **9(Suppl 1)**:F-94

**Introduction**: Withdrawal syndrome (WS) is frequent in Pediatric Intensive Care Unit (PICU). The incidence could reach 50 to 80% of patients. Prolonged sedative drugs give rise to tolerance and withdrawal phenomena. The WAT-1 scale is a specific tool to diagnose WS for children from birth to 18 years old. The main objective of our work was to compare our practices regarding screening and management of WS in our PICU.

**Patients and methods**: The first 50 consecutive patients of each period were included. They were to be ventilated and under sedation with opioids and benzodiazepines. All the WAT-1 scores between 24 h before and three day after extubation were recorded. For each patient, cumulative doses of opioids and benzodiazepines were calculated and other sedative co-treatments reported. The use of clonidine or dexmedetomidine was recorded.

**Results**: A total of 95 patients were analyzed. The proportion of WS was 43% (n = 21) in 2016 and 30% (n = 14) in 2018 (p-value = 0.29). In the group without WS, 29 patients didn’t have any evaluation with the WAT-1 scale during the five days around extubation- 27% (n = 13) in 2016 and 35% (n = 16) in 2018 (p-value = 0.50). Length of sedation was longer in the WS group- 10 versus 5.5 days (p-value = 0.0001), as the length of mechanical ventilation- 10 versus 5 days (p-value = 0.0001). Higher global cumulative doses of sedative treatments were used in the WS group- 28.5 mg kg versus 7.2 mg kg for morphine (p-value = 2 × 10^−6^) and 24.4 mg kg versus 6.1 mg kg for midazolam (p-value = 2 × 10^−5^). In 2018 treatment’s decrease tented to be slower in the WS group- 23.5 versus 8.0 h (p-value = 0.07). The use of clonidine was more important in the WS group during both periods- 62% of the WS group versus 21% in 2016 (p-value = 0.007) and 78% of the WS group versus 28% in 2018 (p-value = 0.003). The use of dexmedetomidine concerned only two patients and could not be studied.

**Conclusion**: Our study is in accordance with the literature concerning the risk factors of WS. Our practices didn’t really change between 2016 and 2018 as the proportion of WAT-1 scale remains the same. The use of WAT-1 should be more systematic. One way of improvement will be the realization of a common protocol in our unit.

### F-95 Use of a french version of the Cornell Assessment Pediatric Delirium (CAPD) in Pediatric Intensive care Unit - a pilot study

#### Jehanne Malek (*speaker*)^1^, Marine Bernard^1^, Sylvain Renolleau^1^, Francoise Yung^2^, Mehdi Oualha^1^, Laure De Saint Blanquat^1^

##### ^1^CHU Necker Enfants Malades, Paris, FRANCE; ^2^CHU Sainte Justine, Montreal, CANADA

###### **Correspondence:** Jehanne Malek - jehanne.malek@gmail.com

*Annals of Intensive Care* 2019, **9(Suppl 1)**:F-95

**Introduction**: In Pediatric Intensive Care Unit (PICU), delirium has a prevalence of 21 to 25% (1). it’s probably underdiagnosed. The Cornell Assessment Pediatric Delirium (CAPD) is a validated scale for the detection of delirium in newborns and children up to the age of 21 years old. (2) The CAPD scale is not currently translated in French. The objective of this pilot study is to assess the use and relevance of a french version of CAPD obtained by a reverse translation in a single PICU.

**Patients and methods**: The CAPD was translated by a validated method of translation - reverse translation. The first step was a translation from English to French by 4 health providers (doctors and nurses). A 2nd group (3 doctors) made a translation back French to English. A third group (doctors and nurses) compared the two English versions and validated a French translation that is as straightforward as possible to the original version. At each stage, the translations had to be consensual. A translation of the developmental anchor points associated with the scale was also carried out. These two elements were presented to caregivers. The scale was printed on a label and stuck on the back of the care sheet. All children were to be screened twice a day over a two-month period. At the end of this period, the caregivers were interviewed by a questionnaire on its straightforwardness of use and relevance. Free comments were possible for each question.

**Results**: During the study period, 445 scales were filled out of 1241 expected, 439 were filled by the nurses alone. Eighty patients were evaluated. 38 had a CAPD score > 9 and had been diagnosed with delirium. At the end of the period, 27 caregivers were interviewed. CAPD was difficult to use for certain patient categories- deeply sedated patients (5 caregivers 20%), patients with prior neurological impairment (8 caregivers 30%), infants (7 caregivers 26%).

**Conclusion**: It’s the first assessment of the use of a french version of CAPD. A large proportion of the caregivers had no difficulties to use it and think that iss a relevant tool. Therefore, further french studies to determine proper delirium prevalance could and should be performed.

**References**

1. Traube et al. Pediatric Delirium in Critically-Ill Children- An International Point Prevalence Study Crit Care Med 2017
2. Traube C et al. Cornell Assessment of Pediatric Delirium- a valid, rapid, observational tool for screening deliriumin the PICU*. Crit Care Med 2014

**Table 1:** 

N − 27 (%)	yes	no	Don’t know
Difficulties	30% (8)	67% (18)	3% (1)
Scales achievables	78% (21)	7% (2)	15% (4)

**Table 2:** 

N = 27	always	often	sometimes	rarely	never
CAPD releavance for patients in PICU	11% (3)	56% (15)	30% (8)	3% (1)	0%

## Posters

### P-01 Opioid overdoses admitted to the intensive care unit over a 10-year period- clinical features and involved toxicants

#### Bruno Megarbane (*speaker*)^1^, Rhianna Willems^2^

##### ^1^Hôpital Lariboisière, réanimation Médicale et Toxicologique, Paris, FRANCE; ^2^Department of Medical and Toxicological Critical Care, Lariboisière Hospital, Paris, FRANCE

###### **Correspondence:** Bruno Megarbane - bruno.megarbane@lrb.aphp.fr

*Annals of Intensive Care* 2019, **9(Suppl 1)**:P-01

**Introduction**: An opioid overdose crisis is currently ongoing in the North-American continent in relation to the misuse of opioid analgesics and the spread of new fentanyloids on the recreational scene. Data regarding the opioid overdoses in France remain scarce. Our objectives were to describe the opioid overdoses admitted to the intensive care unit (ICU) and characterize the involved compounds, the consequent clinical features, the resulting complications and the patient management.

**Patients and methods**: We conducted a retrospective single-centre observational study including all patients admitted in the ICU during a 10 year period (2008–2018) for an oipioid overdose evidenced by the typical opioid toxidrom (coma, pinpoint pupils and bradypnea) associated to a compatible blood and urine toxicological screening.

**Results**: In 10 years, 231 patients (147 males and 84 females + age, 38 years (33–46) [median (percentiles 25–75)]) were admitted for opioid intoxication in the ICU. The patients presented consciousness impairment [Glasgow coma score of 6 (3–10)], respiratory rate of 11 min (8–15) and pinpoint myosis (90%). Naloxone (64%) and flumazenil (20%) were administered to avoid tracheal intubation + but mechanical ventilation was requested in 36% of the patients [duration- 20 h (11–72)]. Duration of ICU stay was 41 h (24–72). The observed complications included aspiration pneumonia (35%), rhabdomyolysis (26%), acute respiratory distress syndrome (13%), cardiovascular failure (11%), withdrawal syndrome (8%), hospital-acquired infection (4%) and death (1%). Toxicity was related to opioid misuse (52%) versus opioid overdose (48%). Multidrug exposure was found in 100% of the cases and the involved opioids as follows- morphine (44%), heroin (32%), buprenorphine (30%), methadone (26%), codeine (24%), tramadol (6%), d-propoxyphene (6%), fentanyl (1%) and oxycodone (0.5%). Prevalence of the different opioids was mainly influenced by the banning of propoxyphene from the market in 2011 and the consecutive increase in tramadol prescriptions.

**Conclusion**: Our large cohort of opioid poisonings clearly supports the risks of life-threatening consequences in case of misuse or overdose. Mixed opioid use and combination to other legal and illegal drugs are frequent supporting the role of drug–drug interactions in the onset of toxicity in these multidrug poisonings.

### P-02 Seizures resulting from ethanol withdrawal- management and prognosis in the intensive care unit

#### Bruno Megarbane (*speaker*)^1^, Camille Tacquin^2^

##### ^1^Hôpital Lariboisière, réanimation Médicale et Toxicologique, Paris, FRANCE; ^2^Department of Medical and Toxicological Critical Care, Lariboisière Hospital, Paris, FRANCE

###### **Correspondence:** Bruno Megarbane - bruno.megarbane@lrb.aphp.fr

*Annals of Intensive Care* 2019, **9(Suppl 1)**:P-02

**Introduction**: In France, excessive ethanol use represents a major public health problem with resulting morbidities and social and medical expenses. Chronic alcohol drinking induces dependence and tolerance and may thus be responsible for withdrawal syndrome complicated by seizures, explained by ethanol-induced alterations in the brain neurotransmitters including at the gamma-aminobutyric acid glutamine balance. Our objective was to describe the patient’s characteristics, morbidities, management and final outcome of the patients admitted to the intensive care unit (ICU) due to ethanol withdrawal complicated by seizures.

**Patients and methods**: We conducted a retrospective monocentre observational study including all presumed chronic alcoholic patients admitted to the ICU from 2014 to 2018 for ethanol withdrawal-induced seizures. The diagnosis was considered by the physicians in charge based on the results of all useful clinical, biological, imaging and EEG data usually required to rule out alternative diagnoses.

**Results**: Fifty-three patients [45 males (85%) and 8 females (15%) + age 49 years (12) (median (interquartile range)] were included in the study. The patients were homeless (44%) and jobless (52%). They had previously presented ethanol withdrawal syndrome (62%) including seizures (53%). Patients developed status epilepticus (70%) associated to delirium tremens (13%). Complications included aspiration pneumonia (49%), cardiovascular failure (18%, mainly vasoplegic shock requiring norepinephrine infusion), neurological deficits (8%) and skin compression lesions (19%). Patients requested mechanical ventilation (53%) during 3 days (12). All patients were treated with anticonvulsive drugs of which the two mostly used were levetiracetam (Keppra^®^, 80%) and clonazepam (Rivotril^®^, 72%). Two patients (4%) died. Readmission to the emergency department (49%) and re-hospitalization in an ICU or medical ward (38%) were remarkable during the first year following their ICU admission, while only 13% had psychological and addictological follow-up.

**Conclusion**: Ethanol withdrawal may be responsible in the chronic alcoholic for seizures requiring ICU admission with life-threatening complications and death. Our data support once again the absolute necessity to strengthen the fight against excessive ethanol drinking in our society.

### P-03 Quetiapine poisoning admitted to the intensive care unit- complications and usefulness of plasma concentration measurement

#### Bruno Megarbane (*speaker*)^1^, Lamia Sara Chenafi^2^, Karim Jaffal^2^, Marion Soichot^3^, Laurence Labat^3^

##### ^1^Hôpital Lariboisière, réanimation Médicale et Toxicologique, Paris, FRANCE; ^2^Department of Medical and Toxicological Critical Care, Lariboisière Hospital, Paris, FRANCE; ^3^Toxicology laboratory, Paris, FRANCE

###### **Correspondence:** Bruno Megarbane - bruno.megarbane@lrb.aphp.fr

*Annals of Intensive Care* 2019, **9(Suppl 1)**:P-03

**Introduction**: Since its marketing in France in 2011, prescriptions of quetiapine, a second generation antipsychotic drug, has exponentially increased with the risk of increase in the number of acute poisonings. Data regarding acute quetiapine overdose are scarce. Our objectives were 1)- to describe the features, complications and management of acute quetiapine poisonings admitted to the intensive care unit (ICU) + 2- to evaluate the prognostic contribution of plasma quetiapine concentration on ICU admission and model quetiapine pharmacokinetics in overdose.

**Patients and methods**: We conducted a retrospective single-center observational study. All quetiapine-poisoned patients (evidenced by measurement of plasma concentration in the toxic range) admitted to the ICU during a 6-year period (2013–2018) were included. Severe extra-neurological complications were defined as the onset of major ECG abnormalities, catecholamine requirement, cardiac arrest and death. Plasma quetiapine concentration was determined using high-performance liquid chromatography coupled to tandem mass spectrometry.

**Results**: Thirty-eight quetiapine-poisoned patients (11 males and 27 females + median age of 45 years [interquartile range, 16] + presumed ingested dose of 3.0 g [6.9]) were included. Poisoning resulted from multidrug ingestions (97%). The patients presented consciousness impairment (Glasgow coma score of 12 [8] requesting mechanical ventilation in 47% of the cases), seizures (8%), hyperglycemia (68%), hypokalemia (18%), intra- (17%) and atrio-ventricular blocks (7%) and QTc prolongation on the ECG (28%). Complications included cardiovascular failure (13%), aspiration pneumonia (39%) and cardiac arrest (5%). One patient died. The length of ICU stay was 58 h [57]. The plasma quetiapine concentration on admission (685 ng ml [1631]) did not allow predicting the onset of severe complications in the ICU. Delayed quetiapine peak was observed at 27 h [23] post-ingestion suggesting prolonged gastrointestinal absorption while quetiapine elimination was prolonged in comparison to the pharmacological conditions.

**Conclusion**: Quetiapine poisoning is relatively rare but may lead to life-threatening cardiovascular and neurological features. The plasma concentration on admission is not helpful to predict the onset of complications, thus suggesting marked inter-individual variability.

### P-04 About thirteen cases of aluminum phosphide poising

#### Mohamed Walid Mhajba (*speaker*)^1^, Hela Maamouri^2^, Aziz Ben Slimen^2^, Nozha Brahmi^2^

##### ^1^Centre d’Assistance Médicale Urgente (CAMU), Tunis, TUNISIA; ^2^Réanimation polyvalente CAMU, Tunis, TUNISIA

###### **Correspondence:** Mohamed Walid Mhajba - mhajbawalid@hotmail.fr

*Annals of Intensive Care* 2019, **9(Suppl 1)**:P-04

**Introduction**: Aluminum phosphide is a solid fumigant pesticide that can cause serious and potentially life-threatening poisoning. Poisoning is a medical emergency, requiring early and adequate management in a specialized resuscitation environment. The objective of this work was to determine the profile of Aluminum phosphide poisoning and to assess the factors related to mortality.

**Patients and methods**: This is a retrospective study of patients admitted to intensive care unit between 2006 and 2018. The inclusion criteria were based on anamnestic and clinical data of poisoning by Aluminum phosphide.

**Results**: Thirteen cases of aluminum phosphide poisoning were identified. There were 7 women and 6 men. The average age was 27 ± 11 years with extremes of 16 and 51 years. The poisoning was accidental by inhalation for 9 patients and voluntary by ingestion as part of a suicide attempt for 4 patients. The delay between intoxication and admission was, on average, 5 ± 2 h. The Glasgow coma score averaged 14 ± 1. Respiratory signs were at the forefront of inhaling dyspnea and irritative cough, whereas each patient which ingested the toxic were in a shock stage. Abdominal pain and vomiting were present for 8 patients. Electrical abnormalities were present for 5 patients with conduction disturbances (n = 3) and repolarization disorders (n = 2). The increase in troponin was observed with the four patients who ingested the toxic.

Mechanical ventilation was necessary in 5 cases and the use of vasoactive amines for 4 patients. The use of extra-renal cleansing and ECMO were performed for one patient. Four patients died (30%) + the highest mortality was observed after ingestion of Aluminum phosphide (75%). The factors which were significantly related to mortality in univariate analysis, were- oral poisoning (p < 0.001), cardiogenic shock (p < 0.001), elevation of troponin (p = 0.001), and use of ventilatory support (p < 0.004).

**Conclusion**: Acute aluminum phosphide poisoning remains grafted with heavy mortality, especially in developing countries. In the absence of antidotal treatment, rapid hemodynamic management may help improve the prognosis, including the use of circulatory assistance.

### P-05 Poisoning-associated rhabdomyolysis- Risk factors for acute kidney injury and prognosis

#### Bruno Megarbane (*speaker*)^1^, Pierre François Rogliano^2^, Jean-Louis Laplanche^3^, Isabelle Malissin^2^, SébastianVoicu^2^, Nicolas Deye^2^, Laurence Labat ^4^

##### ^1^Hôpital Lariboisière, réanimation Médicale et Toxicologique, Paris, FRANCE; ^2^Department of Medical and Toxicological Critical Care, Lariboisière Hospital, Paris, FRANCE; ^3^Biochemistry department, Lariboisière Hospital, Paris, FRANCE; ^4^Toxicology laboratory, Paris, FRANCE

###### **Correspondence:** Bruno Megarbane - bruno.megarbane@lrb.aphp.fr

*Annals of Intensive Care* 2019, **9(Suppl 1)**:P-05

**Introduction**: Acute kidney injury (AKI) is the major complication after rhabdomyolysis. The risk factors in case of poisoning-related rhabdomyolysis are unknown. Our objectives were 1)- to describe the toxicants involved in the poisoning-associated rhabdomyolysis and the resulting complications + and 2)- to identify the predictive factors of AKI in this setting.

**Patients and methods**: We conducted a monocentre retrospective observational cohort study including all poisoned patients admitted in the intensive care unit (ICU) from 01 01 2012 to 30 06 2018 and presentencing rhabdomyolysis defined by elevated phosphocreatinine kinasee (CPK) > 1000 IU L. AKI was defined using the KDIGO criteria. Factors associated to AKI onset were identified using a multivariate analysis based on a step-by-step logistic regression and their characteristics determined using the Receiver operating characteristic (ROC) curves.

**Results**: Two hundred and thirty-seven patients (138 males 99 females + age- 43 ± 15 years) were included. The main involved toxicants were benzodiazepines (14%), antipsychotics (10%), opioids (9%) and serotonin reuptake inhibitors (8%). AKI occurred in 88 patients (37%), requiring hemodialysis in 43 patients (49% of them). The associated complications included sepsis (51%), cardiovascular failure (14%), cardiac arrest (3%), disseminated intravascular coagulation (2%) and compartment syndrome (1%). The overall mortality rate (13%) was higher in the presence of ARF (32% vs. 2%, p < 0.001). Peak serum creatinine and peak serum CPK were significantly correlated (r2 = 0.3, p < 0.0001 + linear regression) Based on a multivariate analysis, the age (p < 0.01), female gender (p < 0.01), lithium intoxication (p < 0.001), hypocalcemia (p < 0.05), hyperphosphoremia (p < 0.05), elevated serum lactate (p < 0.01) and creatinine concentrations (p < 0.0001) were the independent predictive of AKI on ICU admission. The area under the ROC curve of our predictive model was 0.951. Elevation in serum creatinine concentration > 125 µmol L has the best accuracy (area under the curve- 0.871), sensitivity (0.71) and specificity (0.92).

**Conclusion**: We identified the predictive factors of AKI in the poisoned patient admitted to the ICU and presenting rhabdomyolysis. They may help improving the patient prognosis by intensifying the prevention in the selected patient subgroup at risk of AKI.

### P-06 Acute poisoning in tunisia- which epidemiological profiles in 2017?

#### Mohamed Walid Mhajba (*speaker*)^1^, Hela Maamouri^2^, Hind Allouche^2^, Nozha Brahmi^2^

##### ^1^Centre d’Assistance Médicale Urgente (CAMU), Tunis, TUNISIA; ^2^Réanimation polyvalente CAMU, Tunis, TUNISIA

###### **Correspondence:** Mohamed Walid Mhajba - mhajbawalid@hotmail.fr

*Annals of Intensive Care* 2019, **9(Suppl 1)**:P-06

**Introduction**: Acute poisoning is a real public health problem. Its epidemiological profile varies by region and by time. The objective of this study was to study the epidemiological-clinical, evolutionary and therapeutic profile of acute intoxications admitted to the CAMU resuscitation department.

**Patients and methods**: This is a retrospective study conducted in the intensive care unit between January 1st and December 31st, 2017 and includes cases of acute intoxication hospitalized in intensive care.

**Results**: Seven hundred and sixty-two patients were included in this study with an average age of 31 ± 14 years. There was a clear female predominance with a sex ratio of 0.47. Two hundred and forty-three addicts (32%) had a psychiatric history and 137 (18%) already had a history of attempted suicide. The average time to treatment was 4 +or- 2 h. Four hundred and seventy-one patients (62%) were admitted through CAMU emergencies. Intoxication was voluntary in 85% of the cases and secondary oral ingestion in 91% of them. Toxicology analysis showed that drugs ranked first (n = 486 + 64%), pesticides second (n = 179 + 23.5%) and carbon monoxide third (n = 56 + 7%). For drugs- psychotropic drugs, anticonvulsants, cardiotropics, antidiabetics and paracetamol were the most common causes of drug poisoning with a frequency of respectively 58, 22, 21, 14 and 11%. Whereas for pesticides, rodenticides were predominant (n = 140, 78%) followed by chloralose (n = 137) and organophosphorus (n = 29, 16%). Illegal drugs were criminalized in 15 patients (2%). The predominant toxidrome were respectively anticholinergic (18%) serotoninergic (7%) and cholinergic (6%) toxidrome. Lactic acidosis was the most common biological abnormality (9%) followed by biological inflammatory syndrome (7%) and rhabdomyolysis (7%). Mechanical ventilation was required in 290 cases (38%) with the use of vasoactive amines in 7% of the cases. An antidote was prescribed in 190 cases (25%) and a transit accelerator in 32 patients (4%). The evolution was beneficial for 752 patients (98.4%).

**Conclusion**: The general epidemiological profile in 2017 has shown that psychotropic drugs still predominate with a marked increase in the frequency of cardiotropic and antidiabetic poisoning. In contrast, for pesticides, there is a clear decrease in the number of poisoning by organophosphorus in favor of chloralose.

### P-07 State of play on pesticide intoxication in Tunisia

#### Mohamed Walid Mhajba (*speaker*)^1^, Hela Maamouri^2^, Aziz Ben Slimen^2^, Nozha Brahmi^2^

##### ^1^Centre d’Assistance Médicale Urgente (CAMU), Tunis, TUNISIA; ^2^Réanimation polyvalente CAMU, Tunis, TUNISIA

###### **Correspondence:** Mohamed Walid Mhajba - mhajbawalid@hotmail.fr

*Annals of Intensive Care* 2019, **9(Suppl 1)**:P-07

**Introduction**: Pesticide poisoning has experienced some stability over the years, especially in developing countries. Our objective is to determine the epidemiological, therapeutic and prognostic profile of the most widespread pesticide poisoning in Tunisia for the year of 2017.

**Patients and methods**: This was a descriptive retrospective study of all cases of acute intoxication by pesticides admitted to ICU resuscitation care in 2017.

**Results**: One hundred seventy-nine cases of pesticide intoxication were admitted during the study period which represents 23.5% of all acute intoxication cases admitted to intensive care. The average age of the addicts was 30 ± 13 years with a sex ratio of 0.47. The poisoning were occurring at home in 138 of the cases (77%) or in a public space in 41 of them (23%). The poisoning were voluntary in 174 of the cases (97%) and accidental in 5 of them. Patients came directly from emergencies in 40% of the cases and were transferred from other hospitals in 60% of the cases. Rodenticides were the most implicated type of pesticide (n = 140 + 79%) with chloralose as leader (n = 137, 76%) followed by organophosphorus (n = 29 + 16%) and carbamates (n = 8 + 4%). Neurological signs of tremor and loss of consciousness were predominant (n = 144 + 80%) followed by tachycardia (n = 45 + 25%) and digestive signs (n = 27 + 15%). Respiratory distress was present in 11 patients (6%) and hemodynamic failure in three. Inhalation pneumonia complicated poisoning in 51 cases. The use of mechanical ventilation was necessary in 80% of the cases, the use of vasoactive drugs in 5.6% and the use of antibiotic therapy in 28.5%. Gastric lavage was performed in 9 patients (5%). Atropine was administered in 30 patients (16%). The median length of stay was 2.5 days and the overall mortality was 1.1% (n = 2).

**Conclusion**: Pesticide poisoning is a reality in Tunisia. They are characterized by the variability of the toxic substances available in our country with neurological signs in the foreground. This can be at the origin of major and fatal complications if they are not taken care of in due time and in the appropriate place.

### P-08 Survey on the availability of physiological markers that may guide red blood cell transfusion in critically ill children, on behalf of the Paediatric Intensive Care Society Study Group

#### Camille Jutras (*speaker*)^1^, Geneviève Du Pont-Thibodeau^1^, Marisa Tucci^1^, Simon Stanworth^2^, Samiran Ray^3^, Patricia Fontela^4^, Helen Trottier ^5^, Stacey Valentine^6^, Scot Bateman^6^, Jacques Lacroix^1^

##### ^1^Hôpital Sainte Justine, Montreal, CANADA; ^2^NHS Blood & Transplant Oxford John Radcliffe Hospitals, Oxford, UNITED-KINGDOM; ^3^Great Ormond Street Children’s Hospital, Londres, UNITED-KINGDOM; ^4^Montreal Children’s Hospital, Montreal, CANADA; ^5^Centre de recherche CHU Sainte-Justine, Montreal, Montreal; ^6^University of Massachusetts Medical Cente, North Worcester, UNITED STATES

###### **Correspondence:** Camille Jutras - camille.jutras@gmail.com

*Annals of Intensive Care* 2019, **9(Suppl 1)**:P-08

**Introduction**: There is uncertainty regarding the best measure of the need for red blood cell (RBC) transfusions. Many “physiologic markers” are advocated to support this assessment, in addition to the common standard of haemoglobin level in critically ill children. This survey aims to describe which physiologic markers are measured and available in British and Canadian paediatric intensive care units (PICU).

**Patients and methods**: A pre-piloted self-administered questionnaire was electronically sent out thrice, using Survey Monkey, from November 2017 to February 2018, to the director of 16 Canadian and 27 British PICUs. The list of 17 physiologic markers included in the survey was developed by a group of 42 experts who participated in the “Transfusion and anemia expertise initiative” (Pediatr Crit Care Med 2018 Sept + 19(9S Suppl 1)). Respondents were asked to indicate if the markers listed were available in their PICU.

**Results**: Replies were received from 12 Canadian and 25 British PICUs (37 43 = 86%) + three declined the invitation because only premature infants and newborns were admitted into their unit. Results are presented in Table 1.

**Conclusion**: Many physiologic measurements advocated by paediatric intensivists to support RBC transfusion decision are not routinely available at the bedside. Studies to explore the role of physiologic markers to better define the need for RBC transfusion, in addition to haemoglobin concentration, should include the following more commonly available parameters- pulse oximetry, oxygenation index, oxygenation saturation index, blood lactate and troponin level.

### P-09 The socioprofessional impact on parents after the admission of their child in pediatric intensive care

#### Nabil Tabet Aoul (*speaker*), Ali Douah, Zakaria Zohir Addou, Houari Youbi, Mohamed Moussati, Kamel Belhabich, Amel Zerhouni , Sanae Abada, Souad Mir, Nabil Aouffen

##### EHS Canastel Oran Algeria, Oran, ALGERIA

###### **Correspondence:** Nabil Tabet Aoul - tabetrea@yahoo.fr

*Annals of Intensive Care* 2019, **9(Suppl 1)**:P-09

**Introduction**: Resuscitation units receive children with one or more acute short-term life-threatening failures. Our goal is to focus on the experience of families and the socio-professional consequences that admitted a child in intensive care unit.

**Patients and methods**: Descriptive retrospective study over a period of 03 months from April 2017 to June 2017, in the pediatric resuscitation unit of the pediatric EHS Pr Boukhrofa AEK including children aged under 15 years for management of vital distress.

**Results**: We have 78 children, the average age is 4.5 years (1 to 15 years), the sex ratio is 0.46. 25% of parents have a low intellectual level and 15% a very average socio-economic level. 12% of parents are separated. Admission is in 66% during the night accompanied by the whole family in 77% of the cases. 38% of children have a medical history. The main reasons for admission to resuscitation are respiratory distress in 26%, septic shock 20%, neurological in 14% and traumatic 13%. Of the socio-professional consequences on parents, 62% admit having abandoned the family and 75% of disturbances in the work.

**Conclusion**: Hospitalization of children in pediatric intensive care is an extremely painful experience for parents with social and professional consequences.

### P-10 Pediatric mortality and morbidity at the Intensive Care Unit (ICU) of the Centre Hospitalier Universitaire de Libreville- a retrospective study over 4 **years**

#### Laurence Essola-Rerambiah (*speaker*)

##### NA, Libreville, GABON

###### **Correspondence:** Laurence Essola-Rerambiah - laurenceessola@yahoo.fr

*Annals of Intensive Care* 2019, **9(Suppl 1)**:P-10

**Introduction**: severe medical affections or major surgical interventions in children may necessitate admission in the pediatric intensive care unit. The aim of this work was to evaluate the pediatric mortality and morbidity in the Intensive Care Unit.

**Patients and methods**: This was a retrospective, descriptive and analytical study done over 4 years, from January 2012 to December 2015. Included were children from one month to 16 years who were hospitalized for 24 h or more. Studied variables included social and demographic data, clinical, paraclinical, and prognostic data.

**Results**: during the studied period, 229 children were admitted to the ICU, that is 16.1% of admissions. 218 children (15.4%) fitted our criteria for inclusion. Female population presented 52.1% of total. Mean age of patients was 90.6 ± 68.1 months.13 patients (5.9%) were homozygotes SS and had multiple transfusions. Reasons for admission included altered consciousness with fever or without and severe respiratory distress in 25.6% and post-operative care (23.7%). Main pathologies recorded were severe malaria (10.1%), bronchopneumopathies (7.8%), meningitis and meningo-encephalitis (7.8%). Severe maternal pathologies represented 10.6% of admissions. The mean duration of hospitalization was 5.2 days with extremes of 1 and 46 days. Mortality was 27.5%. Factors for bad prognosis were mechanical ventilation (p = 0.00), meningitis and meningo-encephalitis.

**Conclusion**: The infectious pathologies form the main causes of admission of pediatric patients to the ICU. The knowledge of the main causes of referral and of deaths should allow us a better orientation of preventive strategies and a better management of our patients in our ICU.

### P-11 Impact of early drainage of primary spontaneus pneumothorax (PSP) in Young adults

#### Myriam Jugie (*speaker*), Olivier Bustarret, Guillaume Geslain, Julien Grassias, Maxime Eloi, Nadege Salvi, Gilles Orliaguet

##### Hôpital Necker enfants malades, Paris, FRANCE

###### **Correspondence:** Myriam Jugie - Mjugie@free.fr

*Annals of Intensive Care* 2019, **9(Suppl 1)**:P-11

**Introduction**: The primary spontaneous pneumothorax (PSP) Is an idiopathic pneumothorax without pulmonary pathology. The PSP ´s prevalence Is rare - 2.68 in 1997 and 3.41 for 10 000 children in 2006 in United States of America. There is no international consensus for the treatment of PSP. The aim of our study was to retrospectively analyze initial treatment of this PSP in Young adults.

**Patients and methods**: Between 2010 and 2017, all the children with a PSP who came from home to our hospital in emergency were eligible. Âge, sex, kind of pneumothorax, needle ponction, thoracic CT scan realization, Time limit between PSP diagnosis and thoracic drainage, type of drain used, duration of drainage according to the type of drain and eventual recurrences were studied.

**Results**: Fifteen children âged from 9.5 to 15 years (median 14 years) boys mostly (93%) had had a PSP during this period. Among The 15 children, 13 needed a thoracic drain, 2 were simply observed. All The 4 patients who had a complete PSP and had a needle exsufflation needed a thoracic drain. Among The 15 children, 6 had emphysema bubbles on the Thoracic CT scan. (40%). Time limit between PSP diagnosis and drainage was between 1–7 days. (CI 95% 0–7). The duration of drainage according to type of drain was 4.1 days for a Furhman’s drain and 9.4 days for a Monod’s drain. Among the 5 PSP treated with a Monod’s drain, there was 4 recurrences. Among the 8 other PSP drained with a furhman’s system, there was 4 recurrences.

**Conclusion**: Our study showed clearly that exsufflation of large PSP in pediatric intensive care without vital Life issue Is useless and delayed the treatment. Thoracic CT scan in a large PSP or after recurrency Is strongly recommended because of emphysema bubbles diagnosis. Furhman’s drain Is mainly used in the ICU instead of Monod’s drain mostly used by the surgeons. We must now confirm all this by a prospective study with other national or international pediatric intensive care center.

### P-12 Monitoring of Procalcitonin plasma levels evolution could be useful for safely shortening duration of antibiotic treatment in pediatric ventilator-associated pneumonia

#### Olivier Lintz (*speaker*)

##### CHU - Service de réanimation médico-chirurgicale pédiatrique spécialisé, Strasbourg, FRANCE

###### **Correspondence:** Olivier Lintz - olivlintz@gmail.com

*Annals of Intensive Care* 2019, **9(Suppl 1)**:P-12

**Introduction**: Ventilator associated pneumonia (VAP) is a common infection in Pediatric Intensive Care Units (PICUs) and is associated with significant morbidity and mortality. Previous studies suggest that Procalcitonin (PCT) follow-up in VAP may be helpful for reducing duration of antibiotic therapy in adults intensive care patients in order to lower the risk for selection of antibiotic (ATB)-resistant bacteria.

**Patients and methods**: Our main objective was to study the impact of monitoring the PCT plasma levels at day 0 (D0) and day 3 (D3) on the duration of antibiotic treatment in cases of VAP in the Strasbourg University Hospital PICU (France). We prospectively (2016–2018) included hospitalized patients in our unit that developped VAP according to the 2005 and 2016 Infectious Diseases Society of Amarica American Thoracic Society guidelines. Duration of PCT-guided antibiotic treatment was compared to retrospectively collected data in patients treated for VAP between 2014 and 2016.

**Results**: Five patients were included prospectively with a mean duration of ATB of 4.8 days [3–8], compared with 8.7 days [4–15] in the control group (n = 10). The différence was found to be statistically significant (p = 0.002). There was no treatment failure or death attributable to VAP in both groups.

**Conclusion**: These results tend to show that the PCT follow-up between D0 and D3 of VAP diagnosis could favor short-term antibiotic duration in PICUs.

### P-13 Use of biomarkers to improve the diagnostic accuracy of concomitant bacterial infections in critically ill infants with viral bronchiolitis - a pilot study

#### Patricia Fontela (*speaker*), Patricia Papenburg, Guillaume Emeriaud

##### McGill University, Montreal, CANADA

###### **Correspondence:** Patricia Fontela - patricia.fontela@mcgill.ca

*Annals of Intensive Care* 2019, **9(Suppl 1)**:P-13

**Introduction**: Bronchiolitis is the most common lower respiratory tract infection in children < 12 months of age, with 2–12% of patients requiring admission to a pediatric intensive care unit (PICU). Despite its predominantly viral etiology, the use of antimicrobials in PICUs for bronchiolitis is widespread. This pilot prospective cohort study aims to compare the levels of infection markers in critically ill infants with bronchiolitis with and without concomitant bacterial pneumonia.

**Patients and methods**: This is a pilot prospective cohort study including 37 patients < 2 years old diagnosed with a first episode of viral bronchiolitis and admitted to 3 Canadian PICUs between November 1st 2017 and April 30th 2018. We collected data on patient demographics, severity scores, diagnostic criteria for viral bronchiolitis and concomitant bacterial infections, and bacterial culture results. We gathered data on the following infection biomarkers during the first day of PICU admission- temperature, procalcitonin, CRP, and white blood cell count (WBC). We analyzed our preliminary results using descriptive statistics.

**Results**: Our preliminary results include the analysis of data collected for 37 patients. Participants mean age was 1.5 ± 2.3 months. Fifteen (40%) patients were positive for respiratory syncytial virus. Three (8.1%) patients had bacterial pneumonias confirmed by culture results + no other bacterial infections were observed. Twenty (57%) patients received antimicrobials (mean duration 1.9 ± 2.9 days). Regarding infection biomarkers on study day 1, 8 (12%) patients presented fever and 1 (0.3%) hypothermia + the mean levels for procalcitonin and CRP were 1.0 ± 1.7 ng mL and 32 ± 39 mg L, respectively + mean WBC was 9.4 ± 3.6 109 cells L. The mean levels of procalcitonin (1.25 ng mL vs. 1.37 ng mL) and WBC (9.52 109 cells L vs. 8.4 109 cells L) were similar between patients with bronchiolitis and patients with bronchiolitis and concomitant bacterial pneumonia. The mean level of CRP was higher in patients with with bronchiolitis and concomitant bacterial pneumonia (34.12 mg L vs. 81.70 mg L).

**Conclusion**: CRP may be a suitable biomarker for the diagnosis of concomitant bacterial pneumonia in critically ill infants with bronchiolitis.

### P-14 Population pharmacokinetics of meropenem in critically ill children with different renal function

#### Mélanie Rapp (*speaker*)^1^, Saïk Urien^2^, Sihem Benaboud^3^, Emmanuelle Bille^1^, Naïm Bouazza^2^, Déborah Hirt^3^, Inès Gana ^3^, Yi Zheng^3^, Fabrice Lesage^1^, Agathe Béranger^1^,Sylvain Renolleau^1^, Jean-Marc Tréluyer^3^, Mehdi Oualha^1^

##### ^1^Hôpital Necker, Paris, FRANCE; ^2^Site Tarnier, Paris, FRANCE; ^3^Hôpital Cochin, Paris, FRANCE

###### **Correspondence:** Mélanie Rapp - melanie.rapp23@gmail.com

*Annals of Intensive Care* 2019, **9(Suppl 1)**:P-14

**Introduction**: Usual dosing regimens of meropenem could lead to the suboptimal exposure since the pharmacokinetic (PK) between subject variability (BSV) is particularly high in critically ill children with sepsis. We aimed to develop a population PK model then simulate dosing regimens to optimize patient exposure.

**Patients and methods**: This prospective study was conducted in a pediatric intensive care unit (PICU) center at the Necker Hospital. All children aged < 18 years and weighing > 3.5 kg were enrolled. Meropenem plasma concentration was quantified by high performance liquid chromatography. Meropenem PK model was developed using non linear mixed-effect modeling software (MONOLIX version 2018R1). Estimated glomerular filtration rate (eGFR) was estimated by the Schwartz formula.

**Results**: Forty patients (median age of 16.8 months (1.4–187.2), median weight of 9.1 kg (3.8–59) and median eGFR of 151 mL/min 1.73m2 (19–440)) were included. Eleven patients (27%) received continuous replacement renal therapy (CRRT). Data were best described with a two compartment and first order elimination model. Bodyweight (BW) with the allometric relationship, eGFR and CRRT were significant covariates explaining the BSV on clearance (CL), inter compartment clearance (Q) and central peripheral volume of distribution (V1 V2)- V1i = V1pop x (BW 70)1, Qi = Qpopx (BW 70)0.75, V2i = V2pop x (BW 70)1, CLi = (CLpop x (BW 70)0.75) x (eGFR 151)0.355) and for patients with CRRT + CLi = (CLpop x (BW 70)0.75) x 0.8 + were population CL, V1, Q, and V2 are- 7.93 L.h-1, 37.9 L, 1 L.h-1 and 9.66 L. Monte Carlo simulations were performed to provide the highest probability of reaching the target of 50% fT > MIC and 100% fT > MIC depending on renal function. Continuous infusion (both 60 and 120 mg kg per day) seemed to be the best dosing regimen for high MIC value (> 4 mg L) without risk of accumulation except in children with severe renal failure without CRRT. Usual dosing regimens (20 mg kg 8 h over 20 min) are adequate for only patients with a severe renal failure regardless of the MIC value.

**Conclusion**: Continuous infusion allows to reach the targets of fT > MIC (for bacteria with a high MIC value) with safety for children with normal or augmented renal clearance and who received CRRT. Usual dosing regimens should be kept for children with severe renal failure regardless of MIC value.


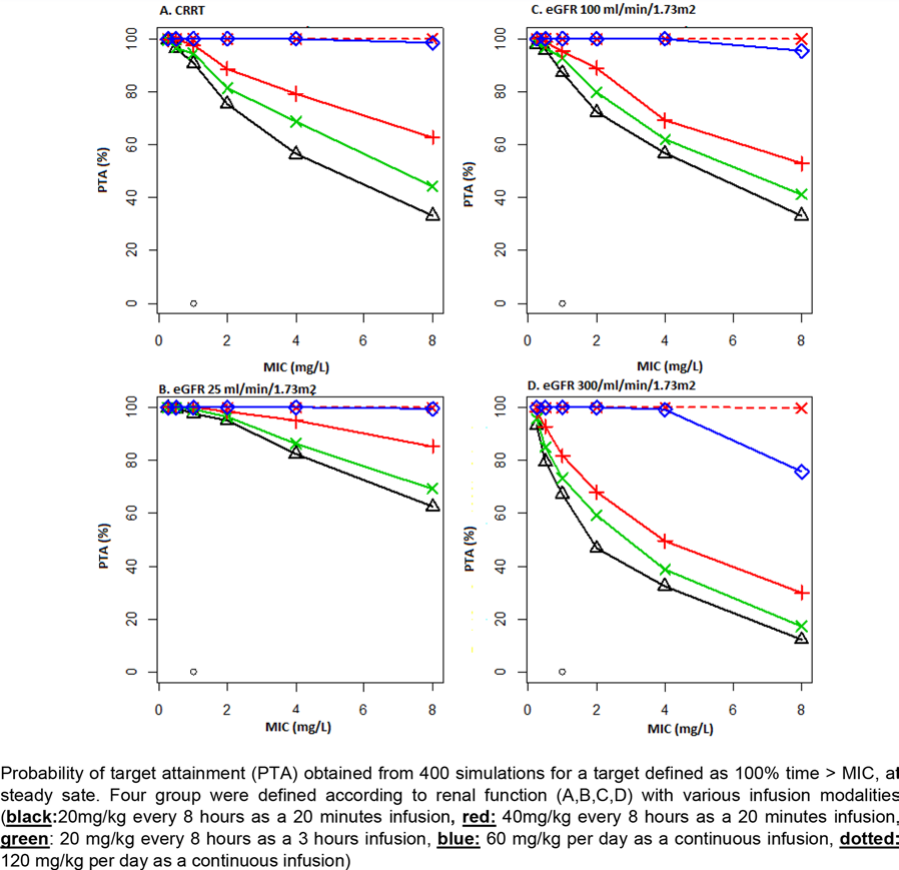


### P-15 Development of a French-Speaking Pediatric Intensive Care Unit Registry- PICURe

#### Stéphane Leteurtre^1^, Aurélie Aldebert^2^, Jean-Benoit Baudelet^1^, Francis Leclerc^1^, Jean-Marie Renard^1^, Stephane Dauger^1^, François Dubos ^3^, Morgan Recher^1^, Hervé Hubert^4^, Gfrup^1^

##### ^1^CHU Lille - Réanimation Pédiatrique, Lille, FRANCE; ^2^Registre des Arrêts cardiaques, Lille, FRANCE; ^3^CHU Lille-Urgences pédiatriques, Lille, FRANCE; ^4^Faculté d’Ingénérie et de Management de la Santé (ILIS), Lille, FRANCE

###### **Correspondence:** Stéphane Leteurtre- stephane.leteurtre@chru-lille.fr

*Annals of Intensive Care* 2019, **9(Suppl 1)**:P-15

**Introduction**: Medical databases and patient registries have expanded considerably, particularly in pediatric critical care medicine (PICANet –UK-, ANZPIC Registry Australia and VPS –USA-). Such a registry is not yet available in French-Speaking countries. The aim of this study was to develop a French-speaking Registry for Pediatric and Pediatric&Neonatal PICU P&NICUs in France to determine structure, personnel and outcome, and to propose an actualized common email directory.

**Patients and methods**: 34 potential French Pediatric and Pediatric Neonatal ICU identified by the GFRUP were solicited. Previously, the necessary variables that ought to be included in a French pediatric critical care registry were identified (Recher, J Eval Clin Pract 2018). Collected variables- hospital and service characteristics (Pediatric, neonatal, Middle Care unit –MCU-, ICU activity characteristic, number of beds, personnel (medical, paramedical -nurse-, number and full-time equivalent –FTE-), patients (number of patients in 2017 and outcome -survivors non survivors-). A secured web site was created. Each head of PICU P&NICU can complete the online data in a secured common email directory.

**Results**: 29 French PICU or P NPICU were included (incomplete data-n = 3). 14 PICU and 15 N&PICU were identified with 23 MCU associated. Pediatric Number of bed varied from 2 to 16 (total pediatric beds = 235 in 29 PCIU or N&PICU) and Neonatal number of beds varied from 8 to 18 (total neonatal beds = 154 in 15 N&PICU). In PICU or N&PICU, median nurse ratio “FTE number of beds” was 2.7 (range 2.02–3.91). In 2017, 11481 pediatric patients were admitted in 26 PICU or N&PICU (mean 393, range 51–841) with non survivors patients (n = 513, range 3–50) i.e. mean mortality rate of 4.5% (range 1.3–11.2). In 2017, 3146 neonates were admitted in 12 N&PICU (mean 262, range 136–458) with non survivors neonates (n = 260, range 3–38) i.e. mean mortality rate of 8.7% (range- 1.4–16).). Mail directory was completed by 240 physicians (n = 26PICU N&PICU).

**Conclusion**: In 2018, the development of a French-speaking Pediatric Intensive Care Unit Registry named PICURe provides a large description of almost French PICUs and N&PICUs, in term of structure and mortality, and an updated and secure mail directory. The next stage will be the implementation of the collection of the patients’ data.

### P-16 Positive multidrug resistant bacteria at admission as a predictive factor of mortality in critically ill patients

#### Khaoula Ben Ismail (*speaker*), Ines Fathalah, Ghada Sboui, Sahar Hbecha, Ameni Sghier, Asma Mahdi, Emna Nouri , Haifa Fazeni, Nadia Kouraichi

##### Hôpital Ben Arous Yassminet, Intensive care unit, Ben Arous, TUNISIA

###### **Correspondence:** Khaoula Ben Ismail - khaoula87@hotmail.fr

*Annals of Intensive Care* 2019, **9(Suppl 1)**:P-16

**Introduction**: The pandemic spread of multidrug-resistant bacteria (MDRB) pose a threat to healthcare Worldwide especially in intensive care unit (ICU). The aim of our study was to investigate the relation between a positive MDRB at admission with infection as well as prognosis among patients admitted in medical ICU.

**Patients and methods**: We conducted a retrospective cohort study included 224 patients admitted to the medical intensive care unit of YASMINET hospital between 1 th September 2016 and 15 th September 2018. We collected the demographic characteristics of patients (Sex, age, IGSII, SOFA, comorbidities…) and the characteristics of infection (number of episods, site, germs, screening MDRB at admission), we also analyzed the length of stay and mortality rate.

**Results**: We enrolled 224 critically ill patients. The mean age of our population was 55.1 ± 20.54 years. The sex ratio was M F = 1.8. The mean IGSII and SOFA scores were respectively- 13 ± 9.23 and 5 ± 4.07. Seventy-three patients (33%) required machanical ventilation. The average of duration of mechanical ventilation was 7, 78 ± 14, 81 days. The screening of MDRB at admission was done in 87.1% (195 cases). Fourty-four patients (19, 6%) had positive MDRB at admission. After univariate analysis, a positive MDRB at admission was significantly associated with- a frequent incidence of infection (30 of 44 cases), a prolonged length of stay (29 ± 23.75 days versus 9.4 ± 10 days) and a higher rate of mortality (18 46 cases) with respectively p < 10^−3^, p < 10^−3^ and p = 0.002. With multivariate analysis, only prolonged length of stay was significantly associated with positive MDRB at admission.

**Conclusion**: A positive MDRB at admission can be a predictor factor of infection, length of stay and mortality in critically ill patients.

### P-17 Establishment of monitoring of colonization by BGN carbapenemases in burn victims in Tunisia

#### Lamia Thabet (*speaker*), Yosra Bourbiaa, Beya Maamar, Khalil Jmal, Imen Rahmani, Amen Allah Messadi

##### Centre de traumatologie et des grands brûlés ben arous, Tunis, TUNISIA

###### **Correspondence:** Lamia Thabet - thabetlamia@gmail.com

*Annals of Intensive Care* 2019, **9(Suppl 1)**:P-17

**Introduction**: Burn patients are at risk for multi-drug resistant (MDR) infections, burdened with a high mortality rate. The aim of this study was to establish the follow-up of digestive colonization by carbapenemase-producing Gram negative bacilli (BGN-PC) in patients admitted to the Burn intensive care unit.

**Patients and methods**: Our study was conducted over a period of 18 months (02 2017–07 18). It focused on rectal samples taken from burns in search of BGN-PC. GeneXpert multiplex real-time PCR (Cepheid) was performed directly on the rectal swabs by Cepheid’s Gene Xpert Carba-R allowing detection of the 5 most prevalent carbapenemase gene families- blaVIM, blaNDM, blaIMP, blaOXA-48 and blaKPC.

**Results**: During the study period, 150 patients underwent rectal swab carbapenemase screening on admission. 95 PCRs (63.3% of the cases) were positive. Among the patients who had a positive PCR on admission, 114 were transferred from another hospital (76%), mainly hospitals in the south of the country. The study of the molecular profile of isolated carbapenemases showed the predominance of a positive result for 3 blaVIM + blaNDM + blaOxa48 genes in 41 cases (i.e. 43.2%), 12 cases were positive for the blaNDM gene (12.6%), 11 cases were positive for the blaVIM gene (11.6%), 9 cases with both blaVIM + blaNDM genes (9.5%), 9 blaOxa48 gene-positive cases (9.5%), 7 blaVIM + blaoxa48 genes (7.4%), and 5 blaNDM genes + blaoxa48. Parallel BMR research using conventional bacteriology confirmed the presence of carbapenem-resistant BGNs in PCR-positive specimens.

**Conclusion**: 63.3% of the patients explored are carriers of carbapenemases. 76% of these patients were transferred from another hospital in Tunisia. This highlights, on one hand, the need of an isolation policy during the care of transferred patients and, on the other hand, the need to set up a control strategy for the spread of these MDRs.

### P-18 Screening of multi-drug resistant bacteria among critically ill patients - two sites versus five sites

#### Khaoula Ben Ismail (*speaker*), Ines Fathalah, Ghada Sboui, Sahar Hbacha, Ameni Sgahier, Asma Mahdi, Haifa Fazzeni , Emna Ennouri, Nadia Kouraichi

##### Hôpital Ben Arous Yassminet, Intensive care unit, Ben Arous, TUNISIA

###### **Correspondence:** Khaoula Ben Ismail - khaoula87@hotmail.fr

*Annals of Intensive Care* 2019, **9(Suppl 1)**:P-18

**Introduction**: Use, overuse and misuse of antibiotics led to emergence of multidrug-resistant bacteria (MDRB) witch is a real problem of public health. To fight against this real scourge, screening is the best tool. We conducted a study to evaluate the efficency of two MDRB screening ways.

**Patients and methods**: Our study was retrospective, it included 224 patients admitted in a medical intensive care unit (ICU) during the periode between September 2016 and September 2018. We collected patients demographic characteristics (Sex, age, IGSII, SOFA, comorbidities…) and infection characteristics (episods, site, germs…). We used two ways of MDRB screening - taking samples from two sites (nasal and rectal) (Janury 2018–September 2018) versus five sites (nasal, buccal, axillary, inguinal and rectal) (October 2016- December 2017).

**Results**: We enrolled 224 critically ill patients. The average age was 55.1 ± 20.54 years. The sex ratio was of 1.8. The median IGSII and SOFA scores were respectively 35 [23 + 49] and 4 [2 + 7]. Seventy-three patients (33%) required mechanical ventilation. Its median duration was of one [1, 9] day. The 2 sites method of screening MDRB was realised in 36.2% (81 cases) while the 5 sites method was practiced in 53.1% (119 cases). Ninety-five episods of infection were detected. The most frequent sites of infection were - pulmonary (13.8%), vascular (12.6) and urinary (11%). Multivariate study reaveled that the two sites method led to adapted antibiotherapy in 68% of cases versus only 36% in the five sites method (p = 0.008).

**Conclusion**: We concluded that two sites MDRB screening method was efficient to detect nosocomial infections and to adjust emperic antibiotherapy. It helped to reduce management costs.

### P-19 The «threatening» water- a potential source for an outbreak of Stenotrophomonas maltophilia in a French intensive care unit

#### Flora Delamaire (*speaker*)^1^, Antoine Caubel^2^, Nathalie Prades^2^, Liliane Grolier-Bois^2^, Pierre-Yves Donnio^1^

##### ^1^CHU RENNES, Rennes, FRANCE; ^2^CH LORIENT, Lorient, FRANCE

###### **Correspondence:** Flora Delamaire - flora_d7@hotmail.com

*Annals of Intensive Care* 2019, **9(Suppl 1)**:P-19

**Introduction**: Stenotrophomonas maltophilia (SM), a non-fermenting gram-negative bacterium (NF-GNB), causes serious opportunistic infections and represents a major threat on intensive care unit (ICU) due to its multidrug resistance (MDR). An outbreak of SM occurred between September 2016 and January 2017 in the ICU of a general hospital. An outbreak control team was convened to investigate, at the same time as an epidemiological survey was conducted and corrective measures were initiated.

**Patients and methods**: The outbreak was investigated by a combination of clinical and environmental samplings and molecular typing using pulse-field gel electrophoresis (PFGE). All patients infected or colonized with SM during this period were included and clinical data were analyzed.

**Results**: Sixteen patients contaminated with SM were included. Among them, 13 had pulmonary contaminations and one bacteraemia. Two deaths probably related to SM infections were reported. Admission screening showed that the outbreak strains were acquired on ICU. The median time to SM contamination was 19.5 days (range 2–43 days). Thirteen patients (81.2%) were intubated, with a median length of 29 days of intubation (range 2–120 days). All patients received multi-antibiotic treatments including broad-spectrum antibiotics [Table]. SM clinical strains genotyping found the same SM genotype for only 3 patients. Environmental sampling revealed contamination of almost all sinks in the ICU- 24 of the 28 sinks in the ICU were contaminated with SM (85.7%), including 15 sinks among the 16 patient rooms (93.7%). Further environmental screening showed an early recontamination after siphons and plugholes had been replaced. Infection control measures, including the eviction of tap water for patients’ cares and a strengthening of preventive hygiene measures, made it possible to control the outbreak by January 2017.

**Discussion**: PFGE results suggest that this outbreak was not related to a unique clone of SM but to a polyclonal colonization of the ICU water system. SM causes severe infections in debilitated patients and is a source of inappropriate antibiotherapy. Should an anti-SM antibiotic be integrated in the probabilistic antibiotherapy within an ICU contaminated with SM?

**Conclusion**: This outbreak report highlights the risk from MDR gram-negative bacterium contaminated water system for ICU patients. Although the complete eviction of the environmental pathogen was impossible, a discontinuation of the outbreak was obtained with infection control procedures, pedagogy and reasoned evictions.


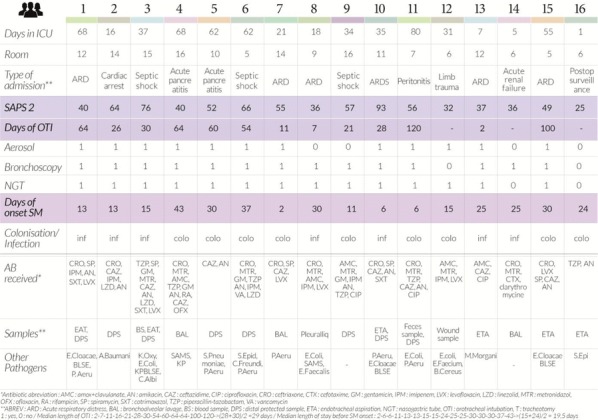


### P-20 Usefulness of repeating routine screening of faecal carriage of multidrug resistant bacteria in ICU patients

#### Ahmed El Kalioubie (*speaker*), Aurelien Donze, Ludivine Wozniak, Frédéric Wallet, Saad Nseir

##### CHRU LILLE, Lille, FRANCE

###### **Correspondence:** Ahmed El Kalioubie - ahmed.elkalioubie@chru-lille.fr

*Annals of Intensive Care* 2019, **9(Suppl 1)**:P-10

**Introduction**: To reduce the incidence of ICU infections with extended-spectrum beta-lactamase-producing (ESBL) and extensively drug resistant (XDR) bacteria, a routine screening for faecal carriage is recommended for all patients and specific contact precautions are applied in colonized patients. The aim of this study is to assess the usefulness of repeating routine screening of these bacteria in ICU patients.

**Patients and methods**: Retrospectively, we studied all patients admitted to our 5 mixed 10-beds ICU units, between January 2014 and December 2016. Systematically, screening was done by anal swab at admission and weekly thereafter, targeting ESBL Enterobacteriaceae, XDR bacteria- Carbapenemase-producing Enterobacteriaceae and Carbapenem-resistant Acinetobacter baumannii, and Pseudomonas aeruginosa. The incidence was assessed and compared over the 3 study years (trend chi2 test).

**Results**: Among the 3684 study patients, 971 (26.4%) were screened positive for multi-drug resistant bacteria (19.8/1000 ICU days). Incidence was 23% in 2014 (17.3/1000 ICU days), 28% in 2015 (20/1000 ICU days), and 28% in 2016 (22/1000 ICU days) (p = NS). This positive carriage was acquired in ICU for 540 patients (14.7%, 11/1000 ICU days)- 158 patients in 2014 (13%, 9.7 1000/ICU days), 169 patients in 2015 (14.5%, 10.5 1000/ICU days), 213 patients in 2016 (16.4%, 13/1000 ICU days). Over the 3 study years, 61% of these patients were male with a mean age of 61(49, 69). Table 1 describes the distribution of multi-drug resistant bacterial faecal carriage over the 3 study years. Acinetobacter baumannii was found as a lone entity in 13 patients, Pseudomonas aeruginosa in 23 patients. Among the 848 ESBL positive patients, further XDR carriage was diagnosed simultaneously in 46(1.2%) patients, and later in 37(1%) patients. Faecal carriage became positive within 2(1, 3) weeks of ICU admission. None of the positive patients became bacteria-free in subsequent anal swabs analysis. After diagnosis of faecal carriage, we pursued weekly anal swabs with a median of 1(0, 3) swabs and a total of 1907 swabs over the 3 study years. With an average 120 euros per anal swab analysis, this represents an additional cost of 228840 euros.

**Conclusion**: Multidrug resistant bacterial faecal carriage is common in our ICU. Once diagnosed, further screening may not be pursued and contact precautions should only be applied. This would probably result in significant cost reductions, with no side effects.


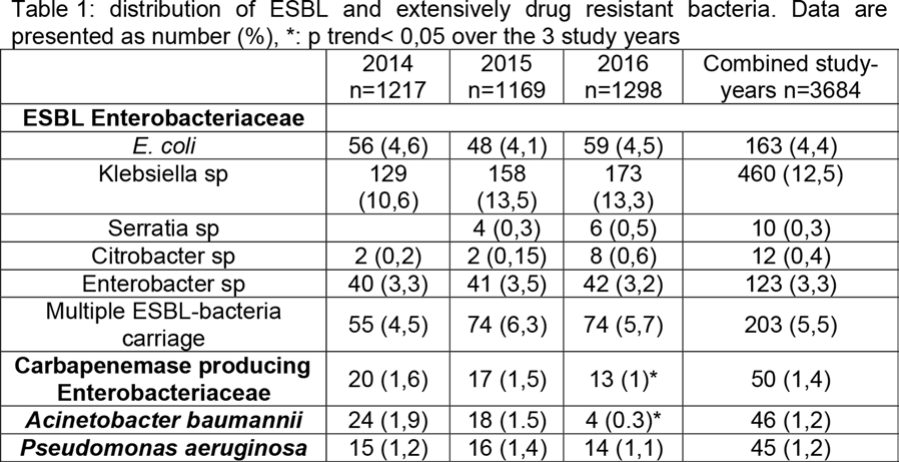


### P-21 Identification of fungi isolated from intensive care unit surfaces

#### Wahiba Imene Ghomari (*speaker*), Yassine Merad

##### CHU Abdelkader Hassani, Sidi-Bel-Abbes, ALGERIA

###### **Correspondence:** Wahiba Imene Ghomari - ghomari_wahiba@yahoo.fr

*Annals of Intensive Care* 2019, **9(Suppl 1)**:P-21

**Introduction**: Nosocomial fungal infections could arise from exposure to spores or filamentous fungi existing in the hospital environment.

**Patients and methods**: The present study aimed to investigate the quality of surfaces in an intensive care unit, to ascertain their potential contribution to fungal infection rate in the hospital. During one morning (28 05 2017) punctual samples were taken from surfaces by moistened sterile swabs and were inoculated on Sabouraud dextrose and Malt extract agar. Qualitative evaluation of moulds was based on the microscopic view and morphological features of colonies, identification of yeasts was based on routine biochemical tests (Auxacolor-Bio-Rad).

**Results**: Out of 100 samples taken from surfaces, 24 were positive for fungal presence, divided into moulds (75%) and yeasts (25%), the identified strains belonged to 9 genera, most frequently to Aspergillus, Candida, Mucor, Cladosporium, Penicillium and Trichosporon, the most predominant fungi encountered is Aspergillusniger (25%).

**Conclusion**: To achieve our professional service and provide a safe environment for patients, punctual fungal monitoring needs to be done consistently.

### P-22 Identifying risk factors for multidrug resistance Gram-negative bacilli pneumonia in intensive care units

#### Wafa Ibn Saied (*speaker*)^1^, Bertrand Souweine^2^, Claire Dupuis^3^, Benoit Misset^4^, Shidasp Siami^5^, Carole Schwebel^6^, Jean-Marie Forel ^7^, Stéphane Ruckly^3^, Michel Darmon^8^, Jean Reignier^9^,Lila Bouada^3^, Jean-François Timsit^3^

##### ^1^INSERM, Paris, FRANCE; ^2^CHRU, Clermont-Ferrand, FRANCE; ^3^INSERM 1137 IAME équipe 5 + HU PARIS NORD SITE BICHAT APHP, Paris, FRANCE; ^4^CHU, Rouen, FRANCE; ^5^CH Sud Essonne site Etampes, Paris, FRANCE; ^6^CHU, réanimation médicale, Grenoble, FRANCE; ^7^CHU, Marseille, FRANCE; ^8^University Hospital, Saint-Etienne, FRANCE; ^9^CHU, réanimation médicale, Nantes, FRANCE

###### **Correspondence:** Wafa Ibn Saied - essaied.wafa@gmail.com

*Annals of Intensive Care* 2019, **9(Suppl 1)**:P-22

**Introduction**: Risk factors of multidrug resistant (MDR) gram negative VAP (GN VAP) are not fully known. In particular the ICU-resistance rate that increases this risk is not known and postulated to be 10 to 25% according to recent published guidelines. Objective- To investigate the risk factors of MDR in GN-VAP.

**Patients and methods**: Adult patients admitted between January 1997 and February 2016 in 17 French ICUs contributing to the OUTCOMEREA prospective database were included. Diagnosis required positive culture of quantitative tracheal aspirate (> 106 cfu ml), plugged telescoping catheters (> 103 cfu ml) or bronchoalveolar lavage (> 104 cfu ml). The rate of MDR-GN GN infections was calculated for each ICU and used as an ICU-variable. Patient-based variables included severity, procedure use, previous antimicrobial use (as  % of days with use), and previous colonization with MDR. A generalized mixed model with binomial distribution was built to assess the risk factors of MDRGN-VAP.

**Results**: A total of 1,335 VAP episodes were studied including 935 GN-VAP episodes. Among them, 634(67.8%) MDR-GN organisms and 301 (32.2%) only susceptible GN organisms. Treatment was more frequently adequate within 24 h in susceptible GN-VAP (229(76%) vs 395 (62%), p < 0.01). Risk factors of MDR-GN VAP are on Table. A rate of 15% or more of MDR-GN infections in the ICU was independently associated with MDR-GN-VAP.

**Conclusion**: In this large cohort of GN-VAP patients, chronic respiratory diseases, previous colonization with MDR GNB and previous antimicrobial use were independent risk factor of MDR-GN-VAP. A rate of MDR GN infections of more than 15% in the ICU is also independently associated with this risk.


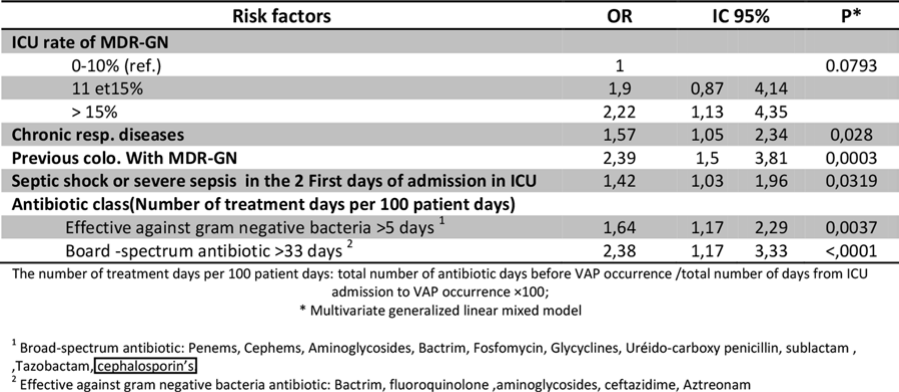


### P-23 Fatal micro outbreak due to NDM-producing Klebsiella Pneumonia in a tertiary belgian ICU

#### David Grimaldi (*speaker*), Martin Vandeputte, Leda Nobile, Alexandre Brasseur, Fabio Taccone, Delphine Martiny, Maya Hites , Baudouin Byl, Jacques Creteur, Frédérique Jacobs

##### Hôpital Erasme, Bruxelles, BELGIUM

###### **Correspondence:** David Grimaldi - david.grimaldi@erasme.ulb.ac.be

*Annals of Intensive Care* 2019, **9(Suppl 1)**:P-23

**Introduction**: Carbapenemase-producing Enterobacteriaceae (CPE) represent a growing problem worldwide. These pathogens are resistant to most of available antibiotic, therefore challenging to treat. New Delhi metallo-β-lactamase (NDM) can hydrolyze penicillins, cephalosporins, and carbapenems1. First isolated from a patient travelling in India in 20082, it has now been described all around the world. In our intensive care unit (Erasme Hospital, Brussels, Belgium), an epidemic of 5 cases in 4 months occured. The aim of this study is to describe characteristics and evolution of colonized infected patients.

**Patients and methods**: We screened all patients with colonization infection with CPE-producing Klebsiella Pneumoniae (KP) within a range of 6 months around identification of the first case. We included the patients with confirmed NDM-KP (by genotyping). We described patient’s profile, previous antibiotic use, infection type, treatment and outcome.

**Results**: Five patients had at least one sample positive with NDM-KP in our ICU between November 2017 and March 2018. Strains were susceptible to fosfomycin, some also to colistin and chloramphenicol. We didn’t perform genotyping. Detailed features are listed in table 1. Patients were young (mean age- 55 years), but suffered from severe medical conditions. 4 out of the 5 patients had stayed in the same ICU subunit. 4 out of the 5 patients presented an infection related to the NDM-KP. Median-time between colonization and infection was 9 days (4–63). All patients had received previous broad-spectrum antibiotherapy. Infections were treated with rarely used antibiotics, highly toxic. We observed microbiological success in 3 patients, relapse in 2. The 4 infected patients died. Cause of death was untracktable infection in 3, comorbidities in 1. The colonized patient survived (mortality rate- 80%).

**Conclusion**: NDM-producing Enterobacteriaceae is emerging in Europe.3 To our knowledge, our series is the first describing locally-acquired cases in Belgium. 4 It is worrying for few reasons. First, the colonization infection ratio was high, attesting the high pathogenic power of these pathogens (and or host frailty). Second, 100% of infected patients died, despite early targeted antimicrobial therapy.

It is well known that genes encoding CPE are usually plasmid-transmitted. The fact that all patients had previous broad-spectrum antibiotic therapy supports this statement. However, the only species found was K.P, all the patients had been staying in the same unit and time proximity was clear. This pleads for a “contact” component of transmission. Hygiene rules, in addition to antibiotic stewardship, are therefore crucial.

### P-24 Risk factors for central line-associated bloodstream infection in pediatric intensive care unit- a monocentric case–control retrospective study

#### Eric Thebault (*speaker*)^1^, Catherine Doit^1^, Michael Levy^1^, Jerome Naudin^1^, Géraldine Poncelet^1^, Camille Ducrocq^1^, Stéphane Bonacorsi ^1^, Stephane Dauger^2^

##### ^1^AP-HP Hôpital Robert Debré, Paris, FRANCE; ^2^Réanimation pédiatrique, hôpital Robert Debré, Paris, FRANCE

###### **Correspondence:** Eric Thebault - eric.thebault.08@gmail.com

*Annals of Intensive Care* 2019, **9(Suppl 1)**:P-24

**Introduction**: Central line-associated bloodstream infections (CLABSI) remain a significant source of morbidity, cost, and mortality in pediatric intensive care unit (PICU). Pediatric international recommendations are extrapolation of adults’guidelines. We sought to determine risk factors (RF) for CLABSI, to describe CLABSI incidence, and to compare evolution of patients with and without central venous catheter (CVC) removal.

**Patients and methods**: Monocentric retrospective case–control study, matched by sex, age, and ICU admission date, in a tertiary 20-bed-PICU from the 01 01 2009 to the 31 12 2017. CLABSI were defined according to the CDC criteria, identified through a prospective data collection of positive hemoculture in the PICU. Premature and patients over 18-years-old on admission were excluded. Control subjects were patients with a CVC without any infection. A list of potential RF was developed by literature review. Comparisons of characteristics between case patients and control subjects were made using the Wilcoxon rank sum test, the Khi-2 test, or the Fisher exact test, as appropriate. Univariate and multivariate conditional logistic regression models were used to identify independent RF for CLABSI. Two-sided P values of less than .05 were considered to be significant.

**Results**: 79 case patients were matched with 158 control subjects. Independent RF for CLASBI were- vein of insertion- jugular (OR = 4.59, IC95% = [1.34–15.74], p = 0.015), and subclavian (OR = 4.97, IC95% = [1.15–21.43], p = 0.031) + congenital metabolic disease (OR = 24.54, IC95% = [2.21–272.34], p = 0.009) + parenteral nutrition (OR = 6.08, IC95% = [2.23–16.56], p = 0.00041) + blood transfusion (OR = 3.30, IC95% = [1.58–6.86], p = 0.0014) + duration of CVC (OR = 1.03, IC95% = [1.005–1.055], p = 0.0188). CVC type, number of lumens, gastrostomy tube, rate of white cells, were not independent RF for CLABSI. 3 case patients went under a guidewire exchange catheterization, and none of the control subjects. The incidence of CLABSI was 4.04 1000 days of CVC [2.74–5.34]. 8 CLABSI were due to extended-spectrum ß-lactamase producing Enterobacteriaceae (10.1%), and 2 were due to methicillin-resistant Staphylococcus aureus (2.5%), all occurring in previously identified carriers.

**Conclusion**: Insertion vein, congenital metabolic disorders, parenteral nutrition, blood transfusion, duration of ICU CVC, were independent RF for CLABSI. These results must be confirmed by a prospective multicenter study.

### P-25 Procalcitonin to reduce exposure to antibiotics during acute chest syndrome in sickle cell disease. PROSTA study

#### Keyvan Razazi (*speaker*)^1^, Ségolène Gendeau^1^, Elise Cuquemelle^1^, Mehdi Khellaf^2^, Constance Guillaud^3^, Bertrand Godeau^4^, Giovanna Melica^5^, Stéphane Moutereau^6^, Camille Gomar^7^, Slim Fourat^7^,Nicolas De Prost^8^, Guillaume Carteaux^8^, Christian Brun Buisson^8^, Pablo Bartolucci^9^, Anoosha Habibi^9^, Armand Mekontso Dessap^8^

##### ^1^CHU Henri Mondor, Créteil, FRANCE; ^2^Service des urgences CHU Henri Mondor, Créteil, FRANCE; ^3^département d’aval des urgences CHU Henri Mondor, Créteil, FRANCE; ^4^service de médecine interne CHU Henri Mondor, Créteil, FRANCE; ^5^service d’immunologie clinique et maladies infectieuses CHU Henri Mondor, Créteil, Créteil; ^6^service de biochimie CHU Henri Mondor, Créteil, FRANCE; ^7^service de bactériologique CHU Henri Mondor, Créteil, FRANCE; ^8^service de réanimation CHU Henri Mondor, Créteil, FRANCE; ^9^Unité des maladies génétiques du globule rouge UMGGR CHU Henri Mondor, Créteil, FRANCE

###### **Correspondence:** Keyvan Razazi - keyvan.razazi@aphp.fr

*Annals of Intensive Care* 2019, **9(Suppl 1)**:P-25

**Introduction**: Acute chest syndrome (ACS) is a major complication of sickle cell disease and the main cause for mortality in adult patients. Current guidelines recommend the use of antibiotics for the treatment of ACS, but bacterial infection is documented in only a minority of cases. The use of procalcitonin (PCT) may allow shortening the exposure to antibiotics during ACS.

**Patients and methods**: Prospective before-after study in a university hospital. Patients received antibiotics according to current French guidelines for ACS. During the control (before) period, clinicians were blinded to PCT concentrations. At the end of this period, these measurements were used to build an algorithm to fasten antibiotic cessation after three days of treatment if bacterial infection was not documented and serial PCT concentrations were < 0.5 μg L. During the intervention (after) period, the PCT algorithm was used, with the final decision to start or stop antibiotics at the discretion of the physician. The primary endpoint was the number of days alive without antibiotics at day-21.

**Results**: The control and intervention periods included 43 and 60 ACS episodes occurring in 41 and 59 patients, respectively. A total of 46 (45%) episodes required ICU admission. A microbiologically documented infection was found in only 7 (7%) episodes. The number of days alive without antibiotics at day-21 was longer during the intervention than the control period (15 [14–18] vs. 13 [13–14] days, p = 0.001). More patients had a short (≤ 3 days) course of antibiotics during the intervention as compared to the control period- 18 (31%) vs 5 (12%), p < 0.01. There was no infection relapse nor pulmonary reinfection. Hospital length of stay was not different between the two groups (control group- 7 [6–11] vs. intervention group- 8.0 [6.0–12.0] days, p = 0.315).

**Conclusion**: A PCT-guided strategy was able to shorten the duration of antibiotic therapy with no apparent adverse outcomes.

### P-26 SVEGFR2- A New Triage biomarker for patient with suspected SEPSIS at Emergency Department admission

#### Thomas Lafon (*speaker*)^1^, Christine Vallejo^1^, Marion Douplat^2^, Franck Verschuren^3^, Saïd Laribi^4^, Thibault Desmettre^5^, Anaïs Colonna^6^, Maxime Maignan^7^, Mustapha Sebbane^8^, Jacques Remize^9^,Olivier Dupeux^10^, Marie-Angélique Cazalis^11^, Laurence Barbier^11^, Bruno François^12^

##### ^1^Inserm CIC 1435, Service des Urgences, CHU Dupuytren, Limoges, FRANCE; ^2^Service des Urgences, Hospices Civils de Lyon, Lyon, FRANCE; ^3^Service des Urgences, Cliniques Universitaires Saint-Luc, Bruxelles, BELGIUM; ^4^Service des Urgences, CHU, Tours, FRANCE; ^5^Service des Urgences, CHU, Besançon, FRANCE; ^6^Service des Urgences, CH, Montauban, FRANCE; ^7^Service des Urgences, CHU Grenoble, La Tronche, FRANCE; ^8^Service des Urgences, CHU, Montpellier, FRANCE; ^9^Service des Urgences, CH, Brive, FRANCE; ^10^Service des Urgences, CH, Angoulême, FRANCE; ^11^Medical Diagnostic Discovery Department MD3, Biomérieux, Marcy-L’étoile, FRANCE; ^12^Inserm CIC 1435, Réanimation Polyvalente, Inserm UMR1092, CHU Dupuytren, Université de Limoges, FRANCE

###### **Correspondence:** Thomas Lafon - thomas.lafon@chu-limoges.fr

*Annals of Intensive Care* 2019, **9(Suppl 1)**:P-26

**Introduction**: An accurate assessment of septic patients at risk for poor clinical outcomes is challenging for emergency physicians. Patients experiencing sepsis can develop organ dysfunctions whose intensity and duration have been linked with deleterious outcomes. As early management of sepsis has been proven successful, accurate triage of these patient is key. In this study, we investigated whether biomarkers could predict the deterioration in patients admitted at ED with a suspected infection and help emergency physicians to better triage manage.

**Patients and methods**: TRIAGE was a prospective, multicentre (14 sites in France and Belgium) observational study. Adult patients admitted in the ED with a suspected infection and at least 2 SIRS criteria were included. Blood samples were collected at 0, 6 and 24 h after admission. Main outcome was subsequent deterioration (death, intensive care admission, increase of SOFA score) within 72 h. This primary endpoint was assessed by an independent adjudication committee of sepsis experts. Biomarkers association with primary endpoint and prognostic performances were assessed on clinical variables (age, sex, Charlson score, SOFA score, qSOFA, lactates). AUC under the ROC curve and their 95% confidence interval were computed using DeLong’s method. Predictive performances were assessed using classification threshold optimized for high sensitivity.

**Results**: sVEGFR2 and sUPAR protein levels were measured in 462 ED patients at 3 time points- H0 (patient’s admission), H6 and H24. Of these 462 patients, 124 (27%) worsened within the 72-hour study period. Compared with other biomarkers, the sVEGFR2 sUPAR protein combination was the most differentially expressed between worsening and non-worsening patients at H0 and H6 (p-value < 0.01, Mann–Whitney test). Interestingly, 233 patients were no considered as sepsis patients at enrolment (SOFA < 2). Of these, 48 (21%) developed organ dysfunction within the 72-hour study period. Again, the combination of sVEGFR2 and sUPAR protein levels was, at inclusion (H0), the best predictor of worsening (AUC = 0.73, sensitivity = 0.92, NPV = 0.95), compared with other biomarkers- CRP (AUC = 0.57), lactates (AUC = 0.48), qSOFA (AUC = 0.54) and PCT (AUC = 0.61). Moreover, this performance was increased at H6 (AUC = 0.79, sensitivity = 0.94, NPV = 0.98).

**Conclusion**: The expression of the sVEGFR2 sUPAR protein significantly predicts the worsening of septic patient within 72 h after their admission to the ED. Such biomarker(s) could enhance early identification of severe patients for an appropriate and rapid management able to decrease risks of poor outcome.

### P-27 TRIAGE Study protocol- Assessment of biomarkers to predict clinical worsening of patients with sepsis admitted in the Emergency Department

#### Thomas Lafon (*speaker*)^1^, Christine Vallejo^1^, Laurence Barbier^2^, Marie-Angélique Cazalis^2^, Thomas Daix^3^, Arnaud Desachy^3^, Valérie Gissot ^4^, Pierre-François Laterre^5^, Karim Tazarourte^6^, Bruno François^7^

##### ^1^Inserm CIC 1435 Service des Urgences, CHU Dupuytren, Limoges, FRANCE; ^2^Medical Diagnostic Discovery Department MD3, Biomérieux, Marcy-L’étoile, FRANCE; ^3^Inserm CIC 1435 Réanimation Polyvalente, CHU Dupuytren, Limoges, FRANCE; ^4^Inserm CIC 1415, CHU Tours, FRANCE; ^5^Service des Soins Intensifs, Cliniques Universitaires Saint-Luc, Bruxelles, BELGIUM; ^6^Service des Urgences, Hospices Civils de Lyon, FRANCE; ^7^Inserm CIC 1435 Réanimation Polyvalente, Inserm UMR1092, CHU Dupuytren, Université de Limoges, FRANCE

###### **Correspondence:** Thomas Lafon - thomas.lafon@chu-limoges.fr

*Annals of Intensive Care* 2019, **9(Suppl 1)**:P-27

**Introduction**: Sepsis is a frequent reason for admission in the Emergency Department (ED) and assessment of septic patients at risk for poor clinical outcomes is challenging for clinicians. Currently, no validated prognostic tool is available. Therefore, identification of patients at high risk of worsening in the ED is key. The TRIAGE objective was to assess the prognostic value of a blood marker panel to predict early clinical worsening of patients admitted in the ED with suspected sepsis.

**Patients and methods**: TRIAGE was a prospective, multicenter (14 sites in France and Belgium) study on biological samples. Adults patients admitted in the ED with suspected or confirmed community-acquired infection and at least 2 SIRS criteria were included. The protocol included 5 clinical and biological time points (H0, H6, H24, H72, D28). Main outcome was subsequent deterioration (defined as any of the following- death, intensive care admission, increase of SOFA score) within 72 h. Patients were divided into 2 groups depending on worsening or not. The evolution criteria were centrally evaluated by an independent adjudication committee of sepsis experts (emergency physicians and intensivists). Accuracy and prognostic performances of biomarkers were assessed. Patients were followed up to day 28 for mortality.

**Results**: The study duration was 3 years, 602 patients were included (wrongly included, n = 38 + secondary excluded, n = 112) so 462 patients were analyzed (mean age 62.6 ± 20.5 y, Charlson score- 3.1 ± 2.6). After adjudication, 124 patients have been classified as worsening and 338 patients without deterioration. At admission there was no difference between the 2 groups on SIRS criteria count, WBC, CRP and lactates (cf table). The centralized analysis is in progress to select the combination of biomarkers with the best prognostic performance.

**Conclusion**: While no vital signs nor biological markers or parameters can predict worsening of patients, the prognostic value of a panel of blood markers in EDs could help identification of septic patient at risk of worsening at time of admission and develop specific management.


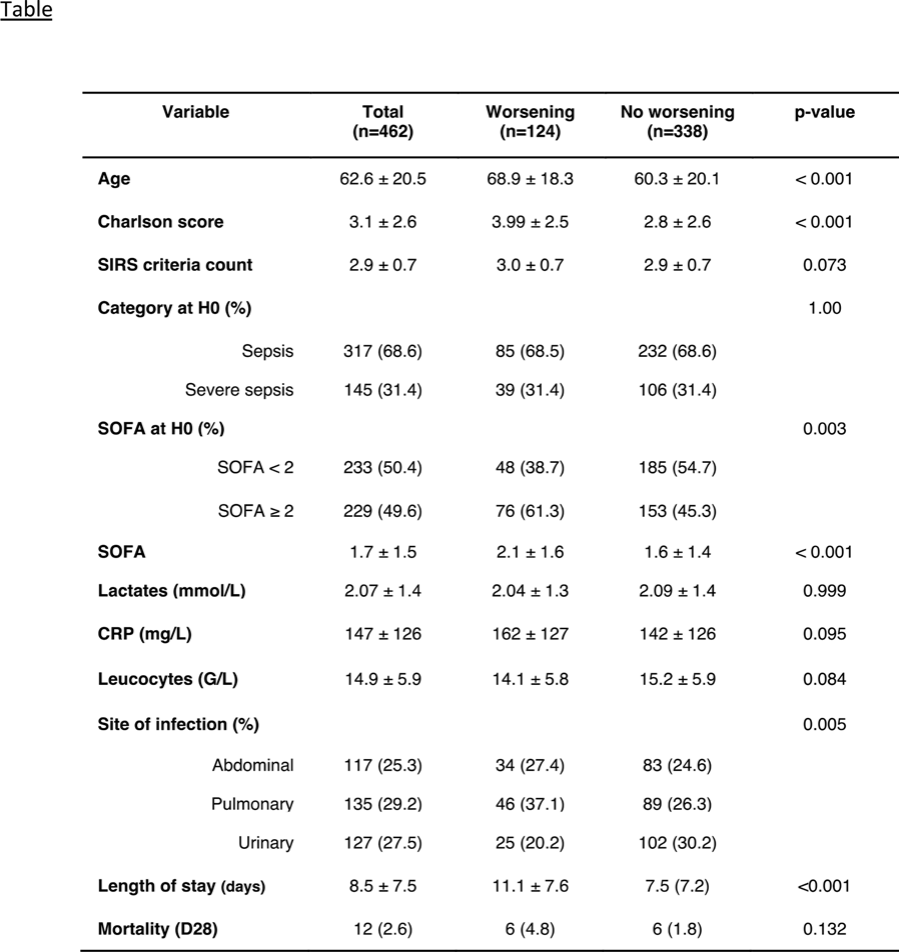


### P-28 The role of biomarkers as predictors of mortality in septic shock

#### Sabrine Bradai (*speaker*), Mariem Dlela, Karama Bouchala, Hela Kallel, OlfaTurki, Mabrouk Bahloul, Mounir Bouaziz

##### Department of Intensive Care, Habib Bourguiba University Hospital, Sfax, TUNISIA

###### **Correspondence:** Sabrine Bradai - Sabrine.bradai2@gmail.com

*Annals of Intensive Care* 2019, **9(Suppl 1)**:P-28

**Introduction**: Septic shock is one of the leading causes of mortality among critically ill patients. Biomarkers offer a tool in facilitating early diagnosis, identifying patients at high risk of complications and monitoring progression of the disease. In this study, we aim to explore the value of procalcitonin (PCT), C-reactive protein (CRP), and cholinesterase activity (SChEA) kinetics, as useful predictors of mortality in patients with septic shock admitted in intensive care unit (ICU).

**Patients and methods**: We conducted a prospective single-blinded study at the ICU of Habib Bourguiba university hospital, Sfax, Tunisia, between January 01, 2017, and December 31, 2017. Were included all patients with 18 years of age, or older, with confirmed septic shock. For all included patients, blood samples of septic biomarkers (PCT, SChEA and CRP) were obtained. Serum was collected at the day of ICU admission (D0), the day of septic shock (D1), then 3 and 5 days after the septic shock development (D3 and D5).

**Results**: During the study period, 60 patients were included. The mean age (± SD) was 47.7 ± 19 years. There were 46 male (74%) and 14 female (26%) patients. Mean SAPSII on ICU admission was 40.7 ± 16 (median- 40) and mean SOFA score on ICU admission was 16 ± 4 (median- 7). Out of the 60 included patients, 37 patients died (The mortality rate was 61%).

On the day when septic shock was settled (D1), there were no differences between the two groups in the mean plasma concentrations, neither in those of SChEA, nor in those of PCT and CRP. However, the comparison of mean plasma concentrations of SChEA, PCT and CRP on D3 and D5, showed a significant difference between survivors and non-survivors. (Table 1).

**Conclusion**: Our study suggests that in a group of critically ill patients with severe septic shock, a fall in procalcitonin and or CRP levels + and or a rise in SChEA were associated with a favorable outcome. Further studies are needed on this subject.


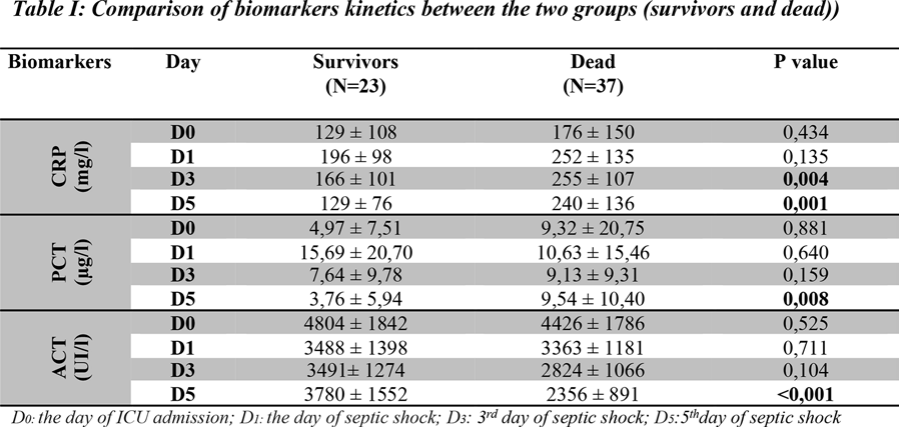


### P-29 Procalcitonin fail to distinguish between bacterial and viral infection in severe AECOPD

#### Cedric Daubin (*speaker*)^1^, François Fournel^1^, Stephane Allouche^1^, Fabrice Thiolliere^2^, Fabrice Daviaud^3^, Michel Ramakers^4^, Andrea Polito^5^, Muriel Fartoukh^6^, Nicolas Terzi^7^, Damien Du Cheyron^1^,Jean-Jacques Parienti^1^

##### ^1^CHU Caen, Caen, FRANCE; ^2^Hospices Civils de Lyon, FRANCE; ^3^Cochin University hospital, Paris, FRANCE; ^4^General hospital, Saint Lo, FRANCE; ^5^Raymond Poincare Hospital, Garches, FRANCE; ^6^Hopital Tenon, Paris, FRANCE; ^7^CHU, Grenoble, FRANCE

###### **Correspondence:** Cedric Daubin - daubin0256@aol.com

*Annals of Intensive Care* 2019, **9(Suppl 1)**:P-29

**Introduction**: Recently in a randomised clinical trial, we failed to demonstrate the non inferiority of a procalcitonin (PCT) guided antibiotic therapy with respect to 3 month mortality among patients with severe acute exacerbation of chronic obstructive pulmonary disease (AECOPD) who needed mechanical ventilation [1]. To explain this result we assessed the capacity of PCT to distinguish between infectious and non infectious causes of severe AECOPD.

**Patients and methods**: The PCT levels of all patients at time of inclusion (H0), 6 h after and at day 1 were measured using a sensitive immunoassay.

**Results**: Regarding all the cohort (n = 302), at any time, the PCT levels were higher in patients with bacterial infection (n = 66) and viral infection (n = 69) than in patients without documented infection (Fig. 1). Interestingly, whether the PCT levels tended to be higher in patients with bacterial infection (n = 33) than in patients with viral infection (n = 37) in the pneumonic group patients, PCT failed to distinguish between bacterial infection (n = 33) and viral infection (n = 32) in non pneumonic group patients.

**Conclusion**: These results underline the interest of PCT as biomarker to distinguish between infectious and non infectious causes of severe AECOPD. However, PCT could fail to distinguish between bacterial and viral infection in this setting.


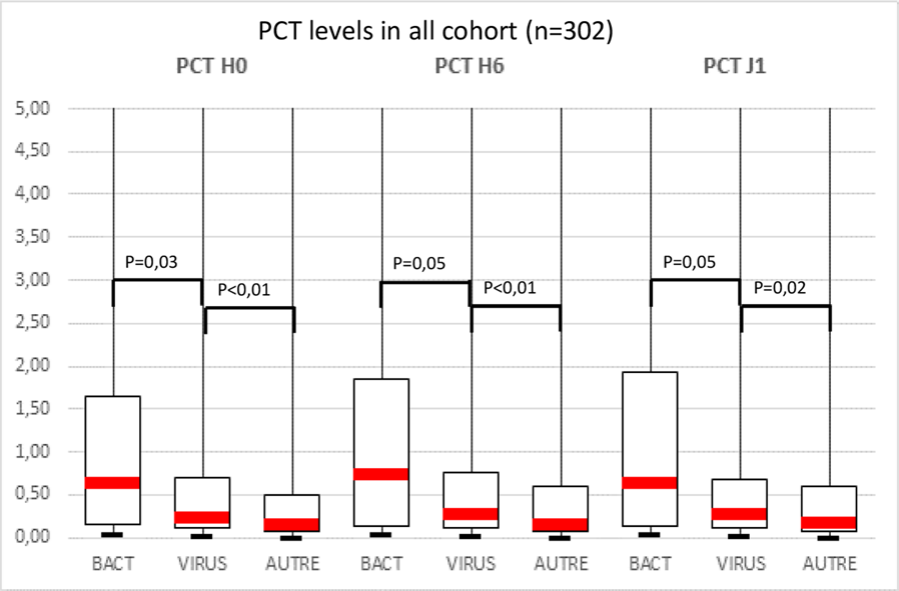


### P-30 Severe eosinopenia as a predictive factor of ICU admission in infected patients admitted in an Emergency Department

#### Jérémy Rosman (*speaker*)^1^, Simon Hainguerlot^2^, Aurélien Cordonnier^3^, Karelle Staffe^3^, Olivier Gallon^4^, Thomas Beuvelet^5^, Xavier Fontaine^2^, Philippe Mateu^1^

##### ^1^Service de Médecine Intensive Réanimation, CH, Charleville-Mézières, FRANCE; ^2^Service d’Accueil des Urgences, CH, Charleville-Mézières, France; ^3^Département de l’Information Médicale, CH, Charleville-Mézières, France; ^4^Service des Maladies Infectieuses, CH, Charleville-Mézières, FRANCE; ^5^Laboratoire de Biologie, Centre Hospitalier de Charleville-Mézières, Charleville-Mézières, FRANCE

###### **Correspondence:** Jérémy Rosman - jrosman@ch-charleville-mezieres.fr

*Annals of Intensive Care* 2019, **9(Suppl 1)**:P-30

**Introduction**: Infectious diseases represent a frequent cause of consultation in the Emergency Department (ED). Biomarkers may help clinicians to assess severity and address them more promptly to the intensive care unit (ICU). Severe eosinopenia may reflect level of systemic inflammation. The aim of our study was to evaluate severe eosinopenia, as defined a value < 0.01 G L (< 10 mm3), in infected patients to predict evolution towards an admission in ICU.

**Patients and methods**: Infected patients admitted in the ED with at least one complete blood count were included. Factors explaining admission in ICU within 7 days following ED admission were evaluated by multivariable logistic regression.

**Results**: Among 15.639 patients screened, 1.643 infected patients were finally included. Nearly half of them had severe eosinopenia at admission in the ED. By multivariable analysis, severe eosinopenia [aOR 2.5 (95% IC 1.2–4.3)], history of chronic renal failure [aOR 2.8 (95% IC 1.4–5.6)], C-reactive protein ≥ 70 mg /L [aOR 2.0 (95% IC 1.4–3.5)] and pulmonary infection [aOR 1.8 (95% IC 1.1–2.9)] were independent factors associated with ICU admission within the following week. All other elements in differential count were eliminated in the model. Severe eosinopenia was also significantly associated with longer length of stay, more bacteremia (17% vs 6%), septic shock within 7 days (2% vs 0.5%), 28-days mortality (5% vs 2%) (p ≤ 0.002 for all outcomes).

**Conclusion**: Severe eosinopenia, a reflect of systemic inflammation, was an independent factor associated with ICU admission within 7 days and was also associated with longer length of stay, bacteremia, septic shock and mortality.


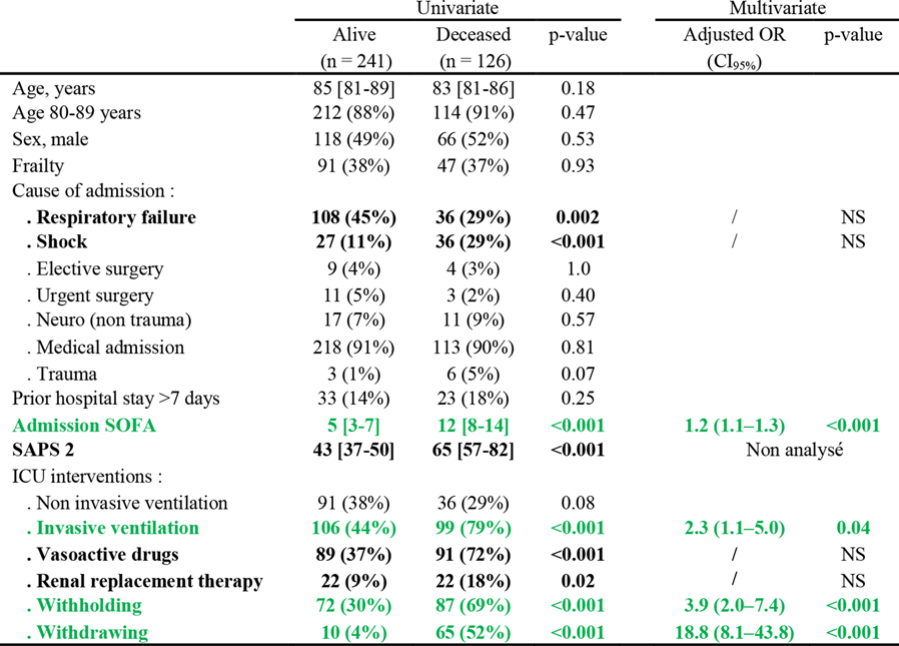



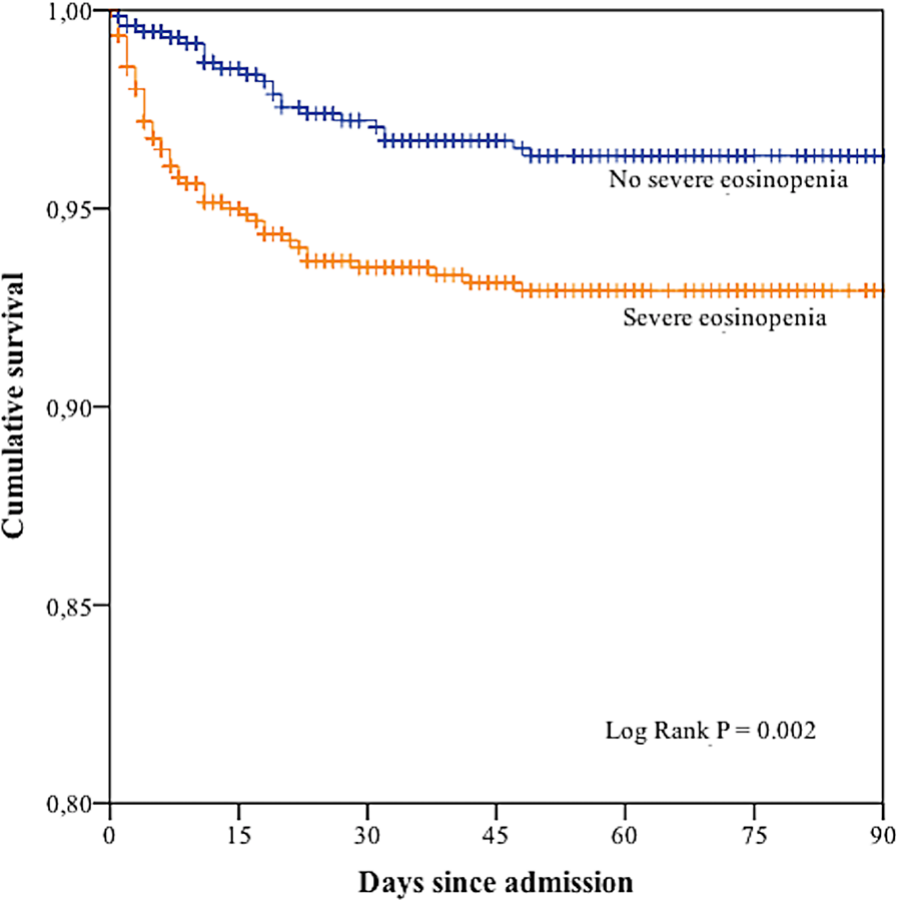


### P-31 Is severe lymphopenia a predictive marker of infection in the Emergency Department ?

#### Thomas Lafon (*speaker*)^1^, Arthur Baisse^2^, Ana Catalina Hernandez Padilla^3^, Thomas Daix^4^, Olivier Barraud^5^, Christine Vallejo^6^, Bruno François ^7^, Philippe Vignon^7^

##### ^1^CHU Dupuytren, Limoges, FRANCE; ^2^Service d’Accueil des Urgences, CHU Dupuytren, Limoges, FRANCE; ^3^Inserm CIC 1435, CHU Dupuytren, Limoges, FRANCE; ^4^Inserm CIC 1435, Réanimation polyvalente, CHU Dupuytren, Limoges, FRANCE; ^5^Inserm UMR 1092, Laboratoire de Bactériologie-Virologie-Hygiène, Université de Limoges, CHU Dupuytren, Limoges, FRANCE; ^6^Inserm CIC 1435, Service d’Accueil des Urgences, CHU Dupuytren, Limoges, FRANCE; ^7^Inserm CIC 1435, Réanimation polyvalente, Inserm UMR 1092, CHU Dupuytren, Université de Limoges, FRANCE

###### **Correspondence:** Thomas Lafon - thomas.lafon@chu-limoges.fr

*Annals of Intensive Care* 2019, **9(Suppl 1)**:P-31

**Introduction**: In the Emergency Department (ED), early and appropriate recognition of infection is crucial to start antibiotic treatment in a timely fashion. Traditional biomarkers remain not specific enough in infected patients. Lymphopenia, known to be associated with sepsis in the ICU, has not yet been evaluated as a potential infection biomarker in the ED. On the contrary, white blood cell (WBC) count is routinely and widely performed as part of admission assessment. We investigated whether isolated or clinically associated severe lymphopenia in ED could be a marker of infection.

**Patients and methods**: We conducted a retrospective single-center study over a 1 year-period. Adult patients admitted in the ED with severe lymphopenia (lymphocyte count < 0.5 G L) were analyzed. Patients with hematological or oncological diseases, HIV infection, hepato-cellular deficiency, immunosuppression and over 85 years old were excluded. Diagnosis of infection was confirmed by an independent adjudication committee. Correlation between lymphopenia and infection was assessed using a univariate analysis and a multivariate logistic regression.

**Results**: From January to December 2017, 953 patients were admitted in ED with severe lymphopenia and 245 were eligible (148 men + mean age 63 ± 19 years). Infection was confirmed in 159 patients (65%) (bacterial- 60%, viral- 30%, other- 10%). Initially, only 61 patients (25%) were referred to ED for suspected infection. Among biological markers, only CRP was different between infected and uninfected groups (109 ± 133 vs 46 ± 62, p < 0.01). The depth of lymphopenia was associated with a higher prevalence of infection, bacteremia, and microbiological identification (Figure). In multivariate analysis, only clinical criteria were independently associated with infection (SIRS criteria- OR = 2.4, p < 0.01 + fever- OR = 3.35, p = 0.02). On the contrary, classical biological biomarkers did not appear associated with infection (WBC count- OR = 0.93, p = 0.07 + CRP- OR = 1.01, p = 0.07).

**Conclusion**: Prevalence of infection is high in patients with severe lymphopenia regardless the reason of admission to the ED and traditional biomarkers results. This easy measurable marker could be used routinely by emergency physicians to assist in the early diagnosis of infection.


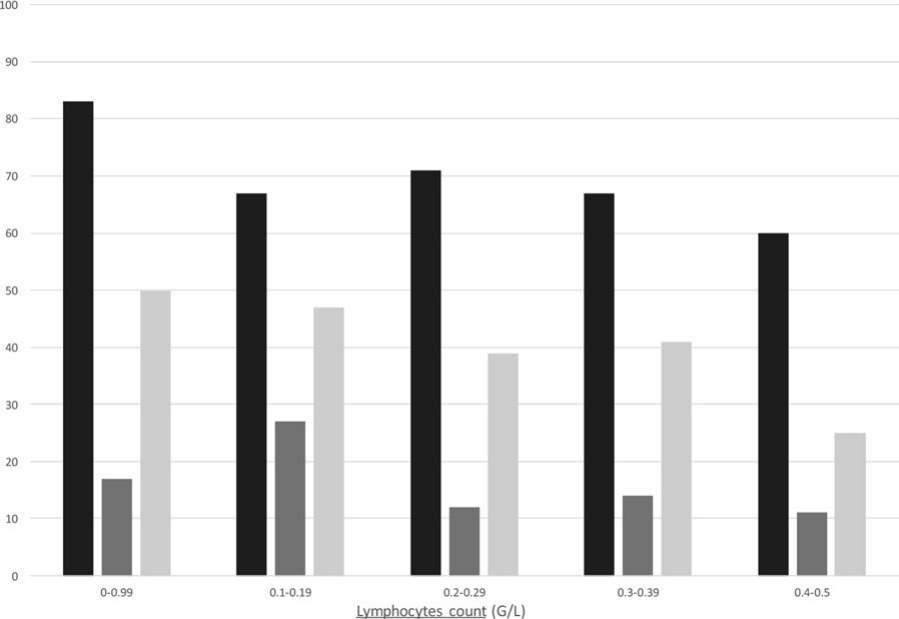


### P-32 Contribution of neutrophil to lymphocyte and platelet to lymphocyte ratios in diagnostic of infection during decompensated cirrhosis

#### Ameni Khaled (*speaker*)^1^, Sana Khedher^2^, Khaoula Ben Ismail^2^, Cyrine Abdennabi^2^, Nasreddine Foudhailin^2^, Mohamed Salem^2^, Radhwan Debbesh^2^

##### ^1^Hopital Mami, Ariana, TUNISIA; ^2^Hepato and Gastroenterology department - Intensive care unit, Charles Nicolle Hospital, Ariana, TUNISIA

###### **Correspondence:** Ameni Khaled - ameni.khaled1988@gmail.com

*Annals of Intensive Care* 2019, **9(Suppl 1)**:P-32

**Introduction**: The prognosis of infection in Acute on chronic liver failure (AoCLF) depends on the rapidity of diagnosis which may be difficult, because of the absence of classical signs as fever. It has been found that Neutrophil–lymphocyte ratio (NLR) and Platelet-lymphocyte ratio (PLR) are regarded as cheap, simple and promising parameters to predict sepsis severity in several diseases. We aim to Evaluate the role of NLR and PLR in the diagnosis of infection in AoCLF.

**Patients and methods**: This was a retrospective follow-up study of 92 cases with the diagnosis of decompensated cirrhosis from January to December 2016.

**Results**: A total of 92 cases of decompensated cirrhosis were enrolled in our study. The mean age was 62 ± 13.4 years. The gender was 0.95. The diagnosis of sepsis was identified in 46% of cases. Multivariate logistic regression analyses showed that there is a significant positive correlation between C-reactive protein (CRP) (p = 0.00), Procalcitonin (p = 0.004), PLR (p = 0.006) and NLR (p = 0.02) with infection. To evaluate the ability of NLR, CRP, Procalcitonin and PLR to predict infection, ROC curves were obtained. Of all the evaluated parameters, NLR showed a strong, statistically significant correlation, a high concordance correlation coefficient.

**Conclusion**: Infection is one of the leading causes of hospitalization of patients with decompensated cirrhosis and is associated with considerable mortality, for which clinicians are seeking useful and easily obtained biomarkers. Our study had shown that Both PLR and NLR were good markers to predict infection in AoCLF.


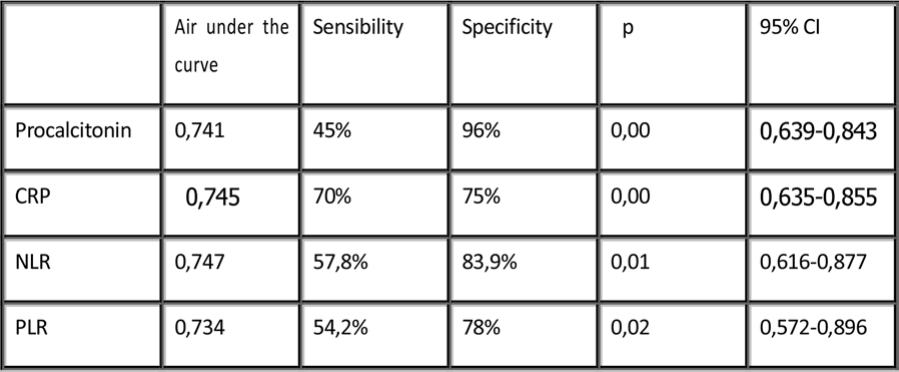


### P-33 Anxiety, depression and post-traumatic stress disorder after intensive care unit discharge- prevalence and predictors

#### Ornella Caruso (*speaker*)

##### CH Cambrai, Lille, FRANCE

###### **Correspondence:** Ornella Caruso - carusornella@gmail.com

*Annals of Intensive Care* 2019, **9(Suppl 1)**:P-33

**Introduction**: Evaluate anxiety, depression and post-traumatic stress disorder prevalence (PTSD) in intensive care unit (ICU) survivors. Determine risk factors of post intensive care unit psychologic comorbidity and evaluate professional practices’ evolution.

**Patients and methods**: The study included adult patients, with length of stay ≥ 48 h in ICU, whom came to the 3-month follow-up’s consultation with the filled form- Post-Traumatic Stress Symptom scale (PTSS-10) and Hospital Anxiety and Depression Scale (HADS), received a month earlier. Potential risk factors of pre, per and post intensive care unit were collected. Prevalences of psychiatric comorbidities were determined by year.

**Results**: Among 120 patients studied, 48.33% (CI 95% (39.12 - 57.63)) had anxiety, 36.67% (CI 95% (28.06 - 45.95)) had depressive symptoms and 8.33% (95% CI (4.07 - 14.79)) had PTSD. Anxiety, depression and PTSD was defined by HADS score ≥ 8 and PTSS-10 > 35. The only predictive factor was age for anxiety (p = 0.001). Any other risk factors of psychiatric comorbidity were no significant. Memory disorder seemed to favor PTSD, as well as sepsis’ presence favors anxiety and non-invasive ventilation favors anxiety and depression. There was no significant change in prevalence due to professional practices’ evolution.

**Conclusion**: Anxiety, depression and PTSD after critical illness are common. The detection of predictors allows an early multidisciplinary management of the patient in order to reduce their prevalence.

### P-34 Clinical and epidemiological aspects of a low income country MICU patients

#### Mohamed Ahmed Boujelben (*speaker*), Hela Kallel, Ines Fathallah, Sahar Habacha, Dora Sakis, Mariem Tobich, Nadia Kouraichi

##### Hopital Régional Yasminette, Ben Arous, TUNISIA

###### **Correspondence:** Mohamed Ahmed Boujelben - m.ahmedboujelben@gmail.com

*Annals of Intensive Care* 2019, **9(Suppl 1)**:P-34

**Introduction**: During the last thirty years, our society has undergone several socio-economic and demographic changes, which implies new challenges in public health and the different aspect of critical care management. The aim was to describe the characteristics and mortality risk factors of patients admitted to a low income country MICU (medical intensive care unit).

**Patients and methods**: A retrospective analysis of chart reviews of all patients admitted to a 6-bed tertiary MICU from October 17th, 2016 to July 17th, 2017. Were collected Patients´ characteristics, evolution and outcomes. Univariate and multivariate regression analyses were performed to identify factors independently associated to mortality.

**Discussion**: 97 patients were admitted during the study period. Their main characteristics were - mean age, 55.9 ± 20.6 years + male gender, 68(70.1%) + mean SAPS II, 41.7 ± 20.4 with a predicted mortality of 28%, median length of stay, 8[4–15.5] days. The common causes for admission were acute respiratory failure (42.2%, n = 41), from which 42.8%(n = 18) were on chronic one + and multi-organ dysfunction (15.4%, n = 15). Upon admission the most occurring diagnoses were - community acquired pneumonia, 15(15.5%) + septic shock, 11(11.3%) and voluntary poisoning, 11(11.3%). 82.5%(n = 80) of the patients were admitted from our local emergency room. The rate of acute renal failure was 45.5%(n = 44). 6(6.2%) patients had chronic kidney disease at admission and 9(9.2%) patients required renal replacement therapy. Non-invasive ventilation (NIV) and invasive mechanical ventilation (MV) were indicated for 37.1%(n = 36) and 50.5% (n = 49) of the patients respectively. The median duration of NIV and MV were 3.5 days [2–7] and of 5 days [3–17]. 9(18.4%) patients were tracheostomized due to difficult weaning. The ICU acquired complications are detailed in Table 1. The mortality rate was 28.9%(n = 28). Multivariate analysis identified two independent risk factors for mortality- SAPSII > = 55.5 (OR = 8.03, CI95[2.52 + 25.6], p < 10^−3^) and acute renal failure (OR = 6.13, CI95[1.84 + 20.37], p = 0.03).

**Conclusion**: This study described the trends of MICU admissions in our district. The high incidence of ICU acquired complications remains a major problem in Tunisia and requires the implementation of several reforms. The independent risk factors for mortality were SAPSII and acute renal failure at admission.

### P-35 Critically ill elderly patients- what about long term caregiver’s burden?

#### Bertrand Guidet (*speaker*)^1^, Hélène Vallet^2^, Ariane Boumendil^1^, Laura Moïsi^2^, Caroline Thomas^2^

##### ^1^Department of critical care, Saint Antoine hospital, Paris, FRANCE; ^2^Department of geriatrics, Saint Antoine hospital, Paris, FRANCE

###### **Correspondence:** Bertrand Guidet - bertrand.guidet@aphp.fr

*Annals of Intensive Care* 2019, **9(Suppl 1)**:P-35

**Introduction**: In intensive care unit, the elderly patient’s admission is growing last years. The mortality rate is high and the loss of autonomy is common among survivors. However, data regarding the impact of critical illness on caregiver’s are lacking. Objectives of this study were to evaluate the caregiver’s burden in a critically ill elderly population and to assess factors associated with high burden level.

**Patients and methods**: All patients were included in ICE-CUB 2 clinical trial (clinical trial gouv NCT 01508819). Inclusion criteria were an age ≥ 75, at least on critical condition, a preserved functional status defined as an Activities of Daily Living (ADL) score ≥ 4, a preserved nutritional status and the absence of known active cancer. Patients, for which the Zarit Burden Interview (ZBI) at 6 months was available, were selected. The primary endpoint was a ZBI ≥ 21 (mild to severe burden) at 6 months.

**Results**: One hundred ninety one patients (median age 86 [81–89] years) were included. The median caregiver’s ZBI at 6 months was 13 [5–27] and ≥ 21 for 71 patients (37%). In multivariate analysis, factors significantly associated with moderate to severe burden were the 6-month ADL decrease (OR- 1.3 [1–1.68], p = 0.049) and the mental component of the quality of life (QoL) score (SF- 12) at 6 months (OR- 0.94 [0.89–0.98], p = 0.0009). In contrast, age, ICU admission and length of hospital stay were not associated with moderate to severe load (OR 0.84 [0.41–1.73], p = 0.64, OR 0.99 [0.93–1.06], p = 0.85 and OR- 1.02 [0.99–1.06], p = 0.12 respectively).

**Conclusion**: Functional autonomy and QoL at 6 months are associated with mild to severe caregiver’s burden, contrary to age, ICU admission and length of hospital stay. Advanced age shouldn’t be an obstacle to the care of critically ill elderly patient. However, improving their long term autonomy and QoL remains important, that means higher implication of geriatricians in their medical care.

### P-36 Post Intensive Care Syndrome - an observational study

#### Julien Le Marec (*speaker*), Youenn Jouan, Maud Vaudour, Stephan Ehrmann, Charlotte Salmon

##### Médecine Intensive Réanimation, CHRU de Tours, Tours, FRANCE

###### **Correspondence:** Julien Le Marec - julienlemarec@msn.com

*Annals of Intensive Care* 2019, **9(Suppl 1)**:P-36

**Introduction**: «Post-intensive care syndrome» is the sum of all the cognitive, psychiatric and physical sequelae after a hospitalization in intensive care unit. These frequent sequelae can have a serious impact on the quality of life, autonomy, morbidity and mortality of such patients, and can last for several years after the initial hospitalization. Although it is not consensual, intensive care unit teams more and more frequently schedule visits to assess the physical and mental status of these patients after their discharge of the ward. The clinical interest of such a practice has not been evidenced yet.

**Patients and methods**: Since 2015, a follow-up visit after intensive care unit discharge has been set in the Médecine Intensive – Réanimation ward of Tours University Hospital Centre, in order to detect the potential occurrence of a post-intensive care syndrome in our patients. Patients admitted for acute respiratory distress syndrome and or septic shock were eligible. We conducted a descriptive study of this cohort to assess their evolution. Patients were informed about the study and could decline participation.

**Results**: Thirty-eight patients (on 41 summoned) were reevaluated at a follow-up visit 1 month after discharge. Among them, 16 (39%) had been admitted to ICU for an acute respiratory distress syndrome, 2 (5%) for a septic shock, 3 (7%) for both acute respiratory distress syndrome and septic shock, and 14 (34%) for another diagnosis, such as acute respiratory failure or severe intoxication. Thirteen patients (34%) were still hospitalized in rehabilitation on the follow-up visit day. Among the remaining 25 patient, 10 (40%) had not consulted their general practitioner yet. Fourteen patients (37%) presented with a cognitive alteration, 10 patients (26%) had psychiatric troubles and 32 patients (84%) had physical sequelae (respiratory, neurological….). One patient out of two had sequelae in at least 2 of the 3 domains of post intensive care syndrome. Only 1 patient (3%) had no sequelae at all (Figure 1). Fifteen patients were reevaluated 3 months after discharge, showing a global improvement in their status but with persistent deficiencies. Overall, 84% were referred to a specialist and or had a modification in their treatment after the follow-up visit.

**Conclusion**: Our observational study confirmed the frailty of the patients discharged of intensive care unit, with 97% of our patients presenting with troubles belonging to the field of post intensive care syndrome + this highlights the potential interest of an early reevaluation.


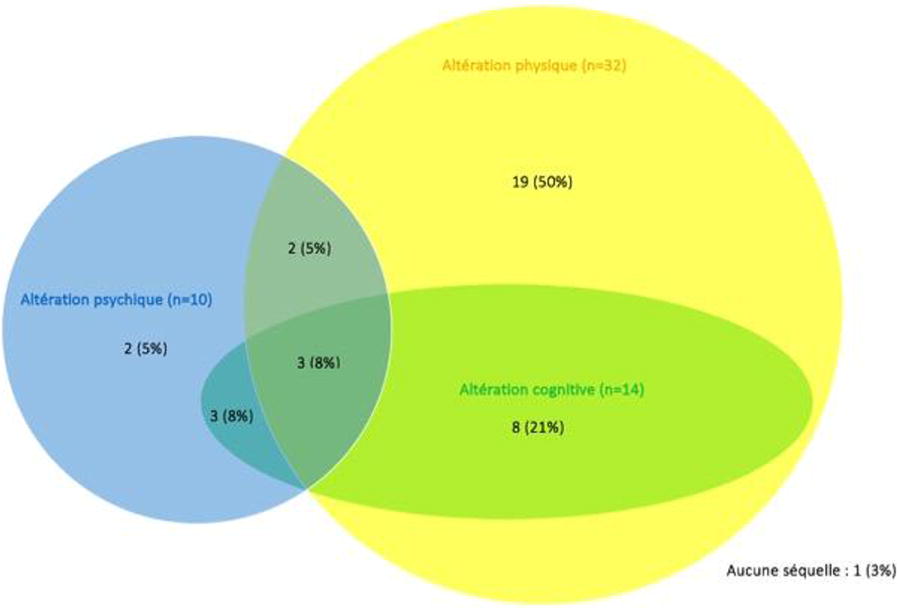


### P-37 Predictive factors of prolonged length of stay of icu elderly patients

#### Dhouha Ben Braiek (*speaker*)^1^, Hend Zorgati^2^, Rafla Ben Dabebiss^2^, Saiid Kortli^2^, Mohamed Ben Rejeb^4^, Houssem Hmouda^5^

##### ^1^CHU Farhat Hached Sousse, Sousse, TUNISIA; ^2^Sahloul University Hospital, Medical Intensive Care Unit., Sousse, TUNISIA; ^4^Sahloul University Hospital, Department of Prevention and Care Safety., Sousse, TUNISIA; ^5^Sahloul University Hospital, Medical Intensive Care Unit., Sousse, TUNISIA

###### **Correspondence:** Dhouha Ben Braiek - bbraiek_dhouha@hotmail.com

*Annals of Intensive Care* 2019, **9(Suppl 1)**:P-37

**Introduction**: Prolonged LOS (length of stay) of ICU patients, especially elderly is a well-defined problem, associated with increased health care costs, resource utilization, delayed ICU admissions from the emergency departments, and may have an impact on morbidity and mortality. Prediction of ICU LOS may contribute to more efficient resources’ allocation and better planning of care. The aim was to determine the incidence and predictive factors of prolonged ICU-LOS in elderly patients admitted to a tunisian medical ICU.

**Patients and methods**: It is a retrospective analytic study conducted in a 4 bedded MICU of a university hospital during a-4 year period including elderly patients (more than 65 years old). ICU stay was considered prolonged when exceeded 15 days. Variables found to be statistically significant in univariate analysis were included into a multivariate regression model to identify factors independently associated with prolonged ICU stay.

**Results**: During the review period, 97 patients were included. Patients’ characteristics were - mean age, 75 ± 7 + mean SAPSII, 40 ± 11 + mean APACHE II, 22 ± 10 + mean SOFA, 7 ± 4 + respiratory disorder on admission, 76(78.4%) + invasive mechanical ventilation, 51(52.6%) and vasopressors use, 56(57.7%). The median length of ICU stay was 8[5–14] days. Twenty-three patients (23.7%) had prolonged ICU stay. Univariate analysis identified several factors associated with prolonged ICU stay- past history of chronic respiratory failure (CRF) (p = 0.026), admission for exacerbation of CRF (p = 0.026), intubation before ICU admission (p = 0.038), hyponatremia at admission (p = 0.019), invasive mechanical ventilation (p = 0.05), sedation (p = 0.002), nosocomial infections (p = 0.003), shock (p = 0.035), ICU-acquired weakness (p = 10^−3^), dyskalemia (p = 0.026), anemia (p = 0.011). In multivariate analysis, three predictors were independently associated with prolonged ICU stay- exacerbation of chronic respiratory failure (OR 4.85, 95%CI [1.44–16.35], p = 0.011), hyponatremia at admission (OR 5.44, 95%CI [1.70–17.43], p = 0.008) and nosocomial infections (OR 4.72, 95%CI [1.51–14.77], p = 0.004).

**Conclusion**: Exacerbation of chronic respiratory failure, hyponatremia on admission and nosocomial infections were the only independent predictors of prolonged ICU stay. Further research is needed for better identification of patients with prolonged ICU stay to ensure optimal use of ICU resources.

### P-38 Trends in use of radiological procedures in medical ICU

#### Emna Ennouri (*speaker*), Ines Fathallah, Asma Mehdi, Sahar Habacha, Hayfa Fazzeni, Eya Seghir, Ghada Sbouii , Khaoula Ben Ismail, Améni Sghaier, Nadia Kouraichi

##### BEN AROUS Regional hospital, Sfax, TUNISIA

###### **Correspondence:** Emna Ennouri - m.na.ennouri@gmail.com

*Annals of Intensive Care* 2019, **9(Suppl 1)**:P-38

**Introduction**: The place of chest film radiography in management of critical care patients is well known. However since advances in imaging technology in last decades, many other tools have been added to the diagnostic arsenal. Few studies investigate the use of other radiological procedures in intensive care unit (ICU). We aimed to determine use frequency, conditions of realization, diagnostic and therapeutic impact, specificity and costs of different radiological procedures in the ICU.

**Patients and methods**: Retrospective study conducted in a medical ICU. All consecutive radiological procedure realized between July 2017 and July 2018 were included data were collected from medical files.

**Results**: One hundred five radiological investigations were realized. Among which 41(41%) Computed Tomography-scan (CT scan), five (5%) Magnetic resonance imaging (MRI) and 57 (54%) echography which includes 21 trans-thoracic echography (TTE), 2 trans-esophageal echography (TEO), 28 abdominal echography and 6 lower limb venous sonography. Forty one (39%) were realized in patients’ bed, 55(52%) were realized in our hospital and 9 (10%) required medical transportation to others centers. Sixty-one (58%) examinations were realized to patient under mechanical ventilation, 69(66%) to patient under sedation and 74(70.5%) to patient under vasopressors. Radiological procedures was compromised in 2%, and complications after realization occurred in 4% (n = 4). Suspected diagnosis was confirmed in 30% (n = 34) of exams. Five (5%) exams was inconclusive. CT scan allowed to confirm 16(37%) suspected diagnosis and echography allowed to confirm 17 (30%). Forty-five percent of suspected pulmonary embolism (PE) were confirmed by pulmonary computed tomography angiography. Forty –four (42%) radiological examinations has a direct therapeutic impact. Mean cost of a radiological exam was 44.7 euro with a global cost of 5004.1 euro.

**Conclusion**: The contribution of imaging in diagnosis and managements of ICU patients is considerable given an important therapeutic impact. Examinations conditions shouldn’t dissuade clinicians for the realization of radiological investigations given the low rate of incidents (2%) and complications (4%).

### P-39 Characteristics and outcomes of elderly patients admitted to a Tunisian intensive care unit (ICU)

#### Hend Zorgati (*speaker*), Imen Ben Saida, Said Kortli, Nesrine Fraj, Mohamed Ahmed Boujelben, Wafa Zarrougui, Khaoula Meddeb, Mohamed Boussarsar

##### Farhat Hached University Hospital, Medical Intensive Care Unit, Sousse, TUNISIA

###### **Correspondence:** Hend Zorgati - zorgati.hend@yahoo.fr

*Annals of Intensive Care* 2019, **9(Suppl 1)**:P-39

**Introduction**: The aging of the population has increased the demand for critical care resources for elderly worldwide. There are few data on the characteristics and outcomes of those patients in ICU. The aim was to assess characteristics and to identify predictors of ICU mortality in elderly patients.

**Patients and methods**: A retrospective study conducted in a medical ICU between January 2016 and December 2017 and included patients aged more than 65 years. Medical patients’ records were reviewed to compile demographic characteristics, severity and diagnosis at admission, management and outcomes. Univariate and multivariate analysis were used to identify predictors of ICU mortality.

**Results**: During the study period, 486 patients were admitted to ICU. 178 (36.6%) were aged more than 65 years. Patients’ characteristics were - median age, 74 years [68–79] + male, 109(61.2%) + median Charlson score, 4 [4–5] + Knauss C or D, 129(72.5%) + MacCabe ≥ 2, 85(47.8%) + median SAPSII, 34[28–44] + median APACHE, 16 [11.75–20] + median SOFA, 4[3–7] + invasive mechanical ventilation (IMV), 80(44.9%) with a median duration at 8 days [3.25–19.75] + vasopressors use, 65(36.5%) + renal replacement therapy, 9(5.1%). The main reasons for admission were - respiratory disorders, 141(79.2%) + shock, 24(13.5%) + neurological disorders, 7(3.9%) and miscellaneous, 6(3.4%). The median length of stay was 8[5–17] days. Mortality rate was 47.2%. The univariate analysis identified respectively for deaths and survivals - Apache II (17.6 ± 6.8 vs 14.9 ± 5.9, p = 0.006) + SOFA (5.89 ± 3.4 vs 3.98 ± 2.3, p = 0.000) + Shock on admission (47.6% vs 26.6%, p = 0.004) + IMV use on admission (42.9% vs 20.2%, p = 0.01) + acute kidney injury (85.7% vs 37.2%, p = 0.00) + Arrythmia (27.4% vs 6.4%, p = 0.000) and nosocomial infection (66.7% vs 12.8%, p = 0.000). Multivariate regression model identified the following factors as independently associated to mortality - nosocomial infection (OR 7.78, 95%CI [3.34-18.09], p = 0.000), acute kidney injury (OR 12.18, 95%CI, [4.5-32.8], p = 0.000) and IMV use on admission (OR 5.95, 95%CI [2.39–14.8], p = 0.000).

**Conclusion**: IMV use on admission, nosocomial infection and acute kidney injury were independently associated to fatal outcome in elderly critical ill patients.

### P-40 How and why do intensivists finance their congresses ?

#### Michael Thy (*speaker*)^1^, Salam Abbara^1^, Redwan Maatoug^2^, Nathan Peiffer-Smadja^3^

##### ^1^Hôpital Necker, Paris, FRANCE; ^2^AP-HP, Paris, FRANCE; ^3^Hôpital Bichat, Paris, FRANCE

###### **Correspondence:** Michael Thy - michael245thy@gmail.com

*Annals of Intensive Care* 2019, **9(Suppl 1)**:P-40

**Introduction**: Congresses are a place of exchange and update of medical knowledge. However, their cost is not negligible and their profitability on the training of participants remains unclear and not studied. The purpose of this study was to identify how and why young intensivits are funding their participation in congresses.

**Patients and methods**: A self-questionnaire in French has been sent to young doctors of any specialty through mailing lists to the interns. People are surveyed about their characteristics, their attendance at a conference, the funding of staff and the total of these and the contribution. The relative data was anonymous. The questionnaire was registered at the CNIL.

**Results**: In total, from August 2017 to November 2017, 1759 people including 185 (10.5%) intensivists responded to a questionnaire, of which 156 (84.3%) internal with an equivalent proportion of each year of internship and 29 (15.7%)) young seniors (clinical leaders and specialist assistants). The last congresses that hosted intensivists were in France for 93.5% of them. The participation of each participant in conferences (75.1% or 139), 15.1% (n = 28) received an invitation, 8.1% (n = 15) received funding and 3.2% (n = 6) had a scholarship. The average amount of personal financing amounted to € 256.7 (SD = 190.3) for an average total amount of € 332.5 (SD = 256.9) (Figure 1). The reasons for their participation were for a simple presence for 77.9% (n = 141) of them and for a presentation at the congresses for 22.1% (n = 40) of them. 90.8% (n = 168) of participants thought that the main interest of conventions was 85.4% (n = 158) thought the price to pay had already been a barrier.

**Conclusion**: Intensivists - self-financed to a significant participation for their participation in congresses while this is organized in a training objective. Better funding opportunities and promotion of fellowships for congresses about intensive care.

### P-41 Midline catheter- an alternative to central venous catheter?

#### Naïke Bigé (*speaker*)^1^, Shobanya Selvarathnam^2^, Jean-Rémi Lavillegrand^1^, Guillaume Dumas^1^, Constance Chateauvieux^2^, Jean-Luc Baudel^1^, Bertrand Guidet ^1^, Hafid Ait-Oufella^1^, Maury Eric^1^

##### ^1^Médecine Intensive Réanimation, AP-HP, Hôpital Saint-Antoine, Paris, FRANCE; ^2^Pharmacie, AP-HP, Hôpital Saint-Antoine, Paris, FRANCE

###### **Correspondence:** Naïke Bigé - naikebige@gmail.com

*Annals of Intensive Care* 2019, **9(Suppl 1)**:P-41

**Introduction**: A midline catheter (MC) is a peripheral venous catheter inserted in a deep-arm vein with extended lifespan up to 30 days. MC could represent an alternative to central venous catheter for patients with poor venous access and or requiring prolonged intravenous treatment such as antibiotics. We seek to determine. The objective of this study was to determine dwell time and safety of MC.

**Patients and methods**: We performed a prospective observational study including consecutive MC insertions in an academic hospital between March and September 2018. Fully integrated PowerGlide ProTM (Bard) device was used. All MCs were inserted using a sterile technique by senior intensivists with experience of ultrasound-guided catheter placement. Before MCs utilization period, nurses and physicians received a training for MCs management. A MCs team (1 pharmacy resident and 1 senior intensivist) followed patients from MC insertion to removal. Ultrasound examination was performed once weekly. MC tips were systematically cultured at removal.

**Results**: Fifty-seven MCs were inserted in 50 patients (men 58%, age 58 years [range 22–96]) representing a total of 691 MC-days. A median of 1 [range 1- 5] catheter was used per insertion. Indications for MC placement were not exclusively- extended antibiotherapy (n = 29), poor venous access (n = 46), iterative blood sampling (n = 12), hydration (n = 11). Median dwell time was 11 days [range -26]. Twenty-nine (47%) MCs were removed without complication. Four patients were lost to follow-up. Twenty-three (40%) MCs were retrieved because of the following complications- accidental removal (n = 4), suspected infection (n = 7) of whom 5 were confirmed by a positive tip-culture, MC dysfunction without vein thrombosis (n = 2), vein thrombosis (n = 10) responsible for MC dysfunction (n = 6) and clinical symptoms (n = 4). No MC-related bloodstream infection (BSI) occurred. The overall incidence of MC-related complications was 33.3 1000 MC-days.

**Discussion**: MC-infection could have been overestimated because 4 of the 5 observed infections occurred in the same patients in whom 4 MC were inserted. Insufficient diameter of the vein (basilic or brachial) in which MC tip was located could be responsible for high rate of vein thrombosis.

**Conclusion**: Despite nurses training and close monitoring of patients, we observed a shorter dwell time than expected and a high rate of MC-associated thrombosis. These results question definite utilization of the MC device we tested. However, longer device with axillary tip could be safer and more durable.

### P-42 Variation of fluid balance as indicator of early clinical outcome in burn patients

#### Achref Laajili (*speaker*)^1^, Alaa Zammit^1^, Hana Fraj^1^, Imen Rahmeni^1^, Sarra Ben Zarrouk^1^, Nejla Ben Slimene^1^, Mehdi Somai^1^, Bahija Gasri^1^, Lotfi Rebai^2^, Amel Mokline^1^,Amen Allah Messadi^1^

##### ^1^Intensive Burn Care Department, Tunis, TUNISIA; ^2^Department of Anesthesiology, Tunis, TUNISIA

###### **Correspondence:** Achref Laajili - laajili@gmail.com

*Annals of Intensive Care* 2019, **9(Suppl 1)**:P-42

**Introduction**: Early and adequate fluid rescuscitation in burns contributes to improve outcomes of severly burned patients, leading to less tissue oedema fluid accumulation and reduces both organ and system dysfunction. The aim of our study was to evaluate the impact of this approach in early burn outcome.

**Patients and methods**: A pospective study was conducted in Intensive Burn Care Department of Tunisia during 5 months (January 2017 and May 2017). Were included patients aged > 18 years, with a total body surface area > 20%, admitted within 24H post burn injury and hospitalized for a duration > 72 h. Patients were resuscitated according to Parkland Formula. Goals of ressuscitation were- Mean arterial pressure (MAP) ≥ 65 mm Hg and Hourly urine output (HUO) ≥ 0.5 ml/kg/h. Body weight was measured at admission and daily using a bed scale. Early clinical outcome in burn patients designed the first week post burninjury.

**Results**: During the period study, 141 patients were admitted in the burn ICU. 34 patients were included. The mean age was 38 ± 15 years with a ratio sex of 4.7. The average TBSA (total body surface area) was 44 ± 20%. Early ICU mortality was 26.5% (n = 9). Patients were assigned into 2 groups-G1- survivors (n = 25) and G2- non survivors (n = 9). Comparative study of 2 groups was as follows (Table 1)- Table 1- Comparative study of 2 groups.

**Conclusion**: Optimal initial fluid resuscitation in burn patients is associated with lower fluids overload during the first 48 h and improves early outcome with a lower early mortality.


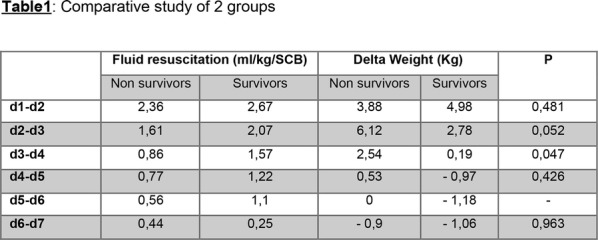


### P-43 Norepinephrine synergistically increases the efficacy of volume expansion on venous return in septic shock

#### Imane Adda (*speaker*), Christopher Lai, Jean-Louis Teboul, Laurent Guerin, Francesco Gavelli, Christian Richard, Xavier Monnet

##### Hôpitaux universitaires Paris-Sud, Hôpital de Bicêtre, APHP, service de médecine intensive-réanimation, Le Kremlin-Bicêtre, FRANCE

###### **Correspondence:** Imane Adda - imane.adda.univ@gmail.com

*Annals of Intensive Care* 2019, **9(Suppl 1)**:P-43

**Introduction**: Through reduction in venous capacitance, norepinephrine increases the mean systemic pressure (Pms) and increases cardiac preload. This effect may be added to the ones of fluids when both are administered in septic shock. Nevertheless, it could be imagined that norepinephrine potentiates in a synergic way the efficacy of volume expansion on venous return by reducing venous capacitance, reducing the distribution volume of fluids and enhancing the induced increase in stressed blood volume. The purpose of this study was to test if the increase in Psm induced by a preload challenge were enhanced by norepinephrine (NE).

**Patients and methods**: This prospective and comparative study had included 25 septic shock adults. To reversibly reproduce a volume expansion and preload increase at different doses of norepinephrine, we mimicked fluid infusion through a passive leg raising (PLR). In patients in which the decrease of NE was planned, we estimated Psm (heart–lung interactions method, using end-inspiratory and end-expiratory occlusions) at baseline and during a PLR test (PLRHigh). The dose of NE was then decreased and Psm was estimated again before and during a second PLR (PLRLow).

**Results**: NE dose decreased from 0.58[0.33–1.1] to 0.44[0.24–0.94] µg kg min (p < 0.0001). The increase in Psm induced by PLRHigh (ΔPsmHigh) at the highest dose of NE was significantly greater than the ΔPsm induced by PLRLow (37[15–48] % vs 12[7.0–32] %, p < 0.001). The increase in cardiac index induced by PLRLow was significantly greater than that induced by PLRHigh (p = 0.0001). ΔPsmHigh - ΔPsmLow was moderately correlated with the diastolic arterial pressure at BaselineHigh (p = 0.014, r = 0.50) and with the NE-induced change in mean arterial pressure (p = 0.0044, r = 0.57).

**Conclusion**: Norepinephrine enhances the increase in Psm induced by a PLR, which mimics a fluid infusion. This suggests that it may potentiate the effects of fluid in a synergetic way in septic shock patients. This may decrease the amount of administered fluids and contribute to decrease the cumulative fluid balance.

### P-44 Transthoracic echocardiography cannot reliably estimate or track changes in central venous pressure in critically-ill patients

#### Mathieu Jozwiak (*speaker*), Alice Boilève, Jean-Louis Teboul, Christian Richard, Xavier Monnet

##### CHU Bicêtre, Le Kremlin-Bicêtre, FRANCE

###### **Correspondence:** Mathieu Jozwiak - mathieu.jozwiak@aphp.fr

*Annals of Intensive Care* 2019, **9(Suppl 1)**:P-44

**Introduction**: As a static marker of cardiac preload, the central venous pressure (CVP) remains a helpful variable at the bedside to guide the hemodynamic management of critically-ill patients. Since transthoracic echocardiography (TTE) is currently the first-line tool for evaluating the hemodynamic conditions in critically-ill patients, we aimed at assessing the ability of TTE to estimate and to track changes in CVP.

**Patients and methods**: In 31 patients (25 mechanically ventilated and four with atrial fibrillation), concomitant CVP measurement (internal jugular catheter) and TTE examination were performed before and after a passive leg raising test or the infusion of 500-mL of saline. We calculated the tricuspid E A waves ratio, the tricuspid E e’ waves ratio, the right ventricular isovolumic time (IVRT), the E IVRT ratio, the S D waves ratio of the supra-hepatic vein flow, the VTI-systolic filling fraction and the peak velocities-systolic filling fraction of the supra-hepatic vein flow, the end-expiratory diameter of the inferior vena cava (IVC) and the respiratory variations of the IVC diameter. For the statistical analysis, we considered that a CVP threshold value of 10 mmHg was clinically relevant.

**Results**: Changes in CVP were induced by a passive leg raising test in 10 patients and by fluid administration in 21 patients. After pooling all values, only the end-expiratory IVC diameter and the respiratory variations of the IVC diameter were correlated to CVP (r = 0.40 and r = -0.26, respectively, p < 0.05 for both). At baseline, an end-expiratory IVC diameter = <14 mm predicted a CVP = or < 10 mmHg with a specificity of 100% (95%CI- 81–100%) but with a sensitivity of 29% (95%CI- 8–58%). An end-expiratory IVC diameter > 26 mm predicted a CVP > 10 mmHg with a specificity of 100% (95%CI- 77–100%) but with a sensitivity of 0% (95%CI- 0–20%). At baseline, the respiratory variations of the IVC diameter could predict neither a CVP = or < 10 mmHg nor a CVP > 10 mmHg (AUC = 0.683, p = 0.08). Only relative changes in the S D ratio and in VTI-systolic filling fraction correlated with relative changes in CVP (r = 0.44 and r = 0.43, respectively, p < 0.05). The concordance rate between changes in S D ratio and CVP was 48% and the concordance rate between changes in VTI-systolic filling fraction and CVP was 55%.

**Conclusion**: In critically-ill patients, TTE cannot reliably estimate CVP or track changes in CVP induced by changes in cardiac preload. Nevertheless, an end-expiratory IVC diameter = ou < 14 mm or > 26 mm can detect a CVP = ou < 10 mmHg or > 10 mmHg respectively with a specificity of 100%.

### P-45 Large arteries in septic shock - a feasibility study for using Speckle Tracking Echocardiography to examine elastance in large arteries

#### Sebastian Knudsen (*speaker*)^1^, Sam Orde^2^, Yoann Zerbib^3^, Elie Zogheib^3^, Julien Maizel^3^, Michel Slama^3^

##### ^1^Sir Charles Gairdner Hospital, Perth, AUSTRALIA; ^2^Intensive Care Unit, Nepean Hospital, Sydney, AUSTRALIA; ^3^CHU Amiens, Amiens, FRANCE

###### **Correspondence:** Sebastian Knudsen - sknudsen@doctors.org.uk

*Annals of Intensive Care* 2019, **9(Suppl 1)**:P-45

**Introduction**: Arterial elastance (Ea) and ventricular end systolic elastance (Ees) have been shown to be linked in a way that may optimize efficiency for converting left ventricular power into forward flow. This relationship may be disrupted in sepsis. We devised a pilot study to investigate the feasibility of using Speckle Tracking Echocardiography (STE) to examine Ea as a surrogate for conventional equations based on blood pressure and stroke volumes in ICU patients. We then chose a group of septic patients and a group of non-septic patients in an attempt to demonstrate a “de-coupling” of Ea and Ees when using conventional equations, and when using STE.

**Patients and methods**: 19 septic and 23 non-septic patients underwent echocardiographic and carotid artery examination. Invasive blood pressure measurements and echocardiographic measurements of stroke volumes and ejection fractions were used to calculate Ea and Ees by conventional means. Carotid STE was performed to analyze circumferential strain and strain-rate.

**Results**: Studying Ea was feasible using STE in all patients. Ea was correlated with circumferential strain (r = -0.37, p = 0.04) and strain-rate (r = 0.46 + p = 0.008). When using conventional methods, we demonstrated a relationship between Ea and Ees (r = 0.77, p < 0.0001), and between Ees and (1) circumferential strain (r = 0.28, p > 0.05) + (2) circumferential strain rate (-0.36, p = 0.08). There was no difference between septic and non-septic patients regarding STE, Ea or Ea Ees.

**Conclusion**: Speckle Tracking Echocardiography (STE) is a feasible way of examining the elastance of the great arteries in ICU patients. No difference was observed between septic and non-septic patients regarding arterial elastance assessed using conventional method or STE.

### P-46 Association between fluid overload and sofa score kinetic from admission to day 5 during septic shock- results of EpiGOAL study

#### Xavier Chapalain (*speaker*)^1^, Véronique Vermeersch^1^, Pierre-Yves Egreteau^2^, Gwenael Prat^3^, Eric Vicaut^4^, Olivier Huet^1^

##### ^1^Brest University Hospital, Department of anesthesiology and intensive care medicine, Brest, FRANCE; ^2^Department of intensive care medicine, Morlaix, FRANCE; ^3^Brest University Hospital, Medical ICU, Brest, FRANCE; ^4^Department of Biostatistics, Hôpital Fernand Widal (APHP), Paris, FRANCE

###### **Correspondence:** Xavier Chapalain - xavier.chapalain@gmail.com

*Annals of Intensive Care* 2019, **9(Suppl 1)**:P-46

**Introduction**: Fluid infusion is one of the cornerstones of sepsis resuscitation therapies. However, a paradigm shift is currently occurring as concerns have been raised about the potential adverse effects of fluid therapy. One of the major adverse effects reported is fluid overload (FO). FO is defined as a fluid accumulation > 10% of baseline body weight. The objective of this study was to assess influence of FO on SOFA score changes from day 0 to day 5.

**Patients and methods**: This study is a retrospective, multicenter, epidemiologic data analysis. All adult patients admitted for septic shock, caused by peritonitis or pneumonia and mechanically ventilated, were enrolled in the study. Delta SOFA score was defined as the SOFA score measured on admission minus SOFA score measured on day 5. Bivariate analysis was performed with parametric tests or non-parametric tests. A multivariate analysis was performed to study the association between FO and Delta SOFA score.

**Results**: 129 patients met the inclusion criteria of the study. Cumulative fluid balance at day 5 was greater in the FO group, with a between-group difference of 5.977 ml (p-value < 0.001). Delta SOFA score was more than two-fold higher in the no FO group than in the FO group with a difference of 2.37 between the two groups (p-value = 0.001). Patients without FO were more rapidly discharge from ICU compared to patients with FO and had one week less of mechanical ventilation compare to FO patients (p-value < 0.05). There was a stepwise decrease of delta SOFA score when duration of fluid overload was greater (p-value = 0.001). In linear modelling, the association between fluid overload status and delta SOFA score was confirmed with an adjusted relative-risk of 0.15 (p-value = 0.009).

**Conclusion**: In this study, we report that- 1) Exposition to fluid overload is a frequent event which occurs in about 40% of the patients of our cohort, 2) Fluid overload patients had more prolonged multi-organ failure during septic shock.


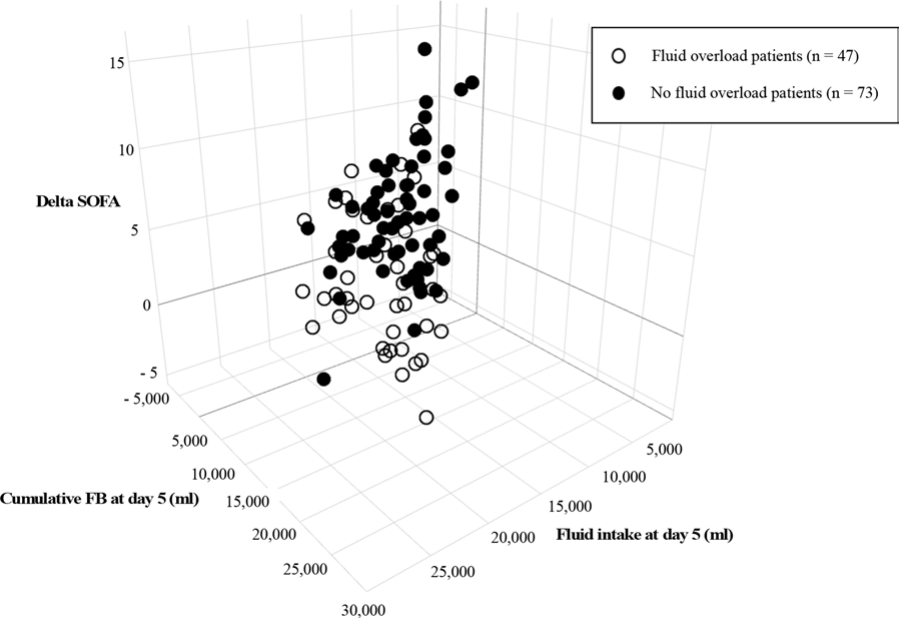


### P-47 Safety and comfort of broncho-alveolar lavage with hypnosis and HFNC in critically ill immunosuppressed patients- preliminary data

#### Virginie Lemiale (*speaker*), Laure Calvet, Michael Darmon, Sandrine Valade, Lionel Kerhuel, Elie Azoulay

##### Medical ICU APHP Saint Louis, Paris, FRANCE

###### **Correspondence:** Virginie Lemiale - vlemiale@yahoo.fr

*Annals of Intensive Care* 2019, **9(Suppl 1)**:P-47

**Introduction**: Patient with acute respiratory failure (ARF), broncho-alveolar lavage (BAL) are at high risk of intubation. Oxygenation procedure could improve safety in that setting. However those procedures are usually associated with discomfort, some patients need sedation which is also associated with a risk of intubation. Hypnosis has been suggested as useful in improving comfort.

**Patients and methods**: Observational feasibility study performed in 9 patients with ARF requiring 11 BAL. Hypnosis was induced by trained physician with technique described by Milton Erickson while fiberoptic bronchoscopies was performed by a pulmonologist after local anesthesia. All patients were highly hypoxemic. Intubation or need of non-invasive ventilation, within 24 h after bronchoscopy was recorded. After bronchoscopy, comfort was assessed. Physician who performed the hypnosis recorded the achievement of hypnosis during bronchoscopy.

**Results**: Nine patients were admitted to ICU for ARF and need BAL. Median age was 52 [40–66] years old, 4 9 (44%) patients were male. Underlying immune defect was related to HIV (2 patients), steroids treatment (1 patient), allogeneic stem cell transplant (2 patients), hematological malignancy (4 patients). Suspected diagnosis of ARF was mainly pneumocystis pneumonia in 7 patients. Mean oxygen level before bronchoscopy was 5 [3.5–6] l min and respiratory rate was 30 [25.5–32.5] min. No patient needed vasopressor before bronchoscopy.

During BAL, HFNC was mainly used (9 11 (82%)) (table 1). No patient required cessation of bronchoscopy for intolerance. Only 4 11 (36%) had cough during BAL. Mechanical invasive ventilation was required within the 24 h after bronchoscopy for one (9%) patient. Comfort was described very good by 4 11 (36%) patients, quite good for 1 11 (9%) patients, bearable for 5 11 (45%) and difficult for 1 (9%). Two patients did not reach hypnosis state during bronchoscopy and they described bronchoscopy as a bearable (1 patient) and difficult (1 patient). In other patients, hypnosis state was achieved (n = 6) or almost achieved (n = 3). One patient who experiment 3 BAL asked for hypnosis for the 2 last bronchoscopies. Pulmonologist reported they felt bronchoscopy was easier than usually with hypnosis.

**Conclusion**: Hypnosis seems feasible and safe for patient with ARF requiring BAL. It seems associated with an increase satisfaction rate for patient and pulmonologist. Additional comparative studies are needed to confirm these results.


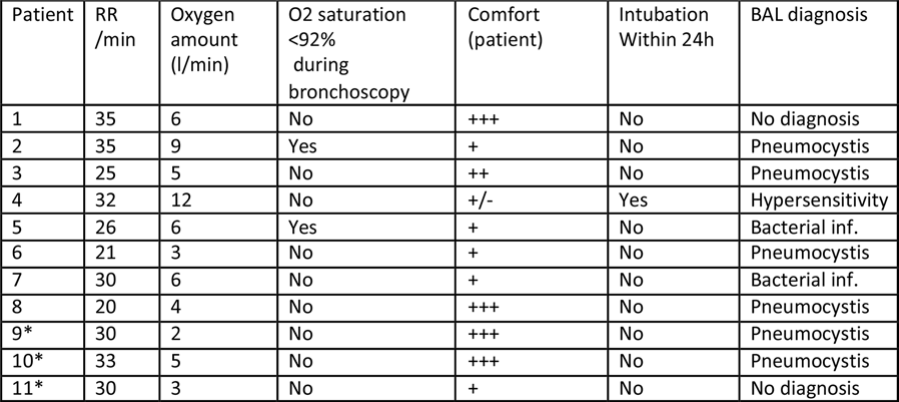


### P-48 Resuscitation residents’ knowledge, attitudes and practices regarding the modalities of administration of antiepileptic drugs by nasogastric tube in comatose patients

#### Ramla Mizouri (*speaker*)^1^, Rim Charfi^2^, Khaoula Ben Ismail^3^, Emna Gaies^2^, Anis Klouz^2^, Sameh Trabelsi^2^

##### ^1^Institut National de Nutrition de Tunis, Service A, Tunis, TUNISIA; ^2^Clinical Pharmacology Department - National Centre of Pharmacovigilance, Tunis El Manar University-Faculty of medicine, Tunis, TUNISIA; ^3^Hopital Yasminet Ben Arous, service réanimation médicale, Tunis, TUNISIA

###### **Correspondence:** Ramla Mizouri - mizouriramla@gmail.com

*Annals of Intensive Care* 2019, **9(Suppl 1)**:P-48

**Introduction**: In comatose patients, antiepileptic drugs (AED) are usually administered by nasogastric tube (NGT). This population often has residual plasma concentrations of AED outside the therapeutic range. The objective of our study was to estimate the modalities of administration of the AED by NGT in the various intensive care units in Tunis.

**Patients and methods**: Our study consisted on investigating the modalities of administration of AED’s with NGT by residents in intensive care during four months. We prepared 10 questions including demographic informations. Participation was voluntary and anonymous. The questionnary was distributed in seven intensive care departments after authorization of each head of the departement. So 40 residents were included.

**Results**: Residents sex-ratio was 0.37 and mean age was 28 ± 2 years. For the period after which the NGT replacement is performed, the average of the responses was 6 ± 3 days. Residents who thought that AED were administered at the same time as food accounted for 42% of the total population. According to 90% of residents, the dosage form administered by NGT the most used in the intensive care unit was the tablets of which 95% think that it should be crushed before administration. Before introducing the drug into the NGT, 58% of residents thought it was mixed with tap water. No resident responded that treatment is introduced dry in NGT. According to residents, the most used AED in resuscitation services were phenobarbital (75%). All residents responded that to detect a possible under or overdose, the therapeutic drug monitoring is the best way in AED adjustment in this particular population. Among the residents, 62% expressed the need for a training on the modalities of administration of the treatment by NGT to improve their knowledge.

**Conclusion**: The management of the comatose under AED requires a control of the methods of administration by NGT. Thus, an action strategy should be put in place to improve the knowledge of the medical sector on this subject.

### P-49 Upper airway viral infection and myasthenia gravis exacerbation

#### Rania Bounab (*speaker*)^1^, Nicholas Heming^1^, Diane Friedmann^1^, Bernard Clair^1^, Pierre Moine^1^, Elyanne Gault^2^, Djillali Annane^1^

##### ^1^Hopital Raymond Poincaré, Service de Réanimation Médicale, Garchess, FRANCE; ^2^Hopital ambroise paré, service de microbiologie, Boulogne Billancourt, FRANCE

###### **Correspondence:** Rania Bounab - rania.bounab@aphp.fr

*Annals of Intensive Care* 2019, **9(Suppl 1)**:P-49

**Introduction**: Myasthenia gravis (MG) is an autoimmune disorder characterized by generalized weakness (Gilhus NE. NEJM. 2016). Treatment of MG is based on immunosuppressants, mainly corticosteroids. The annual incidence of MG ranges between 0.8 to 1 cases per 100 000 (Carr AS et al. BMC Neurol. 2010). Acute exacerbations of MG are characterized by worsening muscle weakness, which may result in acute respiratory failure requiring invasive mechanical ventilation. Upper airway viral infections may induce acute exacerbations. Our aim was to describe the characteristics of myasthenia gravis exacerbations caused by upper airway viral infections, as well as their outcome.

**Patients and methods**: Retrospective cohort study undertaken in the medical ICU of a tertiary care hospital. All adult patients hospitalized for myasthenia gravis exacerbation due to a respiratory viral episode (positive multiplex PCR assay) between July 2009 and December 2017 were included.

**Results**: Upper airway viral infections caused 35 out of a total of 427 hospitalizations for MG exacerbation (8%) over an 8 year period. Affected patients were mainly female (n = 23 + 66%), IGS II- 43 ± 17, age 56 ± 18 years, all patients were treated by immunosuppressors (systemic glucocorticoids and immunosuppressants for > 3 months). Six patients (17%) required invasive mechanical ventilation. Vasopressors were required for 5 patients (14%). ICU-mortality was 11% (n = 4). Bacterial co-infection occurred in 4 patients (11%). Detected viruses were influenza (n = 24 + 69%), parainfluenza (n = 2 + 6%) and Respiratory syncytial virus (RSV) (n = 9 + 26%).

**Conclusion**: Upper respiratory airway infection is not a frequent cause of myasthenia gravis exacerbation despite our population being immunosuppressed.

### P-50 Predictive ability of Early PREdiction of DELIRium for Intensive Care (E-PRE-DELIRIC) to diagnose delirium in a low nurse-to-patient ratio Belgian ICU cohort

#### Olivier Carelli (*speaker*)^1^, Karl Brousmiche^2^, Jean-Charles Preiser^3^, Pascal Sacre^4^

##### ^1^Université Catholique de Louvain - Cliniques Universitaires Saint Luc, Maurage, BELGIUM; ^2^UCL, Etterbeek, BELGIUM; ^3^ULB, Bruxelles, BELGIUM; ^4^GHDC, Gilly, BELGIUM

###### **Correspondence:** Olivier Carelli - olivier.carelli@student.uclouvain.be

*Annals of Intensive Care* 2019, **9(Suppl 1)**:P-50

**Introduction**: The PREdiction of DELIRium for Intensive Care (E-PRE-DELIRIC) model was developed in 2012 and was recalibrated for Early PREdiction (E-PRE-DELIRIC model) in 2015. This test has been proved to be feasible and fast to do in university hospitals or university affiliated teaching hospitals. It would be of high value to confirm this in a non-university ICU. The main outcome of this study was the predictive ability of E-PRE-DELIRIC to diagnose delirium. Therefore, a correlation between a positive E-PRE-DELIRIC score (> or = 50%) and an ICDSC score < or = 4 (Intensive Care Delirium Screening Checklist) was searched. In a subsequent analysis, the performance of E-PRE-DELERIC was used in the cardiac surgery vs other patients. We also compared the abilities of E-PRE-DELERIC and APACHE II score to predict delirium.

**Patients and methods**: This study was approved by the medical ethical committee and all patients signed an informed consent. Data of all patients were collected in a secured electronic system. All consecutive patients aged > or = 18y were included during a 6-week period. Patients were excluded if they had a cognitive dysfunction known at the admission or if chronic alcoholism. The E-PRE-DELERIC score was calculated by the physician 24 h after admission. The presence of delirium was screened by the nurses during a period of five days with ICDSC scale and NICS (Nursing Instrument for Communication of Sedation) score.

**Results**: 80 patients were eligible for analysis. The overall incidence of delirium was 20% with the highest incidence of new-onset delirium on day 2. The results of the ROC analysis (Figure 1.) show that the E-PRE-DELIRIC score is highly discriminant (AUC- 0.881, 95% CI- 0.796–0.966). As seen in other studies, a higher incidence of delirium in the post cardiac surgery group (38 patients), with three times more delirium in this group compared to the general population. For the E PRE-DELERIC score, the threshold of 50% was the one that combined the maximal sensitivity and specificity (maximum Youden index = 0.703) and for the APACHE II score, the threshold would be 15 but the E-PRE-DELIRIC score has more discriminative power than the APACHE II score (AUC - 0.747, 95% CI- 0.5983–0.8948).

**Conclusion**: The E-PRE-DELIRIC delirium prediction model is reliable and remains fast and easy to do in a general non-university ICU, characterized by a great turnover of patients and of caregivers, and a rather low nurse-to-patient ratio (1–3 to 1–4), allowing early preventive measures aimed to reduce delirium incidence in those high risk patients.

**Figure:**
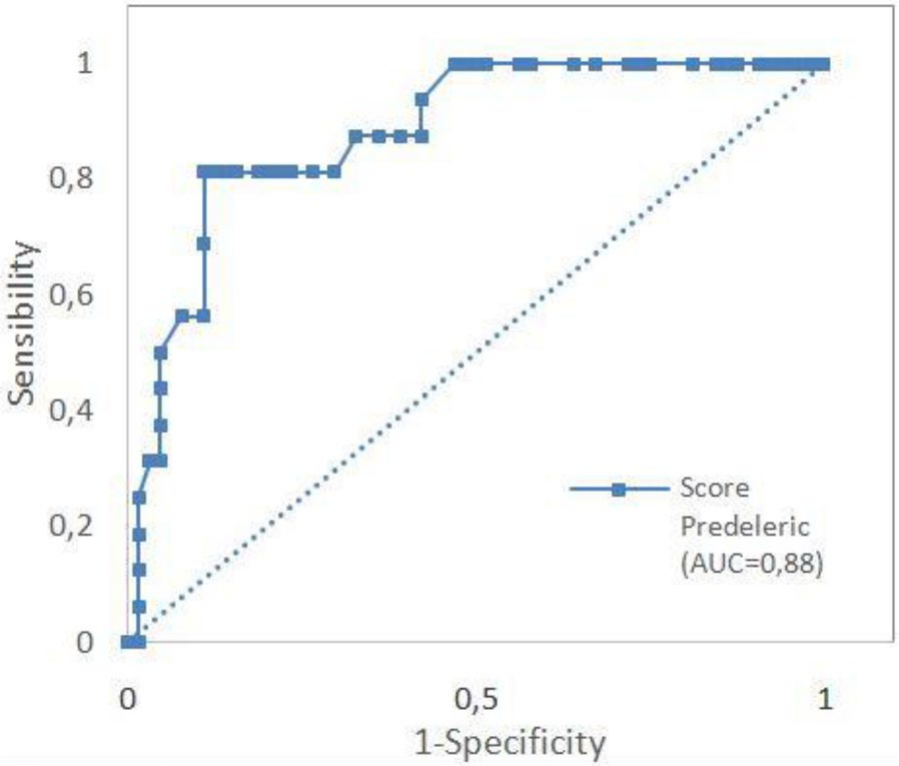
Ability of E-PRE-DELERIC score to predict delirium (ROC curve analysis)

### P-51 Myoclonus after cardiac arrest- incidence, characteristics and outcome

#### Omar Ben Hadj Salem (*speaker*)^1^, Matthieu Jamme^1^, Marine Paul^2^, Lucie Guillemet^3^, Jean-Francois Llitjos^2^, Florence Dumas^2^, Julien Charpentier^2^, Frédéric Pene^2^, Jean-Daniel Chiche^2^, Jean-Paul Mira^2^,Hervé Outin^1^, Alain Cariou^2^

##### ^1^Réanimation médico-chirurgical + CHI Poissy, Paris, FRANCE; ^2^Hôpital Cochin, Paris, FRANCE; ^3^Reanimation chirurgicale, Paris, FRANCE

###### **Correspondence:** Omar Ben Hadj Salem - omar.bhs@live.fr

*Annals of Intensive Care* 2019, **9(Suppl 1)**:P-51

**Introduction**: Post cardiac arrest myoclonus (PCAM) can occur in patients admitted after cardiac arrest (CA) and their association with the outcome is debated. We aimed to identify incidence, risk factors and outcome of PCAM in this setting. We also described clinical characteristics and EEG patterns.

**Patients and methods**: Using the Cochin CA registry (PROCAT), we retrospectively included all consecutive CA patients admitted between January and December 2016 in the ICU. All patients were treated with targeted temperature management. Main outcome was prevalence of PCAM and survival at ICU discharge. Risk factors for PCAM, clinical characteristics and EEG patterns were also assessed.

**Results**: Among the 132 patients (73.5% male, median age 66 years) included in the analysis, 37 (28%) developed PCAM during their ICU stay. PCAM were associated with an initial non-shockable rhythm (p = 0.03) and with hypoxic cause (p = 0.009). Only two patients with PCAM survived (5.4%) and only one PCAM patient had a good outcome (CPC 1), whereas 44 patients without PCAM (46.3%) survived at ICU discharge. Irreversible brain damages were the leading cause of death among patients with PCAM. We observed a strong association between myoclonus during ICU stay and mortality at ICU discharge (odds ratio 17.5 [4.2–123.2]). Clinical characteristics and EEG patterns were also reported.

**Conclusion**: PCAM were observed in nearly one third of CA patients admitted in ICU. Myoclonus were independently but not invariably associated with ICU mortality. A syndromic approach combining semiology and electrophysiology could help to use myoclonus as a tool for prognostication after CA.

### P-52 Therapeutic monitoring of antiepileptic drugs in comatose patients

#### Ramla Mizouri (*speaker*)^1^, Rim Charfi^2^, Khaoula Ben Ismail^3^, Emna Gaies^2^, Anis Klouz^2^, Sameh Trabelsi^2^

##### ^1^Institute National of Nutrition, service A, Tunis, TUNISIA; ^2^Clinical Pharmacology Department - National Centre of Pharmacovigilance –El Manar University-Faculty of medicine, Tunis, TUNISIA; ^3^Service de réanimation médicale Yasminette, Tunis, TUNISIA

###### **Correspondence:** Ramla Mizouri - mizouriramla@gmail.com

*Annals of Intensive Care* 2019, **9(Suppl 1)**:P-52

**Introduction**: If the AED are administered by nasogastric tubes (NGT) in comatose patients, evaluation of AED efficacy and toxicity may be difficult. So, the therapeutic drug monitoring (TDM) may be useful in AED adjustment in this particular population. In this study, we aimed to analyze the trough plasmatic levels (C0) of AED administered by NGT in comatose patients.

**Patients and methods**: We conducted a retrospective study on comatose patients addressed over seven years (2009–2015) for a C0 measurement of AED administered by NGT. It included 105 samples from 44 patients. AED C0 were measured by an automated chimiluminescence technique (ARCHITECT^®^-ABBOTT^®^). We used the pharmacokinetic parameter C0 Dp (dose per weight) to assess the AED bioavailability in the included patients.

**Results**: In this study, the sex-ratio was 2.38. The patients’ median age was 24 years. There was 14% of children (≤ 16 years). Among the 105 samples, C0 measurement concerned valproic acid (VPA) in 56%, phenobarbital (PNB) in 28%, carbamazepine (CBZ) in 14% and phenytoin in 2%. Two AED or more were associated in 42% of patients. AED were associated to other drugs in 85% of cases. Polymedication (≥ 5 drugs) was noted in 21% of cases. The AED C0 were subtherapeutic in 71% of cases. The VPA C0 were subtherapeutic in 88% of cases. Among the samples, 65% corresponded to a value of C0 Dp lower than the recommanded one. In these samples, 55% presented at least one drug association with the concerned AED. In 45% of the cases, there was no drug association but a non-respect of NGT modality of AED administration in patients.

**Conclusion**: TDM is a useful tool to assess drug-drug interactions and to control modalities of AED administration in comatose patients.

### P-53 Early in-hospital management of cardiac arrest from neurological cause- diagnostic pitfalls and treatment issues

#### Stephane Legriel (*speaker*)^1^, Wulfran Bougouin^2^, Richard Chocron^2^, Frankie Beganton^2^, Lionel Lamhaut^2^, Nadia Aissaoui^2^, Nicolas Deye ^3^, Daniel Jost^4^, Armand Mekontso-Dessap^5^, Antoine Vieillard-Baron^10^,Eloi Marijon^2^, Jouven Xavier^2^, Florence Dumas^2^, Alain Cariou^2^

##### ^1^Centre Hospitalier de Versailles, Le Chesnay, FRANCE; ^2^INSERM U970, Paris Cardiovascular Research Center, Paris, FRANCE; ^3^Centre Hospitalier Universitaire Lariboisiere, Paris, Paris; ^4^Brigade de Sapeurs Pompiers, Paris, FRANCE; ^5^Centre Hospitalier Universitaire Henri Mondor, Creteil, FRANCE; ^10^Centre Hospitalier Universitaire Ambroise Pare, Boulogne-Billancourt, FRANCE

###### **Correspondence:** Stephane Legriel - slegriel@ch-versailles.fr

*Annals of Intensive Care* 2019, **9(Suppl 1)**:P-53

**Introduction**: Out-of-hospital cardiac arrest (OHCA) is associated with poor short and long term outcomes. Determinants of outcome are related not only to the initial resuscitation process but also to the cause of the cardiac arrest itself. Cardiac causes of OHCA have been extensively studied in the recent decades + conversely, little is known on non-cardiac causes. In the present study, we aimed at reporting the diagnostic pitfalls and treatment issues in a large series of patients selected from a prospective population-based registry with a final diagnosis of OHCA from neurological cause (OHCA-NC). Furthermore, we also aimed to identify factors that were associated with the use of iCAG as first line investigation during the initial diagnostic check-up.

**Patients and methods**: Retrospective analysis of all consecutive patients from the Paris Sudden Death Expertise Centre (France) registry from May 2011 to September 2015 presenting with a sustained return of spontaneous circulation (ROSC) at hospital admission and a final diagnosis of OHCA-NC. Description of the early diagnostic check-up performed to identify the cause of cardiac arrest. Logistic multivariate regression to identify factors associated with immediate coronary angiography (iCAG) in OHCA-NC patients.

**Results**: Among 3542 patients with ROSC, a final diagnosis of OHCA-NC was established in 247 (7%). The early diagnostic check-up consisted in a total of 207 (84%) immediate cranial CT-scan, 66 (27%) iCAG and 25 (10%) chest CT-scan. The brain CT-scan allowed identifying a neurovascular cause in 116 (47%) patients. An iCAG was performed as the first line exam in 57 (23%) patients, in whom a final diagnosis of neurovascular cause for OHCA-NC was later identified in 41 patients. By multivariate analysis, decision for iCAG was independently associated with ST-segment elevation on post-ROSC electrocardiogram (OR, 5.94 [95%CI, 2.14–18.28] P = 0.0009), whereas an obvious cause of cardiac arrest on scene was negatively associated with iCAG (OR, 0.14 [95%CI, 0.02–0.51] P = 0.01).

**Conclusion**: OHCA-NC is a rare event that is mainly related to neurovascular causes. The initial ECG pattern may be a confounder regarding triage for early diagnostic check-up. Further studies are required to explore the potential harmfulness associated with decision to perform an iCAG in this population.

### P-54 Mood disorders in a Tunisian intensive care unit (ICU) survivors

#### Marwa Zghidi (*speaker*)^1^, Imen Ben Saida^1^, Hend Zorgati^1^, Nesrine Fraj^1^, Mohamed Ahmed Boujelben^1^, Wafa Zarrougui^6^, Badii Amamou ^7^, Mohamed Boussarsar^2^

##### ^1^Farhat Hached University Hospital, Medical Intensive Care Unit, Sousse, TUNISIA; ^2^Fattouma Bourguiba University Hospital, Department of Psychiatry, Sousse, TUNISIA

###### **Correspondence:** Marwa Zghidi - marwa_zghidi@outlook.fr

*Annals of Intensive Care* 2019, **9(Suppl 1)**:P-54

**Introduction**: Anxiety and depression are unpleasant emotions that many ICU survivors experience. Those psychiatric disorders can impact negatively their quality of life. Little is known about the factors associated with anxiety and depression after critical illness. The aim was to evaluate the incidence of anxiety and depression among critical care survivors and identify their risk factors.

**Patients and methods**: It is a mixed method study conducted in a medical ICU from January 2017 to January 2018 including all ICU survivors beyond 3 months after discharge. Data were obtained from medical records. At 3 months post-ICU discharge, patients were contacted by phone to complete the Hospital Anxiety and Depression Scale (HADS). Univariate and logistic regression analyses were used.

**Results**: 393 patients were admitted during the study period. 191 (48.6%) were discharged alive, 56(29.3%) were never successfully contacted and 21(11%) died within the 3 months’ period. 114 patients were enrolled in the study. Patients’ characteristics were - mean age 56.29 ± 17.88 years + male, 66(57.9%) + mean SAPSII, 25.04 ± 12.1 + invasive mechanical ventilation (IMV), 47(41.2%) vasopressors use, 30(26.3%). Anxiety and depressive symptoms were found respectively in 24(21.1%) and 11(9.6%) ICU survivors. Median HADS-A and HADS-D scores were respectively 4 [3–10] and 5 [4–6]. In univariate analysis, factors associated with anxiety were- age (44 ± 20 vs 59.56 ± 15, p = 0.001), female sex (62.5% vs 36.7%, p = 0.023), IMV (75% vs 32.2%, p = 0.000) and sedative use (57% vs 32.2%, p = 0.000). On multivariable logistic regression, sedative use (OR 6.1, 95%CI, [1.9 -19], p = 0.002), female sex (OR 3.08, 95%CI, [1.03 -9.2] + p = 0.044) and age (OR 0.96, 95%CI [0.94–0.99], p = 0.024) were independently associated with anxiety. Only age (41.9 ± 19 vs 57.83 ± 17, p = 0.005) was associated to depression in univariate analysis. On multivariable logistic regression, sedative use (OR 5.26, 95%CI, [1.25 -22], p = 0.023) and female sex (OR 5.07, 95%CI [1.2 -21.24] + p = 0.026) were independently associated to depression.

**Conclusion**: Mood disorders are rather common in ICU survivors. Female sex, young age and sedative use were independently associated with anxiety and depression after ICU discharge.

### P-55 Influence of the noninvasive ventilation protocol on intubations rates in patients with de novo acute respiratory failure: a systematic review of randomized trials

#### Rémi Coudroy (*speaker*), Marie Anne Hoppe, René Robert, Jean-Pierre Frat, Arnaud W. Thille

##### Service de Médecine Intensive et Réanimation, CHU de Poitiers, Poitiers, FRANCE

###### **Correspondence:** Rémi Coudroy - r.coudroy@yahoo.fr

*Annals of Intensive Care* 2019, **9(Suppl 1)**:P-55

**Introduction**: Continuous application of noninvasive ventilation (NIV), using a dedicated ventilator, with high levels of positive end-expiratory pressure (PEEP), and in an expert center could be more efficient to reverse respiratory failure. We performed a systematic review of randomized controlled trials to assess whether the protocol to carry out NIV may influence intubation rates in patients admitted to ICU for de novo acute respiratory failure.

**Patients and methods**: All randomized trials were identified from PubMed and Embase from inception to April 2018. Pediatric studies, performed outside the ICU, including patients treated for another reason than de novo respiratory failure, and in which NIV protocol was not specified were excluded. Two authors independently extracted intubation rates and the NIV protocol especially continuous application or by intermittent sessions, the type of ventilator, set PEEP levels and centers’ experience (single versus multicenter studies).

**Results**: Fourteen studies including 750 ICU-patients treated by NIV for de novo respiratory failure were identified. Overall intubation rate was 39% [33–46] and was not influenced by continuous application of NIV, the type of ventilator, or the expertise of centers. Intubation rates were lower in studies using higher PEEP levels (> 6 cm H2O) than the others (25% [15–37] vs. 35% [33–54], p = 0.025). Inclusion criteria were heterogeneous and numerous critical data on NIV were lacking.

**Conclusion**: Except high PEEP levels that might be associated with lower intubation rates, the protocol to carry out NIV does not seem influence outcomes in patients with de novo respiratory failure.

### P-56 High-flow nasal oxygen therapy- Clinical practice in French north-west intensive care units

#### Emmanuel Besnier (*speaker*)^1^, Sinad Hobeika^2^, Saad Nseir^3^, Fabien Lambiotte^4^, Damien Du Cheyron^2^, Bertrand Sauneuf^5^, Benoit Misset^2^, Fabienne Tamion^2^, Guillaume Schnell^6^, Jack Richecoeur^7^,Julien Maizel^8^, Christophe Girault^2^

##### ^1^Department of Anesthesia and Critical Care, University Hospital, Rouen, FRANCE; ^2^Department of Medical Intensive Care, Rouen, FRANCE; ^3^Department of Medical Intensive Care, Lille, FRANCE; ^4^Intensive care unit, Valenciennes, FRANCE; ^5^Intensive care unit, Cherbourg-En-Cotentin, FRANCE; ^6^Intensive care unit, Montivilliers, FRANCE; ^7^Intensive care unit, Beauvais, FRANCE; ^8^Department of Medical Intensive Care, Amiens, FRANCE

###### **Correspondence:** Emmanuel Besnier - besnier.emmanuel@gmail.com

*Annals of Intensive Care* 2019, **9(Suppl 1)**:P-56

**Introduction**: Despite an extensive use of High-Flow Nasal Oxygen therapy (HFNO) for acute respiratory failure (ARF), daily clinical practice has not been assessed yet. We therefore designed a regional survey to evaluate the physician’s clinical experience.

**Patients and methods**: We performed an observational survey (Surveymonkey online software) to senior physicians belonging to the north-west ICU Boreal Study group, during a 3 months period in 2016–2017. Thirty-four ICU were solicited. The survey included questions regarding the effective use of HFNC, indications and expected efficiency, practical aspects of use (initiation, weaning). Results are presented as medians with first and third quartiles or percentage of responses.

**Results**: 137 ICU physicians over 235 answered (58.3%). All regularly use HFNO. Admissions for ARF were 20.4 ICU bed. Indications and expected results of HFNO are presented in table 1. Hypoxemic ARF was considered as the best indication (100%) with pneumonia and thoracic trauma as favorite. Nevertheless, physicians considered HFNO efficient (i.e. avoiding intubation in at least 60% of cases) for hypoxemic ARF in only 28.6% of cases. Hypercapnic ARF was considered as a good indication for only 32.5% of physicians with an expected success rate of 1.5%. 40.2% of physicians estimated that HFNO was indicated for conventional oxygen needs > 6L/min, 39.1% for > 9L/min, 11.8% for > 12L/min and 8.8% for > 15L/min. HFNO was mainly used via a specific device (91.1%) or a ventilator (65.2%). 58% of physicians initiate HFNO with 50–100% FiO2 and progressive increase in flow. 92.8% regularly use HFNO continuously and 54.1% intermittently with NIV. Practices for HFNO weaning were heterogeneous depending on situations, but FiO2 < 30% was considered as necessary to stop HFNO for 47.8%, whereas such a FiO2 and a flow < 20 L/min was required for 29.7%. Criteria for HFNO failure (i.e. need for intubation) were ventilatory pauses or arrest (93.6%), persistent hypoxemia (94.5%), respiratory acidosis (80.9%), respiratory worsening (94.5%) and bronchial congestion occurrence (78.2%).

**Conclusion**: HFNO can be considered by ICU physicians in many situations of ARF despite a relatively low expected success rate, especially in cases of hypercapnia. Clinical practices appear very heterogeneous regarding administration and weaning modalities. Despite the physiological benefit of HFNO, these results may reflect some discrepancies between current clinical studies. Further prospective observational studies are needed on HFNO daily practices.


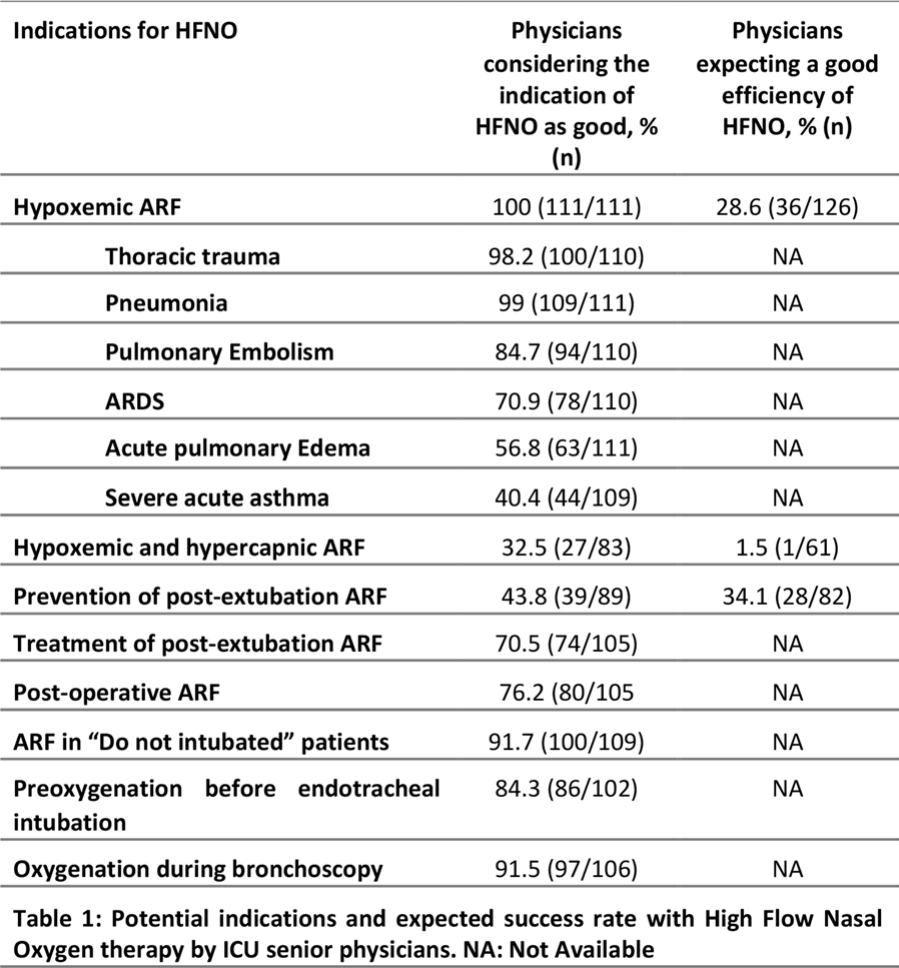


### P-57 Teaching Mechanical Ventilation for Residents in Intensive Care A randomized Trial Using Traditional Lectures VS Computer-Based Simulation (SimVA©)

#### Hadrien Roze (*speaker*)^1^, Etienne Rivière^2^, Remi Dubois^3^, Alexandre Ouattara^1^

##### ^1^SAR sud, CHU, Bordeaux, FRANCE; ^2^Service de medecine interne, CHU, Bordeaux, FRANCE; ^3^IHU Liryc, Fondation Bordeaux Université, Bordeaux, FRANCE

###### **Correspondence:** Hadrien Roze - hadrien.roze@chu-bordeaux.fr

*Annals of Intensive Care* 2019, **9(Suppl 1)**:P-57

**Introduction**: During educational process, trainees apply their knowledge to treat patient in intensive care before achieving full clinical competency. Moreover, advances in knowledge regarding mechanical ventilation in particular lung protective ventilation and asynchronies have been shown to be associated with mortality. For these reasons we developed a simulator of controlled and spontaneous artificial ventilation (SimVA) and virtual breathing patients. Mathematical model resolved differential equations of chest and lung movements according to inspiratory effort or not in order to match with a clinical database. The aim of this study was to compare two teaching modalities on mechanical ventilation- traditional lectures versus virtual simulation.

**Patients and methods**: This randomized controlled study involved 54 residents. One group of 22 participants attended the same didactic lecture on mechanical ventilation (3 h) whereas the other 22 were in the simulator group (3 h). Performance was measured using a pre and post-test evaluation of knowledge on respiratory settings and pressure flow time curves monitoring. A retention test was done at 3 months (The same questioner was used for pre, post and retention test). Comparison was individual in each group (ANOVA, multiple comparison) and between groups (Mann–Whitney), p < 0.05 was considered significant.

**Results**: Baseline knowledge was not different between groups, post-test was significantly improved in both groups (figure) but was significantly higher in the simulator group. Retention test was only significantly different from the pre-test in the simulator group.

**Conclusion**: A computer-based simulation with a modelisation of controlled and spontaneous mechanical ventilation has the potential to improve knowledge and skills in ventilator settings in comparison to traditional didactic lectures.


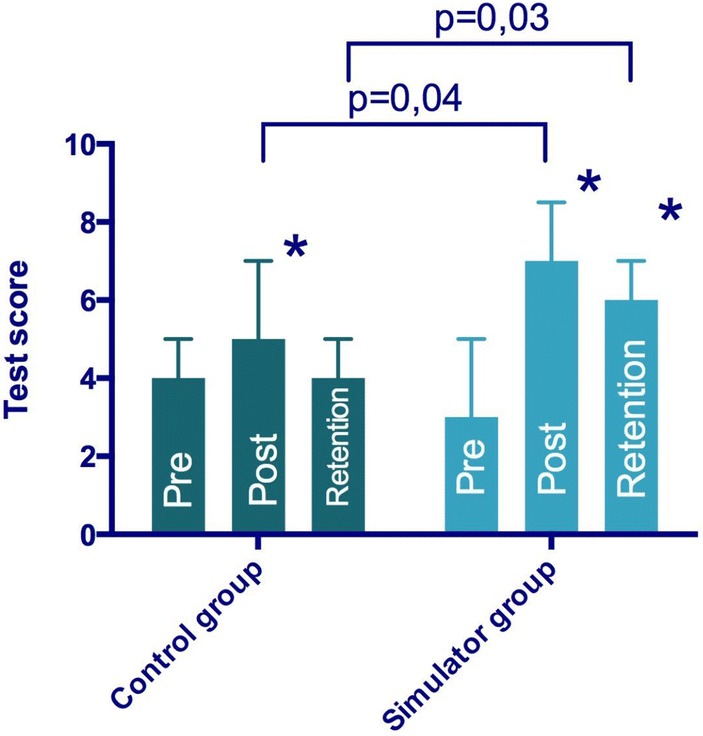


### P-58 Pressure support titration to personalize assistance- preliminary results

#### Francois Perier (*speaker*)^1^, Tommaso Maraffi^1^, Bruno Louis^2^, Keyvan Razazi^1^, Nicolas De Prost^1^, Armand Mekontso Dessap^1^, Guillaume Carteaux^1^

##### ^1^Service de Réanimation Médicale, Hôpitaux Universitaires Henri Mondor – Albert Chenevier, Assistance Publique – Hôpitaux de Paris (AP-HP), Créteil, FRANCE; ^2^IMRB Inserm U955, Créteil, FRANCE

###### **Correspondence:** Francois Perier - francoisperier@hotmail.fr

*Annals of Intensive Care* 2019, **9(Suppl 1)**:P-58

**Introduction**: Usually, clinicians do not measure the respiratory effort under mechanical ventilation, as it requires the use of an esophageal catheter. Keeping patient’s respiratory effort within a normal range during pressure support ventilation (PSV) is therefore difficult to achieve. The main aims of this study were- 1) to describe the relation between the respiratory pattern and respiratory effort indices over a wide titration of pressure support level (PSL) in PSV, 2) to assess new calculated indices of respiratory effort that do not require an esophageal catheter.

**Patients and methods**: Patients were eligible during the first 48 h after the switch from controlled ventilation to PSV. The PSL was decreased by steps of 2 cmH2O from 20 to 6 cmH2O. Flow, airway and esophageal pressures, and electrical impedance tomography (EIT) signals were continuously recorded during the titration. P0.1 was measured at each step. Indices of respiratory effort, including muscle pressure (Pmus) and esophageal pressure–time product (PTPes) were derived from recorded signals. The calculated Pmus (Pmuscalc) and PTPes were computed using the equation of motion of the respiratory system and respiratory mechanics previously collected while patients were under controlled ventilation.

**Results**: These preliminary results involve the first five patients. Tidal volume (Vt) variations over the PSL titration depended on the basal level of respiratory effort. Vt values remained relatively stable during normal or underassisted efforts (PTPes above 50 cmH2O.s.min-1) and increased dramatically in case of overassistance (PTPes below 50 cmH2O.s.min-1) (Figure 1). The Pmuscalcl calculated at peak inspiratory flow correlated with the actual peak of Pmus (r = 0.34 + p < 0.0001). In addition, P0.1 seemed better correlated with Pmus (r = 0.76 + p < 0.0001) than Pmuscalcl. Definitive results, including EIT assessment, will be presented at the meeting.

**Conclusion**: These preliminary data suggest the potential interest of analyzing Vt values evolution over a PSL titration. Furthermore, new calculated tools, as Pmuscalc may help clinicians assessing respiratory effort at the bedsides and personalizing the level of assistance.


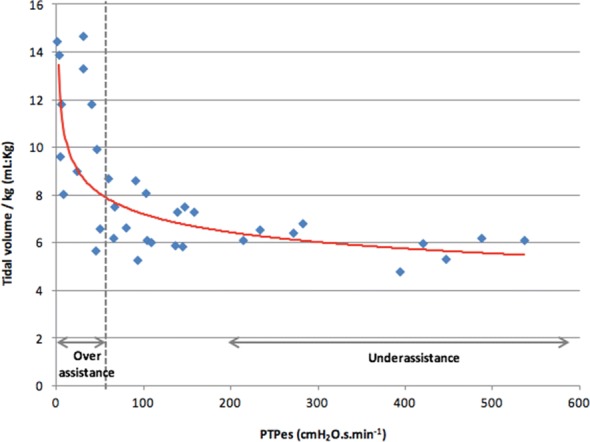


### P-59 Body mass index and outcome of immunocompromised ICU patients

#### Audrey De Jong (*speaker*)^1^, Peter Pickkers^2^, Marcio Soares^3^, Anders Perner^4^, Philippe Bauer^5^, Jordi Rello^6^, Andry Van de Louw ^7^, Pleun Hemelaar^8^, Virginie Lemiale^9^, Fabio Silvo Taccone^10^,Ignacio Martin Loeches^11^, Tine Sylvest Meyhoff^4^, Jorge Salluh^3^, Peter Schellongowski^12^, Katerina Rusinova^13^, Nicolas Terzi^14^, Sangeeta Mehta^15^, Massimo Antonelli^16^, Achille Kouatchet^17^, Andreas Barrat Due^18^, Miia Valkonen^19^, Pearl Landburg^20^, Fabrice Bruneel^21^

##### ^1^CHU SAINT LOUIS and CHRU de Montpellier - Hôpital Saint-Eloi, Paris, FRANCE; ^2^Radboud University Medical Center, Nijmegen, THE NETHERLANDS; ^3^The Department of Critical Care and Graduate Program in Translational Medicine, D’Or Institute for Research and Education, Programa de Pós-Graduação e, Rio de Janeiro, BRAZIL; ^4^Department of Intensive Care, Rigshospitalet, University of Copenhagen, DENMARK; ^5^Pulmonary and Critical Care Medicine, Mayo Clinic, Rochester, UNITED STATES; ^6^CIBERES, Universitat Autonòma de Barcelona, European Study Group of Infections in Critically Ill Patients (ESGCIP), Barcelone, SPAIN; ^7^Division of Pulmonary and Critical Care, Penn State University College of Medicine, Hershey, UNITED STATES; ^8^The Department of Intensive Care Medicine (710), Radboud University Medical Center, Nijmegen, THE NETHERLANDS; ^9^Hopital Saint Louis, Paris, FRANCE; ^10^Department of Intensive Care, Hôpital Erasme, Université Libre de Bruxelles (ULB), Bruxelles, BELGIUM; ^11^Department of Intensive Care Medicine, Multidisciplinary Intensive Care Research Organization (MICRO), St. James’s Hospital, Dublin, IRELAND; ^12^Department of Medicine I, Medical University of Vienna, Vienne, AUSTRIA; ^13^Department of Anesthesiology and Intensive Care Medicine and Institute for Medical Humanities, 1st Faculty of Medicine, Charles University, Prague, CZECH REPUBLIC; ^14^CHU Grenoble Alpes, Service de réanimation médicale, Faculté de Médecine de Grenoble, INSERM, U1042, Université Grenoble-Alpes, Grenoble, FRANCE; ^15^Department of Medicine and Interdepartmental Division of Critical Care Medicine, Sinai Health System, University of Toronto, CANADA; ^16^Agostino Gemelli University Hospital, Università Cattolica del Sacro Cuore, Rome, ITALY; ^17^Department of Medical Intensive Care Medicine, University Hospital of Angers, FRANCE; ^18^Department of Emergencies and Critical Care, University Hospital, Oslo, NORWAY; ^19^Division of Intensive Care Medicine, Department of Anesthesiology, Intensive Care and Pain Medicine, University of Helsinki and Helsinki University Ho, Helsinki, FINLAND; ^20^Department of Critical Care, University Medical Center, Groningen, THE NETHERLANDS; ^21^Center Hospitality, Medical-Surgical Intensive Care Unit, Versailles, FRANCE

###### **Correspondence:** Audrey De Jong - audreydejong@hotmail.fr

*Annals of Intensive Care* 2019, **9(Suppl 1)**:P-59

**Introduction**: In the immunocompromised intensive care unit (ICU) population, the association of prognosis and body mass index (BMI) has never been assessed. The objectives of this study were to determine- 1) the influence of BMI on ICU immunocompromised patient’s mortality. 2) the risk factors of ICU mortality according to the BMI.

**Patients and methods**: A post hoc analysis of a prospective, multinational cohort study, performed in 16 countries in immunocompromised acute-respiratory-failure (ARF) patients, was conducted. The primary endpoint was ICU-mortality rate. Impact of BMI on ICU-mortality and risk factors according to BMI were analysed using multivariate Cox analysis. The best threshold for separating dead from alive patients was determined using the Youden index.

**Results**: In the 1483 patients with BMI available, the overall ICU-mortality rate was 32% (470/1483). Ninety-one (6%) patients were underweight (32% ICU-mortality), 646 (44%) normal weight (32% ICU-mortality), 457 (31%) overweight (31% ICU-mortality), 172 (12%) obese grade 1 (33% ICU-mortality), 80 (5%) obese grade 2 (29% ICU-mortality) and 37 (2%) obese grade 3 (24% ICU-mortality). After multivariate Cox analysis including initial oxygenation strategy, Sequential Organ Failure Assessment (SOFA) score without respiratory item, PaO2 FiO2 ratio < 100 at ICU admission, invasive mechanical ventilation, pattern of chest X ray and etiology of ARF, higher BMI was significantly associated with lower mortality rate (Adjusted HR (aHR) = 0.98(0.97–1.00), P = 0.02). No centre effect was observed. Two BMI groups were separated- low (< 28 kg/m2) and high (BMI ≥ 28 kg/m2), the adjusted risk of dying being lower in the high BMI (≥ 28 kg/m2) group compared to the low BMI (< 28 kg/m2) group- (aHR = 0.81(0.65–1.00), P = 0.05). After multivariate Cox analysis, some risk factors of mortality were common to both groups- SOFA score without respiratory item, NIV use and invasive mechanical ventilation, other found only in the low BMI group- PaO2/FiO2 ratio < 100 mmHg and alveolar-interstitial radiologic pattern (Figure 1).

**Conclusion**: In immunocompromised ARF patients, higher BMI was associated with lower mortality. Contrary to the low BMI group, PaO2/FiO2 ratio and radiological pattern were not associated with mortality in the high BMI group. These results, suggesting that PaO2/FiO2 ratio and radiological pattern are not reliable to predict prognosis of obese ARF patients, might be one explanation to the inverse adjusted association observed between ICU mortality and BMI.


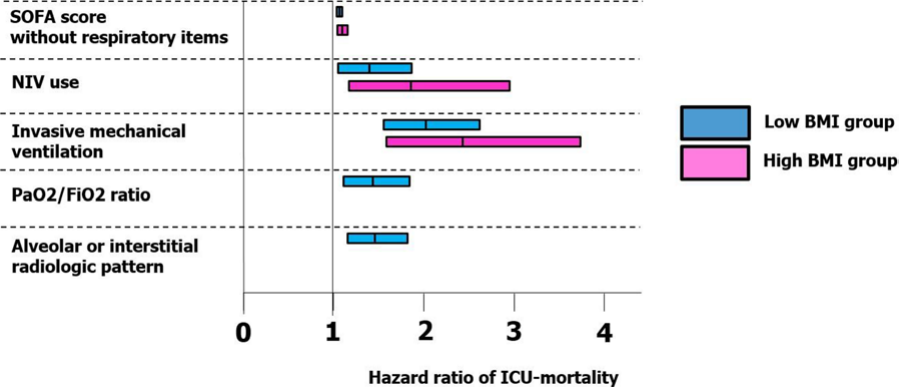


### P-60 Airway pressures and difficult weaning prediction in mechanically ventilated patients

#### Wafa Zarrougui (*speaker*), Nesrine Fraj, Mohamed Ahmed Boujelben, Dhouha Ben Braiek, Hend Zorgati, Marwa Zghidi, Ahmed Khedher, Imen Ben Saida, Abdelbaki Azouzi, Khaoula Meddeb, Mohamed Boussarsar

##### 2Farhat Hached University Hospital, Sousse, TUNISIA

###### **Correspondence:** Wafa Zarrougui - wafa.zarrougui91@gmail.com

*Annals of Intensive Care* 2019, **9(Suppl 1)**:P-60

**Introduction**: Mechanical Ventilation (MV) is intended to improve gas exchange and offset the work of breathing. Newtonian equation of motion clearly illustrates the respective restrictive and or elastic components that could impede ventilation. Static and dynamic airway pressures (Paw) may be of valuable assistance to weaning process. The aim was to identify the discriminative properties of Paw to predict difficult weaning.

**Patients and methods**: A chart reviews of consecutive MV patients admitted to our MICU from November 2015 to February 2018 was performed. Patients’ characteristics at admission, Paw (at admission and at day 4), high pressure ratio (HPR = number of days spent with high pressures- peak >=40 cmH2O and/or plateau >=30 cmH2O and/or driving >=14 cmH2O and/or auto-PEEP >=6 cmH2O, divided by LOS) and outcomes were recorded. Difficult weaning was expressed by prolonged duration of MV > = 7 days, null ventilator-free days at day 28 (VFDs), and a composite outcome - death and or LOS > = 14 days. Univariate and multivariate regression analyses were performed to identify factors independently associated to difficult weaning.

**Results**: Were included 304 MV patients. Their main characteristics were - mean age, 56 ± 18 years + mean SAPSII, 35 ± 14 + pH, 7.3 ± 0.1 + pCO2, 50 ± 23 mmHg + PaO2 FiO2, 204 ± 101 mmHg + AE COPD, 105(34.5%) + ARDS, 25(8.2%) + median MV duration, 6[3 + 14] days + LOS, 13[6 + 21] days + tracheostomy, 44(14.5%) and mortality, 173(56.9%). Mean Paw were respectively for peak, plateau, driving and auto-PEEP at admission- 32.3 ± 9.2, 20.4 ± 6, 13.4 ± 5, 4.4 ± 4.2 cmH2O and at day 4- 32.6 ± 10, 20.9 ± 6.6, 13.8 ± 5.3, 6.5 ± 4.4 cmH2O. Median HPR was 0.15[0–0.6]. Multivariate analysis yielded the following variables as independently associated to the studied endpoints- 1) VFDs- plateau at day 4 (OR, 1.06 + 95%CI, [1.01–1.12] + p = 0.019) and HPR (OR, 1.07 + 95%CI, [1.01- 1.14] + p = 0.03) + 2) prolonged duration of MV - HPR (OR, 3.4 + 95%CI, [2.2- 5.7] + p = 0.000) + 3) composite outcome- plateau at day 4 (OR, 1.13 + 95%CI, [1.05- 1.22] + p = 0.01) and HPR (HR, 1.97 + 95%CI, [1.04–1.37] + p = 0.01).

**Conclusion**: High plateau pressure and the number of days spent with high Paw seem to alter significantly the weaning.

### P-61 Reproducibility of real-time shear wave ultrasound elastography, a new tool for the evaluation of diaphragm and limb muscles stiffness in critically ill patients- preliminary results of the ULTRAMUSCLE Study

#### Yassir Aarab (*speaker*)^1^, Aurélien Flatres^2^, Stefan Matecki^2^, Mathieu Amalric^3^, Jean-JacquesTudesq^3^, Romaric Larcher^3^, David Chapeau^3^, Samir Jaber^4^, Kada Klouche^3^, Boris Jung^3^

##### ^1^CHU Montpellier - Saint Eloi, Montpellier, FRANCE; ^2^Inserm U1046, CNRS UMR9214, Université de Montpellier, FRANCE; ^3^CHU Lapeyronie, Réanimation médicale, Montpellier, FRANCE; ^4^CHU Saint-Eloi, Département d’anesthésie-réanimation B, Montpellier, FRANCE

###### **Correspondence:** Yassir Aarab - aarabyassir89@gmail.com

*Annals of Intensive Care* 2019, **9(Suppl 1)**:P-61

**Introduction**: In critically ill patients, Intensive care unit (ICU)-acquired weakness is a complication commonly observed and is associated with poor outcomes and increased length of stay. The assessment of skeletal muscle and diaphragm ICU-associated morphological changes is usually appreciated by ultrasonography. Shear wave elastography (Swe) is a novel technique which allows to measure the absolute elasticity value (kPa) of soft tissues and to quantify their mechanical properties. We therefore aimed to determine the reliability and reproducibility of ultrasonographic Swe measurements in critically ill patients.

**Patients and methods**: In a first phase, two operators tested, in 16 healthy subjects, various probe positions in order to select the most reproducible. Among them, the best 3 probe positions were used to examine their intra and inter-operator reliability in 15 other healthy subjects. In the second phase, intra and inter-operator reliability of elastography was evaluated in 12 critically ill patients. Each operator was blinded to the measurements of the other one. IRB was obtained (2017-CLER-MTP-09-16 Reliability was calculated using the intra-class correlation coefficient (ICC) and a bootstrap sampling method assessed their consistency.

**Results**: The measurements of elastic modulus of diaphragm, biceps brachii, and rectus femoris on 31 healthy subjects and 12 critically ill patients leaded to the record of 622 Swe elastograms. In the first group of healthy subjects, inter-operator reliability favored longitudinal view rather than the transverse one except for diaphragm. (Respective ICC- 0.29 vs 0.83, 0.88 vs 0.39, and 0.86 vs 0.7). In the second group of healthy subjects, no significant differences were found in the 3 muscles stiffness measurements between the 2 operators results. A very satisfactory inter-operator and intra-operator reliability was observed for all measurements with an ICC values ranging from 0.76 to 0.96 (table 1). In critically ill patients, within-operator and between-operator reliability of Swe measurements was also satisfactory (ICC = 0.83–0.98) (table 1). The probability to reach inter-rater ICCs greater than 0.8 in a 10.000 bootstrap sampling was respectively 98, 100 and 72%.

**Conclusion**: In critically ill patients, the feasibility, reproducibility and reliability of ultrasonographic SWe measurements of limb muscles and the diaphragm assessed herein were fair. Though our results may encourage the promotion of this technique in ICU settings, its application to the understanding and management of ICU-acquired weakness needs to be further studied.

### P-62 Acquired von Willebrand syndrome in patients with extracorporeal CO2 removal

#### Amélie Couteau-Chardon (*speaker*)^1^, Nadia Rivet^2^, Jean-Loup Augy^1^, Damien Vimpere^1^, Aymeric Lancelot^1^, Morgane Commereuc^1^, Clotilde Bailleul^1^, Nadia Aissaoui^1^, Etienne Puymirat^3^, Emmanuel Guerot^1^, Ana Novara^1^, David Smadja^2^, Pascale Gaussem^2^, Jean-Luc Diehl^1^

##### ^1^Medical Intensive Care Unit, Georges-Pompidou European Hospital, Paris, FRANCE; ^2^Hemostasis laboratory, Georges-Pompidou European Hospital, Paris, FRANCE; ^3^Department of cardiology, Georges-Pompidou European Hospital, Paris, FRANCE

###### **Correspondence:** Amélie Couteau-Chardon - a.couteau.chardon@gmail.com

*Annals of Intensive Care* 2019, **9(Suppl 1)**:P-62

**Introduction**: Acquired von Willebrand syndrome (AVWS) is a hemostasis disorder widely described with different extracorporeal circulatory assistances. Conversely, very few data are available in the field of extracorporeal CO2 removal (ECCO2R). We report the results of a prospective monocentric study focusing on von Willebrand disorders induced by ECCO2R devices.

**Patients and methods**: Prospective study performed in 20 consecutive patients treated with ECCO2R (either Hemolung^®^ or iLA activve^®^ device). Biological testing included- platelet function analyzer–adenosine diphosphate (PFA-ADP) and –epinephrine (PFA-EPI), von Willebrand factor antigen (VWF -Ag) and activity (VWF -RCo), and multimeric profile (Hydrasys 2 system). Measurements were performed prior to ECCO2R initiation, then at 5, 30, 60 and 180 min, daily during ECCO2R from D1 to D7, and 24 h after stopping ECCO2R. Bleeding and thrombotic events were recorded.

**Results**: Hemolung^®^ was used in 8 patients, and iLA activve^®^ in 12 between January 2017 and July 2018. The indications for ECCO2R were acute exacerbation of chronic obstructive pulmonary disease (n = 16), acute respiratory distress syndrome (n = 3), and severe acute asthma (n = 1). Significant prolonged PFA-ADP (151 s of median time at D1 versus 82 s at D0, p = 0.0001) and PFA-EPI (214 s at D1 versus 106 at D0, p = 0.0017) were observed in 19 patients (one patient with missing data) during ECCO2R, and normalized in respectively 11 and 10 among the 14 patients tested 24 h after stopping ECCO2R. The VWF -RCo VWF -Ag ratio decreased for all patients between 60 min and D1 (0.65 of median ratio at D1 versus 0.93 at D0, Figure 1A, 1B). The ratio was normalized 24 h after ECCO2R cessation in 8 of the 10 patients tested. Loss of high molecular weight multimers occured in all patients tested (n = 14) as early as 60 min (0.48 of median ratio at D1, versus 1.05 at D0) and was associated with an increase in low molecular weight multimers (Figure 1C, 1D). Fourteen bleeding events were reported. Nine thrombotic events were reported (6 at the right internal jugular cannulation site, 3 device’s thrombosis). There was a trend to a more pronounced decrease in high molecular weight multimers in patients with bleeding events.

**Conclusion**: Von Willebrand disorders were observed in all ECCO2R patients. They may contribute at least in part to the frequency and severity of bleeding complications observed in this population.


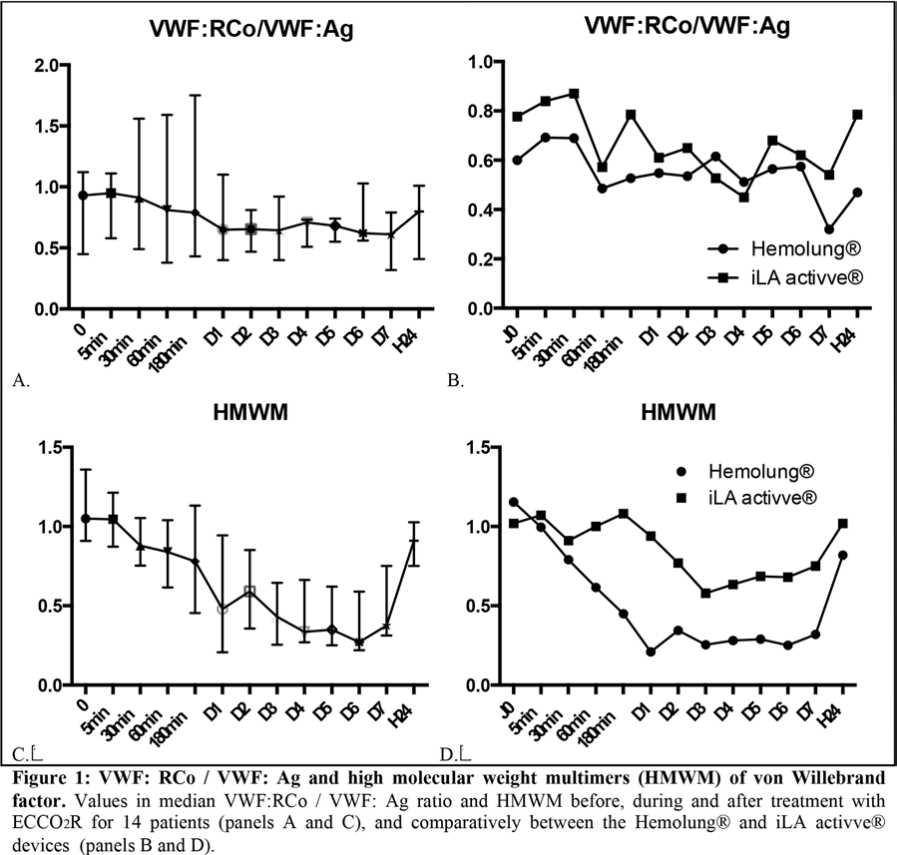


### P-63 Acute exacerbation of chronic obstructive pulmonary desease: prognostic and patients’s characteristics

#### Hela Kallel, Bouchaala Karama, Bradii Sabrine, Triki Amal, Olfa Turki, Mabrouk Bahloul, Mounir Bouaziz

##### CHU Habib Bourguiba, Service de reanimation medicale, Sfax, TUNISIA

###### **Correspondence:** Hela Kallel- hela.kallel.fourati@gmail.com

*Annals of Intensive Care* 2019, **9(Suppl 1)**:P-63

**Introduction**: We aimed to identify the characteristics of patients with severe acute exacerbation of chronic obstructive pulmonary disease (COPD) and evaluate the prognostic of COPD patients.

**Patients and methods**: This is a retrospective study performed during a 10-year period in the ICU of Habib Bourguiba University Hospital (Sfax, Tunisia). All patients with severe acute exacerbation of COPD were included.

**Results**: During the study period, 169 patients with acute exacerbation of COPD were admitted in our ICU, 65(38.5%) were emphysema patients. The mean age (± SD) was 67.99 ± 9.8 years, the sex ratio was at 7.04. The most common comorbidities were hypertension in 54(32%) patients, cardiac failure in 36(21.3%) situations and diabetes in 32(18.9%) cases. The mean SAPSII score was 38.34(± 13.29). On ICU admission 94 of patients have acute circulatory failure and 124(73.4%) patients required mechanical ventilation. Mortality rate in this study was around 49.1%, length of stay (LOS) was at 8 [1 - 69] and length of invasive ventilation medians was 8[1 + 39]. During their ICU stay evolution was marked by death of 83(49.1%) patients mortality was significantly higher in patients with cardiac failure (63.88% vs 44.71%, P = 0.043) and those who necessitate the introduction of catecholamines in admission (60% vs 23% P = 0.000). A high plasma urea and plasma creatinine concentration were significantly associated with mortality P = 0.000, P = 0.003 respectively.

**Conclusion**: COPD exacerbation is a frequent cause of hospitalization in ICU, severe form requiring mechanical ventilation were associated with a poor outcomes. Development of shock and acute kidney injury were significantly associated with mortality.

### P-64 Predictors of invasive ventilation requirement in acute exacerbations of chronic obstructive pulmonary disease

#### Jihene Guissouma (*speaker*), Sourour Belhaj Youssef, Hatem Ghadhoune, Hana Benali, Habib Brahmi, Sana Karrat

##### Faculté de médecine de Tunis (TUNISIA), Bizerte, TUNISIA

###### **Correspondence:** Jihene Guissouma - bahri.jihene@yahoo.fr

*Annals of Intensive Care* 2019, **9(Suppl 1)**:P-64

**Introduction**: Acute exacerbations of chronic obstructive pulmonary disease (AECOPD) are the most important events characterizing respiratory illness progression. Their management often needs intensive care unit hospitalization and ventilator support either noninvasive ventilation (NIV) or invasive ventilation (IV). The objective of our study was to describe the epidemiologic and clinical characteristics of patients admitted for AECOPD, the treatment and the evolution in ICU in order to deduce the factors predicting IV.

**Patients and methods**: A 4-year retrospective analytic observational single-center study including patients with AECOPD. Statistical analyses were performed with SPSS 23.

**Results**:

Eighty nine patients were enrolled. Mean age was 67 ± 9 years and male female ratio was 3.9. Seventy nine percent were smokers and 50% were classified GOLD stage 3. History of noninvasive ventilation (NIV) and invasive ventilation (IV) were found in 64% and 34% of all cases respectively. Mean progression of COPD was 9 ± 5 years and mean exacerbations frequency was 1.89 ± 0.9 per year. Mean duration of symptoms before hospitalization (DSBH) was 5 ± 4 days. Mean SAPS II and APACHE II were 38 ± 15 and 20 ± 8 respectively. Mean pH, paCO2 and paO2 fiO2 were 7.28 ± 0.2, 80 ± 28 mmHg and 221 ± 105 respectively. Mean GCS was 11 ± 4 and it was less than 9 in 25% of all cases. The predominant precipitating factor for acute exacerbation (AE) was respiratory tract infection (69% of all cases). Thirty two percent of our patients had a septic choc at admission. Symptomatic treatment was based on inhaled bronchodilators, corticosteroids and mechanical ventilation- NIV in 70% and IV in 61% of all cases. Twenty two patients were intubated immediately after hospitalization and 32 after NIV failure. Mortality was 40%. The duration of COPD progression (p = 0.039), exacerbations frequency per year (p = 0.013), prior IV (p = 0.04), DSBH (p = 0.002), SAPS II (p = 0.003), GCS (p < 10^−3^), pH (p = 0.01) and septic choc at admission (p = 0.004) were all predictive of IV in univariate analysis but only GCS (p = 0.002) was significant in multivariate analysis.

**Conclusion**: Requirement of IV in AECOPD depends on the severity of the underlying respiratory illness, the gravity of the AE and the quality of an early management. Thus, basic treatment improvement and an early appropriate treatment of the AE might reduce its severity and the need of IV.

### P-65 CORE- REA preliminary study - COPD Right hEart and REspiratory Acidosis

#### Jean-Loup Augy (*speaker*)^1^, Jean-Luc Diehl^1^, Aymeric Lancelot^1^, Clotilde Bailleul^1^, Damien Vimpere^1^, Amélie Couteau^1^, Ana Novara ^1^, Emmanuel Guerot^1^, Nadia Aissaoui^2^

##### ^1^HEGP Critical care department, Paris, FRANCE; ^2^AP-HP-HEGP-Université Paris Descartes, Paris, FRANCE

###### **Correspondence:** Jean-Loup Augy - augyjeanloup@hotmail.com

*Annals of Intensive Care* 2019, **9(Suppl 1)**:P-65

**Introduction**: Influence of respiratory acidosis on right ventricular (RV) function in severe exacerbation of chronic obstructive pulmonary disease (COPD) patients placed under invasive mechanical ventilation (IMV) remains unclear. Few previous studies reported conflicting results and respiratory acidosis might decrease contractile function in cardiomyocytes. Veno-venous extracorporeal CO2 removal (ECCO2R), increasingly used in the setting of COPD allows acute modifications of PaCO2 and pH. We aimed to detect the influence of PaCO2 decreased with ECCO2R on the RV systolic function assessed by variation of the systolic velocity wave at tricuspid annulus (St).

**Patients and methods**: This was a monocentric observational ancillary study of the REXECOR study (NCT02965079). All patients presenting with severe exacerbation of COPD and implanted by ECCO2R [Hemolung (Alung Technologies, Pittsburgh, USA)] were assessed by transthoracic echocardiography (TTE) at baseline and one hour after starting ECCO2R. IMV ventilation parameters were kept unmodified two hours before and one hour after the beginning of ECCO2R. TTE data, IMV parameters and arterial blood gas were simultaneous collected.

**Results**: Seven patients [(5 women, median age 63 years (56–73), median body mass index 39.5 kg m2 (24.1–47.0)] were included from November 2017 to February 2018. Median SAPS2 at admission was 32(29–40) and respiratory SOFA was 3(2–3). Regarding TTE findings, the 7 patients had RV hypertrophy with RV free wall superior to 5 mm (6.9 mm ± 0.9) and no RV dysfunction was reported (Mean St = 20.1 ± 5.1 cm s and mean TAPSE = 21.8 ± 5.7 mm). The ECCO2R induced a significant variation of PaCO2 from 68 mmHg (67–75) to 57 mmHg (52–59), P < 0.009 and of pH from 7.24 (7.23–7.28) to 7.37 (7.27–7.37), P = 0.025. Figure 1 shows evolution of echo parameters and PaCO2 after ECCO2Rimplantation. There was no difference between S’ wave value after significant reduction of PaCO2 by ECCO2R (p = 0.16) whereas systolic pulmonary arterial pressures (sPAP) decreased significantly from 45.2 mmHg (± 5.4) to 38.0 mmHg (± 7.6), P = 0.01. Pulmonary vascular resistance (PVR) was also not significantly decreased [2.24UW (± 0.41) vs. 1.80UW (± 0.39), P = 0.07]. Of note, parameters assessing the LV function did not change during the ECCO2R flow variations.

**Conclusion**: In the physiological study, the decreased of PaCO2 induced by the ECCO2r flow variations lead to a decreased of sPAP without modifying the systolic RV function parameters.


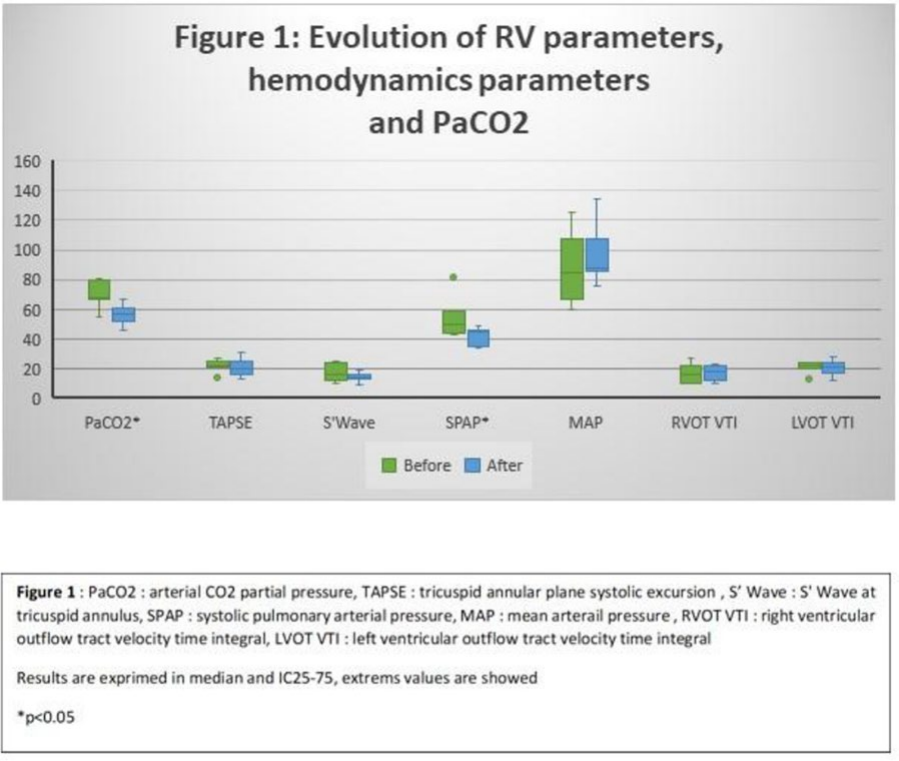


### P-66 Do comorbidity phenotypes in COPD impact the outcome of ICU admission following COPD exacerbation

#### Syrine Maatouk (*speaker*), Wiem Nouira, Zeineb Hamouda, Manel Lahmar, Nacef Ben Mrad, Islem Ouanes, Fahmi Dachraoui , Fekri Abroug, Lamia Besbes

##### CHU Fattouma Bourguiba Monastir, Monastir, TUNISIA

###### **Correspondence:** Syrine Maatouk - syrinemaatouk@gmail.com

*Annals of Intensive Care* 2019, **9(Suppl 1)**:P-66

**Introduction**: “Comorbidomes” correspond to comorbidities that are frequently associated in COPD (such as cardiovascular disease, diabetes, oetoporosis, sarcopenia) carrying a significant impact on mortality. The distribution and the type of comorbidities vary between studies but agreement exists on five phenotypes of comorbidities-The current study was conducted to determine the association between specific comorbidities and ICU survival of acute exacerbation of COPD.

**Patients and methods**: In Consecutive patients admitted to the ICU for hypercapnic exacerbation of COPD were classified according to associated comorbidities in five clusters- cluster 1 included cardiac profile + cluster 2 included less comorbidities + cluster 3 included metabolic syndrome, apnea and anxiety-depression + cluster 4 included denutrition and sarcopenia, and cluster 5 included bronchiectasis. Patients had standard ventilatory (NIV or standard MV), and pharmacologic (nebulized ß2 agonists, steroids, and anticholinergics) management. The discharge status (dead or alive) was compared between the 5 identified comorbid clusters.

**Results**: During the study period, 81 patients (mean age = 67 ± 9 years, 72% male) were consecutively admitted to the ICU for a definitive diagnosis of AECOPD. NIV was used as the primary ventilator modality in 83% while the remaining had conventional invasive ventilation. ICU mortality occurred in 25% following a mean ICU and ventilatory support duration of 17 ± 10 days and 10 ± 7 days, respectively. The figure depicts relative mortality in each comorbidities cluster. The difference in observed mortality rates between clusters was statistically significant.

**Conclusion**: The current study shows that comorbidity phenotypes of COPD is associated with short term outcome and ICU mortality following exacerbation.


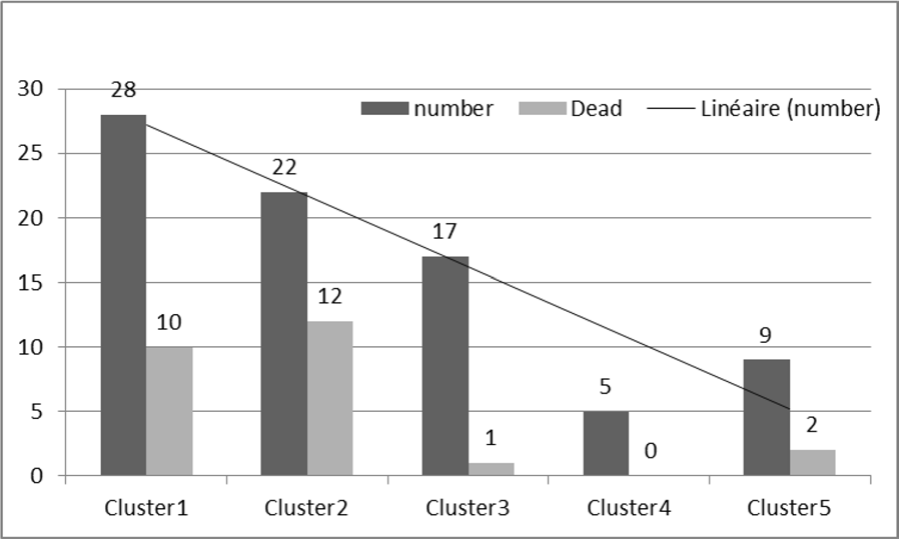


### P-67 What does hypercapnic respiratory failure hide? A prospective ICU-based study

#### Wiem Nouira (*speaker*), Syrine Maatouk, Zeineb Hamouda, Manel Lahmar, Nacef Ben Mrad, Islem Ouanes, Fahmi Dachraoui, Fekri Abroug, Lamia Besbes

##### CHU Fattouma Bourguiba Monastir, Monastir, TUNISIA

###### **Correspondence:** Wiem Nouira - wiemnouira1@gmail.com

*Annals of Intensive Care* 2019, **9(Suppl 1)**:P-67

**Introduction**: Hypercapnic respiratory failure is a frequent cause of admission in the ICU. Several kinds of lung diseases are designated under this nosological framework. Most of these carry prognostic information with a potential impact on the clinical decisions that could be made during the management of these patients. The aim of the study is to assess the frequency of patients admitted for hypercapnic respiratory failure without prior pulmonary diagnosis, and assign a final diagnosis with emphasis on morbid overlaps.

**Patients and methods**: In consecutive patients admitted in the ICU for hypercapnic respiratory failure demographic and clinical data pertaining to current and prior hospitalisations were recorded. Patients were managed for the acute episode, and when they were considered for ICU discharge, the following was performed- pulmonary CT scan with particular emphasis on emphysema and fibrosis patterns, spirometry for the diagnosis of obstructive lung disease, transthoracic cardiac echography, and nocturnal polygraphy for the diagnosis of sleep apnea syndrome (AHI ≥ 5).

**Results**: During the study period, 107 patients (mean age 66 ± 6 years, 63% male) were consecutively admitted for severe hypercapnic respiratory failure requiring ventilatory support. NIV was started in 83% patients with a failure rate 20%. Table 1 reports the pulmonary diseases eventually adjudicated at admission, and at ICU discharge.

**Conclusion**: Reliable information on actual lung diseases of patients admitted for hypercapnic respiratory failure is often lacking at ICU admission. Our study highlights the frequency of COPD, bronchiectasis, and obesity-hypoventilation syndrome in these patients. Overlaps are frequently present in these patients with a need for characterization of their evolutionary genius in the short and long term.


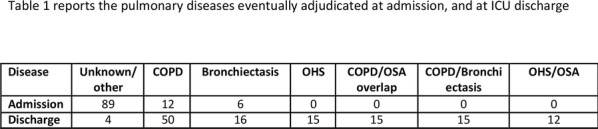


### P-68 Home mechanical ventilation compliance- experience of a low income country MICU

#### Dhouha Ben Braiek (*speaker*), Khaoula Meddeb, Ahmed Khedher, Nesrine Fraj, Said Kortli, Wafa Zarrougui, Abdelbaki Azouzi, Imen Ben Saida, Mohamed Boussarsar

##### Farhat Hached University Hospital, Sousse, TUNISIA

###### **Correspondence:** Dhouha Ben Braiek - bbraiek_dhouha@hotmail.com

*Annals of Intensive Care* 2019, **9(Suppl 1)**:P-68

**Introduction**: Despite the wide spread use of home mechanical ventilation (HMV) to treat chronic hypercapnic respiratory failure, compliance with HMV among patients has seldom been systematically studied. Evaluating outcomes for ICU survivors requiring home mechanical ventilation is also interesting. The aim was to determine patients’ compliance to home mechanical ventilation indicated at discharge and to determine patients’ outcomes and quality of life within three months after discharge.

**Patients and methods**: It is a retros pective observational study conducted in a 9-bed medical ICU. All consecutive patients discharged from ICU with home mechanical ventilation between January, 1st 2015 and of December, 31st 2017 were included. Data collected involved patients’ demographics, past history and underlying diseases, functional state, clinical, paraclinical, therapeutic and ICU stay course characteristics. At discharge, data on types, modes and indications of home mechanical ventilation, were gathered. Compliance reports were obtained from providers after one to three months of use. Vital status and quality of life as assessed by the St. Georges Hospital on Respiratory Problems (SGRQ), were estimated via phone calls at three months after discharge.

**Results**: Among a to tal of 717 ICU admitted patients, 635 required mechanical ventilation. 66(10.4%) patients were discharged with HMV, 38(57.6%) via non-invasive ventilation and 28(42.4%) on tracheostomy (TPPV). They were 61.5 ± 14.4 mean age. They had predominantly chronic respiratory failure, 60(91%) + 45(68.2%), COPD 35(58.3%), mMRC score at III and IV + 18(27.3%), already on HMV, 34(51.5%) patients had at least two comorbidities with Charlson comorbidity index at 2[1–4]. On admission, mean SAPSII, 26 ± 11 with a median ICU stay at 15[10–27] days. After discharge, compliance reports were obtained, only for 20(26%) patients. Respectively, mean percentage daily use of home NIV and TPPV (≥ 4 h per day) were respectively, 92 ± 11% and 96 ± 6%. Twelve patients (18.2%) were readmitted. Mortality at three months was estimated at 19(28.8%). Health related quality of life assessed by SGQR showed a significant impairment, mean total score, 47 ± 7 + (symptoms score, 27 ± 16 + activities score, 44 ± 18 + impacts score, 52 ± 8) compared to population norms (total score, 12 + symptoms score, 16 + activity score, 16 + impacts score, 8).

**Conclusion**: The present study shows rather poor vital and functional outcomes at three months after discharge with HMV albeit a satisfying HMV compliance.

### P-69 Asynchronies using a simulator of artificial ventilation (SimVA) in virtual COPD patients, effects of reducing pressure support or increasing expiratory trigger

#### Hadrien Roze (*speaker*)^1^, Remi Dubois^2^

##### ^1^CHU de Bordeaux, Bordeaux, FRANCE; ^2^IHU Liryc, Fondation Bordeaux Université, Bordeaux, FRANCE

###### **Correspondence:** Hadrien Roze - hadrien.roze@chu-bordeaux.fr

*Annals of Intensive Care* 2019, **9(Suppl 1)**:P-69

**Introduction**: Simulation in intensive care is an innovative method for teaching. Respiratory settings can be responsible for some asynchronies, which may increase mortality of our patients (1). For this reason we develop a simulator of spontaneous artificial ventilation (SimVA) and virtual breathing patients. Mathematical model resolved differential equations of chest and lung movements according to inspiratory effort in order to match with a clinical database. The goal of this study was to evaluate asynchrony index (AI) in virtual COPD patients according to pressure support (PS) level and Inspiratory time (Ti) and to compare the results to the study of Thille et al. (2).

**Patients and methods**: Virtual case were COPD, defined by thoracic and pulmonar compliance, total resistance, lung volumes, and inspiratory adaptive muscle pressure. Asynchrony Index was patient ineffective efforts (IE) (IE +Ventilator Respiratory Rate). Ventilatory protocols were Baseline-PS, Optimal-PS and Optimal-Ti (Optimal meant decreasing PS or Ti in order to reduce AI) as described by Thille et al. (2). Each virtual case was titrated with each protocol. AI was recorded and compared to the results of Thille et al.

**Results**: The optimal protocols titrated PS or Ti in order to reduce AI, the software simulates the corresponding values of tidal volume and respiratory frequency and its effect on intrinsic PEEP and gas trapping. The difference in settings and respiratory mechanic between virtual cases and patients were not significant (Table).

**Conclusion**: AI was able to change according to PS or Ti settings within the same range as the study from Thille et al. Simulation with the software SimVA is realistic and may help to teach interactively ventilatory settings and asynchronies in COPD patients under Pressure Support Ventilation anywhere without any risk for the patient.

**References**

1. Intensive Care Med. 2015 + 41-633-41.
2. Intensive Care Med. 2008 + 34-14773-1486.

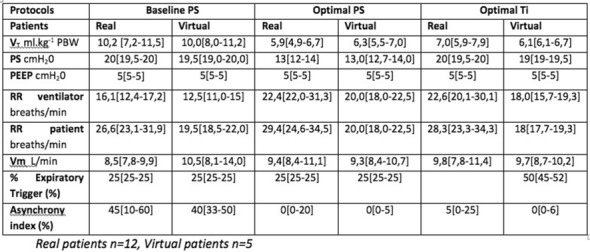


### P-70 Incidence of pulmonary embolism and its impact on the outcomes of chronic obstructive pulmonary disease

#### Hela Kallel (*speaker*), Bouchaala Karama, Triki Amal, Bradii Sabrine, OlfaTurki, Mabrouk Bahloul, Mounir Bouaziz

##### CHU Habib Bourguiba, Service de Reanimation medicale, Sfax, TUNISIA

###### **Correspondence:** Hela Kallel - hela.kallel.fourati@gmail.com

*Annals of Intensive Care* 2019, **9(Suppl 1)**:P-70

**Introduction**: We aimed to determine the incidence and the prognostic impact [mortality and length of intensive care unit (ICU) stay (LOS)] of pulmonary embolism (PE) in critically ill patients with severe acute exacerbation of chronic obstructive pulmonary disease (COPD).

**Patients and methods**: This is a retrospective study performed during a 10-year period in the ICU of Habib Bourguiba University Hospital (Sfax, Tunisia). All patients with severe acute exacerbation of COPD were included. The diagnosis of PE is confirmed by spiral computed tomography scan showing one or more filling defects or obstruction in the pulmonary artery or its branches.

**Results**: During the study period, 169 patients with acute exacerbation of COPD were admitted in our ICU. The mean age (± standard deviation) was 67.99 ± 9.2 years. During their ICU stay, 28 patients (17%) developed PE. The diagnosis was confirmed within 48 h from ICU admission in all cases. The comparison between the two groups (with and without PE) showed that they had the same baseline characteristics. However, most of patients on PE group developed shock (71.4%) on ICU admission or during ICU stay. ICU mortality was significantly higher in the PE group (67.9% vs 44.5%, P = 0.024). In addition, the ICU LOS was significantly higher in the PE group than the PE-free group (P = 0.005).

**Conclusion**: Our study showed that PE is common in patients with severe COPD exacerbation requiring ICU admission. Moreover, PE was significantly associated with higher mortality and ICU LOS in critically ill patients with severe COPD exacerbation.

### P-71 Long-term survival of ICU patients surviving hypercapnic respiratory failure- impact of home ventilation

#### Syrine Maatouk (*speaker*), Manel Lahmar, Zeineb Hamouda, Islem Ouanes, Fahmi Dachraoui, Wiem Nouira, Fekri Abroug, Lamia Besbes

##### CHU Fattouma Bourguiba Monastir, Monastir, TUNISIA

###### **Correspondence:** Syrine Maatouk - syrinemaatouk@gmail.com

*Annals of Intensive Care* 2019, **9(Suppl 1)**:P-71

**Introduction**: The impact of home ventilation on survival of patients with chronic respiratory failure is still debated. Most studies that included patients with chronic hypercapnia away from hospitalisation, showed a positive effect on the frequency of exacerbation. The impact of home ventilation on survival in patients discharged from the ICU is not well proven. The current study reports long term survival according to the use of home ventilation.

**Patients and methods**: All patients surviving their ICU stay for hypercapnic respiratory failure were considered for home ventilation on the following basis- frequent exacerbators under long-term oxygenotherapy in obstructive lung disease, and signs of nocturnal hypoventilation in restrictive lung disease. Vital status was checked in January 2018 and Kaplan–Meier survival curve was drawn (with Log Rank statistical test).

**Results**: During the study-period 229 patients fulfilled the inclusion criteria and were included in the study. The mean age was 67 ± 11 years and 70% were male the majority (74%) had COPD. Home ventilation was administered to 176 patients while the remaining 53 did not. At a mean follow-up of 50 ± 24 months, patients under home ventilation had significantly longer survival than patients without home ventilation (Log Rank < 0.05), Figure.

**Conclusion**: Home ventilation is associated with prolonged survival in ICU patients surviving hypercapnic respiratory failure.


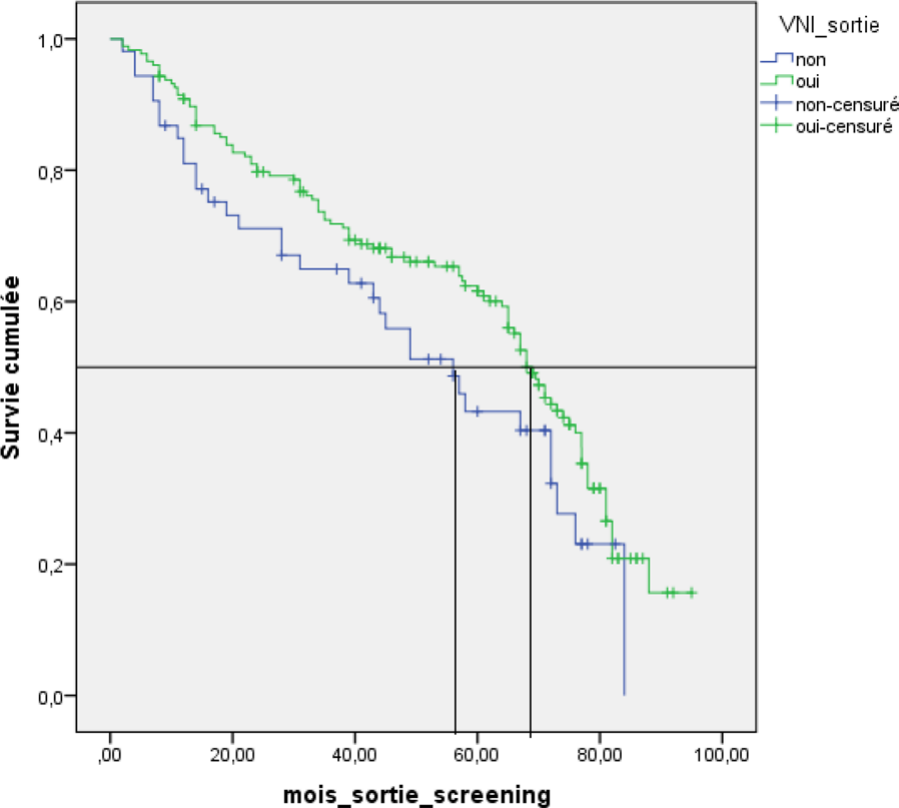


### P-72 The fragility and reliability of conclusions of anesthesia and critical care randomized trials with statistically significant findings- A systematic review

#### François Grolleau (*speaker*)^1^, Gary S. Collins^2^, Romain Pirracchio^3^, Clément Gakuba^1^, Isabelle Boutron^4^, P. J. Devereaux^5^, Yannick Le Manach^6^

##### ^1^Department of Anesthesiology and Critical Care Medicine, Caen, FRANCE; ^2^Centre for Statistics in Medicine, Nuffield Department of Orthopaedics, Rheumatology and Musculoskeletal Sciences, Botnar Research Centre, University, Oxford, UNITED-KINGDOM; ^3^Department of Anesthesiology and Perioperative Care, San Francisco, UNITED STATES; ^4^Centre de Recherche Epidémiologie et Statistique, INSERM U1153, Paris, FRANCE; ^5^Departments of Medicine & Health Evidence and Impact, Michael DeGroote School of Medicine, Faculty of Health Sciences, Hamilton, CANADA; ^6^Departments of Anesthesia & Clinical Epidemiology and Biostatistics, Michael DeGroote School of Medicine, Faculty of Health Sciences, Hamilton, CANADA

###### **Correspondence:** François Grolleau - fc.grolleau@gmail.com

*Annals of Intensive Care* 2019, **9(Suppl 1)**:P-72

**Introduction**: The Fragility Index (FI), which represents the number of patients responsible for a statistically significant finding, has been suggested as an aid for interpreting the robustness of results from clinical trials. A small FI indicates that the statistical significance of a trial depends on only a few events. Our objectives were to calculate the FI of statistically significant results from randomized controlled trials (RCT) of anesthesia and critical care interventions and to determine the frequency of distorted presentation of results or ‘spin’.

**Patients and methods**: We systematically searched MEDLINE from 01 January 2007 to 22 February 2017 to identify RCTs exploring the effect of critical care medicine or anesthesia interventions. Studies were included if they randomized patients 1–1 into two parallel arms and reported at least one statistically significant (P < 0.05) binary outcome (primary or secondary). Two reviewers independently assessed eligibility and extracted data. The FI was determined for the chosen outcome. We assessed the level of spin in negative trials and the presence of recommendations for clinical practice in positive trials.

**Results**: We identified 166 eligible RCTs with a median sample size of 207 patients (interquartile range [IQR] 109 to 497). The median FI was 3 (IQR 1–7), which means that adding three events to one of the trials treatment arms eliminated its statistical significance. Further, 21 (13%) trials had a FI of zero as the statistically significant outcome was found non-significant when recalculating the P-value using a 2-sided Fisher exact test. High spin was identified in 42% (n = 30) of negative RCTs while 21% (n = 20) of positive RCTs provided recommendations. Lower levels of spin and recommendations were associated with publication in journals with high impact factors (P < 0.001 for both).

**Conclusion**: Statistically significant results in anesthesia and critical care RCTs are often fragile, and study conclusions are frequently affected by spin. Routine calculation of the FI in medical literature may allow for better understanding of trials and therefore enhance the quality of reporting.


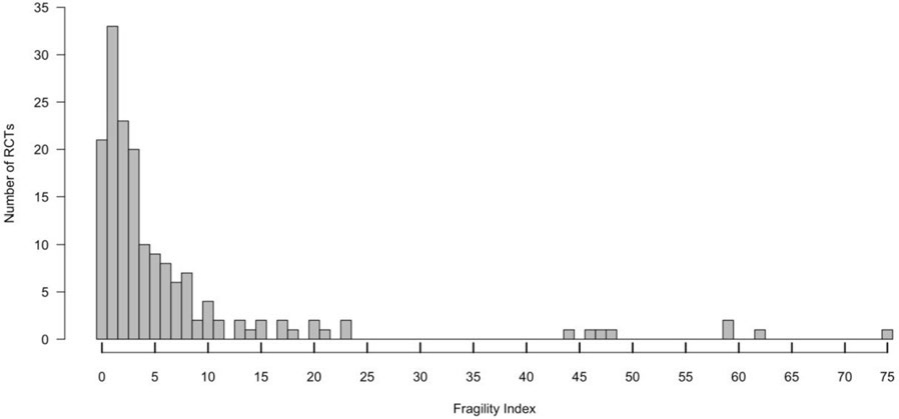


### P-73 Limitation of life-support therapy in Tunisian ICUs- A prospective survey

#### Mariem Tlili (*speaker*), Zeineb Hamouda, Islem Ouanes, Fahmi Dachraoui, Lamia Ouanes-Besbes, Fekri Abroug

##### CHU F.Bourguiba, Monastir, TUNISIA

###### **Correspondence:** Mariem Tlili - tlili.meriem92@gmail.com

*Annals of Intensive Care* 2019, **9(Suppl 1)**:P-73

**Introduction**: The risk of admitting to the ICU patients at end of life has increased with ageing of the population. Yet, it is usually not easy to discern a priori the hospitalization which presages a fatal outcome from that with a significant potential for recovery. Limitation of life-supporting therapy (LLST) has become of current practice in the ICU, but many countries (including Tunisia) lack legal framework for such practice. The aim of the study is to evaluate the magnitude of LLST among intensivists practicing in different healthcare facilities.

**Patients and methods**: A questionnaire was addressed to ICU physicians practicing in university and non-university hospitals in Tunisia. They were invited to participate and respond anonymously to a questionnaire on the limitation and active cessation of care. The questionnaire included description of participants and their workplace characteristics, the frequency of end of life situation in their daily practice, and insights on the way they handle these situations with or without external help from other health workers.

**Results**: 101 intensivists (46% having more than 10 years of experience) practicing principally in ICUs belonging to university hospitals (91%), answered the questionnaire. 69 out of 101 participants found that limitation of life-support therapy was ethically acceptable but active shortening of the dying process was not. The majority were more comfortable with withholding than withdrawing life-supporting therapy. 72 declared they practiced LLST themselves while an additional (22%) said that they were aware of such practice in their ICU. The estimated ICU deaths preceded by LLST were around 20%. A formal written LLST policy was present in 9%, unwritten policy in 52% while 39% declared they did not have any policy. More than one senior ICU doctor usually take part in decision-making regarding LLST in 92%, a single senior ICU doctor in 6%, ICU nurse (s) caring for the patient in 15%. 70% do consider requests for limitation of life-sustaining therapy from patients, families or surrogates and 25% are rarely comfortable when talking about LLST with the family. 54% strongly agreed on the principle of giving analgesics painkillers with LLST to ensure the absence of pain during terminal stages, even if death may be hastened by their use. 69% regret the lack of ethics consultations or committees.

**Conclusion**: Limitation of life-supporting therapy is a frequent practice in Tunisian ICUs although a formal legislative or academic frame is lacking. Intensivists claim a legal framework for this practice.

### P-74 Impact of decisions to forgo life-supporting-therapy on survival in patients admitted to intensive care units for infection

#### Juliette Perche (*speaker*)^1^, Julien Goutay^2^, Aurélia Toussaint^2^, Arthur Durand^2^, ThierryOnimus^2^, Raphael Favory^2^, Sebastien Preau^2^

##### ^1^CH Roubaix, Roubaix, FRANCE; ^2^CHRU Lille, Lille, FRANCE

###### **Correspondence:** Juliette Perche - julietteperche@yahoo.ca

*Annals of Intensive Care* 2019, **9(Suppl 1)**:P-74

**Introduction**: To evaluate the impact of decisions to forgo life-supporting-therapy (DFLST) on survival in patients admitted in intensive care unit (ICU) for an infection.

**Patients and methods**: It was a retrospective, monocentric, observational study, conducted in 2015, in a medical ICU. We included all patients admitted for infection and classified them according to the SEPSIS-3 classification. DFLST were separated into two groups- withholding and withdrawal life-supporting-therapy. We performed logistic regression to identify predictive factors of hospital mortality.

**Results**: On the 444 patients who were included, 124 (28%) had DFLST. Global mortality was 31%. Predictive factors of hospital mortality were on multivariate analysis- DFLST (Odds Ratio (OR) - 42.3, Interval Confidence (IC) 95% [41.50 +43.05], p < 0.01), admission Sepsis-related Organ Failure Assessment (SOFA) score (OR - 1.1, IC 95% [1.05 +1.24], p = 0.04), admission lactatemia (OR - 1.2 IC 95% [1.06 +1.31], p = 0.05), moderate to severe chronic kidney disease (OR - 5.9, IC 95% [4.74 +7.09], p = 0.03), chronic steroids (OR - 3.3, IC 95% [2.25 +4.43] p = 0.02), and chronic hepatopathy (OR - 6.7, IC 95% [5.10 +8.37], p = 0.02). According to the subtype of DFLST (withholding or withdrawal), and the subtype of infection (sepsis or septic shock), there were significative statistical differences on hospital mortality- for sepsis, 17 (8%) patients without DFLST, 22 (55%) patients with withholding, and 30 (100%) patients with withdrawal, p < 0.05 + for septic shock, 20 (23%) patients without DFLST, 14 (73%) patients with withholding, and 32 (100%) patients with withdrawal, p < 0.05 (Figure 1). Early predictive factors of DFLST were in multivariate analysis- age (OR - 1.03 IC 95% [1.01 +1.05], p < 0.01), metastatic cancer (OR 5.2 IC 95% [4.12 +6.12], p < 0.01), respiratory (OR 1.3 IC 95% [1.05 +1.49], p = 0.03), renal (OR 1.3 IC 95% [1.16 +1.54], p < 0.01) and neurologic (OR 1.6 IC 95% [1.32 +1.80], p < 0.01) items of initial SOFA score, and fungal infection (OR 7.6 IC 95% [6.16 +9.14], p < 0.01).

**Conclusion**: DFLST are an independent factor of mortality in patients with sepsis and septic shock in ICU. This result may influence our clinical practice and show up the necessity to collect DFLST in studies.


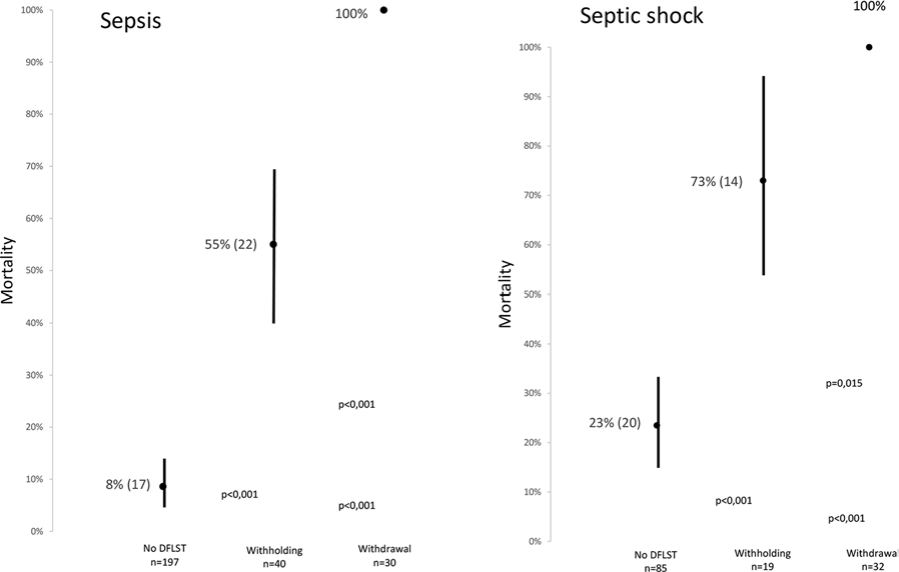


### P-75 Collegial procedures for therapeutic limitations in a French ICU- The perception of the participants

#### Jacques Boutros (*speaker*)^1^, Julien Charpentier^1^, Jean-Paul Mira^1^, Olivier Lesieur^2^

##### ^1^Department of Medical Intensive Care, Cochin University Hospital, Paris, France, Paris, FRANCE;^2^Intensive Care Unit, Saint Louis General Hospital, La Rochelle, FRANCE

###### **Correspondence:** Jacques Boutros - jacques.boutros@gmail.com

*Annals of Intensive Care* 2019, **9(Suppl 1)**:P-75

**Introduction**: Since 2005, the medical decision to withhold or withdraw treatments in a patient who is unable to express his wishes has been legally framed by the Leonetti law- such a resolution can only be adopted after a so-called collegial reflection procedure. Regarding the deliberation that precedes the decision, the medical societies and ethical reflection committees have issued recommendations in terms of standardization, training and transparency. Our study aims to evaluate the deliberative members’ perception of the collegial procedures implemented in a French medical intensive care unit. The main objective of our survey was to assess the role of collegial procedure within the decision-making process, with a core issue- is deliberation perceived as the approval of a decision already taken or as an ethical discussion that lies at the heart of the decision-making?

**Patients and methods**: The perception of nurses, nurse aids, residents and doctors was assessed using a five-section questionnaire with open-ended and multiple answer questions. Characteristics of patient admitted to the ICU in 2017, as well as those of the collegial procedures undertaken during the same period, were collected.

**Results**: Of the 230 formal collegial procedures (14.6% of patients admitted) registered in 2017, over 90% were attended by at least a resident, a nurse and a doctor. The analysis of the 47 questionnaires filled in by 5 doctors, 6 residents, 29 nurses and 10 nurse aids shows global satisfaction regarding the organization, duration, exchanges and consideration of the participants’ opinions. While nurse aids are often absent from the deliberations and show moderate implication in the decision making, their contribution on very specific aspects of individual care is desired by other healthcare professionals. Caregivers also deplore the limited involvement of an external consultant whose role would be to provide neutral expertise on the patient’s conditions. Based on the examination of responses to the open-ended questions, it seems necessary to reposition the collegial procedure within a complex deliberative process which gradually leads, throughout the hospital stay, to contingent and revocable decisions of foregoing certain treatments deemed useless, disproportionate or having no other object than the artificial preservation of life.

**Conclusion**: Despite a limited number of participants, this single-center study suggests several refinements and improvements in our collegial reflection procedure in terms of legal and ethical knowledge, organization, and attendance.

### P-76 Organ donation and transplantation- a large survey of knowledge and perceptions among high school students

#### Khalid Khaleq (*speaker*), Reda Hafiane, Imane Talhi, Z Sgheir

##### CHU IBN ROCHD MPFC, Casablanca, MOROCCO

###### **Correspondence:** Khalid Khaleq - khaleq20@gmx.fr

*Annals of Intensive Care* 2019, **9(Suppl 1)**:P-76

**Introduction**: Organ donation and transplantation remain below requirements in Morocco. Each year we see the number of patients needing organ transplantation (OD) getting bigger. We are facing a real shortage in OD. Moroccan students, who represent the young generation, could be the key for a future improvement in organ transplantation. We lead a study in order to determine their knowledge and aspirations concerning this life saving therapy.

**Patients and methods**: It’s a descriptive prospective study- a survey lead in eight higher education institutions. We used a pre-established questionnaire. We analyzed 4 main themes- knowledge assessment, opinions towards OD, its causes and ways of improving it in our country. Data was analyzed using SPSS 20.0 software.

**Results**: We questioned 991 students- 97, 2% had already heard about OD, and 69.9% were aware that it was possible to practice it in Morocco. 80.3% had an idea about lethal diseases requiring the use of transplantation and 75% were able to identify transplantable organs and tissues, 43.5% underestimated the number of people waiting for transplants, 57.6% thought that the acts of organs removal and transplantation are done in private clinics. 62.8% were aware of the law governing OD in Morocco, only 31.4% had trust in this law and 91.3% ignored the registration steps in the donation acceptance register. 71.4% were convinced of the perfect compatibility of OD with Muslim religion, the majority of students (92.5%) were in favor of OD. Among the group of respondents refusing the donation of their organs, the right of refusal and religious obstruction were at the top of the list of the determinants of the refusal with respective prevalence of 47.10% and 24.3%, and only 23.3% of the students had expressed their opinion to their relatives.

**Conclusion**: This study shows that young Moroccans are supportive of OD despite their limited knowledge of the subject. The development of this therapy must go through information and regular motivation of people. General conferences and seminars represent a solution in spreading awareness among generations in order to establish a strong policy of organ donation in an early future.

### P-77 Concept of encephalic death and organ donation to consultants in a Moroccan non university hospital center

#### Hanane Ezzouine (*speaker*)^1^, Karim Mediouni^2^, Soukaina Benyamna^2^, Amine Raja^2^, Abdellatif Benslama^2^

##### ^1^Faculté de médecine -Université Hassan II-Casablanca, Casablanca, MOROCCO; ^2^University Hassan II, Faculte of Medecine ans Pharmacy, Casablanca, MOROCCO

###### **Correspondence:** Hanane Ezzouine - ezzouinehanane@yahoo.fr

*Annals of Intensive Care* 2019, **9(Suppl 1)**:P-77

**Introduction**: Encephalic death and organ donation are widely debated.The promotion of organ donation including a brain dead donor requires us to explore the concept among our population.We aimed to evaluate the knowledge and attitude of consultants in a non-university hospital center concerning organ donation and the concept of brain death.

**Patients and methods**: We carried out a descriptive study for one year in a Moroccan non-university hospital center. The target population was the consultants of this hospital center. A questionnaire was filled out anonymously and aimed to evaluate organ donation, knowledge of the concept of brain death and organ transplantation in brain-dead patients, the wearing of a voluntary organ donor card, the discussion of donations of family organs, family consent in case of cadaveric donor, receiving organs from a living donor and donation of organs and religion.

**Results**: 746 people were included. Their average age is 21.30 ± 9.2 years with male predominance. The values associated with organ donation are for 38.49% of saving lives for 37.57% a gesture of charity, 12.17% a gesture of altruism and 11.77% a duty.85.81% do not know what it is an encephalic death .70.37% do not know that there were donations of organs of patients in a state of brain death .34.26% know it there are contraindications to the organ donation of patients in a state of encephalic death .55.95% do not agree to donate one of their organs during their lifetime.57.69%are for organ donation for the purpose of treating patients and 27.45% are without opinion. 71.16%. refuse to carry a voluntary organ donor card. 86.24% agree to receive an organ from a living donor and 12.42% to receive an organ from a deceased donor .88.22% have never reported their positions to their loved ones. 73.94% are in favor of organ donation after their death .45.50% replied that the Muslim religion allows living organ donation to live and 21.72% replied that it authorizes the donation of organs from living to organs of a patient in a state of encephalic death to a living.

**Conclusion**: A clear disparity is found in the conception of brain death and organ donation despite information campaigns and media coverage. The population studied has socio-demographic and cultural characteristics that require adapting the information tools with a cultural approach.

### P-78 Investigation of the time-course of the serotoninergic syndrome in relation to the plasma MDMA concentrations in the severely MDMA-poisoned patients

#### Bruno Megarbane (*speaker*), Mathieu Bouthemy

##### Department of Medical and Toxicological Critical Care, Lariboisière Hospital, Paris, FRANCE

###### **Correspondence:** Bruno Megarbane - bruno.megarbane@lrb.aphp.fr

*Annals of Intensive Care* 2019, **9(Suppl 1)**:P-78

**Introduction**: The recreational use of 3.4-methylenedioxymethamphetamine (MDMA) has become common since the end of the 90 s and the number of poisonings has significantly increased during these last years. MDMA is responsible for serotoninergic toxicity leading to the onset of hyperthermia and multiorgan failure. Our objectives were to describe the time course of body temperature, serotoninergic signs and biological parameters in the severely MDMA-poisoned patients in order to understand the evolution of these parameters in relation to the plasma concentration of MDMA and its main metabolite, methylenedioxyamphetamine (MDA).

**Patients and methods**: We conducted a retrospective single-centre observational study including all MDMA-poisoned patients admitted to the intensive care unit (ICU) and who developed fever (body temperature > 38.5 °C) and symptoms signs of serotoninergic syndrome (according to Sternbach’s criteria). Plasma MDMA and MDA concentrations were determined using liquid chromatography coupled to mass spectrometry.

**Results**: Sixteen MDMA-poisoned patients (out of 58 MDMA-exposed patients admitted to the ICU over a 10-year period) who presented serotoninergic syndrome with fever (> 38.5 °C) attributed to MDMA exposure and treated with supportive care, external cooling and cyproheptadine (a non-specific serotonin receptor antagonist) were included in this study. The patients were 6 females et 10 males, of 22-year old [20 - 26] (median [percentiles 25 - 75] and had used recreational MDMA and found in a night-club (75%), in the street (19%) or at home (6%). The body temperature reached 39.7 °C [38.8 - 40.9] and the presentation was complicated by cardiac arrest (19%), cardiovascular failure (44%), aspiration pneumonia (56%), hospital-acquired infections (25%) and fatality (6%). In four patients (25%), worsening in the clinical (heart rate, pyramidal syndrome, EEG encephalopathy) and biological parameters (transaminases, prothrombin index, creatinine phosphokinase) included in the serotoninergic syndrome was surprisingly observed in parallel to the increase in body temperature (due to a concomitant infectious or inflammatory non-toxic event). The observed worsening occurred in the absence of any increase in the plasma MDMA and MDA concentrations (N = 2) and even in the presence of undetectable concentrations (N = 2).

**Conclusion**: MDMA use results in serotonin syndrome possibly leading to the onset of hyperthermia, organ failure and death, despite optimal care. Worsening in the serotonin syndrome during the ICU stay of severely MDMA-poisoned patients, without increase in plasma MDMA concentration is observed in 25% of the patients. The exact molecular mechanisms involved in this “serotoninergic memory” remain to be clarified.

### P-79 Carbon monoxide induced coma - prognostic factors

#### Paris Meng (*speaker*)^1^, Ophélie Constant^1^, David Luis^1^, Vivien Hong Tuan Ha^1^, Sivanthiny Sivandamoorthy^1^, Nicholas Heming^1^, Sylvie Chevret ^2^, Djillali Annane^1^

##### ^1^Service de réanimation médico-chirugicale, CHU Raymond Poincaré, APHP, Université de Versailles Saint Quentin en Yvelines, Garchess, FRANCE; ^2^SBIM, CHU Saint Louis, AHPH, Université Paris Diderot, Paris, France, Paris, Paris

###### **Correspondence:** Paris Meng - paris.meng@aphp.fr

*Annals of Intensive Care* 2019, **9(Suppl 1)**:P-79

**Introduction**: Carbon monoxide (CO) is a leading cause of poison related lethality in France. Moreover, survivors may develop severe neuro-cognitive sequelae. Few studies sought to determine prognostic factors related to CO induced coma. The primary objective of our study was to determine prognostic factors for ICU-mortality following CO induced coma. Our secondary objective was to determine prognostic factors of CO related cognitive sequelae, at the time of intensive care unit (ICU) discharge.

**Patients and methods**: Retrospective observational study from January 2000 to December 2012. All comatose patients (Glasgow coma score < 8) due to carbon monoxide poisoning, treated by hyperbaric oxygen therapy in a tertiary hospital in the greater Paris area were included in the current study. Clinical, biological, iconographic and electrophysiological data were collected from medical files.

**Results**: 184 patients were included, median [IQR] age was 42 [30 - 56] years, 105 patients (57.4%) were male. Causes of poisoning were mainly CO exposure n = 55 (30%) and smoke inhalation n = 107 (58.5%). 30 patients (17%) died during their ICU stay while 26 (14.7%) presented cognitive sequelae at ICU discharge. Multivariate analysis found that cardiac arrest (OR 1.28; IC95% [1.14 -1.44] + p < 0.001), EEG anomalies (OR 1.34 + IC95% [1.21–1.49] p < 0.001), and the Simplified Acute Physiology Score (SAPS II) (OR 1.04 + IC95% [1.01–1.07) + p = 0.02) were associated with ICU-mortality. Neuroimaging anomalies (OR 1.53 + IC95% [1.36–1.72] + p < 0.001) were associated with cognitive sequelae at ICU discharge. Neither lactate nor carboxyhemoglobin levels were associated with cognitive sequelae or mortality in our cohort.

**Conclusion**: In our study, predictive factors of ICU-mortality in CO induced comatose patients were a cardiac arrest, EEG anomalies and the SAPS II score. Neuroimaging anomalies were predictive of cognitive sequelae at ICU discharge.

### P-80 Poppers poisoning admitted to the intensive care unit- Is there a methemoglobinemia threshold responsible for tissue dysoxia?

#### Bruno Megarbane (*speaker*), May Yaker

##### Department of Medical and Toxicological Critical Care, Lariboisière Hospital, Paris, FRANCE

###### **Correspondence:** Bruno Megarbane - bruno.megarbane@lrb.aphp.fr

*Annals of Intensive Care* 2019, **9(Suppl 1)**:P-80

**Introduction**: The recreational use of poppers has been increasing in France since the last judgment of the National Council in June 2013 re-authorizing their marketing. Poppers contain various alkyle nitrites which are highly oxidant compounds able to induce methemoglobinemia (MetHb) with deleterious and even life-threatening consequences in humans. Our objective was 1)- to describe the circumstances, complications and outcome of the patients admitted to the intensive care unit (ICU) for MetHb onset attributed to the exposure to poppers and 2)- to investigate the relationship between the serum lactate concentration and the MetHb before treatment.

**Patients and methods**: We conducted a retrospective monocentre observational study including all patients admitted to the ICU with increased blood MetHb (> 0.7%) following the exposure to poppers. We investigated the relationships between SpO2, serum lactate concentration and MetHb on ICU admission by calculating the Pearson’s coefficients and using the Bartlett’s test of sphericity.

**Results**: Twenty-six patients (24 males and 2 females aged of 42 years (35 - 48) [median (percentiles 25 and 75)]) were included. The poppers had been snorted (77%), ingested (19%) or snorted + ingested (4%), in a recreational multi-drug exposure (65% + mostly accompanied by gamma-hydroxybutyrate use). On admission, MetHb was 20.0% (1.5 - 44.0). The patients received methylene blue infusion (62%), mechanical ventilation (35%), catecholamine infusion (12%) and exsanguino-transfusion (4%). On admission, the SpO2 was 91% (83 - 94) and weakly correlated to the MetHb (R2 = 0.3, p = 0.01) while the serum lactate concentration was 2.2 mmol/L (1.3 - 3.9) and highly correlated to the MeHb (R2 = 0.7, p < 0.0001). Plasma lactate concentration of > 2 mmol/L was highly predictive of MetHb > 20%, with 91.7% sensitivity, 90.9%, specificity, 91.7% positive predictive value and 90.9% negative predictive value.

**Conclusion**: The use of poppers is responsible for life-threatening consequences attributed to MetHb. Our data clearly supported the recommendation by the international and French guidelines to administer methylene blue in patients developing MetHb > 20% by evidencing that tissue hypoxia is almost consistently present above this threshold.

### P-81 Hemodynamic profile of acute intoxications

#### Mohamed Anass Fehdi (*speaker*), Amine Raja, Amine Zerhouni, Mohammed Mouhaoui

##### CHU Ibn Rochd, Casablanca, MOROCCO

###### **Correspondence:** Mohamed Anass Fehdi - mohamedanassf@gmail.com

*Annals of Intensive Care* 2019, **9(Suppl 1)**:P-81

**Introduction**: Cardio-circulatory failure is one of the leading causes of death in acute poisoning. The aim of our work was to analyze the hemodynamic profile of intoxications to non-cardiotropic products, and to identify the prognostic factors.

**Patients and methods**: This was a prospective, 1-year study, including any patient over the age of 15 years, in primary admission to the vital emergency room, for non-cardiotropic drug intoxication but also non-drug. The analyzed parameters were- clinical examination, electrical abnormalities, echocardiographic data and troponin level, this evaluation performed with H24 intake. The statistical analysis was univariate with a p < 0.05.

**Results**: 251 patients were included, with an average age of 29, with a _ sex ratio. The toxic products found were organophosphorus (39.1%), benzodiazepines (16.4%), aluminum phosphide (14.7%), cocaine (12.3%), carbon monoxide (8.9%),, 8%) and tricyclic antidepressants (5.2%). Cardiocirculatory insufficiency was observed in 62 patients (24.6%), electrical abnormalities in 45 patients (17.9%), elevation of troponin in 22 patients (8.7%) and echocardiographic abnormalities in 20 patients (7.9%).The incidence was particularly high for aluminum phosphide and tricyclic antidepressants.The overall mortality rate was 9% (36 deaths), depending on the offending product- 45.9% for aluminum phosphide, 30.7% for tricyclic antidepressants, 12.9% for cocaine, 8, 1% for organophosphorus compounds and 4.9% for benzodiazepines. (Figure 1) The correlation between clinical and paraclinical abnormalities and the occurrence of death is shown in Table 1.

**Discussion**: In toxicology, the range of cardio-toxic products is much wider than the class of cardiotropic drugs. Indeed, very many products are at the origin of a cardiovascular toxicity. The mechanism of this failure is variable- membrane stabilizing effect (antidepressants, beta-blockers, calcium channel blockers, organophosphorus), diastolic dysfunction, rhythm disorders, toxic myocarditis, myocardial necrosis (Phostoxin, cocaine, CO) or vasodilatation (antihypertensives).

**Conclusion**: The difficulty of accurately assessing the incidence and prevalence of cardio-circulatory failure due to toxic causes is due, among other things, to the lack of validated studies to propose better diagnostic and therapeutic techniques. In our study, the 2 main factors of poor prognosis were cardiocirculatory insufficiency and echocardiographic abnormalities.


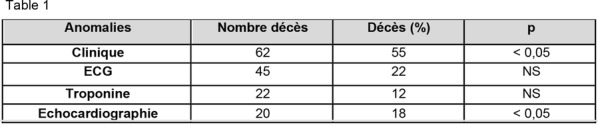



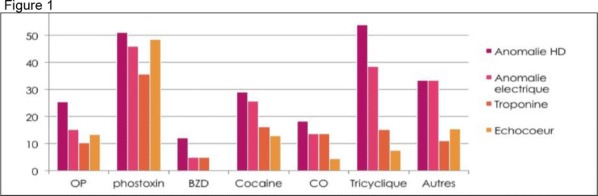



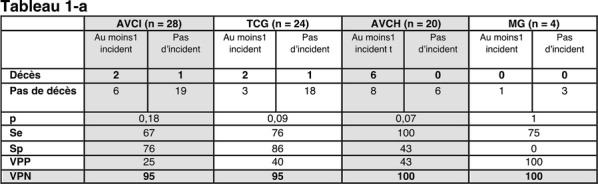



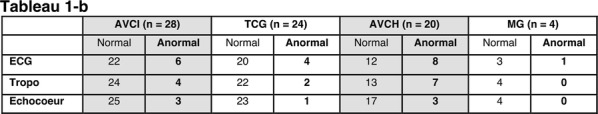



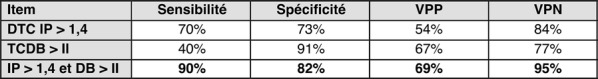


### P-82 Cocaine poisoning in the intensive care unit- are there differences between cocaine hydrochloride- and crack-related toxicity?

#### Bruno Megarbane (*speaker*)^1^, Lisa Catherine^1^, Marion Soichot^2^, Laurence Labat^2^, Nicolas Deye^1^, Isabelle Malissin^1^, Sébastian Voicu^1^

##### ^1^Department of Medical and Toxicological Critical Care, Lariboisière Hospital, Paris, FRANCE; ^2^Toxicology laboratory, Paris, FRANCE

###### **Correspondence:** Bruno Megarbane - bruno.megarbane@lrb.aphp.fr

*Annals of Intensive Care* 2019, **9(Suppl 1)**:P-82

**Introduction**: Cocaine is the most frequently used psychostimulant illicit drug worldwide. In France, its use has significantly increased during the last ten years, with two main modalities- the recreational snorting of cocaine hydrochloride and the inhalation of smoked “crack” (free-base form of cocaine, obtained by cocaine salification using common baking soda or ammoniac) in highly dependent and dissocialized users. The neurological, cardiovascular, respiratory and metabolic complications of cocaine are relatively well-known + however, differences between cocaine hydrochloride and crack-related toxicities have been poorly investigated. Our objectives were to describe and compare the circumstances and the resulting complications of cocaine use in the patients admitted to the intensive care unit (ICU) in relation to the route of exposure, i.e. snorting versus inhalation.

**Patients and methods**: We conducted a retrospective single-centre observational study including all cocaine-poisoned patients admitted to the intensive care unit over an 8-year period (2011–2018). Comparisons between the two routes of cocaine exposure were performed using an univariate analysis (Chi-2 and Mann–Whitney tests, as requested).

**Results**: Seventy-two patients (age, 35 years [30 - 46] (median, [percentiles 25 and 75]) were included. Toxicity mainly resulted from multidrug use [benzodiazepines (79%), ethanol (32%), methadone (29%), tetrahydrocannabinol (23%) and amphetamines (21%)]. The Glasgow coma score on admission was 10 [3-15]. Complications included cardiac arrest (N = 9), aspiration pneumonia (N = 19), acute renal failure (N = 15), malignant hypertension (N = 7), ischemia thrombosis events (N = 5), seizures (N = 5), hyperthermia (N = 5), myocardial infarction (N = 1) and death (N = 5). Users by snorting (N = 34) presented significantly more severe symptoms than users by inhalation (N = 25), with lower Glasgow coma score (p = 0.02), more intense adrenergic syndrome (p = 0.009 for tachycardia), more elevated plasma lactate concentration (p = 0.002), lower platelet count (p < 0.0001) and more marked rhabdomyolysis (p = 0.02). Snorting cocaine was less frequently associated with methadone use (p < 0.0001). Cocaine snorting patients more frequently developed cardiac arrest (p = 0.02), aspiration pneumonia (p = 0.04) and requested mechanical ventilation (p = 0.0002).

**Conclusion**: Cocaine use may lead to severe complications requiring ICU admission. Our findings suggest that complications attributed to cocaine hydrochloride snorting are more severe than the complications attributed to crack inhalation. Experimental investigations may interestingly complement our study to allow better understanding of the mechanistic reasons supporting these observed differences.

### P-83 Bromazepam poisoning in the intensive care unit- usefulness of the plasma bromazepam concentration for patient management

#### Bruno Megarbane (*speaker*)^1^, Sybille Riou^1^, Marion Soichot^2^, Nicolas Péron^1^, Pierre Mora^1^, Isabelle Malissin^1^, Laurence Labat ^2^

##### ^1^Department of Medical and Toxicological Critical Care, Lariboisière Hospital, Paris, FRANCE; ^2^Toxicology laboratory, Paris, FRANCE

###### **Correspondence:** Bruno Megarbane - bruno.megarbane@lrb.aphp.fr

*Annals of Intensive Care* 2019, **9(Suppl 1)**:P-83

**Introduction**: Bromazepam is the most commonly used and the most frequently involved benzodiazepine in acute drug poisonings in France. Our objectives were 1) to report the complications and management of bromazepam-poisoned patients admitted to the ICU and, 2) to investigate bromazepam pharmacokinetics in overdose and the relationships between the coma depth and plasma bromazepam concentration on admission.

**Patients and methods**: We conducted a retrospective single-centre observational study including all bromazepam-poisoned patients admitted in 2011–2018, evidenced by at least one plasma bromazepam concentration in the toxic range during their stay. We studied the correlation between the presumed ingested dose of bromazepam, the plasma concentration of bromazepam and the coma depth determined by the Glasgow coma score by calculating the Pearson’s coefficients and using the Bartlett’s test for sphericity.

**Results**: One-hundred and sixty-four patients [112 females and 52 males age, 51 years (41–63) (median (percentiles 25–75)] were included. Toxicity resulted from multidrug ingestions (75%), with a presumed bromazepam ingested dose of 180 mg (113–180) and plasma bromazepam concentration on admission of 1.88 mg L (0.87–2.70). Consciousness impairment was marked [Glasgow coma score, 9 (3–14)], hypotonic coma (43%) and decrease loss in tendon reflexes (26%)]. Complications included aspiration pneumonia (49%), increase in liver enzyme (79%), cardiovascular (21%) and renal failure (10%). Sino- and atrio-ventricular blocks were found in 17% of the cases. Flumazenil [bolus dose of 0.3 mg (0.2–0.4) followed by infusion rate of 0.4 mg h (0.3–0.6) during 24 h (17–60)] was administered in 28% of the patients while 40% of the patients were intubated and mechanically ventilated. In the subgroup of mono-intoxications with bromazepam, no significant correlation between the coma depth and the plasma bromazepam concentration was observed (R2 = 0.1 + Bartlett’s test, p = 0.3). Factors associated with the requirement of tracheal intubation (vs. flumazenil use) included lower Glasgow coma score (p = 0.002) and more elevated serum lactate concentration (p = 0.03). No significant relationship was evidenced between the ingested dose and the plasma concentration of bromazepam on admission.

**Conclusion**: Acute bromazepam poisoning is frequent and may be responsible for life-threatening consequences. The ingested dose and the plasma concentration of bromazepam are not correlated with the coma depth and do not predict the necessity of tracheal intubation, thus suggesting high inter-individual variability in the drug toxicity and optimal management.

### P-84 Ecstasy an unsafe recreational drug- Experience of 10 years ICU practice

#### Takoua Khzouri (*speaker*), Hela Maamouri, Meriem Fatnassi, Rim Jemmali, Nozha Brahmi

##### Camu, Tunis, TUNISIA

###### **Correspondence:** Takoua Khzouri - takoua_kh2@yahoo.fr

*Annals of Intensive Care* 2019, **9(Suppl 1)**:P-84

**Introduction**: 3.4-Methylenedioxymethamphetamine (MDMA), also known as Ecstasy, is a recreational drug, popular among youth in Europe and America since the 1990s. During the last decade, there has been a remarquable increase in cases of MDMA poisoning admitted in our ICU. The present study aimed to better know the epidemiological, clinical and therapeutic characteristics of this poisoning in order to improve its prognosis.

**Patients and methods**: It was an observational retrospective study spread over nine years from 1st January 2010 to 22th September 2018 in a toxicological ICU, including all patients admitted for acute MDMA poisoning.

**Results**: During the study period, MDMA poisoning accounted for 0.15% (n = 14) of all the acute poisonings requiring hospitalization in our Intensive Care Unit. Most of them were between 2016 and 2018. Their mean age was of 20 years [15, 30], with a sex-ratio of 6. Eight patients (57%) were drug-addicted. Exposures were single-drug in 4 cases (28.6%). In the other cases, Ecstasy was co-ingested with alcohol in 6 cases, benzodiazepine in 2 cases, trihexyphenidyl in 2 cases and both cannabis and heroin in one case. The supposed ingested dose (SID) was unknown in 4 cases, for the other ones, the median SID was 1 pill and the half [1, 4]. The consultation delay was of 6 ± 3 h after ingestion. The main symptoms were mydriasis (79%), agitation (71.4%), tachycardia (64.3%) with an average of 114 bpm [102, 145], hallucinations (57%) and hypertension (57%). The five coma patients were intubated and required sedation with midazolam in 60%. Only one patient required curare for malignant hyperthermia at42°c. Eleven patients developed rhabdomyolysis with average rates of creatine phosphokinase and lactate deshydrogenase respectively of 9479 UI [326, 54000] and 680 UI [144, 2825]. Two patients developed disseminated intravascular coagulation and fulminant hepatitis with high rates of alanine aminotransferase (4631 UI and 2223 UI), aspartate aminotransferase (3695UI and 1876 UI) and low factorV (5% and 60%] for which they received N-acetylcysteine. One patient received cyproheptadine as antidote at 12 mg a day. Thirteen patients were discharged from the ICU with a mean length of stay of three days. One patient died of hepatic encephalopathy with cerebral herniation.

**Conclusion**: As it was shown, the MDMA poisoning presents a vital risk due to the serotonin syndrome. The clinician must be warned to recognize better its symptoms and its severity in order to improve its management.

### P-85 Important changes in clinical presentation and outcomes of patients treated for severe malaria in a referral French university hospital from 2004 to 2017

#### Jordane Lebut (*speaker*), Bruno Mourvillier, Camille Vinclair, Radj Cally, Aguila Radjou, Claire Dupuis, Nicolas Argy , Stéphane Ruckly, Romain Sonneville, Mathilde Neuville, Michel Wolff, Lila Bouadma, Jean-François Timsit

##### Bichat-Claude Bernard Hospital, Paris, FRANCE

###### **Correspondence:** Jordane Lebut - jordane.lebut@hotmail.fr

*Annals of Intensive Care* 2019, **9(Suppl 1)**:P-85

**Introduction**: Incidence of imported severe malaria (SM) increased and patients characteristics and prognosis changed since early 2000. Our objective was to analyze changes in clinical presentation and outcomes since artesunate became first-choice treatment of SM.

**Patients and methods**: Retrospective observational single-center study in the medical ICU of a referral university hospital conducting on patients admitted for SM over a 14-year period (2004–2017). Demographic variables, severity scores, WHOs severity criteria on admission, ICU and hospital lengths of stay were collected. Patients’ characteristics and outcomes were compared between two periods, namely 2004–2012 and 2013–2017 when artesunate has become first-choice treatment. A poor outcome was defined as the composite endpoint of death, or ICU length of stay > 2 days, or requirement for vasopressors, invasive mechanical ventilation and or renal replacement therapy started after the first day in ICU. Univariate analysis and stepwise multivariate logistic regression stratified by period were performed to identify factors associated with a poor outcome.

**Results**: 189 patients were included, 98 before and 91 after 2013. Main epidemiological and clinical characteristics are on Table. Even if the number of WHO criteria for SM was comparable in both groups, SAPS II, SOFA and ICU length of stay were significantly higher before 2013. Patients visiting friends or relatives (VFR) in their home country or living in endemic areas seemed more frequent after 2013 (p = 0.07).

Poor outcome occurred in 63 cases before 2013 and 32 cases after 2013 (p < .01). Risk factors of poor outcome were impaired consciousness (adjOR = 3.25, 95%CI (1.50–7.07), p = 0.003), shock (adjOR = 3.46, 95%CI (1.36–8.85), p = 0.01) and creatinine > 265 µmol L (adjOR = 14.16, 95%CI (4.95–40.48), p < .001). Patients VFR or living in endemic areas were associated with a better outcome (adjOR = 0.34, 95%CI (0.15–0.77), p = 0.01). In the final model, artesunate therapy did not significantly improve the outcome as compared to quinine-based therapy (adjOR = 0.53, 95%CI (0.16–1.71), p = 0.28).

**Conclusion**: Patients with SM admitted in our ICU after 2013 were less severe than those before 2013. These trends could be partially explained by the increasing proportion of immune patients VFR or living in endemic areas. After adjustment on severity and stratification by period, artesunate was not significantly associated with a better outcome than IV quinine.


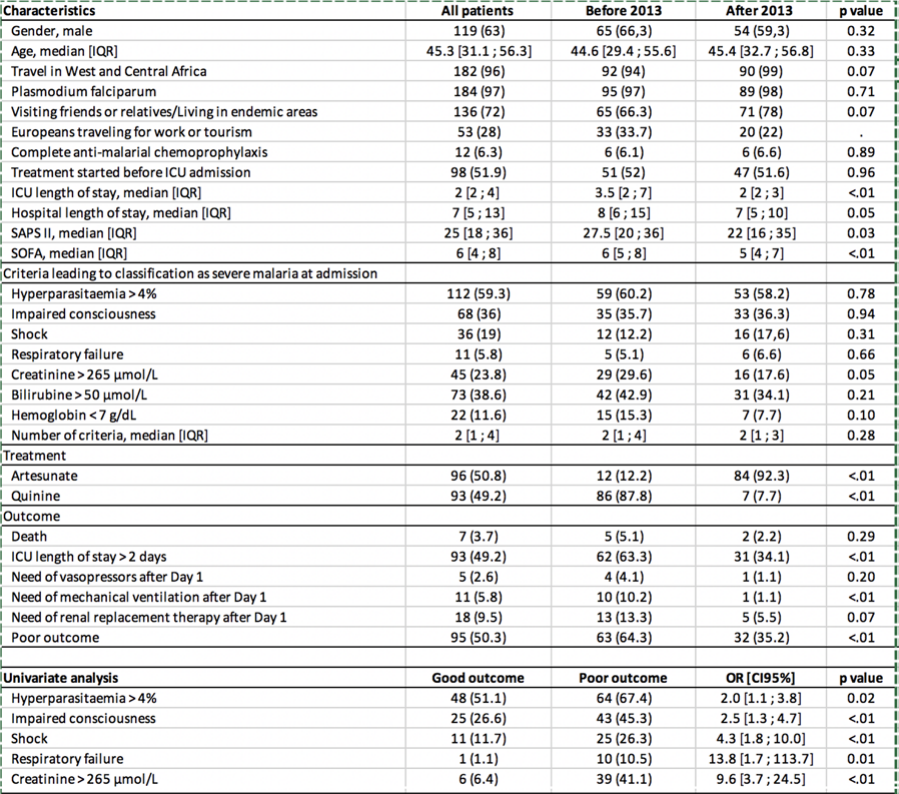


### P-86 Clostridium bacteriemia in critically ill patients

#### Guillaume Morel (*speaker*)^1^, Etienne Ghrenassia^1^, Moustafa Abdel Nabey^1^, Julien Mayaux^2^, Naike Bige^3^, Guillaume Dumas^3^, Amélie Seguin^4^, Guillaume Voiriot^5^, Bruno Megarbane^6^, Frédéric Pene^7^,Yacine Tandjaoui Lambiotte^8^, Anne-Sophie Moreau^9^, Frédéric Wallet^9^, Djamel Mokart^10^, Muriel Picard^11^, David Rousset^11^, Elie Azoulay^1^, Lara Zafrani^1^

##### ^1^CHU Saint Louis, Paris, FRANCE; ^2^CHU Pitié Salpétrière, Paris, FRANCE; ^3^CHU Saint Antoine, Paris, FRANCE; ^4^CHU, Nantes, Nantes; ^5^CHU Tenon, Paris, FRANCE; ^6^CHU Lariboisière, Paris, FRANCE; ^7^CHU Cochin, Paris, FRANCE; ^8^CHU Avicenne, Bobigny, FRANCE; ^9^CHRU Lille, Lille, FRANCE; ^10^Insititut Paoli-Calmettes, Marseille, FRANCE; ^11^Hopital de Purpan, Toulouse, FRANCE

###### **Correspondence:** Guillaume Morel - morel_guillaume@hotmail.fr

*Annals of Intensive Care* 2019, **9(Suppl 1)**:P-86

**Introduction**: Clostridium species are important agents of anaerobic infections that may be associated with a wide range of severe clinical diseases requiring ICU admission. Data focusing on non difficile Clostridium species bacteriemia in ICU are scarce and rely mainly on case reports. We sought to define the clinical and biological characteristics, risk factors for acquisition, Clostridium ecology and outcomes of non difficile Clostridium species bacteriemia in critically ill patients.

**Patients and methods**: This is a multi-centric retrospective cohort study (from 6 ICUs) including all patients diagnosed with non-difficile clostridium bacteriemia between January 2002 and June 2018.

**Results**: Fifty patients were included. Median age was 65 (53–80) years old. For 70% of patients, Clostridium bacteriemia occurred the day of ICU admission. Median Charlson score was 5 (3–7). Underlying conditions included- onco-haematological malignancy (38%), chronic alcohol abuse (28%), diabetes (28%), chronic cardiac failure (24%), obesity (24%), cirrhosis (18%), and chronic kidney injury (12%). First symptoms started the day of ICU admission (median 0 (0–1) day between symptoms and ICU admission). Patients presented with fever in only 25% of the cases, hypothermia in 16%, shock in 83%, coma in 40%, acute respiratory failure in 21%, and cardiac arrest in 26%. Digestive symptoms were presents in 52% cases. At ICU admission, median SAPS II score was 69 (49–91), median SOFA score was 11(8–14). Overall, 85% patients required vasopressors, 81% invasive mechanical ventilation, and 44% renal replacement therapy. Twenty-four percent of patients experienced massive hemolysis. Median serum lactate level was 6 mM (3–10). Most of infections (44%) were due to C. perfringens, followed by C. ramosum (10%), C. septicum (10%) and C. tertium (8%). Digestive infection was diagnosed in 62% of the cases, and skin infection in 4% of the cases. Gas gangrene was present in 12% of the patients, and surgery was necessary in 28%. Most of species were sensitive to Penicillin (93%). Two third (66%) of patients died in ICU, and hospital mortality was 80%. In non survivors, median time between ICU admission and hospital death was 1 (0–9) days.

**Conclusion**: Alcohol consumption, diabetes and underlying malignancies are the main risk factors of Clostridium bacteriemia. CLinical manifestations are non specific, but digestive symptoms and hemolysis should alert the physicians. Considering the risk of fulminant course and the high lethality of these infections, adequate antibiotics should be started as soon as the diagnosis is suspected.

### P-87 Management of tetanus in an Intensive Care Unit of Centre Hospitalier Universitaire de Libreville - a ten-years retrospective study

#### Laurence Essola-Rerambiah (*speaker*)

##### NA, Libreville, GABON

###### **Correspondence:** Laurence Essola-Rerambiah - laurenceessola@yahoo.fr

*Annals of Intensive Care* 2019, **9(Suppl 1)**:P-87

**Introduction**: tetanus, an infective non-immunizing disease, is still endemic in many developing countries and is responsible for a high mortality. The aim of our work was to describe the management of patients admitted to the ICU for tetanus.

**Patients and methods**: this is a descriptive study based on retrospective analysis done over a 10 years period, from January 2008 to December 2017 in Intensive Care Unit. Included were patients admitted for tetanus. Studied variables were sociodemographic characteristics, clinical, therapeutic and prognostic data.

**Results**: 53 out of 3031 patients (1.7%) were admitted for tetanus. Mean nage of patients was 21.2 ± 18.1 years. Showing male predominance with a sex ratio of 2.5. Trismus associated to generalised contractures and spasms was present in 32 patients (60.4%). The port of entry was found in 40 patients (75.5%). The mean score of Dakar was 2.3 ± 0.8. To struggle against spasms and contractures, the combination of magnesium sulfate and diazepam was administered in 33 patients (62.3%). Mechanical ventilation was needed in 17 patients (32.1%). The mean length of hospitalisation was 12.8 ± 8.6 days. The lethality was to 51%.

**Conclusion**: tetanus a pathology still met in our unit. Its lethality remains high. Intensification of the vaccination campaign in necessary to eradicate this disease.

### P-88 The impact of SOFA score and Lymphocytic alveolitis on predicting prognosis and mortality of pneumocystis pneumonia

#### Benjamin Gaborit (*speaker*)^1^, Benoit Tessoulin^2^, Lavergne Rose Anne^3^, Florent Morio^4^, Christine Sagan^5^, Cédric Bretonniere^6^, Raphael Lecomte^1^, Paul Le Turnier^1^, Colin Deschanvres^1^, Lydie Khatchatourian^1^, Nathalie Asseray^1^, Charlotte Garret^6^, Mickael Vourch^6^, Delphine Marest^6^, François Raffi^1^, David Boutoille^1^, Jean Reignier^6^

##### ^1^Department of Infectious Diseases, University Hospital of Nantes and CIC 1413, Nantes, FRANCE; ^2^U1232, Université de Nantes, Service d’Hématologie, Nantes, FRANCE; ^3^Laboratoire de Parasitologie-Mycologie, Institut de Biologie, Nantes, FRANCE; ^4^Laboratoire de Parasitologie-Mycologie, Institut de Biologiesité de Nantes + Service d’Hématologie, Nantes, FRANCE; ^5^INSERM, UMR1087, l’institut du thorax, Nantes, FRANCE; ^6^Medical Intensive Care, Nantes, FRANCE

###### **Correspondence:** Benjamin Gaborit - benjamin.gaborit@chu-nantes.fr

*Annals of Intensive Care* 2019, **9(Suppl 1)**:P-88

**Introduction**: Pneumocystis jirovecii pneumonia (PJP) is associated with higher rates of intubation and mortality in non-HIV immunocompromised hosts. The objectives of our study were the establishment of early risk factors of severe PJP and mortality in non-HIV patients, with a focus on the impact of broncho alveolar lavage (BAL) cytology.

**Patients and methods**: We prospectively enrolled patients with PJP admitted to Nantes University Hospital from January 2012 to January 2017. Severity was defined as acute hypoxemic respiratory failure (ARF) with high-flow oxygen use (FiO2 ≥ 50% or PaO2 FIO2 < 150). Factors associated with severity and PJP specific death were analysed by non-parametric tests and logistic regression (univariate and multivariate) in whole population and non-HIV patients.

**Results**: Among the 506 patients with pneumocystis identification, 107 patients met criteria for PJP of whom 53 (49.5%) met criteria of severity, 51 patients required ICU admission, 30 of them requiring mechanical ventilation. Death within 90 days post-admission occurred in 45% of severe vs 9% of non-severe cases (p < 0.001). Early risk factors associated with severity were- age > 55 years (odds Ratio (OR) = 2.6, 95% confidence interval (95%CI) = 1.12–6.3, p < 0.02), albuminemia < 27 g L (OR = 3.3, 95%CI = 1.25–9, p < 0.001), blood neutrophil > 6.5G L (OR = 6.5, 95%CI = 2.4–20, p < 0.001), bronchoalveolar lavage (BAL) neutrophil > 12% (5.7, 2–18, p < 0.001), BAL PJ positive direct examination (OR = 2.8, 1.2–6.9, p = 0.01), in the overall cohort. In contrast, HIV positive status (OR = 0.33, 95% CI = 0.1–1, p = 0.05), and alveolitis on BAL (0.3, 0.1 = 0.8, p = 0.01) were protective factors for severity. In multivariate analysis among the non HIV patients, the lymphocytic alveolitis (HR = 0.22, 95% CI = 0.05–0.94, p = 4e-2) was associated with improved prognosis, whereas SOFA score ≥ 5 on admission (HR = 14, 95% CI = 6–36, p = 6e-9) was associated with pneumocystosis-specific mortality.

**Conclusion**: The initial severity of pneumocystosis evaluated by SOFA score is a major prognostic factor predictive of the global and specific mortality of pneumocystosis. Age, albuminemia, bronchoalveolar lavage (BAL) neutrophil > 12%, BAL PJ positive direct examination and SOFA score ≥ 5 are associated with severe pneumocystosis with the worst prognosis.


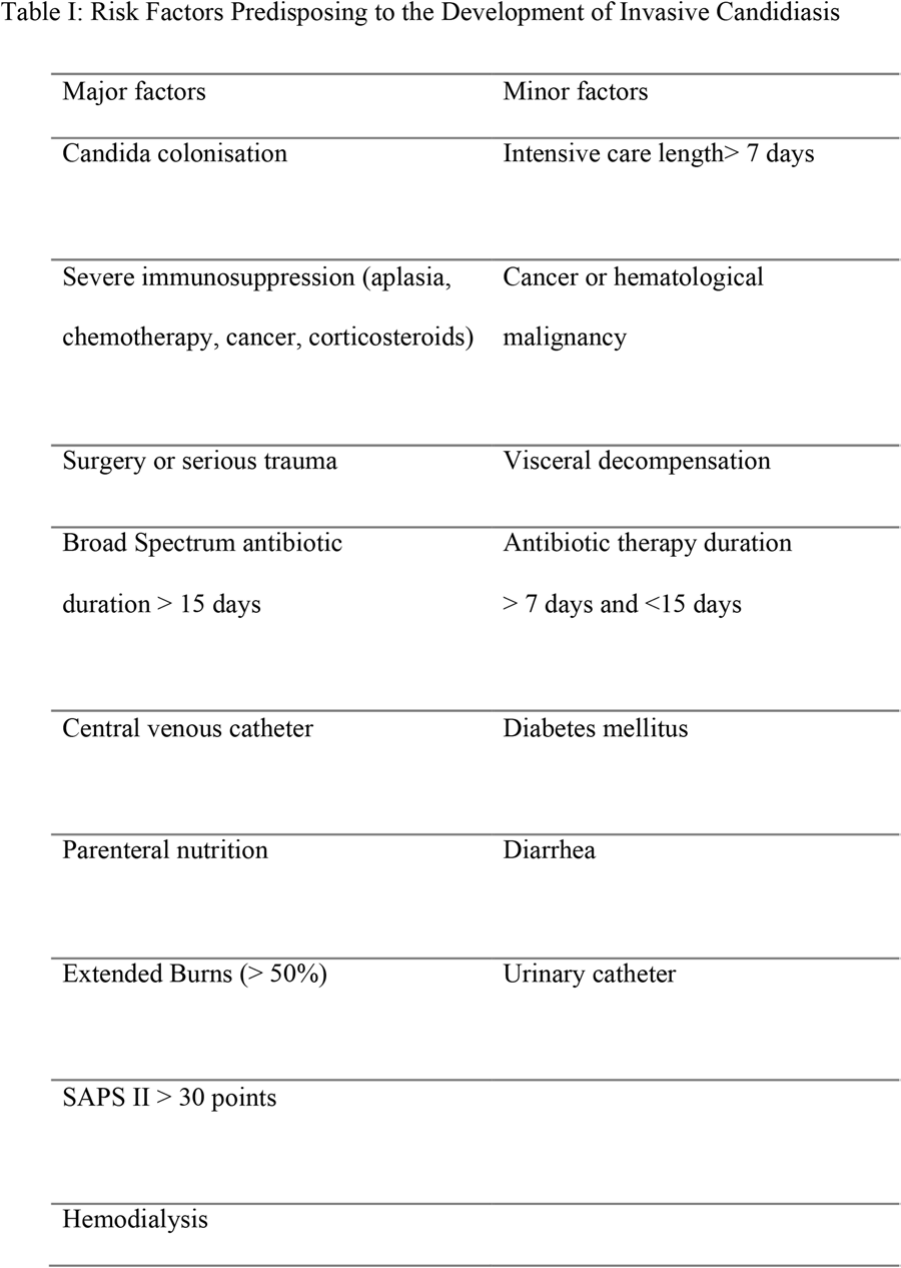


### P-89 Significance and value of candiduria for the early diagnosis of invasive candida infection in a Tunisian intensive care unit

#### Rania Ammar (*speaker*), Bouattour Abir, Chtara Kamilia, Zekri Manel, Hammami Maha, Ben Hamida Chokri, Bahloul Mabrouk , Ayedi Ali, Bouaziz Mounir

##### University of sfax, Sfax, TUNISIA

###### **Correspondence:** Rania Ammar - rania.ammarzayani@gmail.com

*Annals of Intensive Care* 2019, **9(Suppl 1)**:P-89

**Introduction**: Candiduria is increasingly frequent among patients admitted to intensive care unit. There is no clear discriminating threshold that can predict the occurrence of invasive candidiasis. Purpose- To identify the interest of the yeasts numeration in the urine for the early diagnosis of invasive candidiasis.

**Patients and methods**: A prospective study was carried out in Sfax-Tunisia intensive care unit over a period of 4 months (March–June 2016). Selection criteria- Patients included were those having at least one major risk factor or two minor risk factors to develop IC (Table I). Once included in the study, a search for Candida in urine was performed on the 3rd day of hospitalization and then once a week. Patients with candiduria carried a multiple mycological samples from other body sites and blood culture. Pittet index was calculated as well as Candida Score. The diagnosis of invasive candidiasis was made on the basis of the presence of candidemia or after expert advice during daily visits to patients with at least one of the following symptom + fever (> 38.5 °C) or hypothermia (< 36 °C), unexplained hypotension or absence of response to adequate antibiotic treatment for a suspected bacterial infection.

**Results**: heighty height patient were included. Candiduria was found in 25 patients (28.4%) and 13 patients had candiduria density > 105 CFU/mL. C. albicans was found in 13 patients. The mean interval between admission and the onset of candiduria was 11.9 ± 16.42 days. Seven patients (9%) developed candidemia + from whom 5 had a concomitant candiduria. The diagnosis of invasive candidiasis was made in 14 patients (9%). The risk factors to develop invasive candidiasis in patients with candiduria were- renal failure (p = 0.17), candida score > 2.5 points (p = 0.04) and Pittet index > 0.5 (p = 0.01). By logistic regression, only renal failure was the independent factor associated with invasive candidiasis in patients with candiduria (p = 0.02, OR 17.6, 95% CI 1.5–203.8). An association candiduria > 105 CFU ml and a Pittet index > 0, 5 were significantly associated with invasive candidiasis (P = 0.04). The mortality rates were at 37.5%.

**Conclusion**: Candiduria > 105 CFU ml in the intensive care patients with several risk factors can predict invasive candidiasis. So a permanent mycological surveillance is widely recommended in order to make the early diagnosis and to start appropriate antifungal therapy.

### P-90 Fungal colonization and infection in critically ill patients- epidemiology and risk factors

#### Ghada Sbouii (*speaker*)^1^, Ines Fathallah^2^, Khaoula Ben Ismail^2^, Sahar Habacha^2^, Haifa Fazzeni^2^, Amani Sghaier^2^, Eya Sghir^2^, Emna Ennouri^2^, Asma Mehdi^2^, Nadia Kouraichi^2^

##### ^1^Yasminet hospital, Kairouan, ABKHAZIA; ^2^Ben Arouss Regional Hospital, Medical ICU, Ben Arouss, TUNISIA

###### **Correspondence:** Ghada Sbouii - ghadasrlf@hotmail.com

*Annals of Intensive Care* 2019, **9(Suppl 1)**:P-90

**Introduction**: Fungal infections (FI) had risen and caused significant morbidity and mortality in critically ill patients. Our aims were to describe epidemiology of fungal colonization and infection in patients admitted in medical intensive care unit (ICU), and to determinate their risk factors.

**Patients and methods**: Retrospective study carried in an ICU from October 2016 to 15 September 2018. Demographic characteristics of patients and risk factors for fungal infection and colonization were evaluated. The data about epidemiology, patient significant clinical data, surgery, mechanical ventilation, dialysis, central venous catheter, urinary catheter, arterial catheter, total parenteral nutrition, leucopenia, neutropenia, previous antimicrobial therapy or prophylaxis were collected.

**Results**: Twenty-three per cent of the 164 patients enrolled in our study, had fungal infection (25 cases) or fungal colonization (13 patients). Median score was 3 [2–3]. Candida albicans was isolated in 60% of infections cases and 46.1% in colonization cases. Candiduria was detected in 40% and candidaemia was observed in 52% of patients. Patients who had developed fungal infections had central line insertion in (92%), prolonged length of stay (76%), prolonged antibiotic therapy (68%), use of corticosteroids (36%), and neutropenia (12%). Fluconazole was the first line used antifungal treatment (n = 23). Amphotericine B was used in three patients. Median hospital stay was 27 [18 - 49] days. Multivariate analysis revealed that catecholamine use was associated with fungal infection and colonization (p = 0.04).

**Conclusion**: Fungal infection and colonization were frequent in our population but only use of catecholamine appeared as a risk factor.

### P-91 Evaluation of antifungal therapy in the ICU- A bi-centre Tunisian cohort

#### Nouha Bouker (*speaker*)^1^, Zied Hajjej^2^, Zeineb Hamouda^1^, Islem Ouanes^1^, Fahmi Dachraoui^1^, Lamia Ouanes-Besbes^1^, Mustapha Ferjani ^2^, Fekri Abroug^1^

##### ^1^CHU F.Bourguiba, Monastir, TUNISIA; ^2^Hôpital Principal d’Instruction Militaire, Tunis, TUNISIA

###### **Correspondence:** Nouha Bouker - nouha.bkrr@gmail.com

*Annals of Intensive Care* 2019, **9(Suppl 1)**:P-91

**Introduction**: Invasive fungal infections are increasingly observed in the ICUs where they concern more specifically the non-neutropenic patients. In the absence of simple and accessible techniques for early microbiological diagnosis, the use of antifungals is steadily increasing, with a more frequent use of echinocandins. Little is known on the extent of the problem and the typology of antifungal prescription in Tunisian ICUs. In this bicentre study, we describe the prescription circumstances of antifungals in 2 Tunisian ICUs.

**Patients and methods**: During the study period (2014–2017) all prescription of antifungals were analysed. Analysis concerned demographics, clinical circumstances (history and acute disease, procedures, previous drugs, and life-threatening conditions at the time of antifungal therapy), as well as the basis of antifungal prescribing (targeted vs. preemptive empiric).

**Results**: 112 patients were enrolled in the study (64 men, mean age- 56 ± 18 years) were admitted. Leuconeutropenia was present in 5%, and steroids were administered to 20%. The majority of patients were mechanically ventilated (83%), had central venous line (80%), had either severe sepsis or septic shock (86%), were under large-spectrum antibiotherapy (96%) for more than 3 days (72%). Antifungal treatment was started more often on a preemptive empiric basis (52%) consisting more often in echinocandins (63%). Prescription of azoles was more often a targeted therapy (70%). Antifungal de-escalation was performed in only 2%. Infection resolved in 42%, and overall mortality was 63%.

**Conclusion**: Antifungal prescription is not exceptional in Tunisian ICUs. The preemptive empirical prescription based primarily on echinocandins reflects the lack of efficient laboratory support prompting physicians to rely on clinical information.

### P-92 Sepsis uncouples C-peptide and insulin levels in critically ill diabetic patients

#### Laurent Bitker (*speaker*)^1^, Salvatore Lucio Cutuli^1^, Luca Cioccari^1^, Eduardo A Osawa^1^, Lisa Toh^1^, Nora Luethi^2^, Helen Young^1^, Leah Peck^1^, Glenn Eastwood^1^, Johan Martensson^3^, Rinaldo Bellomo^1^

##### ^1^Department of Intensive Care, Austin Hospital, Melbourne, Australia, Heidelberg, AUSTRALIA; ^2^Australian and New Zealand Intensive Care Society (ANZICS) Research Centre, Melbourne, AUSTRALIA; ^3^Section of Anesthesia and Intensive Care Medicine, Department of Physiology and Pharmacology, Karolinska Institutet, Stockholm, Sweden, Stockholm, SWEDEN; ^11^Department of Intensive Care, Austin Hospital, Melbourne, Australia, Heidelberg, AUSTRALIA

###### **Correspondence:** Laurent Bitker - laurent.bitker@chu-lyon.fr

*Annals of Intensive Care* 2019, **9(Suppl 1)**:P-92

**Introduction**: Critically ill patients with type 2 diabetes have an increased risk of infection. Pro-insulin connecting peptide (C-peptide) has protective immunomodulatory features. Its levels may be affected by the presence of sepsis and exogenous insulin treatment. We aimed to assess how sepsis and exogenous insulin administration affected C-peptide levels and C-peptide to insulin ratio in critically ill diabetic patients.

**Patients and methods**: We studied 31 critically ill adults with type 2 diabetes. We measured serum insulin, and C-peptide levels during the first 3 days of ICU stay and recorded daily exogenous insulin dose. We obtained control data in eight volunteers. In patients unexposed to exogenous insulin therapy, we first compared those with sepsis to those without. Then, we compared septic patients unexposed to insulin therapy to those with sepsis treated with insulin. We determined parameters associated with C-peptide levels and C-peptide to insulin ratio, using multivariate linear regression.

**Results**: Sepsis was diagnosed in 22 (44%) patients. Diabetic patients with sepsis had significantly higher C-peptide levels compared to healthy controls (2.5 [1.8 + 2.8] vs. 0.5 [0.5 + 0.6] nmol/L, p < 0.01). Diabetic patients with sepsis had a 5-fold higher C-peptide to insulin ratio compared to controls (48 [33 + 72] vs. 10 [10 + 13], p < 0.01), and a 3-fold increase compared to non-septic patients (17 [12 + 35], p = 0.01). When exposed to insulin therapy, septic patients had a marked decrease in their C-peptide to insulin ratio, compared to septic patients unexposed to insulin (5 [2 + 10], p < 0.01). On multivariate analysis, C-peptide levels were significantly and negatively associated with exogenous insulin therapy, and positively associated with serum insulin levels and glucose intake. C-peptide to insulin ratio was significantly and positively associated with sepsis, and negatively with exogenous insulin therapy (p = 0.03 and p < 0.01, respectively).

**Conclusion**: In type 2 diabetic critically ill patients, sepsis is associated with a marked increase in C-peptide and C-peptide to insulin ratio, implying uncoupling of their relative secretion or clearance or both. This uncoupling, however, was markedly inhibited by the administration of exogenous insulin.

### P-93 Prognostic impact of early adjunctive corticosteroid therapy on pneumocystis pneumoniae in non-HIV patients

#### Mehdi Assal (*speaker*)^1^, Jérôme Lambert^2^, Laurent Chow Chine^1^, Magali Bisbal^1^, Lucas Servan^1^, Frédéric Gonzalez^1^, Jean-Manuel De Guibert ^1^, Marion Faucher^1^, Antoine Sannini^1^, Djamel Mokart^1^

##### ^1^Institut Paoli-Calmettes, Marseille, FRANCE; ^2^Service de Biostatistique et Information Médicale, Paris, FRANCE

###### **Correspondence:** Mehdi Assal - mehdi.assal@yahoo.fr

*Annals of Intensive Care* 2019, **9(Suppl 1)**:P-93

**Introduction**: While adjunctive corticosteroid therapy has been proven effective in HIV-patients with Pneumocystis Pneumoniae (PCP), data remains unclear and controversial concerning non-HIV related-patients. We evaluated the effects on mortality of early adjunctive corticosteroid therapy in non-HIV PCP-related patients.

**Patients and methods**: This retrospective cohort study included patients without HIV with PCP diagnosis admitted in Institut Paoli Calmette, a cancer referral centre, from January-1-2010 to December-31-2016. We compared 30-days and 1-year mortality rate, change in the respiratory item of the Sequential Organ Failure Assessment score (delta SOFA-resp), change in the global SOFA score (SOFA-aggravation) and use of intubation between day-1 and day-5 of anti-pneumocystis therapy, and occurrence of coinfections between early adjunctive corticosteroid recipients within 48 h (landmark analysis) and late or no corticosteroid recipients, using a naïve and Inverse Probability Weighted in survival analysis (IPW).

**Results**: 133 HIV-negative patients with PCP were included (early corticosteroid n = 88, late or no corticosteroid n = 45). The main underlying conditions were haematological malignancies (n = 107, 80.5%), solid tumor (n = 27, 20.3%) and stem cell transplantation (n = 17, 12.8%). Overall 30-days and 1-year mortality was respectively 24.1% and 58.2%. IPW analysis found no differences on 30-days (HR = 1.45, 95% CI [0.7–3.04], p = 0.321) and 1-year (HR = 1.25, CI 95% [0.75–2.09], p = 0.39) mortality rate between the both groups. In the same way, no differences in delta-SOFA-resp, SOFA-aggravation, use of intubation and occurrence of coinfections were found between the both groups.

**Conclusion**: The addition of early adjunctive corticosteroid to anti-pneumocystis therapy in non-HIV patients with PCP was not associated with improved outcomes concerning 30-days and 1-year mortality and respiratory evolution. Further studies are needed to evaluated this therapeutic strategy.

### P-94 Diagnostic Accuracy of PCR and B-D-glucan for the diagnosis of pneumocystis jirovecii pneumonia in immunocompromised patients with acute respiratory failure (ARF)

#### Laure Calvet (*speaker*)^1^, Virginie Lemiale^2^, Audrey De Jong^2^, Lionel Kerhuel^2^, Etienne Ghrenassia^2^, Sandrine Valade^2^, Bertrand Souweine^3^, Lara Zafrani^2^, Elie Azoulay^4^, Michael Darmon^2^

##### ^1^CHU Gabriel Montpied, Clermont-Ferrand, FRANCE; ^2^Hopital Saint Louis, Paris, FRANCE; ^3^CHU, Clermont-Ferrand, FRANCE; ^4^Hopital Saint Louis, Clermont-Ferrand, FRANCE

###### **Correspondence:** Laure Calvet - laure.calvet@yahoo.fr

*Annals of Intensive Care* 2019, **9(Suppl 1)**:P-94

**Introduction**: Accuracy of a test depends on its intrinsic characteristics (sensitivity and specificity) and of disease prevalence. These parameters are uncommonly taken into account when assessing diagnostic accuracy (1.2). To illustrate this relationship in non-HIV patients with pneumocystis pneumonia, whose prevalence is low and where available diagnostic tests have high intrinsic performance. This study aims to assess post-test probability of pneumocystis pneumonia, according to results of PCR and BDG tests in non-HIV patients with ARF.

**Patients and methods**: A systematic review was performed to assess diagnostic performance of PCR and Beta-D-Glucan (BDG). Prevalence of Pneumocystis pneumonia was assessed in a dataset of 2243 immunocompromised patients with ARF using supervised classification tree. Prevalence of pneumocystis pneumonia was simulated using r software and assuming a normal distribution in 5000 subjects on the basis of previously observed prevalence. Post-test probability was assessed using Bayes theorem. Analyses were performed using R software.

**Results**: Prevalence of pneumocystis pneumonia in ARF patients was 4.1% (95% CI 3.3–5). Supervised classification identified 4 subgroups - Patients without ground glass opacities (prevalence 2.0% + 95% CI 1.4–2.8), those with ground glass opacities but a) with prophylaxis (Prevalence 4.9% + 95% CI 1.6–11), b) without prophylaxis (Prevalence 10.0% + 95% CI 6.4–14.7), and c) without prophylaxis and with lymphoid malignancy or stem cell transplantation (Prevalence 20.2% + 95% CI 14.1–27.7). In the overall population, positive predictive value (PPV) was 32.9% (95%CI 31.1–34.8) and 22.8% (95%CI 21.5–24.3) for PCR and BDG respectively. Negative predictive value was low (0.10% (95% CI 0.09–0.11) and 0.23% (95%CI 0.21–0.25)) for PCR and BDG respectively. In the highest risk subgroup, PPV was 74.5% (95%CI 72.0–76.7) and 63.8% (95%CI 60.8–65.8) for PCR and BDG respectively.

**Conclusion**: Although both PCR and BDG yield a high intrinsic performance, the low incidence of pneumocystis pneumonia translates into limited PPV, even in the highest risk group. Our results underline the need for adequate pre-test probability assessment. They suggest a method to illustrate pre and post-test probability relationship that may improve perception of diagnostic test performance in patients with predefined clinical vignette.


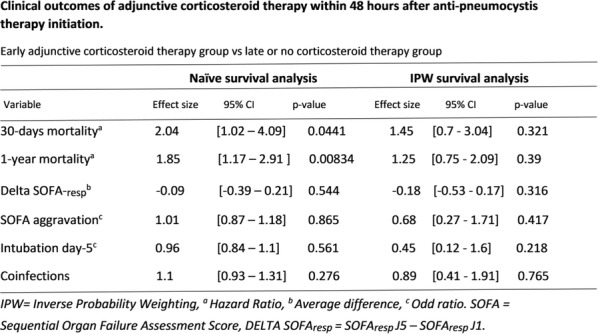


### P-95 Microbial prediction of community-acquired pneumonia- can physicians or a data-driven method differentiate viral from bacterial pneumonia at the patient presentation?

#### Claire Lhommet (*speaker*)^1^, Denis Garot^1^, Cassandra Jourdannaud^1^, Pierre Asfar^2^, Christophe Faisy^3^, Gregoire Muller^4^, Emmanuelle Mercier^1^, Sylvie Robert^1^, Philippe Lanotte^1^, Alain Goudeau^1^, Helene Blasco^1^, Antoine Guillon^1^

##### ^1^CHU, Tours, FRANCE; ^2^CHU, Angers, FRANCE; ^3^UPRES EA220, Laboratoire de Pharmacologie, 92150 Suresnes, FRANCE; ^4^CHR, Orléans, FRANCE

###### **Correspondence:** Claire Lhommet - clairelhommet@gmail.com

*Annals of Intensive Care* 2019, **9(Suppl 1)**:P-95

**Introduction**: Severe community-acquired pneumonia (sCAP) requires urgent and specific antimicrobial therapy. However, few diagnostic tools are available to diagnose the responsible pathogens when the anti-infective therapeutics must be initiated. Finally, we questioned our ability to predict the microbial etiology of sCAP within the first hours of hospitalization. As emerging evidences recently suggested that artificial intelligence-derived methods could efficiently assisted medical decision process, we wondered if mathematical model of diagnostic prediction could be more efficient than evaluation made by experimented physicians. The objective of this study was to compare the ability of a panel of experts and a mathematical model to predict the microbial etiology of sCAP.

**Patients and methods**: We conducted a prospective non-interventional study. First, we included patients hospitalized for sCAP in ICU and recorded clinical paraclinical data available in the three first hours of care. Final microbial diagnosis was established from microbiological examinations including bacterial cultures and multiplex PCR on respiratory fluids. sCAP with mixed etiology or without microbiological documentation were excluded. Second, we built a mathematical model of prediction (Random Forest method with LOOCV) using all data initially collected. Finally, an independent sample of the study population was used to test the performance of the pathogen prediction by- (i) a panel of 3 experts, (ii) the mathematical algorithm. Both were blind regarding the microbial diagnosis. Positive likelihood ratio (LR +) > 10 and negative LR < 0.1 were considered clinically relevant.

**Results**: We included 153 patients with sCAP (70.6% men, mean age, 62 [51–73] years + mean SAPSII, 37 [27–47]). Responsible pathogens were- 37% viral, 24% bacterial, 20% mixed etiology and 19% unidentified. The data-driven approach defined five items to create the final mathematical model- BMI, systolic blood pressure, symptom-to-hospitalization time and procalcitonin. Neither the experts nor the algorithm were able to predict the responsible pathogens of the pneumonia. Discriminant abilities of the algorithm were moderate to low (LR + = 2.12 for viral and 6.29 for bacterial pneumonia) and discriminant abilities of experts were low to very low (LR + = 3.81 for viral and 1.89 for bacterial pneumonia).

**Conclusion**: Our study shows that neither experts nor mathematical algorithm can predict the microbial etiology of sCAP within the first hours of hospitalization while there is an urge to define anti-infective therapeutic strategy. Our results highlight the need of developing point-of-care tests for rapid microbial diagnosis.

### P-96 Staphylococcus aureus community-acquired bacteriuria in Emergency Department - warning marker for infective endocarditis

#### Thomas Lafon (*speaker*)^1^, Lucie Lavaud^2^, Ana Catalina Hernandez Padilla^3^, Arthur Baisse^2^, Olivier Barraud^4^, Thomas Daix^5^, Marine Goudelin^6^, Bruno Evrard^6^, Bruno François^7^, Philippe Vignon^7^

##### ^1^CHU Dupuytren, Limoges, FRANCE; ^2^Service d’Accueil des Urgences, CHU Dupuytren, Limoges, FRANCE; ^3^Inserm CIC 1435, CHU Dupuytren, Limoges, FRANCE; ^4^Inserm UMR 1092 Laboratoire de Bactériologie-Virologie-Hygiène, Université de Limoges, CHU Dupuytren, Limoges, FRANCE; ^5^Inserm CIC 1435, Réanimation polyvalente, CHU Dupuytren, Limoges, FRANCE; ^6^Réanimation polyvalente, CHU Dupuytren, Limoges, Limoges; ^7^Inserm CIC 1435 Réanimation polyvalente Inserm UMR 1092, CHU Dupuytren Université de Limoges, FRANCE; ^10^Inserm CIC 1435 Réanimation polyvalente Inserm UMR 1092, CHU Dupuytren Université de Limoges, FRANCE

###### **Correspondence:** Thomas Lafon - thomas.lafon@chu-limoges.fr

*Annals of Intensive Care* 2019, **9(Suppl 1)**:P-96

**Introduction**: Urinary tract (UT) infection is a frequent diagnosis at the Emergency Department (ED). Staphylococcus aureus (Sa) is an uncommon isolate in urine cultures (0.5–6% of positive urine cultures) and is more frequent in population with risk factors for UT colonization. In the absence of urological invasive procedures or risk factors for Sa colonization, community-acquired Sa bacteriuria may be related to deep-seated Sa infection with septic embolisms and could be considered as trigger symptom. This cohort study aims to assess the prevalence of Infective Endocarditis (IE) in patients with community-acquired Sa bacteriuria in ED.

**Patients and methods**: We conducted a prospective single-center study from April 2017 to July 2018. All patients admitted in the ED with Sa bacteriuria (104 CFU ml Sa isolated from a single urine sample) and without risk factors for UT colonization (i.e., < 1 month UT surgery, UT catheterization) were included. Blood cultures were collected in patients with clinical symptoms of infection (SIRS criteria) for concomitant bacteremia (72 h). In this case, transthoracic echocardiography (TTE) or transesophageal echocardiography (TEE) were performed. Diagnosis of IE was based on the Duke criteria.

**Results**: During the study period, 56 patients with Sa bacteriuria were identified in the ED. After excluding patients with risk factors of UT colonization (UT catheterization n = 13, UT surgery n = 5), 38 patients were included (27 men, 68 [IQR- 44–80] years-old). Twenty-two patients had clinical symptoms of infection, 7 of them with UT infection. Eleven patients had concomitant Sa bacteremia and TTE or TEE was performed in 10 patients which confirmed IE in 9 patients. IE affected essentially the left heart with the mitral and aortic valves involved. Sa bacteriuria was identified significantly earlier before IE diagnosis with a mean difference of 3 days. Among Duke minor criteria, 89% of patients had septic emboli, 56% fever and 33% predisposing heart condition. The 28-day mortality was 67%.

**Conclusion**: In the absence of risk factors, community-acquired Sa bacteriuria should not be interpreted as an isolated UT infection, but as an early warning of IE, and requires further explorations to accurately and timely begin antibiotic treatment.

### P-97 Unusual bacterial epidemiology of community-acquired bacterial meningitis admitted to intensive care units in the French West Indies

#### Ulrich Clarac (*speaker*)^1^, Pascale Piednoir^1^, Amélie Rollé^1^, Frédéric Martino^1^, Hossein Mehdaoui^2^, Sébastien Breurec^3^, Sylvaine Bastian^3^, Michel Carles^1^

##### ^1^Service de Réanimation Polyvalente, CHU Guadeloupe, Les Abymes, FRANCE; ^2^Service de Réanimation Polyvalente, CHU Martinique, Fort de France, MARTINIQUE; ^6^Laboratoire de Microbiologie Clinique et Environnementale, CHU, Les Abymes, GUADELOUPE

###### **Correspondence:** Ulrich Clarac - ulrich.clarac@gmail.com

*Annals of Intensive Care* 2019, **9(Suppl 1)**:P-97

**Introduction**: Spontaneous community-acquired bacterial meningitis (CABM) are rare and of poor prognosis. CABM epidemiology has never been studied in the French West Indies. Thus, we assessed clinical and microbiological characteristics, and outcome of spontaneous CABM in adults requiring admission to intensive care units (ICUs) in French West Indies.

**Patients and methods**: Charts of consecutive patients over 18 years old requiring admission to ICUs for CABM in Guadeloupe and Martinique from January 1st, 2012 to December 31th, 2017 were retrospectively analyzed. Patients having meningitis clinical signs and positive bacterial cerebrospinal fluid cultures were included. Data are given in absolute values, percentage or median values [Q1-Q3]. Chi-2 or Mann–Whitney tests were used if required. The study protocol was approved by the local ethical committee.

**Results**: During the study period, 25 cases of CABM were identified, due to Streptococcus pneumoniae (n = 7, 28%), Klebsiella pneumoniae (n = 6, 24%), Staphylococcus aureus (n = 5, 20%), Escherichia coli (n = 2, 8%), Neisseria meningitidis B (n = 2, 8%) Streptococcus spp (n = 1, 4%), Pseudomonas aeruginosa (n = 1, 4%), Escherichia coli, Streptococcus spp (n = 1, 4%). All strains had a wild-type phenotype of antibiotic resistance. All patients were febrile, with neurologic signs (headache 56%, stroke 20%, nuchal stiffness 52%, Glasgow coma scale alteration 10 [9–14]). The median IGS score was 56 [36–57]. The hospital mortality rate was 52%. Patients stayed in ICUs for 10 [3–17] days, requiring mechanical ventilation for 72% of cases. Interestingly 4 5 Staphylococcus aureus CABM were associated with endocarditis. Whatever the bacteria, bacteraemia occurred frequently, i.e. 72% of cases, associated in 4 cases with abscesses. Initial therapeutic regimen was a third-generation cephalosporin (n = 24) alone (n = 15) or in combination with an aminoglycoside (n = 5), and or an aminopenicillin (n = 7), an aminopenicillin alone (n = 1). In comparison to other CABM (Staphylococcus aureus endocarditis excluded), Klebsiella pneumoniae CABM (n = 6) had no specific clinical presentation (p = ns).

**Conclusion**: Conversely to the epidemiology of CABM in Europe and North America, we found a high incidence of Klebsiella pneumoniae as causative microorganisms of CABM among French West Indies population. This epidemiology could be related to the specific bacterial ecology in the Caribbean area. Clinical presentation and outcome seems to be similar whatever the involved bacteria.

### P-98 Pleural Empyema in medical ICU patients

#### Dhouha Lakhdhar (*speaker*), Mohamed Slim Amri, Ghassen Ben Amor, Amira Jamoussi, Samia Ayed, Jalila Ben Khelil, Mohamed Besbes

##### Hopital A. mami Ariana, Ariana, TUNISIA

###### **Correspondence:** Dhouha Lakhdhar - lakdardoha@gmail.com

*Annals of Intensive Care* 2019, **9(Suppl 1)**:P-98

**Introduction**: The clinical presentation of pleural empyema depends upon many factors including the causative infectious agent. Patients with pleural empyema admitted in ICU often present a life threatening complication like respiratory failure or septic shock. The aim of this work was to study the bacteriological profile, the clinical presentation, causes of pleural empyema in MICU and its effect on patient outcomes.

**Patients and methods**: We conduced a retrospective, mono-centric and observational study. During eight years period, from April 2010 to July 2018, case notes of patients diagnosed with pleural empyema were reviewed. The following data were collected- clinical informations, severity upon admission, micro-organisms, treatment and outcome.

**Results**: In total, 42 cases were included, the sex ratio was 2.5. The median age was 40 years old [29, 59.5]. Median severity scores were 9 [4.5, 19] for APACHE II and 26.5 [16, 36] for SAPS II. Most of patients had an acute respiratory failure at the time of admission 76.2% (n = 32) and quite all of them (n = 41) had an associated pneumonia. Nine patients presented a septic shock and the median rate of lactates was 3.1[1.99, 6.67]. Most of pleural empyema cases were suspected by ultrasound 40.5%(n = 17), then by X chest ray 37.7%(n = 15) then by CT scan 23.8% (n = 10). Macroscopic presence of pus was noted in 25 patients (59.6%). We found negative pleural-fluid cultures in 52.4% of patients (n = 22), Alpha-hemolytic Streptococcus in ten patients, Klebsiella pneumoniae in two and anaerobic bacteria in three. Drainage of pleural content was performed in 36 patients, 23 had chest drain, three had thoracentesis and ten had both. The median duration of drainage was five days [2.75, 9.25]. Eleven patients required invasive mechanical ventilation (26.2%). Most of cases were treated by clavulanic acid, amoxicillin (n = 26). Intrapleural fibrinolytic therapy was performed in 15 cases (35.7%). Most of patients 69%(n = 29) fully recovered from pleural empyema. The median duration stay in ICU was nine days [3.75, 14.25].

**Conclusion**: Pneumonia was the main cause of pleural empyema. A high SAPS score, a bilateral pneumonia and the presence of septic shock were correlated with a high mortality. Lung abscess and duration of stay in ICU were not correlated to mortality.

### P-99 Predictors factors of mortality during spontaneous bacterial peritonitis with cirrhosis

#### Khaoula Ben Ismail (*speaker*)^1^, Sana Khedher^2^, Ameni Khaled^2^, Mohamed Salem^2^

##### ^1^Hôpital Ben Arous Yassminet, Hammam Plage, Ben Arous, TUNISIA; ^2^Hepato and Gastroenterology department - Intensive care unit, Charles Nicolle Hospital, Tunis

###### **Correspondence:** Khaoula Ben Ismail - khaoula87@hotmail.fr

*Annals of Intensive Care* 2019, **9(Suppl 1)**:P-99

**Introduction**: Spontaneous bacterial peritonitis (SBP) is serious complication of cirrhosis. Despite standard treatment, mortality remained high. We aimed to evaluate the predictors for the mortality in patients with SBP.

**Patients and methods**: It is a retrospective work, carried out over a year. Consecutive patients with approved SBP admitted in our department are included. All clinical and biological data were collected from the medical records. Univariate and multivariate analysis were used to identify the associated factors of death.

**Results**: A total of 64 patients diagnosed with SBP and cirrhosis were enrolled in this study. Mean of age was 62.05 (18–88). Sex ratio = 1. HCV (39%) was the main etiology of cirrhosis. at least one complication occurred during the evolution in 32.8%. The septic shock was found in12.5%. six patients died of SBP (9.4%). There were no significant differences in the sex, aetiology of cirrhosis, ascites abundance, receiving prophylactic antibiotics between the surviving group and the patients who died. However there was a statistically significant association between mortality and onset of complication (P = 0.00), low systolic (P = 0.003) and diastolic blood (P = 0.000) pressure and tachycardia ((P = 0.005) at admission. empiric antibiotic therapy was statically a preventive factor (P = 0.039).

**Conclusion**: SBP is a serious event in the history of cirrhotic disease. Particular caution must be exercised with regard to any state of hemodynamic instability present at admission. adequate empirical treatment would improve prognosis.

### P-100 Intra-Abdominal Infections in ICU patients (ICUBE) - Epidemiology and Risk Factors for Isolation of Multi-Drug-Resistant Organisms, a preliminary study

#### Olivier Martin (*speaker*), Nicolas Bonnet, Françoise Jaureguy, Johanna Oziel, Guillaume Van Der Meersch, Yacine Tandjaoui-Lambiotte, Florent Poirson, Philippe Karoubi, Yves Cohen

##### Hôpital Avicenne, Réanimation, Paris, FRANCE

###### **Correspondence:** Olivier Martin - olivier-martin@live.com

*Annals of Intensive Care* 2019, **9(Suppl 1)**:P-100

**Introduction**: Intra-abdominal infection (IAI) is a common cause of hospitalization in intensive care unit (ICU). The epidemiology of the microorganism depends mainly on the patients bacterial flora causes and risk factors for multi-drug-resistant organisms (MDROs) are difficult to identify. French expert advises have been published but few French studies have investigated the epidemiology of community and nosocomial-acquired IAI and more specifically the incidence of MDROs.

**Patients and methods**: We perform a prospective epidemiological multi-center study and included all consecutively hospitalized adult patients with IAI requiring a hospitalization in ICU. Cultures were performed on blood simple and intra-operative samples of peritoneal fluid or purulent exudate discrete abscesses. We evaluate the epidemiology and the factors associated with the isolation of a MDRO in enrolled patients.

**Results**: Among 48 patients included in the study, a total of 62 micro-organisms were isolated from intra-peritoneal fluid and blood samples in 56.3% of cultures, two or more pathogens were identified and in 5% candida spp. were identified. The MDROs represented 9.9% of the total of isolated microorganisms. Among this patients, 75% of MDROs colonization was found before or just after surgery and 25% were acquired during hospitalization. The MDROs were more frequently isolated in patients with health-care-associated IAIs (25.4%). Uninominal logistic regression analysis of risk factors demonstrated that statistically significant risk factors independently associated with the occurrence of MDROs IAI were previous know carrying at MDROs and antimicrobial therapy administered within 3 month before operation.

**Conclusion**: The study showed that MDROs represented a tenth of IAI and knowledge of previous colonization by MDROs or previous antibiotherapy within 3 month might guide to identify patients with IAIs caused by MDROs and therefore permit to introduce adequate empiric antimicrobial therapy.

### P-101 Improvement of patient outcomes following centralization of toxic epidermal necrolysis management

#### Florine Richeux (*speaker*), Laure Fayolle-Pivot, Marc Bertin-Maghit, Olivier Martin, Benoit Bensaid, Julien Textoris, Thomas Rimmele

##### Hopital Edouard Herriot, Lyon, FRANCE

###### **Correspondence:** Florine Richeux - florine.richeux@chu-lyon.fr

*Annals of Intensive Care* 2019, **9(Suppl 1)**:P-101

**Introduction**: Toxic Epidermal Necrolysis (TEN) is a very rare cutaneous disease (2 cases per million and per year worldwide) but potentially fatal. Morbidity is also high with the persistence of long-term sequelae (ophthalmological and psychological mainly). In November 2016, an Auvergne-Rhone-Alpes reference center was implemented in order to centralize and optimize the care of these patients. The ultimate goal of this initiative was to improve patient outcomes. The aim of this study was to assess the impact of the implementation of this centralized network on patient outcomes.

**Patients and methods**: Two groups of patients were compared- the first one from November 2012 to October 2016 before the implementation of the centralized network of care and the second one after the centralization of care, from November 2016 to December 2017. All patients suffering from TEN in the Auvergne-Rhone-Alpes area were included. The primary endpoint was procedural pain assessed by visual analogue scale (VAS).

The secondary endpoints were patient recruitment, need for general anesthesia for wound treatment, treatment duration, ICU and hospital length of stay, secondary infection rate, mortality rate at day 28, proportion of patients lost to follow-up at three months, and reported sequelae. Observed mortality was compared to that predicted by SCORTEN tool. Statistical analysis- Student or Wilcoxon test were performed for continuous variables and Chi-2 or Fisher test were performed for qualitative variables p value < 0.05 was considered statistically significant.

**Results**: 7 patients were enrolled before and 14 patients after centralization. Demographic characteristics were similar between groups. All results are presented in table 1. No fatality has occurred in the aftercare centralization whereas expected mortality according SCORTEN tool was about 35%.

**Discussion**: Centralized care of TEN in a reference center led to significant reduction of procedural pain. That seems to decrease too treatment duration, ICU and hospital length of stay, morbidity and mortality. These results are most likely due to the optimization of the patient’s course and the growing experience of the caregivers. In our opinion, the increased number of reported sequelae may highlight the under-diagnosis of these complications during the pre-centralization period.

**Conclusion**: The implementation of centralized medical care of TEN in a referrence center leads to a significant decrease of procedural pain for patients, improving their comfort thanks to an experienced team.


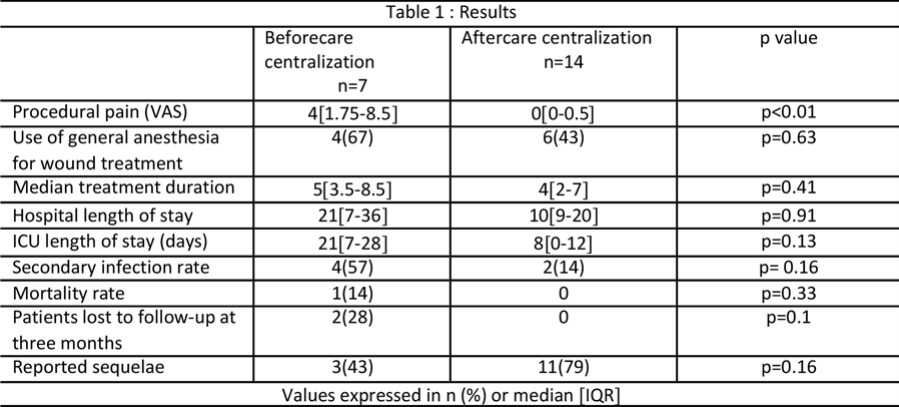


### P-102 Prediction of acute kidney injury using conventional and novel biomarkers in critically ill patients

#### Laurent Bitker (*speaker*)^1^, Salvatore Lucio Cutuli^1^, Lisa Toh^1^, Intissar Bittar^2^, GlennEastwood^1^, Rinaldo Bellomo^1^

##### ^1^Department of intensive care, Austin Healtl, Heidelberg, AUSTRALIA; ^2^Department of pathology, Austin Healtl, Heidelberg, AUSTRALIA

###### **Correspondence:** Laurent Bitker - laurent.bitker@chu-lyon.fr

*Annals of Intensive Care* 2019, **9(Suppl 1)**:P-102

**Introduction**: Acute kidney injury (AKI) is a rapidly evolving condition, requiring early identification. Urine output and serum creatinine levels are conventional markers of renal function that could help improve AKI detection if measured more frequently. AKI early identification may also be enhanced by measuring urinary tissue inhibitor of metalloproteinases-2 (TIMP-2) and insulin-like growth factor binding protein-7 (IGFBP-7). We aimed to assess the performance of conventional and novel biomarkers in predicting AKI in a general ICU population.

**Patients and methods**: In this prospective study, we enrolled 96 critically ill adults at risk of developing AKI. We excluded patients with stage 2 or 3 AKI at enrollment. We reported urine output (UO6H) and changes in serum creatinine (sCr6H), measured over the 6 h preceding inclusion. Urinary levels of TIMP-2 and IGFBP-7 were measured at inclusion. We computed a composite risk score as the ratio of log (sCr6H) to UO6H. AKI was defined as the presence of KDIGO stage 2 or 3 AKI, occurring within 12 h of inclusion. Biomarkers’ performance was expressed using the area under the receiver operator characteristics (AUROC) with 95% confidence interval.

**Results**: AKI occurred in 32 (33%) patients. At inclusion, UO6H was significantly lower, and sCr6H was significantly higher in patients with AKI, compared to non-AKI patients (0.4 [interquartile range, 0.3 to 0.7] mL/kg/h vs. 0.8 [0.5 to 1.1] mL/kg/ h and 10 [3 to 23] μmol/ L vs. +2 [-2.5 to +8] μmol L). AKI risk prediction of UO6H was fair (AUROC-0.76 [0.65 to 0.86]), and poor using sCr6H (AUROC- 0.69 [0.57 to 0.82]). TIMP-2•IGFBP-7 was significantly higher in patients with AKI, compared to those without (0.9 [0.4 to 1.8] (ng mL)2 1000 vs. 0.3 [0.1 to 0.7] (ng mL)2 1000). It had an AUROC to predict AKI of 0.72 [0.62 to 0.83]. The composite risk score showed the best AKI risk predictive performance, with an AUROC of 0.80 [0.70 to 0.90].

**Conclusion**: Combining short-termed urine output with changes in serum creatinine showed good performance for the prediction of AKI in a general ICU population. Our preliminary results suggest that conventional indicators of renal function, if measured 6-hourly, are equivalent to more recent AKI biomarkers.

### P-103 Predicting kidney dysfunction from arterial blood pressure during early septic shock

#### Bouchaala Karama (*speaker*), Rania Ammar, Emna Nouri, Kallel Hela, SabrineBradai, Mabrouk Bahloul, Olfa Turki , Kamilia Chtara, Chokri Ben Hamida, Hedi Chelly, Mounir Bouaziz

##### CHU Habib bourguiba service réanimation medicale, Sfax, TUNISIA

###### **Correspondence:** Bouchaala Karama - karamamnif@gmail.com

*Annals of Intensive Care* 2019, **9(Suppl 1)**:P-103

**Introduction**: Acute kidney injury (AKI) is a frequent and serious complication in intensive care unit (ICU) patients. Many studies have already demonstrated that sepsis and septic shock are the most important causes of AKI in critically ill patients. There is strong evidence that AKI in septic shock was associated with an important disorders in hemodynamic parameters. Despite extensive research in this field, only few studies identified a target hemodynamic status in patients with septic shock to improve kidney function. Objective- to determine the value of mean arterial blood pressure leading to acute kidney failure during early septic shock and factors associated with mortality.

**Patients and methods**: We conduct a prospective study during 6 months (from January to August 2018) including all patients presented septic shock. Patients with cardiogenic or and hypovolemic shock were excluded. We analyzed demographic characteristics, comorbidities, SAPSII score, respiratory, hemodynamic and neurological parameters, use of noninvasive or invasive ventilation, the impact of hemodynamic status on kidney function, length of stay and mortality.

**Results**: We include 43 patients, sex ratio was 2.02, and SAPSII at admission averaged 52 ± 14 points. Thirty-seven percent of cases were admitted for respiratory distress. AKI was developed in 74.4%.

Multivariable logistic regression analysis revealed that development of septic AKI was associated with older age, pre-existing chronic kidney disease, low mean arterial pressure (MAP) in septic shock day and in 24 h later. The ROC curve showed that AKI was developed in patients with MAP in septic-shock-day under 53 mmHg and under 75 mmHg 24 h later. In our study, incidence of AKI was higher in patients whom didn’t receive fluid resuscitation (27 vs 5, p = 0.01). Mortality rates was higher in AKI group (p = 0, 01). The coexisting AKI, septic myocarditis and septic cholestasis was associated with a low outcome.

**Conclusion**: The development of septic AKI is associated with poor clinical outcomes. Prevention and attenuation of septic AKI need a good fluid management and hemodynamic status adjusting. Despite large researches, the incidence and the mortality of septic AKI are high. Extensive studies to have best histopathologic information may be needed to a better management of kidney dysfunction in patient with septic shock.

### P-104 Evaluation of Sodium flux during hemodialysis and hemodiafiltration treatment of ICU acute kidney injury- Effects of dialysat Na concentration at 140 and 145 mmol/l

#### Aurèle Buzancais (*speaker*)^1^, Vincent Brunot^2^, Kada Klouche^2^

##### ^1^CHU NIMES, Nimes, FRANCE; ^2^CHU MONTPELLEIR, Montpellier, FRANCE

###### **Correspondence:** Aurèle Buzancais - aurele.buzancais@hotmail.fr

*Annals of Intensive Care* 2019, **9(Suppl 1)**:P-104

**Introduction**: Acute kidney injury (AKI) requiring renal replacement therapy (RRT) occurs in 5 to 6% of critically ill patients and is associated with high mortality. Higher sodium (Na) dialysate concentration (145 to 150 mmol/L) is recommended in order to improve intradialytic hemodynamic tolerance but may lead to sodium loading to the patient. Fluid overload has been associated with adverse ICU outcome. We aimed therefore to evaluate the flux of sodium according to 2 Na dialysat concentrations - 140 and 145 mmol/L during hemodialysis (HD) and hemodiafiltration (HDF) sessions in ICU AKI patients.

**Patients and methods**: All AKI patients requiring RRT were included prospectively in the study. Each patient underwent consecutive HD and HDF sessions with Na dialysate concentrations at 140 and 145 mmol/L. Sodium concentrations were measured in plasma before and after sessions and in affluent and effluent fluids. Flux of sodium during RRT sessions was estimated using mean sodium logarithmic concentration including diffusive and convective influx. We compared flux of sodium between HD 140 and 145, and between HDF 140 and 145 mmol/L Na concentration dialysate.

**Results**: Four-teen patients entered the study with KDIGO3 AKI mostly septic of origin. Almost all of the patients required need vasopressor drugs (71%) and mechanical ventilation (79%). They underwent 39 RRT sessions - 9 HD140, 10 HDF 140, 9 HD145 and 11 HDF145. A negative Na gradient from the dialysate replacement fluid to the patient was observed with each technique and each dialysate sodium concentration inducing a fluid overload. The comparison of HD 145 to HD 140 and HDF 145 to HDF 140 showed that higher Na dialysat induced a significant higher flux of Na to the patient and consequently fluid overload (Table). ICU mortality rate was around 30% and survived patients have a mean creatinine clairance at 38.3 ml/min/1.73m2 at ICU discharge.

**Conclusion**: Our study showed that during RRT a substantial Na loading occured and this Na loading increased significantly with elevated Na dialysate concentration from 140 to 145 mmol/L. Clinical and intradialytic hemodynamic tolerance of increased Na dialysate needs however to be further studied and analyzed.


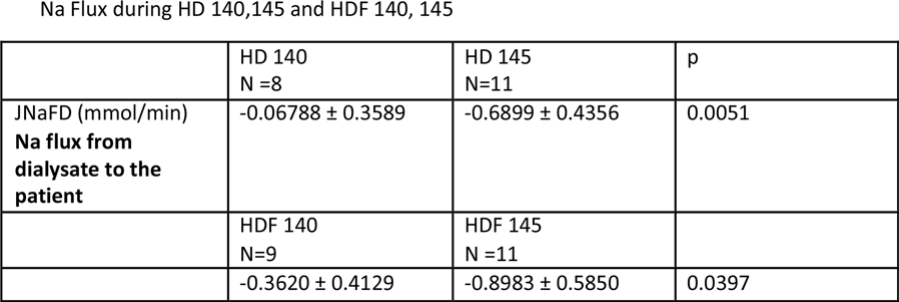


### P-105 Sodium disorders in medical intensive care - who’s the friend and who’s the foe?

#### Hanane Ezzouine (*speaker*)^1^, Amine Raja^1^, Boutaina Labib^2^, Amine Korchi^2^

##### ^1^Faculté de médecine, Université Hassan II-Casablanca, Casablanca, MOROCCO; ^3^Medical intensive care unit, University teaching hospital Ibn Rushd, Casablanca, MOROCCO

###### **Correspondence:** Hanane Ezzouine - ezzouinehanane@yahoo.fr

*Annals of Intensive Care* 2019, **9(Suppl 1)**:P-105

**Introduction**: Sodium disorders are frequent among intensive care unit patients. We aimed to determine the characteristics and the prognostic factors of the patients who developed sodium disorders in intensive care.

**Patients and methods**: We conducted a retrospective study for one year in the medical intensive care unit, of Ibn Rushd hospital in Casablanca-Morocco.The epidemiological, clinical, and therapeutic data were collected, studying two categories of patients, those who presented hyponatremia or hypernatremia in intensive care unit.

**Results**: The incidence of hyponatremia was 28.3%. the average age of the patients was 42 years. 46.5% were admitted for respiratory diseases. The mean APACHE II was at 9.9, SAPS II was at 29.2. The mean Charlson comorbidity index was 1.74. 70% of patients experienced early hyponatremia (by day 5). 40.6% of patients had mechanical ventilation, 71.9% had received antibiotics. The use of vasoactive drugs was necessary in 22.9% of cases. 36.5% of patients received diuretics, 18.8% received corticoids. 25% of patients received sodium supplementation. The outcome was favorable in 62.5%. The incidence of hypernatremia was18.28%. The average age of the patients was 43 years. Endocrine pathology is the main reason for hospitalization. The mean APACHE II 12.4; SAPS II 32.2 and OSF 3.88. The mean Charlson comorbidity index was 4.41. 29% of patients experienced early hypernatremia (by day 5). 51.6% of patients had mechanical ventilation, 79% had received antibiotics. Vasoactive drugs were necessary in 29%. 22.6% of patients received diuretics. The outcome was favorable in 59.4%. For patients who presented hyponatremia, the factors associated with mortality were APACHE II scores, reason for admission, oxygen saturation, degree of severity of the hyponatremia, hepatic function, low serum albumin, hyperfibrinigenemia, mechanical ventilation, antibiotics, vasoactive drugs,, blood transfusion and sedation, length of stay, nosocomial infection. For patients who had hypernatremia, the prognosis factors were sex gender, APACHE II score, oxygen saturation, length of stay, severity of hypernatremia, hepatic dysfunction, anemia, low serum albumin, sedation, antibiotics, vasoactive drugs, blood transfusion.

**Conclusion**: Sodium disorders are associated with a high mortality risk. Among patients who presented hyponatremia, APACHE II and SAPS II scores, early hyponatremia, mechanical ventilation, antibiotics, vasoactive drugs, corticoids, blood transfusion and sedation, length of stay, nosocomial infection and sepsis were predictive factors of mortality. On the other hand, low serum albumin, sedation and APACHE II score were associated with mortality among patients who had hypernatremia.

### P-106 Efficacity and tolerance of sustained low-efficiency dialysis (SLED) with calcium free citrate-containing dialysate anticoagulation

#### Clara Vigneron (*speaker*), Juliet Schurder, Eric Rondeau, Adrien Joseph, ChristopheRidel, Matthieu Jamme, Cédric Rafat

##### Service des Urgences Néphrologiques et Transplantation rénale, Hôpital Tenon, Paris, FRANCE

###### **Correspondence:** Clara Vigneron - claravigneron@hotmail.fr

*Annals of Intensive Care* 2019, **9(Suppl 1)**:P-106

**Introduction**: SLED is a hybrid technique using intermittent hemodialysis (iHD) equipment with lower blood and dialysate flows along with longer dialysis sessions (DS). It has gained popularity as it may allow for more efficient ultrafiltration (UF) and enhanced hemodynamic tolerance in the setting of major fluid overload. Regional citrate anticoagulation (CA) has emerged as the preferred anticoagulation technique in continuous replacement therapy thanks to decreased bleeding risk and increased extracorporeal circuit (ECC) lifetime but at the expense of an augmented risk of metabolic disorder. Herein we describe modified protocol using dialysate as a source of CA.

**Patients and methods**: Patients hospitalized in a single center nephrological intensive care who required prolonged DS with UF were included over a 6 months period. Patients treated with curative anticoagulation were excluded. Clinical, biological and metabolic characteristics and coagulation scores were collected. Patients had alternatively iHD during 4 h or SLED during 6 h. During iHD, we prescribed 250 to 300 mL/min blood flow with a 500 mL/min dialysate flow while during SLED, we used respectively 250 mL/min, 300 mL/min. UF was left at the physician’s discretion. The dialysate composition was- potassium 3 mmol/L, sodium 139.75 mmol/L, magnesium 0.5 mmol/L, calcium 0 mmol/L, citrate 0.8 mmol/L and glucose 1 g:L. Calcium and magnesium infusion rates were based on ionic dialysance following a chart previously devised for standard iHD sessions.

**Results**: 38 DS prescribed for 4 patients were analysed- 16 iHD, 22 SLED. Weight loss over 24 h was significantly increased after SLED (2.25L [1.25 − 3]) compared to iHD (0L [0 − 1.])(p = 0.001). Concerning safety, there was no difference of ionized calcium (iCa) measured every hour during the session without citrate overload (table 1). Levels of sodium, magnesium, potassium, phosphate, bicarbonate and anion gap after session were not different. There was no difference regarding pressure profile evolution and early interruption of dialysis or UF, despite higher UF with SLED. iCa measurements from the dialyzer outlet were consistently below the predefined threshold of 0.4 mmol/L. We observed higher scores of membrane clotting but not of ECC clotting with SLED. No bleeding event was observed.

**Conclusion**: SLED using a modified dialysate as a source of CA appears to be a safe and efficient technique to provide UF. It may represent a useful renal replacement therapy in patients with major fluid overload and a high bleeding risk.


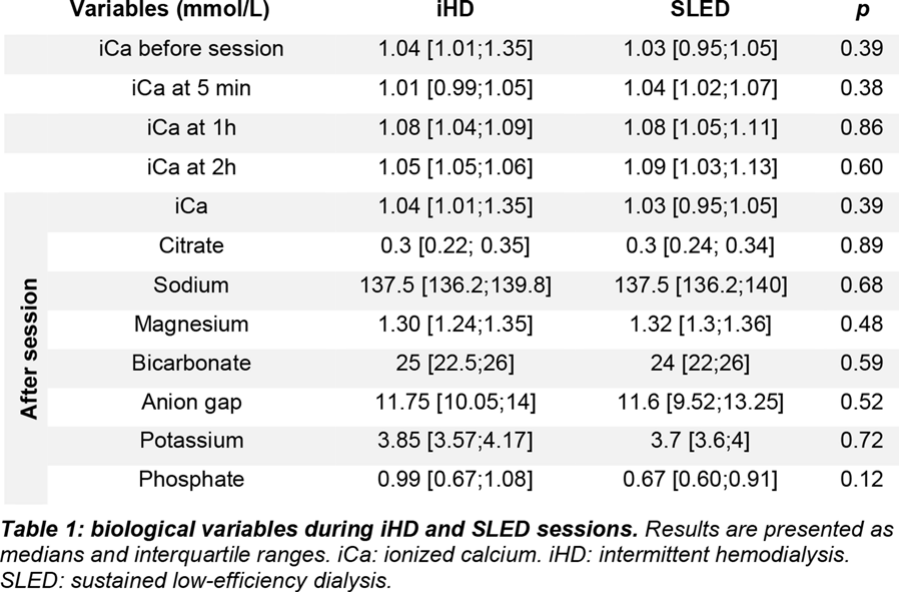


### P-107 Management of acute renal failure in severe malaria in children at the Centre Hospitalier Universitaire de Libreville

#### Laurence Essola-Rerambiah (*speaker*)

##### NA, Libreville, GABON

###### **Correspondence:** Laurence Essola-Rerambiah- laurenceessola@yahoo.fr

*Annals of Intensive Care* 2019, **9(Suppl 1)**:P-107

**Introduction**: Acute renal failure is a rare complication of severe malaria in children. The aim of this study was to evaluate the therapeutic management in our context.

**Patients and methods**: We have realized an observational, transverse and descriptive study over a 21 months, (January 1st 2015 to September 30, 2017) at the Pediatric Emergency Department and Intensive care unit of the Centre Hospitalier Universitaire de Libreville. All patients having acute renal failure with positive thick film, were included in this study. Studied variables included social and demographic data, clinical, paraclinical, therapeutic and prognostic data.

**Results**: during the study period 1629 patients (35%) were admitted for management of malaria. Among them 12 (0.7%) patients, 6 boys (50%) and 6 girls (50%) presented with acute renal failure due to malaria. Mean age was 102.2 ± 66.7 months. Renal Replacement Therapy, indicated for all patients, was effective in 4 patients (33.3%). Hemodialysis was the only technique used. The mean duration of hospitalization was 10.8 ± 4.3 days and mortality was 33.3%.

**Conclusion**: This study have shown that acute renal failure complicating severe malaria in children is really rare and that renal replacement therapy is only feasible in older children (≥ 11years). It is essential to improve the facilities for managing children.

### P-108 Cardio renal syndrome and acute exacerbation of COPD (AE COPD)- An ignored combination?

#### Wafa Zarrougui (*speaker*), Sameh Ben Farhat, Jihene Mahmoud, Rafla Ben Dabbebis, Hend Zorgati, Said Kortli, Ahmed Seghaier, Houssem Hmouda

##### Sahloul University hospital Sousse, Sousse, TUNISIA

###### **Correspondence:** Wafa Zarrougui - wafa.zarrougui91@gmail.com

*Annals of Intensive Care* 2019, **9(Suppl 1)**:P-108

**Introduction**: Cardio renal syndrome (CRS) is a group of disorders resulting from the pathological interaction between the heart and the kidneys. Studies on the prevalence of CRS in patients admitted for AE COPD are limited. In this setting, the discovery of a renal dysfunction is not rare, and has important therapeutic implications, particularly when it is a CRS. Its impact on mortality, and its prognostic value in this group of patients deserves a special emphasis. AIM- to evaluate the prevalence of CRS, as well as its impact on mortality in patients admitted for AE COPD.

**Patients and methods**: We retrospectively reviewed the charts of consecutive patients admitted for AE COPD in a medical ICU from November 2015 to February 2018. We collected clinical features at admission, severity of illness, ICU course, and the occurrence of CRS. Univariate and multivariate regression analyses were performed to identify factors independently associated with CRS, and to determine the risk of mortality secondary to CRS.

**Results**: A total of 21 patients were admitted for AE COPD. Their main characteristics were-.

mean age, 63 ± 11 years, male, 91(19%), COPD GOLD D, 14(66.7%), mean APACHE II, 15.8 ± 6.2, pH, 7.32 ± 0.1, PaCO2, 65.6 ± 29 mmHg, PaO2, 163 ± 133 mm Hg, initial invasive mechanical ventilation, 12 (57.1%), mean duration of mechanical ventilation, 9.03 ± 7.38 days, mean length of stay, 12.1 ± 10.8 days, mortality, 3(14.2%). Eight patients (38.1%) developed CRS. Among them, 5 patients (62.5%) had type 1 CRS, 1 patient (12.5%), had type 2 CRS, and 3 patients (37.5%) had type 5 CRS. Underlying Cardiac diseases in patients with CRS were hypertensive cardiomyopathy in 2 cases (25%), ischemic cardiomyopathy in 1 case (12.5%). Pulmonary arterial hypertension was found in all patients having CRS with a mean systolic PAP of 42.6 ± 11.6 mmHg. All patients with CRS had septicemia. Univariate analysis showed a significant association between CRS and acute circulatory failure at admission (p = 0.02). The occurrence of CRS was associated with an increased risk of mortality (p = 0.017).

**Conclusion**: CRS is frequent in patients admitted for AE COPD and was significantly associated with circulatory failure at admission, as well as a significant risk of death.

### P-109 The Artificial Kidney Initiation in Kidney Injury 2 (AKIKI 2)- where are we for this multicenter randomized controlled trial?

#### Stéphane Gaudry (*speaker*)^1^, David Hajage^2^, Jean-Pierre Quenot^3^, Laurent Martin Lefevre^4^, Guillaume Louis^5^, Steven Grange^6^, Jean Reignier ^7^, Julien Mayaux^8^, Beatrice La Combe^9^, Nicolas Chudeau^10^, Rémi Bruyère^11^, Badie Badie^12^, Jonathan Messika^13^, Karim Lakhal^14^, Dimitri Titeca^15^, Nicolas De Prost^16^, Nadia Aissaoui^17^, Guillaume Chevrel^18^, Saad Nseir^19^, Alain Combe^8^, Yves Cohen^20^, Marion Beuzelin^21^, Julien Bohé^22^

##### ^1^Hôpital AVICENNE, Bobigny, FRANCE; ^2^Unité de Recherche Clinique des Hôpitaux Universitaires Pitié Salpêtrière – Charles Foix, Paris, FRANCE; ^3^CHU Dijon Bourgogne, Dijon, FRANCE; ^4^CHU Dijon Bourgogne, La Roche Sur Yon, FRANCE; ^5^CHR-Mertz-Thionville, Hôptital de Mercy, Metz, FRANCE; ^6^CHU, Rouen, FRANCE; ^7^Hôtel Dieu, Nantes, FRANCE; ^8^Hôpital Pitié Salpêtrière, Paris, FRANCE; ^9^CH Bretagne Sud, Lorient, FRANCE; ^10^CH, Le Mans, FRANCE; ^11^CH de Bourg-En-Bresse - Fleyriat, Bourg En Bresse, FRANCE; ^12^Hôpital Nord Franche-Comt, CH, Belfort, FRANCE; ^13^Hopital louis mourier, Colombes, FRANCE; ^14^Hopital Nord Laennec, Nantes, FRANCE; ^15^CHU. D’Amiens Picardie, Amiens, FRANCE; ^16^Hôpital Henri Mondor, Créteil, FRANCE; ^17^Hôpital Georges Pompidou, Paris, FRANCE; ^18^CH Sud Francilien, Corbeil Essonnes, FRANCE; ^19^Hopital Roger Salengro, Lille, FRANCE; ^20^Hôpital Avicenne, Bobigny, FRANCE; ^21^CH, Dieppe, FRANCE; ^22^CH LYON-SUD, Pierre Bénite, FRANCE

###### **Correspondence:** Stéphane Gaudry - stephanegaudry@gmail.com

*Annals of Intensive Care* 2019, **9(Suppl 1)**:P-109

**Introduction**: Timing of renal replacement therapy (RRT) for severe acute kidney injury (AKI) is highly debated. Three multicenter trials assessed this question. Both AKIKI and IDEAL-ICU showed no mortality difference between an early and delayed RRT strategy. Pending results of STARRT-AKI (which is ongoing), the bulk of evidence suggests that, in the absence of severe complications of AKI, delaying RRT is safe. Duration of anuria and serum urea concentration were among criteria mandating RRT in the delayed strategy of AKIKI. The validity of such criteria is open to debate. We designed a study that compares the AKIKI “delayed strategy” with a further delayed one.

**Patients and methods**: AKIKI 2 is a prospective, multicenter, open-label, randomized trial. Randomization is preceded by an observational stage where patients receiving (or having received) catecholamines and or mechanical ventilation and with severe KDIGO3 AKI and no potentially life-threatening condition (severe hyperkalemia, severe acidosis or pulmonary edema resulting in severe hypoxemia) are observed. If one or both of the following criteria occur- serum urea concentration > 40 mmol/L and or oliguria anuria > 72 h, patients will be randomly allocated to one of the two arms- 1 “no further delayed strategy”- RRT will be initiated within 12 h after documentation of randomization, 2 “further delayed strategy”- RRT will be initiated only if one or more life-threateningcondition (see above) occur or if serum urea concentration reaches 50 mmol/L. The primary outcome is the number of RRT-free days 28 days after randomization. Considering a mean RRT-free days at day 28 of 17 ± -11.4 days in the “No further delayed strategy” (data derived from AKIKI), total sample size of 270 patients is required in the randomized stage to demonstrate an increase of 4 days (25%) in “further delayed strategy”. Approximately one third of patients in observational stage should be eligible for the randomization stage then we expect that to 810 patients will be included in the observational stage.

**Results**: Five months after study beginning, 27 centers are active (on the 43 planned to open), 142 patients were included and 33 randomized. Inclusions should be completed in December 2019.

**Conclusion**: AKIKI 2 will allow a precise description of the natural history of AKI KDIGO3 and will expand results of previous large randomized controlled trials by showing whether or not it is possible to further delay RRT initiation in this population.

### P-110 Team communication in an acute medical unit: A Social network analysis

#### Sara Benammi (*speaker*)^1^, N. Madani^1^, K. Abidi^1^, T. Dendane^1^, A.Zeggwagh^1^, Jihane Belayachi^2^, R. Abouqal^1^

##### ^1^Faculty of Medicine of Rabat UM5, Rabat, MOROCCO; ^2^Centre Hospitalier Universitaire Avicenne, Rabat, MOROCCO

###### **Correspondence:** Sara Benammi - sara-benammi@hotmail.fr

*Annals of Intensive Care* 2019, **9(Suppl 1)**:P-110

**Introduction**: Social network analysis seeks to understand networks and their participants and has two main focuses- the actors and the relationships between them in a specific social context. Applications in the health sector remain underutilized. We sought to use Social Network Analysis (SNA) to describe the patterns of communications in teamwork of an acute medical unit (AMU).

**Patients and methods**: Network Analysis was conducted to examine network structure of teamwork professional communication in an AMU of a university Hospital. All eligible personnel (n = 58) were included in SNA survey. Team members reported the frequency (0 to 10 + times) of professional discussion with every other coworker during the last 48-hours. To examine the structure of the network, density, degree and betweenness centralization, degree and betweenness centrality were calculated. Scores range from 0 to 100%. Higher Value indicated, the more dense, more centralized is the network, and most central are team member in the network respectively. We examined the homophily of the network using E-I index (from -1to 1), where -1represented communication only between staff of different function and 1 communication only between staff with Similar function. P-value was obtained based on 1000 quadratic assignment procedure QAP permutations of the network. The network analysis was used to construct network maps using multidimentional scaling and generates a visual representation of networks through network diagrams.

**Results**: The mean age of participants was 37 ± 13 years, there were 460connections (density = 28%). The whole network has a moderate degree centralization (37%) and lowerbetweeness centralization (8%). The seven team members most central (centrality degree > 50%) to the network included three senior physicians, the head nurse, the physiotherapist, the medical secretary and the archivist. There was evidence regarding homophily in a network indicated by high level of E-I index value 0.44 (P < 0.01, by QAP) indicating low degree of communication among different function team member.

**Conclusion**: SNA revealed moderate team member connectedness in the network as measured by density. The AMU Network showed moderate degree of centralization, and low betweenness centralization which supposed that team is connected by well positioned members to support inter-team communication, and a greater number of gatekeepers dominate the network over other members in the network. In effect, five function profile, have a central role in bridging communication. However, this analysis also shows a possible point of weakness of our medical unit, represented by the low degree of communication among different function team member.

### P-111 Adverse events in a Tunisian intensive care unit- frequency, risk factors and outcomes

#### Said Kortli (*speaker*), Imen Ben Saida, Hend Zorgati, Nesrine Fraj, Nawres Kaacem, Wafa Zarrougui, Mohamed Ahmed Boujelben, Mohamed Boussarsar

##### Farhat Hached University Hospital, Medical Intensive Care Unit, Sousse, TUNISIA

###### **Correspondence:** Said Kortli - kortlisaiid@gmail.com

*Annals of Intensive Care* 2019, **9(Suppl 1)**:P-111

**Introduction**: Adverse events (AEs) are common. Recognizing and reporting AEs is a crucial step for caregivers to implement adequate strategies to improve patient safety. The aim was to evaluate the rate, risk factors of AEs and their impacts.

**Patients and methods**: A prospective study conducted from October 2017 to June 2018 in a 9-bed medical ICU. All included patients were monitored for AEs. Variables found to be statistically significant in univariate analysis were introduced into a multivariate regression model to identify factors independently associated to AEs.

**Results**: 137 patients were included. Patients’ characteristics were- median age, 60[49–68] years, female, 29(27.9%), median SAPSII, 27[22–33.5], Invasive mechanical ventilation (IMV), 57(41.6%), vasopressors use, 52(38%) and respiratory disorder was the main reason for admission in 103 patients (75.2%). 177 AEs occurred in 69 patients during a median length of stay (LOS) of 10 [5.5–20]. The most frequent AEs were- ventilator acquired pneumonia, 40(29.2%), removing gastric tube, 25(18.2%) pressure sores, 21(15.3%), removing central peripheral line, 20(14.6%), removing bladder catheter, 16(11.7%), accidental extubation, 13(9.5%), fall, 13(9.5%), catheter related bloodstream infection, 12(8.8%), acute renal failure, 11(8%) and venous thromboembolic events, 6(4.4%). Patients who presented AEs had significantly longer duration of IMV (13.52 ± 110.97 vs 3.5 ± 7.07 days, p = 0.000), longer LOS (18.20 ± 13.34 vs 10.04 ± 9.78 days, p = 0.000) and higher mortality rate (36.2% vs 13.2%, p = 0.008). Univariate analysis revealed the following factors to be associated to AEs respectively - age ≥ 65 years, (49.3% vs 13.2%, p = 0.044), SAPSII (30.67 ± 9.5 vs 24.6 ± 7.9, p = 0.000); IMV use (88.4% vs 23.5%, p = 0.00); vasopressors use (56.5% vs 19.1%, p = 0.00); corticosteroids’ use (71.7% vs 50%, p = 0.012); sedative agents use (71% vs 22.1%, p = 0.000) and delirium (50.7% vs 32.4%, p = 0.000). Multivariate regression model identified two factors as independently associated to AEs: delirium (OR, 3.6; 95%CI, [1.3- 10]; p = 0.013) and IMV duration (OR, 1.11; 95%CI, [1.05- 1.18]; p = 0.000).

**Conclusion**: This study highlights the serious problem of AEs in ICU. Delirium and IMV duration were identified as independently associated to AEs.

### P-112 Impact of early ICU admission on outcome of critically ill and critically ill cancer patients

#### Yannick Hourmant (*speaker*), Sandrine Valade, Lara Zafrani, Michael Darmon, Elie Azoulay

##### Hopital Saint Louis- Médecine Intensive Réanimation, Paris, FRANCE

###### **Correspondence:** Yannick Hourmant - yannick.hmt@gmail.com

*Annals of Intensive Care* 2019, **9(Suppl 1)**:P-112

**Introduction**: Early ICU admission has been proposed as a mean to limit risk of clinical worsening and to improve outcome of ICU patients. The aim of this review was to investigate the impact of early ICU admission in the general ICU population and in critically-ill cancer patients (CICP).

**Patients and methods**: This systematic review was performed according to PRISMA statements and the protocol was registered in the PROSPERO database (CRD42018094828). Studies reporting impact of delay before ICU on outcome were searched on PubMed (1980- 2017) for adult patients with or without cancer. Differences in term of mortality is reported as Risk Ratio (95%CI). Publication bias was assessed by visually inspecting the funnel plot and summary estimates of relative risk and their 95% confidence interval were calculated using both fixed and random-effects model.

**Results**: Overall ICU population - Among the 663 citations identified for general ICU population, 29 studies reporting on 72,801 patients were included, including 66 768 patients for the early admission group. Early ICU admission was associated with decreased mortality using a random effect model (RR 0.65, 95% confidence interval 0.58–0.73, I2 = 66%) (figure). CICP - Among the 932 citations identified, 14 studies reporting on 2,414 patients (including 1,272 with early ICU admission) were included. Early ICU admission was associated with decreased mortality using a random effect model (RR 0.69, 95% confidence interval 0.52–0.90, I2 = 85%). To explore heterogeneity, a meta-regression was performed. Characteristics of the trials (prospective vs. retrospective, monocenter vs. multicenter) had no impact on findings. Publication after 2010 (median publication period) was associated with a lower treatment effect (estimate 0.37, 95%CI 0.14–0.60, P = 0.002) in the general ICU population. A significant publication bias was observed. Several sensitivity analyses were performed using trim and fill method, Copas method and taking into account Outcome Reporting Bias, which confirmed findings to be robust in both the general ICU population and the CICP.

**Conclusion**: These results suggest that early ICU admission is associated with decreased mortality in the general ICU population and in CICP. These results were however obtained from high risk of bias studies and a high heterogeneity was noted. Additional studies are required to confirm potential benefit of early ICU admission along with its cost–benefit ratio.


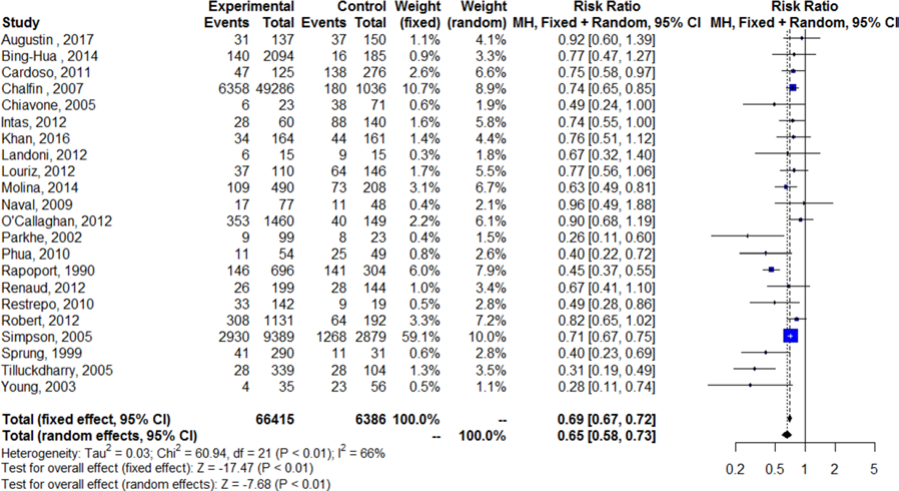


### P-113 ICU Health Care Workers are poorly aware of the costs of the devices they use for patients’ care

#### Paul Gabarre (*speaker*)^1^, Pierre-Yves Boelle^1^, Muriel Fartoukh^1^, Christophe Guitton^1^, Guillaume Dumas^1^, Jean-Rémi Lavillegrand^1^, Jean-Luc Baudel^1^, Naike Bigé^1^, Daniel Zafimahazo^1^, Hafid Ait Oufella^1^, Eric Maury^2^

##### ^1^Medical ICU, Paris, FRANCE; ^2^Hôpital SAint Antoine, AP-HP, Paris, FRANCE

###### **Correspondence:** Paul Gabarre - paul.gabarre@aphp.fr

*Annals of Intensive Care* 2019, **9(Suppl 1)**:P-113

**Introduction**: ICU Heath care workers (ICU HCW) use expensive devices for the care of their patients. Unfortunately lifetime of some of these multi users devices is often shorter than that could be waited for a single user utilization. The aim of this survey was to evaluate ICU HCWs’ knowledge of the costs of device they regularly use during and for ICU patients’ care.

**Patients and methods**: HCWs of three ICUs- medical (1) medico surgical (2) university affiliated (2) were proposed to answer to an anonymous questionnaire aimed at measure their estimation of the cost of 44 devices or systems used in daily patients care. These include catheter, ultrasound device + Renal Replacement therapy device, ventilator…, Costs’ estimation are expressed as percentage of real costs. Costs’ estimation are compared using median and [1st and 3rd quartile] according to HCW status and magnitude of costs of device. A correct estimation was defined by an estimated cost equivalent to real cost ± 50%.

**Results**: 128 HCWs accepted to take part to the survey providing more than 5600 costs estimation. Median global cost estimation for all the devices was 50% [14–215] and decreased with the real cost of devices - 428% [375–2500] for a real cost < 100 €, 70% [18–210] for a real cost more than 100€ and < 1000 €, 52% [12–163] real cost between 10 000 and 100 000€ and 34% [9–78] for devices with a cost more than 100 000€. Accuracy of costs estimation was 6% (real cost ± 10%, 15% for an estimated cost ± 25% and 26% for an estimates cost ± 50%. Senior physicians performed better than junior physicians.

**Conclusion**: HCWs are unaware of the real cost of the devices they use for ICU patients’ care in 75% of the cases.

### P-114 Comparison of the mortality prediction of different ICU scoring systems (Apache II, SAPSII, SOFA and CSS) in a low income country medical ICU

#### Imen Ben Saida (*speaker*), Said Kortli, Hend Zorgati, Nawres Kacem, ImenEl Meknessi, Sana Rouis, Ahmed Khedher, Abdelbaki Azouzi, Khaoula Meddeb, Mohamed Boussarsar

##### Farhat Hached University Hospital, Medical Intensive Care Unit, Sousse, TUNISIA

###### **Correspondence:** Imen Ben Saida - imen.bensaida@yahoo.com

*Annals of Intensive Care* 2019, **9(Suppl 1)**:P-114

**Introduction**: Clinical assessment of the severity of illness is an essential component of medical practice, especially in the intensive care unit (ICU). Multiple scoring systems have been developed for the ICU to risk stratify patient, predict outcome, help care providers in decision making and guide the allocation of resources. The aim of the study was to compare the performance of a local Validated clinical severity score (CSS) to severity scoring systems- Physiology and Chronic Health Evaluation (APACHE) system II, the Simplified Acute Physiology Score II (SAPS II), and the Sequential Organ Failure Assessment score (SOFA).

**Patients and methods**: A retrospective study was performed in our MICU between January 2017 and December 2017. Data were collected by reviewing the medical patients’ charts. Scoring systems were calculated based on the worst values recorded during the first 24 h of admission. Discrimination was evaluated using receiver operating characteristic (ROC) curves.

**Results**: A total of 301 patients were enrolled in the study. Mean age was 55.5 ± 19.1. 63.1%(n = 190) were male. The most common reasons for admission were acute respiratory failure in 183 (60.8%) patients, neurological and cardiovascular disorders in respectively 46 (15.3%) and 35 (11.6%) patients. 177 patients (58.8%) were intubated and 129 (42.9%) needed vasopressors. Mean length of ICU stay was 11.2 ± 17 days. The mean SAPSII, Apache II, SOFA and CSS were respectively 31.2 ± 12, 12.9 ± 6.7, 4.4 ± 2.7 and 20.8 ± 11.9. The overall mortality rate was 29.9%.

The best performing ICU scoring system in this study was CSS which had an area under the ROC curve (AUC) of 0. 786 (95% CI- 0.728–0.845) (p = 0.000). SOFA, SAPSII and Apache II have respectively an AUC of 0.712 (95% CI- 0.649–0.775) (p = 0.00) + 0.677 (95% CI- 0.61–0.74) (p = 0.000) and 0.64 (95% CI- 0.576–0.711) (p = 0.000) (figure 1).

**Conclusion**: The findings of the present study showed that SOFA, SAPS II and APACHE II had good accuracy in predicting mortality in ICU. However, they are partially perfect. This emphasize the importance of repeated validations of these scores. CSS is an interesting, not time-consuming, costless and minimally invasive tool for predicting mortality in ICU.


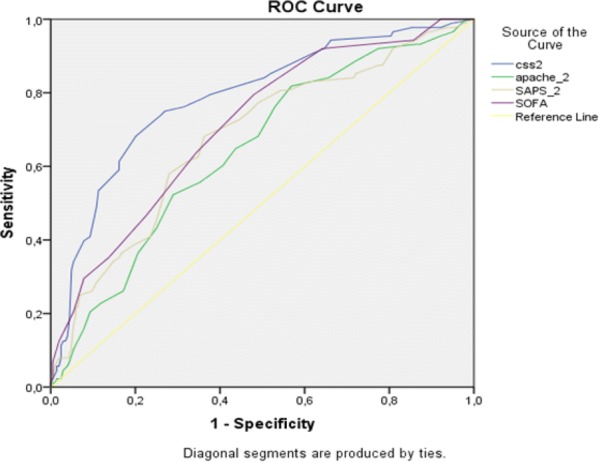



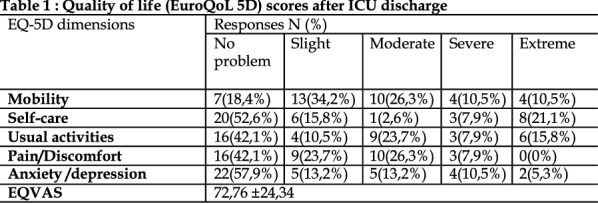


### P-115 Appropriateness of Admission and Hospitalizations Days in an Acute Medical Unit

#### Sara Benammi (*speaker*)^1^, A. Saadi^1^, N. Madani^1^, K. Abidi^1^, T. Dendane^1^, A.a. Zeggwagh^1^, Jihane Belayachi^2^, R. Abouqal^1^

##### ^1^Faculty of Medicine of Rabat UM5, Rabat, MOROCCO; ^2^Centre Hospitalier Universitaire Avicenne, Rabat, Rabat

###### **Correspondence:** Sara Benammi - sara-benammi@hotmail.fr

*Annals of Intensive Care* 2019, **9(Suppl 1)**:P-115

**Introduction**: Eliminating unnecessary health care may reduce the cost and improve the quality of healthcare. This study aimed to measure the appropriateness of hospitalization, and to identify patient and hospitalization characteristics associated with appropriateness of hospitalization in an Acute Medical unit of University Hospital.

**Patients and methods**: Prospective data including all patients admitted to the Acute Medical unit were collected over 2 months. The appropriateness of 2263 hospital days contributed by 300 patients was assessed by means of Appropriateness Evaluation Protocol. We assessed equity in the distribution of hospital resources among patients, and the cumulative appropriateness of hospitalization by the means of Gini coefficient. The Gini index ranged from 0 to 1, where zero represents perfect equality in the distribution. Predictor variables included patient’s anthropometric characteristics, and hospitalization characteristics. Associations between patient-related variables, and inappropriate hospital days related variables were studied using Generalized Estimation Equations for univariate and multivariate logistic regression analysis.

**Results**: Overall, 10% of hospital admissions and 5% of hospital days were rated as inappropriate. In univariate analysis, inapproprite hospital days were more frequent among patients whose admission was inappropriate. Factors significantly related to inappropriate stays- Hospitalization in a single room (OR = 0.55, 95%CI-0.34 to 0.88, p=0.01). Absence of previous hospitalization (OR = 0.65, 95%CI-0.3to0.98, p = 0.04). No transfer request to another department (OR = 1.88, 95%CI-1.23to2.88, p = 0.003). No transfer done to another department (OR = 2.26, 95% CI-1.28 to 4, p = 0.005). Diagnosis of hematological disease (OR = 0.53, 95% CI-0.27 to 1.03, p = 0.05). In multivariate analysis, only the diagnosis of hematological disease remained significantly related to inappropriate stay (OR = 0.4, 95% CI- 0.20 to 0.80, p = 0.001). The days of inappropriate hospitalization were less evenly distributed than the appropriate days, the Gini coefficients were 0.34 and 0.26 respectively.

**Conclusion**: This study shows that inappropriate hospitalizations in an acute medical unit are frequent. At admission, one bed out of ten could be free, and one patient out of twenty could have a shorter stay. Considering that emergency departments are chronically saturated and have limited funds, inadequate hospitalization results in unnecessary cost and less efficiency and quality of care in the acute medical unit.

### P-116 Factors predicting mortality in elderly patients admitted to a tunisian medical intensive care unit

#### Dhouha Ben Braiek (*speaker*)^1^, Hend Zorgati^2^, Saiid Kortli^2^, Rafla Ben Dabebiss^2^, Mohamed Ben Rejeb^3^, Houssem Hmouda^2^

##### ^1^CHU Farhat Hached Sousse, Sousse, TUNISIA; ^2^Sahloul University Hospital, Medical Intensive Care Unit., Sousse, TUNISIA; ^3^Sahloul University Hospital, Department of Prevention and Care Safety., Sousse, TUNISIA

###### **Correspondence:** Dhouha Ben Braiek - bbraiek_dhouha@hotmail.com

*Annals of Intensive Care* 2019, **9(Suppl 1)**:P-116

**Introduction**: With increasing life expectancy, elderly patients will require intensive care unit (ICU) admission more frequently. Age is thought to be strongly associated with ICU mortality, but other clinical variables may be incriminated. Early recognition of patients at high risk of mortality will help outcome prediction, better care planning, and health care cost containment strategies development. The aim of our study was to describe the characteristics of tunisian elderly patients admitted to ICU, and identify predictive factors of ICU mortality.

**Patients and methods**: A prognostic study type survival analysis was conducted in a medical ICU of a university hospital during a 4-year period, including patients ≥ 65 years. Baseline characteristics, clinical and laboratory parameters, treatment, and outcome were recorded. Univariate and multivariate analysis were performed using survival analysis.

**Results**: During the study period, 420 patients were admitted, of whom 25.7% (n = 97) were included. The mean age was 75 ± 7 years. The overall ICU mortality was 22%. Acute respiratory failure was the most common reason of hospitalization (78.4%), and community-acquired pneumonia was the main etiology (70.1%). Mean APACHE II, SAPS II and SOFA scores were 22 ± 10, 40 ± 11 and 7 ± 4. Fifty-three per cent required invasive mechanical ventilation, 57.7% required vasoactive drugs. The most common complications were hemodynamic disorders (80.4%) followed by nosocomial infections (51.5%), and renal failure (57.7%). The mean LOS was 12 ± 11 days. On univariate analysis, factors associated with mortality were- a past history of chronic renal failure (p = 0.006), shock (p = 0.036), ARDS (p = 0.016), resuscitation for cardiac arrest (p = 0.02), severity scores as SOFA (p < 10^−3^), APACHE II (p < 10^−3^), and SAPS II (p < 10^−3^), acute renal failure at admission (p = 0.029), vasoactive drugs (p = 0.026) and sedation (p = 0.014). On multivariate analysis, independent predictive factors of mortality were severity scores SAPS II (HR, 1.06, 95%CI [1.02–1.09], p = 0.003) and APACHE II (HR, 1.07, 95%CI, [1.02–1.13], p = 0.01).

**Conclusion**: Even though old age is linked to a high risk of death, age alone does not appear a strong predictor of mortality. Severity of illness on ICU admission was the main predictive factor of death. Further longitudinal studies of long-term survival in the elderly are needed.

### P-117 Out of the ICU shifting by intensivist

#### Hamid Merdji (*speaker*)^1^, Raphael Clere-Jehl^1^, Auguste Dargent^2^, Pascal Andreu^2^, Audrey Large^2^, François Lefebvre^1^, Maleka Schenk ^1^, Julie Helms^1^, Jean-Pierre Quenot^2^, Ferhat Meziani^1^

##### ^1^Hôpitaux Universitaires de Strasbourg, Strasbourg, FRANCE; ^2^CHU de Dijon, Dijon, FRANCE

###### **Correspondence:** Hamid Merdji - hamidmerdji@hotmail.fr

*Annals of Intensive Care* 2019, **9(Suppl 1)**:P-117

**Introduction**: Many studies have focused on the work of hospital doctors, but little is known about the work of Intensive Care Unit (ICU) physicians, especially concerning the medical time spent outside the unit and its workload that has never been evaluated to date. The main objective of our study was to evaluate the time spent by ICU doctors outside the unit, for the management of their patients during the intra-hospital transport (IHT) and for the care of patients in other departments (vital emergency, advices on medical care and ethical discussion). Secondary objectives were to describe the organization and distribution of medical time outside the ICU.

**Patients and methods**: In this prospective and observational study, which took place during 5 years, from January 2012 to December 2016 in two academics medical ICU (Strasbourg and Dijon), after each intervention outside the ICU, the intensivist doctor timed the intervention. Every day during the morning medical staff, the anonymized data were collected in a register.

**Results**: During the five years of the study, 2874 h have been spent by intensivists outside the ICU in Strasbourg and 1740 in Dijon. This corresponds to an average of 574 h 32 min (± 175) per year, about 94 min per day in Strasbourg, and an average of 349 h (± 82) per year, about 58 min per day for Dijon. Over these five years, there were 5463 shifting, which corresponds to an average of 1,092 shifting (± 169) per year in Strasbourg. And 3028 shifting in Dijon, which corresponds to an average of 605,6 shifting (± 165) per year. About one third of this time was spent in the emergency department, one third in intra-hospital medical wards and the last third during IHT for ICU patients requiring a computerized tomography scan and or a cardiac catheterization.

**Discussion**: A better understanding of the different activities and the respective part they occupy in the daily life of intensivists is essential in order to optimize the care for hospitalized patients.

**Conclusion**: This prospective French bicentric study highlights the fact that the intensivists’ medical activity outside their own unit is time-consuming. This part of the intensivist activity is often unrecognized by the hospital administration and should now be taken into account concerning the routine activity of an ICU as well as the care given at the patient’s bedside, the time devoted to the reception of families, and the time devoted to meetings.


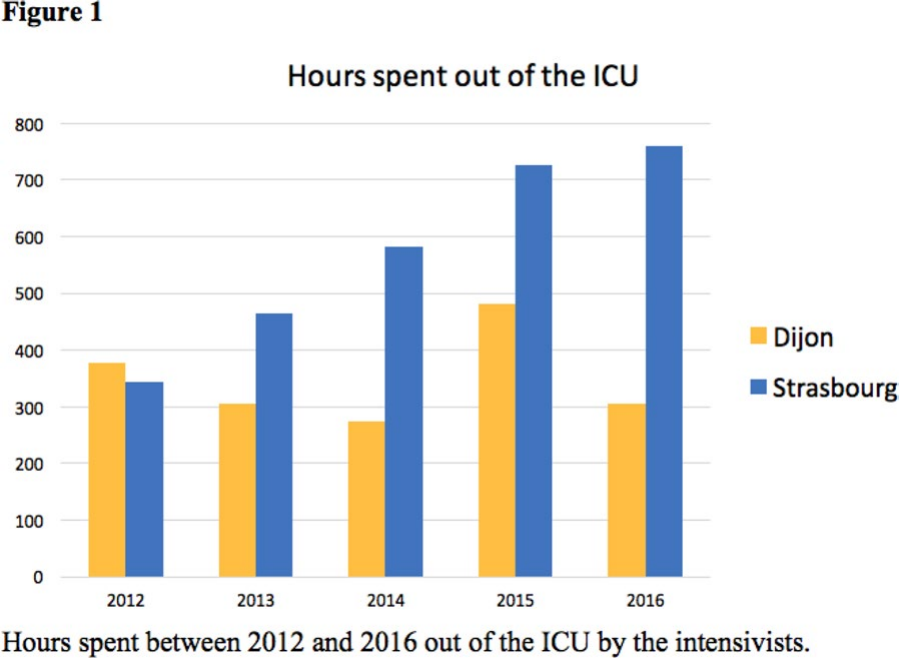


### P-118 Training of French nursing students on drawing blood culture: results from a broad electronic survey

#### Barbara Alves (*speaker*), Vivien Benoit, Romain Jouffroy

##### Departments of Anaesthesia & Intensive Care Unit, SAMU, Hôpital Universitaire Necker - Enfants Malades, Université Paris Descartes, Paris, FRANCE

###### **Correspondence:** Barbara Alves - brb.alv@gmail.com

*Annals of Intensive Care* 2019, **9(Suppl 1)**:P-118

**Introduction**: Bacteraemia induces a high rate of morbi-mortality. Blood cultures (BCs) are the standard method for the diagnosis of bacteraemia. In France, practice recommendations exist concerning BCs with an update in 2016 to avoid inadequate sample resulting in false diagnosis, inadequate antibiotherapy, and prolonged hospital length of stay. Appropriate training for nursing students could promote good clinical practice since, in France, BC are mainly taken by nurses. The aim of this study is to evaluate the theoretical and practical training received by French nursing students at school and at hospital on BC execution as well as related hygiene practices.

**Patients and methods**: We performed a cross-sectional study to evaluate the theoretical and practical training received by French nursing students and the hygiene practices concerning BC. The study was based on an electronic survey using a questionnaire sent out to all French nursing students between October 2017 and January 2018. The survey encompassed 22 short answer and multiple-choice questions.

**Results**: One thousand and thirty-six nursing students filled out the survey- 90% female, mean age of 24 ± 6 years. At nursing school, only 57% of the nursing students declared to have received theoretical training only on BC execution, 87% who received practical training only and 30% theoretical and practical training. During internship, 49% declared to have received practical training, 6% theoretical training and 45% declared no training. Among BC execution recommendations, peripheral stick and first aerobic are well known among 88% and 83% of the nursing students, respectively. Similarly, the practice of washing hands and cleaning the venepuncture site prior to BC execution are known among 96% and 94% of the students. In contrast, the practice of wearing gloves (80%) and facial mask (15%) is relatively lower.

**Conclusion**: There are discrepancies between the knowledge base of nursing students and good practice recommendations for blood culture execution and related hygiene practices. Strengthening the teaching practices will likely improve students’ knowledge base, reduce blood culture contamination and improve quality of care.

### P-119 Cardiogenic shock in France- what and who are we talking about? A descriptive analysis of the FRENSHOCK multicenter prospective registry

#### Clément Delmas (*speaker*)^1^, Etienne Puymirat^2^, Meyer Elbaz^3^, Bruno Levy^4^, Guillaume Laurent^5^, Stéphane Manzo-Silberman^6^, Laurent Bonello ^7^, Sebastien Champion^8^, Nadia Aissaoui^9^, Francis Schneider^10^, Edouard Gerbaud^11^, Nicolas Lamblin^12^, Francois Roubille^13^, Patrick Henry^14^, Eric Bonnefoy^15^

##### ^1^Rangueil University Hospital, Toulouse, FRANCE; ^2^Intensive Cardiac care Unit, Cardiology department, Paris, FRANCE; ^3^Intensive Cardiac care Unit, Cardiology department, Toulouse, FRANCE; ^4^Brabois medical Intensive Cardiac Unit, Nancy University Hospital, Vandoeuvre-Les-Nancy, FRANCE; ^5^Intensive Cardiac Care Unit, Cardiology department, Rennes, FRANCE; ^6^Intensive Cardiac Care Unit, Cardiology department, Paris, FRANCE; ^7^Intensive Cardiac Care Unit, Cardiology department, Marseille, Marseille; ^8^Intensive Care Unit, Le Chesnay, FRANCE; ^9^Medical Intensive Care Unit, Paris, FRANCE; ^10^Medical Intensive Care Unit, Strasbourg, FRANCE; ^11^Intensive Cardiac Care Unit, Cardiology department, Pessac, FRANCE; ^12^Intensive Cardiac Care Unit, Cardiology department, Lille, FRANCE; ^13^Intensive Cardiac Care Unit, Cardiology department, Montpellier, FRANCE; ^14^Intensive Cardiac Care Unit, Cardiology department, Paris, FRANCE; ^15^Intensive Cardiac Care Unit, Cardiology department, Lyon, FRANCE

###### **Correspondence:** Clément Delmas - delmas.clement@chu-toulouse.fr

*Annals of Intensive Care* 2019, **9(Suppl 1)**:P-119

**Introduction**: Epidemiologic data about cardiogenic shock (CS) are still poor and focused on ischemic CS, forgetting all part of the CS encountered in clinical practice.

**Patients and methods**: FRENSHOCK registry (NCT02703038) was a large prospective multicenter registry of non-selected CS patients admitted in critical care units realized between April and October 2016 in France. Patients were included if they met the following three criteria- (1) low cardiac output defined by SBP < 90 mmHg and or the need of amines, or a low cardiac output defined by CI < 2.2L/min/m2 (TTE or Swan-Ganz) + (2) elevation of left and or right heart pressures defined by clinic radiology biology echocardiography Swan-Ganz + and (3) clinical and or biological hypoperfusion.

**Results**: 772 patients were included in 49 centers (male 71.5%, mean age of 66y ± 15). Comorbidities were classical- previous coronary revascularization 26%, history of extra cardiac arterial disease 15%, previous renal failure 21% and COPD 6%. Cardiovascular risk factors included diabetes (28%), active tobacco (28%), dyslipidemia (35%) and hypertension (47%). 56% were known for previous cardiomyopathy (especialy 30% ischemic origin). CS etiology often associated several triggers but ischemic was retained for only 36.4% (n = 281) of patients with type 1 infarction for 17.4% (n = 134). Non-ischemic trigger factors were predominant (n = 491 + 63.9%)- supra ventricular (13.2%) and ventricular arrythmia (12.6%), infection (11.9%), iatrogenic (6.1%), conductive disorders (2.3%), non-observance (3.5%), and others (13.7%). At admission median SBP was 101 mmHg ± 25. Sinusal rhythm was present in only 52%. Right heart failure signs were present in 49% and left signs in 72% (Killip IV for 49%). Biological analysis found signs of hypoperfusion with high lactate (3.0 95% CI [2.0–4.8]), renal (eGFR 49.6 ± 26.8 mL/min/m2) and hepatic alteration (ASAT 90 UI ml, 95% IC [39–300], Prothrombin time 57 ± 25%). Biventricular failure was frequent (LVEF was 26% ± 13 + TAPSE 13 mm ± 5). When realized (n = 399 + 52%) coronarography was pathological in 81% (n = 321) (monotroncular 31%, bitroncular 35% and tritroncular 34%). A culprit lesion was found in 79% and concern LVA in 48%, RCA in 23% and left main in 15%.

**Conclusion**: This large multicentric and prospective registry confirmed the heterogeneity of CS in terms of etiology, presentation and prognosis with a predominance of non-ischemic CS in practice.

### P-120 Incidence and severity of RV size in patients with distributive shock. Value of tricuspid annular plan systolic excursion (TAPSE)

#### Amélie Prigent (*speaker*)^1^, Philippe Vignon^2^, Xavier Repesse^1^, Gwenael Prat^3^, Cyril Charron^1^, Michel Slama^4^, Antoine Vieillard-Baron^1^, Guillaume Geri^1^

##### ^1^University hospital Ambroise Paré, Boulogne-Billancourt, FRANCE; ^2^University hospital, Limoges, FRANCE; ^3^University hospital La Cavale Blanche, Brest, FRANCE; ^4^University hospital, Amiens, FRANCE

###### **Correspondence:** Amélie Prigent - prigent.amelie@gmail.com

*Annals of Intensive Care* 2019, **9(Suppl 1)**:P-120

**Introduction**: Experts proposed to define RV failure as a state in which RV is unable to meet the demands for blood flow without excessive dilatation. While TAPSE (tricuspid annular plan systolic excursion) was frequently reported as a pertinent parameter to study the RV function, this is still questionable. Our goal was (i) to report the incidence of RV dilatation and its severity in distributive shock, (ii) to report the distribution of TAPSE in different groups according to the RV size and the serum lactate level.

**Patients and methods**: Retrospective analysis of an observational, prospective multicenter study, which included 540 patients admitted in the ICU for shock, under mechanical ventilation, in whom an echocardiography (transthoracic and transesophageal) was systematically performed. After exclusion of cardiogenic, hypovolemic and obstructive shock, 345 patients were screened. Combining the lactate level and the end-diastolic ratio between the right and the left ventricle (RV LV EDA), 4 groups were defined. Group 1 without RV dilatation (RV LV EDA ≤ 0.6), group 2 and 3 with a moderate RV dilatation (RV LV EDA > 0.6 ≤ 0.8) without or with an increased lactate level (≤ or > 2 mmol/L) and group 4 with a severe RV dilatation (RV LV EDA > 0.8).

**Results**: 327 patients were analyzed. Median age was 66 [iqr 57–75], SAPS2 57 [iqr 43–72] and SOFA score 10 [iqr 7.5–12]. Cause of shock was septic in 85% of cases. The overall in-ICU mortality was 37.6%. 150 patients (45.9%) were in group 1, 51 (15.6%) in group 2, 53 (16.2%) in group 3 and 71 (22.3%) in group 4. 29.1% patients were ventilated for an ARDS. Median RV LV EDA was 0.5 [iqr 0.4–0.5], 0.7 [iqr 0.6–0.7], 0.7 [iqr 0.6–0.7] and 0.9 [iqr 0.9–1.1] in groups 1, 2, 3 and 4 respectively. No inter-group difference was observed for TAPSE with the same median value of 18 mm (p = 0.48). Central venous pressure was significantly higher in group 4 (12 [9–15] mmHg) compared to group 1 (9 [7–12]), 2 (9 [7–12]) and 3 (9 [7–13]).

**Conclusion**: RV dilatation was observed in 54.1%, moderate in 59% and severe in 41%. TAPSE was not discriminant with a normal value in all groups.

### P-121 Management and outcome of out-of-hospital cardiac arrest in elderly patients- a regional experience in Lower Normandy

#### Bertrand Sauneuf (*speaker*)^1^, Xavier Souloy^1^, Maxime Leclerc^2^, Benoit Courteille^3^, Julien Dupeyrat^4^, Michel Ramakers^5^, Frédéric Godde^6^, Cédric Daubin^7^

##### ^1^CH Public du Cotentin, Cherbourg, FRANCE; ^2^CH Mémorial, St-Lo, FRANCE; ^3^CH Avranches Granville, Avranches, FRANCE; ^4^CHU, Caen, FRANCE; ^5^CH Mémorial, St-Lo, FRANCE; ^6^CH Avranches Granville, Avranches, FRANCE; ^7^CHU, Caen, FRANCE

###### **Correspondence:** Bertrand Sauneuf - b.sauneuf@ch-cotentin.fr

*Annals of Intensive Care* 2019, **9(Suppl 1)**:P-121

**Introduction**: In-hospital admission of elderly patients resuscitated from out-of-hospital cardiac arrest (OHCA) has increased. If some authors reported favourable outcomes, others reported an increase risk of death or survival with loss of autonomy. In addition, the benefit of some interventions (i.e. transport to percutaneous coronary intervention (PCI)) and the time and resources associated with, should be evaluated. Consequently, we conducted a study evaluating the prognosis of the elderly OHCA patient in a region, with only one PCI center.

**Patients and methods**: We retrospectively included all patients aged 75 or older, admitted in 4 intensive care units (1 university- and 3 non university hospitals) after OHCA between January 2009 and December 2016. Cardiac arrest and patients characteristics have been collected and the Cardiac Arrest Hospital Prognosis (CAHP) score has been calculated. The primary outcome was the neurocognitive function assessed by the cerebral performance category on hospital discharge.

**Results**: During the study period, 176 patients were included (median age 81 [79–84], 72% male). Most of the patients presented significant comorbid condition (median Charlson index 5 [3–6]).

Sixty-eight patients had initial shockable rhythm (38.6%). Hundred sixty-six patients presented without obvious extra-cardiac cause. PCI was performed in 63 (35.8%) patients. The number of non-neurologic organ failure on admission was lower in patients with favorable outcome. All patients who received renal replacement therapy had an unfavourable outcome. The rate of favorable neurological outcome at hospital discharge was 9% (16 patients) including 14 patients with initial first shockable rhythm. At 6 months, this rate decreases to 6% (11 patients), including 9 patients with initial first shockable rhythm. According to the CAHP score, 21 patients had a low risk of poor neurological outcome, 44 had an intermediate risk and 70 had a high risk. Based on this CAHP score stratification, 14 patients (66.7%) in the low risk group, 21 patients (44.7) in the intermediate risk group and 18 patients (25.7%) in the high-risk group underwent early PCI.

**Conclusion**: Survival of elderly patients is low after OHCA, especially for patients with first non shockable rhythm. However, patients with first shockable rhythm and low risk of death may have favorable long term outcome. The level of organ failure seems to be strongly associated with prognosis. Stratification by CAHP score suggest that the use of PCI coud be improved with a better selection of patients likely to benefit from it.

### P-122 Healthcare costs and resource utilization associated with treatment of out-of-hospital cardiac arrest

#### Guillaume Geri (*speaker*)^1^, Damon C Scales^2^, Maria Koh^3^, Harindra C Wijeysundera^2^, Dennis TKo^2^, Steve Lin^4^, Michael Feldman^2^, Sheldon Cheskes^2^, Paul Dorian^4^, Wanrudee Isaranuwatchai^5^,Laurie J Morrison^4^

##### ^1^Ambroise Paré Hospital, APHP, Boulogne-Billancourt, FRANCE; ^2^Sunnybrook Health Sciences Centre, Toronto, CANADA; ^3^Institute for Clinical Evaluative Sciences, Toronto, CANADA; ^4^Rescu, Li Ka Shing Knowledge Institute at St Michael’s Hospital, Toronto, CANADA; ^5^Centre for Excellence in Economic Analysis Research, Toronto, CANADA

###### **Correspondence:** Guillaume Geri - guillaume.geri@aphp.fr

*Annals of Intensive Care* 2019, **9(Suppl 1)**:P-122

**Introduction**: The management of out-of-hospital cardiac arrest (OHCA) patients requires the coordination of prehospital, in-hospital and post-discharge teams. Data reporting a comprehensive analysis of all costs associated with treating OHCA are scarce.

**Patients and methods**: We performed an analysis on a merged database of the Toronto Regional RescuNet Epistry database (prehospital data) and administrative population-based databases in Ontario. All non-traumatic OHCA patients over 18 years of age treated by the EMS between January 1, 2006, and March 31, 2014, were included in this study. The primary outcome was per patient longitudinal cumulative healthcare costs, from time of collapse to a maximum follow&#8208 + up until death or 30 days after the event. We included all available cost sectors, from the perspective of the health system payer. We used multivariable generalized linear models with a logarithmic link and a gamma distribution to determine predictors of healthcare costs.

**Results**: 25,826 44,637 patients were treated by EMS services for an OHCA (mostly male 64.4%, mean age 70.1). 11,727 (45%) were pronounced dead on scene, 8,359 (32%) died in the emergency department, 3,640 (14%) were admitted to hospital but died before day-30, and 2,100 (8.1%) were still alive at day-30. Total cost was $690 [interquartile range (IQR) $308, $1,742] per patient + ranging from $290 [IQR $188, $390] for patients who were pronounced on scene to $39,216 [IQR 21,802, 62,093] for patients who were still alive at day-30. In-hospital costs accounted for 93% of total costs. After adjustment for age and gender, rate of patient survival was the main driver of total costs- the rate ratio was 3.88 (95% confidence interval 3.80, 3.95), 49.46 and 148.89 for patients who died in the ED, patients who died after the ED but within 30 days, and patients who were still alive at day-30 compared to patients who were pronounced dead on scene, respectively. Factors independently associated with costs were the number of prehospital teams, the need for hospital transfer, coronary angiography and targeted temperature management.

**Conclusion**: Clinical outcome is the main driver of total costs of treating OHCA patients in a large Canadian health system. Potentially modifiable factors include the number of prehospital teams that arrive to the scene of the arrest and the need for between-hospital transfers after successful resuscitation.

### P-123 Hyperkalemia is related more to hypercapnia than to acidemia during lactate accumulation after cardiac arrest: impact on clinical management

#### Matthieu Jamme (*speaker*)^1^, Guillaume Geri^2^, Thomas Robert^3^, Alain Cariou^4^, Laurent Mesnard^1^

##### ^1^Urgences Néphrologiques et Transplantation Rénale, Hôpital Tenon, APHP, Paris, FRANCE; ^2^Medecine intensive Réanimation, Hôpital Ambroise Paré, APHP, Boulogne-Billancourt, FRANCE; ^3^Néphrologie et Transplantation rénale, Hopital de la Conception, APHM, Marseille, FRANCE; ^4^Medecine intensive Réanimation, Hôpital Cochin, APHP, Paris, FRANCE

###### **Correspondence:** Matthieu Jamme - mat.jamme@gmail.com

*Annals of Intensive Care* 2019, **9(Suppl 1)**:P-123

**Introduction**: Low pH acidemia has been historically described as one of the major factors associated with hyperkalemia. But a few older studies suggested that, in the context of an elevated anion gap metabolic acidosis, acidemia is not linked to hyperkalemia. As a consequence, the administration of sodium bicarbonate, long considered as first-line therapy for acute hyperkalemia, was recently re-assessed for the treatment of either hyperkalemia. Regarding the high prevalence of hyperkalemia and acidemia in resuscitated out-of-hospital cardiac arrest (OHCA) patients, we share here our experience of severe acute type I lactic acidosis, the most frequent cause of metabolic acidosis in this setting.

**Patients and methods**: We retrospectively analyzed first arterial blood gas results of 828 successfully resuscitated OHCA patients admitted to a tertiary Parisian intensive care unit. Baseline characteristics were compared according to the presence of hyperkalemia and factors associated with kalemia were identified using multivariable linear regression. sensitivity analysis was repeated in the subgroups of patients without chronic respiratory disease (CRD) and those without acute kidney injury (AKI).

**Results**: Admission blood lactate was below 1.5 mmol/L in 88 828 (10.6%) patients and potassium levels were above 5.5 mmol/L in 93 828 (11.2%). Patients with hyperkalemia presented more frequently with initial non-shockable rhythm and experienced longer collapse before resuscitation. At ICU admission, patients with hyperkalemia presented with deeper acidemia (7.1 [6.95–7.18] vs 7.23 [7.13–7.32], p < 0.001), higher lactatemia (7.3 [3.7–12.5] vs 5.1 [2.5–9], p < 0.001) and higher serum creatinine level (159 [102–230] vs 103 [77–137], p < 0.001). Following multivariate linear regression, kalemia was only associated with initial non-shockable rhythm (β = -0.34, 95%CI = -0.58 ± 0.11), higher creatininemia (β = 0.003, 95%CI = 0.002, 0.004) and elevated PaCO2 (β = 0.01, 95%CI = 0.005 + 0.02) but not elevation of lactate levels. Indeed, a statistical negative trend between potassium blood level and lactatemia was observed (β = -0.02, p = 0.07). Sensitivity analyses performed on patients without AKI and CRD showed similar results.

**Conclusion**: Our study demonstrates the absence of an association between blood lactate and potassium levels during acidosis with severe acidemia in the successfully resuscitated OHCA patient setting. This phenomenon appears to be independent of AKI. Our results suggest that kaliemia are in fact strongly linked to arterial PaCO2.


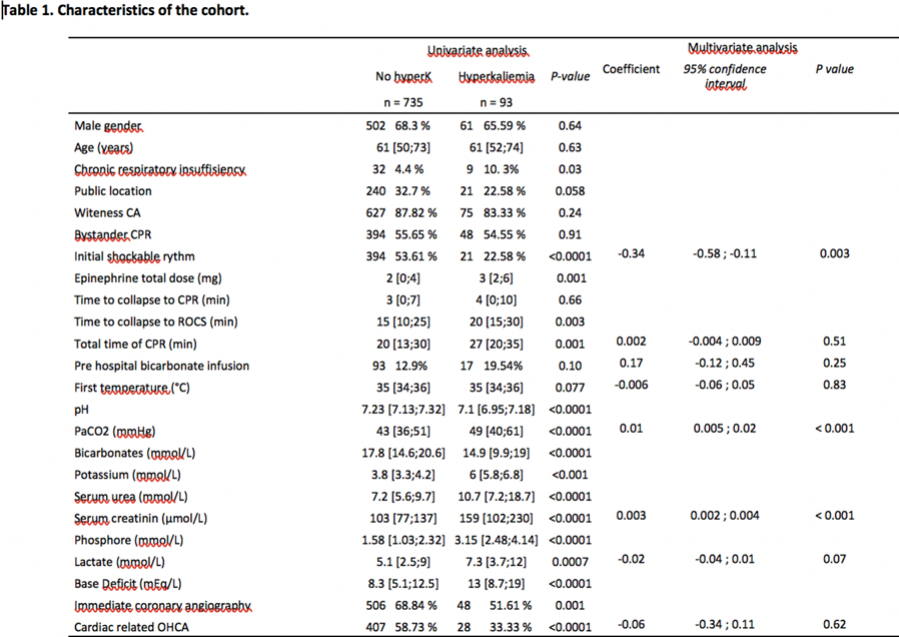
 
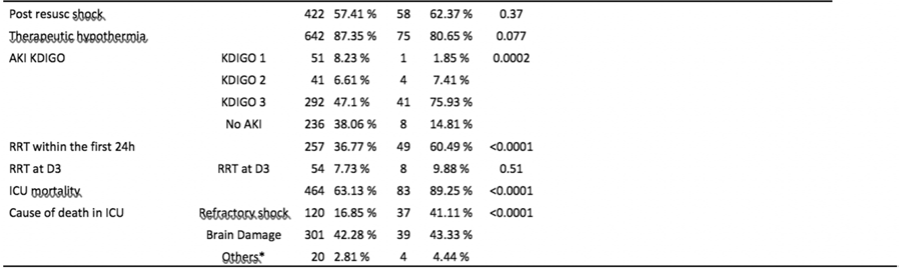


### P-124 Prevalence and risk factors of vascular complications following veno-venous and veno-arterial ECMO weaning

#### Cecile Bouges (*speaker*)^1^, Timothee Abaziou^2^, Jean Porterie^3^, Fanny Vardon^2^, Thierry Seguin^2^, Vincent Minville^2^, Bernard Georges^2^, Jean Marie Conil^2^, François Xavier Lapebie^4^, Alessandra Bura Riviere^4^, Laurent Brouchet^5^, Bertrand Marcheix^3^, Clément Delmas^2^

##### ^1^CHU, Toulouse, FRANCE; ^2^Intensive Care Unit, Rangueil University Hospital, Toulouse, FRANCE; ^3^Cardiovascular surgery department, Rangueil University Hospital, Toulouse, FRANCE; ^4^Vascular and Internal medicine department, Rangueil University Hospital, Toulouse, FRANCE; ^5^Thoracic surgery department, Rangueil University Hospital, Toulouse, FRANCE

###### **Correspondence:** Cecile Bouges - bouges.c@gmail.com

*Annals of Intensive Care* 2019, **9(Suppl 1)**:P-124

**Introduction**: Veno-venous (VV-ECMO) and veno-arterial (VA-ECMO) extracorporeal membrane oxygenation are increasingly used for the treatment of refractory acute respiratory distress syndrome, or cardiac arrest and refractory cardiogenic shock respectively. Our aims were to describe the prevalence of vascular complications following ECMO weaning and to identify the associated risk factors.

**Patients and methods**: From January 2014 to December 2017, 237 patients were managed by ECMO in our unit (Rangueil University Hospital, Toulouse). Among the 136 weaned patients, 81 benefitted from post-decannulation venous and arterial Doppler ultrasound to assess vascular complications, and were included in this retrospective study.

**Results**: Thirty-one patients (38.3%) showed vascular complications after ECMO cannula withdrawal- Twenty-two patients (27.2%) had arterial complications and 26 (32.1%) had venous complications. In 67.7% of the case, vascular complication were asymptomatic. Patients with vascular complications were younger (48 vs 56 respectively, p = 0.029). Patients with deep vein thrombosis (DVT) had longer ECMO duration (12.8 [7.25–15.5] vs 6 [4.5–9] days, p = 0.002) and prolonged ICU length of stay (31.5 [18.3–46.5] vs 17.0 [11.5–35.5] days, p = 0.037) compared to patients without DVT. Finally, prevalence of DVT was higher in patients with VV ECMO (50%) compared to patients with VA ECMO (23.6%, p = 0.018). Vascular complication occurrence tended to be associated with an increased mortality at 3 months (13% versus 27% p = 0.115).

**Conclusion**: VV and VA ECMO are associated with an elevated prevalence of vascular complication. Systematic doppler ultrasound evaluation after weaning could optimize management of these specific patients.

### P-125 Estimation of Pulmonary artery occlusion pressure assessed by tissue Doppler imaging in Ventilated and on catecholamine patients in ICU. A preliminary study

#### Nicolas Bonnet (*speaker*), Olivier Martin, Marouane Boubaya, Florent Poirson, Johanna Oziel, Yacine Tandjaoui-Lambiotte, Guillaume Van Der Meersch, Abdelaziz Bouguerba, Yves Cohen, Philippe Karoubi

##### Hôpitaux Universitaires Paris Seine-Saint-Denis - Hôpital Avicenne, Bobigny, FRANCE

###### **Correspondence:** Nicolas Bonnet - nb.bonnet@gmail.com

*Annals of Intensive Care* 2019, **9(Suppl 1)**:P-125

**Introduction**: Mitral velocity (E wave velocity and A wave velocity), and early diastolic mitral annulus velocity E’ assessed by transthoracic echocardiography (TTE) and tissue Doppler imaging (TDI) are correlated to pulmonary artery occlusion pressure (PAOP) in cardiologic patients. These parameters are dependent on loading conditions, modified by mechanical ventilation and the use of catecholamine. In ICU ventilated patients receiving catecholamine, E A ratio and E’ velocity has not been evaluated.

**Patients and methods**: This observational prospective study included 12 consecutives patients (mean age = 61 ± 15.3 years) with septic shock and ARDS. All patients were treated with catecholamines (mean dose (mg h) = 3.4 ± 3.0) and ventilated (mean FIO2 (%) = 0.8 ± 0.3, mean tidal volume (mL kg) = 6.2 ± 0.5). A volume expansion was performed with Ringer lactate solution at the discretion of clinician. Echocardiographic, Doppler examinations and hemodynamic measurements were repeated after volume expansion. We obtained 32 measurements. Doppler mitral inflow and TDI mitral annulus velocities were determined and compared with PAOP measured by a Swan-Ganz catheter. All TTE parameters were analyzed off-line by an independent operator, blinded to clinical history and PAOP values.

**Results**: Fluid challenge increased the ScVO2 (from 73.3 ± 10.1 to 76.0 ± 7.8%) and PAOP (from 11.2 ± 3.0 to 12.8 ± 3.4 mmHg). However, there was no difference in cardiac index (from 4.0 ± 2.1 to 4.0 ± 2.0 l min m^2^). There was no significant correlation between PAOP and the E wave (Beta 4.64 IC 95% [-0.18, 9.47] p = 0.079), E A ratio (beta 1.82, IC 95% [-2.21, 5.84] p = 0.39) and E E’ ratio (beta 0.94, IC95% [-2.56, 4.46], p = 0.61).

**Conclusion**: In this preliminary study, no significant correlation was found between E wave, E A ratio, E E’ ratio and PAOP in ventilated patients receiving catecholamines. More inclusions are needed to confirm or refute this result.

### P-126 Evaluation of perfusion practices in the central venous system in pediatric intensive care units in a teaching hospital

#### Chloé Levenbruck (*speaker*), Eve-Marie Thillard, Morgan Recher, Stéphanie Genay, Bertrand Décaudin, Pascal Odou

##### CHU, Lille, FRANCE

###### **Correspondence:** Chloé Levenbruck - chloelevenbruck@gmail.com

*Annals of Intensive Care* 2019, **9(Suppl 1)**:P-126

**Introduction**: The central venous catheter (CVC) allows to infuse several drugs simultaneously in intensive care units thanks to the use of Y-set infusion lines. Many constraints are encountered in the management of pediatric populations- limited infusion rates according to the age of children, maximum infusion volumes in relation to their weight. Indeed, a limitation of the water supplies has to be performed because of the hyper-permeabilization of the vessels in this population. So, optimization of infusion sets is therefore essential to improve and secure this support. Our main objective was to evaluate infusion practices and to identify optimization suggestions.

**Patients and methods**: Observation of infusion lines on CVC were conducted for 3 months (December 2017 to March 2018) in the pediatric resuscitation department. A restitution of these observations was carried out in the unit to form a working group to optimize the infusion setups. Several proposals for perfusion set will be discussed.

**Results**: Conducted interviews with nurses lasted approximately 5 to 10 min according to a grid composed of multiple-choice questions and open-ended questions. 32 infusion lines were observed and 14 nurses interviewed (34% of the nurses staff). This observation concerned 14 children under 1 year old, 12 children between 1 and 6 years old and 6 children over 6 years old. 37% of CVC were placed in jugular vein, 25% in femoral, 31% in subclavian and 7% other. There were 50% trilumen, 38% bilumen and 12% monolumen CVC. Only 51.9 ± 0.2% of available ports were used on infusion lines. CVC dressings were changed every 4 days or when they were soiled or unstuck. 71% of nurses declared problems with the dressing- dressings are too bigs for the youngers, inadapted for burned children, not yet sticky for those who drool. 79% of nurses have encountered drug incompatibilities during infusion through CVC but all surveyed nurses knew the existence of the unit’s drug incompatibilities table.

**Conclusion**: The evaluation of nurses practices highlighted a good knowledge of the types of infusion lines compared to the recommendations. However, efforts must be made to rationalize the number of lines available on the infusion set by integrating infusion devices with optimized geometry to limit drug incompatibilities. A discussion with the medical team about sizes of catheters according to the child weight will be engaged. Smaller dressings have been referenced in the unit and a modification in dessings change frequency was considered at 7 days.

### P-127 Risk factors for not meeting the recommendations for enteral nutrition in critically Ill children

#### Mylène Jouancastay (*speaker*)^1^, Camille Guillot^2^, Jean-Benoît Baudelet^3^, Morgan Recher^2^, Yasmin Karaca^2^, François Machuron^6^, Stephane Leteurtre^2^

##### ^1^Réanimation pédiatrique CHRU, Lille, FRANCE; ^2^Service de Réanimation pédiatrique, Hôpital Jeanne de Flandres et Université de Lille II, Lille, FRANCE; ^3^Service de Cardiologie pédiatrique, Institut Coeur Poumon et Université de Lille II, Lille, FRANCE; ^6^Unité de Méthodologie, Biostatistique et Data Management CHRU Lille, FRANCE

###### **Correspondence:** Mylène Jouancastay - mylenejouancastay@hotmail.com

*Annals of Intensive Care* 2019, **9(Suppl 1)**:P-127

**Introduction**: Malnutrition is prevalent in children admitted to the pediatric intensive care unit (PICU). Inadequacy energy intake is known to be a risk factor for morbidity and mortality in critically children. SFAR (Société Française d’Anesthésie et de Réanimation) published in 2014 European nutritional guidelines. This study aimed to evaluate nutritional practices in a French PICU by comparing energy and protein intake using SFAR’s recommendations and 2017 American recommendations and to identify risk factors of inadequate energy intake using SFAR’s recommendations.

**Patients and methods**: This study was retrospective in one PICU in a University’s Hospital from 2014 to 2016. Children aged one month to eighteen years old who were receiving an exclusive enteral nutrition (EN) were included. Patient hospitalized less than 48 h, patient fed exclusively by oral or parenteral route were excluded. Individual energy and protein intake were calculated for each day and compared to SFAR’s recommendations. Satisfactory energy intake was considered to be followed if equal to or greater than 90% of SFAR’s recommended intake. Two groups were constituted and compared to identify risk factors between patients following or not the SFAR’s recommendations- “optimal EN group” - children who received more than 90% of energy recommendation maintained for a least half of the ICU stay and “no optimal EN” if children didn’t.

**Results**: 418 patients were involved in this study. Malnutrition at admission occurred in 151 patients (36.6%). Average energy intake were 47.5 kcal kg day (IC95% = 27–62) that represented 75% and 87.8% of energy estimated intake by SFAR’s recommendations and Schofield equation respectively. Energy intake received was closer to intake estimated using Schofield equation than those recommended by SFAR (Figure 1). SFAR’s recommendations were respected for 43% of patients. The 2017 American recommendations were taken on board for 80% of patients. Average protein intake were 1.2 g kg day (IC95% = 0.6–1.5) that represented 67% of intake recommended by SFAR. 88 patients (21%) had an “optimal EN”. The median time to initiate EN was longer in “no optimal EN” group. Vasopressors were identified to be a risk factor of no meeting the SFAR’s recommendations (OR = 5.34, IC95% = 1–28.4, p = 0.04).

**Conclusion**: Only 43% of patients respected SFAR’s recommendations. Vasopressor was a risk factor of no meeting SFAR’s recommendations. The 2017 American recommendations using Schofield equation were respected in 80% of patients. An enteral feeding protocol could reduce the median time to initiate an EN support.


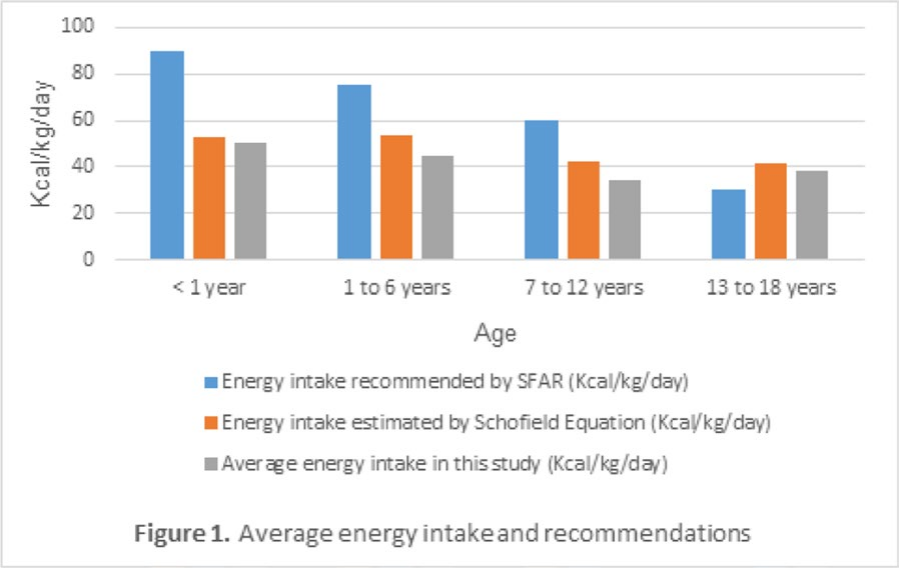


### P-128 Predicting hemodynamic intolerance to depletion by measuring the variation of the stroke volume index during a calibrated abdominal compression in pediatric ICU - a prospective study

#### Julie Hentzen (*speaker*), Matthias Jacquet-Lagrèze, Laurent Chardonnal, Capucine Didier, Sabine De Lamer, Dominique Bompard, Catherine Koffel , Jean-Luc Fellahi

##### CHU Lyon Louis Pradel, Bron, FRANCE

###### **Correspondence:** Julie Hentzen - julie.hentzen@sfr.fr

*Annals of Intensive Care* 2019, **9(Suppl 1)**:P-128

**Introduction**: Fluid overload (FO) is common in pediatric ICU particularly after septic shock or cardiac surgery and is responsible for acute kidney injury, decreased ventilation-free days, increased length of stay in ICU and generation of morbi-mortality. Predictors of hemodynamic intolerance (HI) to depletion for children don’t exist to our knowledge. Another study showed that calibrated abdominal compression (CAC) was able to predict fluid responsiveness in children with acute circulatory failure.

**Patients and methods**: The primary objective of our study was to determine if the variation of stroke volume index (SVi) during CAC -our test index- can predict HI to depletion defined as a decrease of 15% of cardiac output after depletion of at least 10 mL kg in children with FO. We conducted a prospective non-interventional study in a french teaching hospital with a cardiologic ICU. All patients under eight years old who might be suffering from FO and who needed depletion with diuretics were selected. We only included those who had urinated more that 10 mL kg in less that two hours. We assessed the SVi with the Aortic Flow Velocity Integral by transthoracic echocardiography before depletion, during the CAC and after depletion. We recorded other hemodynamic parameters considered as secondary outcomes.

**Results**: 47 patients were included after cardiac surgery. Only six had a decreased cardiac output by more than 15% after depletion. The area under the curve of the receiving operative curve (ROCAUC) of our test index was 0.47, 95%CI [0.23 + 0.73] (figure 1). Its median values of specificity, sensitivity, positive and negative predictive values were 0.58, 0.66, 0.21 and 0.93 respectively. The ÄSVi-CAC was not significantly correlated to the ÄSVi before and after depletion (r = 0.26, 95%CI [-0.03 + 0.51], p = 0.08). However, respiratory variations in aortic blood flow peak velocity (ÄVPeak) and variations of central venous pressure (CVP) during CAC appeared to be good predictors of HI to depletion with ROCAUC of 0.91, 95%CI [0.8 + 0.98] and 0.84, 95%CI [0.68 + 0.97] respectively (figure 1). Surprisingly, patients who have shown an HI to depletion had a profile of right ventricular failure instead of the expected profile of hypovolemia.

**Conclusion**: Our test index was inconclusive in predicting HI to depletion in children admitted in ICU, but other variables like ÄVPeak or change in CVP during CAC seemed to be superior.


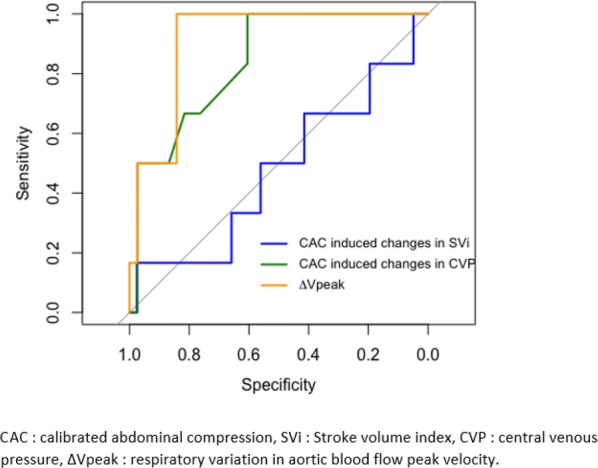


### P-129 PEDIATRIC SEPTIC SHOCK- time course and prognosis of organ dysfunctions according to different definitions

#### Luc Panetta (*speaker*)^1^, Stéphane Leteurtre^2^, Solenn Rémy^3^, Alain Duhamel^2^, Hélène Béhal^2^, François Machuron^2^, Florent Baudin ^4^, Etienne Javouhey^4^

##### ^1^HCL, Lyon, FRANCE; ^2^CHRU, Lille, FRANCE; ^3^Hospices Civils de Lyon, Groupement Hospitalier Est-Hôpital Femme Mère Enfant, Bron, FRANCE; ^4^Hospices Civils de Lyon, Bron, FRANCE

###### **Correspondence:** Luc Panetta - luc.panetta@hotmail.fr

*Annals of Intensive Care* 2019, **9(Suppl 1)**:P-129

**Introduction**: In 2016 an international task force changed the definitions of sepsis and septic shock in adults, using an organ failure score named the Sequential Organ Failure Assessment (SOFA). The concept of new and progressive multiple organ dysfunction syndrome (NPMODS), corresponding to organ dysfunction aggravation, has been proposed as proxy of mortality in some critical diseases. Many criteria exist to define these organ dysfunctions- the Proulx criteria, the Goldstein criteria, the Pediatric Logistic Organ Dysfunction-2 (PELOD2) score, and the Pediatric SOFA score. The aim of the study was to compare these four definitions with each other, to find the best definition of NPMODS that could be used as surrogate outcome in the pediatric septic shock therapeutic trials.

**Patients and methods**: This is a retrospective observational study, from January 2011 to December 2016, in a single PICU. Inclusion criteria were age < 18 years, hospitalization in PICU for septic shock (2005 definition). The primary outcome measure was PICU mortality. The secondary outcome was an evaluation of morbidity-mortality using the evolution of the Pediatric Overall Performance Category (POPC).

**Results**: 149 patients were included. Mean age was 5 years (± 5.5 years), 16 patients (10.7%) were less than 28 days old. 18 patients died (12.1% of fatality rate). 44 patients (29.5%) had a significant worsening delta-POPC. The most common causes of septic shock were meningitis or meningoencephalitis (15.8%), purpura fulminans (15.1%), and intraabdominal infection (15.1%). Patients developed NPMODS in 26.2% according to Proulx criteria, 30.9% according to Goldstein criteria, 30.2% for PELOD2, and 28.9% for pSOFA. The four definitions used to define NPMODS were significantly associated with mortality and delta-POPC (p < 0.001). The deceased patients did not have scores that worsened in the first five days, but the scores did not improve. On the contrary, the scores of survivors improved within the first 5 days whatever the definition used.

**Conclusion**: NPMODS definition could be a surrogate outcome of mortality, usable in pediatric septic shock therapeutic trials, whether using the Proulx, Goldstein, PELOD2 or pSOFA criteria. However, this definition only considers the onset or the aggravation of organ dysfunction. Further studies would be needed to create and validate an alternative definition, which incorporates the notion of non-improvement of organ dysfunction over time, which would better reflect the natural course of the most severe patients with pediatric septic shock.

### P-130 Comparison of cardiac outpout monitoring between electrical velocimetry and transthoracic echocardiography in the PICU. A pilot Study

#### Julien Baleine

##### CHU Montpellier, Montpellier, FRANCE

###### **Correspondence:** Julien Baleine- jf-baleine@chu-montpellier.fr

*Annals of Intensive Care* 2019, **9(Suppl 1)**:P-130

**Introduction**: Electrical velocimetry (EV) is a non-invasive method of continuous left cardiac output monitoring. The main objective was to validate EV by investigating the agreement in cardiac output measurements performed by EV and trans-thoracic echocardiography.

**Patients and methods**: We conducted a prospective single-center pilot study in the 8 beds pediatric intensive care unit (PICU). We included children from 28 days to 10 years, with hemodynamic instability or circulatory failure, and excluded children with congenital heart disease or impossible chest access impossible. We simultaneously measured cardiac output by EV and echocardiography. Agreement, bias and precision of the measurements were analyzed by the Bland–Altman method. Bias < 10% and percentage error < 30% were considered clinically acceptable. Parameters of contractility (ICON for EV and Left ventricular ejection fraction) and preload dependance (Stroke Volume Variation SVV for EV and variation in aortic blood flow velocity in echocardiography) were compared.

**Results**: 10 patients were included (median age 3.5 years [0.8–7], median weight 17.25 kg [5.36–28]), and 38 measurements were performed and analyzed. The bias and percentage error for cardiac output measurement were 0.46% and 21.73%, respectively. There was no correlation between the parameters assessing contractility (left ventricular ejection fraction in echocardiography and contractility index in EV) (r = 0.14, p = 0.38), or those evaluating the preload dependence (variation in aortic blood flow velocity in echocardiography and SVV in EV) (r = 0.193, p = 0.244).

**Conclusion**: EV appears to be a reliable method of continuous CO monitoring compared to trans-thoracic echocardiography, but has its limitations on contractility and preload dependence parameters. The CARDIOREAPED study will follow this pilot study + it will start in November 2018 and will focus on 50 children.


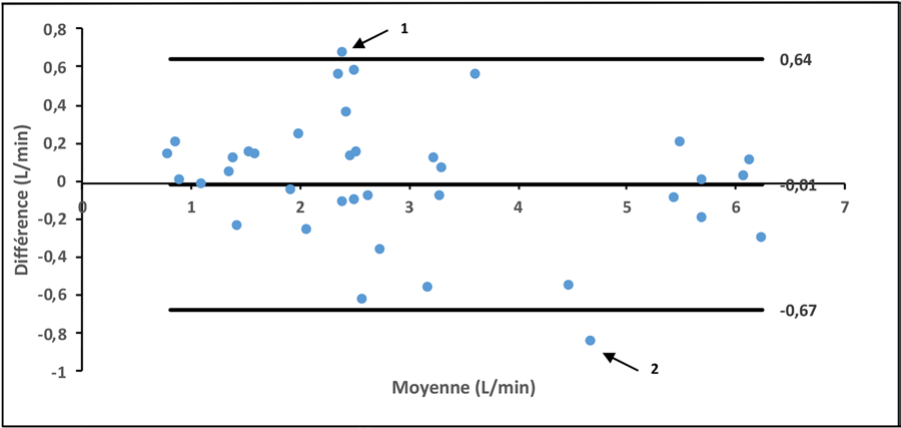


### P-131 Kawasaki Disease in intensive care unit in France - are complete and incomplete forms different?

#### Hélène Yager (*speaker*)^1^, Bilade Cherqaoui^1^, Fleur Lebourgeois^2^, Blandine Vanel^3^, Isabelle Kone-Paut^1^, Maryam Piram^1^

##### ^1^Hôpital Bicêtre, Paris, FRANCE; ^2^Hôpital Robert Debré, Paris, FRANCE; ^3^Hospices Civils de Lyon, Lyon, FRANCE

###### **Correspondence:** Hélène Yager - heleneyager91@gmail.com

*Annals of Intensive Care* 2019, **9(Suppl 1)**:P-131

**Introduction**: Kawasaki disease (KD) is a rare acute systemic vasculitis occurring mainly in children under 5 years of age. The major challenge is the cardiac involvement that may require admission in intensive care unit (ICU) and can be life-threatening. It is common that patients requiering ICU don’t meet all the American Heart Association (AHA) criteria, vascular damages being then often from the beginning complicated and the differential diagnosis - especially infectious - challenging. The risk is a delay of the treatment, in this case intravenous immunoglobulin (IVIg).

**Patients and methods**: The aim of the study was to specify the caracteristics and the outcome of patients admitted to ICU for a potential KD complication and comparing patients who met the AHA criteria (AHA +) with patients who did not (AHA-).

To that end, we collected national retrospective cases between 2001 et 2018, following a call from the scholarly associations (SOFREMIP, GFUP). We collected clinical, biological and echocardiography datas. Comparisons were based on Fisher’s exact test for categorical variables and the student’s t-test for continuous variables. Statistical analyses were performed using PRISM 5.0 and MedCalc + All tests were two-tailed, and P < 0.05 was considered significant.

**Results**: Patients who were not yet KD diagnosed at the admission to ICU presented either a sepsis in 25−48 (52%) (including toxic shock syndrom) or an acute abdomen in 12−48 (25%).

Nearly half of the patients received vasoactive drugs. All patients except two received broad-spectrum antibiotics. All patients received at least one IVIG infusion. Resistance to first IVIG infusion was observed in 24−48 patients (50%). A second IVIG infusion was chosen in all of them. A third treatment line was needed in 16−24 (66.7%)- IV corticoid infusion was chosen in 15−16, in association with anti-interleukin 1 in 4 15 (Anakinra). 25−78 (52%) patients KD-ICU presented the AHA criteria, compared with 23−48 (48%) who didn’t. Patients AHA- were younger, had a lower heamoglobine concentration and have been more intubated for mechanical ventilation.

**Conclusion**: KD-ICU patients had no initial or evolutive differences whether they had complete or incomplete AHA criteria. A strong suspicion of KD in ICU is therefore enough to initiate specific therapeutics such as IVIG. Further analysis will define the phenotype of patients with KD-ICU to target them earlier, comparing them to KD who did not need ICU transfer, via the KAWANET French database.


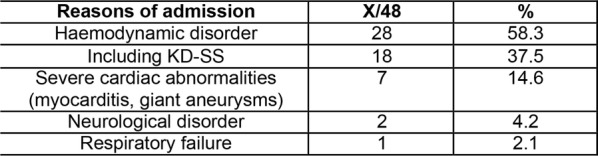


### P-132 Hyponatremia in critically ill infants with bronchiolitis - occurrence and associated morbidity

#### Florent Baudin (*speaker*), Annabelle Huget, Frédéric Valla, Etienne Javouhey

##### Pediatric Intensive Care Unit, HFME, Hospices Civils de Lyon, Lyon-Bron, FRANCE

###### **Correspondence:** Florent Baudin - florent.baudin@chu-lyon.fr

*Annals of Intensive Care* 2019, **9(Suppl 1)**:P-132

**Introduction**: Bronchiolitis is the first cause of hospitalization in infants. Six to 20% of them are admitted in a pediatric intensive care unit (PICU). Some will develop complications related to respiratory distress, like seizures and hyponatremia. The aim of our study was to describe the occurrence of hyponatremia in bronchiolitis infants under the age of three months admitted to PICU and the associated morbidity.

**Patients and methods**: We conducted a retrospective study including all infants younger than 6 months, admitted to Lyon-France PICU with a diagnosis of bronchiolitis between January 2010 and April 2018. Children with previous history of significant heart diseases or neurological impairment were excluded. The study was approved by the ethical committee of the French society of intensive care. Hyponatremia was defined as a plasma sodium level < 135 mmol/L at any time during PICU stay. T test and Chi2 were used to compare the hyponatremia group and the no-hyponatremia group. A p-value below 0.05 was considered significant.

**Results**: A total of 803 infants were included and among them, 726 had at least one available natremia value. hyponatremia occurred in 33.6% (n = 244) children and 6.8% (n = 50) had a natremia below 130 mmol/L. Age (36 + - 21 vs 36 + - 19 days in hyponatremia group, p = 0.83) and weight (3879 + - 884 g vs 3835 + - 784 g, p = 0.49) was similar between the two groups. Comparison on severity factors between the 2 groups are presented in table 1.

**Conclusion**: Hyponatremia occurred in one third of infant younger than 3 months years old admitted to PICU for bronchiolitis. Hyponatremia was associated with other severity factors of bronchiolitis, with more frequent seizures and with longer length of PICU stay. Hyponatremia needs to be monitored, prevented and treated accurately in infants with severe bronchiolitis.


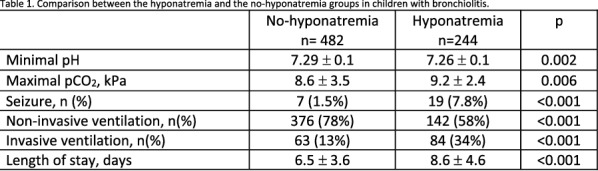


### P-133 Iatrogen hyponatremies in children- circumstances of survival

#### Samia Benouaz (*speaker*)^1^, Faiza Nadia Benatta^2^, Setti Aouicha Zelmat^2^, Djahida Djamila Batouche^2^, Ibtissem Bouanani^1^

##### ^1^Faculté de médecine, Sidi-Bel-Abbès, ALGERIA; ^2^Faculté de médecine, Oran, ALGERIA

###### **Correspondence:** Samia Benouaz - dr_benouaz@hotmail.fr

*Annals of Intensive Care* 2019, **9(Suppl 1)**:P-133

**Introduction**: Hyponatremia is a common fluid and electrolyte disorder. Its incidence is estimated at 0. 34% in operated children. 10% of hyponatraemia occur in the postoperative period. Objective- To determine the circumstances of occurrence of this hyponatremia.

**Patients and methods**: Retrospective study, from (2015–2017), carried out in the pediatric surgery and multipurpose resuscitation departments of CHU Sidi-Bel-Abbès. Included were all acute hyponatraemia, with or without neurologic symptomatology, following controlled surgery in healthy children and or with a pathology that did not lead to fluid and electrolyte disturbance.

**Results**: Of all the hyponatraemia identified during the study regardless of the mechanism, 17 children presented with iatrogenic acute hyponatremia. The children were operated on for circumcision (2), fracture reduction (6), orthopedic surgery (clubfoot and LCH) 7cas and thyroglossal cyst (2). The serum sodium ranged from 114 mmol/L to 128 mmo L. All patients presented with vomiting. Three children had generalized seizures. Brain CT found diffuse cerebral edema. The evolution was favorable in 16 17 cases with one death. In addition to anticonvulsant therapy in 03 patients, the treatment of hyponatremia involved the administration of sodium salts at a rate of 1.5 to 2 mmol hour to the PES during the 12 h under control of clinical status. and the ionogram.

**Discussion**: These children, who presented with iatrogenic acute hyponatremia, underwent simple surgeries, and were, with one exception, children of ASA1 class. They were all perfused in the perioperative period with hypotonic, high-throughput fluid. Blood glucose was moderately high, children were not hypovolemic on arrival. The circumstances of occurrence can be explained by-A dilution related to the infusion of hypotonic solution too abundant with a flow not monitored by our nurses due to lack of personnel. Prolonged preoperative fasting (more than 8 h) in our series that may be accompanied by hypovolemia responsible for the secretion of ADH, a component added to the iatrogenic factor.

**Conclusion**: Follow-up of good practices for perioperative pediatric perfusion, the use of adapted equipment (precision infusion pump, volumetric pumps), a strict control of the infusion rate, an education of the nursing staff and a permanent abandonment of pseudo prescriptions of the type to keep the vein are fundamental to avoid this type of ionic disorder in children postoperatively.

### P-134 Triage of severely injured children admitted to a Level 1 Pediatric Trauma Center- evaluation at different support stages

#### Sonia Courtil-Teyssedre (*speaker*)^1^, Lucile Genere^1^, Jean-Christophe Bouchut^1^, Blandine Gadegbeku^2^, Hélène Tardy^2^, Etienne Javouhey^3^

##### ^1^HCL de Lyon - HFME, Bron, FRANCE; ^2^I.F.F.S.T.A.R, Bron, FRANCE; ^3^HCL-HFME, Bron, FRANCE

###### **Correspondence:** Sonia Courtil-Teyssedre - sonia.teyssedre@chu-lyon.fr

*Annals of Intensive Care* 2019, **9(Suppl 1)**:P-134

**Introduction**: In traumatology, triage is a dynamic process that starts at the trauma scene including prehospital care, continues with trauma team activation in a trauma center when required and ends with patient admission to an adequate unit hospital. Referral of severely injured children to a level 1 Pediatric Trauma Center (PTC), like the Hôpital Femme Mère Enfant (HFME) (Lyon, France), reduces mortality. In HFME, local guidelines have been established for trauma team activation by prehospital carriers from the trauma scene or by emergency physicians from the emergency department (Level 1 criteria- vital distress, Level 2 criteria- vital distress stabilized after prehospital care, Level 3 criteria- no vital distress but mechanism of injury criteria).

**Patients and methods**: We conducted a retrospective study of 215 patients admitted to HFME in 2016 with trauma team activation criteria according to local guidelines. Level 1 or 2 patients, and level 3 patients presenting with an injury severity score (ISS) > 15, were considered severely injured. Four types of undertriage were defined at the different stages of support- prehospital undertriage «transport», prehospital undertriage «orientation», «emergency department undertriage» and «final undertriage», considered to be the main undertriage (Figure 1).

**Results**: Eighty eight patients were severely injured. The rates of undertriage were respectively- 23.8% for prehospital undertriage «transport», 15.7% for prehospital undertriage «orientation», 79% for «emergency undertriage» and 18.2% (16 88) for «final undertriage». Factor associated with «final undertriage» was firefighters prehospital care and transport (without medical transport team intervention)- OR 13.55 [2.16–85.00]. Median ISS was lower in the «final undertriage» subgroup- 17 [16–34] versus 23 [16–21] (p < 0.05) + «final undertriage» was not associated with a higher death rate or a longer hospital stay. However, 31.3% of this patients subgroup required critical care within the 24 h after admission.

**Conclusion**: The rate of undertriage in our PTC in 2016 is above recommendations of less than 5%. The prehospital triage seems to strongly impact the course of patient care. Evaluation of the traumatized child is complicated even for professional first-aiders. The high rate of «emergency department undertriage» shows that the triage also needs to be improved in emergency departments.


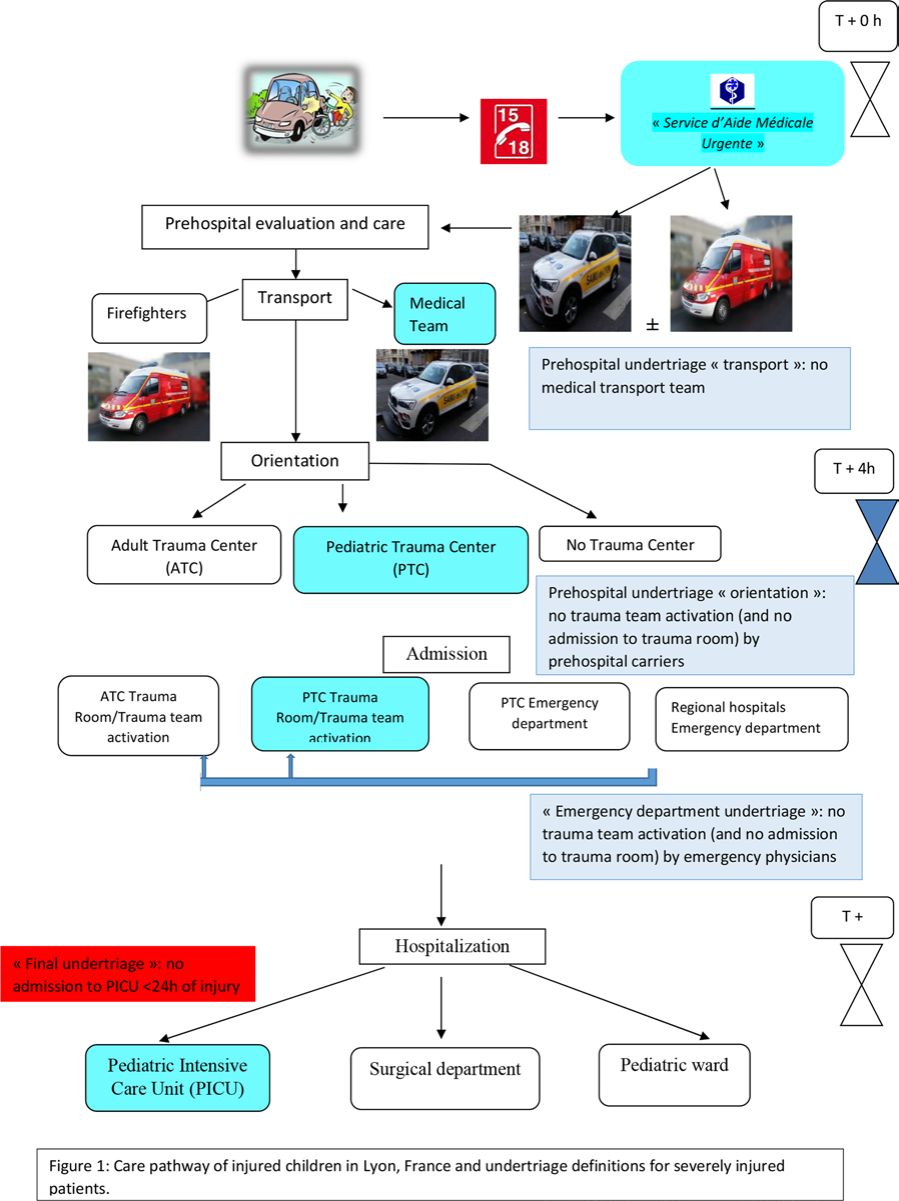


### P-135 Diaphragm and cardiovascular interaction during weaning from mechanical ventilation- a physiological study

#### Emmanuel Rozenberg (*speaker*), Maxens Decavèle, Elise Morawiec, Julien Mayaux, Julie Delemazure, Thomas Similowski, Alexandre Demoule, Martin Dres

##### APHP, Groupe Hospitalier Pitié-Salpêtrière Charles Foix, Service de Pneumologie, Médecine intensive – Réanimation, Paris, FRANCE

###### **Correspondence:** Emmanuel Rozenberg - emmanuelrozenberg@gmail.com

*Annals of Intensive Care* 2019, **9(Suppl 1)**:P-135

**Introduction**: Diaphragm dysfunction and weaning induced pulmonary edema (WIPE) are two main causes of weaning failure. Since spontaneous breathing-increased venous return is a key determinant of WIPE, we hypothesized that diaphragm dysfunction could not provide a thoracic depression enabling the occurrence of WIPE. Therefore, the objective of the study was to determine the prevalence of WIPE and diaphragm dysfunction and their coexistence.

**Patients and methods**: Patients intubated since more 48 h were eligible after they failed at a first spontaneous breathing trial (SBT). Before and after the following SBT, diaphragm function was evaluated with the reference method (drop in tracheal pressure induced by a bilateral phrenic nerves stimulation) and cardiac echo (early (E) over late (A) peak diastolic velocities ratio and tissue Doppler imaging of mitral annulus velocities including early (Ea) peak diastolic velocity over A wave ratio) and biological (hemoconcentration) markers of WIPE were searched for. Diaphragm dysfunction was defined by a Ptr, stim < -11 cmH20. WIPE was defined by a failed SBT associated with 1) either the combination of E A > 0.95 and E Ea > 8.5 at the end of the SBT or 2) an increase in plasma protein concentration or in hemoglobin > 5% during the SBT.

**Results**: Among 34 patients included, twenty-one (62%) failed in the SBT. WIPE was present in 15 21 (71%) patients who failed. Diaphragm dysfunction was found in 17 21 (81%) patients who failed and both mechanisms were present in 14 21 (67%) patients. Except in one patient, in all patients in whom WIPE occurred, diaphragm dysfunction was found (figure).

**Conclusion**: Coexistence of diaphragm dysfunction and WIPE is frequent at the time of liberation from mechanical ventilation in difficult to wean patients. The presence of diaphragm dysfunction doesn’t reduce the risk of WIPE.


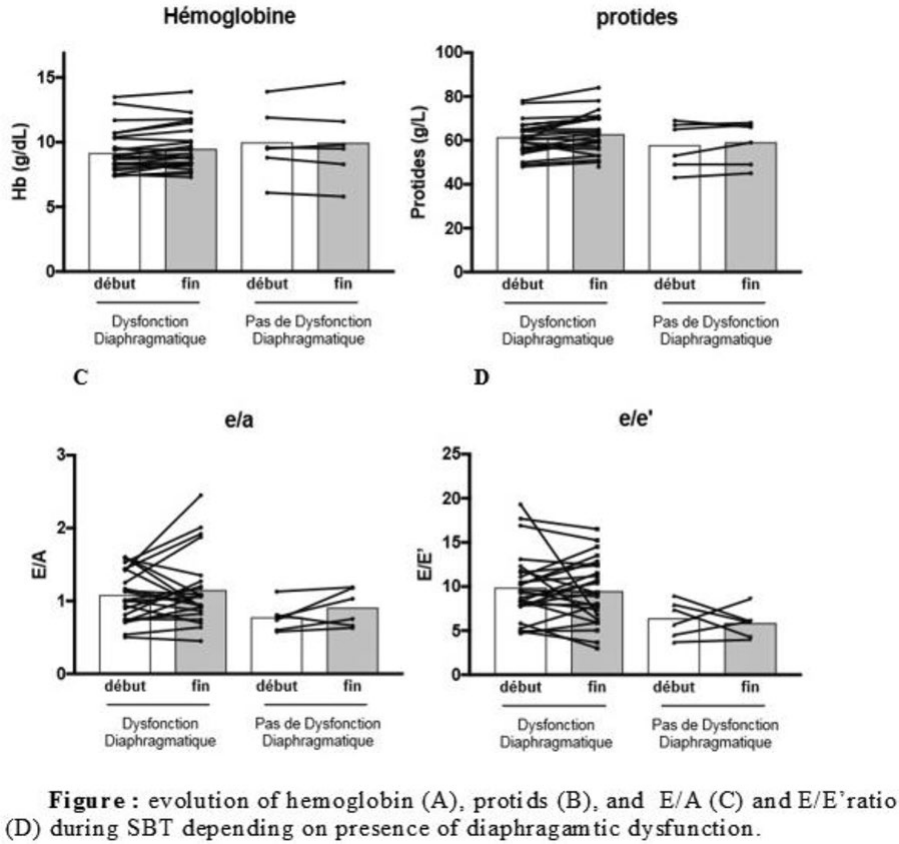


### P-136 Extracorporeal membrane oxygenation in adult cancer patients with severe acute respiratory failure- view of a cancer center

#### Afef Hammami (*speaker*)^1^, Aymen M’Rad^1^, Alain Gaffinel^1^, Lilia Berrahil-Meksen^1^, Annabelle Stoclin^1^, Bertrand Gachot^1^, Alain Combes^2^, François Blot^1^

##### ^1^Gustave Roussy, Villejuif, FRANCE; ^2^Réanimation médicale + GH Pitié-Salpêtrière, Paris, FRANCE

###### **Correspondence:** Afef Hammami - afef.hammami@gustaveroussy.fr

*Annals of Intensive Care* 2019, **9(Suppl 1)**:P-136

**Introduction**: Using extracorporeal membrane oxygenation (ECMO) in adult cancer patients is controversial. This study was designed to report outcomes of adult cancer patients treated with ECMO for severe acute respiratory distress syndrome (ARDS) after the failure of optimal conventional therapy. We also identified their pre-ECMO predictors of ICU and 6-month mortality.

**Patients and methods**: The charts of all cancer patients admitted to our oncology ICU and receiving ECMO support for ARDS from 2011 to 2017 were retrospectively reviewed. The cases were recorded using both PMSI data and analysis of computerized records by keywords systematic detection. After placement of cannulas, all patients were transferred from our hospital to a specialized ICU for ECMO therapy.

**Results**: Fourteen patients received ECMO during the study period (Table). One patient received venoarterial-venous ECMO because of acute circulatory failure in addition to ARDS. All other patients received venovenous ECMO. ECMO-related major bleeding and ventilator-associated pneumonia were frequent (50% and 42.9%, respectively). Respective median ECMO duration and ICU stay were 10 (5–43) and 27.5 (11–57) days. ICU and 6-month survival was 28.6%. The four survivors had a significantly shorter time interval between ICU admission and start of ECMO therapy (4 vs. 11 days), lower PCO2 at baseline (45 vs. 62 mmHg), and, after ECMO initiation, lower health care-associated infection (1 vs. 9 episodes) and shorter ECMO duration (6 vs. 19 days).

**Discussion**: The limitations of this study are its retrospective design and the small number of cases involved. Obvious selection bias was induced because only the patients treated with ECMO were included. The systematic computerized analysis ensured the lack of missing data. Despite the high rate of ICU mortality, this study showed that all survivors were still alive 6 months later. Early beginning of ECMO, avoiding ventilator-induced lung injury, was associated with a better prognosis. These findings encourage identifying as soon as possible patients with a favorable prognosis of malignancy and having an isolated respiratory failure, in order to optimize their chances of recovery from ARDS. A close cooperation of intensivists and oncologists is warranted.

**Conclusion**: ECMO therapy may be beneficial in selected cancer patients. Ongoing investigations are needed to better select patients with a likely benefit, and determine optimal timing for initiation of ECMO.


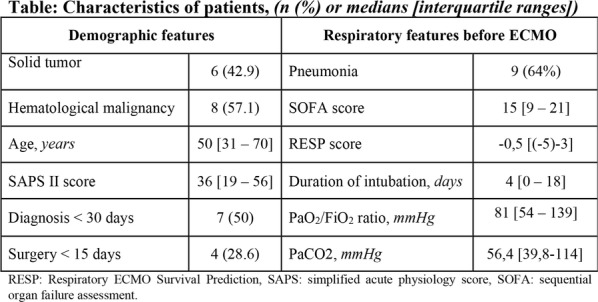


### P-137 Impact of a failed spontaneous breathing trial on dyspnea

#### Maxens Decavèle (*speaker*)^1^, Emmanuel Rozenberg^2^, Julien Mayaux^2^, Elise Morawiec^2^, Julie Delemazure^2^, Thomas Similowski^2^, Alexandre Demoule^2^, Martin Dres^2^

##### ^1^Hôpital La Pitié-Salpêtrière, Paris, FRANCE; ^2^Sorbonne Universités, UPMC Univ Paris 06, INSERM, UMRS_1158 Neurophysiologie respiratoire expérimentale et clinique. AP-HP, Groupe Hospitalier Pitié- Salpêtrière, Paris, FRANCE

###### **Correspondence:** Maxens Decavèle - maxencesar@hotmail.fr

*Annals of Intensive Care* 2019, **9(Suppl 1)**:P-137

**Introduction**: Dyspnea is a frequent and potentially intense symptom in mechanically ventilated patients. Whether the presence and intensity of dyspnea could interfere with the result of a spontaneous breathing trial (SBT) is unknown. Alternatively to the use of visual assessment scale of dyspnea that is challenging in critically ill patients, the five-item Mechanical Ventilation – Respiratory Distress Observation Scale (MV-RDOS) has been proposed as a reliable surrogate of dyspnea in non-communicative intubated patients [1]. In the present study, we sought 1) to describe the prevalence and changes in MV-RDOS during a SBT and 2) to evaluate the performance of MV-RDOS to predict SBT failure.

**Patients and methods**: Patients from a single center, intubated since more 48 h were eligible after they failed at a first spontaneous breathing trial (SBT). Dyspnea was assessed with the MV-RDOS at the beginning and the end of the SBT. Dyspnea was define by a MV-RDOS > 2.3 [1]. The area under receiver operating characteristic (ROC) curve of the MV-RDOS measured at the onset of the SBT was computed to predict the risk of SBT failure.

**Results**: Thirty-five patients (39 SBTs) (age 60 [49–68], SAPS II 71 [56–82] + med [IQR]) were included and in total 39 SBTs were analyzed. All patients were deemed ready to be weaned criteria (FiO_2_ 30% [30–40] + positive end expiratory pressure 5 cmH2O [5–6] + respiratory rate 22 cycles min [17–26]) but dyspnea was present in 22 39 (56%) at the beginning of the SBT. Twenty-three (59%) SBTs lead to failure and 16 (41%) were considered as success. The changes in MV-RDOS in patients who succeeded and failed the SBT are depicted in Figure 1. The proportion of SBT failure was higher in patients who presented dyspnea at the beginning of the SBT (74% vs. 31%, p = 0.008). A MV-RDOS value above 2.47 at the beginning of the SBT predicted SBT failure with a 65% sensitivity and 81% specificity (AUC = 0.778 + 95%CI [0.634, 0.927]).

**Conclusion**: Despite patients met classical readiness to wean criteria, dyspnea was frequent at the beginning of SBT and increased only in patients who fail the SBT. MV-RDOS could predict SBT failure with good performances.

**Reference**

1. Decavèle M, Gay F, Persichini R, Mayaux J, Morélot-Panzini C, Similowski T, Demoule A. The Mechanical Ventilation–Respiratory Distress Observation Scale as a surrogate of self-reported dyspnoea in intubated patients. Eur Respir J. 2018;52:1800598. 10.1183/13993003.00598-2018.

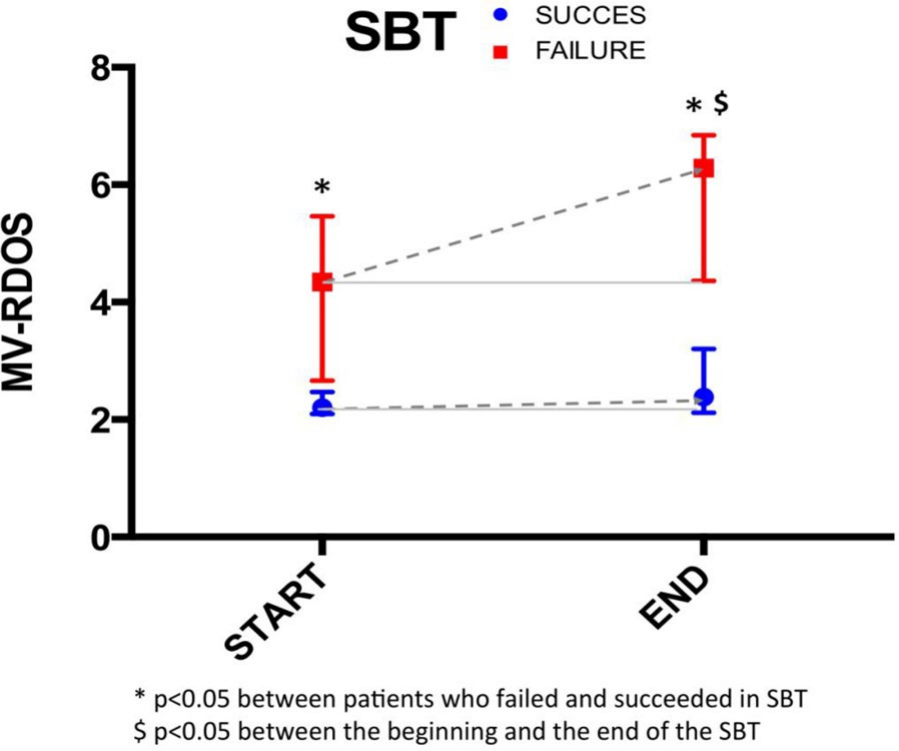


### P-138 Acute interstitial pneumonia of systemic lupus erythematosus

#### Emmanuelle Guérin (*speaker*)^1^, Marc Pineton de Chambrun^2^, Samia Boussouar^3^, Alexis Mathian^1^, Nicolas Bréchot^2^, Micheline Pha^1^, Guillaume Franchineau^2^, Ania Nieszkowska^2^, Loic Le Guennec^2^, Guillaume Hekimian^2^, Philippe Rouvier^4^, Miguel Hie^1^, Matthieu Schmidt^2^, Alain Combes^2^, Charles-Edouard Luyt^2^, Zahir Amoura^1^

##### ^1^Service de médecine interne 2, Institut E3 M, Hôpital La Pitié-Salpêtrière, Sorbonne Université, APHP, Paris, France, Paris, FRANCE; ^2^Service de médecine intensive-réanimation, ICAN, Hôpital La Pitié-Salpêtrière, Sorbonne Université, APHP, Paris, France, Paris, FRANCE; ^3^Service de radiologie cardiovasculaire diagnostique et interventionnelle, Hôpital La Pitié-Salpêtrière, Sorbonne Université, APHP, Paris, France, Paris, FRANCE; ^4^Service d’anatomopathologie, ICAN, Hôpital La Pitié-Salpêtrière, Sorbonne Université, APHP, Paris, France, Paris, FRANCE

###### **Correspondence:** Emmanuelle Guérin - emmanuelle.guerin@aphp.fr

*Annals of Intensive Care* 2019, **9(Suppl 1)**:P-138

**Introduction**: Systemic lupus erythematosus is a chronic autoimmune disease characterised by frequent skin, joint, haematological and renal involvement. Acute interstitial pneumonia (AIP) is infrequent and poorly investigated in this disease. The aim of this study was to describe the clinical characteristics, the course and the outcome of AIP in SLE.

**Patients and methods**: We conducted a monocentric retrospective, observational, cohort study between November 1996 and September 2018. We included all patients with SLE (defined using the American College of Rheumatology 1997 criterion) and AIP (defined as acute respiratory manifestation with diffuse pulmonary opacities on chest radiography or lung tomography and exclusion of alternate diagnosis including acute respiratory infection).

**Results**: Fourteen patients (male female sex ratio, 0.3 + mean ± SD age, 23.7 ± 10.6 years) presenting 16 episodes were included. AIP was present at disease onset or during the first year after SLE diagnosis in respectively 9 14 (64%) and 5 14 (36%) patients. Eleven (68.8%) episodes required ICU admission because of respiratory failure with 7 11 (63.6%) episodes requiring mechanical ventilation and 2 11 (18.2%) VV-ECMO. Median [IQR 25–75] SAPSII and SOFA score at ICU admission were- 32 [27–39] and 4 [2.5–8.5] respectively. Frequencies of associated SLE-related organ involvement were- kidney 13 16 (81.3%), arthritis 13 16 (81.3%), fever 13 16 (81.3%), serositis 12 16 (75%), skin 11 16 (68.7%), haematological manifestation 8 16 (50%) and neurological manifestation 3 16 (18.7%). Median [IQR 25–75] SLEDAI-2 K was 18.5 [14.75–25.75]. Bronchoalveolar lavage was available for 12 episodes and revealed alveolar haemorrhage in 8 12 (66.7%). Chest tomography revealed bilateral consolidations predominating in the lower parts of the lungs in most patients. SLE treatments were as follow- corticosteroid pulses 16 16 (100%), cyclophosphamides 9 16 (56%), plasmapheresis 4 16 (25%). Evolution was favourable in most patients and 90-day survival was 93.8%. During follow-up, only one patient had asymptomatic residual lung interstitial opacities while all other patient had normal lung CT-scan.

**Conclusion**: AIP is a rare manifestation of SLE that occurs during the first year of SLE onset in young patients. AIP of SLE can be severe, requiring mechanical ventilation and even ECMO. Patients usually exhibit multiple other SLE-related organ involvement. Mortality in our cohort is low and most patients recover completely without chronic interstitial lung disease during follow-up.

### P-139 Implication of unconventional T cells during severe pneumonia

#### Yonatan Perez (*speaker*)^1^, Florent Creusat^2^, Chloé Boisseau^2^, Mustapha Si-Tahar^2^, Christophe Paget^2^, Youenn Jouan^1^

##### - INSERM U1100, Faculté de Médecine de Tours + 2 - Service de médecine intensive et réanimation, CHU de Tours, Tours, FRANCE; ^2^1 - INSERM U1100, Faculté de Médecine, Tours, FRANCE

###### **Correspondence:** Yonatan Perez - yonatperez@gmail.com

*Annals of Intensive Care* 2019, **9(Suppl 1)**:P-139

**Introduction**: Severe pneumonia is frequently associated with acute respiratory distress syndrome (ARDS). Uncontrolled inflammatory response in the lung is a key factor in the transition from pneumonia to ARDS. However, the underlying mechanisms are still poorly understood. To assess this, a heterogeneous population of T lymphocytes called “unconventional T cells” (UTC) deserves greater attention. These cells comprise Natural Killer T (NKT) cells, mucosal-associated invariant T cells (MAIT) and Gamma Delta T cells. Pre-clinical studies have shown their versatile properties and their key role in immune responses against invading pathogens. Thus, we hypothesize that a tight regulation of their functions is mandatory to fine-tune the host inflammatory response in the infected lungs, and, subsequently to prevent emergence of an aberrant response leading to tissue damages. Despite this strong rationale, human data are however lacking.

**Patients and methods**: Single-center prospective study on patients hospitalized in intensive care for severe pneumonia. From blood and respiratory samples, we performed a flow cytometry-based analysis of these cells in order to determine- (1) their frequency at different time-points during hospitalization, (2) the presence of activating regulating markers (CD69 and PD-1), (3) their ability to produce cytokines involved in ARDS pathogenesis and (4) their cytotoxic capacity.

**Results**: 26 patients have been included to date, that were compared to healthy controls. Half of the patients presented ARDS, and median SAPSII was 35.5 (30–51). In patients with severe pneumonia, we observed a striking decrease in circulating MAIT cells, compared to healthy controls (Fig. 1A), but not for NKT or Gamma Delta T cells. This observation may suggest a recruitment of these cells to inflammatory site since a high proportion of MAIT cells can be detected in respiratory fluids of some patients with ARDS. In addition, circulating MAIT cells of patients expressed high levels of CD69 and PD-1 (Fig. 1B). Interestingly, the proportion of CD69 + MAIT cells decreased with clinical improvement, while proportion of PD-1 + remained stable. Upon ex vivo stimulation, proportion of IFN-gamma-producing MAIT cells was significantly decreased in patients, compared to healthy controls (Fig. 1C), while proportions of IL-17 and TNF-alpha-producing MAIT cells were similar.

**Conclusion**: MAIT cells are recruited, activated and have an altered cytokine profile secretion during severe pneumonia. These preliminary data justify pursuing in-depth analysis of MAIT cell functions in patients, in correlation with clinical condition.


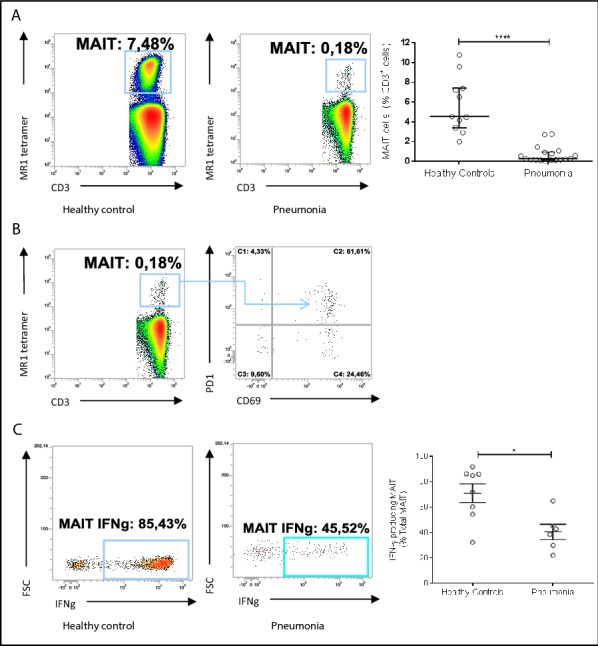


### P-140 Predictive factors for tracheal intubation in cancer patients presenting with de novo acute respiratory failure (ARF) and treated with high-flow oxygen through a nasal cannula (HFNC)

#### Morgane Tramier (*speaker*), Laurent Chow-Chine, Magalie Bisbal, Fredérique Gonzales, Luca Servan, Marion Faucher, Jean Manuel De Guibert , Antoine Sannini, Djamel Mokart

##### Institut Paoli Calmettes, Marseille, FRANCE

###### **Correspondence:** Morgane Tramier - tramier.morgane@gmail.com

*Annals of Intensive Care* 2019, **9(Suppl 1)**:P-140

**Introduction**: For cancer patients presenting with ARF the use of mechanical ventilation (MV) is frequent and associated with high mortality. Preventing endotracheal intubation appears to be a crucial step in the therapeutic strategy. Therapy using HFNC offers an alternative for those patients. The objective of this study was to determine the predictive factors of the endotracheal intubation in cancer patients presenting de novo ARF and firstly treated with HFNC in the intensive care unit (ICU).

**Patients and methods**: This retrospective study was conducted in a cancer referral center from 1st January 2012 to end 2016. Data were recorded from 301 consecutive critically ill cancer patients presented with ARF and treated with HFNC. Two groups were compared using non parametric tests- intubated patients versus those that were not. Then, independent predictive factors for tracheal intubation were determined using logistic regression.

**Results**: Two hundred nine patients presented with de novo ARF and were finally selected for analysis. The ICU mortality was 27.5% (n = 58), hospital mortality 43.5% (n = 91) and intubation rate 50% (n = 104). Median (IQR) age was 63 (53–69), male were 40% (n = 84), haematological disease was present in 68% (n = 142) of cases. By multivariable analysis, factors associated with tracheal intubation were- pulmonary infection (OR 2.5, 95%IC [1.1–5.3]), viral infection (OR 3.19, 95% CI [1.18–8.63]), multi-drug resistant bacteria infection (OR 4.9, 95% [1.6 -15]), SAPS II score (OR 1.04, 95% CI [1.01–1.07), Nb of pathologic X-chest quadrants > 2 at HFNC initiation (OR 6.6, 95% CI [3.1- 14.07]) as well as a cardiologic SOFA > 2 at the start of HFNC (OR 3.1, 95% CI [1.1–8.6]), FiO2 greater > 60% at the start of HFNC (OR 4.7[2.2–10]), and an SpO2 level < 95% 15 min after starting HNFC (OR 3.3, 95%IC [1.5 -7.1]).

**Conclusion**: For these specific population, intubation rate was about 50%. In this study intubation predictors are clinical parameters easily collected at bedside. In order to improve outcome, these parameters should be used to categorized high risk patients.


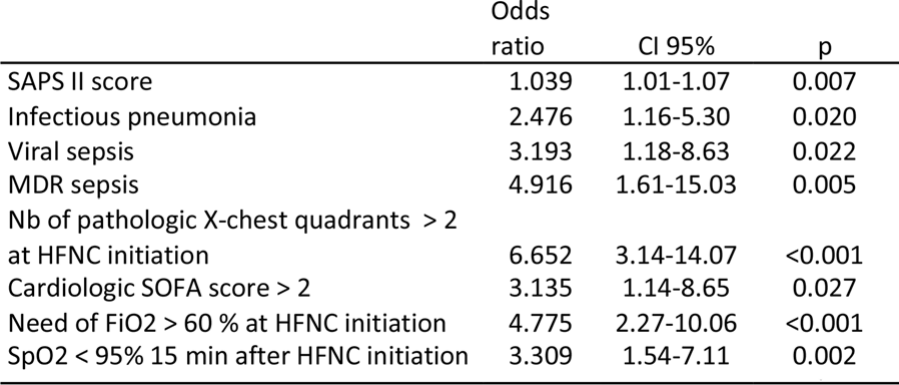


### P-141 Optimal positive end-expiratory pressure: Interest of Electrical Impedance Tomography for Veno-Arterial Extracorporeal Membrane Oxygenation–treated patients

#### Christelle Soule (*speaker*), Floriane Puel, Laure Crognier, Fanny Vardon, Jean Marie Conil

##### CHU TOULOUSE, Toulouse, FRANCE

###### **Correspondence:** Christelle Soule - christelle@iplanete.net

*Annals of Intensive Care* 2019, **9(Suppl 1)**:P-141

**Introduction**: Venoarterial ECMO-treated patients present many risk factors of breathing failure associated to heart failure. It seems essential to optimize the ventilatory parameters in order to limit lung injuries. The aim of this study is to evaluate the ability of electrical impedance tomography to set the optimal level of Positive End Expiratory Pressure (PEEP).

**Patients and methods**: We performed an alveolar recruitment maneuver followed by a decremental PEEP trial (from 20 to 5 cmH2O) with 5cmH2O decrements. Thanks to clinical and ultrasonographic parameters (heart and lung), we define an optimal PEP according to respiratory criteria (PEEPlung), then an adjustment according to hemodynamic and cardiac tolerances defines the PEPheart + lung. EIT datas (regional distribution of ventilation, z-compliance, overdistension collapsus ODCL and global inhomogeneity index GI) were recorded during the procedure and analyzed retrospectively to define the optimal PEEPs- PEEPCOMP, PEEPODCL, PEEPGI. A Friedman test compares the different optimal PEEPs between them, as well as the regional ventilation distribution at the 4 PEP levels. Agreement between PEEPlung and other measured PEEPs was analyzed by Cohen’s kappa coefficient.

**Results**: 23 patients were included and analyzed during a 9 months period. PEEP decreasing increments ventilation in the ventral region from 12 to 22% (corresponding to overdistension), and a decrease in the dorsal region from 11 to 5% reflecting collapse. The collapse (CL) is maximum at PEEP5 29% [21–45.8], and the distension is maximum at PEEP20cmH20 34% [24.5–40]. There is no significant difference between the different optimal PEEPs. The agreement is average between PEEPlung and optimal PEEP calculated from the EIT parameters (kappas 0.41 and 0.47). Our choice of PEEPlung is questionable because based on the global static respiratory compliance parameter that is measured on a single respiratory cycle. Measuring transpulmonary pressures could precise our measurements. PEPlung is statistically higher than the PEPheart + lung (p < 0.05), reflecting a bad hemodynamic tolerance of high levels of PEEP due to a complex phenomenon of heart–lung interactions.

**Conclusion**: EIT helps find the best compromise between overdistension and lung collapse, and detect regional distributions of the mechanical ventilation effects. However, evaluation of the chosen PEEP hemodynamic tolerance is necessary. The association EIT-echocardiography seems to be an efficient tool to provide monitoring of the PEEP effects.

### P-142 Comparison of occlusion pressure at 100 ms measured on Evita XL ventilator, at airway opening and from esophageal pressure in patients at the time of weaning from invasive mechanical ventilation

#### Claude Guerin (*speaker*), Mehdi Mezidi, Loredana Baboi, Nader Chebib, Floriane Lissonde, Hodane Yonis, Louis Kreitmann, Emilie Joffredo

##### Réanimation Médicale, Lyon, FRANCE

###### **Correspondence:** Claude Guerin - claude.guerin@chu-lyon.fr

*Annals of Intensive Care* 2019, **9(Suppl 1)**:P-142

**Introduction**: Airway pressure 100 ms after airway occlusion (P0.1) assesses the respiratory drive intensity and is measured at the mouth in pulmonary function tests lab. In ICU ventilators P0.1 may not reflect mouth P0.1 due to the compliance of the circuit. We aimed to compare P0.1 measured with the Evita XL ICU ventilator (P0.1, Evita) to airway (P0.1, aw) and esophageal P0.1 (P0.1, es) pressure in ICU patients, during a spontaneous breathing trial.

**Patients and methods**: We compared pressure support (PS) 7 cmH2O + PEEP 4 cmH2O (treatment A) to PS 0 cmH2O + PEEP 4 cmH2O + 100% automatic tube compensation (treatment B), each applied for 30 min. Before each treatment the baseline PS was applied for 30 min. Paw and flow were measured at the proximal tip of the endotracheal tube. Pes was obtained from esophageal balloon whose right position and optimal volume were checked properly. Three to ten P0.1 measurements were performed through the P0.1 built-in function available in the Evita XL ventilator, each separated by 4–8 breaths, in each condition in each patient. P0.1, Evita was read at the ventilator screen, and Paw, Pes and flow signals were recorded with BIOPAC150. Values are expressed as mean ± SD. Data were assessed by using Bland and Altman representation. A linear mixed effect model was used to assess the role on P0.1, Evita value of method of P0.1 measurement, rank of measurement and condition (as the fixed variables), the patient being taken as the random variable.

**Results**: Eighteen patients were included totalizing 280 measures. P0.1, es was discarded in 4 instances from 4 different patients for inaccuracy. P0.1, Evita averaged 2.8 ± 2.4, P0.1, aw 2.5 ± 2.1 and P0.1, es 2.5 ± 2.4 cmH2O. Bias and limits of agreement between P0.1, Evita and P0.1, aw and P0.1, Evita and P0.1, es were 0.3 (-1.5 ++2.1) and 0.3 (-3.2 ++3.9) cmH2O, respectively. Neither method used, rank of the measurement nor condition had a significant effect on P0.1, Evita (Table 1).

**Conclusion**: P0.1, Evita provides a reasonable estimate of P0.1 measured near to the patient.


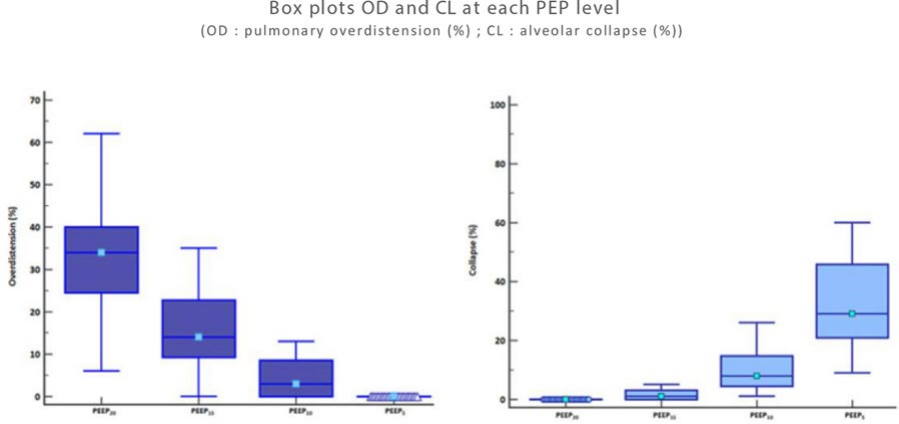


### P-143 Patients near to cardiogenic shock (CS) but without hypotension have similar prognosis when compared to patients with classical CS criteria: time for redefine CS? A FRENSHOCK multicenter registry analysis

#### Clément Delmas (*speaker*)^1^, Bruno Levy^2^, Nadia Aissaoui^3^, Etienne Puymirat^4^, Guillaume Leurent^5^, Vincent Labbe^6^, Sebastien Champion ^7^, Stéphane Manzo-Silberman^8^, Meyer Elbaz^9^, Nicolas Lamblin^10^,Laurent Bonello^11^, Edouard Gerbaud^12^, Patrick Henry^13^, Eric Bonnefoy^14^, Francois Roubille^15^

##### ^1^Rangueil University Hospital, Toulouse, FRANCE; ^2^Brabois Medical Intensive Care Unitl, Vandoeuvre-Les-Nancy, FRANCE; ^3^Medical Intensive Care Unit, Paris, FRANCE; ^4^Intensive Cardiac Care Unit, Cardiology department, Paris, FRANCE; ^5^Intensive Cardiac Care Unit, Cardiology department, Rennes, FRANCE; ^6^Intensive Care Unit, Paris, FRANCE; ^7^Intensive Care Unit, Le Chesnay, FRANCE; ^8^Intensive Cardiac Care Unit, cardiology department, Paris, FRANCE; ^9^Intensive Cardiac Care Unit, cardiology department, Toulouse, FRANCE; ^10^Intensive Cardiac Care Unit, Cardiology department, Lille, FRANCE; ^11^Intensive Cardiac Care Unit, cardiology department, Marseille, FRANCE; ^12^Intensive Cardiac Care Unit, Cardiology department, Pessac, FRANCE; ^13^Intensive Cardiac Care Unit, Cardiology department, Paris, FRANCE; ^14^Intensive Cardiac Care Unit, Cardiology department, Lyon, FRANCE; ^15^Intensive Cardiac Care Unit, Cardiology department, Montpellier, FRANCE

###### **Correspondence:** Clément Delmas - delmas.clement@chu-toulouse.fr

*Annals of Intensive Care* 2019, **9(Suppl 1)**:P-143

**Introduction**: Classical definition of cardiogenic shock (CS) combine a systolic blood pressure (SBP) < 90 mmHg with a low cardiac output and tissue hypoperfusion. By contrast, in practice, the spectrum of presentations is by far more complex. We compared presentation and prognosis between hypotensive and normotensive low cardiac output patient.

**Patients and methods**: FRENSHOCK was a multicenter, prospective, observational survey realized between 04 and 10.2016 in 48 centers in France. Patients were included if they met the criteria below (1) a low cardiac output (SBP < 90 mmHg and or need of amines, and or a low cardiac index < 2.2L min m2 on TTE or Swan-Ganz) + and (2) clinical, radiological, biological (NTproBNP or BNP), echocardiography, or invasive hemodynamics overload signs + and (3) a clinical and or biological hypoperfusion (lactates > 2 mmol/L, hepatic or renal failure).

**Results**: 772 patients were included (male 72%, median age 66y)- 678 with SBP < 90 mmHg (group A) and 94 with a proven low cardiac output without hypotension (group B). Group B patients have more chronic renal failure, more idiopathic dilated cardiomyopathy, more previous treatment by furosemide, and CS was more frequently caused by drug inobservance. They have less marbles, lower LVEF, more mitral insufficiency and higher pH but without significant difference for lactate. They were more treated by furosemide (95 vs 81%, p = 0.001), but less by dobutamine (71 vs 80%, p = 0.002), norepinephrine (21 vs 58%, p = 0.01), epinephrine (4 vs 13%, p = 0.012), invasive mechanical ventilation (15 vs 41%, p < 0.01), renal replacement therapy (3 vs 18%, p < 0.001) and circulatory support (11 vs 20%, p = 0.035). At 30-days no difference in mortality, heart transplantation and or VAD implantation was observed.

**Conclusion**: Normotensive patients with low cardiac output, overload and hypoperfusion signs present similar prognosis to classical CS. So, current definitions for CS could be challenged to avoid underestimate these patients.


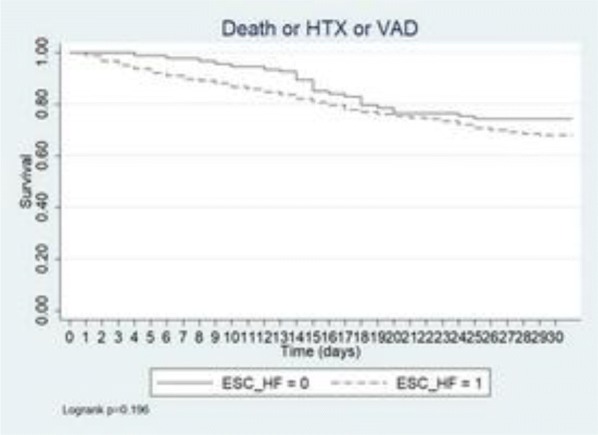



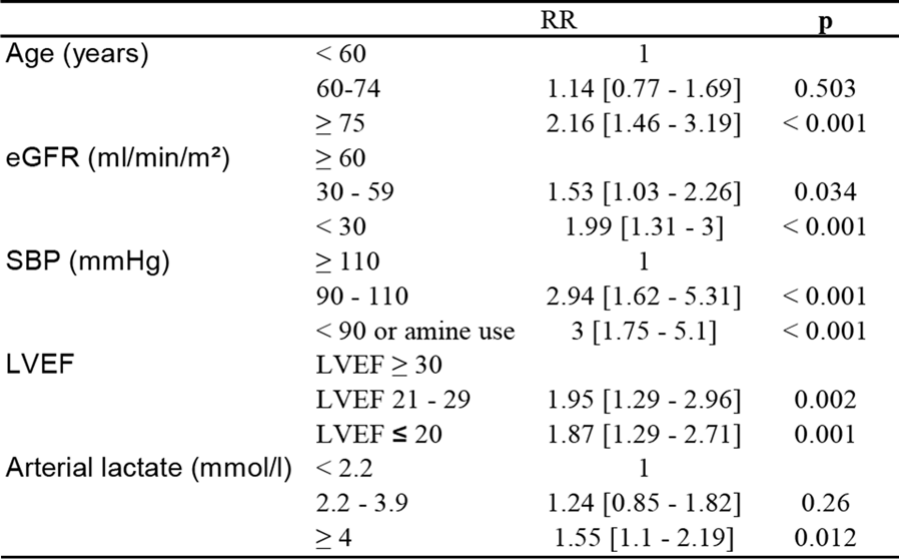


### P-144 Increase in central venous pressure during passive leg raising cannot predict preload unresponsiveness in critically ill patients

#### Olfa Hamzaoui^1^, Corentin Gouëzel^1^, Mathieu Jozwiak^2^, Maude Millereux^1^, BenjaminSztrymf^1^, Dominique Prat^1^, Frédéric Jacobs^1^, Xavier Monnet^2^, Christian Richard^2^, Pierre Trouiller^1^,Jean-Louis Teboul^2^

##### ^1^Service de réanimation polyvalente, Hôpital Antoine Béclère, Hôpitaux universitaires Paris-Sud, Assistance Publique-Hôpitaux de Paris, Clamart, FRANCE; ^2^Service de réanimation médicale, Hôpital Bicêtre, Hôpitaux universitaires Paris-Sud, Assistance Publique-Hôpitaux de Paris, Le Kremlin-Bicêtre, FRANCE

###### **Correspondence:** Olfa Hamzaoui- olfa.hamzaoui@aphp.fr

*Annals of Intensive Care* 2019, **9(Suppl 1)**:P-144

**Introduction**: Passive leg raising (PLR) is routinely used to predict preload responsiveness in critically ill patients. However, real-time measurements of cardiac output are required to assess its effects. Some authors have suggested that in fluid non-responders, central venous pressure (CVP) increased markedly (1). By analogy with the CVP rules proposed by Weill et al. to assess a fluid challenge (2), we hypothesized that an increase in CVP ≥ 5 mmHg during PLR can predict preload unresponsiveness by the absence of increase in velocity–time integral (VTI) of the flow in the left ventricular outflow tract by more than 10%.

**Patients and methods**: Critically ill patients with a central venous catheter in place and for whom the physician decided to test preload responsiveness by PLR were prospectively included. Transthoracic echocardiography was performed to obtain VTI. The CVP and VTI were measured before and during PLR.

**Results**: Forty-seven measurements were performed in 42 patients. Their mean age was 68 ± 16, their mean SAPSII was 49 ± 20. At baseline, blood lactate level was of 2.2 ± 2.5 mmol/L, MAP was 81 ± 13 mmHg, CVP was 9 ± 4 mmHg and VTI was 19 ± 4 cm. Thirty-nine patients received norepinephrine (mean dose: 0.42 ± 0.53 µg/kg/min). A positive response to PLR (was found in 20 cases. An increase in CVP ≥ 5 mmHg was measured in five of them (25%). A negative response to PLR was found in 27 cases. An increase in CVP ≥ 5 mmHg was measured in only four of them (15%). The changes in CVP during PLR between responders and non-responders were not different: 3.0 ± 2.0 mmHg and 2.7 ± 1.6 mmHg (p = 0.7), respectively. No correlation between the changes in CVP and the changes in VTI (r = -0.19; p = 0.7) was found. Neither the baseline CVP nor its change during PLR predicted preload unresponsiveness with areas under the receiver operating characteristic curves (with 95% CIs) being 0.55 (0.29–0.69) and 0.52 (0.38–0.68), respectively (fig 1).

**Conclusion**: Changes in CVP during PLR are not useful at the bedside to predict preload unresponsiveness in critically ill patients.

**References**

1. De Backer D, Vincent JL. Crit Care 2018; 22:43
2. Weil MH, Henning RJ. Anesth Analg 1979; 58:124–132.

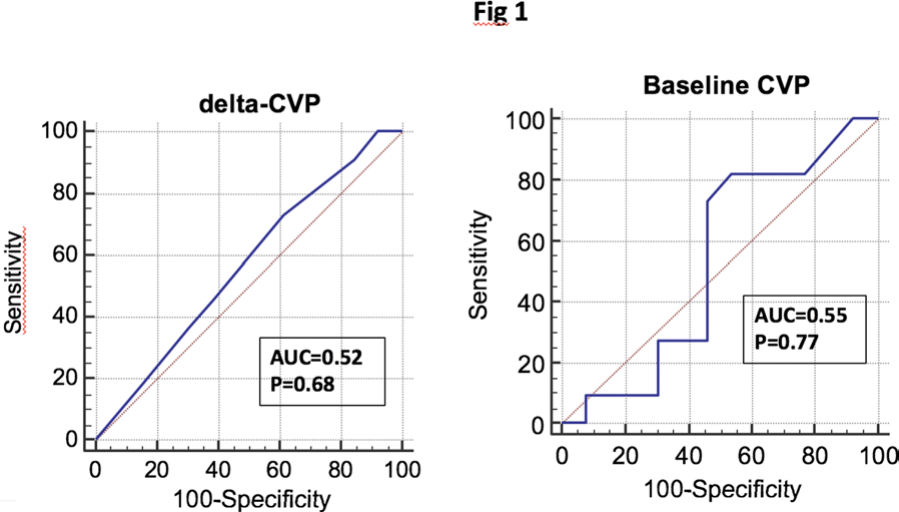


### P-145 Respiratory variation in IVC cross-sectional surface to predict fluid responsiveness - a prospective study

#### Ali Basbous (*speaker*)^1^, Didier Ledoux^2^, Jean-Luc Canivet^2^

##### ^1^CHU Liège, Herstal, BELGIUM; ^2^CHU Liège, Liège, BELGIUM

###### **Correspondence:** Ali Basbous - Ali.Basbous@alumni.uliege.be

*Annals of Intensive Care* 2019, **9(Suppl 1)**:P-145

**Introduction**: Adequate tissue perfusion in postoperative period is a priority after cardiac surgery. To insure this, optimizing cardiac output is required and can be obtained, among others, by volume expansion. However, it is crucial to distinguish patients who will respond to fluid challenge by improving their cardiac output from those who will not. Inferior vena cava (IVC) diameter variation is one of the several indicators proposed to predict fluid responsiveness. However, the efficacy of this indicator remains controversial. In this study, we tested the hypothesis that respiratory variation in IVC cross-sectional surface could be a better predictor of fluid responsiveness than IVC diameter variation.

**Patients and methods**: Prospective study in mechanically ventilated patients after cardiac surgery. A 500-ml crystalloid fluid challenge was administered over a 25-minute period of time. Collected data included pulse pressure variation, respiratory variation in IVC diameter and respiratory variation in IVC cross-sectional surface. The IVC was studied in a sub-xyphoidal view. Fluid responsiveness was defined as a 15% increase of the cardiac output obtained by Swan-Ganz catheter continuous thermodilution. Analyses were performed using parametric or non-parametric tests according to variables distribution. The ability of parameters to predict fluid response was assessed using ROC curves.

**Results**: Population of the study consisted of 31 consecutive patients aged 68 ± 10 years- 65% were male and the main types of surgeries were coronary artery bypass grafting (42%) and valvular surgery (32%). There were no statistical differences between responders and non-responders for static parameters including heart rate, central venous pressure, minimal and maximal IVC diameters. Respiratory variation in IVC diameter and IVC distensibility index were unable to predict fluid response as showed by an area under the ROC Curve (AUC) of 0.45 for both indicators. Discrimination power of pulse pressure variation was fair (AUC- 0.6). In our study, the best predictor of fluid responsiveness was the respiratory variation in IVC cross-sectional surface with an AUC of 0.7 (Figure 1).

**Conclusion**: The novel dynamic index obtained from cyclic variation of the IVC cross-sectional surface could be a candidate to predict fluid responsiveness. Consistently with previous studies, IVC diameters failed to distinguish fluid responders from non-responders. In our study, the fair ability of pulse pressure variation to predict fluid response could be explained by rapid changes in patients’ hemodynamics, as shown by the significant drop of systemic vascular resistance observed during the fluid challenge.


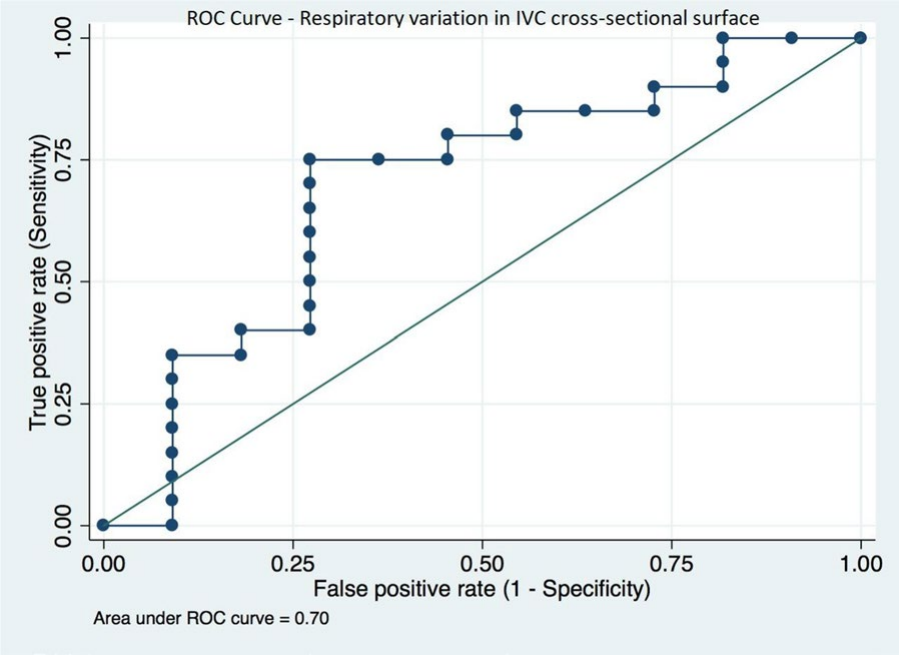


### P-146 Impact of smoking status on patients with septic shock

#### Sabrina Chaouech (*speaker*), Oussama Jaoued, Nejla Tilouche, Habiba Ben Sik Ali, Rim Gharbi, Mohamed Fekih Hassen, Souheil Elatrous

##### Hopital taher sfar mahdia, Mahdia, TUNISIA

###### **Correspondence:** Sabrina Chaouech- sabrina_chaouch_89@hotmail.fr

*Annals of Intensive Care* 2019, **9(Suppl 1)**:P-146

**Introduction**: Tobacco smoking is an important cause of preventable death worldwide. It predispose to infection by altering the immune system. Objective- to investigate whether smoking status had an impact on morbidity and mortality in patients with septic shock.

**Patients and methods**: This is a retrospective study conducted in the medical intensive care unit of a teaching hospital in Mahdia over a period of 4 years. All immunocompetent adults with septic shock were included. We adopted the Center for Disease Control and Prevention’s definition of current smoker (smoked ≥ 100 cigarettes and still smoked daily or quit within a year), former smoker (smoked ≥ 100 cigarettes and quit smoking more than 1 year) and never smoker (never smoked or smoked ≤ 100 cigarettes during his lifetime). We defined two groups- Tobacco group (current smoker and former smoker) and non-smoker group (never smoker). We recorded the demographic characteristics, co- morbidities, SAPSSII score, smoking status, site of infection, length of stay, duration of mechanical ventilation and mortality.

**Results**: A total 102 patients were included. The mean age was 64 ± 15 years and mean SAPSII score was 42 ± 16. Septic shock was caused by community-acquired infection in 68 (66%) patients. SOFA Score on first day of shock was 9.7 ± 3.5. Current smokers accounted for 22% of patients, former smokers represented 30% of patients and 48% of patients were never smokers. The mortality in current smokers, former smokers and never smokers was respectively 24.5, 23 and 43%.

Patients on tobacco group were older (67 ± 12 versus 60 ± 17, p = 0.017). Pulmonary infection was more common in Tobacco (40 53 versus 28 49, p = 0.05). Nosocomial acquired infection was more frequent in tobacco group (49% versus 16%, p = 0.001). Duration of mechanical ventilation and length of stay were longer in the Tobacco group, respectively 16[10–34] days versus 10[5–21] days p = 0.001 and 18[11–35] days versus 13[3–17] days p = 0.001. There was no significant difference in 28-day mortality between Tobacco and non-smoker groups (47% vs 57%, p = 0.31). In the multivariable analysis factors associated with mortality were age (OR = 1.048, 95% CI 1.005–1.093, p = 0.025) and use of ventilator assistance (OR = 12.8, 95% CI 1.105–148.548 p = 0.04).

**Conclusion**: In this study smoking status was not associated with mortality in patients with septic shock.

### P-147 Effects of prone positioning on venous return determinants and mean systemic pressure in patients with acute respiratory distress syndrome

#### Christopher Lai (*speaker*), Imane Adda, Jean-Louis Teboul, Laurent Guérin, Christian Richard, Xavier Monnet

##### Service de Médecine Intensive-Réanimation, Hôpitaux universitaires Paris-Sud, Hôpital de Bicêtre, APHP, Le Kremlin-Bicêtre, FRANCE

###### **Correspondence:** Christopher Lai- christopher.lai@hotmail.fr

*Annals of Intensive Care* 2019, **9(Suppl 1)**:P-147

**Introduction**: In acute respiratory distress syndrome (ARDS), prone positioning (PP) increases cardiac preload, but its effects on the determinants of systemic venous return (mean systemic pressure (Pms) and resistance to venous return (Rvr)) are unknown.

**Patients and methods**: We included nineteen patients with ARDS for whom it was decided to perform PP. Prior to PP, preload reserve was assessed by a passive leg raising test or an end-expiratory occlusion test. Hemodynamic measurements, including cardiac index (CI), central venous pressure (CVP), Pms and Rvr (with the heart–lung interactions method), and intra-abdominal pressure (IAP) were measured in semi-recumbent, supine horizontal (in 12 patients) and after 15 min of starting PP.

**Results**: PP significantly increased IAP (14 ± 4 to 19 ± 4 mmHg), Pms (24 ± 9 to 34 ± 11 mmHg) and Rvr (1.9 (1.4–2.8) to 2.8(2.3–3.7) mmHg.min.L-1). In 6 patients, CI increased ≥ 15% during PP. All these patients had preload reserve. Their Pms-CVP gradient increased by 74% (60%-176%) and the Rvr by 44% (33%-92%). In the other patients, CI did not increase ≥ 15% during PP. Ten of these patients had no preload reserve before PP. Their Pms-CVP gradient did not change during PP. The other three patients were preload-dependent at baseline. Their Pms-CVP gradient increased by 47%, but Rvr increased by 94%. The transition from the semi-recumbent to the supine horizontal position (studied in 12 patients) resulted in an increase of Pms without significantly modifying Rvr. In patients with preload reserve, this was accompanied by an increase in the Pms-CVP gradient and CI, unlike in patients without preload reserve.

**Conclusion**: PP increases Pms, CVP, Rvr and IAP. CI increases only if patients have preload reserve and if the increase of Rvr is less than the increase of the Pms-CVP gradient. The hemodynamic effects of PP are related to the increase of IAP during the transition to PP, but also to the increase of the Pms during the transfer from the semi-recumbent position to supine horizontal position.

### P-148 The implementation of the Ultrasound-guided subclavian catheterization in an intensive care unit is not associated with an increase of mechanical complication

#### Yoann Zerbib (*speaker*), Clément Brault, Dimitri Titeca Beauport, Loay Kontar, Anne Sagnier, Bertrand De Cagny, Thierry Soupison, Michel Slama, Julien Maizel

##### Medical Intensive Care Unit, BoReal Study group, CHU, Amiens, FRANCE

###### **Correspondence:** Yoann Zerbib - zerbib.yoann@chu-amiens.fr

*Annals of Intensive Care* 2019, **9(Suppl 1)**:P-148

**Introduction**: As the majority of Intensive care unit (ICU) we usually inserted our central venous catheters (CVC) in the jugular and femoral sites using the ultrasound guided technique. Since the recent publications describing the benefits of using ultrasound-guided technique for subclavian-vein catheterization and the reduction of bloodstream infections in subclavian site compare to jugular and femoral sites, we have modified our central venous catheters insertion protocol. Instead of privileging femoral and jugular sites we decided to implement a new protocol where the operator considered the three sites of insertions (femoral, jugular and subclavian) taking into account the clinical parameters (coagulation disturbance, obesity…) and the results of the ultrasound first look (visualization and diameter of the vein). The objective of our study was to compare the number of catheters placed in the subclavian position between the two periods and the rate of mechanical complications.

**Patients and methods**: We carried out an observational, retrospective, monocentric study in the intensive care unit in Amiens University Hospital between May 1st, 2015 and November 1st, 2016. The first period (jugular and femoral) lasted from May 2015 to January 2016 and the second period (jugular, femoral and subclavian) from February to July 2016. All procedures of CVC in adults patients were included. In our ICU all CVC procedures are reported in the patient record. Subclavian vein catheterization was done by ultrasound-guidance in the longitudinal axis and jugular or femoral in the short axis.

**Results**: Three hundred forty six procedures were included + 207 during the first period and 139 after protocol’s modifications. Number of catheters placed in subclavian site was greater during the second period (n = 36 (25%) vs 7 (3.4%) + (p < 0.0001) with a reduction jugular vein catheterization (139 (67.2) vs 67 (48.2), p = 0.0004) while the choice of femoral site was stable (P1 = 61 (29.4) vs P2 = 36 (25.9), p = 0, 4693) Subclavian vein catheterization was not associated with an increase number of mechanical complications (hematoma, pneumothorax, arterial puncture), only the number of malposition was significantly greater in the subclavian group (n = 3 (6.9%) vs n = 1 (0.3%), p = 0.0001). The two main reasons not to choose subclavian site were coagulation disorders (n = 33 103, 32%) and poor visualization of the vessel (n = 18 103 + 17%).

**Conclusion**: Implementing the ultrasound-guided subclavian catheterization is possible and was not associated with an increase of mechanical complications.

### P-149 Hypoproteinemia as a predictor of morbidity and mortality in elderly icu patients

#### Ghada Sbouii (*speaker*)^1^, Olfa Beji^2^, Rabia Atig^2^, Nesrine Baili^2^, Houssem Hmouda^2^

##### ^1^Yasminet hospital, Kairouan, ABKHAZIA; ^2^Hopital Sahloul, Sousse, TUNISIA

###### **Correspondence:** Ghada Sbouii - ghadasrlf@hotmail.com

*Annals of Intensive Care* 2019, **9(Suppl 1)**:P-149

**Introduction**: To examine the characteristics and the prognostic value of hypoproteinemia on ICU admission and at discharge in elderly critically ill patients.

**Patients and methods**: A retrospective study conducted in patients (≥ 65 years), from January 2015 to December 2016, admitted to Sahloul medical ICU. Patients were divided into normal, moderate and severe hypoproteinemia groups according to serum protein levels. The analysis included patients’ characteristics (age, gender, APACHEII, SOFA scores), the characteristics of pulmonary infections, (the onset, pathogens). The nutritional support, the length of stay, mortality in ICU and after discharge were analyzed.

**Results**: Fourty-six patients aged 75 ± 7 years with sex ratio F M = 1, 09 were investigated. The main causes of admission- acute respiratory failure (65.2%), septic shock (13%) and coma (10.9%). The mean APACHE II and SOFA scores were respectively 21 ± 8 and 7 ± 4. Twenty patients were mechanically ventilated during 3 days [1–34], four patients had tracheostomy during 27 ± 16 days. Enteral nutrition support was the frequent method of feeding (8 ± 10 days). At discharge, most patients still had moderate to severe hyporoteinemia (p = 0, 7). The highest APACHE II score was recorded in patients with moderate (21 ± 8, 6) and severe (25.6 ± 7.2) hypoproteinemia on admission (p = 0.048). Only 12 patients (26%) with moderate (n = 8) to severe (n = 4) hypoproteinemia contracted pulmonary infection (p = 0.005) within 6 ± 4 days. The type of pathogen had no significant relation with the level of hypoproteinemia (p = 0, 06). However, a trend to fungal infection was observed in these patients (candida albicans = 2 candida tropicalis = 2 candida glabrata = 1). The mean ICU LOS was 12 ± 11 days. It was longer among patients with moderate to severe hypoproteinemia (13 days versus 11 ± 6 days) compared to patients without hypoproteidemia (10 days), (p = 0, 6). The mortality in ICU was 21, 7%. Nine patients from 10 who died in ICU had moderate to severe hypoproteinemia with survivors who had normal serum protein level (p = 0, 21). Mortality after ICU discharge was 29, 6% (n = 8). The relation between patients who died post-ICU and the hypoproteinemia at discharge was statistically significant (p = 0, 02).

**Conclusion**: Hypoproteinemia at discharge can be a predictor of mortality in elderly patients admitted in ICU. Serum protein levels on admission can be used as an index to observe the illness severity and determine the prognosis of critically ill ageing patients.

### P-150 Is early administration of high dose of chloride associated with renal outcome in septic patients?

#### Xavier Chapalain (*speaker*)^1^, Thibault Balzer^1^, Olivier Huet^1^, Agathe Delbove^2^, Frédéric Martino^3^, Sophie Jacquier^4^, Cédric Darreau^5^, Marjorie Saint Martin^5^, Nicolas Lerolle^5^, Cécile Aubron^6^

##### ^1^CHRU de Brest, Département d’anesthésie réanimation, Brest, FRANCE; ^2^CHRU de Nantes, Médecine Intensive Réanimation, Saint-Herblain, FRANCE; ^3^CHU de Martinique, Réanimation Polyvalente, Fort-De-France, FRANCE; ^4^CHR d’Orléans, Médecine Intensive Réanimation, Orléans, FRANCE; ^5^CHRU d’Angers, Médecine Intensive Réanimation, Angers, Angers; ^6^CHRU de Brest, Médecine Intensive Réanimation, Brest, FRANCE

###### **Correspondence:** Xavier Chapalain - xavier.chapalain@gmail.com

*Annals of Intensive Care* 2019, **9(Suppl 1)**:P-150

**Introduction**: Fluid administration is a corner stone of early sepsis and septic shock management. Normal saline may induce hyperchloraemia. However, it remains controversial whether hyperchloraemia is associated with worse outcome in this setting.

**Patients and methods**: This study is a prospective, multicenter, observational study. All adults admitted to one of the 6 participating intensive care units (ICU) for septic shock and who received vasopressors infusion while non-intubated were included. Patients who were on chronic dialysis were excluded. Cumulative chloride dose administered within the first 48 h of septic shock management was calculated. The associations between high dose of chloride administration (more than 18 grams of chloride) and kidney injury defined as an increase in renal SOFA score of 1 or more during the first 2 days or RRT (Renal Replacement Therapy) requirement during the ICU (Intensive Care Unit) stay were studied. Between-group differences were tested with parametric tests (Student t test for continuous variables, Chi Square test for categorical variables). For multivariate analysis, an IPTW (Inverse Probability Treatment Weighting) methodology was performed to take into account for main confounders.

**Results**: A total of 239 patients were included over a one year study period. At ICU admission, the mean SOFA score was 9.59 (SD = 5.03). Day-28 mortality was 24.4% (n = 58). Considering cumulative chloride infusion within the first 48 h, 171 patients received more than 18 grams of chloride. After IPTW, there was no significant difference in use of RRT between patients who received high load of chloride versus low load (22.1% vs. 23.5% + RR = 0.85 + 95%CI, 0.55 to 2.40 + p = 0.58). There was no difference in persistent kidney injury between groups (33.8% vs. 43.3% + OR = 0.75 + 95%CI, 0.48 to 1.20 + p = 0.23). In univariate analysis, ICU length of stay (11.9 days vs. 12.7 days + p = 0.92) and 28-day mortality (26.5% vs. 23.5% + p = 0.74) did not differ between both groups.

**Conclusion**: In this cohort of patients with septic shock, high dose of chloride administered within the first 48 h of management was not associated with renal failure or RRT requirement. High dose of chloride infusion was also not associated with other outcomes, including 28-day mortality. Therefore, resuscitation with normal saline could be used safely in this setting.

### P-151 Withdrawal syndrome in chronically baclofen-treated rats- description and reversal

#### Bruno Megarbane (*speaker*)^1^, Solène Palmieri^2^, Pierre François Rogliano^2^, Lucie Chevillard^2^, Nadia Benturquia^2^, Patricia Risède^2^, Marion Soichot ^3^, Laurence Labat^3^

##### ^1^Hôpital Lariboisière, réanimation Médicale et Toxicologique, Paris, FRANCE; ^2^INSERM UMRS-1144, Paris-Descartes University, Paris, FRANCE; ^3^Toxicology laboratory, Paris, FRANCE

###### **Correspondence:** Bruno Megarbane - bruno.megarbane@lrb.aphp.fr

*Annals of Intensive Care* 2019, **9(Suppl 1)**:P-151

**Introduction**: Baclofen, a GABAB receptor agonist, is increasingly used to manage alcohol dependence, leading to increasing poisonings. Preclinical and clinical studies point out the onset of withdrawal syndrome following baclofen cessation. In the baclofen-poisoned patient admitted to the intensive care unit, withdrawal syndrome rapidly follows toxicity with intricate features and difficult diagnosis. Our objectives were to characterize baclofen withdrawal in the rat and study its reversal using different common drugs.

**Patients and methods**: The study was conducted in a Sprague–Dawley rat model, pretreated with repeated and increasing doses of baclofen from 5 to 15 mg kg 3 times daily during 15 days, using neurobehavioral (locomotion, anxiety, and memory tests) and electroencephalography (EEG using Markand’s scale [1]) investigations to evidence withdrawal syndrome and the effectiveness of the different treatments (topiramate, diazepam, gamma-hydroxy-butyrate [GHB] and baclofen, daily administered at pharmacological doses from day 5, as evidenced to be the peak of withdrawal syndrome) used to reverse its deleterious effects.

**Results**: Baclofen withdrawal resulted in marked encephalography (peaking at grade 3 of Markand scale), anxiety (p < 0.05) + but no significant effects on locomotion and memory were observed. Topiramate and diazepam administration led to the reversion of baclofen withdrawal-induced encephalopathy. GHB administration at the tested dose resulted in additional signs of toxicity.

**Conclusion**: Baclofen withdrawal is responsible in the rat for marked encephalopathy with possible beneficial reversion using topiramate and diazepam. However, the exact molecular mechanisms involved remain to be investigated.

**Reference**

1. Markand ON. Electroencephalography in diffuse encephalopathies. J Clin Neurophysiol1984 + 1- 357-407

### P-152 Hyperlactatemia in the acutely ethanol-poisoned patient- what is the direct role of ethanol?

#### Bruno Megarbane (*speaker*), Elmire Chauvière

##### Department of Medical and Toxicological Critical Care, Lariboisière Hospital, Paris, FRANCE

###### **Correspondence:** Bruno Megarbane - bruno.megarbane@lrb.aphp.fr

*Annals of Intensive Care* 2019, **9(Suppl 1)**:P-152

**Introduction**: Acute ethanol poisoning represents a frequent cause of admission to the emergency department and intensive care unit (ICU) in France. Ethanol is theoretically able to increase the serum lactate concentration. However, hyperlactatemia in the acutely ethanol-poisoned patient has been rarely reported and its exact mechanism is still debated. We aimed to determine the prevalence and describe the etiologies of lactate elevation following excessive ethanol ingestion.

**Patients and methods**: We conducted an retrospective single-centre observational study including all patients admitted to the ICU during 7 years (2011–2018) for acute drunkenness (defined by ethanol concentration > 1 g L, in the absence of significant drug co-ingestions) and who presented hyperlactatemia (defined by lactate concentration > 2 mmol/L).

**Results**: Fifty-four patients [16 females and 38 males + median age, 41 years (inter-quartile interval, 26) with past history of chronic alcoholism, 52%] were included, representing 54% of the patients admitted for acute drunkenness to the ICU during the same period. Patients were comatose [Glasgow coma score, 3 (4)] requesting tracheal intubation (81%). Complications included aspiration pneumonia (41%), sepsis (26%), cardiovascular failure (13%), vomiting (9%), seizures (6%), hospital-acquired infections (2%) and death (6%). Blood ethanol concentration was 3.4 g L (2.3) on admission and plasma lactate concentration 2.5 mmol/L (0.9) on admission, peaking at 2.9 mmol/L (1.1), with no significant correlations between both parameters (R2 = 0.006 and 0.03, respectively). The main reasons for increase in lactate concentration were sepsis (26%), hypovolemia (19%) and trauma (9%). The existence of underlying alcoholic cirrhosis explained 2% of the hyperlactatemia cases. In 30% of the cases, no etiology except ethanol was found to explain hyperlactatemia onset. In these patient subset, peak lactate concentration was significantly less elevated (2.4 mmol/L (0.9) vs. 3.0 mmol/L (1.6), p = 0.02) and decrease in serum bicarbonate more limited (23.4 mmol/L (1.6) vs. 22.0 mmol/L (4.5), p = 0.05).

**Conclusion**: Acute drunkenness can be complicated by hyperlactatemia. In ~ 30% of the cases, elevation in serum lactate can only be attributed to the presence of ethanol in the body. Metabolic investigations should be conducted to better characterize the involved mechanism, one hypothesis being the disequilibrium induced by ethanol in the intracellular NADH NAD + ratio.

### P-153 Flecainide poisoning- outcome and usefulness of plasma flecainide concentration on admission

#### Bruno Megarbane (*speaker*), Katia Carvalho-Alves

##### Department of Medical and Toxicological Critical Care, Lariboisière Hospital, Paris, FRANCE

###### **Correspondence:** Bruno Megarbane - bruno.megarbane@lrb.aphp.fr

*Annals of Intensive Care* 2019, **9(Suppl 1)**:P-153

**Introduction**: The acute poisoning with flecainide, a 1c anti-arrhythmic drug of the Vaughan-Williams’ classification is rare but may be responsible for life-threatening consequences. Our objectives were 1)- to report the features, complications and management of flecainide poisonings admitted in the intensive care unit (ICU) + 2)- to investigate the prognostic contribution of the plasma flecainide concentration measured on admission.

**Patients and methods**: We conducted a retrospective single-centre observational study including al flecainide poisoned-patients admitted to the ICU during a 20-year period (1998–2018), as evidenced by at least one plasma flecainide concentration in the toxic range. Plasma flecainide concentration was measured using liquid chromatography coupled to mass spectrometry. Identification of the predictive factors of death was performed using an univariate analysis (Chi-2 and Mann–Whitney tests, as required).

**Results**: Forty-eight flecainide-poisoned patients (54% males and 46% females + age, 53 years [32 + 58] (median [percentiles 25 + 75])) were included. The patients had ingested 3.0 g [1.7 + 3.0] of flecainide (including 1 3 with slow-release formulations) and were admitted 3.0 h [1.9 + 7.6] post-ingestion. They developed cardiovascular failure with membrane stabilizing effect on the ECG (58%). Plasma flecainide concentration was 2.3 mg L [1.3 + 3.0] on ICU admission. Management included mechanical ventilation (77%), catecholamine (81%), 8.4% sodium bicarbonate (63%), activated charcoal (54%), defibrillation (23%) and veno-arterial ECMO (3%). Ten patients (21%) died. Based on an univariate analysis, the non-survivors presented more marked hypotension (p = 0.05), bradycardia (p = 0.02), elevation in plasma lactate concentration (p = 0.04), elevation in transaminases (p = 0.006) and decrease in serum bicarbonate concentration (p = 0.04) than the survivors. The presumed ingested dose (p = 0.02) and the plasma concentration of flecainide on admission (p = 0.0003) were significantly correlated to the onset of death.

**Conclusion**: Flecainide poisoning is responsible for acute cardiovascular failure due to membrane stabilizing effects leading to a high mortality rate. The plasma flecainide concentration on admission is highly predictive of the onset of death and its bedside availability should be strongly encouraged. The identification of the clinical prognosticators is helpful when deciding to implement ECMO in the flecainide-poisoned patient.

### P-154 Valproic acid poisoning in the intensive care unit- outcome and analysis of the usefulness of L-carnithine infusion

#### Bruno Megarbane (*speaker*), Philippe Nguyen

##### Department of Medical and Toxicological Critical Care, Lariboisière Hospital, Paris, FRANCE

###### **Correspondence:** Bruno Megarbane - bruno.megarbane@lrb.aphp.fr

*Annals of Intensive Care* 2019, **9(Suppl 1)**:P-154

**Introduction**: Valproic acid (VPA) overdose causes life-threatening disorders including hyperammonemia and hyperlactatemia. L-carnitine has been used for several years as antidote to reverse VPA toxicity without evidence of effectiveness. We aimed 1)- to describe the features and outcome of VPA-poisoned patients admitted to the ICU and 2)- to investigate the effects of L-carnitine treatment on VPA pharmacokinetics (PK) and its contribution to the patient prognosis.

**Patients and methods**: We conducted a retrospective single-center cohort study. All consecutive patients admitted to the ICU for acute VPA intoxication (defined as presence of compatible symptoms with plasma VPA concentration > 90 mg L) between February 1998 and December 2016 were included. L-carnithine was administered according to the physician in charge. VPA elimination half-lives were determined using a non-compartmental approach. Population-based model for VPA PK and individual pharmacodynamics (PD) were obtained. The effects of L-carnitine were investigated using multivariate analysis in a propensity score matching study.

**Results**: Sixty-nine patients were included. The most frequent presentation was consciousness impairment (70%) with multiorgan failure (SOFA score, 4 [1–6] (median [percentiles 25–75]). The most frequent biological impairment was increase in lactate concentration (2.9 mmol/L [1.8–4.2]). Patients treated with L-carnithine presented significantly higher peak lactate concentration (4.6 mmol/L [3.0–6.1] vs. 3.5 [2.5–4.9], p = 0.002) and more severe neurological disorders (79% vs. 34%, p = 0.001) but not significantly different VPA concentrations. However, based on the propensity score approach, the only difference associated with L-carnithine administration was plasma lactate on admission (p = 0.001). When comparing matched L-carnithine-treated and non-treated patients, no significant benefit of L-carnithine was demonstrated using the difference between the worse and the admission values of the SOFA score, the delay in plasma lactate normalization and the VPA half-life. The pharmacokinetic parameters of the well-fitted population-based model of VPA PK in overdose were Ka of 0.5 h-1, Ke of 0.03 h-1, and half-live of 4.2 h.

**Conclusion**: VPA poisoning is responsible for life-threatening features including hyperlactatemia. Treatment using L-carnithine did alter neither VPA elimination nor patient outcome in the ICU. Our PK and PD models appear helpful to improve the management of the VPA-poisoned patients by allowing the identification of the conditions of inter-individual variability.

### P-155 Acute poisoning- factors associated with mortality in intensive care

#### Mohamed Walid Mhajba (*speaker*)^1^, Hela Maamouri^2^, Wided Derwich^2^, Nozha Brahmi^2^

##### ^1^Centre d’Assistance Médicale Urgente (CAMU), Tunis, TUNISIA; ^2^Réanimation polyvalente CAMU, Tunis, TUNISIA

###### **Correspondence:** Mohamed Walid Mhajba - mhajbawalid@hotmail.fr

*Annals of Intensive Care* 2019, **9(Suppl 1)**:P-155

**Introduction**: Acute intoxication is a common reason for admission to emergency care and resuscitation services. The objective of this study was to determine prognostic factors related to acute intoxication for a young population.

**Patients and methods**: This is a retrospective study which includes acute intoxications admissions to the intensive care unit for the year of 2017. Epidemiological, biological and therapeutic parameters were collected and an uni following by a multi-varied analysis were performed to determine the independent factors of mortality.

**Results**: During the study period, 762 patients were admitted to intensive care for acute intoxication. The average age was 31 ± 14 years old. When intoxication was voluntary in order to commit suicide, the most incriminated toxicants were drugs (n = 486 + 64%) especially psychotropic drugs (n = 284, 58%) and pesticides (n = 179 + 23.5%) with chloralose as a leader (n = 137 + 76%). For accidental poisoning, carbon monoxide (CO) ranked first (n = 56 + 7.3%). The clinical picture was dominated by coma (n = 292, 38%) and digestive disorders (n = 129, 17%). The IGSII and APACHE II admission severity scores were 16 ± 6 and 8 ± 3, respectively. During the stay, the main complications were inhalation pneumonia in 102 cases (30% of rodenticide poisoning), shock in 50 cases (7.8% of drug poisoning) and rhabdomyolysis in 44 case (18% of CO poisoning). Nosocomial infection was observed in 14 patients (2%).

Therapeutic management was mainly symptomatic with the use of mechanical ventilation in 290 cases (38%), antibiotics in 14% of cases and vasoactive amines in 7% of cases. Overall mortality was 1.4% of all acute poisonings (n = 11). The independent mortality factors were- a greater score than 18 (OR = 2 + p < 0.001) for APACHE II, the occurrence of hepatic cytolysis (OR = 1.3 + p = 0.001), an acute renal failure (OR = 1.2 + p < 0.001), recovered cardio-respiratory arrest (OR = 2.4 + p < 0.001) and length of stay more than 05 days (OR = 1.4 + p < 0.001).

**Conclusion**: Although mortality from acute intoxication is low, it is essential not only to raise awareness of the dangers of poisoning by certain cardiotoxic drugs, but also to legislate the sale of insecticide products.

### P-156 Minoxidil- from a hair lotion to a toxic shock in intensive care unit

#### Takoua Khzouri (*speaker*), Hela Maamouri, Nasreddine Foudhaili, Ikram Benjaberi, Nozha Brahmi

##### Camu, Tunis, TUNISIA

###### **Correspondence:** Takoua Khzouri - takoua_kh2@yahoo.fr

*Annals of Intensive Care* 2019, **9(Suppl 1)**:P-156

**Introduction**: Minoxidil is commonly used for the treatment of androgenic alopecia and has been wrongly considered safe. Several poisoning cases mainly pediatric proved that the ingestion of a few milliliters can lead to significant intoxication. In this perspective, we carried out this to illustrate the different characteristics of this poisoning in order to emphasize on its gravity when it is wrongly used and to improve its management.

**Patients and methods**: It was an observational retrospective study spread over nine years from 1st January 2010 to 22th September 2018 in a toxicological ICU, including all patients admitted for acute Minoxidil poisoning.

**Results**: During the study period, 12 patients were eligible for minoxidil poisoning which accounted 0.13% of all the acute poisonings requiring hospitalization in our Intensive Care Unit. Patients were aged of 42 years [17, 71]. Only one patient was a male. All Exposures were single-drug and 92% of them were accidental. The supposed ingested dose was unknown in all the cases. The delay of consultation was of 9 ± 6 h after ingestion. Gastrointestinal decontamination was not performed at all. Tachycardia was constant with an average of 120 beats per minute [92, 150]. Vomiting was present in 7 patients (58%). Nine patients (75%) presented hypotension requiring crystalloids resuscitation with an average of 1650 ml [1000, 2000]. Nine patients required vasopressor support by norepinephrine with an average of 1.5 mg h [0.5, 5.5] and a weaning delay of 2 days [1, 4]. Five presented myocardial suffering with decreased ST segment in all cases, negative waves in two times and three of them had cardiogenic pulmonary edema with an average pro-PNB level of 4080 pg mL [1680,700]. No one had coronary angiography immediately and only one had anti-ischemic treatment. All patients performed favorably and were discharged from the ICU after a mean length stay of two days. Three patients who suffered from cardiogenic edema have been referred to a cardiology department for exploration.

**Conclusion**: As it was shown, Minoxidil is an unsafe product because of its vasodilator action which may be responsible for prolonged tachycardia and profound hypotension requiring vasopressor support. Manufacturers should enhance packaging security and the over-the-counter availability must be questioned.

### P-157 Gamma-hydroxybutyric acid and gamma-butyrolactone poisonings- features and usefulness of the plasma gamma-hydroxybutyric acid concentration measurement

#### Bruno Megarbane (*speaker*), Charlotte Heliodore

##### Department of Medical and Toxicological Critical Care, Lariboisière Hospital, Paris, FRANCE

###### **Correspondence:** Bruno Megarbane - bruno.megarbane@lrb.aphp.fr

*Annals of Intensive Care* 2019, **9(Suppl 1)**:P-157

**Introduction**: Gamma-hydroxybutyric acid (GHB) and gamma-butyrolactone (GBL) poisonings have been reported to increase for a few months in France. GHB, an anesthetic compound, has been classified as Schedule III controlled substance due to its recreational abuse potential and use for sexual assault. GBL, a GHB precursor, is legally marketed as solvent although responsible for the same neurological toxicity and abuse liability. Our objectives were 1)- to describe the clinical features of acute GHB GBL poisonings + 2)- to investigate the predictive value of the plasma GHB concentration on admission + 3)- to study the relationships between the plasma GHB concentration and the coma depth.

**Patients and methods**: We conducted a retrospective monocentre observational study including all GHB GBL-poisoned patients admitted to the intensive care unit (ICU) during7 years (January 2011 to July 2018). Plasma GHB concentration was measured using liquid chromatography coupled to mass spectrometry. We determined the GHB pharmacokinetic parameters, studied the correlation between the plasma GB concentration and coma depth on admission, and modeled the relationships between the plasma GB concentrations and the Glasgow coma score (GCS) in each poisoned patient when possible.

**Results**: One hundred and thirteen GHB GBL-poisoned patients (14 females 99 males, aged of 33 years [16] (median, inter-quartile interval) were included. An increase trend was observed in the number of GHB GBL and mainly GBL poisonings. On ICU admission, the patients presented deep consciousness impairment (GCS of 3 [4]), with marked hypotonia (36%), osteotendinous reflexe abolition (47%) and myosis (48%), marked hypothermia (35.5 °C [1.4]) and increase in plasma creatinine phosphokinase (235 UI L [903.25]). Treatment was supportive including mechanical ventilation (76%). Outcome was favorable with rapid wake-up and short hospitalization (17 h [21]). Three major complications were noted including aspiration pneumonia (36%), rhabdomyolysis (9%) and withdrawal syndrome (2%). No significant correlation between the coma depth and the plasma GHB concentration were observed on admission (R2 = 0.015). Individual modeling of the GCS plasma GHB concentration relationships allowed recognizing the role of ethanol co-ingestion and tolerance acquisition to GHB due to its chronic use.

**Conclusion**: GHB GBL poisonings are increasing in the ICU with life-threatening presentations. Despite the well-fitted sigmoidal relationships between the GCS and GHB concentration at each individual level, inter-individual variability in the expression of GHB-induced neurotoxicity exists, mainly related to ethanol co-ingestion and tolerance acquisition.

### P-158 Analgesia in chest trauma - Serratus plane block by non-anesthesiologist physicians- a cadaveric feasibility study

#### Matthias Huck (*speaker*), Thomas Leclerc, Justine Simonet, Elisabeth Falzone, Clément Hoffmann

##### Burn and trauma center - Percy Military Teaching Hospital, Clamart, FRANCE

###### **Correspondence:** Matthias Huck - matthias.huck@yahoo.fr

*Annals of Intensive Care* 2019, **9(Suppl 1)**:P-158

**Introduction**: Analgesia is an essential component of chest trauma management to prevent medical complications, particularly respiratory ones. Serratus plane block (SPB) is a recent ultrasound-guided regional anesthetic technique which has proved capable of providing effective analgesia in chest trauma. The main objective of the study was to assess the feasibility of the SPB by non-anesthesiologist physicians.

**Patients and methods**: A cadaveric feasibility study was conducted in the anatomy lab of the Faculty of Medicine of Lyon Est. All participating physicians were voluntary and specialized in emergency medicine. They had to perform one SPB on a corpse by injecting a methylene blue solution. The pleura was previously filled with eosin red solution. The primary endpoint was the success of the block, the composite of 5 items- correct identification of anatomy, respect of safety rules, no pleural puncturing (aspiration of red solution), validation of procedure after checking by ultrasound and anatomical dissection (presence of methylene blue solution in the anatomic space between the Latissimus dorsi and the Serratus anterior).

**Results**: Twelve emergency physicians participated in the study, 10 were military ones. Each doctor realized one SPB. 10 out of 12 SPB were successful. The obtained results are summarized in Table 1. There was no pleural puncturing.

**Conclusion**: This study shows that the SPB is achievable by emergency physicians. This easy and safe anesthetic block provides effective analgesia for chest trauma. Its dissemination in emergency departments and in prehospital setting would improve early multimodal analgesia, including in the absence of available anesthetists.

### P-159 Eclampsy about 63 cases

#### Setti Zelmat (*speaker*)^1^, Djamila-Djahida Batouche^1^, Nadia Faiza Benatta^1^, Samia Benouaz^2^

##### ^1^Faculte de medecine, Oran, ALGERIA; ^2^Faculté de médecin, Sba, ALGERIA

###### **Correspondence:** Setti Zelmat - Settiaouichazelmat@yahoo.fr

*Annals of Intensive Care* 2019, **9(Suppl 1)**:P-159

**Introduction**: Eclampsia is a serious complication of pre-eclampsia, It is an obstetric emergency with a significant maternal–fetal morbidity and mortality, that can occur before during or after childbirth. Our study aims to describe the epidemiological and clinical profile + women with eclampsia in the gynecological obstetrics department of Oran EHU.

**Patients and methods**: We carried out a single-centric prospective study including all women who had been treated for eclampsia, in the obstetrics and gynecology department of the Oran EHU. (Level 3 maternity) over a period of one year from January 1st, 2017 to December 31st, 2017.

**Results**: 63 cases of eclampsia were recorded over this period with an incidence of 0.3%. Eclampsia occurred in antepartum in 90% of cases, postpartum in 10%, and 82% of postpartum eclampsia convulsed in the first 24 h, others occurred late at 6th day, 7th day and even at 60th day. The epidemiological profile is that of a young woman (64% were under 29 years old), primipara (68%), not followed (77%), 24 patients developed a complication, mostly a HELLP syndrom (21 patients, 33, 3%). Various treatments were put in place- Magnesium sulphate (80%), Nicardipine (85%), Alpha methyl dopa (100%), Nifedipine (90%). The main route of delivery was caesarean section 80% under general anesthesia, in our study we deplored no maternal deaths, perinatal death was 20%.

**Discussion**: Eclampsia has become a rare complication in developed countries thanks to early management of one of the main signs of preeclampsia. On the other hand, the surveillance of pregnancies by a qualified health staff, the screening of pregnancies at risk and the information of the patients made it possible to make this pathology regress. However, it remains common in our developing countries (1.35% in our series) and this prevalence is almost constant in sub-Saharan Africa.

**Conclusion**: the improvement of the maternal–fetal prognosis, during the eclampsia, rests mainly on- The detection and the early diagnosis of the severe forms, an early and adequate care, as well as on the availability of a multidisciplinary team.


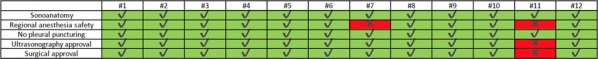


### P-160 Post traumatic cerebral venous thrombosis in 22 cases

#### Sabrine Bradai (*speaker*)^1^, Aziza Talbi^2^, Moez Kammoun^1^, Manel Zekri^1^, Kamilia Chtara^1^, Rania Ammar^1^, Olfa Turki ^1^, Mabrouk Bahloul^1^, Mounir Bouaziz^1^

##### ^1^Department of Intensive Care, Habib Bourguiba University Hospital, Sfax, TUNISIA; ^2^Departement of Emergency Medicine, Habib Bourguiba University Hospital, Sfax, TUNISIA

###### **Correspondence:** Sabrine Bradai - Sabrine.bradai2@gmail.com

*Annals of Intensive Care* 2019, **9(Suppl 1)**:P-160

**Introduction**: Cerebral venous sinus thrombosis (CVST) following a cranial trauma constitutes a rare but serious clinicopathological entity, described in literature along with the lack of consensus regarding diagnosis and management. The aim of this study is to point out the incidence of CVST after head trauma, its clinical presentation and course.

**Patients and methods**: It is a retrospective descriptive study, conducted at the intensive care unit (ICU) of HabibBourguiba university hospital, Sfax, Tunisia, between January 01, 2016, and December 31, 2017. All patients who were victims of polytrauma during the study period were enrolled. They underwent full-body computed tomography (CT). Additional CT or MRI angiographies were performed in patients presenting radiological suspicion of CVST.

**Results**: During the study period, 452 patients were admitted to the ICU department for polytrauma. Among those patients, we included 22 patients with the diagnosis of post traumatic CVST (4.8%). The sex ratio was 10. The mean age was 33.1 ± 15.9. On ICU admission, mean SAPSII was 30.7 ± 11.7 and mean SOFA score was 5.6 ± 2.8. Causes of trauma were dominated by traffic injury in 18 patients. Mean Glasgow Coma Scale score was 9.8 ± 4 (median- 10). Extracranial injuries were found in 10 patients. The brain CT scan presented- subarachnoid hemorrhage in 19 patients, basilar skull fractures in 16 patients, cranial vault fractures in 15 patients, cerebral contusion in 16 patients, subdural hematoma in 10 patients, extradural hematoma in 7 patients and diffuse axonal injury in 7 patients. The CSTV was diagnosed fortuitously on full-body CT in 14 patients. In the other 8 cases- 2 were diagnosed on MRI and 5 in control CT angiography. The CSVT was located in the transverse sinuses in 15 patients, the internal jugular vein in 10 patients, the superior sagittal sinus in 4 patients and cavernous sinus in 2 patients. Intravenous unfractionated heparin was used in 17 patients with a mean dose of 161.7 mg ± 54.5 mg per day (range 100–300 mg). Low molecular weight heparin was used in 5 patients. The delay of anticoagulation was 3 ± 1.4 days (range 0–6 days). The outcome was favorable in 20 patients.

**Conclusion**: Although, head trauma is a rare cause of CVST it should be considered in any head traumatic patient who develops symptoms of increased intracranial pressure. To date, initiation of anticoagulation stills a matter of debate because of the risk of worsening traumatic haemorrhage.

### P-161 Prognostic impact of early hyponatremia in patients with severe brain injury

#### Olfa Turki (*speaker*), Amal Triki, Kallel Hela, Mounir Yousfi, Bradii Sabrine, Mariem Dlela, Karama Bouchala, Chelly Hedi, Bahloul Mabrouk, Mounir Bouaziz

##### Departement of intensive care Habib Bourguiba University Hospital, Sfax, TUNISIA

###### **Correspondence:** Olfa Turki - olfa.turki.rea@gmail.com

*Annals of Intensive Care* 2019, **9(Suppl 1)**:P-161

**Introduction**: Hyponatremia (defined as serum sodium < 135 mEq L) is the most common electrolyte abnormality in traumatic brain injury (TBI) and is also an independent predictor of poor neurologic outcome. We encounter hyponatremia frequently in our practice, and we therefore decided to review data from our center to estimate the incidence of hyponatremia, to analyse the incidence and the impact outcame in patient admitted with TBI in our intensive care unit.

**Patients and methods**: It is a retrospective study conducted over a period of 4 years (from January 2009 to December 2012) in a polyvalent reanimation setting with 22 beds. All patients with postraumatic head injury who developed hyponatremia (serum sodium < 135 mmol/L) during the first 36 h of their stay were included. The hyponatremia was devided into mild (130 ≤ Na + < 135), moderate (125 ≤ Na + < 130) and severe hyponatremia (Na + < 125). Epidemiological, clinical and evolutionary data were collected and a descriptive and analytical statistical study was conducted.

**Results**: Two hundred and twenty one patients were included. All of them came from a road accident with an average age of 31.8 years. The male sex was predominant with a percentage of 84%. Eighty seven percent of patients presented mild hyponatremia whereas 12% had a moderate hyponatremia and only 1% developed a severe hyponatremia. The 25 patients with moderate hyponatremia experienced longer ventilator-free days (24 vs 16 +p=0.32), longer intensive care unit stays (22 vs 28 +p=0.15), and less favorable outcomes compared to the 193 patients who have mild hyponatremia + however, these differences were not significant.

**Conclusion**: Our study showed an elevated frequency of hyponatremia in patients with brain injuries in ICU which demands the effective approaches for an accurate and timely diagnosis of this electrolyte disorder. Further studies are needed to determine the optimal management strategy for TBI-associated hyponatremia in the intensive care unit setting.

### P-162 Terrorist threat- construction of a damage control training program for non-specialized caregivers

#### Pierre Pasquier (*speaker*)^1^, Astrée Swiech^1^, Thibault Martinez^1^, Gaël De Rocquigny^1^, Gwion Loarer^2^, Sylvain Vico^3^, Jérôme Planchon^4^, Arnaud Le Goff^5^, Kilian Bertho^6^, Clément Derkenne^6^,Stéphane Travers^7^, Brice Malgras^4^, Christophe Martinaud^8^, Cyril Carfantan^9^, Stéphane Gaudry^10^, Mathieu Boutonnet^1^

##### ^1^Percy Military Teaching Hospital, Clamart, FRANCE; ^2^French Military Medical Service, Vincennes, FRANCE; ^3^Sainte-Anne Military Teaching Hospital, Toulon, FRANCE; ^4^Bégin Military Teaching Hospital, Saint-Mandé, FRANCE; ^5^French Military Medical Service, Tours, FRANCE; ^6^Paris Fire Brigade, Paris, FRANCE; ^7^Ecole du Val-de-Grâce, Paris, FRANCE; ^8^French Military Blood Institute, Clamart, FRANCE; ^9^French Military Medical Service, Paris, FRANCE; ^10^Avicenne Hospital - APHP, Bobigny, FRANCE

###### **Correspondence:** Pierre Pasquier - pasquier9606@me.com

*Annals of Intensive Care* 2019, **9(Suppl 1)**:P-162

**Introduction**: The terrorist threat in France is real, with very different forms of attacks. So, it is challenging to train numerous non-specialized caregivers, with different backgrounds, in damage control (DC) strategies. The purpose of this work is to propose a specific training program, providing them a standard of care, with the very ambitious goal of zero avoidable deaths.

**Patients and methods**: A Task Force of 15 civilian and military physicians, experts in tactical medicine and medical training, met for a 24 h session, to propose the construction of a DC training program for non-specialized caregivers.

**Results**: DC training programs exist already but are heterogeneous, mainly theoretical and almost only for physicians. The Task Force has identified holes in existing training. A program named Damage Control for Terrorist Attack Victims (DC-TAV) was then proposed. Identified training targets were caregivers (physicians, nurses and nursing assistants), from prehospital and hospital staffs, with no trauma experience. The training objectives were the improvement of individual and collective skills for DC strategies for management of terrorist attacks casualties. The tools selected for training concerned e-learning on a dedicated digital teaching platform (including a core section of 4 modules with types and mechanisms of injury, basic DC techniques, triage, organization of emergency medical response + and 2 complementary modules for doctors with DC resuscitation and DC surgery), hands-on workshops with procedural simulation and full scale simulation exercises, technical (tourniquets, hemostatic gauzes, needle thoracostomy, chest tube drainage, management of airway, coniotomy) and non-technical (leadership, communication, coordination and triage, decision-making, appropriate use of resources) skills. A feasibility pilot study has been proposed in a rural area with no Trauma center, nor University Hospital. Finally, an evaluation of DC-TAV was planned, using the same tools as those used for training.

**Conclusion**: The DC-TAV program is an ambitious, civilian-military, national and long-term program. It is based on a harmonized standard of care and includes multidimensional training.

### P-163 Starting controlled donation after circulatory death (Maastricht III category) - Evaluation of potential donnor

#### Marie Jullien (*speaker*)

##### Dole, FRANCE

###### **Correspondence:** Marie Jullien - jullien.marie@gmail.com

*Annals of Intensive Care* 2019, **9(Suppl 1)**:P-163

**Introduction**: Despite an increase of organ donation, hundreds of patients die on waiting list every year. One of a solutions suggested by the Agence de la Biomédecine (ABM) was to develop organ donation from non-heart beating donor (NHBD) and particularly NHBD from category III of Maastricht classification (NHBD M3). This has been allowed since 2014 after some changes in French laws and a program launched by the ABM. We want to initiate organ donation from NHBD MIII in our hospital, CHU Besancon, in order to increase our graft pool. In 2015, 42 patients died after brain death and 28 patients were organ donor (DBD) in our hospital.

**Patients and methods**: We performed a retrospective analysis all of the deaths following a withdrawal of life sustaining support treatment (WLST) in intensive care unit (ICU) in 2015, using data from medical record. Primary endpoint was to identify how many patients could be NHBD M3. Eligibly criteria were- death in ICU after WLST, age between 18 and 65 years, major brain damage with no hope for recovery. Were excluded patients who present a contraindication of organ donation.

**Results**: In 2015, 68 patients died after WLST and 29 were 65 year old or less. We excluded 13 patients- 7 patients with cancer, 4 patients with multiple organ failures, 1 patient died before WLST, 1 patient was not brain injured.

16 patients could be NHBD M3 with a median age of 49[39–58], 80% were men. The cause of brain damage was for 11 patients a post anoxic encephalopathy, 3 subarachnoid hemorrhage, and for 2 patients brain trauma. The examination of medical record suggests that 15 patients could donate their kidneys and 11 patients could donate their liver (based on data few days before death). 50% patients died in the 3 h after WLST, and 1 patient has an uncontrolled infection which left 7 potential kidney donors and 5 potential liver donors.

**Conclusion**: In 2015, 16 patients were identified to be potentially a NHBD M3 in CHU Besancon in addition to 28 DBD. The data shows that patients were young and in good health which suggest a great quality of graft despite potential warm ischemic injuries due to the NHBD. Positive experience of the other hospitals since 2014 encourages us to initiate our program.


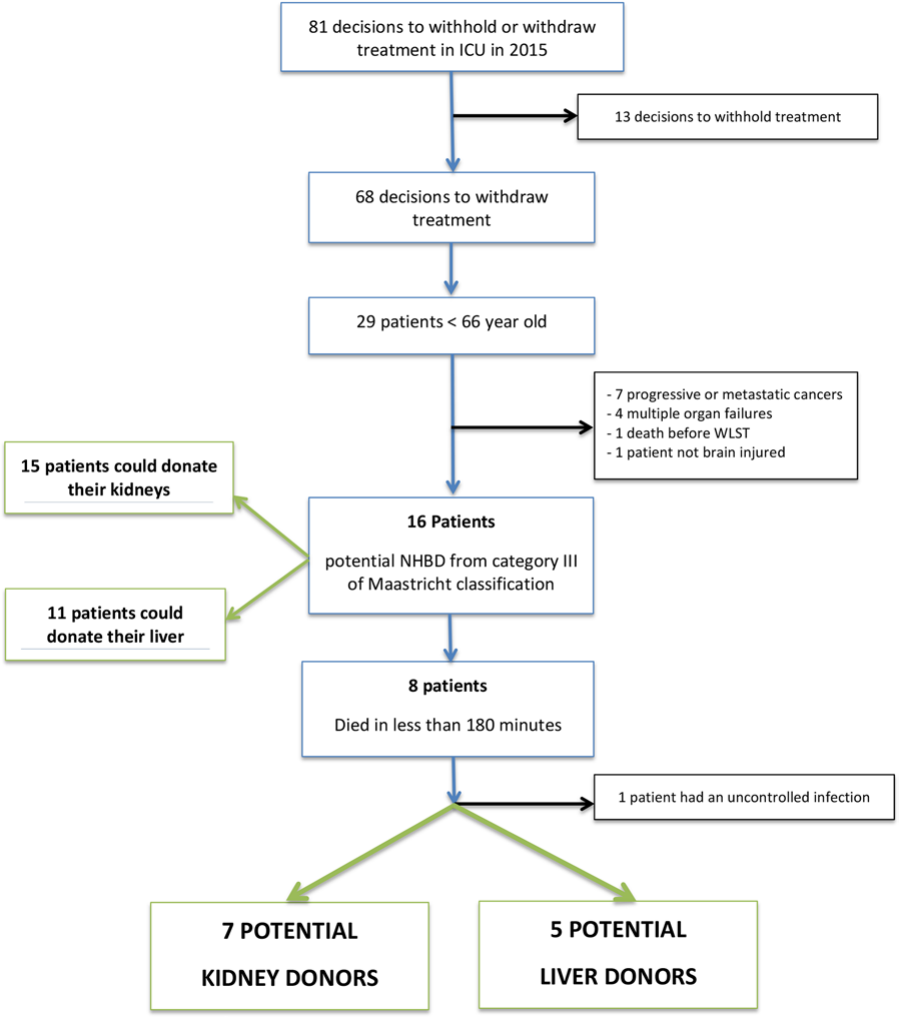


### P-164 Transcranial doppler coupling and trauma coma databank in moderate traumatic brain injury- what are the statistical and therapeutic impacts?

#### Mohamed Anass Fehdi (*speaker*), Amine Raja, Mohammed Mouhaoui

##### CHU Ibn Rochd, Casablanca, MOROCCO

###### **Correspondence:** Mohamed Anass Fehdi - mohamedanassf@gmail.com

*Annals of Intensive Care* 2019, **9(Suppl 1)**:P-164

**Introduction**: Transcranial Doppler (TCD) is a non-invasive examination that allows velocimetric study of intracranial vessels via an ultrasound beam. The authors propose to study the statistical and therapeutic impact of TCD and trauma coma databank (TCDB) data coupling in moderate cranial trauma in ED.

**Patients and methods**: This was a 12-month prospective study, including all patients > 15 years old, who had moderate head injury (GCS 9 to 13), initially admitted to the vital emergency room, and having immediately benefited from a cranio-cerebral CT and a DTC within 12 h post-admission, without respiratory and or hemodynamic distress and without gasometric anomalies (normal PaCO2 and absence of anemia). TDM allowed to distinguish 6 classes of trauma coma databank. The measurement of DTP was considered from a stable pattern over ten cardiac cycles. The parameters measured were- systolic velocimetry (SV), mean velocimetry (MV), and diastolic velocimetry (DV) as well as the pulsatility index (PI). The cerebral perfusion pressure was deduced by the equation- MAP x (DV MV) + 14. The data collection was concerned with demographic data, vital parameters and neurological deterioration at H48. The statistical analysis was univariate, (p < 0.05).

**Results**: 32 patients were included in this study, divided into two groups- group 1 (n = 22 69%) without neurological deterioration, and group 2 (n = 10 21%) with neurological deterioration. The mean age of the two groups was 36.56 ± 20.09 years, with a sex ratio of 16H 6 for group 1 and 7H 3F for group 2. The average initial GCS was 12 15 for the group 1 and 10 15 for group 2. 20 patients in group 1 had a TCDB of 1 to 2, while only 2 had a TCDB of 3 to 5. In group 2, 6 patients had a TCDB of 1 to 2, and 4 patients had a TCDB of 3 to 5. The PI of group 1 was 1.26 ± 0.60, while the PI of group 2 was 1.74 ± 0.26. The statistical performance of DTC, CTDB, and DTC CTDB coupling for the occurrence of neurological worsening is illustrated in the following table 1.

**Conclusion**: Coupling DTC TCDB data would increase the statistical predictive performance of early neurological deterioration, with high sensitivity, specificity and VPN. It would also guide the intensity of neuro-resuscitation. Results to be confirmed on a larger cohort.


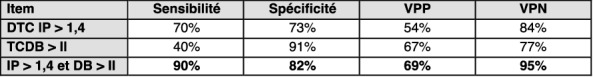


### P-165 Sepsis-induced inhibition of malignant tumor growth is associated with functional modulation of tumor-infiltrating natural killer cells

#### Clara Vigneron (*speaker*), Adrien Mirouse, Christophe Rousseau, Hamid Merdji, Clément Cousin, Fanny Alby-Laurent, Jean-Paul Mira, Jean-Daniel Chiche, Jean-François Llitjos, Frédéric Pène

##### Institut Cochin, INSERM U1016, CNRS UMR8104, Paris, FRANCE

###### **Correspondence:** Clara Vigneron - claravigneron@hotmail.fr

*Annals of Intensive Care* 2019, **9(Suppl 1)**:P-165

**Introduction**: The immune system plays a central role in surveillance against neoplasms development. We developed a research project investigating the impact of sepsis-induced immune dysfunctions on malignant tumor growth. In sepsis-then-cancer model, it is likely that sepsis-induced immune suppression promotes tumor growth. In cancer-then-sepsis model, sepsis may rather inhibit tumor growth. We herein investigated how sepsis and Toll-like receptor (Tlr) signaling may promote the antitumoral functions of cytotoxic natural killer (NK) cells and TCD8 lymphocytes (TCD8).

**Patients and methods**: We used C57BL 6 J wild-type (WT), Tlr4- -, Tlr2- - and Myd88- - mice. Mice were first subcutaneously inoculated with MCA205 fibrosarcoma cells. Fourteen days later, mice were subjected to a septic challenge through polymicrobial sepsis induced by cecal ligation and puncture (CLP) or endotoxinic shock induced by intraperitoneal lipopolysaccharides (LPS) injection. The main functions of tumor-associated cytotoxic NK cells and TCD8 were assessed by flow cytometry through intracellular expression of interferon-gamma (IFN-&#947 +) and of cytotoxic molecules perforin and granzyme, as well as outer membrane expression of the degranulation marker CD107a. In order to address their cytotoxic functions in vitro, NK cells were isolated from septic or control mice and exposed to MHC1low YAC-1 or MCA205 tumor cells.

**Results**: Polymicrobial sepsis dampened tumor growth in WT and Tlr2- - mice, but neither in Tlr4- - nor in Myd88- - counterparts. A similar tumor growth inhibition was observed following a LPS challenge in WT mice. The distribution of tumor-infiltrating NK and TCD8 cells was weakly affected by sepsis. TCD8 did not undergo any significant functional changes. Among NK cells, LPS induced an expansion of the cytotoxic CD11b + CD27 + and CD11b + CD27- subsets along with increased expression of the activation marker NKG2D. Tumor-infiltrating NK cells obtained from septic mice exhibited enhanced antitumoral properties. NK cells obtained from CLP-operated mice displayed increased expression of IFN-&#947 + . The behaviour of NK cells obtained from LPS-challenged mice was consistent with an active degranulation process, as suggested by increased CD107a membrane expression along with decreased intracellular expression of perforin and granzyme. MHC1 expression was low in MCA205 cells, similar to that in YAC-1 cells. The antitumoral properties of NK cells obtained from septic mice were exacerbated when cultured with MHC1low YAC-1 or MCA205 cells.

**Conclusion**: Sepsis promotes the main antitumoral functions of NK cells in cancer mice. Our results point to a critical regulatory role of Tlr4 in this setting.

### P-166 Early administration of fibrinogen concentrates to improve postpartum hemorrhage

#### Anouar Jarraya (*speaker*), Manel Kammoun, Wassim Ferjani, Aslam Abid, Grati Faiza, Kamel Kolsi

##### Hedi chaker university hospital, Sfax, TUNISIA

###### **Correspondence:** Anouar Jarraya - dranouarjarraya1983@gmail.com

*Annals of Intensive Care* 2019, **9(Suppl 1)**:P-166

**Introduction**: To date, maintaining fibrinogen levels above 2 g L is a recommended therapeutic target in postpartum hemorrhage. However, the timing of fibrinogen supplementation is still controversial. The purpose of this retrospective study was to describe our experience in early administration of fibrinogen concentrate in severe PPH.

**Patients and methods**: We analyzed a database of 33 patients who needed fibrinogen concentrate transfusion for the treatment of severe postpartum hemorrhage after cesarean delivery to treat coagulopathy (plasmatic fibrinogen < 2 g/L), or after massive transfusion, or earlier (before the result of plasmatic concentration of fibrinogen). Patients were divided into two groups. • Group E (early) - received fibrinogen concentrates within the first hour after delivery. • Group L (late)- received fibrinogen transfusion later than 1 h. Then, we assessed the blood loss and the transfusion requirements.

**Results**: Demographic parameters (age, weight, patient’s height, gestity and parity) and pre-operative hemostatic status were comparable in both groups. Blood loss was correlated to the delay of fibrinogen administration (Fig. 1). The Pearson correlation coefficient was 0.688. The mean blood loss was 2486 ml in group E (n = 12) versus 5310 ml in group L (n = 21) with p = 0.002. Red blood cell transfusion requirement was 4.58 units patient in group E versus 8.14 in group L (p = 0.01). The need of fresh frozen plasma was 7 units patient in group E versus 12.3 in group L (p = 0.045).

**Discussion**: FIDEL study [1], a prospective multi centric French study, whose results are not yet published, may give the answer for the best timing of fibrinogen concentrate infusion in severe PPH.

**Conclusion**: Early administration of fibrinogen within the first hour following delivery may reduce bleeding in severe postpartum hemorrhage and transfusions requirement.

### P-167 Which optimal daily dosing of Enoxaparinin acute burn patients?

#### Amel Mokline (*speaker*)^1^, Hana Benali^1^, Kawther Elfeleh^1^, Hana Fraj^1^, Imen Rahmeni^1^, Beya Maamer^1^, Achref Laajili^1^, Manel Ben Saad^1^, Lamia Thabet^2^, Amen Allah Messadi^1^

##### ^1^Intensive Burn Care Department, Burn and Trauma Center, Tunis, TUNISIA; ^2^Laboratory of clinical biology, Burn and Trauma Center, Tunis, TUNISIA

###### **Correspondence:** Amel Mokline - dr.amelmokline@gmail.com

*Annals of Intensive Care* 2019, **9(Suppl 1)**:P-167

**Introduction**: Venous thromboembolic events (VTE) is an often silent complication in burn patients, which is strongly associated with poor outcomes. Previous research [1] has shown that burn injury size (TBSA) and weight affected Enoxaparin dosing in burns. The aim of our study was to assess this hypothetis in burns treated with Enoxaparin for VTE prophylaxis to achieve adequate anti factor Xa levels (anti-Xa) with initial dosing and its impact to reduce VTE.

**Patients and methods**: This study was conducted in burn center in Tunis from February 2018 To September 2018. Acute burn patients admitted to the burn center and anticipated to be nonambulatory for greater than 48 h were included. Were excluded patients with any contraindication to the use of enoxaparin, including intracranial bleeding or hemorrhagic stroke (within 48 h), neurotrauma, suspected or proven bleeding, and those with creatinine clearance < 30 ml mn or creat > 1.6 mg/dL. Enrolled patients received Enoxaparin as following- Enoxaparin dose in mg Q12Hrs = 22.8 + (3.3 x  % TBSA 10) + (1.89 x (weight in kg) 10) [1]. Peak anti-Xa was obtained between 3 and 5 h after the third enoxaparin dose. Doses of enoxaparin were titrated up or down by 20% to achieve the recommended anti-Xa of 0.2 to 0.4U ml.

**Results**: 30 burned patients were included. The mean age was 35 ± 17 years with a ratio sex of 3.29. The average TBSA was 41.5 ± 17% with a body weight of 68.5 ± 17 kg. Fourteen patients (46%) reached anti-Xa target initially, 15 patients’ anti-Xa was below target and only 1 patient’ anti-Xa was above target.The median final enoxaparin dose was 40 mg Q12Hrs (range, 30–80 mg) for all patients who were at anti-Xa target. Comparative study of 2 groups of patients- anti-Xa target initially (G1) and below target (G2) was as follows (Table 1)- No episodes of hemorrhage, thrombocytopenia, or heparin-associated allergy documented in any of the study patients.VTE occured in 5 patients among which 4 cases in patients’ anti-Xa below target initially.

**Conclusion**: Enoxaparin dosing equation in burns allows to reach a prophylactic initial anti-Xa level, and was associated with a low incidence of VTE events and no bleeding complications. Enoxaparin dosing correlates strongly with burn size, patient weight and clearance of creatinine. Thus, a standard dose for all adult acute burn patients is recommended.

**Reference**

1. Faraklas I, and al. J Trauma 2011;71:1557–61.

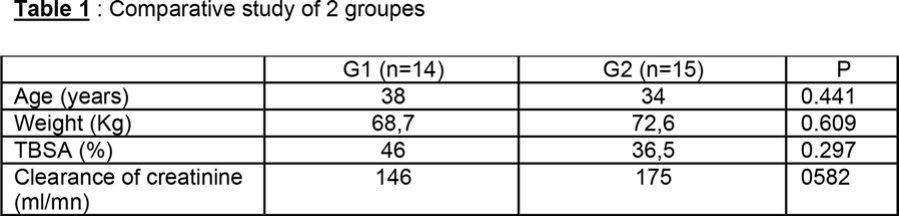



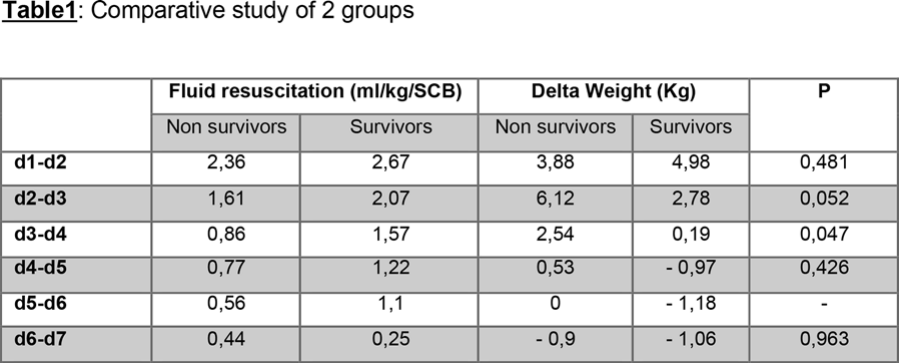


### P-168 Performance of diagnostic scores in patients with severe thrombotic microangiopathies

#### Eric Mariotte (*speaker*)^1^, Lara Zafrani^1^, Lionel Galicier^2^, Etienne Ghrenassia^1^, Lionel Kerhuel^1^, Laure Calvet^1^, Audrey De Jong^1^, Virginie Lemiale^1^, Sandrine Valade^1^, Elie Azoulay^1^,Michael Darmon^1^

##### ^1^Médecine Intensive Réanimation - Hôpital Saint Louis - APHP, Paris, FRANCE; ^2^Immunopathologie Clinique - Hôpital Saint Louis - APHP, Paris, FRANCE

###### **Correspondence:** Eric Mariotte - eric.mariotte@aphp.fr

*Annals of Intensive Care* 2019, **9(Suppl 1)**:P-168

**Introduction**: Thrombotic thrombocytopenic purpura (TTP), an element of the thrombotic microangiopathy (TMA) syndrome, is caused by a severely deficient ADAMTS13 activity leading to the spontaneous formation of thrombi in the microcirculation. Early TTP recognition is critical as the disease is almost always lethal if not treated promptly with plasma exchanges. As ADAMTS13 dosage is unavailable in routine, scores have been developed to help differentiating TTP from other TMAs. The aim of this work was to study the accuracy of these diagnostic scores in ICU setting.

**Patients and methods**: The performance of Coppo and PLASMIC scores was studied in a cohort of TMA patients admitted to our university hospital ICU from 2006 to 2017. Results are presented as median (interquartile range) and numbers (%). TTP and non-TTP groups were compared using non-parametric tests. ROC curves were established for each score, confidence intervals of the AUC were determined using DeLong’s bootstrap method. A multivariate logistic regression analysis was then performed.

**Results**: During the study period 154 patients with TMA required ICU admission, including 99 (64.2%) TTP and 55 (35.7%) non-TTP patients. Hematologic signs were more pronounced in the TTP group with hemoglobin rates of 7.6 g/dL (6.1–9.0) vs 8.6 g/dL (7.1–10.0, p < 0.01) + and platelet rates of 11G L (8–20) vs 45G L (23.5–70.5, p < 0.01). In TTP patients, renal involvement was less severe (creatinine 96 µmol L [75–146] vs 267 µmol L [141–511], p < 0.01) and neurological involvement was more prevalent (83.9% vs 58.2%, p < 0.01) than in non-TTP patients. Mortality rates were 6.1% for TTP patients versus 16.4% for non-TTP patients (p = 0.07). AUC under ROC curve in predicting TTP was 0.863 (CI95% 0.8061–0.9192) for the Coppo score, 0.6697 (CI95% 0.582–0.7566) for the PLASMIC Score, and 0.863 [CI95% 0.806–0.9202] for thrombocytopenia alone (figure 1). These results were confirmed after adjustment for confounders using multivariate logistic regression and comparing both calibration and variance explained by the models.

**Conclusion**: In a cohort of severe TMA patients requiring ICU admission, the PLASMIC score had limited performance for the diagnosis of TTP. The performance of the Coppo score was good but similar to a single highly discriminant item- platelet count.


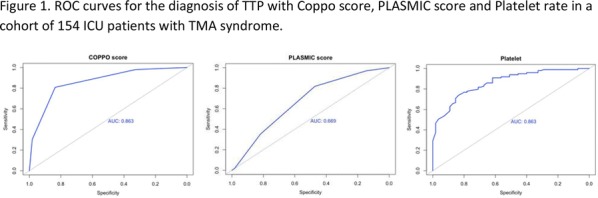


### P-169 The new definition of sepsis and septic shock is usable in cancer patients

#### Nathan Neveux (*speaker*), Marianne Paesmans, Lieveke Ameye, Jean-Paul Sculier, Anne-Pascale Meert

##### Institut Jules Bordet, Bruxelles, BELGIUM

###### **Correspondence:** Nathan Neveux - Nathan.Neveux@ulb.ac.be

*Annals of Intensive Care* 2019, **9(Suppl 1)**:P-169

**Introduction**: In 2016, a new definition of sepsis and septic shock, based on the SOFA score and the qSOFA score, has emerged. Some studies conducted in general populations demonstrated that SOFA score is more accurate than SIRS score to predict hospital mortality of infected patients requiring intensive care. However, nowadays, in an oncological population, only one study has shown that this new definition can predict hospital mortality with the same reliability as in the general population.

**Patients and methods**: We have analyzed all the records of cancer patients admitted for an infection from 01 01 2013 to 31 12 2016 in our oncological ICU. For all them, we have calculated the SOFA, qSOFA and SIRS scores. We have tried to determine the most accurate score to predict hospital mortality. We also have compared the new and the old definitions of septic shock. Finally, we have analyzed the prognostic factors for hospital mortality.

**Results**: Our study includes 353 patients- 241 with a solid tumor (of whom 177 have metastasis) and 112 with a hematological malignancy (of whom 30 allografts). The hospital mortality rate is 37% (68% in patients with septic shock according to the new definition and 60% according to the old definition). 92% of patients have a SOFA score increased by more than 2 points since the admission in the ICU, 63% have a qSOFA > or = 2 and 99% a SIRS score > or = 2. The SOFA score reaches the best diagnostic performance to predict hospital mortality + Area under the ROC curve (AUC) of 0.74 compared to AUC 0.65 for qSOFA and 0.58 for the SIRS score. In multivariate analysis, a higher SOFA score or a higher qSOFA score predicts poor prognosis- odds ratio (OR) per one-point increase 1.28 (95% CI, 1.18 to 1.39) and 1.48 (95% CI, 1.04 to 2.11), respectively, while complete remission of cancer is a good prognostic factor for hospital mortality- OR 0.39 (95% CI, 0.22 to 0.67).

**Conclusion**: The new definition of sepsis and septic shock is usable in an ICU oncological population with a same reliability similar to that reported in general populations. In oncological patients admit to the ICU for a suspected infection, the SOFA score is more accurate than qSOFA and SIRS scores to predict hospital mortality.

### P-170 Major abdominal or pelvic surgery in cancer patients ≥ 80 years- long-term prognosis and associated risk factors

#### Fanny Depeyre (*speaker*), Marion Faucher, Jean Manuel De Guibert, Smail Hamouda, Sylvie Cambon, Lam Nguyen Dong, Florian Cardot, Clément Brun, Laurent Chow-Chine, Magali Bisbal,Bernard Lelong, Olivier Turrini, Antoine Sannini, Djamel Mokart

##### Institut Paoli-Calmettes, Marseille, FRANCE

###### **Correspondence:** Fanny Depeyre - depeyrefanny@gmail.com

*Annals of Intensive Care* 2019, **9(Suppl 1)**:P-170

**Introduction**: Due to the evolution of the demography, surgical management of elderly oncology patients evolves steadily. In this population preoperative benefit-risk balance remains difficult to establish. The main objective of our study was to evaluate long-term mortality and associated risk factors in cancer patients ≥ 80 years undergoing major abdominal and or pelvic surgery and systematically admitted to intermediate care unit (IMC) or intensive care unit (ICU).

**Patients and methods**: We performed an observational, retrospective and monocentre cohort study in the IMC and ICU of the Paoli Calmettes Institute in Marseille from March 2009 to April 2016. All cancer patients aged ≥ 80 and undergoing abdominal or pelvic surgery were included. Univariate and multivariate Cox analysis of 1-year mortality were performed.

**Results**: 178 patients met the inclusion criteria and were analyzed. The median age was 83 [81–86] years. Colorectal surgery accounted for 43.8% (n = 78) interventions, splenic and pancreatic surgery 13.4% (n = 24), hepatic surgery 9.5% (n = 17), urological surgery 7.3% (n = 13), gynecological surgery 3.3% (n = 6) and gastric surgery 3.3% (n = 6). Median duration of follow-up was 26.6 months 95% CI [19.3–33.9]. Mortality observed during this period was 29.8% (n = 53), 95% CI [23 to 37.1]. In univariate analysis, risk factors associated with mortality were- a score ASA > 2 (p = 0.004), pre-operative malnutrition (p < 0.001), pre-operative cognitive disorders (p = 0.001), emergency surgery (p = 0.019) and intraoperative vasopressor (p = 0.031). Curative surgery was associated with better long-term survival (P < 0.001). The type of the cancer, the type of surgery and a metastatic disease did not influence long-term mortality. Using multivariate analysis, five factors were associated with prognosis- history of diabetes (p = 0.008), cognitive disorders (p = 0.008), pre-operative malnutrition (p = 0.007), and intraoperative vasopressor (p = 0.006). The curative nature of the surgery was associated with better survival (p < 0.001).

**Conclusion**: With 70% long-term survival, major oncological surgery may be considered in patients ≥ 80 years. Even though the curative nature of surgery remains a determining factor, our work strongly highlights the importance of comorbidities in this context. In view of our data, preoperative optimization, within the framework of specific pre-habilitation protocols must be evaluated prospectively. Preoperative optimization, within the framework of specific pre-habilitation protocols, must be prospectively evaluated.


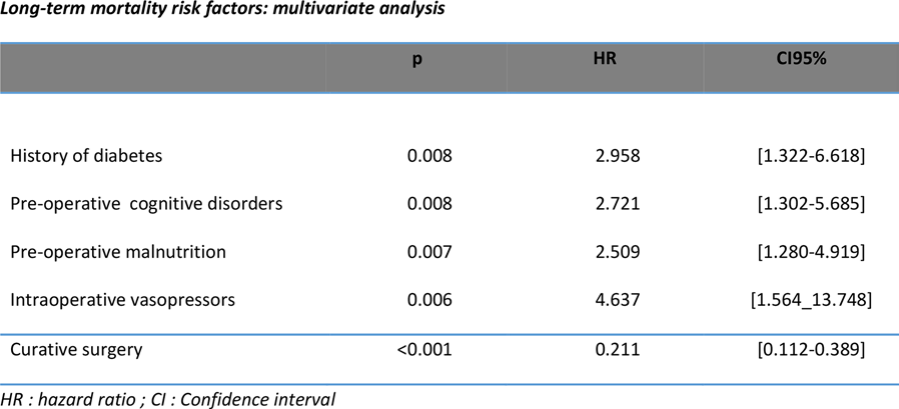


### P-171 Sepsis and septic shock of biliary origin in onco-haematology patients (OHP) admitted to intensive care unit (ICU)- Mortality and associated factors

#### Laurent Chow-Chine (*speaker*), Antoine Sannini, Fabrice Caillol, Magali Bisbal, Luca Servan, Frédéric Gonzalez, Jean Manuel De Guibert, Marion Faucher, Djamel Mokart

##### Institut paoli-Calmettes, Marseille, FRANCE

###### **Correspondence:** Laurent Chow-Chine - chowchinel@ipc.unicancer.fr

*Annals of Intensive Care* 2019, **9(Suppl 1)**:P-171

**Introduction**: Incidence of cancers is constantly increasing. The survival of critically ill cancer patients has improved over the past decades. Data are scarce regarding biliary sepsis septic shock in OHP (BSSOHP). The main objective of this study was to evaluate hospital and 1-year mortality as well as the associated factors in a context of BSSOHP.

**Patients and methods**: Observational retrospective monocentric study including 122 patients between November 2008 and October 2017. We described two groups of patients- a group consisting of 84 OHP and a control group of 38 non-cancer patients. All presented with BSSOHP at ICU admission. Perioperative parameters were collected as well as performans status (PS), Karnofsky score and the stage of the malignancy at hospital discharge and 1 year. Statistic- univariate and multivariate analysis using logistic regression. p < 0.05 was considered as significant.

**Results**: For OHP, hospital mortality was 24%(n = 20) and 1-year mortality 49%(n = 37). In the control group, it was 5.3%(n = 2), and 5.4%(n = 2) respectively. For OHP and using multivariate analysis, factors independently associated with hospital mortality were- SAPSII (OR 1.134, CI 95% 1.055–1.218), renal replacement treatment (OR = 8.6 95% CI 1.44–51.32), biliary ESBL before endoscopy (OR 7.63 95% CI 1.26–46.11) and the presence of duodenal stenosis (OR 13.7 95% CI 2.08–90.66). Factors associated with 1-year mortality were- the presence of metastasis (OR 6.5 95% CI 1.17–36.77), PS > 2(OR 14.56 95% CI 1.088–168.93), SAPSII (OR 1.079 95% CI 1.023–1.139), biliary procedures > 2(p = 0.008 OR 3.65 95% CI 1.394–9.559). Surgery before biliary drainage (OR 0.062 95% CI 0.013–0.0292) was associated with a good prognosis. At 1 year, 64.8%(n = 24) of the survivors continued to receive chemotherapy and 13.5%(n = 10) radiotherapy, 43.6% (n = 17) were in remission of their disease. Physical status was quiet conserved with a Karnofsky score of 80 (IQR 70–90) and a PS > 2 in only 7.6% (n = 3) of 1 year survivors. Finally, 12.8% (n = 5) of them had palliative care.

**Conclusion**: Hospital mortality was low. Organ failures, ESBL documentation and difficulty of biliary drainage are major factors for short-term outcome. Malignancy seems to only affect long-term mortality.

### P-172 NIV indication for critically ill cancer patients admitted to the ICU for mixed lesional and cardiac acute respiratory failure? Results from an observational study

#### Colombe Saillard (*speaker*), Damien Mallet, Laurent Chow-Chine, Magali Bisbal, LucaServan, Frédéric Gonzalez, Jean Manuel De Guibert, Marion Faucher, Antoine Sannini, Djamel Mokart

##### Insitut Paoli-Calmettes, Marseille, FRANCE

###### **Correspondence:** Colombe Saillard - saillardc@ipc.unicancer.fr

*Annals of Intensive Care* 2019, **9(Suppl 1)**:P-172

**Introduction**: Acute respiratory failure (ARF) is a severe life-threatening complication in onco-hematology patients. ARF is frequently mixed, associating lesional and cardiogenic edema. Survival benefits from NIV could be harder to demonstrate or may have been balanced by changes in ventilation strategy selection. Strategies can differ according to ARF etiology and severity. NIV has been validated in acute cardiac pulmonary edema and acute exacerbations of chronic obstructive pulmonary disease with decreased intubation and mortality rates. at the opposite, high-flow oxygen through a nasal cannula (HFNC) has been validated in a context of de novo ARF. Data of NIV in cancer patients with mixed ARF are lacking. The aim of this study was to assess prognostic factors, including ventilation strategies, associated with intensive care unit (ICU) mortality.

**Patients and methods**: We conducted an observational retrospective study in Institut Paoli-Calmettes, a cancer-referral center. Between 2008 and 2015, 124 critically ill cancer patients with mixed ARF were analyzed. Factors associated with ICU mortality, using univariate, multivariate and matched propensity score analysis, were evaluated.

**Results**: ICU and hospital mortality were 29% and 57%. Initial ventilation strategy at ICU admission consisted of mechanical ventilation (MV) in 21%. Others patients received noninvasive ventilation (NIV) in 50%, associated with oxygen in 21% and high flow nasal oxygen (HFNO) in 29%, HFNO alone in 6% and standard oxygen in 23%. During ICU stay, 48% of patients required intubation. Multivariate analysis identified 3 independent factors associated with ICU mortality- SAPSII at admission (OR = 1.07 point, 95%CI = 1.03–1.11, p < 0.001), invasive fungal infection (OR = 7.65, 95%CI = 1.7–34.6, p = 0.008) and initial ventilation strategy (p = 0.015). Compared to NIV, HFNO alone and standard oxygen alone were associated with an increased ICU mortality, with respective OR of 19.56 (p = 0.01) and 10.72 (p = 0.01). We subsequently realized a propensity score analysis including 40 matched patients, 20 in the NIV arm and 20 receiving other ventilation strategies, excluding initial MV patients. ICU mortality rate was significantly lower in patients treated with NIV (10%), versus 50% in patients receiving other ventilation strategies (p = 0.037).

**Conclusion**: For onco-hematology patients presenting with mixed ARF, severity at ICU admission, invasive fungal infections and initial ventilation strategy were independently associated with ICU mortality. NIV was a protective factor on ICU mortality. NIV should be evaluated for mixed ARF in cancer patients.

### P-173 Prognostic factors of acute pancreatitis in intensive care. Retrospective study over 7 years

#### Benoît Courteille (*speaker*)^1^, Xavier Valette^1^, Suzanne Goursaud^1^, Jean-Luc Hanouz^2^, DamienDu Cheyron^1^

##### ^1^Réanimation médicale - CHU, Caen, FRANCE; ^2^Département d’Anesthésie-Réanimation CHU, Caen, FRANCE

###### **Correspondence:** Benoît Courteille - benourt@gmail.com

*Annals of Intensive Care* 2019, **9(Suppl 1)**:P-173

**Introduction**: To define prognostic factors of mortality in critically ill patients admitted to intensive care unit for acute pancreatitis.

**Patients and methods**: Retrospective, single-center study in two intensive care units in the Caen University Hospital between January 1, 2010 and December 31, 2016. All patients admitted for acute pancreatitis were included. Patients with isolated high level of lipasemia, extra-pancreatic disease responsible for acute illness, cardiopulmonary arrest or polytrauma were secondarily excluded. Demographic, clinical, and biological data, as well as different characteristics of pancreatitis defined according to the revised Atlanta classification of 2012, and pancreatitis induced-complications during ICU stay were collected.

**Results**: One hundred and two patients were included, among them 58 and 44 in medical intensive care and surgical intensive care units, respectively. The overall mortality was 29%. All the deceased patients had pancreatitis classified as severe. SOFA score at admission was significantly lower for survivors than for non-survivors, respectively 8 (IQ - 5–11) and 12 (9–16) p < 0.0001. IGS II score was also significantly lower among survivors than among non-survivors. The mortality rates were similar in patients with or without necrosis infection. In univariate analysis, sofa score at admission, multiple organ failure, renal dysfunction, metabolic acidosis, hypoxemia and haemoconcentration were associated with death, as well as the need for renal replacement therapy and mesenteric ischemia during ICU stay. In different models of multivariate analysis, the SOFA score at ICU admission (OR 1.24 [95%CI, 1.01–1.45], p = 0.006), the need for renal replacement therapy (OR 5.9 [1.8–19.2], p < 0.03), bacteremia (OR 6.8 [1.8–26.8], p = 0.005) and mesenteric ischemia (OR 9.0 [1.5–53.1], p = 0.01) during ICU stay were identified as independent risk factors of mortality. Conversely, enteral nutrition was independently associated with survival (OR 0.05 [0.01–0.2], p = 0.0001).

**Conclusion**: The SOFA score at admission, the need for renal replacement therapy, and complications such as bacteremia and mesenteric ischemia during ICU stay were identified as independent predictors of mortality in patients admitted to intensive care units for acute pancreatitis. Enteral nutrition was an independent factor of survival.


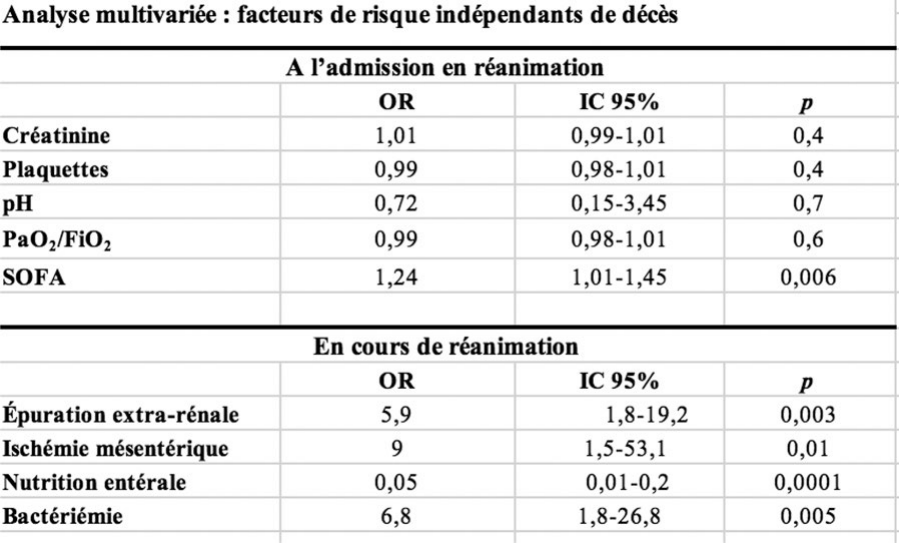


### P-174 Infected pancreatic necrosis in the course of severe acute pancreatitis- can we predict catheter drainage failure and need for additional necrosectomy?

#### Charlotte Garret (*speaker*)^1^, Marion Douillard^1^, Morgane Pere^1^, Eric Frampas^1^, Ludivine Legros^2^, Rubeshen-Pillay Arnachellum^3^, Jean Reignier^1^, Nicolas Regenet^1^, Mathieu Peron^1^, Jérôme Gournay^1^,Marc Lerhun^1^, Isabelle Archambeaud^1^, Arthur David^1^, Emmanuel Coron^1^

##### ^1^CHU, Nantes, FRANCE; ^2^CHU, Rennes, FRANCE; ^3^CHU, Brest, FRANCE

###### **Correspondence:** Charlotte Garret - charlotte.garret@chu-nantes.fr

*Annals of Intensive Care* 2019, **9(Suppl 1)**:P-174

**Introduction**: Recent guidelines advocate the step-up approach for the management of infected pancreatic necrosis (IPN). Nearly half of patients will require secondary necrosectomy after drainage. A previous study (Hollemans et al.) identified 4 risk factors of catheter drainage failure- male gender, multiple organ failure, percentage of necrosis and heterogeneous collection and they constructed a nomogram with an area under receiver operating characteristic (ROC) the curve of 0.76. Our primary objective was to validate de constructed nomogram. Our secondary goal was to explore others possible predictors of catheter drainage failure.

**Patients and methods**: We retrospectively studied 72 consecutive patients admitted for suspected IPN requiring interventions by catheter drainage first between 2012 and 2016 admitted in 3 university hospitals. All computed tomography (CT) prior to the first drainage procedure were reviewed by a single blinded radiologist.

**Results**: Catheter drainage procedure was a success for 44.4% of patients. Nomogram predicted catheter drainage failure with an area under the ROC curve of 0.71. A score ≤ 8 results in a 100% success chance of primary catheter drainage. Similarly, unfavorable score of 40 points results in a 100% failure of primary catheter drainage. In multivariate analysis, catheter drainage failure was independently associated with body mass index (BMI) (OR- 1.14 + 95% CI- 1.01–1.29 + P- 0.02), heterogeneous collection (OR- 18.84 + 95% CI- 2.02–175.86 + P- 0.01) and respiratory failure 24 h prior catheter drainage (OR- 16.76 + 95% CI- 1.94–144.4 + P- 0.01).

**Conclusion**: Half of the patients required necrosectomy after primary catheter drainage. Nomogram can be useful to predict catheter drainage failure, especially in the extreme ranges. Heterogeneous collection on CT and respiratory failure 24 h prior to drainage were strongly associated with catheter drainage failure. Early identification of patients at risk of drainage failure alone could improve their management and reduce antibiotics duration.

### P-175 Redefining the role of antibiotics in infected pancreatic necrosis during severe acute pancreatitis- an observational study of 137 microbiological samples

#### Charlotte Garret (*speaker*), Stephane Corvec, Jean-Baptiste Lascarrou, Emmanuel Coron, Aurélie Le Thuaut, Nicolas Regenet, Mathieu Peron, Isabelle Archambeaud, Frederic Douane, Marc Lerhun,Cedric Bretonniere, David Boutoille, Jean Reignier

##### CHU, Nantes, FRANCE

###### **Correspondence:** Charlotte Garret - charlotte.garret@chu-nantes.fr

*Annals of Intensive Care* 2019, **9(Suppl 1)**:P-175

**Introduction**: Recent guidelines ruled out prophylactic antibiotics to prevent infected pancreatic necrosis (IPN) and advocate the step-up approach for the management of IPN. Emerging drug resistant bacteria is a rising problem among SAP and ICU patients. Our primary objective was to determine the impact of antibiotherapy on samples cultures collected during interventions for an IPN among patients admitted for severe acute pancreatitis (SAP). Description of microbiological species and their patterns of resistance were our secondary goals.

**Patients and methods**: We retrospectively studied 62 consecutive patients admitted in ICU for suspected IPN requiring interventions. We collected data about microbiological samples, as well as antimicrobial therapy. We classified species according to the definitions proposed by Magiorakos et al.

**Results**: IPN was confirmed for 48 patients, with 137 samples collected during interventions. 57% of positive cultures were levied under efficient antibiotherapy > 24 h, previous antibiotherapy did not influence cultures results (p = 0.84). 13 of samples were polymicrobial. Gram staining had a sensitivity of 64.5% and a specificity of 96.7%, a Positive Predictive Value (PPV) of 98.6% and a Negative Predictive Value (PNV) of 43.2%. Half of the patients developed multi and extensively drug resistant bacteria. Prolonged antibiotics did not sterilized collections or necrotic tissues.

**Discussion**: Previous antibiotherapy did not influence microbiological culture results and prolonged adapted broad-spectrum antibiotics did not allow sterilizing necrotic tissue or fluid collections. Our results showed a 50% prevalence of drug resistant bacteria. Gram staining can not be used to invalidate IPN diagnosis. Negative cultures among patients under antibiotics should question the diagnosis of IPN and antibiotics discontinuation.

**Conclusion**: Prolonged broad Spectrum antibiotics did not permit to sterilize IPN, but prevalence of drug resistance bacteria is raising, up to 50% in our study. Strategies to reduce antimicrobial therapy use and exposure in the course of SAP are needed. Futures prospective studies are needed to determine the optimal antimicrobial therapy duration.

### P-176 Severe acute liver injury - Etiologies and outcome

#### Khaoula Ben Ismail (*speaker*)^1^, Sana Khedher^2^, Ameni Khaled^2^, Mohamed Salem^2^

##### ^1^Hôpital Ben Arous Yassminet, Hammam Plage | Ben Arous, TUNISIA; ^2^Hôpital Charles Nicolle, Tunis, TUNISIA

###### **Correspondence:** Khaoula Ben Ismail - khaoula87@hotmail.fr

*Annals of Intensive Care* 2019, **9(Suppl 1)**:P-176

**Introduction**: Severe acute liver injury is characterized by an acute liver damage without underlying chronic liver disease. It is a critical condition as it may lead to acute liver failure. The aim of this work was to provide epidemiological and prognostic features of this condition.

**Patients and methods**: It was a retrospective 1-year study from January to December 2017. We included all patients admitted for severe acute liver injury (ALI).

**Results**: Forty-six patients were included with a sex-ratio at 0.5 and a mean age of 48 ± 19 years (16–87). The main comorbidities were diabetes mellitus (31%) and hypertension (28%). Principal etiologies of ALI were hepatitis (46%) and drugs (28%). Hepatitis type B was incriminated in 11 patients (24%), type A in 8 patients and type E in 2 patients. Acute liver failure (ALF) occurred in 7 patients. ALF was classified as hyperacute in 2 patients, acute in 3 patients and subacute in 2 patients. Six patients died (13%) because of ALF. Patients with unfavorable outcome (death or ALF), in whom demographic characteristics were similar to patients with favorable outcome, had lower platelet count (198.103 ml vs 110 .103 ml, p = 0.025), lower prothrombin time ratio (22 ± 11% vs 45 ± 22%, p < 0.001), higher INR level (1.52 ± 2.1 vs 3.6 ± 0.44 p < 0.001), lower hemoglobin amount (9.5 ± 3.4 g/dL vs 12 ± 2.2 g/dL, p = 0.029), lower diastolic blood pressure (58 ± 8 mmHg vs 72 ± 13 mmHg, p = 0.015) and lower mean blood pressure (76 ± 9 mmHg vs 89 ± 13 mmHg, p = 0.026). The results of the logistic regression analysis showed that only diastolic blood pressure level was significantly related to unfavorable outcome. ROC curve analysis showed that a diastolic blood pressure level < 60 mmHg was a predictive factor of unfavorable outcome with a sensibility of 75% and specificity of 95% (AUR = 0.802, 95% IC 0.627–0.977).

**Conclusion**: ALI is associated with a non-negligible mortality mainly due to ALF. A diastolic blood pressure level less than 60 mmHg may help to predict unfavorable outcome.

### P-177 Acquired hepatic dysfonction in critically ill patients

#### Ghada Sbouii (*speaker*)^1^, Ines Fathallah^2^, Khaoula Ben Ismail^2^, Sahar Habacha^2^, Haifa Fazzeni^2^, Eya Sghir^2^, Amani Sghaier^2^, Emna Ennouri^2^, Asma Mehdi^2^, Nadia Kouraichi^2^

##### ^1^Yasminet hospital, Kairouan, ABKHAZIA; ^2^Ben arouss regional hospital, medical ICU, Ben Arouss, TUNISIA

###### **Correspondence:** Ghada Sbouii - ghadasrlf@hotmail.com

*Annals of Intensive Care* 2019, **9(Suppl 1)**:P-177

**Introduction**: Hepatic dysfunction is common in intensive care; however, few data are available. Hepatic dysfonction in critically ill patients remains a significant problem. The purpose of our study was to evaluate incidence, time of onset, characteristics and possible etiologies of liver function abnormalities in an intensive care unit (ICU).

**Patients and methods**: A retrospective cohort study conducted in patients admitted in an ICU, from the 1th January 2018 to 15th September 2018. We included all patients who presented during their stay elevation of hepatic transaminases (ASAT ALAT) and or elevation of gamma-glutamyl transpeptidase (GGT) more than twice normal level and or alkalin phosphatase (ALP) more than one and half times normal level and or bilirubin more than two and half times normal level. In patients with previous liver dysfunction, new episode was defined by a further increase of one of this variables more than fifty per cent. Admitted patients who did not developed liver disturbances defined the “group control”.

**Results**: During the period of study, 72 patients were admitted, from which 49 presented liver dysfunction (68%). Median age was 65 years [23 + 84], mean APACHE II and SOFA scores were respectively 17.5 ± 11.22 and 6.35 ± 4.11. Acquired liver dysfunction occurred with a median delay of one day [1 + 6]. Thirty-eight patients (81%) had hepatic cytolysis, 22 (46, 8%) had GGT elevation, 18 (38, 3%) had bilirubin elevation and nine (19, 1%) had ALP elevation. Twelve patients (25, 53%) had hepato-biliary imaging and the diagnosis of cholecystitis was retained only in one case. All patients had serology of hepatic viruses, they were all-negative except one. Fifty-nine episodes have been collected. Median number of hepatic dysfunction episodes per patient was one [1 + 2], ten patients had two episodes. Retained etiologies were- multifactorial in forty cases (sepsis, hypoxia, drug toxicity), sepsis in seven cases and drug toxicity in two cases. Compared to group control (25 patients), mortality was higher (34% vs 16%, p = 0.09) and length of stay was more prolonged (15 vs 13 days, p = 0.7) but the difference was not significant.

**Conclusion**: Liver abnormalities are common in intensive care. Multifactorial liver dysfunction were the most frequent in our population. Drug toxicity was commonly suspected but it was difficult to retain it as the only incriminated factor.

### P-178 Cirrhotic patients, especially those with neurological symptoms, display dramatically increased levels of several xenobiotics in plasma, a metabolomic study

#### Nicolas Weiss (*speaker*)^1^, Pierre Barbier Saint Hilaire^2^, Benoit Colsch^2^, Suleiman Attala^2^, Augustin Schaefer^3^, Marika Rudler^3^, Charlotte Bouzbib^3^, Foudil Lamari^4^, Junot Junot^2^, Dominique Thabut^3^

##### ^1^Sorbonne Université, Asnières-Sur-Seine, FRANCE; ^2^CEA, iBiTec-S, Service de Pharmacologie et d’Immunoanalyse, Laboratoire d’Etude du Métabolisme des Médicaments, MetaboHUB-Paris, Gif-Sur-Yvette, FRANCE; ^3^Sorbonne Université, Brain Liver Pitié-Salpêtrière (BLIPS) study group, Groupement Hospitalier Pitié-Salpêtrière-Charles Foix, Paris, France, Paris, FRANCE; ^4^Sorbonne Université, Groupement Hospitalier Pitié-Salpêtrière-Charles Foix, Paris, FRANCE

###### **Correspondence:** Nicolas Weiss - nic.weiss@wanadoo.fr

*Annals of Intensive Care* 2019, **9(Suppl 1)**:P-178

**Introduction**: Encephalopathy is a common complication of liver disease and/or portosystemic shunts. Its pathophysiology is not completely understood; mechanisms include the role of elevated ammonia levels in association with systemic inflammation. An impairment of blood–brain barrier (BBB) permeability is also hypothetised. Metabolomics enables to detect a wide range of metabolites without any a priori. In a recent metabolomic study including patients who underwent cerebrospinal fluid (CSF) collection, our group outlined that xenobiotics/drugs that usually don’t cross BBB were retrieved in the CSF, suggesting a potential neurological toxicity of drugs. CSF collection is invasive. Hence, we aimed to describe the xenobiotics present in the plasma of cirrhotic patients, using the same metabolomic approach.

**Patients and methods**: We conducted a retrospective study of plasma samples in the Hepatological ICU. Plasma samples from cirrhotic patients displaying encephalopathy were compared to plasma from cirrhotic patients without neurological symptoms, and to plasma from healthy controls. Liquid chromatography coupled to high-resolution mass spectrometry was performed and then after the metabolic fingerprints were compared to database and between the different groups.

**Results**: Plasma samples were obtained from 12 cirrhotic patients with encephalopathy (age 59 [40–68], MELD 20 [16–31], alcohol 58%), 13 cirrhotic patients without encephalopathy (age 56 [55–64], MELD 17 [14–29], alcohol 38%) and 9 healthy controls. Among 495 identified metabolites, 25 corresponded to xenobiotics or its derivatives. Fluoxetine was detected with a more than 200 fold increase, aminosalicylic acid with a more than 10 fold increase and benzyl alcohol (present in cough pills and antiseptics) with a 3 fold increase in cirrhotic patients with encephalopathy as compared to cirrhotic patients. In cirrhotic patients with or without encephalopathy, propranolol was detected with a more than 8500 fold increase, acetaminophen with a 40 fold increase, penicillamine and ampicillin both with a 2 fold increase as compared to healthy controls. Interestingly, several substances which were not expected to have systemic diffusion were detected in cirrhotic patients and in healthy controls- eugenol, isoeugenol (used in mouth bathing solution), triethanolamine (trolamin, used in cutaneous creams) and resorcinol monoacetate (used in mouth bathing solution and in cutaneous creams).

**Conclusion**: Cirrhotic patients, especially those with neurological symptoms, display dramatically increased levels of several xenobiotics in plasma. These results confirm that PK PD parameters of commonly used drugs are highly modified in those patients. This suggests a potential role of xenobiotics in the pathophysiology of encephalopathy in patients with liver diseases.

### P-179 Neutrophil- lymphocyte ratio- prognostic value in cirrhosis

#### Ameni Khaled (*speaker*)^1^, Sana Khedher^2^, Khaoula Ben Ismail^2^, Cyrine Abdennabi^2^, Mohamed Salem^2^

##### ^1^Hopital Mami Ariana, Ariana, TUNISIA; ^2^Hepato and Gastroenterology department - Intensive care unit, Charles Nicolle Hospital, Tunis, Tunisia., Ariana, TUNISIA

###### **Correspondence:** Ameni Khaled - ameni.khaled1988@gmail.com

*Annals of Intensive Care* 2019, **9(Suppl 1)**:P-179

**Introduction**: Different scoring models are used to predict the severity of Acute-on-chronic liver failure (AoCLF) as Child–Pugh, Different scoring models are used to predict the severity of Acute on chronic liver failure (AoCLF) as Child–Pugh, Model of End-Stage Liver Disease (MELD), and Clif SOFA. Previous studies had shown that Neutrophil-lymphocytr ratio (NLR) and Platelet-lymphocyte (PLR) can be used as prognostic markers in various disease processes. We aimed to evaluate the role of NLR in the prediction of 3-month mortality in patients with AoCLF.

**Patients and methods**: This was a retrospective follow up study including all patients with decompensated cirrhosis between January and December 2016.

**Results**: A total of 92 case of decompensated cirrhosis with AoCLF, were enrolled in the present study. The mean age was 62 ± 13.4 years. The sex ratio was 0, 95. The mean Child and MELD were respectively 8 and 18. Chronic hepatitis C infection remains a major case of the cirrhosis in our study (42%). The leading cause of hospitalization was an Oedemato-ascitic decompensation (40%). The diagnosis of sepsis was identified in 46% of cases. Our findings indicated that 3 month -mortality had interseted 25 patients. Univariate logistic regression analyses showed that there is a significant positive correlation between NLR and 3- months mortality (p = 0.02) also with a high MELD score (p = 0.01), Child score (p = 0.00) and Clif SOFA (p = 0.03). Multivariate logistic regression analyses proved that a high NLR was an additional independent risk factor for 3-month mortality (p = 0.02) as well as for Child score (p = 0.00). To evaluate the ability of NLR and Child scores to predict mortality, ROC curves were obtained.The AUC values were 0.841 ± 0.061 for the Child score and 0.728 ± 0.046 for NLR (both p < 0.005).

**Conclusion**: In our study, the NLR seems to be a good marker, easily achievable, to assess the prognosis immediately up on admission in decompensated cirrhosis.

### P-180 Multidrug resistant Pseudomonas aeruginosa and mortality in mechanically ventilated ICU patients

#### Jean-Baptiste Denis (*speaker*)^1^, Samuel Lehingue^2^, Vanessa Pauly^3^, Nadim Cassir^4^, Laurent Papazian^1^

##### ^1^Assistance Publique - Hôpitaux de Marseille, Marseille, FRANCE; ^2^APHM-HOPITAL NORD-Réanimation des Détresses Respiratoires et des Infections Sévères, Marseille, FRANCE; ^3^APHM-Hôpital La conception, Marseille, FRANCE; ^4^APHM - Hôpital Nord, Marseille, FRANCE

###### **Correspondence:** Jean-Baptiste Denis - jbaptiste.denis@gmail.com

*Annals of Intensive Care* 2019, **9(Suppl 1)**:P-180

**Introduction**: The combination of increased antibiotic resistance and decreased discovery of new antibiotic molecules is a serious concern in the intensive care unit. However, the link between bacterial resistance and prognosis remains controversial. The predominant pathogen causing ventilator-associated pneumonia (VAP) is Pseudomonas Aeruginosa (Pa), which has increasingly become multidrug resistant (MDR). The aim of this study was therefore to evaluate the relationship between MDR VAP Pa episodes and 30-day mortality.

**Patients and methods**: From a longitudinal prospective French multicenter database (2010–2016), Pa-VAP onset and physiological data were recorded. MDR was defined as non-susceptibility to at least one agent in three or more antimicrobial categories. To analyze if MDR episodes were associated with higher in-hospital 30-day mortality, we performed a multivariate survival analysis using the multivariate non-linear Frailty model.

**Results**: A total of 230 patients presented 280 Pa-VAP. A maximum of 3 episodes per patient was observed. 73 episodes were MDR, and 213 were susceptible. In the multivariate model, factors independently associated with 30-day mortality included age (HR, 1.02, 95% CI, 1.01–1.04, p = 0.0064), hospitalization in the 6 months preceding the first episode (HR, 2.31, 95% CI, 1.50–3.60, p = 0.0002), chronic renal failure (HR, 2.34, 95% CI, 1.15–4.77, p = 0.0196) and VAP Pa recurrence (HR, 2.29, 95% CI, 1.79–4.87, p = 0.032). Finally, MDR Pa-VAP was not associated with death (HR, 0.87, 95% CI, 0.52–1.45, p = 0.59).

**Conclusion**: This study does not identify a relationship between the resistance profile of Pseudomonas aeruginosa and mortality.

### P-181 Corynebacterium striatum nosocomial outbreak in a Belgian intensive care unit

#### Leda Nobile (*speaker*), Magali Dodemont, Charlotte Michel, Valérie Van Ruychevelt, Elodie De Groote, Jacques Creteur, Frédérique Jacobs, David Grimaldi

##### Hôpital Erasme, Bruxelles, BELGIUM

###### **Correspondence:** Leda Nobile - leda.nobile@erasme.ulb.ac.be

*Annals of Intensive Care* 2019, **9(Suppl 1)**:P-181

**Introduction**: Between October 2017 and March 2018 a nosocomial outbreak of Corynebacterium striatum occured in the ICU of our tertiary hospital. We aimed to characterize the population affected and to define the risk factors by matching equivalents controls and help to avoid futures outbreaks.

**Patients and methods**: We retrospectively identified patients with positive cultures for Corynebaterium striatum between 01 01 2016 and 01 07 2018 regardless of infectious signs. We included 3 controls for each positive patient matched on the ICU length of stay (+ - 3d), the unit of hospitalization and the presence of mechanical ventilation trying to identify risk factors for C. striatum acquisition.

**Results**: Whereas there were respectively 1 and 1 patients with C. striatum positive specimen in 2016 and in the 10 first months of 2017, we identified 11 patients with C. striatum culture between Oct 2017 and March 2018. All isolates were identified using MALDI-TOF and share the same susceptibility, being resistant to all antibiomicrobial except vancomycin. Genotyping was not performed. Our cohort included 5 (45.5%) male with a mean age of 58 years, a median of ICU stay of 22 days (IQR 15–61). Main comorbidity was cancer and diabetes in 3 (27.3%) patients followed by immunodepression, COPD, cirrhosis, and chronic kidney disease (n = 2, 18.2% each). 91% were medical admission with only one case of elective post-operative admission. Survival rate was 55.5%. All patients but one were intubated + median ventilation length was 22 days (14–42). All patients but one had positive respiratory specimen (either tracheobronchial aspiration or bronchoalveolar lavage) for C. striatum + one patient had peritoneal liquid as first positive culture and one had soft tissue colonization. 2 over 11 cases were considered as an actual infection and accordingly treated by vancomycine + both cases showed clinical recovery despite 1 microbiological failure. 45.5% had had NIV and 27.3 high flow oxygen therapy. 7 (63.6%) patients had an infection and 8 (72.7%) were treated with antibiotics before Corynebacterium positive cultures. Among those, 7 (87.5%) were treated with broad spectrum antibiotics. The characteristics of control patients are currently recorded, the data of the comparison between case and control will be shown in the congress.

**Conclusion**: C. striatum can cause micro-epidemic in the ICU in mechanically ventilated, comorbid patients with high LOS. Virulence seems low with infection in 1 5 of patients and no infection-related mortality.

### P-182 Intestinal richness and diversity are not related to relative abundance of Multidrug-resistant Gram-negative bacilli but are related to Enterococci abundance in the digestive microbiota intensive care unit patients

#### Candice Fontaine (*speaker*)^1^, Laurence Armand Lefevre^1^, Mélanie Magnan^1^, Romain Sonneville^2^, Lila Bouadma^2^, Jean-François Timsit^2^, Etienne Ruppé^1^

##### ^1^INSERM, IAME, UMR 1137, Hôpital Bichat-Claude Bernard, Paris, FRANCE; ^2^Bichat medical-infectious deseases ICU, Paris, FRANCE

###### **Correspondence:** Candice Fontaine - candicefontaine@yahoo.com

*Annals of Intensive Care* 2019, **9(Suppl 1)**:P-182

**Introduction**: Multidrug-resistant Gram-negative bacilli (MDR-GNB) infections are frequent and serious in Intensive Care Unit (ICU) patients. The intestinal microbiota is the reservoir of MDR-GNB. Besides, alterations of the intestinal microbiota, such as those related to the antibiotic intake, could be associated with nosocomial infections (including those involving MDR-GNB) and poor outcome. Our main objective was to assess the link between the intestinal colonization by MDR-GNB and the composition of this microbiota (richness and diversity).

**Patients and methods**: We performed a 2-month prospective, monocentric cohort study in the medical ICU of Bichat hospital in Paris. Patients ventilated more than 3 days and spontaneously passing feces were included. For each patient, a fecal sample was collected the day of inclusion and then twice a week. MDR-GNB (defined by third generation cephalosporins resistant Enterobacteriaceae) but also Enterococcus faecium were search by culture methods. For MDR-GNB, the relative abundance (RA) was calculated (defined by the concentration of MDR-GNB on the concentration of total Enterobacteriaceae). The composition of the intestinal microbiota was assessed by 16S profiling and we calculated richness (by the number of Operational Taxonomic Unit) and diversity (by the Shannon’s index).

**Results**: We collected 62 stool sample from 31 patients, including 18 feces for MDR-GNB (including 15 extended-spectrum beta-lactamase-producing Enterobacteriaceae and 3 cephalosporinase overproducing Enterobacteriaceae) in 13 different patients. The average RA of MDR-GNB was 57% (0.5 + 100%). Twenty-two stool samples were positive for E. faecium. We did not observe a link between the diversity and the richness of the intestinal microbiota and the MDR-GNB intestinal RA. Conversely, intestinal diversity and richness decreased along the relative abundance of reads assigned to Enterococcus sp. (p < 0.001) (figure) and when Enterococcus faecium intestinal carriage was detected (p < 0.001). We detected 7 MDR-GNB nosocomial infections (including 5 ventilator associated pneumonia) within 7 days of positive MDR-GNB stool sample but the MDR-GNB intestinal RA was not associated to the occurrence of infections caused by MDR-GNB.

**Conclusion**: The intestinal MDR-GNB relative abundance was not associated to the diversity nor the richness of the intestinal microbiota, but that of Enterococcus sp. relative abundance and Enterococcus faecium intestinal carriage were.


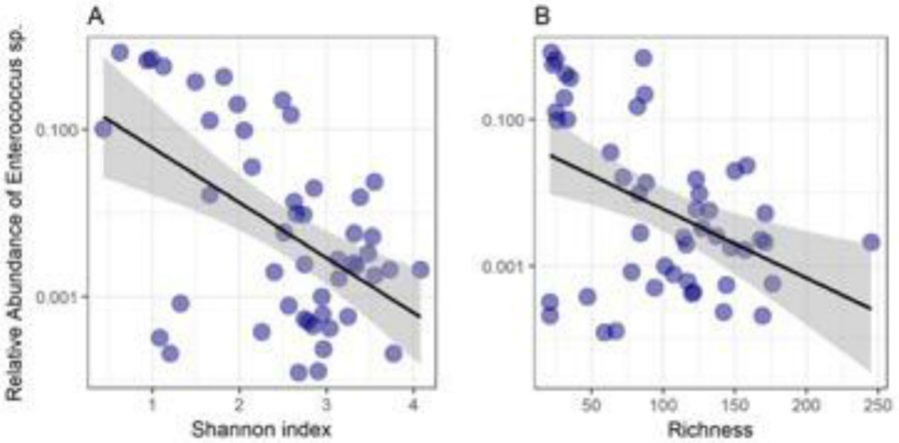


### P-183 Effects of Air Handling Unit on nosocomial infections in the Intensive-Care unit

#### Ameni Sghaier (*speaker*), Sahar Habacha, Hayfa Fazzeni, Ines Fathallah, Eya Seghir, Emna Ennouri, Asma Mehdi, Nadia Kouraichi

##### Regional Hospital of Ben Arous, Intensive care Unit, Ben Arous, TUNISIA

###### **Correspondence:** Ameni Sghaier - sghaier.ameni@gmx.com

*Annals of Intensive Care* 2019, **9(Suppl 1)**:P-183

**Introduction**: The performance of air handling unit (AHU) at reducing airborne particle transmission has been widely demonstrated in literature in terms of decrease of bacterial and fungal concentration in air and on surfaces. Our study aimed to evaluate the efficiency of AHU to reduce incidence of infections in intensive care unit (ICU).

**Patients and methods**: The annual maintenance of AHU was performed in our ICU on the 1st of January 2018. We conducted a retrospective comparative study including two groups of patients admitted for more than 48 h. -Group 1- patients admitted between October 2016 and October 2017 -Group 2- patients admitted between January and August 2018. Clinical and bacteriological data of patients were recorded to identify those who developed nosocomial infections during their ICU stay. Incidence rates were adjusted for patient-time and differences were analyzed using the Chi square test.

**Results**: Of a total of 164 patients included in our study, 54 (32.9%) belonged to group 2. Mean age was 55 ± 20 years and sex ratio was 1.92. The main etiologies of admission were acute respiratory failure (48%), coma (15.1%) and shock (7.9%). The overall incidence of nosocomial infection was 4.55 100 patient days and ventilation acquired pneumonia (VAP) had an overall incidence of 2.67 100 patient-ventilation-days, results were summarized in table 1. Catheter-related infection accounted for an overall incidence of 1.24 100 patient days and differences between the two incidence rates within the two groups were statistically significant with a preventive fraction of AHU amounting to 50.58% (table). The median time before onset of VAP was also significantly longer for patients admitted after AHU (median = 6.8 days prior to AHU Vs 13.7 days after AHU, p = 0.001).

**Conclusion**: Our study has shown that AHU appears to reduce significantly the incidence of catheter related infection. It has also shown to slow the development of VAP after intubation but had no impact on the overall incidence of nosocomial infections.


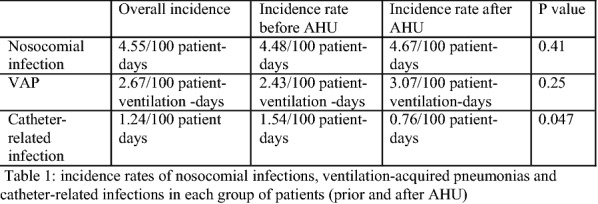


### P-184 Acinetobacter baumanii infection in ICU - how to fight it ?

#### Ameni Sghaier (*speaker*), Ines Fathallah, Hayfa Fazzeni, Sahar Habacha, Asma Mehdi, Eya Seghir, Emna Ennouri, Ghada Sboui, Khaoula Ben Ismail, Nadia Kouraichi

##### Ben Arous Regional Hospital, Intensive care Unit, Ben Arous, TUNISIA

###### **Correspondence:** Ameni Sghaier - sghaier.ameni@gmx.com

*Annals of Intensive Care* 2019, **9(Suppl 1)**:P-184

**Introduction**: Acinetobacter baumanii is an opportunistic nosocomial pathogen and an important multidrug-resistant microorganism. It is associated with a high mortality rate. Our study aimed to evaluate performance of a dual flow air handling unit (AHU) after annual maintenance and the use of a new surface and aerial disinfectant for reducing the risk of Acinetobacter baumanii infections in an intensive care unit ICU.

**Patients and methods**: We performed the annual maintenance on the AHU and we have changed surface and aerial disinfectant on the 1stof January 2018. We conducted a retrospective comparative study including two groups of patients -Group 1- patients admitted for more than 48 h between October 2016 and October 2017. -Group 2- patients admitted for more than 48 h between January and August 2018. We identified patients who developed Acinetobacter baumanii infection.

**Results**: During the study period, 164 patients were collected with a mean age of 55 ± 20 years and a sex ratio of 1.92. The median causes of admission to ICU were acute respiratory failure (48.2%), coma (15.1%) and septic shock (7.9%). The global mortality rate in ICU was 29.9% (49 cases).

We recorded 13 cases of Acinetobacter baumanii infection- ventilator associated pneumonia (n = 7), catheter related infection (n = 4) and urinary tract infection (n = 2) with a global mortality rate of 60%. Among the 13 cases, 12 were recorded before the maintenance of AHU and the use of the new surface and aerial disinfectant. These results were statistically significant (p = 0.004).

**Conclusion**: The use of a new surface and aerial disinfectant and the maintenance on the AHU has reduced significantly Acinetobacter baumanii infections.

### P-185 Acinetobacter Baumannii ventilator-associated pneumonia epidemiology, risk and prognosis factors

#### Walid Sellami (*speaker*), Zied Hajjej, Inès Ben Mrad, Hedi Gharssallah, Iheb Labbène, Mustapha Ferjani

##### Department of critical care medecine and anesthesiology, LR12DN01, Military Hospital of Tunis, Tunisia, Tunis, TUNISIA

###### **Correspondence:** Walid Sellami - drsellamiwalid@yahoo.fr

*Annals of Intensive Care* 2019, **9(Suppl 1)**:P-185

**Introduction**: Ventilator-associated pneumonia (VAP) is the most common nosocomial infection in critically ill patients, reaching up to 30 to 50%, with a high mortality rate. Acinetobacter Baumannii (AB) has emerged as a pathogen frequently incriminated in VAP’s in Tunisia. The aim of this study was to describe the epidemiological characteristics of Acinetobacter Baumannii ventilator-associated pneumonia, to identify the risk factors and the predictors of poor outcome of VAP with AB.

**Patients and methods**: A retrospective study was conducted in the intensive care unit of the Military Hospital of Tunis, from January 2016 to December 2017. All patients with VAP’s documented infection were included. VAP’s patients with AB vs VAP’s patients due to other pathogens.

**Results**: Seventy patients (10%) developed VAP. The incidence of VAP with AB was 6.28%. Previous antibiotic therapy was identified as a risk factor for Acinetobacter Baumanii-induced pneumonia, unlike the underlying disease. AB was resistant to ceftazidime in 100%, imipenem in 97.5% with sensitivity to colistin in 100% of cases. Multidrug-resistant AB accounted for 22.5% and highly resistant AB accounted for 77.5%. Patients with AB pneumonia were more frequently complicated by acute respiratory distress syndrome compared to other patients (37.5% versus 8.9%, p = 0.02), leading to higher mortality (52.5% versus 20%, p = 0.02).

**Conclusion**: The increasing incidence of VAP in multidrug-resistant and highly resistant AB predicts a high morbidity and mortality. Hence, the risk factors related to poor outcome in VAP’s need to be identified. The implementation of infection-control measures, mainly the cross-transmission, may be needed to improve outcome.

### P-186 Proteus mirabilis resistant to carbapenems in burn patients

#### Lamia Thabet (*speaker*), Beya Maamar, Yosra Bourbiaa, Khalil Jmal, Imen Rahmani, Amen Allah Messadi

##### Centre de traumatologie et des grands brûlés ben arous, Tunis, TUNISIA

###### **Correspondence:** Lamia Thabet - thabetlamia@gmail.com

*Annals of Intensive Care* 2019, **9(Suppl 1)**:P-186

**Introduction**: Carbapenemases are responsible for many therapeutic failures in addition to their epidemic power. The acquisition of this type of resistance by Proteus mirabilis, naturally resistant to tigecycline and colistin, makes the therapeutic options even more restricted. The aim of our study was to define the mechanisms of carbapenem resistance in P. mirabilis and to identify the antibiotic resistance profile of these strains.

**Patients and methods**: This study was conducted over a period of 6 months (from January to June 2017) on strains of P. mirabilis isolated in burned patients. The MICs of the carbapenems were performed by E-test^®^. Multiplex real-time PCR was performed with Cepheid’s GeneXpert Carba-R, allowing detection of the most prevalent carbapenemasegene families (blaVIM, blaNDM, blaIMP, blaOXA-48 and blaKPC).

**Results**: During the study period, 49 strains of P. mirabilis were isolated, ranking fifth among all species isolated from burned patients, and the third among the enterobacteriaceae. It was mainly found in cutaneous samples (42%), blood cultures (17%) and catheters (13%). The resistance rate to at least one carbapenem was 24% (12 out of 49), the carbapenem most often concerned being imipene (100% of cases). For 8 of 12 strains, imipenem was the only affected carbapenem. Four strains were resistant to ertapenem. All these isolates were sensitive to meropenem. Except carbapenems, these germs expressed high levels of resistance to almost all antibiotics- 11 of 12 were resistant to fluoroquinolones, also 11 12 to aminoglycosides and fosfomycin. The C3G resistance rate was 100%, and 50% of the strains were susceptible or intermediate to cefepime. Of these 12 isolates, 9 were sensitive or intermediate to the piperacillin-tazobactam and all were sensitive to aztreonam. All of these strains were sensitive to temocillin and the EDTA test was positive for 11 of 12. Eleven of these isolates had hyperproduced cephalosporinase, and one an ESBL. Molecular identification showed that 11 samples tested had blaNDM gene and one, which expressed ESBL, was negative.

**Conclusion**: The presence of carbapenemases in P.mirabilis is alarming. In our study, ertapenem does not have the best sensitivity for their detection. Faced with the risk of therapeutic impasse, preventive measures remain the best solution.

### P-187 Infections caused by Gram-negative bacilli with reduced sensitivity to carbapenems- risk factors, clinical features and prognosis

#### Mayssa Daiki (*speaker*)^1^, Rym Jeljli^2^, Emel Rafrafi^3^, Walid Samoud^4^, Olfa Yengui^4^, Walid Sellami^4^, Mohamed Selim El Asli ^5^, Zied Hajjej^4^, Mohamed Ben Moussa^5^, Mustapha Ferjani^4^

##### ^1^Faculty of Medecine od Tunis, University Tunis Elmanar, Military Hospital, Tunis, TUNISIA; ^2^Faculty of pharmacy of Monastir, Tunis, TUNISIA; ^3^Faculty of Medecine of Tunisia, Tunis, TUNISIA; ^4^Faculty of Medecine of Tunis, critical care department, Military hospital of Tunis, TUNISIA; ^5^Faculty of pharmacy of Monastir, microbiology laboratory, Military hospital of Tunis, TUNISIA

###### **Correspondence:** Mayssa Daiki - mayassamed1@gmail.com

*Annals of Intensive Care* 2019, **9(Suppl 1)**:P-187

**Introduction**: Antibiotic resistance has become a major public health problem worldwide. Currently, the drastic increase of the carbapenem resistance in Gram-negative bacilli (CRGNB) is a major threat because not only of their fast spread rate but also their high morbidity and mortality rate.

**Patients and methods**: This is a retrospective study in which we investigated the epidemiological profile and prognosis of Gram-negative bacilli with reduced sensitivity to carbapenems (GNBRSC) among 50 patients admitted to the intensive care unit in the military hospital of Tunis during a period of six months (January-June 2017). EUCAST carbapenem breakpoints were used to interpret susceptibility to carbapenems.

**Results**: Acinetobacter baumanii was the most isolated bacterial species (63%), followed by Pseudomonas aeruginosa (13%) and Klebsiella pneumonia (12%). The most common infections were ventilator associated pneumonia (52%), bacteremia (12%), suppurative infections (8%), urinary tract infections (12%) and pneumopathies (8%). The resistance of Gram-negative bacilli to carbapenems is associated with resistance to most antibiotics, only colistin remains active on all naturally sensitive organisms. The risk factors for the acquisition of these infections are the advanced age, the state of immunosuppression of the patients, a long duration of hospitalisation, prior colonisation with GNBRSC and specially a previous taking of antibiotics during the last 3 months. Mortality within 30 days of infection onset was 30%. By multivariate analysis, predictors of in-hospital mortality were Sepsis-related Organ Failure Assessment (SOFA) score at the day of infection (p = 0.001), inadequate empirical antimicrobial therapy (p = 0.03), chronic cardiac failure (p = 0.04).

**Discussion**: In our series, the epidemiological and microbiological profile of GNBRSC was comparable to litterature data. CRGNB screening and confirmation requires a combination of phenotypic and genotypic tests to increase accuracy of detection which were not performed in our series.

**Conclusion**: The emergence of CRGNB should call for the rigorous application of the main preventive measures, the reinforcement of hygiene measures and the strict monitoring of these highly resistance bacteria. Adequate source control and appropriate use of antimicrobial therapy are the most important modifiable risk factors that have shown a survival benefit in this population.

### P-188 Immunomodulatory effects of antibiotics during septic shock: the endothelial cell’s role

#### Stéphanie Pons (*speaker*)^1^, Maud Loiselle^1^, Manel Nouacer^1^, Julien Lion^1^, Karine Poussin^1^, Elie Azoulay^2^, Nuala Mooney^1^, Lara Zafrani^2^

##### ^1^U1160 INSERM- Hôpital Saint-Louis, Paris, FRANCE; ^6^Médecine Intensive Réanimation-Hôpital Saint Louis, Paris, FRANCE

###### **Correspondence:** Stéphanie Pons - pons.stephanie0@gmail.com

*Annals of Intensive Care* 2019, **9(Suppl 1)**:P-188

**Introduction**: Sepsis is defined as the host’s inflammatory response to a life-threatening infection potentially leading to a septic shock and the failure of organs initially not infected. Besides their antimicrobial effect, some antibiotics could have intrinsic immunomodulatory properties. Endothelial cells are able to detect danger signals from pathogens and to initiate the innate immune response, and in some specific conditions, the adaptive immunity. The aim of our study was to highlight the effects of three antibiotic classes on endothelial cells’ immune properties during sepsis.

**Patients and methods**: Human Microvascular Endothelial Cells (HMEC) were stimulated by Lipopolysaccharide, Interferon gamma and Tumor Necrosis Factor alpha (Cytomix) during 24 h before the administration of different antibiotics. The surface expression of Inter-Cellular Adhesion Molecule 1 (CD54), Program-Death Ligand 1 (CD274), Human Leukocyte Antigens (HLA) class 1 and HLA-DR were determined using flow cytometry 24 h after antibiotics’ administration. The production of cytokines such as Interkeukin (IL)-6 or IL-8 by HMEC was determined by Enzyme-Linked Immunoabsorbent Assay (ELISA). Quantitative polymerase chain reaction (qPCR) was used to analyze GAPDH (control), IL-6, HLA-A and HLA-DR genes expression.

**Results**: Compared to Cytomix alone, spiramycine (1 ug mL) associated to Cytomix significantly decreased the expression of HLA-1 (Mean Fluorescence Intensity (MFI) 52649 versus 98701 p < 0.01) and HLA-DR (MFI 355 versus 562 p < 0.01) on HMEC surface. Clarithromycin and erythromycin also significantly decreased both expressions of HLA-1 and HLA-DR. In qPCR, clarithromycin significantly decreased HLA-DR gene expression (1 versus 0.78 HLA-DR fold expression to GAPDH p < 0.01) and IL-6 gene expression (1 versus 0.67 IL-6 fold expression to GAPDH p = 0.03) compared to Cytomix (Figure 1). Compared to Cytomix, piperacillin-tazobactam (1 ug mL) significantly decreased the expression of HLA-1 on HMEC surface (p < 0.02) but did not significantly affect HLA-DR expression. Vancomycin did not change the expression of CD 54, CD 274, HLA-1 and HLA-DR on HMEC surface. Under Cytomix stimulation, IL-6 and IL-8 production by HMEC were not significantly modified by antibiotics administration (macrolides, penicillin, glycopeptides).

**Conclusion**: Macrolides and Penicillin modulate the expression of HLA molecules on microvascular endothelial cells’ surface. The use of co-cultures of endothelial cells and peripheral blood mononuclear cells may enlighten the pathophysiological implications of these results.


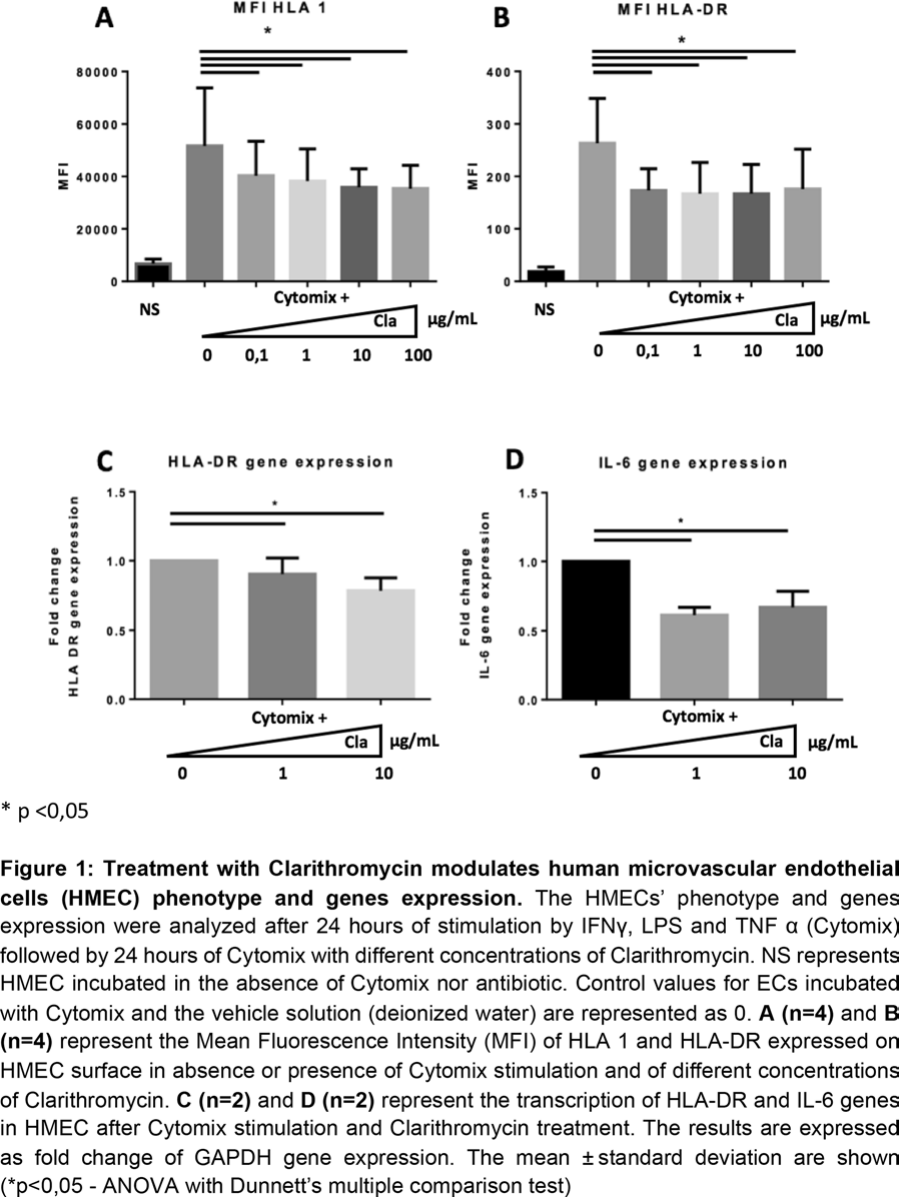


### P-189 Gene expression of endoplasmic reticulum stress during septic shock in human

#### Thomas Clavier (*speaker*), Pierre-Alain Thiebaut, Emmanuel Besnier, Pressat-Laffouilhere Thibaut, Steven Grangé, Dorothée Carpentier, Christophe Girault , Gaetan Beduneau, Sylvanie Renet, Vincent Richard,Benoit Veber, Fabienne Tamion

##### Rouen University Hospital, Rouen, FRANCE

###### **Correspondence:** Thomas Clavier - thomasclavier76@gmail.com

*Annals of Intensive Care* 2019, **9(Suppl 1)**:P-189

**Introduction**: During inflammation, endoplasmic reticulum protein folding capacities are surpassed leading to the accumulation of unfolded peptides, a process known as endoplasmic reticulum stress (ERS). In murine models of sepsis, ERS blockers decrease expression of inflammatory cytokines and reduce organ dysfunction and mortality. The objective of this work was to search for a link between the gene expression of ERS and organ failure in patients with septic shock.

**Patients and methods**: We conducted a prospective study in a tertiary care hospital which included patients admitted to intensive care unit (ICU) for septic shock. Blood samples were collected on days 1 (D1), 3 and 5 (D5) of ICU stay. After extraction of total RNA, quantitative PCR was performed to evaluate the expression of genes coding for key elements of ERS- HSPA5 (coding for GRP78), ATF6, ATF4, CHOP, sXBP1 or for the endothelium-related markers (ICAM1 and ET-1). The main objective was the correlation of HSPA5 expression with Sequential Organ Failure Assessment (SOFA) score (Spearman method).

**Results**: We included 41 patients with a median age of 67 [57–73] years, a median SAPSII of 50 [39–61], a median length of stay of 8 [5–16] days and a mortality rate of 14.6%. There was no correlation between HSPA5, ATF4, sXBP1 and CHOP expression and SOFA score. However, a correlation between SOFA score and ATF6 expression was observed (r = 0.27 [0.08 + 0.43]), as well as with expression of endothelial aggression markers ICAM1 and ET-1 (r = 0.23 [0.03 + 0.41] and r = 0.30 [0.11 + 0.47] respectively). CHOP, ATF6 and HSPA5 were correlated with the expression of ICAM1 and ET1. ATF6 expression followed SOFA variation between D1 and D5 (r = 0.40 [0.02 + 0.69]).

**Conclusion**: Our study has evaluated for the first time the expression of ERS markers in patients with septic shock, revealing that some ERS markers might be partly correlated with the severity of septic shock and with endothelial dysfunction.

### P-190 Effects of enteral administration of citrulline in the treatment of sepsis induced immunosuppression in mice

#### Florian Reizine (*speaker*)^1^, Murielle Gregoire^2^, Elise Dessauge^2^, Arnaud Gacouin^1^, Caroline Piau^2^, Cécile Le Naoures^2^, Valerie Bordeau ^3^, Christian Michelet^1^, Yves Le Tulzo^1^, Karin Tarte^4^,Jean-Marc Tadie^1^

##### ^1^CHU Rennes, Maladies Infectieuses et Réanimation Médicale, Rennes, FRANCE; ^2^CHU Rennes, Pôle Biologie, Rennes, France, Rennes, FRANCE; ^3^Université de Rennes 1, Inserm U1230-UPRES EA 2311, Biochimie Pharmaceutique, Regulatory RNA and Medicine (RMM), Rennes, FRANCE; ^4^Inserm U1236, université de Rennes 1, Rennes, FRANCE

###### **Correspondence:** Florian Reizine - florian.reizine@chu-rennes.fr

*Annals of Intensive Care* 2019, **9(Suppl 1)**:P-190

**Introduction**: The widespread recognition that critical illness is characterized as a state of immunosuppression has led to the development of nutritional support products or interventions designed to enhance the host immune response. While immune modulating diets (IMDs) containing nutrients displaying immunoregulating properties such as arginine are conceptually appealing, data from individual trials and several meta-analyses have failed to produce convincing results. Different studies found that early enteral administration of l-arginine to patients in ICU increased ornithine synthesis suggesting a preferential use by the arginase pathway, while citrulline NO synthesis was barely stimulated and immune functions were unaffected. Recent data suggest that citrulline supplementation may be more efficient to increase arginine availability and NO production than arginine supplementation. Our hypothesis is that supplementation with citrulline is more efficient than arginine to increase systemic arginine availability and decrease post-sepsis immunosuppression.

**Patients and methods**: Using C57Bl6 mice, we performed a cecal ligation and puncture (CLP). At day 5 mice were sacrificed after performing cardiac puncture. We monitored immune function, especially those associated with impaired outcomes and nosocomial infections acquisition- lymphocyte apoptosis, MDSCs, regulatory T-Cells. In a second set of experiments, we performed a secondary infection (pneumonia) at day 5 after CLP. Severity of pulmonary infection was assessed using bronchoalveolar lavage and histological analysis. Of note, spleen and kidney were harvested to evaluate bacterial dissemination. These experiments were performed in 3 different groups of mice- citrulline (200 g kg j), arginine (200 g kg j) or an isonitrogenous placebo diets during five days following CLP. Continuous variables represented as mean ± SD were compared using Student t test. P < 0.05 indicated statistically significant differences.

**Results**: Citrulline supplementation was associated with a diminished proportion of apoptotic lymphocytes and a lower recruitment of regulatory T-cells. We observed a trend towards hypoargininemia correction and a higher citrullinemia in the citrulline group. Assessment of MRSA pneumonia severity showed a reduced bacterial load in BAL of citrulline fitted mice. Bacterial dissemination was also significantly lower in this group.

**Conclusion**: Enteral administration of citrulline was associated with a less pronounced post septic immunoparalysis and a diminished susceptibility to secondary infections in a murine two hit model of sepsis.

### P-191 Remodeling and reprogramming of tissue-resident alveolar macrophages after recovery of lung infection

#### Antoine Guillon (*speaker*)^1^, Emad Arafa^2^, Kimberly A. Barker^2^, Anna C. Belkina^3^, Ian Martin^2^, Anukul T. Shenoy^2^, Alicia K. Wooten^2^, Carolina Lyon de Ana^2^, Jaileen Hernandez Escalante^4^, Hans Dooms^4^,Katrina E. Traber^2^, Matthew R. Jones^2^, Lee J. Quinton^2^, Joseph P. Mizgerd^2^

##### ^1^Pulmonary Center, Boston University School of Medicine + collaboration with service de Médecine Intensive Réanimation, CHRU Tours, Boston, UNITED-STATES; ^2^Pulmonary Center, Boston University School of Medicine, Boston, UNITED STATES; ^3^Flow Cytometry Core Facility, Department of Pathology and Laboratory Medicine Boston University School of Medicine, Boston, UNITED STATES; ^4^Department of Medicine, Arthritis Center Rheumatology Section, Boston University School of Medicine, Boston, UNITED STATES

###### **Correspondence:** Antoine Guillon - antoine.guillon@univ-tours.fr

*Annals of Intensive Care* 2019, **9(Suppl 1)**:P-191

**Introduction**: Community-acquired pneumonia is a widespread disease with significant morbimortality. Pneumonia rates drop during childhood and then remain low for decades, until they rise again in the elderly. The dogma that only adaptive immunity builds immunological memory has recently been challenged + emerging evidences indicated that innate immune cells display long-term changes after infection. Alveolar macrophages (AM) are tissue-resident lung cells that play a crucial role in innate immunity. Our current understanding of AM is based on studies conducted in animals without preexisting conditions. We hypothesized that AM can display adaptive characteristics after resolution of pneumonia. In this study, we described to what extent AM change after recovery of lung infection in mice.

**Patients and methods**: Mice were infected with 2 intranasal or left bronchial instillations of Streptococcus pneumoniae (Sp 19F) with one week intervals between infections. Infections were self-limiting and mice were studied 4–6 weeks later (named thereafter Veteran or Naïve for control mice). Single-cell suspensions were generated from lungs and assessed by flow cytometry. Data were analyzed using traditional manual 2D gating. In addition, an unsupervised computational approach (t-SNE) was used for dimension reduction of the data and analysis. AM were sorted for transcriptome analysis. Veteran and Naive mice were subsequently infected with lethal dose of Sp 3 with without AM depletion induced by clodronate.

**Results**: Lung-resident myeloid cell populations were identical in Veteran and Naïve mice as defined by manual gating. However, examination of t-SNE-visualized map of the dataset revealed a difference in one cluster corresponding of the AM. We identified multiple subtle changes in the immunophenotype of Veteran AM including decreased SiglecF expression, increased MHCII and CD64 expression. This remodeling was- (i) long-lasting (still observed 6 months post infection) and (ii) regionally localized (only observed in the left lungs of mice infected through the left bronchus). Veteran newly infected by Sp3 had better bacterial clearance compared to Naïve + however, this enhanced protection was not observed in Veteran mice with AM depletion. Finally, transcriptome profiling demonstrated marked differences between Naive and Veteran AM, including immune functions, cell cycle and metabolism.

**Conclusion**: AM are remodeled and reprogrammed after mild and self-limiting respiratory infection, these changes being long-lasting, compartmentalized and associated with enhanced lung protection. Determining how Veteran AM respond differently to subsequent infections may offer new mechanisms to understand what is altered in adults with high susceptibility to pneumonia

### P-192 Histone deacetylases inhibition significantly alters gene expression that regulate T-cell apoptosis and exhaustion in a murine model of sepsis-induced immunosuppression

#### Fanny Alby-Laurent (*speaker*)^1^, Ariane Gavaud^1^, Marine Torta^1^, Christophe Rousseau^1^, Clara Vigneron^1^, Laurence Romy^1^, Jean-Paul Mira^2^, Fréderic Pène^1^, Julie Toubiana^1^, Jean-Daniel Chiche^2^

##### ^1^Institut Cochin, Paris, FRANCE; ^2^Hôpital Cochin - HUPC, Paris, FRANCE

###### **Correspondence:** Fanny Alby-Laurent - fanny_alby@hotmail.com

*Annals of Intensive Care* 2019, **9(Suppl 1)**:P-192

**Introduction**: Sepsis induces long lasting alterations of transcriptional programs that lead to sepsis-induced immune suppression (SIIS), secondary infections and death. A shift toward repressive histone modifications with overexpression of histone desacetylases (HDACs) is observed in patients with SIIS. We have shown that HDACs inhibition with trichostatin A (TSA) reduces mortality, improves bacterial clearance and prevents lymphocytes apoptosis in a relevant murine model of SIIS. To investigate epigenetic changes induced in this model and reversed by TSA, we performed transcriptional analysis in splenocytes and studied H3 acetylation in T-cells.

**Patients and methods**: We used a model of SIIS following polymicrobial sepsis.1 Briefly, C57BL 6 mice were treated with TSA (2 mg kg ip) or not (CTL) before cœcal ligation & puncture (CLP). Surviving mice underwent intratracheal instillation of 2x106 CFU of Pseudomonas aeruginosa 8 days after CLP. For gene-expression studies, microarray experiments were performed using Affymetrix Clariom S Mouse genome-wide array after RNA isolation from isolated T-cells after CLP (Days 1&8). We identified differentially expressed genes using ANOVA for each gene, then used unadjusted p-value and fold changes to filter and select differentially expressed genes. We used mean centered data to perform samples and genes classification by hierarchical clustering, using the Spearman correlation similarity measure and average ward linkage algorithm. Diseases & functions representing key genes were identified using Ingenuity software. In T-cells, we used Luminex Histone PTM Multiplex assay to evaluated the effect of TSA on global H3 acetylation with focus on 13 acetylation marks.

**Results**: More than 60 genes were differentially expressed between CLP TSA-treated mice and CLP mice 1 day after CLP. This effect was more pronounced at D8 of sepsis (> 250 genes differentially expressed). At day 8 of sepsis, TSA up-regulates 15 genes down-regulated by CLP and down-regulates 2 genes up-regulated by CLP. 12 of these genes were directly involved in immune responses (Fig 1A) modulated by TSA in our model (including T-cell apoptosis and T-cell expression of PD1 and PD-L1). Other genes affected are genes involved in cellular movement, immune cell trafficking, inflammatory response, antimicrobial response and cell death and survival (Fig 1B). Pan-histone 3 acetylation was significantly increased in T-cells 1 day after sepsis.

**Conclusion**: Treatment with TSA significantly alters histone 3 acetylation and expression of 17 genes involved in the modulation of cellular function linked to SIIS, T-cell apoptosis and exhaustion.


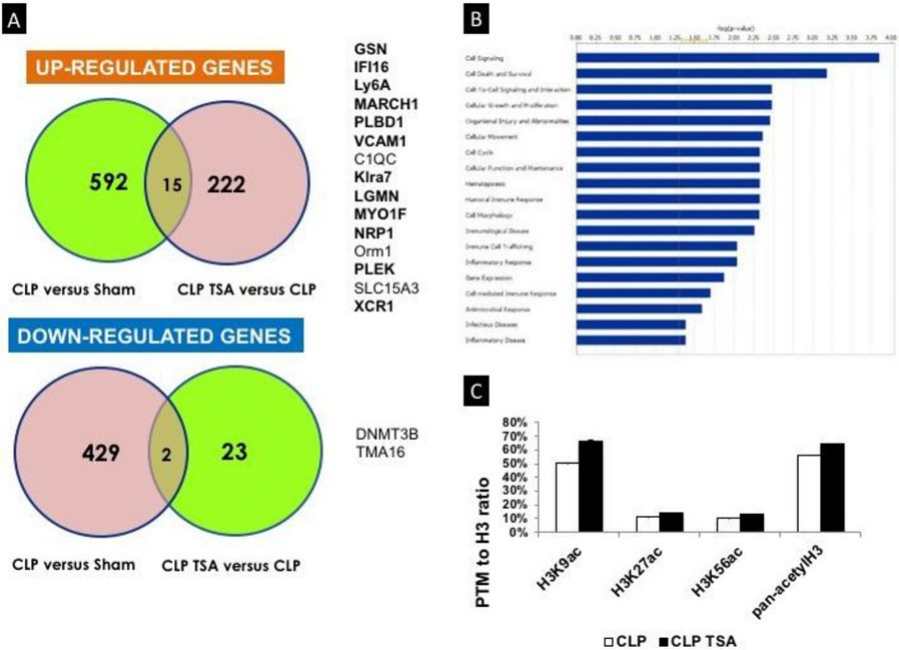


### P-193 Immune dysfunctions and increased susceptibility to staphylococcus aureus infection following cecal ligation and puncture

#### Florian Reizine (*speaker*)^1^, Murielle Gregoire^2^, Elise Dessauge^2^, Arnaud Gacouin^1^, Caroline Piau^2^, Valerie Bordeau^3^, Cécile Le Naoures^2^, Christian Michelet^1^, Karin Tarte^4^, Yves Le Tulzo^1^,Jean-Marc Tadie^1^

##### ^1^CHU Rennes, Maladies Infectieuses et Réanimation Médicale, Rennes, FRANCE; ^2^CHU Rennes, Pôle Biologie, Rennes, FRANCE; ^3^Université de Rennes 1, Inserm U1230-UPRES EA 2311, Biochimie Pharmaceutique, Regulatory RNA and Medicine (RMM), Rennes, FRANCE; ^4^Inserm U1236, université de Rennes 1, Rennes, FRANCE

###### **Correspondence:** Florian Reizine - florian.reizine@chu-rennes.fr

*Annals of Intensive Care* 2019, **9(Suppl 1)**:P-193

**Introduction**: Sepsis initiates a complex immune response with the concomitant occurrence of both pro and anti-inflammatory reaction that can lead to an increased susceptibility to secondary infections which represent a significant cause of mortality and morbidity. Although we observed a significant decrease in sepsis associated mortality over the last decade, clinical trial of adjuvant therapies failed to demonstrate an improvement in mortality despite significant effect in animal studies. These discrepancies might be due to the lack of recommendations in pre-clinical sepsis models. To enhance translational value of sepsis models in experimental studies, guidelines have been recently published (1). However, there is no data regarding the effectiveness of such recommendations. The aim of our study is to demonstrate that, when following newly published Minimum Quality Threshold in Pre-Clinical Sepsis Studies, a mouse model of cecal ligation and puncture (CLP) can mimic sepsis induced immunosuppression that worsen secondary infections.

**Patients and methods**: C57Bl6 mice were used to perform CLP and compared to a control group (sham). At day 5 to 7 mice were sacrificed after performing cardiac puncture. We monitored immune function, especially those associated with impaired outcome and nosocomial infections acquisition- lymphocyte apoptosis, MDSCs, regulatory T-Cells, blood phenotype and T-lymphocytes proliferation index.

A second group of mice were used to study the severity of a secondary infection (MRSA pneumonia) at day 5 after CLP or sham surgery. 12 h later, severity of infection was assessed. Continuous variables represented as mean ± SD were compared using Student T test. P < 0.05 indicated statistically significant differences.

**Results**: We found a significant increase of G-MDSC, M-MDSC, Regulatory T-Cells, monocytes and neutrophils recruitment in CLP mice. We also observed a higher concentration of apoptotic lymphocytes and a diminished lymphocyte proliferation index in this group. MRSA pneumonia was more severe in CLP mice as bacterial load in BAL and MRSA bacteremia (concentration in harvested spleen and kidney) were significantly higher than in sham group.

**Conclusion**: We observed that, when newly published guidelines are followed, CLP induces immune dysfunction which is responsible for more severe secondary infection. Standardization of animal models of sepsis should improve their translational value.

**Reference**

1. Osuchowski, Marcin F., et al. “Minimum Quality Threshold in Pre-Clinical Sepsis Studies (MQTiPSS).” Shock, 2018, p. 1., 10.1097/shk.0000000000001212.

### P-194 Contribution of lung ultrasound in early diagnosis of ventilator-associated pneumonia

#### Walid Sellami (*speaker*), Zied Hajjej, Dhekra Sebki, Inès Ben Mrad, Hedi Gharssallah, Iheb Labbène, Mustapha Ferjani

##### Department of critical care medecine and anesthesiology, LR12DN01, Military Hospital of Tunis, Tunisia, Tunis, TUNISIA

###### **Correspondence:** Walid Sellami - drsellamiwalid@yahoo.fr

*Annals of Intensive Care* 2019, **9(Suppl 1)**:P-194

**Introduction**: Pneumonia is usually presented as a forgotten killer. Ventilator-associated pneumonia represent the most frequent nosocomial infection in the intensive care unit. Their diagnosis remains problematic and an early diagnosis could largely improve the prognostic outcomes. Several clinical, radiologic and biologic tools were developped to impove the speed and the diagnostic performance. Recently, lung ultrasound became a tool allowing to estimate the lung morphology at the bedside. The purpose of this study was to determine the sensibility, the specificity and the performance of diagnosis of lung ultrasound alone and associated to bronchoalveolar lavage.

**Patients and methods**: In a monocenter prospective study of 60 patients with suspected ventilator-associated pneumonia, we investigated the diagnostic performance of lung ultrasound and the sensibility and specificity of the signs ultrasound of pneumonia which are - subpleural consolidation, lobar consolidation and arborescent linear air bronchogram. We also evaluated the combination of lung ultrasound with direct examination of bronchoalveolar lavage.

**Results**: The prevalence of ventilator-associated pneumonia was 60%. The two groups (patients with and without Ventilator-associated pneumonia) were similar in terms of general characteristics. The only significant differences between the two groups occurred in purulent secretions. Lobar hemilobar consolidation had a specificity of 33% while subpleural consolidation and arborescent linear air bronchogram had a specificity of 100% and a positive predictive value of 100%. The association between lung ultrasound and bronchoalveolar lavage had a specificity of 100% and a positive predictive value of 100%.

**Conclusion**: The lung ultrasound is a vailable tool of practical bedhead among critical patients in intensive care unit. It is a valid alternative for the early and reliable diagnosis of ventilator-associated pneumonia. She could also allow to follow their evolution under treatment.

### P-195 Development of dosing nomograms for amikacin first dose in critically-ill patients

#### Anne Coste (*speaker*)^1^, Grégoire Matthieu^2^, Guillaume Deslandes^3^, Ronan Bellouard^2^, Laurence Jalin^4^, Claire Roger^5^, Cédric Bretonnière^6^

##### ^1^Brest Hospital, Brest, FRANCE; ^2^Service de pharmacologie clinique - Hôpital Hôtel Dieu, Nantes, FRANCE; ^3^Hopital Dieu - Service de pharmacologie clinique, Nantes, FRANCE; ^4^Unité de neuro-anesthésie réanimation, Paris, FRANCE; ^5^Service de réanimation chirurgicale, Nîmes, FRANCE; ^6^Service de réanimation médicale, Nantes, Nantes

###### **Correspondence:** Anne Coste - annecoste89@gmail.com

*Annals of Intensive Care* 2019, **9(Suppl 1)**:P-195

**Introduction**: French guidelines recommend to reach an amikacin concentration 1 h after beginning the infusion C1 h ≥ 8 to 10 times the Minimal Inhibitory Concentration (MIC) of the responsible bacterium. For probabilistic therapy, MIC is considered to be 8 mg L. This target is rarely achieved in the ICU despite a 30 mg kg recommended dosage. We aimed to elaborate nomograms guiding clinicians in choosing the right first amikacin dose for ICU patients in septic shock.

**Patients and methods**: Data from two prospective French cohorts of ICU patients treated with amikacin for sepsis were analyzed. Two pharmacokinetic models, one parametric and one non-parametric, were built using a population approach with Monolix^®^ and Pmetrics^®^ softwares, respectively. Using the non-parametric model, we produced dosing nomograms with Monte-Carlo simulations. The amikacin amount needed to achieve a C1 h ≥ 8 × MIC and C1 h ≥ 10 x MIC according to the model covariates was depicted.

**Results**: We analyzed 407 observations from 138 patients. Median Simplified Acute Physiology Score II was 43. Ninety-four patients (68.6%) required invasive mechanical ventilation and 79 patients (57.7%) needed vasopressors. Two-compartment pharmacokinetics models with total body weight (TBW) influence on central compartment volume and creatinine clearance according to the Chronic Kidney Disease - Epidemiology Collaboration (CKD-EPI) formula were chosen. The non-parametric and parametric models adequately described C1 h and had similar performances. Coefficient of correlation R2 was 0.963. Dosing nomograms were elaborated according to patient’s TBW and creatinine clearance. Recommended amikacin doses to achieve a C1 h ≥ 64 mg L ranged from 1900 mg to 5000 mg and from 30 mg kg to 58 mg kg.

**Conclusion**: The first amikacin dose should depend on the patient’s TBW and creatinine clearance, resulting frequently in dose ≥ 30 mg kg. External validation of the nomograms and evaluation of amikacin toxicity with high doses is necessary before using these nomograms in clinical practice.


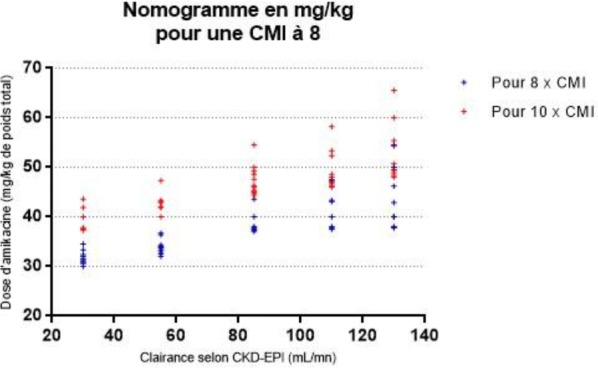


### P-196 Severe Bronchiolitis in Critical Ill Children - Impact of the Identified Viruses on Outcomes

#### Michael Thy (*speaker*), Marie-Chlotilde Orcel, Florence Moulin, Mehdi Oualha, Sylvain Renolleau

##### CHU Necker-Enfants Malades, Paris, FRANCE

###### **Correspondence:** Michael Thy - michael245thy@gmail.com

*Annals of Intensive Care* 2019, **9(Suppl 1)**:P-196

**Introduction**: Bronchiolitis is still one of the most leading causes of morbidity and mortality in children (1,2). Advances in molecular diagnosis have led to increased testing for single and multiviral respiratory infection in routine clinical practice (3,4). The aim of the study was to identify the viral identification on the outcomes for severe bronchiolitis requiring non-invasive ventilation (NIV) or high-flow nasal cannula oxygen (HFNC).

**Patients and methods**: We reviewed all cases of bronchiolitis requiring NIV or HFNC in our pediatric intensive care department during 2016 and 2017. The organisms detected by a naso-pharyngeal swab for a panel included in the Multiplex PCR assay (RespiFinder). The data were collected from our electronic medical record (EMR). The principal studied outcome was the rate of NIV failure defined as the rate of intubation. The secondary outcomes were length of hostpital stay, length of ventilation and mortality.

**Results**: 152 cases of bronchiolitis were treated by NIV in our center during the period of study. A viral identification was done in 88.8% (n = 135) of the cases of bronchiolitis (Table 1). 71.6% (n = 106) have been under NIV with 28.4% (n = 42) under HFNC. 27.7% (n = 41) had viral bacteria co-infections who were significantly more susceptible to get intubated with 5 patients intubated against 1 patient in the group without bacterial co-infection (p = 0.008). 10.7% (n = 19) were co-infected by another virus but no significant difference was found on the studied outcomes. Rhino Enterovirus was associated with longer length of stay, especially on heart disease, with a larger temporal distribution during the year.

**Conclusion**: We found a higher rate of NIV failure in the patients having viral bacteria co-infections but no differences between viral co-infections. In addition to the medical background, the type of the virus and the multiple infections especially bacterial should lead to better care of the children at high risk of complications including the risk of NIV failure.

### P-197 Amikacin dosing of 30 mg kg in critically ill children- do we achieve the peak plasmatic target?

#### Mathieu Genuini (*speaker*)^1^, Mehdi Oualha^2^, Rym Hanna^3^

##### ^1^Hôpital Robert-Debré, Paris, FRANCE; ^2^Hôpital Necker, Paris, FRANCE; ^3^Bry Sur Marne, FRANCE

###### **Correspondence:** Mathieu Genuini - mathieu.genuini@hotmail.fr

*Annals of Intensive Care* 2019, **9(Suppl 1)**:P-197

**Introduction**: Amikacin efficacy required Cmax between 60–80 mg l corresponding to 8–10 times the minimal inhibitory concentration (MIC) breakpoint for sensitive strain. To reach plasma peak target, 25–30 mg kg of amikacin is recommended in patients with altered pharmacokinetics properties, leading to underdosage in 20–30% critically ill adult. We aimed to assess the incidence and predictive factors of Cmax < 60 mg L in a population of critically ill children.

**Patients and methods**: All children admitted in the PICU located within two French tertiary academic pediatric hospitals receiving amikacin at a dose of 30 mg kg were included in a retrospective observational study between November 2017 and June 2018.

**Results**: Clinical and biological data, amikacin dosing information, and plasma concentrations were recorded. The target Cmax was between 60–80 mg L. Risk factors of Cmax < 60 mg L were identified by univariate analysis. Cmax was < 60 mg L and between 60–80 mg L in 16 (67%) and 8 (23%) of the 24 included patients, respectively. None had Cmax > 80 mg L. 13 14 (93%) patients with measured MIC achieved the pharmacokinetic pharmacodynamic ratio of 8xMIC. Risk factor for Cmax < 60 mg L was low blood urea concentration with p = 0.01. Amikacin prescription was based on admission weight, which was 10% less than actual weight. AKI occurred in 12 (50%) of patients after amikacin injection, related to severe sepsis and use of nephrotoxic agents. Trough concentration was > 2.5 mg L in 40% of patients measured Cmin.

**Conclusion**: Despite the use of the maximal amikacin dose of 30 mg kg, Cmax were below pharmacokinetic target in 67% of our population. None of them suffered from amikacin overexposure. Dosing strategy of amikacin should be optimized using actual weight rather than dry weight and the maximal dose of 30 mg kg. Regarding high prevalence of AKI, the maximal dose of amikacin should concern patients with altered pharmacokinetics properties. Trough concentration should be systematically monitored in patients receiving high doses of aminoglycosides.


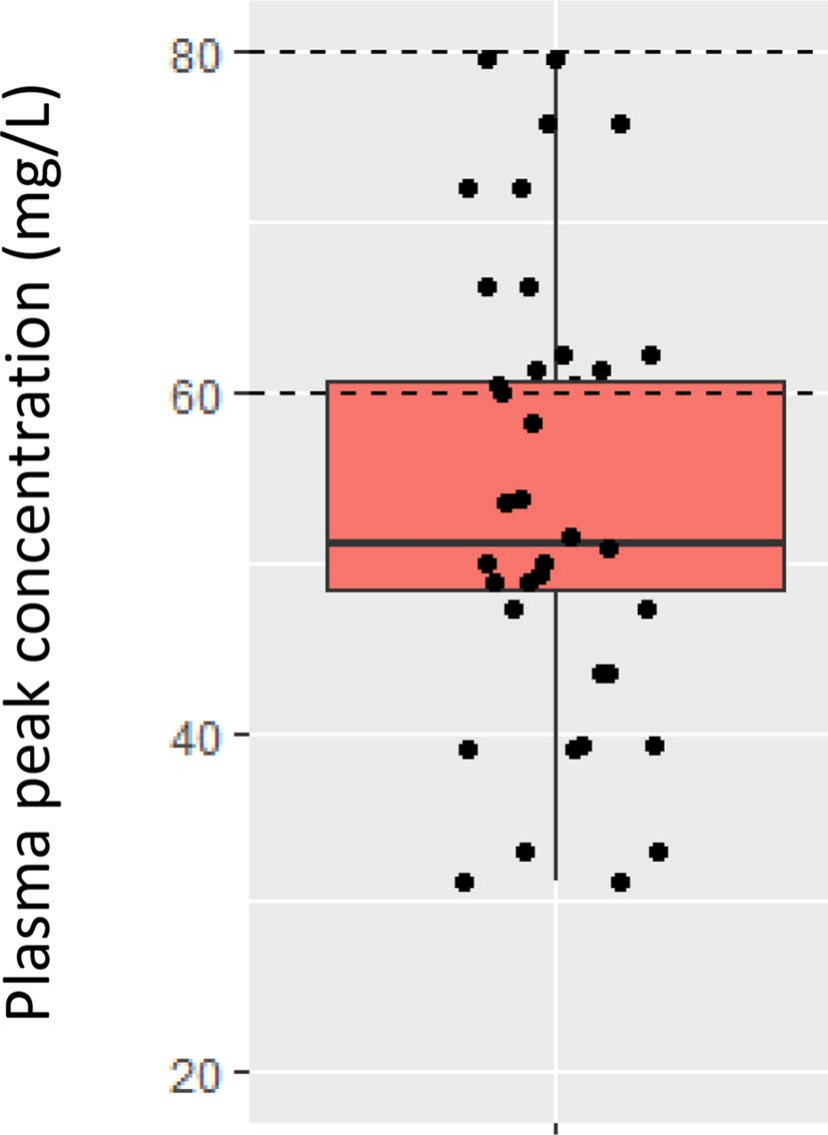


### P-198 Physiological basis regarding flow setting with High Flow Nasal Cannula (HFNC) in infants with mild acute viral bronchiolitis: “Peak tidal inspiratory flow” (PTIF) Evaluation

#### Christophe Milesi (*speaker*)^1^, Stefan Matecki^2^, Anne Requirand^3^, Julien Baleine^1^, Aymeric Douillard^4^, Flora Habas^1^, Johan Moreau^5^, Gilles Cambonie^1^

##### ^1^Réanimation pédiatrique, Montpellier, FRANCE; ^2^Hopital Arnaud de Villeneuve - Physiologie pédiatrique, Montpellier, FRANCE; ^3^Physiologie pédiatrique, Montpellier, FRANCE; ^4^Département d’informatique médicale, Montpellier, FRANCE; ^5^Pneumologie pédiatrique, Montpellier, Montpellier

###### **Correspondence:** Christophe Milesi - c-milesi@chu-montpellier.fr

*Annals of Intensive Care* 2019, **9(Suppl 1)**:P-198

**Introduction**: PTIF in infants with bronchiolitis has never been determined. However this data is important to consider for the settings of HFNC because its efficiency is optimal when the settled flow is equal or higher than the patient’s PTIF.

**Patients and methods**: Prospective physiological observational study -Primary outcome: PTIF measurement in a population of mild bronchiolitis. -Secondary outcome: Correlation between PTIF and respiratory distress scores (Silverman score, modified Wood’s clinical asthma score, (m-WCAS) and respiratory rate (RR). Inclusion criteria: (1) Infants with bronchiolitis up to 6 months old, treated with HFNC, (2) Mild severity (2 < m-WCAS < 5), (3) No need for invasive or non-invasive ventilation (4) Signed parental consent. Study design: Patients were evaluated in the ICU of Montpellier. Spirometer was applied for the recording of 20 consecutives spontaneous respiratory cycles. PTIF, Tidal volume (TV) and RR were recorded. Clinical evaluation: respiratory distress scores rating (Silverman, m-WCAS).

**Results**: (mean (DS)) From November to February 2018, 33 patients, 1.6 (1.04) months old and 4487 (1280) g were evaluated. Spirometric measurements were: PTIF = 7.47 (3.4) l/min; PTIF indexed to the weight = 1.7 (0.8) l/min/kg, TV/kg = 5.9 (2.3) ml/kg. Pearson correlation between PTIF and weight was good (r = 0.7(0.47; 0.85) p < 0.001). There was no correlation between PTIF or PTIF/Kg and Silverman score (r = -0.05(-0.4; 0.3) p = 0.7), mWCAS (r = 0.28(-0.07; 0.5) p = 0.11) or RR (r = 0.28 (-0.007; 0.5) p = 0.11).

**Conclusion**: PTIF is independent from the clinical signs of respiratory distress. If HFNC is used in this situation, this study suggests to set the flow close to 2 l min kg whatever the severity.


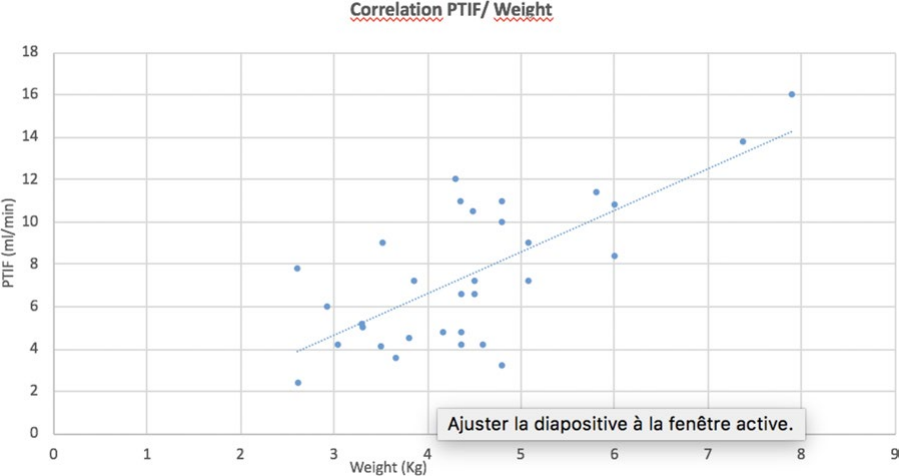


### P-199 Prone positioning effects on lung recruitment during pediatric refractory Acute Respiratory distress Syndrom with Extracorporeal membrane oxygenation

#### Pierre-Louis Leger (*speaker*), Isabelle Guellec, Alexandra Delarrard, Alain Amblard, Emilie Thueux, Marie Reymond, Jérôme Rambaud

##### APHP- PICU Trousseau, Paris, FRANCE

###### **Correspondence:** Pierre-Louis Leger - leger.pierrelouis@gmail.com

*Annals of Intensive Care* 2019, **9(Suppl 1)**:P-199

**Introduction**: Prone positioning (PP) is a strategy widely used in the management of severe adult and pediatric acute respiratory distress syndrome (p-ARDS). Refractory p-ARDS can benefit from extracorporeal respiratory membrane oxygenation (ECMO) but the PP is not proved in this situation. The objective of the study was to evaluate the effect of PP on lung recruitment in p-ARDS under ECMO, and also compare ECMO versus non-ECMO patients.

**Patients and methods**: This single-center observational study has been performed in the PICU at Trousseau Hospital from June 2016 to June 2018. A prone positioning protocol was used. The PP was prescribed by intensivists for duration 20 h 24 h. The lung recruitment was assessed by static compliance at H0 (supine position) and H4, H12 and H20 during PP.

**Results**: Twenty-five p-ARDS patients were included, 17 children were treated with conventional mechanical ventilation and 8 treated with ECMO associated with apneic ventilation. The mean age was 14 ± 26 months and the weight was 7.5 ± 6.8 kg. Before PP the mean FiO2 = 85 ± 19%, the mean arterial saturation was 94 ± 4%, the mean PaO2 FiO2 = 125 ± 65. The ventilatory parameters before ECMO were different between non-ECMO group and ECMO group- plateau pressure = 29 ± 7 vs 28 ± 4 cmH2O (p = 0.8341), PEEP = 8 ± 3 vs 14 ± 2 cmH2O (p = 0.0001), driving pressure = 20 ± 7 vs 14 ± 2 cmH2O (p = 0.02), tidal volume = 5.7 ± 0.8 vs 3.2 ± 1.8 ml Kg (p = 0.0001). The compliances increased in both groups from H0 to H12 (Table 1). At H20, the indexed pulmonary compliance was significantly lower in ECMO than non-ECMO patients.

**Discussion**: The patients presented moderate p-ARDS with PaO2 FiO2 = 125 and followed PP adult recommendations. In ECMO group, the driving pressure was significantly higher than non-ECMO group due to an apneic ventilation (high PEEP and limited plateau pressure). The PP might not be an effective strategy for p-ARDS with ECMO due to absence of maintained lung recruitment by low tidal volume ventilation. These results should be confirmed on larger cohorts.

**Conclusion**: The prone positioning in pediatric ECMO-ARDS could be insufficient to maintain the lung recruitment over a long time.


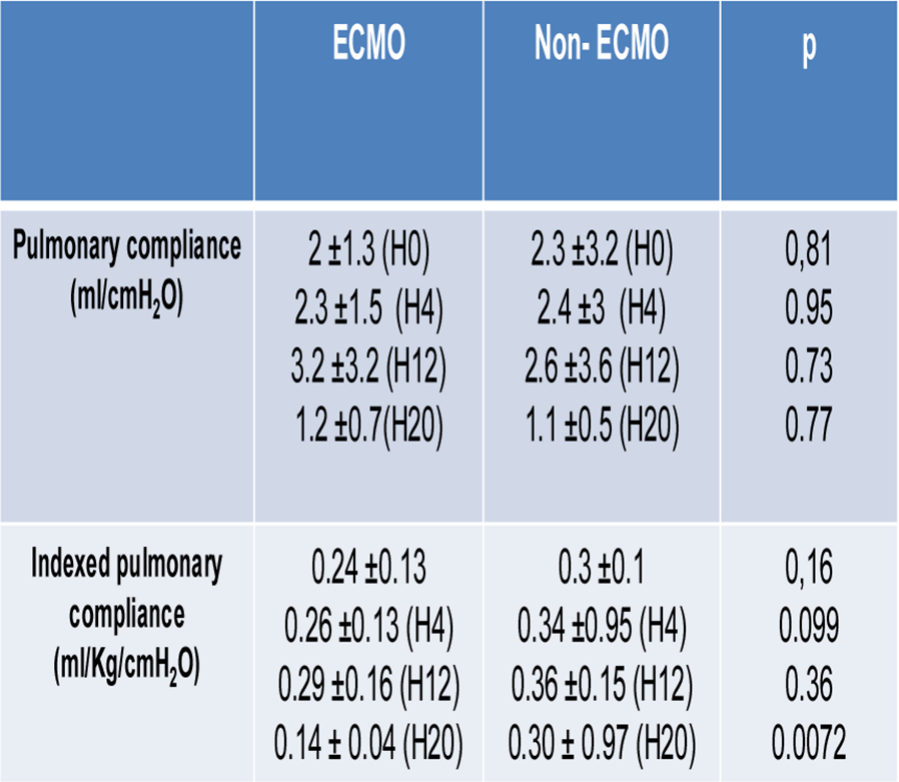


### P-200 Prevalence of multi-resistant bacterial carriage and incidence of related healthcare-associated infections in a French paediatric intensive care unit

#### Michael Levy (*speaker*), Jérôme Naudin, Marion Caseris, Patricia Mariani-Kurkdjian, Eric Thebault, Maryline Chomton, Stéphane Bonacorsi, Stéphane Dauger, Catherine Doit

##### Hôpital Robert-Debre, Paris, FRANCE

###### **Correspondence:** Michael Levy - michael.levy@aphp.fr

*Annals of Intensive Care* 2019, **9(Suppl 1)**:P-200

**Introduction**: Few data exist concerning multidrug-resistant (MDR) bacterial carriage and its impact on health-care associated infections (HCAI) in paediatric intensive care units (PICU). The objective of this study was to estimate the prevalence of MDR bacterial carriage in PICU and describe the epidemiology of related HCAI.

**Patients and methods**: We performed a monocentric prospective cohort study in our tertiary 20 bed-PICU from the 1st of January 2011 to the 31st of December 2016 in Paris, France.

**Results**: Among the 5,419 patients admitted during the study period, 550 patients (10.1%) were extended-spectrum ß-lactamase producing Enterobacteriaceae (ESBL-E) carriers and 99 patients (1.8%) were methicillin-resistant Staphylococcus aureus (MRSA) carriers (respectively 90.4% and 86% colonized on admission). The median prevalence rate per year of the 3 major HCAI was 3.1% (1.8% to 4.3%) with an incidence of 4.8 infections per 1000 patient-days (3.3 to 6.2). 146 HCAI occurred including 41.8% of ventilator-associated pneumonia (VAP), 39% of central line-associated bloodstream infection (CLABSI), and 19.2% of catheter-associated urinary tract infection (CAUTI). 11 were due to ESBL-E (7.5%) whereas 3 were due to MRSA (2.0%), all occurring in previously identified carriers. There was no difference in length of stay and mortality between HCAI due to ESBL-E versus HCAI not due to ESBL-E (respectively 21.5 days versus 20 days (p = 0.93) and 25% versus 23% (p = 1.00)).

**Conclusion**: Prevalence of acquired MDR bacterial carriage is low in our PICU and patients who develop HCAI with MRSA or ESBL-E have a previous carriage that can be identified on surveillance swabs. These findings enable a limited use of vancomycin or carbapenems in case of HCAI in patients with no know colonization.

### P-201 The use of automated pupillometry to assess cerebral autoregulation

#### Armin Quispe Cornejo (*speaker*), Ilaria Alice Crippa, Lorenzo Peluso, Lorenzo Calabró, Jean-Louis Vincent, Jacques Creteur, Fabio Silvio Taccone

##### Hôpital Erasme, Bruxelles, BELGIUM

###### **Correspondence:** Armin Quispe Cornejo - arminquispe@gmail.com

*Annals of Intensive Care* 2019, **9(Suppl 1)**:P-201

**Introduction**: Automated pupillometry (AP) can be used to quantify pupil light reflex (PLR) in critically ill patients. However, the complexity of sympathetic and parasympathetic pathways involved in the PLR may expand the use of AP to quantify other phenomena related to nervous system, such as the regulation of vascular tone and regional blood flow.

**Patients and methods**: Observational ongoing study including critically ill patients admitted to the Intensive Care Unit. Exclusion criteria were- ocular diseases + intracranial disease&#894 + arrhythmias&#894 + extracorporeal membrane oxygenation&#894 + supra-aortic arteriopathy. Quantitative pupillometry was performed using the NPi^®^-200 pupillometer (Neuroptics^®^), which calculates the Neurological Pupil Index (NPI), pupillary contraction, latency, constriction velocity and dilation velocity. The mean value of these variables measured on each eye was calculated. Transcranial Doppler (DWL, Germany) was performed insonating the left middle cerebral artery (LMCA) with a 2 MHz probe. LMCA blood flow velocity (FV) and arterial blood pressure (BP) signals were simultaneously recorded&#894 + Pearson´s correlation coefficient between BP and FV (Mxa) was calculated using MATLAB (MathWorks, USA). Impaired CAR was defined as Mxa > 0.3.

**Results**: We studied 35 patients (median age 61 [52–69] years and median APACHE II score on admission 16 [9–25]), including 20 (57%) with sepsis. Median NPI was 4.5 (4.1–4.8) and median Mxa 0.28 (0.13–0.50) + 14 (40%) patients had altered CAR. We observed a significant correlation between Mxa and NPI (r = -0.51 + p = 0.001) but not with other variables measured by the AP. NPI was significantly lower in patients with altered CAR than others (4.0 (3.6–4.6] vs. 4.6 [4.4–4.8] + p = 0.03). NPI values had an AUC of 0.72 [0.53–0.90] to predict altered CAR. A NPI < 4.0 had a sensitivity of 44% and a specificity of 95% to predict altered CAR.

**Conclusion**: The Neurological Pupil Index measured with automated pupillometry allows to assess cerebral autoregulation in critically ill patients.


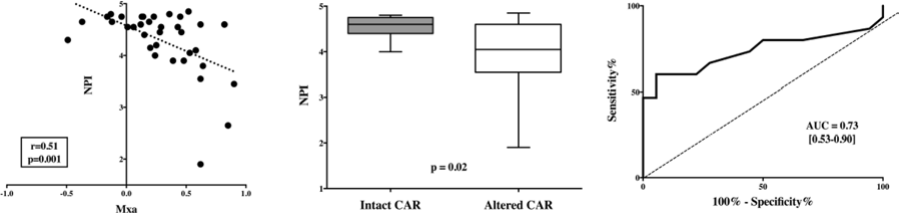


### P-202 Comparison of 2 Weaning Methods for External Ventricular Drainage (EVD)

#### Anne-Gaelle Si Larbi (*speaker*), David Cortier, Vincent Reina, Mathilde Phillips Houlbracq, Catherine Horodyckid, Guillaume Tachon, Charles Cerf

##### Hopital Foch, Suresnes, FRANCE

###### **Correspondence:** Anne-Gaelle Si Larbi - agsl17@club-internet.fr

*Annals of Intensive Care* 2019, **9(Suppl 1)**:P-202

**Introduction**: Most often, external ventricular drainage (EVD) is set up for a limited time. The question is to find the right time to remove it. Leaving an EVD longer than necessary exposes to complications, especially infectious ones. On the other hand, wrongly removing an EVD exposes to recurrence of hydrocephalus and the risk of set up a new EVD. There is no formal recommendation for the weaning of EVD. The objective of this study is to describe and compare the 2 weaning methods performed in our department.

**Patients and methods**: Between the first July 2011 and 30th June 2017, 367 EDV were placed in 285 patients and 161 EDV had at least one weaning test. There are two different methods performed in our department- a rapid weaning (RW) which consists of clamping the EVD and a gradual weaning (GW) that consists in gradually increasing the level of the EVD before clamping. A brain CT scan is performed before clamping and after 48 h of clamping, before removing of EVD. Quantitative values are expressed as mean (SD) and compared with Student test. Qualitative values are expressed as number of value (%) and compared with Chi2 test.

**Results**: 195 weaning tests were performed on 161 EVD, 61 (31%) were RW and 134 (69%) were GW. Description and comparison of patient and EVD characteristics by type of weaning is shown in the table. The 52 weaning failures were - 37 neurological symptoms (confusion or somnolence n = 21, headache n = 10, coma n = 4, nausea n = 1, aphasia n = 1), 5 elevated intracranial pressure (ICP), 7 ventricular dilatation on CT scan, 3 cerebrospinal fluid (CSF) related problems (2 hemorrhagic CSF, 1 CSF leak through the scar). After a first failure, the EVD was finally successfully removed in 26 (50%) cases after an average of 9.9 days.

**Conclusion**: The two weaning methods seem to be performed under the same conditions and within the same delays. The failure rate is similar but rapid weaning significantly reduces the duration of EVD by an average of 2.4 days.


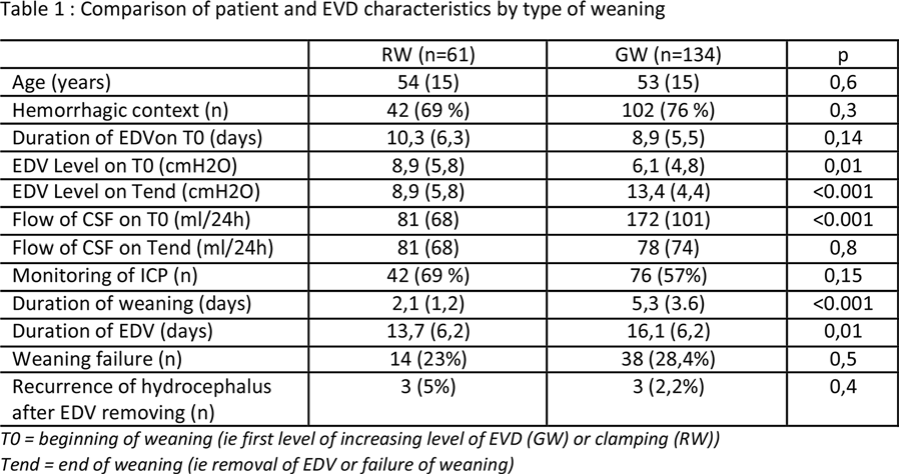


### P-203 Pneumonia in critically ill ischemic stroke patients-epidemiology and prognostic impact

#### Etienne De Montmollin (*speaker*)^1^, Stephane Ruckly^2^, François Philippart^3^, Carole Schwebel^4^, Daniel Da Silva^5^, Eric Mariotte^6^, Guillaume Marcotte ^7^, Yves Cohen^8^, Benjamin Sztrymf^9^, Fabrice Bruneel^10^,Marc Gainnier^11^, Shidasp Siami^12^, Romain Sonneville^13^, Jean François Timsit^13^

##### ^1^Université Paris Diderot, IAME, UMR 1137, Sorbonne Paris Cité, Paris, FRANCE; ^2^ICU REsearch, Département de biostatistiques, Paris, FRANCE; ^3^Service de Médecine Intensive et Réanimation, Groupe Hospitalier Paris Saint-Joseph, Paris, FRANCE; ^4^Service de Réanimation Médicale, Hôpital Albert Michallon, La Tronche, FRANCE; ^5^Service de Médecine Intensive et Réanimation, Hôpital Delafontaine, Saint-Denis, FRANCE; ^6^Service de Réanimation Médicale, Hôpital Saint-Louis, AP-HP, Paris, FRANCE; ^7^Département d’anesthésie-Réanimation, Hôpital Édouard Herriot, Lyon, FRANCE; ^8^Service de Réanimation Médico-Chirurgicale, Hôpital Avicenne, AP-HP, Bobigny, FRANCE; ^9^Service de Réanimation, Hôpital Antoine Béclère, AP-HP, Clamart, FRANCE; ^10^Service de Réanimation Médico-Chirurgicale, Hôpital André Mignot, Le Chesnay, FRANCE; ^11^Service de Réanimation, Hôpital de La Timone, AP-HM, Marseille, FRANCE; ^12^Service de Réanimation, Hôpital de La Timone, AP-HM, Etampes, FRANCE; ^13^Service de Réanimation Médicale, Hôpital Bichat Claude Bernard, AP-HP, Paris, FRANCE

###### **Correspondence:** Etienne De Montmollin - etienne.demontmollin@aphp.fr

*Annals of Intensive Care* 2019, **9(Suppl 1)**:P-203

**Introduction**: Infections are a frequent complication after stroke. Pneumonia is the most frequent site of infection and is associated with lower survival and impaired neurologic outcome. Studies focusing on intensive care patients show the highest incidence of pneumonia, but are very heterogeneous in the severity of selected patients. To date, there is no study focusing exclusively on the epidemiology and prognostic impact of pneumonia in mechanically ventilated stroke patients. Furthermore, previous studies lack the adjustment for early decisions to forgo life-sustaining treatment (DFLST) which is a significant confounder in these patients.

**Patients and methods**: We conducted a retrospective analysis of a large prospective multicenter database over a 20-year period (1997–2016). We included all adult ischemic stroke patients admitted to the ICU who required invasive mechanical ventilation at ICU admission. The relation between the occurrence of pneumonia during ICU stay and 30-day mortality was investigated using a Cox proportional hazard model, adjusted on DFLST. Data are presented as median (interquartile range) or numbers (percentages).

**Results**: We identified 195 patients (age 69 (61–76) years, male gender 132 (67.7%) patients) from 11 ICUs. On ICU admission, the Glasgow coma scale score (GCS) was 6 (3-, 0) and the Simplified Acute Physiology score 2 (SAPS 2) score was 56 (45–66). Patients required vasopressors in 92 (47.2%) cases and renal replacement therapy in 17 (8.7%) cases. DFLST occurred 5 (2–8) days after ICU admission in 65 (33.3%) patients. Withholding of care and withdrawal of care were observed in 31 (15.9%) and 34 (17.4%) cases, respectively. Survival at day 3six, 6 months and one year was 44%, 32, 6% and 29.7% respectively. During ICU stay, there were 90 pneumonia episodes, occurring at least once in 74 (37.9%) patients. Pneumonia caused sepsis and septic shock (SEPSIS-3 definition) in 40 (44.4%) and 33 (36.7%) cases respectively. After adjustment, the occurrence of pneumonia during ICU stay was not associated with 30-day mortality (figure 1).

**Conclusion**: In this cohort of mechanically ventilated patients with acute ischemic stroke, pneumonia was a frequent complication but was not associated with 30-day mortality. Impact of pneumonia on functional outcomes in survivors should be investigated in further studies.


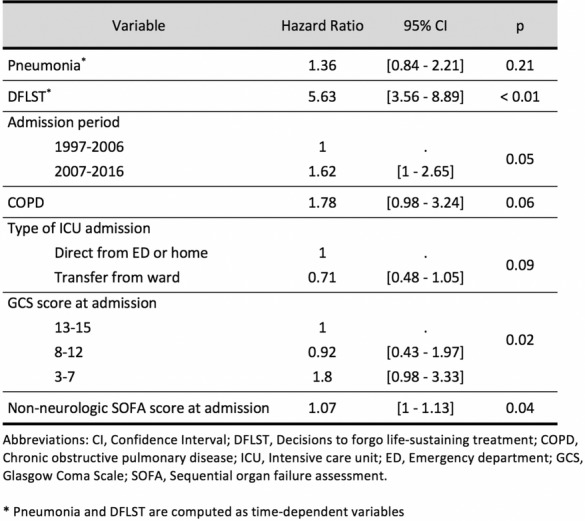


### P-204 Pilot study of cerebrovascular accidents at Sidi-Bel-Abbès University Hospital: which risk factors? Assumption of responsibility and proposal to be adopted

#### Samia Benouaz (*speaker*)^1^, Setti Zelmat^2^, Faiza Nadia Benatta^2^, Djahida Djamila Batouche^2^

##### ^1^Faculté de médecine, Sidi-Bel-Abbes, ALGERIA; ^2^Faculte de medecine, Oran, ALGERIA

###### **Correspondence:** Samia Benouaz - dr_benouaz@hotmail.fr

*Annals of Intensive Care* 2019, **9(Suppl 1)**:P-204

**Introduction**: Stroke is the most common neurological condition. Management has progressed in recent years, but morbidity and mortality remain high. Stroke is the second leading cause of death in the world. in developing countries, behind cardiovascular diseases. According to the 2015 estimate- 60,000 new cases registered each year in Algeria and 20,000 deaths. First cause of acquired mental and physical disability. The objective- To analyze the risk factors related to stroke and to evaluate its management.

**Patients and methods**: Retrospective study covering a period of 16 months (October 2016 to January 2018). Included were all patients with acute neurologic impairment admitted to the emergency department. Evaluation criteria- NIHSS score, Glasgow score, blood pressure, reason for orientation.

**Results**: We collected 430 files of patients admitted for acute neurological deficit, 86% of strokes were ischemic. Patients admitted to intensive care- 82 430.We note a predominance of male 279 430 and an average age of 62.The patients arrived several hours after the onset of symptoms. The NIHSS score > 25 (7%), between 5–25 (77%), NIHSS < 5 (14%). The analysis of risk factors shows- HTA in 277 430 on ARA II, diabetes 246 430, atrial fibrillation arrhythmia in 321 430 cases, dyslipidemia 398 430 and cardiovascular history in 378 430 cases. The relationship between HTA and the NIHSS score- presence of arterial hypertension- NIHSS < 5 (68%), NIHSS 5–25 (62%) and NIHSS > 25 (70%), atrial fibrillation arrhythmia relationship and NIHSS score- presence atrial fibrillation arrhythmia- 12% for NIHSS score < 5, 8% for NIHSS 5–25 and 40% for NIHSS score > 25.

**Discussion**: The frequency and risk factors for the occurrence of stroke are similar to those found in the literature. However, the management is not optimal.

**Conclusion**: Stroke is a major public health problem in our country. Our study shows that hypertension is the most frequently associated risk factor, which needs to be strengthened with screening and treatment. The number of patients admitted to intensive care can be reduced thanks to the creation of neurovascular unit and the control of risk factors. Summary- To improve the management of patients with stroke, an assessment was needed. This evaluation has shown the relationship between risk factors, namely hypertension and atrial fibrillation arrhythmia, which must be controlled.

### P-205 Prognostic value of troponin in the acute phase of ischemic stroke

#### Mohamed Walid Mhajba (*speaker*)^1^, Mohamed Ali Cherif^2^

##### ^1^Centre d’Assistance Médicale Urgente (CAMU), Tunis, TUNISIA; ^2^Service des urgences et rénimation CHU Hbib Thameur, Tunis, TUNISIA

###### **Correspondence:** Mohamed Walid Mhajba - mhajbawalid@hotmail.fr

*Annals of Intensive Care* 2019, **9(Suppl 1)**:P-205

**Introduction**: Stroke is an important cause of morbidity and mortality. Several independent factors influence the overall survival. The prognostic value of cardiac troponin and electrocardiographic signs are poorly studied. The aim of this work was to evaluate the frequency of troponin elevation in the acute phase of stroke and its association with electrocardiographic signs and mortality.

**Patients and methods**: Records for ischemic stroke were collected between July 2016 and March 2017 amongst the patients. Patients who received troponin were included. Clinical and para-clinical data were also collected. The association between elevation of troponin (> 0.09 ng mL) along with the presence of electrical signs of ischemia and mortality were studied.

**Results**: 283 patients were included with mean age of 69 ± 12 years and sex ratio 1.02. Comorbidities were dominated by high blood pressure (69%) followed by diabetes (46%). The concept of coronary insufficiency was known in 51 patients (18%). Troponin was positive for 42 patients (14.8%), 73% of whom did not have a known history of coronary artery disease. The diagnosis of Troponin-positive NSTEMI associated with stroke was retained in three patients. Half of the patients with a positive troponin had consistent ECG changes with myocardial ischemia (p < 0.001, OR = 3.63, 95% CI- 1.84–7.16). The electrical signs were mainly subepicardial ischemia (52%) followed by the subendocardial lesion (35%). ACFA was present for 69 patients and it was not associated with a positive troponin (p = 0.767) and the same goes to the occurrence of haemorrhagic transformation (p = 0.713). The overall mortality was 13.4% and 34% of the deceased patients had a positive troponin (p < 0.001).

**Conclusion**: In the acute phase of stroke, the elevation of troponin or the occurrence of electrocardiographic changes must be part of a comprehensive diagnostic approach as well as an approach to the prevention and management of comorbidities.

### P-206 Decompressive Craniectomy for Ischemic Stroke- Morbidity and Outcomes

#### Rania Ammar (*speaker*), Smaoui Mariem, Kolsi Fatma, Hammami Maha, Chtara Kamilia, Turki Olfa, Ben Hamida Chokri, Bahloul Mabrouk, Chelly Hedi, Bouaziz Mounir

##### University of sfax, Sfax, TUNISIA

###### **Correspondence:** Rania Ammar - rania.ammarzayani@gmail.com

*Annals of Intensive Care* 2019, **9(Suppl 1)**:P-206

**Introduction**: Malignant cerebral edema following ischemic stroke is life threatening. It can cause inadequate blood flow and perfusion and increase intracranial pressure and brain shift leading to herniation syndrome then to brain death. Multiple randomized clinical trials have shown that decompressive hemicraniectomy (DhC) effectively reduces mortality and morbidity. Objective of our study is to evaluate outcomes (mortality and morbidity) of patients with malignant ischemic stroke who underwent DhC.

**Patients and methods**: We retrospectively collected data of malignant Ischemic Stroke of patients requiring DhC from April 2015 to July 2018. Clinical outcomes were assessed using the modified Rankin scale (mRS).

**Results**: Fourteen patients were included, Mean age was 57, 36 years (SD ± 12.43 years), 5 patients were older than 60 years (35.71%), sex ratio was 1.33. SAPSII at admission averaged 40.29 (SD ± 13.59) points. Median of Glasgow coma scale was 8. Seven strokes (50%) occurred in the left middle cerebral artery (MCA) territory, while 5 (35, 72%) occurred in the right MCA territory, one stroke (7, 14%) occurred in posterior cerebral artery territory and one stroke (7, 14%) occurred in anterior cerebral artery territory. The mean time of decompressive hemicraniectomy was 2.5 days. The median modified Rankin scale (mRS) at 3 month was 5.5. Mortality was 50%.

**Conclusion**: Although DCH is considered a live-saving procedure and decrease mortality, but many patients suffering a worse functional outcome and disability.

### P-207 Early outcome predictors of traumatic brain injury

#### Mariem Dlela (*speaker*), Amal Triki, Olfa Turki, Sabrine Bradaii, Mabrouk Bahloul, Chokri Ben Hamida, Mounir Bouaziz

##### Hbib bourguiba university hospital, Sfax, TUNISIA

###### **Correspondence:** Mariem Dlela - mariem241090@gmail.com

*Annals of Intensive Care* 2019, **9(Suppl 1)**:P-207

**Introduction**: Brain-injury is a leading factor of mortality and morbidity from traumatic injury in young and previously healthy patients. Early evaluation of the prognosis of a patient is necessary to determine the most suitable medical care strategy. The aim of this study was to predict mortality and outcome in brain injured population based on the first clinical and biological evaluation.

**Patients and methods**: We conducted an eight month long prospective cohort, including all patients admitted to a university hospital ICU with moderate to severe traumatic brain injury (TBI), defined as a Glasgow coma scale below twelve (GCS < 12). Outcome was evaluated by incidence of death, and Glasgow outcome scale (GOS) on day thirty. Both univariate and multivariate analysis were used to determine level of significance.

**Results**: During the study period, 118 trauma patients were admitted to our ICU, 83presented with moderate to severe TBI and were included. There were 69 males (83.1%) and 14 females (16.9%) patients. The mean age was at 32.06 ± 18.07 and mean injury severity score (ISS) was 28.96 ± 10.16. According to our analysis mortality rate among patients sustaining moderate to severe TBI was 15.7%. Average ICU LOS was 14.6 days and average ventilator days was 9.68 days. Using the multivariable logistic regression model, only initial glucose serum levels (p = 0.002) and pupil response (p- 0.004) were found to be independent risk factors of mortality. Linear regression models also identified glucose levels as predictive of GOS on day 30 (p = 0.019).

**Conclusion**: We conclude that initial glucose level is an independent predictor of mortality and morbidity in brain injured patients. Hyperglycemia could be considered as a simple yet valuable marker ofbrain injury and, when present upon admission, could reflect extensivebrain damage, frequently associated with mortality and bad outcome.

### P-208 Translation and validation of a Tunisian version of the Confusion assessment method for the intensive care unit (CAM-ICU)

#### Saïd Kortli (*speaker*)^1^, Imen Ben Saida^1^, Kacem Nawres^1^, Hend Zorgati^1,^ Mohamed Boujelben^1^, Waffa Zarrougui^1^, Nesrine Fraj^1^, Meriem Ghardallou^2^, Badii Amamou^3^, Mohamed Boussarsar^1^

##### ^1^Farhat Hached University Hospital, Medical Intensive Care Unit, Sousse, TUNISIA; ^2^Department of Preventive Medicine, Faculty of Medicine Sousse, Sousse, TUNISIA; ^3^Fattouma Bourguiba University Hospital, Department of Psychiatry, Monastir, TUNISIA

###### **Correspondence:** Saïd Kortli - kortlisaiid@gmail.com

*Annals of Intensive Care* 2019, **9(Suppl 1)**:P-208

**Introduction**: Delirium is an acute brain dysfunction commonly seen in critically ill patients and associated with poor outcomes. Delirium is usually underdiagnosed by physicians due to the lack of adequate screening tool. The Confusion Assessment Method for the Intensive Care Unit (CAM-ICU) is one of the most widely used screening methods for detection of ICU delirium. It has been translated into over 25 languages. This scale, however, has not been translated and validated into Tunisian Arabic dialect language. The aim of this study was to translate, retranslate and validate a Tunisian version of the CAM-ICU.

**Patients and methods**: After permission from Ely et al., the Forward and backward translation of the Tunisian version of the CAM-ICU was performed according to the protocol of the “MAPI Research Institute which includes conceptual analysis, forward translation by two translators, consensus between the two versions, backward translation, comparison between the source and the backward translation, pilot testing to obtain a final translated version and validation. For validation and interrater reliability assessment of the Tunisian CAM-ICU, two intensivists independently assessed delirium in ICU patients and the results were compared with the reference evaluation, which was done by a psychiatrist using the Diagnostic and Statistical Manual of Mental Disorders V (DSM-V). Interrater reliability was calculated using kappa statistics.

**Results**: During the study period between October 2017 and June 2018, 137 patients were evaluated by two intensivists and one psychiatrist expert independently. **Based on DSM-V criteria, 46 out of 137 (33.6%) patients developed delirium. According to the Tunisian CAM-ICU, the frequency of delirium was consecutively 38 of 137 (27.7%) in the first evaluation by the intensivist 1 and 45 of 137 (32.6%) by the second intensivist. The Tunisian CAM-ICU was done with acceptable interrater reliability between intensivist 1 and intensivist 2 in terms of assessing delirium (Kappa = 0.844, p < 0.001). The sensitivities of the two intensivists’ evaluations using the Tunisian CAM-ICU were 80.4% for intensivist 1 and 95.7% for intensivist 2. Their specificities were 98.9% and 98.9% respectively.

**Conclusion**: The Tunisian version of the CAM-ICU showed good validity and reliability to detect delirium in critically ill patient. It could therefore be appliable in Tunisian ICUs after appropriate training.

### P-209 Accuracy of pulse oximetry in the intensive care unit - a prospective study

#### Myriam Kallel (*speaker*), Amira Jamoussi, Samia Ayed, Fatma Jarraya, Dhouha Lakhdher, Jalila Ben Khelil, Mohamed Besbes

##### Medical Intensive Care Unit, Abderrahmen Mami pneumology hospital, Ariana, TUNISIA

###### **Correspondence:** Myriam Kallel - myriamkallel1991@gmail.com

*Annals of Intensive Care* 2019, **9(Suppl 1)**:P-209

**Introduction**: Pulse oximetry is a non-invasive method for monitoring the oxygen saturation of critically ill patients. Few studies have investigated its accuracy in Intensive Care Units (ICU). We aimed to determine the difference between pulse oximeter oxygen saturation (SpO2) and arterial oxygen saturation (SaO2) for the critically ill patients and to assess the influence of specific physiologic factors on SpO2 accuracy.

**Patients and methods**: We conducted a prospective study between July 1st, 2018 and September 15, 2018. Inclusion criteria were simultaneous arterial blood gas (ABG) and SpO2 value records. For each pair ABG-SpO2, we collected concomitant haemoglobin, temperature, lactatemia and the requirement for vasoactive drugs. The bias calculated as the difference (SpO2-SaO2) was considered ‘acceptable’ if it ranged from -2% to +2% + and non-acceptable if not + then we analysed it according to concomitant conditions.

**Results**: During the study period, we collected 81 ABG-SpO2 pairs. The mean bias was: 1.106% ± 2.435% [−9.30–4.20]. The bias was acceptable in 42 pairs (52%) and non-acceptable in the 39 others (48%). We compared subgroups of patients according to biases acceptability as shown in table 1.

**Conclusion**: The bias (SpO2-SaO2) in critically ill patient is high. SpO2 accuracy was significantly impaired when Hemoglobin was 10 g/dl. Nevertheless, it was independent of hemodynamic and respiratory status.

### P-210 Management of respiratory failure in pregnant and post-partum patient

#### Dhouha Lakhdhar (*speaker*), Fatma Jarraya, Myriam Kallel, Amira Jamoussi, Samia Ayed, Jalila Ben Khelil, Mohamed Besbes

##### Medical Intensive Care Unit, Abderrahmen Mami pneumology hospital, Ariana, TUNISIA

###### **Correspondence:** Dhouha Lakhdhar - lakdardoha@gmail.com

*Annals of Intensive Care* 2019, **9(Suppl 1)**:P-210

**Introduction**: Respiratory failure during pregnancy and post-partum period is an uncommon but particular situation. There is a lack of literature concerning the assessment and management of these patients, the choice of investigations and treatments may affect the mother and the fetus.**The aim of this study was to study characteristics and outcome of these patients admitted in ICU.**.

**Patients and methods**: This was a mono centric, descriptive and retrospective study. The datas of pregnant and post-partum patients admitted in ICU from January 2009 to August 2018 were reviewed. We collected informations regarding clinical presentation, treatment and outcome.

**Results**: Forty patients were included in the study, account for 0.96% of our ICU admissions. Twenty-five were pregnant and 15 were in the post-partum period. The median age was 32 years old [27 − 37]. Median severity scores were 7 [3 − 15] for APACHE II and 15.5 [10 − 37] for SAPS II. Median gestational age was 24 weeks [20 − 29]. Thirtythree patients had Acute Respiratory Failure at the time of admission, the rest developped it later during their stay.The leading cause of respiratory failure was pulmonary infection 42.4% (n = 14), 11 patients were diagnosed with viral pneumonia (78.6%), 9 patients with asthma (27.3%), 4 patients with pulmonary embolism (12.2%) and 6 patients with pulmonary edema (18.2%).Six patients had a septic shock at the time of the admission, seven had a severe ARDS. H1N1 was isolated in eleven patients. Five women had CPAP among them four survived and three lost their babies. Eleven were ventilated with non invasive ventilation (NIV), nine women survived and three lost their babies + 19 were under invasive ventilation (IV), among them five deceased. Seven patients had prone positioning (PP) with three mothers and three babies survived, among them two were delivered by cesarian.. Invasive ventilation (p = 0.002), sedation (p = 0.004) and curarisation (p = 0.002) acute kidney failure (p = 0.003) and a prolonged hospitalization in ICU (p = 0.004) did significantly increase the mortality of both pregnant and patients in the post-partum period. The median duration stay in ICU was 5.5 days [3 − 9.75]. Most of the patients 87% (n = 35) left the ICU without major dysfunction.

**Discussion**

**Conclusion**: NIV should be tried when it’s possible even in mild ADRS or asthma, IV and sedation should be avoided if possible. PP can be considered in severe ARDS.

### P-211 Cognitive impairment in affected tunisians individuals chronic broncho-pneumopathy obstruction

#### Fatma Bouhaouala (*speaker*), Houda Snène, Salma Chérif , Sonia Toujani, Nozha Ben Salah, Nadia Mehiri, Béchir Louzir

##### Université de Tunis El Manar, Faculté de médecine de Tunis, Tunis, TUNISIA

###### **Correspondence:** Fatma Bouhaouala - bouhaouala_fatma@yahoo.fr

*Annals of Intensive Care* 2019, **9(Suppl 1)**:P-211

**Introduction**: Chronic obstructive pulmonary disease (COPD) is one of the leading causes of morbidity and mortality among chronic diseases. Cognitive dysfunction exacerbates the prognosis by affecting adherence and quality of life. The objectives of our work were to evaluate the cognitive function in the patients followed for COPD, to determine the most affected areas as well as the predictive factors of this attack.

**Patients and methods**: Transversal study conducted at the outpatient clinic of the pneumology departments of La Rabta and Mongi Slim La Marsa. All patients had respiratory functional investigations and the severity of COPD was assessed by the GOLD 2014 classification and the BODE index. Cognitive function was assessed using the Mini Mental State Examination (MMSE) questionnaire translated into dialectal Arabic.

**Results**: One hundred and fifty patients were collected (37% illiterate), with a mean age of 66 years. Mean smoking was 60 ± 33.9 PY, 33.3% patients had cardiovascular comorbidities and 40.7% were frequent exacerbators. Respiratory functional investigations showed- mean CVF at 62.9%, mean FEV1 at 47.5%, mean PaO2 at 72.8 mmHg (less than 55 mmHg = 6.7%), and mean PaCO2 at 42.6 mmHg (greater than 45 mmHg = 32.4% of cases). Fifty-four percent of the patients were GOLD D and the average BODE index was four. Cognitive dysfunction was found in 10.7% of cases (13.3%, among the educated and 5.8% among the illiterate). This impairment of cognitive function was correlated with- age (p < 0.001), number of years of study (p < 0.001), frequency of exacerbations (p = 0.01), lower PaO2 at 55 mmHg (p = 0.05), PaCO2 greater than 45 mmHg (p = 0.01) and BODE index (p = 0.02). The multivariate linear regression study found a correlation with the number of years of study (p < 0.001), rhythm disorders (p = 0.04) and the PaO2 at 55 mmHg (p = 0.017).

**Conclusion**: The rate of cognitive impairment in our population, assessed by the MMSE questionnaire, is lower than that reported in the literature and this is due, among other things, to the difference in the methodology and level of schooling of the selected population. The number of years of study, the rhythm disorders and the frequency of the exacerbations are the factors significantly correlated with the cognitive impairment.

### P-212 Prospective observational study on the association between serum mannose-binding lectin levels and severe outcome in critically ill patients with pandemic influenza type A (H1N1) infection

#### Elie Zogheib (*speaker*)^1^, Remy Remy^1^, Taieb Chouaki^2^, Boualem Sendid^2^, Julien Monconduit^1^, Vincent Jounieaux^1^, Christine Segard^3^, Julien Maizel^1^,Hervé Dupont^1^

##### ^1^Réanimation médicale - CHU Amiens, Amiens, France; ^2^Service de mycologie et parasitologie - CHU Amiens, Amiens, France; ^3^Service de virologie - CHU Amiens, Amiens, France

###### **Correspondence:** Elie Zogheib - eliezogheib1@yahoo.fr

*Annals of Intensive Care* 2019, **9(Suppl 1)**:P-212

**Introduction**: Mannose-Binding Lectin (MBL) plays an important role in the innate immune response. Our aim was to determine whether baseline serum levels of MBL at admission to ICU could predict mortality in ICU patients with pdmH1N1 infection.

**Patients and methods**: Prospective observational study performed in ICU patients with ARDS due to A (H1N1) pdm09 virus. Demographic characteristics and severity indices were recorded at ICU admission. MBL was assayed from blood drawn at H1N1 diagnosis. Outcomes were compared according to MBL levels. Results are expressed as median and interquartile range.

**Results**: MBL levels were studied in 27 patients (median age- 56 [29] years) with severe pdmH1N1 infection. Median admission SAPS II and SOFA scores were 49 [26] and 12 [5], respectively. Thirty-day mortality rate was 37% (n = 10 27). MBL was significantly higher in non-survivors (3741 ng ml [2336]) vs survivors (215 ng ml [1307]), p = 0.005. MBL cut-off > 1870 ng ml had a sensitivity of 80% and a specificity of 88.2% for mortality (AUC = 0.82 (95%CI = 0.63–0.94)). Kaplan–Meier analysis demonstrated a strong association between MBL levels and mortality (logrank 7.8, p = 0.005). MBL > 1870 ng ml was independently associated with mortality (adjusted HR = 8.7, 95%CI = 1.2–29.1, p = 0.007).

**Conclusion**: This study shows that baseline MBL > 1870 ng ml is associated with higher mortality in ICU patients with severe pdmH1N1 infection.

### P-213 Automatic oxygen weaning of patients following mechanical ventilation admitted to a Tunisian ICU

#### Islem Ouanes (*speaker*)^1^, Fatma Bouahaouala^1^, Syrine Maatouk^1^, Nouha Bouker^1^, Meriem Tlili^1^, Wiem Nouira^1^, Feriel Ben Aba^1^, Yosra El Ouaer^1^,Zeineb Hammouda^1^, Fahmi Dachraoui^1^, Lamia Ouanes-Besbes^1^,Erwan L’Her ^2^,Fekri Abroug^1^

##### ^1^CHU Fattouma Bourguiba, Université de Monastir, Monastir, TUNISIA; ^2^LATIM INSERM UMR 1101, Université de Bretagne Occidentale, Brest, France

###### **Correspondence:** Islem Ouanes - ouanes.islem@gmail.com

*Annals of Intensive Care* 2019, **9(Suppl 1)**:P-213

**Introduction**: Recently it has been demonstrated that conservative protocol for oxygen (O2) therapy resulted in lower ICU mortality than conventional therapy. FreeO2 device, automatically adjusts oxygen flow based on patients’ needs (SaO2), in the emergency department it was associated with improved oxygenation and adherence to guidelines. The aim of this study was to evaluate the feasibility FreeO2 in weaning of oxygen following mechanical ventilation (MV) in patients admitted in a Tunisian ICU.

**Patients and methods**: In a prospective cohort study including a cohort of patients admitted to the ICU (between June and September 2018), and requiring MV (NIV or intubation), we recorded Free O2 generated curves during 2 h when acute clinical condition was controlled. We assessed, O2 flow rate and the percentage of patients less than 1 l min at the end of the record, O2 consumption variation, the time in the SpO2 target, the time with severe desaturation (SpO2 < 85%), and the time with hyperoxia (SpO2 > 5% above the target). Statistics- dichotomic parameters are expressed in percentage whereas continuous variables are expressed in median and 25th-75th percentile interquartile ranges, linked parameters were assessed with Wilcoxon test.

**Results**: Twenty six records were collected in 18 patients. Median age was 62 years (54–80), 61.5% were male. 53.8% with COPD, 46.2% with Hypertension and 7.7% with diabetes. The first ventilator modality at admission was NIV in 57.7%, the main reason for ICU admission was acute respiratory failure in 76.9% (11.5% de novo, 65.4% on chronic). Free O2 records were made on day 8 (4–23) after ICU admission for a median duration of interpretable records was 1.64 h (1.9–1.97). Mean SpO2 target set was 93% (92–94). Table I summarizes main findings after Free O2 records. In 73.1% of records we observed a decrease in O2 flow and median reduction of flow was 109.8 L of O2 per hour (51.6–205.2). At the end of Free O2 record we noted that the median O2 flow 2.2 (1–4.5), and an O2 flow **≤** 1 l min was noted in 10 records (38.5%).

**Conclusion**: In our cohort automatic O2 titration with Free O2 was associated with substantial reduction in O2 delivery during record in patients following mechanical ventilation (MV). Further studies are needed to assess its impact on patient’s outcomes and cost-effectiveness.

### P-214 Weaning of mechanical ventilation in ICU burns

#### Kaouthar Faleh (*speaker*), Hana Fredj , Mehdi Somai , Imène Rejeb , Achraf Laajili , Najla Benslimen , Yasmine Garbaa , Imen Rahmani ,Amel Mokline, Amen Messadi

##### Service de réanimation des brûlés au centre de traumatologie et grands brulés Ben Arous, Tunis, TUNISIA

###### **Correspondence:** Kaouthar Faleh - kaouther_1@yahoo.fr

*Annals of Intensive Care* 2019, **9(Suppl 1)**:P-214

**Introduction**: Withdrawal of mechanical ventilation (MV) is a crucial moment of ICU stay and may influence patient outcome. Fail extubation is associated with high mortality rate ranging around 25 to 50%[1]. Adoption weaning protocols can prevent extubation failure. We aim to review our practice in weaning of MV in ICU burns.

**Patients and methods**: A retrospective study was conducted in burn center in Tunis from April 2017 to December 2017. Were included mechanically ventilated patients who have undergone weaning trial. Were excluded early died patients (< 24 h). We categorized ventilated patients into three groups according to the difficulty of weaning process [2]. Simple weaning includes patients who succeed the first weaning trial; Difficult weaning - patient who failed the first weanig trial and required up to 3 trials or 7 days to achieve success weaning. Prolonged weaning - patients required more than 7 days of weaning after the first weaning trial. Weaning criteria were- SpO2 > 90% and FIO2 40%, PEEP 5 cmH2O, respiratory rate/tidal volume < 105, no myocardial ischemia, no need or minimal vasopressor, hemoglobin > 10 g/dL. Demographic, clinical and outcomes data of patients were analysed.

**Results**: During the period of study, 240 patients were admitted. 63 patients were intubated (26%) and 43 were included. The mean age was 32 ± 11 years with a sex-ratio of 3.21. The average TBSA was 39 ± 20% including face and neck in all patients. The average duration of sedation was 9 ± 6 days. In our study, 10 patients (23%) had a simple weaning trial, 8 (18%) had a difficult weaning trial and 2 (4%) had prolonged weaning trial. Weaning from MV was successful in 47% of cas and failed in 53% of cases. Causes of extubation’s failure were esentially nosocomial pneumonia (n = 10) and ICU acquired weakness (n = 8). In our study, the total body burn surface area was associated with extubation failure - TBSA in successful weaning group was 30 ± 15% vs 48 ± 20% in failed weaning group (p = 0.003).

**Conclusion**: Our results suggest that the incidence of extubation failure was high «53%» in our patients due essentially to extented burns (48%) including neck and face region.

**References**

1. Esteban A and all. Am J Respir Crit Care Med 1997 + 156-459-465
2. Vallverdu I and all. Am J Respir Crit Care Med 1998 + 158-1855-1862

### P-215 Acute dyspnea in hospitalized patients in observation units- epidemiology and prognostic factors

#### Ines Sedghiani (*speaker*), Oussama Hergli, Dhekra Hosni , Youssef Zied Elhechmi, Mohamed Mezghani , Imen Zaghdoudi , Zouheir Jerbi

##### Hôpital Habib Thameur, Tunis, TUNISIA

###### **Correspondence:** Ines Sedghiani - sedghiani.ines@gmail.com

*Annals of Intensive Care* 2019, **9(Suppl 1)**:P-215

**Introduction**: Acute dyspnea is a common symptom encountered in hospitalized patients in emergency room with a clinical presentation sometimes atypical especially in the elderly. It has a wide variety of etiologies that can sometimes be life threatening. Our work aimed to establish the epidemiological and etiological profile of the patients admitted in emergency room and to identify its prognostic factors.

**Patients and methods**: This was a 16-month retrospective study including patients hospitalized in observation unit for acute dyspnea, which was defined as a breathing discomfort that had developed or worsened for less than two weeks. Were excluded all the patients who were admitted immediately in resuscitation unit and those suffering from post-traumatic dyspnea.

**Results**: We included 265 patients hospitalized for acute dyspnea which accounted for 26% of admissions. The median age of the patients was of 72 years. Acute respiratory failure was the main clinical presentation in our patients, which was hypoxemic in 60% of cases and hypercapnic in 37% of cases. Dyspnea was multifactorial in 15% of patients and the main etiologies were acute community-acquired pneumonia (42.6%), acute heart failure (36%) and decompensation of chronic obstructive pulmonary disease (COPD) (10%).

Non-invasive ventilation and invasive ventilation were respectively necessary in 44% and 2.2% of cases. The intra-hospital mortality was 18.5%. It highly increased in patients aged more than 70 years, alteration of consciousness, hemodynamic instability, IGS II score&#8805 + 29, in case of acidemia (pH7.25) or hypoxemia (PaO2 FiO2240).

The use of mechanical ventilation as well as the need of vasoactive drugs were also associated with higher mortality rates. Predictors of ventilatory support were polypnea > 25 cycles minute, COPD, signs of respiratory distress and blood pressure > 140 70 mm Hg. COPD was the only predictor of 3-month readmission in patients with acute dyspnea, whereas higher age of the patients was correlated with a longer hospital stay.

**Conclusion**: Acute dyspnea is one of the leading causes of emergency hospitalization of elderly patients. It’s mainly caused by the decompensation of respiratory and cardiac comorbidities. Age is the main prognostic factor.

### P-216 Mechanical ventilation weaning- conditions and procedures

#### Asma Mehdi (*speaker*), Ines Fathallah, Eya Seghir, Sahar Hbecha , Ameni Sghaier , Ghada Sbouii , Khawla Ben Ismail , Hayfa Fezzeni

##### Yasminette Hospital IUC, Ben Arous, TUNISIA

###### **Correspondence:** Asma Mehdi - asmaelmahdi245@gmail.com

*Annals of Intensive Care* 2019, **9(Suppl 1)**:P-216

**Introduction**: Problems related to mechanical ventilation weaning are common in intensive care. Many factors can affect weaning process and make it short, difficult or prolonged. We aimed to study weaning conditions and procedures in our department.

**Patients and methods**: Retrospective cohort study conducted in patients admitted in an ICU, from the first January 2017 to the 15th September 2018. We included all patients who have been mechanically ventilated more than 24 h during this period. We used the weaning definition to classify patients- Group no weaning- patients never experienced any separation attempt. Group 1 (short weaning)- First separation attempt resulted in a termination of the weaning process within 24 h (successful separation or early death). Group 2 (difficult weaning)-weaning was terminated after more than 1 day but in less than 1 week after the first separation attempt (successful separation or death). Group 3 (prolonged weaning)- weaning was still not terminated 7 days after the first separation attempt (by success or death).

**Results**: During the study period, 69 patients were included. Median age was 55 years [34 + 66]. Median APACHE II and SOFA score were respectively 21 [14 + 30] and six [4 + 8].

Median rate of hemoglobin and the mean PaO2 FiO2 level at the first separation attempt were respectively 9.8 g/dL [8.5 + 12.15] and 350 ± 70 mmHg. The first separation attempt was carried out essentially as a spontaneous ventilation test (95%) with a median delay of 217 h [60 + 444]. The first spontaneous ventilation test was successfully fulfilled in 43 patients (95%). Fifty percent of patients were classified “short weaning”. Distribution of different groups was summered in table 1. Ten patients required tracheostomy. Median duration of sedation was 96 h [24. 336]. Median mechanically ventilation duration and stay were respectively 9 [3 + 22] and 15 [5 + 30] days. Overall mortality rate was 47%.

**Conclusion**: The first separation attempt was successful in most cases of our patients but the delay was prolonged.

